# Supplementary material for: DMRforPairs: identifying Differentially Methylated Regions between unique samples using array based methylation profiles
Source: BMC Bioinformatics. 2014 May 15;15:141. doi: 10.1186/1471-2105-15-141 (PMC4046028; doi:10.1186/1471-2105-15-141)
Supplement: Additional file 2 — DMRforPairs output for the comparison of A431-MCF7 and NA17018-NA17105. Please start from the HTML files in each folder. Available via the BMC Bioinformatics website. [file 1471-2105-15-141-S2.zip › 1394847754114233_MOESM2_ESM/NA17105M_NA17018F/relevant.html]

DMRforPairs output generated on Tue Nov 19 09:30:45 2013. Identified regions were set to contain at least 4 probes with a maximum distance of 200 bp between individual probes (n=29404). Regions in which median methylation levels (M-values) between the samples differed at least |1.4| (n=4146) (=relevant) were tested for statistical significance (significant: p<0.05; multiple testing adjusted, n=28). The folowing samples were studied: NA17105-M,NA17018-F. Chromosomal positions are indicated in bp. N indicates the number of probes in a region. ID indicates the region ID. Values in the columns per sample indicate median beta values. dM indicates the largest median difference (absolute) between any of the sample pairs. p indicates uncorrected p-value from Mann-Whitney U test (n=2) or Kruskal Wallis test (n>2). p.adj denotes the multiple testing corrected p-value (method:fdr). Gene symbols of overlapping transcripts are listed for the exact region and within a margin of 10000 bp of the region.

| Thumbnail | Chr | Start | End | Links | Length | n | ID | Class | NA17105-M | NA17018-F | Gene.Symbol | dM | p | p.adj |
| --- | --- | --- | --- | --- | --- | --- | --- | --- | --- | --- | --- | --- | --- | --- |
|  | 17 | 40489513 | 40489785 | PDFSTATSENSEMBLUCSC | 273 | 5 | 16579 | gene | 0.004694 | 0.9636622 | Not queried | 14.04 | 7.94e-03 | 0.1589602024 |
|  | 7 | 28452066 | 28452131 | PDFSTATSENSEMBLUCSC | 66 | 4 | 37507 | tss | 0.990161 | 0.0226299 | Not queried | 13.37 | 2.86e-02 | 0.1830867741 |
|  | 7 | 28452066 | 28452289 | PDFSTATSENSEMBLUCSC | 224 | 5 | 36945 | gene;island | 0.989786 | 0.0072924 | Not queried | 11.53 | 7.94e-03 | 0.1589602024 |
|  | 10 | 63809073 | 63809170 | PDFSTATSENSEMBLUCSC | 98 | 6 | 4600 | gene | 0.019912 | 0.9700297 | Not queried | 11.23 | 2.16e-03 | 0.0934794372 |
|  | 12 | 25150402 | 25150626 | PDFSTATSENSEMBLUCSC | 225 | 4 | 9609 | tss | 0.002884 | 0.7435135 | Not queried | 10.95 | 2.86e-02 | 0.1830867741 |
|  | 1 | 110254662 | 110254854 | PDFSTATSENSEMBLUCSC | 193 | 7 | 1810 | tss | 0.011935 | 0.9485183 | Not queried | 10.24 | 5.83e-04 | 0.0548331102 |
|  | 2 | 111875749 | 111876008 | PDFSTATSENSEMBLUCSC | 260 | 5 | 24611 | island | 0.010095 | 0.8522432 | Not queried | 10.18 | 7.94e-03 | 0.1589602024 |
|  | 19 | 36246395 | 36246408 | PDFSTATSENSEMBLUCSC | 14 | 4 | 19993 | gene;island | 0.022923 | 0.9661384 | Not queried | 10.15 | 2.86e-02 | 0.1830867741 |
|  | 2 | 65593761 | 65594021 | PDFSTATSENSEMBLUCSC | 261 | 4 | 22709 | gene | 0.140944 | 0.9824322 | Not queried | 9.82 | 2.86e-02 | 0.1830867741 |
|  | 21 | 38362632 | 38362754 | PDFSTATSENSEMBLUCSC | 123 | 5 | 26540 | tss;island | 0.942682 | 0.0115774 | Not queried | 9.76 | 5.56e-02 | 0.2315877671 |
|  | 19 | 23945472 | 23945761 | PDFSTATSENSEMBLUCSC | 290 | 6 | 20666 | tss | 0.018089 | 0.9405765 | Not queried | 9.72 | 2.16e-03 | 0.0934794372 |
|  | 5 | 110062384 | 110062473 | PDFSTATSENSEMBLUCSC | 90 | 4 | 32204 | tss | 0.512734 | 0.0026809 | Not queried | 9.67 | 2.86e-02 | 0.1830867741 |
|  | 2 | 240196769 | 240197131 | PDFSTATSENSEMBLUCSC | 363 | 4 | 23112 | gene;island | 0.027964 | 0.9387757 | Not queried | 9.66 | 2.86e-02 | 0.1830867741 |
|  | 17 | 38821155 | 38821403 | PDFSTATSENSEMBLUCSC | 249 | 4 | 16566 | gene | 0.011024 | 0.8064254 | Not queried | 9.65 | 2.86e-02 | 0.1830867741 |
|  | 1 | 205818956 | 205819251 | PDFSTATSENSEMBLUCSC | 296 | 4 | 970 | gene | 0.893684 | 0.0145689 | Not queried | 9.41 | 2.86e-02 | 0.1830867741 |
|  | 2 | 37571677 | 37571732 | PDFSTATSENSEMBLUCSC | 56 | 5 | 23306 | tss | 0.019335 | 0.6384695 | Not queried | 9.38 | 7.94e-03 | 0.1589602024 |
|  | 17 | 33759929 | 33759986 | PDFSTATSENSEMBLUCSC | 58 | 4 | 17269 | tss | 0.565305 | 0.0017409 | Not queried | 9.36 | 2.86e-02 | 0.1830867741 |
|  | 10 | 88295210 | 88295591 | PDFSTATSENSEMBLUCSC | 382 | 4 | 5905 | island | 0.020808 | 0.9162930 | Not queried | 9.27 | 2.86e-02 | 0.1830867741 |
|  | 5 | 68628240 | 68628738 | PDFSTATSENSEMBLUCSC | 499 | 5 | 32851 | island | 0.507248 | 0.0045282 | Not queried | 9.25 | 7.94e-03 | 0.1589602024 |
|  | 11 | 3817692 | 3817849 | PDFSTATSENSEMBLUCSC | 158 | 4 | 6480 | gene;tss;island | 0.578252 | 0.0042910 | Not queried | 9.23 | 2.86e-02 | 0.1830867741 |
|  | 6 | 44265644 | 44265696 | PDFSTATSENSEMBLUCSC | 53 | 4 | 35011 | tss;island | 0.003045 | 0.4535841 | Not queried | 9.06 | 2.86e-02 | 0.1830867741 |
|  | 6 | 2891973 | 2892152 | PDFSTATSENSEMBLUCSC | 180 | 5 | 33534 | gene;island | 0.962667 | 0.0549850 | Not queried | 9.06 | 7.94e-03 | 0.1589602024 |
|  | 10 | 97515222 | 97515398 | PDFSTATSENSEMBLUCSC | 177 | 4 | 4689 | gene;tss | 0.003970 | 0.7209471 | Not queried | 9.02 | 2.86e-02 | 0.1830867741 |
|  | 19 | 23945472 | 23945996 | PDFSTATSENSEMBLUCSC | 525 | 8 | 21849 | island | 0.014871 | 0.9137979 | RP11-255H23.2; RPSAP58 (margin: RP11-255H23.2; RPSAP58; ZNF681) | 8.75 | 1.55e-04 | 0.0296603652 |
|  | 5 | 140792511 | 140792700 | PDFSTATSENSEMBLUCSC | 190 | 5 | 32381 | tss | 0.467823 | 0.0036103 | Not queried | 8.64 | 7.94e-03 | 0.1589602024 |
|  | 5 | 178986620 | 178986906 | PDFSTATSENSEMBLUCSC | 287 | 5 | 31933 | gene;island | 0.583700 | 0.0055649 | Not queried | 8.48 | 7.94e-03 | 0.1589602024 |
|  | 17 | 37894258 | 37894413 | PDFSTATSENSEMBLUCSC | 156 | 4 | 16555 | gene | 0.018304 | 0.7741049 | Not queried | 8.41 | 2.86e-02 | 0.1830867741 |
|  | 12 | 49726500 | 49726893 | PDFSTATSENSEMBLUCSC | 394 | 4 | 9118 | gene;island | 0.061202 | 0.9289986 | Not queried | 8.39 | 2.86e-02 | 0.1830867741 |
|  | 11 | 120434710 | 120435056 | PDFSTATSENSEMBLUCSC | 347 | 4 | 8896 | island | 0.024088 | 0.9753791 | Not queried | 8.26 | 2.86e-02 | 0.1830867741 |
|  | 17 | 37894014 | 37894413 | PDFSTATSENSEMBLUCSC | 400 | 7 | 17316 | tss | 0.010675 | 0.7647515 | Not queried | 8.15 | 5.83e-04 | 0.0548331102 |
|  | 19 | 617133 | 617432 | PDFSTATSENSEMBLUCSC | 300 | 4 | 21170 | island | 0.033967 | 0.7497014 | Not queried | 8.12 | 2.86e-02 | 0.1830867741 |
|  | 10 | 128593922 | 128594144 | PDFSTATSENSEMBLUCSC | 223 | 5 | 6246 | island | 0.066768 | 0.9325082 | Not queried | 8.05 | 1.59e-02 | 0.1830867741 |
|  | 4 | 123747447 | 123747750 | PDFSTATSENSEMBLUCSC | 304 | 7 | 30581 | tss;island | 0.012916 | 0.7316144 | Not queried | 7.95 | 6.99e-03 | 0.1589602024 |
|  | 2 | 37571677 | 37571901 | PDFSTATSENSEMBLUCSC | 225 | 6 | 24223 | island | 0.022218 | 0.6348199 | Not queried | 7.91 | 2.16e-03 | 0.0934794372 |
|  | 1 | 147012684 | 147012802 | PDFSTATSENSEMBLUCSC | 119 | 4 | 1894 | tss | 0.007150 | 0.5971200 | Not queried | 7.86 | 5.71e-02 | 0.2315877671 |
|  | 10 | 18629497 | 18629602 | PDFSTATSENSEMBLUCSC | 106 | 4 | 4974 | tss | 0.178035 | 0.9308506 | Not queried | 7.83 | 2.00e-01 | 0.3826488233 |
|  | 22 | 46770063 | 46770440 | PDFSTATSENSEMBLUCSC | 378 | 4 | 26913 | gene;island | 0.024020 | 0.4929873 | Not queried | 7.80 | 2.86e-02 | 0.1830867741 |
|  | 15 | 83477608 | 83477965 | PDFSTATSENSEMBLUCSC | 358 | 5 | 13860 | tss;island | 0.002033 | 0.4617145 | Not queried | 7.76 | 3.17e-02 | 0.1866936846 |
|  | 2 | 158731577 | 158731796 | PDFSTATSENSEMBLUCSC | 220 | 4 | 22904 | gene | 0.001699 | 0.3110054 | Not queried | 7.70 | 2.86e-02 | 0.1830867741 |
|  | 8 | 143407432 | 143407817 | PDFSTATSENSEMBLUCSC | 386 | 4 | 39460 | gene;island | 0.923908 | 0.0347564 | Not queried | 7.61 | 2.86e-02 | 0.1830867741 |
|  | 12 | 54410657 | 54410727 | PDFSTATSENSEMBLUCSC | 71 | 5 | 9158 | gene | 0.467615 | 0.0057533 | Not queried | 7.50 | 7.94e-03 | 0.1589602024 |
|  | 12 | 13256639 | 13256869 | PDFSTATSENSEMBLUCSC | 231 | 4 | 9581 | tss;island | 0.008945 | 0.6121293 | Not queried | 7.45 | 1.14e-01 | 0.3123457953 |
|  | 4 | 81119178 | 81119473 | PDFSTATSENSEMBLUCSC | 296 | 6 | 30034 | gene;island | 0.034875 | 0.8729079 | Not queried | 7.42 | 4.33e-03 | 0.1459191215 |
|  | 21 | 47604052 | 47604361 | PDFSTATSENSEMBLUCSC | 310 | 4 | 26473 | gene;island | 0.007180 | 0.4704738 | Not queried | 7.32 | 2.86e-02 | 0.1830867741 |
|  | 7 | 156741514 | 156741808 | PDFSTATSENSEMBLUCSC | 295 | 5 | 38018 | tss;island | 0.748953 | 0.0208939 | Not queried | 7.13 | 5.56e-02 | 0.2315877671 |
|  | 19 | 50861774 | 50862121 | PDFSTATSENSEMBLUCSC | 348 | 5 | 20174 | gene;island | 0.007593 | 0.4888662 | Not queried | 7.12 | 7.94e-03 | 0.1589602024 |
|  | 8 | 58055876 | 58056175 | PDFSTATSENSEMBLUCSC | 300 | 4 | 40319 | island | 0.152164 | 0.9166491 | Not queried | 7.06 | 5.71e-02 | 0.2315877671 |
|  | 5 | 148521450 | 148521623 | PDFSTATSENSEMBLUCSC | 174 | 4 | 31856 | gene;island | 0.589446 | 0.0117224 | Not queried | 7.04 | 2.86e-02 | 0.1830867741 |
|  | 10 | 38692145 | 38692317 | PDFSTATSENSEMBLUCSC | 173 | 4 | 5038 | tss;island | 0.266319 | 0.0129665 | Not queried | 6.99 | 2.00e-01 | 0.3826488233 |
|  | 6 | 32551749 | 32552670 | PDFSTATSENSEMBLUCSC | 922 | 17 | 34122 | gene;island | 0.062812 | 0.9760723 | HLA-DRB1 (margin: HLA-DRB1) | 6.99 | 8.31e-08 | 0.0000689340 |
|  | 1 | 207842833 | 207843084 | PDFSTATSENSEMBLUCSC | 252 | 4 | 979 | gene;island | 0.570939 | 0.0393072 | Not queried | 6.96 | 2.86e-02 | 0.1830867741 |
|  | 1 | 203320386 | 203320541 | PDFSTATSENSEMBLUCSC | 156 | 4 | 2192 | tss | 0.337663 | 0.0068693 | Not queried | 6.95 | 2.86e-02 | 0.1830867741 |
|  | 16 | 54965084 | 54965100 | PDFSTATSENSEMBLUCSC | 17 | 4 | 15238 | tss | 0.407386 | 0.0076154 | Not queried | 6.93 | 2.86e-02 | 0.1830867741 |
|  | 16 | 2908157 | 2908245 | PDFSTATSENSEMBLUCSC | 89 | 4 | 15623 | island | 0.583395 | 0.0233641 | Not queried | 6.88 | 2.86e-02 | 0.1830867741 |
|  | 1 | 68517177 | 68517273 | PDFSTATSENSEMBLUCSC | 97 | 4 | 1687 | tss;island | 0.057630 | 0.8188631 | Not queried | 6.84 | 2.86e-02 | 0.1830867741 |
|  | 1 | 67772896 | 67773044 | PDFSTATSENSEMBLUCSC | 149 | 5 | 1683 | tss | 0.006256 | 0.5565591 | Not queried | 6.84 | 7.94e-03 | 0.1589602024 |
|  | 16 | 54965084 | 54965142 | PDFSTATSENSEMBLUCSC | 59 | 5 | 15931 | island | 0.395572 | 0.0097971 | Not queried | 6.81 | 7.94e-03 | 0.1589602024 |
|  | 7 | 27170241 | 27170394 | PDFSTATSENSEMBLUCSC | 154 | 4 | 36933 | gene | 0.906976 | 0.1607960 | Not queried | 6.75 | 2.86e-02 | 0.1830867741 |
|  | 18 | 77724395 | 77724453 | PDFSTATSENSEMBLUCSC | 59 | 4 | 19338 | tss;island | 0.004452 | 0.2069730 | Not queried | 6.70 | 2.86e-02 | 0.1830867741 |
|  | 5 | 114516315 | 114516422 | PDFSTATSENSEMBLUCSC | 108 | 4 | 32223 | tss | 0.410756 | 0.0354266 | Not queried | 6.69 | 6.86e-01 | 0.7223604052 |
|  | 5 | 172664995 | 172665149 | PDFSTATSENSEMBLUCSC | 155 | 4 | 33408 | island | 0.019835 | 0.6341242 | Not queried | 6.67 | 2.86e-02 | 0.1830867741 |
|  | 6 | 32557321 | 32557478 | PDFSTATSENSEMBLUCSC | 158 | 5 | 34123 | gene | 0.012535 | 0.4154388 | Not queried | 6.63 | 5.56e-02 | 0.2315877671 |
|  | 2 | 74725040 | 74725369 | PDFSTATSENSEMBLUCSC | 330 | 4 | 22743 | gene;island | 0.018271 | 0.4085096 | Not queried | 6.63 | 2.86e-02 | 0.1830867741 |
|  | 6 | 33043072 | 33043220 | PDFSTATSENSEMBLUCSC | 149 | 4 | 34888 | tss | 0.002132 | 0.3019556 | Not queried | 6.63 | 3.43e-01 | 0.4672865596 |
|  | 5 | 74162621 | 74162924 | PDFSTATSENSEMBLUCSC | 304 | 7 | 32127 | tss | 0.022193 | 0.9340233 | Not queried | 6.62 | 5.83e-04 | 0.0548331102 |
|  | 6 | 30954872 | 30955142 | PDFSTATSENSEMBLUCSC | 271 | 4 | 33845 | gene | 0.000792 | 0.0691426 | Not queried | 6.59 | 2.86e-02 | 0.1830867741 |
|  | 22 | 45704902 | 45705042 | PDFSTATSENSEMBLUCSC | 141 | 6 | 27221 | tss;island | 0.078227 | 0.9417578 | Not queried | 6.59 | 4.11e-02 | 0.2180389943 |
|  | 1 | 38461540 | 38461896 | PDFSTATSENSEMBLUCSC | 357 | 4 | 3081 | island | 0.576984 | 0.0144314 | Not queried | 6.56 | 2.86e-02 | 0.1830867741 |
|  | 10 | 38645346 | 38645450 | PDFSTATSENSEMBLUCSC | 105 | 4 | 4560 | gene;island | 0.348107 | 0.0039324 | Not queried | 6.56 | 2.86e-02 | 0.1830867741 |
|  | 10 | 64565642 | 64565772 | PDFSTATSENSEMBLUCSC | 131 | 4 | 4601 | gene;island | 0.017740 | 0.6045827 | Not queried | 6.55 | 2.86e-02 | 0.1830867741 |
|  | 6 | 31322577 | 31322996 | PDFSTATSENSEMBLUCSC | 420 | 5 | 33867 | gene;island | 0.012559 | 0.4097707 | Not queried | 6.54 | 1.51e-01 | 0.3618000441 |
|  | 17 | 53800225 | 53800554 | PDFSTATSENSEMBLUCSC | 330 | 4 | 17519 | tss | 0.455268 | 0.0237989 | Not queried | 6.47 | 1.14e-01 | 0.3123457953 |
|  | 7 | 4751840 | 4752170 | PDFSTATSENSEMBLUCSC | 331 | 7 | 36842 | gene | 0.486500 | 0.9872781 | Not queried | 6.45 | 5.83e-04 | 0.0548331102 |
|  | 11 | 504784 | 504937 | PDFSTATSENSEMBLUCSC | 154 | 5 | 6377 | gene;island | 0.575586 | 0.0111722 | Not queried | 6.45 | 7.94e-03 | 0.1589602024 |
|  | 17 | 53800041 | 53800554 | PDFSTATSENSEMBLUCSC | 514 | 5 | 16701 | gene | 0.492922 | 0.0387231 | Not queried | 6.43 | 3.10e-01 | 0.4672865596 |
|  | 8 | 86350889 | 86351018 | PDFSTATSENSEMBLUCSC | 130 | 5 | 39795 | tss | 0.013715 | 0.6743677 | Not queried | 6.42 | 7.94e-03 | 0.1589602024 |
|  | 19 | 11531294 | 11531469 | PDFSTATSENSEMBLUCSC | 176 | 4 | 20472 | tss | 0.016321 | 0.3556879 | Not queried | 6.41 | 2.86e-02 | 0.1830867741 |
|  | 17 | 1617291 | 1617465 | PDFSTATSENSEMBLUCSC | 175 | 5 | 16993 | tss | 0.009562 | 0.4183915 | Not queried | 6.40 | 7.94e-03 | 0.1589602024 |
|  | 16 | 51189801 | 51190201 | PDFSTATSENSEMBLUCSC | 401 | 5 | 15921 | island | 0.321934 | 0.0051882 | Not queried | 6.39 | 5.56e-02 | 0.2315877671 |
|  | 10 | 73136913 | 73137135 | PDFSTATSENSEMBLUCSC | 223 | 4 | 5828 | island | 0.548947 | 0.0244379 | Not queried | 6.38 | 2.86e-02 | 0.1830867741 |
|  | 22 | 24891129 | 24891452 | PDFSTATSENSEMBLUCSC | 324 | 8 | 27021 | tss;island | 0.256461 | 0.9320004 | Not queried | 6.36 | 2.07e-02 | 0.1830867741 |
|  | 19 | 44763979 | 44764380 | PDFSTATSENSEMBLUCSC | 402 | 5 | 22106 | island | 0.007470 | 0.5008962 | Not queried | 6.33 | 7.94e-03 | 0.1589602024 |
|  | 1 | 108023249 | 108023482 | PDFSTATSENSEMBLUCSC | 234 | 5 | 660 | gene;island | 0.456695 | 0.0105990 | Not queried | 6.31 | 7.94e-03 | 0.1589602024 |
|  | 8 | 86350889 | 86351195 | PDFSTATSENSEMBLUCSC | 307 | 7 | 40432 | island | 0.019367 | 0.5792245 | Not queried | 6.28 | 1.17e-03 | 0.0833132385 |
|  | 5 | 1594579 | 1595048 | PDFSTATSENSEMBLUCSC | 470 | 9 | 32635 | island | 0.495912 | 0.0131407 | CTD-2012J19.3; SDHAP3 (margin: CTD-2012J19.3; CTD-2012J19.2; SDHAP3; RP11-43F13.1) | 6.27 | 1.65e-04 | 0.0296603652 |
|  | 17 | 1617102 | 1617465 | PDFSTATSENSEMBLUCSC | 364 | 6 | 16318 | gene;island | 0.011185 | 0.4151169 | Not queried | 6.27 | 2.16e-03 | 0.0934794372 |
|  | 18 | 21719263 | 21719433 | PDFSTATSENSEMBLUCSC | 171 | 5 | 19210 | tss | 0.013548 | 0.5238099 | Not queried | 6.25 | 1.51e-01 | 0.3618000441 |
|  | 15 | 91427821 | 91428056 | PDFSTATSENSEMBLUCSC | 236 | 5 | 13478 | gene;tss;island | 0.012881 | 0.1703054 | Not queried | 6.23 | 2.22e-01 | 0.3935758299 |
|  | 6 | 31164743 | 31164994 | PDFSTATSENSEMBLUCSC | 252 | 12 | 34803 | tss;island | 0.529376 | 0.0106260 | HCG27; XXbac-BPG299F13.17 (margin: HCG27; XXbac-BPG299F13.17; XXbac-BPG299F13.14) | 6.22 | 2.96e-06 | 0.0017522235 |
|  | 13 | 25506131 | 25506383 | PDFSTATSENSEMBLUCSC | 253 | 4 | 11107 | gene;island | 0.781167 | 0.1542156 | Not queried | 6.22 | 1.14e-01 | 0.3123457953 |
|  | 18 | 74499372 | 74499584 | PDFSTATSENSEMBLUCSC | 213 | 5 | 19581 | island | 0.037555 | 0.5231263 | Not queried | 6.19 | 3.17e-02 | 0.1866936846 |
|  | 3 | 71833543 | 71833907 | PDFSTATSENSEMBLUCSC | 365 | 5 | 27860 | gene;island | 0.092742 | 0.0041900 | Not queried | 6.18 | 9.52e-02 | 0.3104222821 |
|  | 16 | 2286370 | 2286786 | PDFSTATSENSEMBLUCSC | 417 | 4 | 15599 | island | 0.039669 | 0.7518968 | Not queried | 6.18 | 1.14e-01 | 0.3123457953 |
|  | 8 | 99984350 | 99984763 | PDFSTATSENSEMBLUCSC | 414 | 4 | 40489 | island | 0.170208 | 0.9603202 | Not queried | 6.18 | 5.71e-02 | 0.2315877671 |
|  | 1 | 205818956 | 205819609 | PDFSTATSENSEMBLUCSC | 654 | 8 | 4138 | island | 0.502067 | 0.0239484 | Not queried | 6.17 | 4.66e-03 | 0.1498346615 |
|  | 17 | 1509883 | 1510080 | PDFSTATSENSEMBLUCSC | 198 | 5 | 16316 | gene;island | 0.025488 | 0.5173631 | Not queried | 6.16 | 7.94e-03 | 0.1589602024 |
|  | 11 | 60534911 | 60535066 | PDFSTATSENSEMBLUCSC | 156 | 4 | 6622 | gene;island | 0.780722 | 0.0675438 | Not queried | 6.15 | 2.86e-02 | 0.1830867741 |
|  | 5 | 74162458 | 74162924 | PDFSTATSENSEMBLUCSC | 467 | 9 | 32881 | island | 0.023500 | 0.6888514 | Not queried | 6.14 | 1.23e-03 | 0.0862637156 |
|  | 15 | 74528522 | 74528606 | PDFSTATSENSEMBLUCSC | 85 | 4 | 13791 | tss | 0.009907 | 0.3859442 | Not queried | 6.11 | 2.86e-02 | 0.1830867741 |
|  | 22 | 42078217 | 42078723 | PDFSTATSENSEMBLUCSC | 507 | 7 | 26883 | gene;island | 0.499550 | 0.0106589 | Not queried | 6.10 | 5.83e-04 | 0.0548331102 |
|  | 6 | 32031395 | 32031820 | PDFSTATSENSEMBLUCSC | 426 | 4 | 34027 | gene | 0.036104 | 0.6808349 | Not queried | 6.09 | 2.00e-01 | 0.3826488233 |
|  | 17 | 8056950 | 8057058 | PDFSTATSENSEMBLUCSC | 109 | 4 | 17110 | tss;island | 0.006821 | 0.3169140 | Not queried | 6.08 | 1.14e-01 | 0.3123457953 |
|  | 2 | 219848258 | 219848580 | PDFSTATSENSEMBLUCSC | 323 | 5 | 23022 | gene;island | 0.287540 | 0.0101754 | Not queried | 6.07 | 1.51e-01 | 0.3618000441 |
|  | 8 | 25902284 | 25902611 | PDFSTATSENSEMBLUCSC | 328 | 6 | 40170 | island | 0.562687 | 0.0355912 | Not queried | 6.06 | 1.52e-02 | 0.1830867741 |
|  | 5 | 1594676 | 1595048 | PDFSTATSENSEMBLUCSC | 373 | 8 | 31971 | tss | 0.495503 | 0.0152052 | Not queried | 6.05 | 6.22e-04 | 0.0548331102 |
|  | 19 | 5838729 | 5838999 | PDFSTATSENSEMBLUCSC | 271 | 7 | 19757 | gene | 0.589162 | 0.0235604 | Not queried | 6.03 | 2.62e-02 | 0.1830867741 |
|  | 7 | 96654152 | 96654198 | PDFSTATSENSEMBLUCSC | 47 | 4 | 37735 | tss | 0.007670 | 0.3856614 | Not queried | 6.03 | 2.86e-02 | 0.1830867741 |
|  | 7 | 16505589 | 16505664 | PDFSTATSENSEMBLUCSC | 76 | 5 | 37452 | tss | 0.606578 | 0.0159037 | Not queried | 6.02 | 3.17e-02 | 0.1866936846 |
|  | 17 | 48049952 | 48049979 | PDFSTATSENSEMBLUCSC | 28 | 4 | 16677 | gene;tss;island | 0.014207 | 0.5747780 | Not queried | 6.01 | 2.86e-02 | 0.1830867741 |
|  | 10 | 23216637 | 23216847 | PDFSTATSENSEMBLUCSC | 211 | 6 | 4986 | tss | 0.016638 | 0.3911388 | Not queried | 6.00 | 4.11e-02 | 0.2180389943 |
|  | 11 | 2321770 | 2322050 | PDFSTATSENSEMBLUCSC | 281 | 9 | 6453 | gene;tss | 0.007924 | 0.2786334 | Not queried | 5.98 | 1.85e-03 | 0.0934794372 |
|  | 22 | 39784481 | 39784982 | PDFSTATSENSEMBLUCSC | 502 | 5 | 27504 | island | 0.121670 | 0.0073780 | Not queried | 5.96 | 3.17e-02 | 0.1866936846 |
|  | 5 | 178986131 | 178986412 | PDFSTATSENSEMBLUCSC | 282 | 4 | 31932 | gene;tss;island | 0.542875 | 0.0575700 | Not queried | 5.94 | 2.00e-01 | 0.3826488233 |
|  | 1 | 31231214 | 31231488 | PDFSTATSENSEMBLUCSC | 275 | 4 | 1442 | tss | 0.110274 | 0.8751956 | Not queried | 5.94 | 2.86e-02 | 0.1830867741 |
|  | 1 | 153283628 | 153283994 | PDFSTATSENSEMBLUCSC | 367 | 4 | 1940 | tss | 0.010301 | 0.2158472 | Not queried | 5.94 | 5.71e-02 | 0.2315877671 |
|  | 8 | 37605552 | 37605978 | PDFSTATSENSEMBLUCSC | 427 | 5 | 39646 | tss | 0.599871 | 0.0299448 | Not queried | 5.93 | 1.51e-01 | 0.3618000441 |
|  | 7 | 38350921 | 38351226 | PDFSTATSENSEMBLUCSC | 306 | 5 | 38422 | island | 0.606805 | 0.0251554 | Not queried | 5.91 | 3.17e-02 | 0.1866936846 |
|  | 1 | 24152579 | 24152774 | PDFSTATSENSEMBLUCSC | 196 | 4 | 1350 | tss | 0.014213 | 0.5286260 | Not queried | 5.90 | 5.71e-02 | 0.2315877671 |
|  | 1 | 19970204 | 19970337 | PDFSTATSENSEMBLUCSC | 134 | 4 | 284 | gene;tss;island | 0.019627 | 0.4917562 | Not queried | 5.88 | 2.86e-02 | 0.1830867741 |
|  | 17 | 37856133 | 37856230 | PDFSTATSENSEMBLUCSC | 98 | 4 | 17314 | tss | 0.455261 | 0.0266922 | Not queried | 5.86 | 3.43e-01 | 0.4672865596 |
|  | 4 | 86851225 | 86851425 | PDFSTATSENSEMBLUCSC | 201 | 4 | 30048 | gene | 0.003711 | 0.4119328 | Not queried | 5.84 | 5.71e-02 | 0.2315877671 |
|  | 1 | 67772896 | 67773175 | PDFSTATSENSEMBLUCSC | 280 | 8 | 3365 | island | 0.009940 | 0.3705543 | Not queried | 5.82 | 6.22e-04 | 0.0548331102 |
|  | 1 | 167090972 | 167091161 | PDFSTATSENSEMBLUCSC | 190 | 4 | 873 | gene;island | 0.012627 | 0.6329090 | Not queried | 5.81 | 3.43e-01 | 0.4672865596 |
|  | 11 | 68924577 | 68924956 | PDFSTATSENSEMBLUCSC | 380 | 5 | 8586 | island | 0.040708 | 0.7068889 | Not queried | 5.81 | 3.17e-02 | 0.1866936846 |
|  | 17 | 38501397 | 38501773 | PDFSTATSENSEMBLUCSC | 377 | 4 | 16564 | gene;island | 0.026062 | 0.4687828 | Not queried | 5.81 | 2.86e-02 | 0.1830867741 |
|  | 19 | 11531294 | 11531519 | PDFSTATSENSEMBLUCSC | 226 | 5 | 19840 | gene;island | 0.017772 | 0.0795056 | Not queried | 5.78 | 5.56e-02 | 0.2315877671 |
|  | 15 | 60289239 | 60289532 | PDFSTATSENSEMBLUCSC | 294 | 4 | 14187 | island | 0.266093 | 0.0215595 | Not queried | 5.78 | 5.71e-02 | 0.2315877671 |
|  | 6 | 74104388 | 74104600 | PDFSTATSENSEMBLUCSC | 213 | 4 | 34388 | gene;island | 0.985075 | 0.4831894 | Not queried | 5.77 | 2.86e-02 | 0.1830867741 |
|  | 17 | 40575462 | 40575582 | PDFSTATSENSEMBLUCSC | 121 | 6 | 17362 | tss | 0.011147 | 0.3610249 | Not queried | 5.77 | 2.16e-03 | 0.0934794372 |
|  | 19 | 3480363 | 3480672 | PDFSTATSENSEMBLUCSC | 310 | 5 | 21330 | island | 0.848780 | 0.1021668 | Not queried | 5.77 | 7.94e-03 | 0.1589602024 |
|  | 1 | 40782991 | 40783264 | PDFSTATSENSEMBLUCSC | 274 | 7 | 1535 | tss | 0.020585 | 0.5042090 | Not queried | 5.76 | 1.75e-02 | 0.1830867741 |
|  | 3 | 194119861 | 194120150 | PDFSTATSENSEMBLUCSC | 290 | 4 | 29788 | island | 0.762812 | 0.0474323 | Not queried | 5.76 | 2.00e-01 | 0.3826488233 |
|  | 16 | 1133074 | 1133378 | PDFSTATSENSEMBLUCSC | 305 | 5 | 15515 | island | 0.063503 | 0.6648238 | Not queried | 5.76 | 5.56e-02 | 0.2315877671 |
|  | 19 | 13124604 | 13125281 | PDFSTATSENSEMBLUCSC | 678 | 7 | 19875 | gene;island | 0.008964 | 0.2293308 | Not queried | 5.76 | 1.17e-03 | 0.0833132385 |
|  | 22 | 50064736 | 50065058 | PDFSTATSENSEMBLUCSC | 323 | 4 | 27605 | island | 0.501878 | 0.0314037 | Not queried | 5.76 | 2.86e-02 | 0.1830867741 |
|  | 3 | 193987426 | 193987757 | PDFSTATSENSEMBLUCSC | 332 | 4 | 29785 | island | 0.022605 | 0.5100914 | Not queried | 5.75 | 5.71e-02 | 0.2315877671 |
|  | 7 | 155174991 | 155175340 | PDFSTATSENSEMBLUCSC | 350 | 4 | 39065 | island | 0.486428 | 0.0273886 | Not queried | 5.75 | 5.71e-02 | 0.2315877671 |
|  | 6 | 114177329 | 114177535 | PDFSTATSENSEMBLUCSC | 207 | 4 | 35184 | tss;island | 0.001654 | 0.0310833 | Not queried | 5.73 | 2.86e-02 | 0.1830867741 |
|  | 20 | 55841869 | 55842071 | PDFSTATSENSEMBLUCSC | 203 | 8 | 25840 | tss;island | 0.456524 | 0.0190137 | RP4-813D12.3; BMP7 (margin: RP4-813D12.3; BMP7) | 5.73 | 1.55e-04 | 0.0296603652 |
|  | 10 | 70231928 | 70232188 | PDFSTATSENSEMBLUCSC | 261 | 5 | 5113 | tss;island | 0.018447 | 0.6309838 | Not queried | 5.73 | 5.56e-02 | 0.2315877671 |
|  | 17 | 406249 | 406501 | PDFSTATSENSEMBLUCSC | 253 | 4 | 17796 | island | 0.466888 | 0.9579900 | Not queried | 5.71 | 2.86e-02 | 0.1830867741 |
|  | 3 | 160475035 | 160475128 | PDFSTATSENSEMBLUCSC | 94 | 4 | 28017 | gene;island | 0.751198 | 0.0991635 | Not queried | 5.66 | 2.86e-02 | 0.1830867741 |
|  | 6 | 101846650 | 101846805 | PDFSTATSENSEMBLUCSC | 156 | 7 | 35143 | tss | 0.118637 | 0.9476104 | Not queried | 5.66 | 7.28e-02 | 0.2713481487 |
|  | 2 | 232260116 | 232260305 | PDFSTATSENSEMBLUCSC | 190 | 4 | 23939 | tss;island | 0.010783 | 0.2456547 | Not queried | 5.65 | 1.14e-01 | 0.3123457953 |
|  | 12 | 9217390 | 9217769 | PDFSTATSENSEMBLUCSC | 380 | 8 | 9556 | tss | 0.024435 | 0.4924536 | A2M-AS1; LINC00612 (margin: A2M-AS1; LINC00612; A2M) | 5.63 | 3.11e-04 | 0.0495606957 |
|  | 7 | 37959686 | 37959966 | PDFSTATSENSEMBLUCSC | 281 | 4 | 37543 | tss;island | 0.112338 | 0.8866424 | Not queried | 5.63 | 2.00e-01 | 0.3826488233 |
|  | 5 | 54528100 | 54528369 | PDFSTATSENSEMBLUCSC | 270 | 4 | 31615 | gene;island | 0.344857 | 0.0152431 | Not queried | 5.60 | 1.14e-01 | 0.3123457953 |
|  | 5 | 54281198 | 54281362 | PDFSTATSENSEMBLUCSC | 165 | 4 | 31614 | gene | 0.037765 | 0.6573436 | Not queried | 5.59 | 2.86e-02 | 0.1830867741 |
|  | 5 | 134783075 | 134783273 | PDFSTATSENSEMBLUCSC | 199 | 4 | 32304 | tss | 0.048225 | 0.4280515 | Not queried | 5.58 | 2.86e-02 | 0.1830867741 |
|  | 5 | 75380782 | 75381070 | PDFSTATSENSEMBLUCSC | 289 | 5 | 31650 | gene;island | 0.262406 | 0.0175304 | Not queried | 5.57 | 3.17e-02 | 0.1866936846 |
|  | 6 | 47253910 | 47254169 | PDFSTATSENSEMBLUCSC | 260 | 5 | 34361 | gene | 0.155644 | 0.9598674 | Not queried | 5.56 | 3.17e-02 | 0.1866936846 |
|  | 2 | 176968584 | 176968920 | PDFSTATSENSEMBLUCSC | 337 | 4 | 24895 | island | 0.024754 | 0.4158494 | Not queried | 5.54 | 2.86e-02 | 0.1830867741 |
|  | 11 | 58673453 | 58673615 | PDFSTATSENSEMBLUCSC | 163 | 4 | 8298 | island | 0.854440 | 0.0518914 | Not queried | 5.53 | 5.71e-02 | 0.2315877671 |
|  | 5 | 76010444 | 76010658 | PDFSTATSENSEMBLUCSC | 215 | 4 | 32134 | tss;island | 0.281026 | 0.0058946 | Not queried | 5.53 | 2.86e-02 | 0.1830867741 |
|  | 8 | 22223852 | 22224272 | PDFSTATSENSEMBLUCSC | 421 | 6 | 39585 | tss;island | 0.965579 | 0.3667395 | Not queried | 5.53 | 9.31e-02 | 0.3104222821 |
|  | 13 | 114786825 | 114786904 | PDFSTATSENSEMBLUCSC | 80 | 4 | 11307 | gene;island | 0.976869 | 0.5728202 | Not queried | 5.53 | 2.86e-02 | 0.1830867741 |
|  | 10 | 108924366 | 108924560 | PDFSTATSENSEMBLUCSC | 195 | 4 | 5351 | tss;island | 0.571817 | 0.0370838 | Not queried | 5.53 | 2.86e-02 | 0.1830867741 |
|  | 7 | 114561804 | 114562063 | PDFSTATSENSEMBLUCSC | 260 | 5 | 37847 | tss;island | 0.565096 | 0.9722679 | Not queried | 5.51 | 3.10e-01 | 0.4672865596 |
|  | 1 | 1104257 | 1104366 | PDFSTATSENSEMBLUCSC | 110 | 4 | 1141 | tss | 0.011158 | 0.3980179 | Not queried | 5.51 | 2.00e-01 | 0.3826488233 |
|  | 1 | 34629098 | 34629559 | PDFSTATSENSEMBLUCSC | 462 | 5 | 408 | gene;island | 0.024379 | 0.3808904 | Not queried | 5.48 | 1.59e-02 | 0.1830867741 |
|  | 22 | 18632404 | 18632618 | PDFSTATSENSEMBLUCSC | 215 | 5 | 26949 | tss | 0.039747 | 0.6642032 | Not queried | 5.45 | 1.59e-02 | 0.1830867741 |
|  | 1 | 59043173 | 59043370 | PDFSTATSENSEMBLUCSC | 198 | 6 | 1654 | tss | 0.408430 | 0.9753593 | Not queried | 5.44 | 2.40e-01 | 0.4133265075 |
|  | 5 | 218073 | 218200 | PDFSTATSENSEMBLUCSC | 128 | 4 | 31498 | gene;tss;island | 0.120301 | 0.0076117 | Not queried | 5.44 | 1.14e-01 | 0.3123457953 |
|  | 5 | 140578896 | 140579303 | PDFSTATSENSEMBLUCSC | 408 | 7 | 32366 | tss | 0.012741 | 0.9148090 | Not queried | 5.42 | 1.28e-01 | 0.3397986247 |
|  | 19 | 11353961 | 11354401 | PDFSTATSENSEMBLUCSC | 441 | 5 | 19832 | gene;island | 0.576828 | 0.1551352 | Not queried | 5.40 | 4.21e-01 | 0.5404252807 |
|  | 6 | 29893926 | 29894341 | PDFSTATSENSEMBLUCSC | 416 | 16 | 34757 | tss;island | 0.021777 | 0.4924359 | HLA-K; HCG4B (margin: HLA-K; HLA-U; HCG4B) | 5.39 | 4.63e-07 | 0.0003195879 |
|  | 19 | 12876846 | 12877188 | PDFSTATSENSEMBLUCSC | 343 | 4 | 19866 | gene;island | 0.031259 | 0.4443288 | Not queried | 5.39 | 2.86e-02 | 0.1830867741 |
|  | 12 | 9217390 | 9218039 | PDFSTATSENSEMBLUCSC | 650 | 11 | 10235 | island | 0.025855 | 0.4992826 | Not queried | 5.38 | 1.04e-03 | 0.0821403610 |
|  | 15 | 74421523 | 74421584 | PDFSTATSENSEMBLUCSC | 62 | 4 | 13415 | gene | 0.183881 | 0.0119519 | Not queried | 5.38 | 2.86e-02 | 0.1830867741 |
|  | 5 | 8457548 | 8458089 | PDFSTATSENSEMBLUCSC | 542 | 6 | 32684 | island | 0.011357 | 0.2245731 | Not queried | 5.37 | 2.16e-03 | 0.0934794372 |
|  | 18 | 74535840 | 74536226 | PDFSTATSENSEMBLUCSC | 387 | 6 | 19582 | island | 0.585095 | 0.0504722 | Not queried | 5.36 | 1.32e-01 | 0.3397986247 |
|  | 18 | 74535840 | 74536113 | PDFSTATSENSEMBLUCSC | 274 | 5 | 19328 | tss | 0.556322 | 0.0373539 | Not queried | 5.36 | 9.52e-02 | 0.3104222821 |
|  | 5 | 140792511 | 140792992 | PDFSTATSENSEMBLUCSC | 482 | 8 | 31832 | gene;island | 0.301042 | 0.0068122 | Not queried | 5.35 | 1.04e-02 | 0.1806165940 |
|  | 1 | 247802703 | 247803166 | PDFSTATSENSEMBLUCSC | 464 | 7 | 4420 | island | 0.025863 | 0.4915442 | Not queried | 5.34 | 5.30e-02 | 0.2315877671 |
|  | 22 | 45809596 | 45810043 | PDFSTATSENSEMBLUCSC | 448 | 8 | 26904 | gene | 0.156687 | 0.0134295 | Not queried | 5.34 | 6.99e-03 | 0.1589602024 |
|  | 6 | 32181764 | 32182326 | PDFSTATSENSEMBLUCSC | 563 | 6 | 34093 | gene | 0.027788 | 0.4495004 | Not queried | 5.32 | 1.32e-01 | 0.3397986247 |
|  | 17 | 40715222 | 40715281 | PDFSTATSENSEMBLUCSC | 60 | 5 | 16580 | gene;island | 0.071585 | 0.8278033 | Not queried | 5.31 | 7.94e-03 | 0.1589602024 |
|  | 7 | 2316876 | 2317124 | PDFSTATSENSEMBLUCSC | 249 | 6 | 36798 | gene;island | 0.288396 | 0.7521380 | Not queried | 5.31 | 1.52e-02 | 0.1830867741 |
|  | 10 | 134150451 | 134150760 | PDFSTATSENSEMBLUCSC | 310 | 8 | 4862 | gene | 0.008994 | 0.1272652 | Not queried | 5.30 | 1.48e-02 | 0.1830867741 |
|  | 10 | 134150451 | 134150581 | PDFSTATSENSEMBLUCSC | 131 | 6 | 5448 | tss | 0.011755 | 0.1272652 | Not queried | 5.30 | 6.49e-02 | 0.2477578846 |
|  | 10 | 93334974 | 93335196 | PDFSTATSENSEMBLUCSC | 223 | 4 | 4672 | gene | 0.001477 | 0.0341800 | Not queried | 5.30 | 5.71e-02 | 0.2315877671 |
|  | 4 | 46126245 | 46126448 | PDFSTATSENSEMBLUCSC | 204 | 6 | 30371 | tss | 0.139935 | 0.0056398 | Not queried | 5.28 | 4.11e-02 | 0.2180389943 |
|  | 13 | 76334583 | 76334866 | PDFSTATSENSEMBLUCSC | 284 | 4 | 11182 | gene | 0.351713 | 0.0104228 | Not queried | 5.26 | 5.71e-02 | 0.2315877671 |
|  | 3 | 55518091 | 55518142 | PDFSTATSENSEMBLUCSC | 52 | 4 | 27839 | gene;island | 0.510060 | 0.0194786 | Not queried | 5.25 | 2.86e-02 | 0.1830867741 |
|  | 3 | 155394109 | 155394428 | PDFSTATSENSEMBLUCSC | 320 | 5 | 28004 | gene;tss | 0.019622 | 0.4311865 | Not queried | 5.24 | 9.52e-02 | 0.3104222821 |
|  | 20 | 21378072 | 21378278 | PDFSTATSENSEMBLUCSC | 207 | 7 | 25629 | tss | 0.478938 | 0.0250791 | Not queried | 5.24 | 1.75e-02 | 0.1830867741 |
|  | 2 | 223169352 | 223169609 | PDFSTATSENSEMBLUCSC | 258 | 4 | 23052 | gene;island | 0.679054 | 0.0154962 | Not queried | 5.24 | 1.14e-01 | 0.3123457953 |
|  | 4 | 154125208 | 154125594 | PDFSTATSENSEMBLUCSC | 387 | 6 | 30640 | tss | 0.233898 | 0.9133312 | Not queried | 5.24 | 2.60e-02 | 0.1830867741 |
|  | 10 | 49812686 | 49813128 | PDFSTATSENSEMBLUCSC | 443 | 4 | 4580 | gene | 0.056221 | 0.0168780 | Not queried | 5.24 | 5.71e-02 | 0.2315877671 |
|  | 18 | 70211774 | 70211974 | PDFSTATSENSEMBLUCSC | 201 | 6 | 19318 | tss;island | 0.448068 | 0.0199522 | Not queried | 5.22 | 1.80e-01 | 0.3826488233 |
|  | 5 | 140578896 | 140579644 | PDFSTATSENSEMBLUCSC | 749 | 9 | 33223 | island | 0.061806 | 0.5573654 | Not queried | 5.22 | 6.25e-02 | 0.2477578846 |
|  | 20 | 36148133 | 36149455 | PDFSTATSENSEMBLUCSC | 1323 | 41 | 25416 | gene;tss;island | 0.977346 | 0.5178004 | NNAT; BLCAP (margin: NNAT; PPIAP3; RP11-425M5.5; BLCAP) | 5.21 | 1.28e-11 | 0.0000000529 |
|  | 5 | 54281437 | 54281733 | PDFSTATSENSEMBLUCSC | 297 | 4 | 32062 | tss | 0.021359 | 0.2787144 | Not queried | 5.21 | 2.86e-02 | 0.1830867741 |
|  | 7 | 27265004 | 27265522 | PDFSTATSENSEMBLUCSC | 519 | 5 | 38364 | island | 0.074195 | 0.2193581 | Not queried | 5.21 | 1.51e-01 | 0.3618000441 |
|  | 1 | 110230252 | 110230633 | PDFSTATSENSEMBLUCSC | 382 | 5 | 3570 | island | 0.012832 | 0.2712789 | Not queried | 5.20 | 7.94e-03 | 0.1589602024 |
|  | 5 | 140613740 | 140613884 | PDFSTATSENSEMBLUCSC | 145 | 5 | 32368 | tss | 0.020419 | 0.3064247 | Not queried | 5.20 | 9.52e-02 | 0.3104222821 |
|  | 2 | 134326055 | 134326366 | PDFSTATSENSEMBLUCSC | 312 | 5 | 23641 | tss | 0.035473 | 0.7882106 | Not queried | 5.20 | 5.56e-02 | 0.2315877671 |
|  | 14 | 100625027 | 100625253 | PDFSTATSENSEMBLUCSC | 227 | 4 | 12206 | gene;island | 0.132817 | 0.7360527 | Not queried | 5.20 | 1.14e-01 | 0.3123457953 |
|  | 17 | 73083632 | 73083887 | PDFSTATSENSEMBLUCSC | 256 | 6 | 17642 | tss;island | 0.014612 | 0.3163723 | Not queried | 5.19 | 4.33e-03 | 0.1459191215 |
|  | 22 | 37099037 | 37099259 | PDFSTATSENSEMBLUCSC | 223 | 4 | 27111 | tss;island | 0.001962 | 0.1277975 | Not queried | 5.19 | 2.86e-02 | 0.1830867741 |
|  | 13 | 108866993 | 108867154 | PDFSTATSENSEMBLUCSC | 162 | 4 | 11221 | gene;island | 0.892977 | 0.4940247 | Not queried | 5.19 | 3.43e-01 | 0.4672865596 |
|  | 3 | 45077649 | 45078075 | PDFSTATSENSEMBLUCSC | 427 | 6 | 29046 | island | 0.006921 | 0.7977079 | Not queried | 5.18 | 4.11e-02 | 0.2180389943 |
|  | 10 | 94820892 | 94820988 | PDFSTATSENSEMBLUCSC | 97 | 6 | 5233 | tss | 0.625951 | 0.9798912 | Not queried | 5.18 | 2.16e-03 | 0.0934794372 |
|  | 10 | 94451351 | 94451736 | PDFSTATSENSEMBLUCSC | 386 | 4 | 4676 | gene;island | 0.025563 | 0.4395985 | Not queried | 5.18 | 2.86e-02 | 0.1830867741 |
|  | 1 | 151812421 | 151812710 | PDFSTATSENSEMBLUCSC | 290 | 4 | 756 | gene;island | 0.036210 | 0.3632121 | Not queried | 5.18 | 5.71e-02 | 0.2315877671 |
|  | 17 | 2907895 | 2908369 | PDFSTATSENSEMBLUCSC | 475 | 5 | 16334 | gene | 0.030995 | 0.4715480 | Not queried | 5.16 | 7.94e-03 | 0.1589602024 |
|  | 6 | 30173936 | 30174261 | PDFSTATSENSEMBLUCSC | 326 | 7 | 33750 | gene | 0.038350 | 0.5739863 | Not queried | 5.16 | 9.73e-02 | 0.3123457953 |
|  | 2 | 119605210 | 119605441 | PDFSTATSENSEMBLUCSC | 232 | 4 | 22842 | gene;island | 0.384170 | 0.0174242 | Not queried | 5.15 | 5.71e-02 | 0.2315877671 |
|  | 11 | 843897 | 844400 | PDFSTATSENSEMBLUCSC | 504 | 8 | 7033 | tss | 0.037768 | 0.5299714 | Not queried | 5.15 | 1.95e-01 | 0.3826488233 |
|  | 11 | 110583882 | 110584082 | PDFSTATSENSEMBLUCSC | 201 | 4 | 7680 | tss;island | 0.116571 | 0.0080829 | Not queried | 5.13 | 1.14e-01 | 0.3123457953 |
|  | 1 | 151689979 | 151690075 | PDFSTATSENSEMBLUCSC | 97 | 4 | 1930 | tss | 0.070568 | 0.6105439 | Not queried | 5.13 | 1.14e-01 | 0.3123457953 |
|  | 3 | 350503 | 351003 | PDFSTATSENSEMBLUCSC | 501 | 6 | 27637 | gene;island | 0.974273 | 0.5238795 | Not queried | 5.13 | 2.16e-03 | 0.0934794372 |
|  | 17 | 40575289 | 40575582 | PDFSTATSENSEMBLUCSC | 294 | 7 | 18337 | island | 0.012102 | 0.2966170 | Not queried | 5.12 | 2.33e-03 | 0.0974014615 |
|  | 11 | 49579997 | 49580074 | PDFSTATSENSEMBLUCSC | 78 | 4 | 7295 | tss | 0.116313 | 0.6179595 | Not queried | 5.11 | 1.14e-01 | 0.3123457953 |
|  | 12 | 2944286 | 2944493 | PDFSTATSENSEMBLUCSC | 208 | 6 | 9491 | tss | 0.399595 | 0.9458567 | Not queried | 5.11 | 4.11e-02 | 0.2180389943 |
|  | 11 | 45868398 | 45868501 | PDFSTATSENSEMBLUCSC | 104 | 4 | 7268 | tss;island | 0.036109 | 0.6300213 | Not queried | 5.11 | 2.86e-02 | 0.1830867741 |
|  | 12 | 119772354 | 119772421 | PDFSTATSENSEMBLUCSC | 68 | 4 | 10050 | tss | 0.011164 | 0.3469812 | Not queried | 5.11 | 3.43e-01 | 0.4672865596 |
|  | 3 | 98376390 | 98376574 | PDFSTATSENSEMBLUCSC | 185 | 4 | 29316 | island | 0.138696 | 0.0068149 | Not queried | 5.10 | 2.86e-02 | 0.1830867741 |
|  | 1 | 154539974 | 154540159 | PDFSTATSENSEMBLUCSC | 186 | 4 | 1967 | tss | 0.040628 | 0.5109139 | Not queried | 5.09 | 3.43e-01 | 0.4672865596 |
|  | 3 | 197401858 | 197402207 | PDFSTATSENSEMBLUCSC | 350 | 5 | 28105 | gene;tss | 0.985084 | 0.5200625 | Not queried | 5.08 | 5.56e-02 | 0.2315877671 |
|  | 2 | 66809780 | 66809985 | PDFSTATSENSEMBLUCSC | 206 | 4 | 24358 | island | 0.005937 | 0.2516030 | Not queried | 5.07 | 3.43e-01 | 0.4672865596 |
|  | 4 | 91156040 | 91156290 | PDFSTATSENSEMBLUCSC | 251 | 4 | 30060 | gene | 0.210539 | 0.8317414 | Not queried | 5.07 | 5.71e-02 | 0.2315877671 |
|  | 1 | 236228598 | 236228837 | PDFSTATSENSEMBLUCSC | 240 | 6 | 2339 | tss;island | 0.494590 | 0.0239711 | Not queried | 5.07 | 2.16e-03 | 0.0934794372 |
|  | 8 | 37605359 | 37605978 | PDFSTATSENSEMBLUCSC | 620 | 7 | 39279 | gene | 0.593654 | 0.0299448 | Not queried | 5.07 | 2.62e-02 | 0.1830867741 |
|  | 3 | 8809306 | 8809715 | PDFSTATSENSEMBLUCSC | 410 | 4 | 27646 | gene;island | 0.011997 | 0.3695701 | Not queried | 5.06 | 2.86e-02 | 0.1830867741 |
|  | 1 | 9788517 | 9788769 | PDFSTATSENSEMBLUCSC | 253 | 4 | 205 | gene;island | 0.972305 | 0.5121900 | Not queried | 5.06 | 2.86e-02 | 0.1830867741 |
|  | 10 | 94820892 | 94821085 | PDFSTATSENSEMBLUCSC | 194 | 7 | 5949 | island | 0.637901 | 0.9739686 | Not queried | 5.06 | 2.62e-02 | 0.1830867741 |
|  | 12 | 43946284 | 43946518 | PDFSTATSENSEMBLUCSC | 235 | 4 | 9647 | tss;island | 0.048522 | 0.4932203 | Not queried | 5.04 | 2.86e-02 | 0.1830867741 |
|  | 8 | 95962352 | 95962463 | PDFSTATSENSEMBLUCSC | 112 | 5 | 39821 | tss;island | 0.009554 | 0.1993835 | Not queried | 5.04 | 9.52e-02 | 0.3104222821 |
|  | 19 | 22605151 | 22605234 | PDFSTATSENSEMBLUCSC | 84 | 4 | 20663 | tss | 0.029923 | 0.4247256 | Not queried | 5.04 | 2.86e-02 | 0.1830867741 |
|  | 7 | 27183133 | 27183274 | PDFSTATSENSEMBLUCSC | 142 | 4 | 36934 | gene | 0.512322 | 0.9721518 | Not queried | 5.04 | 2.86e-02 | 0.1830867741 |
|  | 1 | 111743020 | 111743271 | PDFSTATSENSEMBLUCSC | 252 | 4 | 682 | gene | 0.551104 | 0.9709660 | Not queried | 5.04 | 2.86e-02 | 0.1830867741 |
|  | 11 | 61522741 | 61522807 | PDFSTATSENSEMBLUCSC | 67 | 5 | 6632 | gene;tss;island | 0.559439 | 0.9098985 | Not queried | 5.03 | 7.94e-03 | 0.1589602024 |
|  | 6 | 33141678 | 33142006 | PDFSTATSENSEMBLUCSC | 329 | 7 | 34202 | gene | 0.021523 | 0.3298105 | Not queried | 5.03 | 2.62e-02 | 0.1830867741 |
|  | 11 | 31848347 | 31848828 | PDFSTATSENSEMBLUCSC | 482 | 5 | 8170 | island | 0.142804 | 0.0250653 | Not queried | 5.03 | 1.51e-01 | 0.3618000441 |
|  | 5 | 140561893 | 140562236 | PDFSTATSENSEMBLUCSC | 344 | 6 | 31812 | gene;island | 0.014005 | 0.3831282 | Not queried | 5.03 | 1.52e-02 | 0.1830867741 |
|  | 12 | 89748726 | 89749142 | PDFSTATSENSEMBLUCSC | 417 | 9 | 10666 | island | 0.028909 | 0.4848829 | Not queried | 5.02 | 3.99e-03 | 0.1459191215 |
|  | 11 | 705692 | 706100 | PDFSTATSENSEMBLUCSC | 409 | 7 | 7024 | tss;island | 0.046495 | 0.6118280 | Not queried | 5.02 | 1.17e-03 | 0.0833132385 |
|  | 12 | 103359273 | 103359572 | PDFSTATSENSEMBLUCSC | 300 | 4 | 10730 | island | 0.047543 | 0.4558243 | Not queried | 5.01 | 2.86e-02 | 0.1830867741 |
|  | 7 | 48019388 | 48019713 | PDFSTATSENSEMBLUCSC | 326 | 4 | 37590 | tss | 0.162706 | 0.8216916 | Not queried | 5.01 | 2.00e-01 | 0.3826488233 |
|  | 13 | 100310162 | 100310405 | PDFSTATSENSEMBLUCSC | 244 | 4 | 11205 | gene;island | 0.059154 | 0.8741912 | Not queried | 5.00 | 5.71e-02 | 0.2315877671 |
|  | 3 | 48694451 | 48694673 | PDFSTATSENSEMBLUCSC | 223 | 4 | 27768 | gene;island | 0.532859 | 0.9696640 | Not queried | 5.00 | 2.86e-02 | 0.1830867741 |
|  | 15 | 90455635 | 90455799 | PDFSTATSENSEMBLUCSC | 165 | 4 | 13475 | gene;island | 0.015269 | 0.0198965 | Not queried | 5.00 | 6.86e-01 | 0.7223604052 |
|  | 6 | 31148332 | 31148748 | PDFSTATSENSEMBLUCSC | 417 | 16 | 35754 | island | 0.535283 | 0.9640636 | POU5F1 (margin: POU5F1; PSORS1C3) | 4.98 | 3.33e-09 | 0.0000034488 |
|  | 12 | 133016214 | 133016787 | PDFSTATSENSEMBLUCSC | 574 | 6 | 11055 | island | 0.054808 | 0.9120153 | Not queried | 4.98 | 9.31e-02 | 0.3104222821 |
|  | 2 | 74780172 | 74780617 | PDFSTATSENSEMBLUCSC | 446 | 6 | 22750 | gene;island | 0.032229 | 0.5529991 | Not queried | 4.98 | 4.11e-02 | 0.2180389943 |
|  | 1 | 3663164 | 3663832 | PDFSTATSENSEMBLUCSC | 669 | 6 | 144 | gene | 0.295363 | 0.0097385 | Not queried | 4.97 | 8.66e-03 | 0.1595382395 |
|  | 8 | 120219902 | 120220131 | PDFSTATSENSEMBLUCSC | 230 | 7 | 39885 | tss;island | 0.923056 | 0.3317258 | Not queried | 4.95 | 4.08e-03 | 0.1459191215 |
|  | 3 | 48413550 | 48413707 | PDFSTATSENSEMBLUCSC | 158 | 4 | 28296 | tss | 0.038226 | 0.5362540 | Not queried | 4.95 | 2.86e-02 | 0.1830867741 |
|  | 12 | 6420020 | 6420052 | PDFSTATSENSEMBLUCSC | 33 | 6 | 9023 | gene;tss;island | 0.283022 | 0.0171517 | Not queried | 4.94 | 2.16e-03 | 0.0934794372 |
|  | 19 | 7735239 | 7735510 | PDFSTATSENSEMBLUCSC | 272 | 4 | 21461 | island | 0.007351 | 0.1123205 | Not queried | 4.94 | 1.14e-01 | 0.3123457953 |
|  | 19 | 53757910 | 53758309 | PDFSTATSENSEMBLUCSC | 400 | 7 | 22371 | island | 0.255299 | 0.0133766 | Not queried | 4.94 | 1.17e-03 | 0.0833132385 |
|  | 11 | 116700436 | 116700556 | PDFSTATSENSEMBLUCSC | 121 | 4 | 7713 | tss | 0.032137 | 0.4295356 | Not queried | 4.93 | 2.86e-02 | 0.1830867741 |
|  | 12 | 119772354 | 119772577 | PDFSTATSENSEMBLUCSC | 224 | 5 | 10874 | island | 0.017043 | 0.5616242 | Not queried | 4.92 | 3.10e-01 | 0.4672865596 |
|  | 22 | 30476089 | 30476345 | PDFSTATSENSEMBLUCSC | 257 | 9 | 27055 | tss | 0.115289 | 0.9223177 | Not queried | 4.92 | 3.99e-03 | 0.1459191215 |
|  | 3 | 183543564 | 183544049 | PDFSTATSENSEMBLUCSC | 486 | 6 | 28748 | tss;island | 0.685619 | 0.0611229 | Not queried | 4.91 | 8.66e-03 | 0.1595382395 |
|  | 2 | 74729100 | 74729710 | PDFSTATSENSEMBLUCSC | 611 | 7 | 22744 | gene;tss;island | 0.037372 | 0.5756058 | Not queried | 4.91 | 5.30e-02 | 0.2315877671 |
|  | 1 | 241520598 | 241520941 | PDFSTATSENSEMBLUCSC | 344 | 6 | 2349 | tss;island | 0.015350 | 0.4575193 | Not queried | 4.91 | 2.60e-02 | 0.1830867741 |
|  | 17 | 7555132 | 7555411 | PDFSTATSENSEMBLUCSC | 280 | 4 | 16408 | gene;island | 0.009619 | 0.2344496 | Not queried | 4.91 | 2.86e-02 | 0.1830867741 |
|  | 15 | 37179603 | 37179779 | PDFSTATSENSEMBLUCSC | 177 | 4 | 13569 | tss;island | 0.021763 | 0.2806584 | Not queried | 4.91 | 2.86e-02 | 0.1830867741 |
|  | 7 | 142583555 | 142583836 | PDFSTATSENSEMBLUCSC | 282 | 4 | 37948 | tss | 0.012480 | 0.2072602 | Not queried | 4.90 | 2.00e-01 | 0.3826488233 |
|  | 4 | 111540884 | 111541339 | PDFSTATSENSEMBLUCSC | 456 | 4 | 30094 | gene;island | 0.013676 | 0.2969051 | Not queried | 4.90 | 2.86e-02 | 0.1830867741 |
|  | 11 | 101454676 | 101454996 | PDFSTATSENSEMBLUCSC | 321 | 7 | 7643 | tss | 0.056417 | 0.4534735 | Not queried | 4.90 | 1.28e-01 | 0.3397986247 |
|  | 16 | 69141250 | 69141478 | PDFSTATSENSEMBLUCSC | 229 | 7 | 14817 | gene;island | 0.008911 | 0.2556180 | Not queried | 4.88 | 1.75e-02 | 0.1830867741 |
|  | 20 | 2188420 | 2188663 | PDFSTATSENSEMBLUCSC | 244 | 4 | 25942 | island | 0.035850 | 0.4261043 | Not queried | 4.88 | 5.71e-02 | 0.2315877671 |
|  | 8 | 144654887 | 144655030 | PDFSTATSENSEMBLUCSC | 144 | 4 | 40672 | island | 0.160018 | 0.8568316 | Not queried | 4.88 | 3.43e-01 | 0.4672865596 |
|  | 6 | 152126736 | 152126938 | PDFSTATSENSEMBLUCSC | 203 | 5 | 34512 | gene;island | 0.270620 | 0.5778432 | Not queried | 4.87 | 1.51e-01 | 0.3618000441 |
|  | 19 | 46001550 | 46001746 | PDFSTATSENSEMBLUCSC | 197 | 4 | 20873 | tss;island | 0.021071 | 0.5629363 | Not queried | 4.86 | 2.86e-02 | 0.1830867741 |
|  | 21 | 36259067 | 36259460 | PDFSTATSENSEMBLUCSC | 394 | 4 | 26412 | gene;island | 0.571410 | 0.0489187 | Not queried | 4.86 | 2.86e-02 | 0.1830867741 |
|  | 1 | 27961563 | 27962037 | PDFSTATSENSEMBLUCSC | 475 | 7 | 2925 | island | 0.035234 | 0.5355589 | Not queried | 4.86 | 1.11e-02 | 0.1821650572 |
|  | 7 | 1882997 | 1883420 | PDFSTATSENSEMBLUCSC | 424 | 6 | 36767 | gene;island | 0.444649 | 0.9684212 | Not queried | 4.85 | 1.52e-02 | 0.1830867741 |
|  | 18 | 23713838 | 23714084 | PDFSTATSENSEMBLUCSC | 247 | 4 | 19079 | gene | 0.958339 | 0.4553496 | Not queried | 4.85 | 2.86e-02 | 0.1830867741 |
|  | 14 | 53418879 | 53419111 | PDFSTATSENSEMBLUCSC | 233 | 5 | 12402 | tss;island | 0.017951 | 0.5144407 | Not queried | 4.85 | 4.21e-01 | 0.5404252807 |
|  | 10 | 38691787 | 38691801 | PDFSTATSENSEMBLUCSC | 15 | 4 | 5037 | tss;island | 0.386527 | 0.0266214 | Not queried | 4.85 | 2.86e-02 | 0.1830867741 |
|  | 17 | 19883326 | 19883716 | PDFSTATSENSEMBLUCSC | 391 | 4 | 18108 | island | 0.498896 | 0.9530463 | Not queried | 4.84 | 5.71e-02 | 0.2315877671 |
|  | 4 | 83956648 | 83957095 | PDFSTATSENSEMBLUCSC | 448 | 5 | 30042 | gene | 0.192508 | 0.0029961 | Not queried | 4.83 | 5.56e-02 | 0.2315877671 |
|  | 17 | 46622454 | 46622899 | PDFSTATSENSEMBLUCSC | 446 | 8 | 17457 | tss;island | 0.635788 | 0.9574357 | Not queried | 4.83 | 6.50e-02 | 0.2477578846 |
|  | 16 | 3225355 | 3225686 | PDFSTATSENSEMBLUCSC | 332 | 5 | 15639 | island | 0.010129 | 0.2240548 | Not queried | 4.82 | 9.52e-02 | 0.3104222821 |
|  | 6 | 166876490 | 166877038 | PDFSTATSENSEMBLUCSC | 549 | 8 | 34545 | gene | 0.010924 | 0.2902401 | Not queried | 4.82 | 6.50e-02 | 0.2477578846 |
|  | 14 | 105830509 | 105830859 | PDFSTATSENSEMBLUCSC | 351 | 5 | 12262 | gene;island | 0.545313 | 0.0343959 | Not queried | 4.82 | 5.56e-02 | 0.2315877671 |
|  | 10 | 133793442 | 133793734 | PDFSTATSENSEMBLUCSC | 293 | 5 | 4847 | gene;island | 0.974366 | 0.5660057 | Not queried | 4.82 | 7.94e-03 | 0.1589602024 |
|  | 17 | 17109570 | 17110353 | PDFSTATSENSEMBLUCSC | 784 | 14 | 18060 | island | 0.016359 | 0.4901897 | Not queried | 4.82 | 2.10e-01 | 0.3935758299 |
|  | 7 | 101768610 | 101768874 | PDFSTATSENSEMBLUCSC | 265 | 4 | 37161 | gene | 0.170622 | 0.7654548 | Not queried | 4.82 | 5.71e-02 | 0.2315877671 |
|  | 6 | 34499314 | 34499504 | PDFSTATSENSEMBLUCSC | 191 | 4 | 34304 | gene;island | 0.891195 | 0.4481749 | Not queried | 4.81 | 2.86e-02 | 0.1830867741 |
|  | 19 | 53758233 | 53758309 | PDFSTATSENSEMBLUCSC | 77 | 4 | 21026 | tss | 0.235898 | 0.0119834 | Not queried | 4.81 | 2.86e-02 | 0.1830867741 |
|  | 13 | 112720171 | 112720565 | PDFSTATSENSEMBLUCSC | 395 | 6 | 11909 | island | 0.046706 | 0.5045828 | Not queried | 4.79 | 6.49e-02 | 0.2477578846 |
|  | 4 | 111551642 | 111552040 | PDFSTATSENSEMBLUCSC | 399 | 4 | 30097 | gene;island | 0.027254 | 0.3072172 | Not queried | 4.78 | 2.86e-02 | 0.1830867741 |
|  | 16 | 67428155 | 67428497 | PDFSTATSENSEMBLUCSC | 343 | 4 | 15301 | tss;island | 0.037789 | 0.3791329 | Not queried | 4.78 | 5.71e-02 | 0.2315877671 |
|  | 11 | 66444632 | 66444806 | PDFSTATSENSEMBLUCSC | 175 | 4 | 6735 | gene;island | 0.031232 | 0.0039076 | Not queried | 4.77 | 3.43e-01 | 0.4672865596 |
|  | 4 | 4866047 | 4866192 | PDFSTATSENSEMBLUCSC | 146 | 4 | 30871 | island | 0.815303 | 0.0199716 | Not queried | 4.77 | 3.43e-01 | 0.4672865596 |
|  | 14 | 51339072 | 51339216 | PDFSTATSENSEMBLUCSC | 145 | 4 | 12078 | gene;island | 0.045052 | 0.5146187 | Not queried | 4.76 | 2.00e-01 | 0.3826488233 |
|  | 4 | 154125208 | 154125720 | PDFSTATSENSEMBLUCSC | 513 | 9 | 30152 | gene | 0.649592 | 0.9464361 | Not queried | 4.76 | 1.88e-02 | 0.1830867741 |
|  | 21 | 27372387 | 27372461 | PDFSTATSENSEMBLUCSC | 75 | 4 | 26392 | gene | 0.033666 | 0.4811059 | Not queried | 4.76 | 2.86e-02 | 0.1830867741 |
|  | 20 | 23549455 | 23549525 | PDFSTATSENSEMBLUCSC | 71 | 5 | 25641 | tss | 0.033461 | 0.5019344 | Not queried | 4.76 | 7.94e-03 | 0.1589602024 |
|  | 2 | 208890341 | 208890496 | PDFSTATSENSEMBLUCSC | 156 | 4 | 23845 | tss | 0.001161 | 0.0147307 | Not queried | 4.76 | 1.14e-01 | 0.3123457953 |
|  | 8 | 105342214 | 105342491 | PDFSTATSENSEMBLUCSC | 278 | 4 | 40522 | island | 0.016720 | 0.3133816 | Not queried | 4.75 | 2.86e-02 | 0.1830867741 |
|  | 3 | 112013079 | 112013357 | PDFSTATSENSEMBLUCSC | 279 | 4 | 28501 | tss | 0.664826 | 0.9812223 | Not queried | 4.75 | 2.86e-02 | 0.1830867741 |
|  | 10 | 125425583 | 125425692 | PDFSTATSENSEMBLUCSC | 110 | 5 | 5419 | tss;island | 0.019683 | 0.6236136 | Not queried | 4.75 | 1.59e-02 | 0.1830867741 |
|  | 10 | 13748985 | 13749625 | PDFSTATSENSEMBLUCSC | 641 | 9 | 4504 | gene | 0.176326 | 0.0100498 | Not queried | 4.73 | 5.64e-03 | 0.1589602024 |
|  | 17 | 4486934 | 4487125 | PDFSTATSENSEMBLUCSC | 192 | 5 | 17026 | tss;island | 0.558222 | 0.9471588 | Not queried | 4.73 | 5.56e-02 | 0.2315877671 |
|  | 22 | 45073031 | 45073165 | PDFSTATSENSEMBLUCSC | 135 | 5 | 26893 | gene | 0.008589 | 0.2915127 | Not queried | 4.72 | 2.22e-01 | 0.3935758299 |
|  | 6 | 50817157 | 50817450 | PDFSTATSENSEMBLUCSC | 294 | 5 | 36199 | island | 0.017360 | 0.2985601 | Not queried | 4.72 | 5.56e-02 | 0.2315877671 |
|  | 11 | 73358518 | 73358657 | PDFSTATSENSEMBLUCSC | 140 | 4 | 6822 | gene;tss | 0.351410 | 0.0142168 | Not queried | 4.71 | 2.86e-02 | 0.1830867741 |
|  | 1 | 110210265 | 110210641 | PDFSTATSENSEMBLUCSC | 377 | 4 | 1809 | tss | 0.036149 | 0.5720959 | Not queried | 4.70 | 2.00e-01 | 0.3826488233 |
|  | 2 | 215275757 | 215276325 | PDFSTATSENSEMBLUCSC | 569 | 6 | 23860 | tss | 0.004457 | 0.1140217 | Not queried | 4.69 | 8.66e-03 | 0.1595382395 |
|  | 1 | 99729757 | 99729807 | PDFSTATSENSEMBLUCSC | 51 | 6 | 1773 | tss;island | 0.017749 | 0.2304625 | Not queried | 4.69 | 8.66e-03 | 0.1595382395 |
|  | 6 | 28226885 | 28227093 | PDFSTATSENSEMBLUCSC | 209 | 7 | 34738 | tss | 0.457823 | 0.0277058 | Not queried | 4.69 | 5.83e-04 | 0.0548331102 |
|  | 12 | 23998921 | 23999045 | PDFSTATSENSEMBLUCSC | 125 | 4 | 9082 | gene | 0.467753 | 0.9454656 | Not queried | 4.69 | 2.86e-02 | 0.1830867741 |
|  | 4 | 57773149 | 57773436 | PDFSTATSENSEMBLUCSC | 288 | 5 | 30412 | tss;island | 0.043664 | 0.5136327 | Not queried | 4.68 | 3.17e-02 | 0.1866936846 |
|  | 1 | 20669724 | 20669975 | PDFSTATSENSEMBLUCSC | 252 | 4 | 287 | gene;island | 0.044276 | 0.4179377 | Not queried | 4.67 | 2.86e-02 | 0.1830867741 |
|  | 7 | 5111488 | 5111669 | PDFSTATSENSEMBLUCSC | 182 | 8 | 37420 | tss;island | 0.474622 | 0.0182271 | Not queried | 4.65 | 6.22e-04 | 0.0548331102 |
|  | 2 | 50201382 | 50201511 | PDFSTATSENSEMBLUCSC | 130 | 5 | 22675 | gene | 0.016937 | 0.5079488 | Not queried | 4.65 | 1.59e-02 | 0.1830867741 |
|  | 18 | 32398024 | 32398287 | PDFSTATSENSEMBLUCSC | 264 | 4 | 19086 | gene;tss | 0.215588 | 0.4662638 | Not queried | 4.65 | 6.86e-01 | 0.7223604052 |
|  | 1 | 32410413 | 32410678 | PDFSTATSENSEMBLUCSC | 266 | 4 | 2979 | island | 0.029360 | 0.3300163 | Not queried | 4.65 | 2.86e-02 | 0.1830867741 |
|  | 6 | 76203530 | 76203675 | PDFSTATSENSEMBLUCSC | 146 | 6 | 35077 | tss | 0.439292 | 0.8551064 | Not queried | 4.64 | 2.16e-03 | 0.0934794372 |
|  | 8 | 105479318 | 105479420 | PDFSTATSENSEMBLUCSC | 103 | 4 | 39861 | tss | 0.023804 | 0.4697303 | Not queried | 4.64 | 5.71e-02 | 0.2315877671 |
|  | 20 | 13976093 | 13976143 | PDFSTATSENSEMBLUCSC | 51 | 6 | 25605 | tss | 0.965744 | 0.3174987 | Not queried | 4.64 | 2.60e-02 | 0.1830867741 |
|  | 7 | 158750244 | 158750607 | PDFSTATSENSEMBLUCSC | 364 | 4 | 39157 | island | 0.083961 | 0.7157174 | Not queried | 4.63 | 5.71e-02 | 0.2315877671 |
|  | 6 | 29596840 | 29597113 | PDFSTATSENSEMBLUCSC | 274 | 5 | 33679 | gene;tss;island | 0.003409 | 0.0320932 | Not queried | 4.62 | 9.52e-02 | 0.3104222821 |
|  | 17 | 46831306 | 46831742 | PDFSTATSENSEMBLUCSC | 437 | 4 | 18508 | island | 0.330166 | 0.0144688 | Not queried | 4.62 | 1.14e-01 | 0.3123457953 |
|  | 20 | 13976093 | 13976190 | PDFSTATSENSEMBLUCSC | 98 | 7 | 26012 | island | 0.964378 | 0.1845648 | Not queried | 4.62 | 7.28e-02 | 0.2713481487 |
|  | 2 | 20865651 | 20865847 | PDFSTATSENSEMBLUCSC | 197 | 4 | 23214 | tss;island | 0.010929 | 0.1713343 | Not queried | 4.62 | 2.86e-02 | 0.1830867741 |
|  | 19 | 19648890 | 19649054 | PDFSTATSENSEMBLUCSC | 165 | 7 | 20645 | tss | 0.356053 | 0.0417914 | Not queried | 4.60 | 7.28e-02 | 0.2713481487 |
|  | 10 | 56561096 | 56561213 | PDFSTATSENSEMBLUCSC | 118 | 4 | 5087 | tss | 0.613328 | 0.2016158 | Not queried | 4.60 | 5.71e-02 | 0.2315877671 |
|  | 10 | 89263900 | 89264128 | PDFSTATSENSEMBLUCSC | 229 | 6 | 5202 | tss;island | 0.476123 | 0.9812681 | Not queried | 4.60 | 6.49e-02 | 0.2477578846 |
|  | 7 | 31232532 | 31232861 | PDFSTATSENSEMBLUCSC | 330 | 6 | 38391 | island | 0.027387 | 0.2222159 | Not queried | 4.59 | 6.49e-02 | 0.2477578846 |
|  | 10 | 97200911 | 97201172 | PDFSTATSENSEMBLUCSC | 262 | 4 | 4688 | gene | 0.354050 | 0.8986871 | Not queried | 4.59 | 1.14e-01 | 0.3123457953 |
|  | 1 | 85527430 | 85527943 | PDFSTATSENSEMBLUCSC | 514 | 7 | 1721 | tss | 0.068178 | 0.0063141 | Not queried | 4.59 | 2.59e-01 | 0.4381244265 |
|  | 10 | 75531971 | 75531995 | PDFSTATSENSEMBLUCSC | 25 | 5 | 5163 | tss | 0.132219 | 0.0050752 | Not queried | 4.59 | 3.17e-02 | 0.1866936846 |
|  | 2 | 213400697 | 213401333 | PDFSTATSENSEMBLUCSC | 637 | 5 | 23005 | gene;island | 0.023302 | 0.0148530 | Not queried | 4.58 | 5.48e-01 | 0.6179718485 |
|  | 11 | 113746456 | 113746831 | PDFSTATSENSEMBLUCSC | 376 | 5 | 7706 | tss;island | 0.110392 | 0.0053712 | Not queried | 4.57 | 1.51e-01 | 0.3618000441 |
|  | 14 | 101036192 | 101036470 | PDFSTATSENSEMBLUCSC | 279 | 4 | 12605 | tss;island | 0.420754 | 0.0213335 | Not queried | 4.57 | 1.14e-01 | 0.3123457953 |
|  | 1 | 156696747 | 156697041 | PDFSTATSENSEMBLUCSC | 295 | 4 | 828 | gene;island | 0.034085 | 0.4764645 | Not queried | 4.57 | 2.86e-02 | 0.1830867741 |
|  | 19 | 52391234 | 52391789 | PDFSTATSENSEMBLUCSC | 556 | 10 | 20999 | tss | 0.975093 | 0.6137836 | CTC-429C10.2; ZNF577; ZNF649 (margin: CTC-429C10.2; ZNF577; ZNF649) | 4.56 | 3.25e-04 | 0.0498675731 |
|  | 17 | 78800574 | 78800806 | PDFSTATSENSEMBLUCSC | 233 | 5 | 16875 | gene;island | 0.645066 | 0.1962501 | Not queried | 4.56 | 7.94e-03 | 0.1589602024 |
|  | 5 | 140473857 | 140474184 | PDFSTATSENSEMBLUCSC | 328 | 6 | 32361 | tss | 0.028922 | 0.4164774 | Not queried | 4.56 | 2.60e-02 | 0.1830867741 |
|  | 17 | 7341436 | 7341641 | PDFSTATSENSEMBLUCSC | 206 | 4 | 17083 | tss;island | 0.133342 | 0.8110419 | Not queried | 4.56 | 5.71e-02 | 0.2315877671 |
|  | 6 | 24358304 | 24358327 | PDFSTATSENSEMBLUCSC | 24 | 4 | 34667 | tss | 0.022145 | 0.3741606 | Not queried | 4.55 | 2.86e-02 | 0.1830867741 |
|  | 21 | 34398085 | 34398265 | PDFSTATSENSEMBLUCSC | 181 | 8 | 26642 | island | 0.012381 | 0.1706910 | Not queried | 4.55 | 6.22e-04 | 0.0548331102 |
|  | 3 | 53190593 | 53190763 | PDFSTATSENSEMBLUCSC | 171 | 4 | 29198 | island | 0.355074 | 0.0181910 | Not queried | 4.55 | 1.14e-01 | 0.3123457953 |
|  | 1 | 1266468 | 1266700 | PDFSTATSENSEMBLUCSC | 233 | 5 | 1147 | tss | 0.886919 | 0.2308060 | Not queried | 4.55 | 7.94e-03 | 0.1589602024 |
|  | 1 | 111743020 | 111743537 | PDFSTATSENSEMBLUCSC | 518 | 7 | 3588 | island | 0.493765 | 0.9550531 | Not queried | 4.54 | 5.83e-04 | 0.0548331102 |
|  | 6 | 10414350 | 10414565 | PDFSTATSENSEMBLUCSC | 216 | 4 | 33559 | gene;island | 0.011297 | 0.3656582 | Not queried | 4.54 | 5.71e-02 | 0.2315877671 |
|  | 8 | 25902400 | 25902611 | PDFSTATSENSEMBLUCSC | 212 | 5 | 39608 | tss | 0.540746 | 0.0388134 | Not queried | 4.53 | 5.56e-02 | 0.2315877671 |
|  | 1 | 85514554 | 85514708 | PDFSTATSENSEMBLUCSC | 155 | 4 | 1720 | tss;island | 0.082153 | 0.0046746 | Not queried | 4.53 | 5.71e-02 | 0.2315877671 |
|  | 6 | 31237013 | 31237199 | PDFSTATSENSEMBLUCSC | 187 | 4 | 33864 | gene;island | 0.772152 | 0.2335941 | Not queried | 4.52 | 5.71e-02 | 0.2315877671 |
|  | 3 | 181437145 | 181437712 | PDFSTATSENSEMBLUCSC | 568 | 5 | 28043 | gene;island | 0.003133 | 0.2850011 | Not queried | 4.52 | 1.51e-01 | 0.3618000441 |
|  | 10 | 77164987 | 77165293 | PDFSTATSENSEMBLUCSC | 307 | 4 | 4644 | gene;island | 0.956553 | 0.7227986 | Not queried | 4.52 | 1.14e-01 | 0.3123457953 |
|  | 7 | 143582146 | 143582630 | PDFSTATSENSEMBLUCSC | 485 | 4 | 37256 | gene;island | 0.224821 | 0.0094937 | Not queried | 4.51 | 2.86e-02 | 0.1830867741 |
|  | 20 | 23015899 | 23015936 | PDFSTATSENSEMBLUCSC | 38 | 5 | 25635 | tss;island | 0.512118 | 0.0368236 | Not queried | 4.51 | 1.59e-02 | 0.1830867741 |
|  | 8 | 72757787 | 72758095 | PDFSTATSENSEMBLUCSC | 309 | 4 | 39764 | tss;island | 0.030796 | 0.4146138 | Not queried | 4.51 | 2.86e-02 | 0.1830867741 |
|  | 12 | 49488554 | 49488898 | PDFSTATSENSEMBLUCSC | 345 | 4 | 10385 | island | 0.011572 | 0.1002816 | Not queried | 4.51 | 2.86e-02 | 0.1830867741 |
|  | 12 | 49942757 | 49943340 | PDFSTATSENSEMBLUCSC | 584 | 5 | 9120 | gene;island | 0.066279 | 0.3095010 | Not queried | 4.50 | 5.56e-02 | 0.2315877671 |
|  | 11 | 64058878 | 64059057 | PDFSTATSENSEMBLUCSC | 180 | 4 | 6677 | gene | 0.098692 | 0.2654192 | Not queried | 4.50 | 3.43e-01 | 0.4672865596 |
|  | 1 | 181451906 | 181452137 | PDFSTATSENSEMBLUCSC | 232 | 6 | 2129 | tss;island | 0.010685 | 0.1513533 | Not queried | 4.50 | 2.16e-03 | 0.0934794372 |
|  | 3 | 197807842 | 197808169 | PDFSTATSENSEMBLUCSC | 328 | 5 | 28829 | tss;island | 0.022708 | 0.4503911 | Not queried | 4.49 | 7.94e-03 | 0.1589602024 |
|  | 6 | 136871881 | 136872119 | PDFSTATSENSEMBLUCSC | 239 | 8 | 35242 | tss | 0.167717 | 0.0100143 | Not queried | 4.49 | 6.22e-04 | 0.0548331102 |
|  | 19 | 51141227 | 51141413 | PDFSTATSENSEMBLUCSC | 187 | 4 | 20180 | gene;island | 0.100668 | 0.4914495 | Not queried | 4.49 | 2.86e-02 | 0.1830867741 |
|  | 3 | 164914471 | 164914621 | PDFSTATSENSEMBLUCSC | 151 | 5 | 28699 | tss | 0.053107 | 0.6152631 | Not queried | 4.48 | 7.94e-03 | 0.1589602024 |
|  | 3 | 42947263 | 42947565 | PDFSTATSENSEMBLUCSC | 303 | 5 | 28250 | tss | 0.181297 | 0.5223723 | Not queried | 4.48 | 2.22e-01 | 0.3935758299 |
|  | 11 | 118550443 | 118550644 | PDFSTATSENSEMBLUCSC | 202 | 4 | 7737 | tss | 0.016582 | 0.2279150 | Not queried | 4.48 | 1.14e-01 | 0.3123457953 |
|  | 10 | 3824387 | 3824687 | PDFSTATSENSEMBLUCSC | 301 | 4 | 4476 | gene;island | 0.027255 | 0.5177853 | Not queried | 4.48 | 2.00e-01 | 0.3826488233 |
|  | 14 | 96152464 | 96152706 | PDFSTATSENSEMBLUCSC | 243 | 5 | 12589 | tss | 0.514913 | 0.9577324 | Not queried | 4.48 | 1.51e-01 | 0.3618000441 |
|  | 2 | 217560272 | 217560403 | PDFSTATSENSEMBLUCSC | 132 | 5 | 23871 | tss;island | 0.136569 | 0.0070700 | Not queried | 4.48 | 3.17e-02 | 0.1866936846 |
|  | 6 | 24358236 | 24358327 | PDFSTATSENSEMBLUCSC | 92 | 5 | 33590 | gene;island | 0.019462 | 0.3646849 | Not queried | 4.47 | 5.56e-02 | 0.2315877671 |
|  | 6 | 132945515 | 132945592 | PDFSTATSENSEMBLUCSC | 78 | 4 | 35229 | tss | 0.803828 | 0.9874499 | Not queried | 4.47 | 2.86e-02 | 0.1830867741 |
|  | 2 | 27665017 | 27665306 | PDFSTATSENSEMBLUCSC | 290 | 7 | 24185 | island | 0.669726 | 0.9765713 | Not queried | 4.47 | 5.83e-04 | 0.0548331102 |
|  | 2 | 45397776 | 45398036 | PDFSTATSENSEMBLUCSC | 261 | 4 | 24270 | island | 0.329663 | 0.8258134 | Not queried | 4.46 | 2.86e-02 | 0.1830867741 |
|  | 1 | 9884565 | 9884732 | PDFSTATSENSEMBLUCSC | 168 | 8 | 1230 | tss;island | 0.068205 | 0.4993870 | Not queried | 4.45 | 6.99e-03 | 0.1589602024 |
|  | 12 | 109728043 | 109728241 | PDFSTATSENSEMBLUCSC | 199 | 4 | 9291 | gene;island | 0.201317 | 0.6909341 | Not queried | 4.45 | 5.71e-02 | 0.2315877671 |
|  | 3 | 193587490 | 193587939 | PDFSTATSENSEMBLUCSC | 450 | 4 | 29778 | island | 0.126042 | 0.5526444 | Not queried | 4.45 | 1.14e-01 | 0.3123457953 |
|  | 11 | 129817591 | 129817717 | PDFSTATSENSEMBLUCSC | 127 | 4 | 7814 | tss | 0.497435 | 0.2067574 | Not queried | 4.43 | 2.86e-02 | 0.1830867741 |
|  | 19 | 38794554 | 38794664 | PDFSTATSENSEMBLUCSC | 111 | 4 | 20749 | tss | 0.831982 | 0.1888633 | Not queried | 4.42 | 2.86e-02 | 0.1830867741 |
|  | 6 | 157931791 | 157932180 | PDFSTATSENSEMBLUCSC | 390 | 6 | 34524 | gene | 0.494312 | 0.9437816 | Not queried | 4.42 | 2.16e-03 | 0.0934794372 |
|  | 20 | 5891730 | 5891968 | PDFSTATSENSEMBLUCSC | 239 | 5 | 25586 | tss | 0.171598 | 0.0099436 | Not queried | 4.41 | 5.56e-02 | 0.2315877671 |
|  | 1 | 1266468 | 1266781 | PDFSTATSENSEMBLUCSC | 314 | 6 | 2426 | island | 0.875268 | 0.2396280 | Not queried | 4.41 | 2.16e-03 | 0.0934794372 |
|  | 19 | 52391078 | 52391789 | PDFSTATSENSEMBLUCSC | 712 | 13 | 22344 | island | 0.970656 | 0.5902092 | CTC-429C10.2; ZNF577; ZNF649 (margin: CTC-429C10.2; ZNF577; ZNF649) | 4.40 | 2.67e-05 | 0.0110819376 |
|  | 14 | 100069535 | 100069840 | PDFSTATSENSEMBLUCSC | 306 | 4 | 12204 | gene;island | 0.360339 | 0.9276096 | Not queried | 4.40 | 2.00e-01 | 0.3826488233 |
|  | 11 | 63258712 | 63258803 | PDFSTATSENSEMBLUCSC | 92 | 6 | 7375 | tss | 0.006487 | 0.0659551 | Not queried | 4.40 | 1.52e-02 | 0.1830867741 |
|  | 11 | 18813466 | 18813556 | PDFSTATSENSEMBLUCSC | 91 | 5 | 7190 | tss;island | 0.206647 | 0.0192811 | Not queried | 4.40 | 5.56e-02 | 0.2315877671 |
|  | 2 | 27665017 | 27665150 | PDFSTATSENSEMBLUCSC | 134 | 6 | 23270 | tss | 0.681965 | 0.9748718 | Not queried | 4.39 | 2.16e-03 | 0.0934794372 |
|  | 8 | 11555178 | 11555548 | PDFSTATSENSEMBLUCSC | 371 | 4 | 40084 | island | 0.028992 | 0.3428891 | Not queried | 4.38 | 5.71e-02 | 0.2315877671 |
|  | 6 | 75912410 | 75912711 | PDFSTATSENSEMBLUCSC | 302 | 4 | 34394 | gene;island | 0.042643 | 0.3364853 | Not queried | 4.38 | 5.71e-02 | 0.2315877671 |
|  | 6 | 36645500 | 36645886 | PDFSTATSENSEMBLUCSC | 387 | 5 | 34940 | tss;island | 0.017759 | 0.4819231 | Not queried | 4.38 | 5.56e-02 | 0.2315877671 |
|  | 10 | 468051 | 468412 | PDFSTATSENSEMBLUCSC | 362 | 5 | 4439 | gene;island | 0.047484 | 0.7374656 | Not queried | 4.38 | 1.51e-01 | 0.3618000441 |
|  | 10 | 75531971 | 75532169 | PDFSTATSENSEMBLUCSC | 199 | 6 | 5856 | island | 0.120887 | 0.0049145 | Not queried | 4.38 | 1.52e-02 | 0.1830867741 |
|  | 6 | 27647713 | 27648057 | PDFSTATSENSEMBLUCSC | 345 | 5 | 35560 | island | 0.086451 | 0.0294615 | Not queried | 4.38 | 2.22e-01 | 0.3935758299 |
|  | 11 | 78673073 | 78673290 | PDFSTATSENSEMBLUCSC | 218 | 4 | 6847 | gene;island | 0.112673 | 0.0156897 | Not queried | 4.37 | 3.43e-01 | 0.4672865596 |
|  | 6 | 28058715 | 28058918 | PDFSTATSENSEMBLUCSC | 204 | 8 | 34731 | tss | 0.495596 | 0.9442076 | ZNF165; ZSCAN12P1 (margin: ZNF165; ZSCAN12P1; RP1-313I6.12) | 4.37 | 1.55e-04 | 0.0296603652 |
|  | 5 | 63461216 | 63461654 | PDFSTATSENSEMBLUCSC | 439 | 8 | 32088 | tss | 0.039060 | 0.3699438 | Not queried | 4.37 | 3.79e-02 | 0.2121545571 |
|  | 7 | 154003215 | 154003309 | PDFSTATSENSEMBLUCSC | 95 | 4 | 37303 | gene;island | 0.101762 | 0.6972957 | Not queried | 4.37 | 2.00e-01 | 0.3826488233 |
|  | 3 | 184279593 | 184279728 | PDFSTATSENSEMBLUCSC | 136 | 4 | 28064 | gene | 0.207339 | 0.0065175 | Not queried | 4.37 | 1.14e-01 | 0.3123457953 |
|  | 2 | 165812079 | 165812297 | PDFSTATSENSEMBLUCSC | 219 | 4 | 23694 | tss | 0.037549 | 0.6399491 | Not queried | 4.37 | 3.43e-01 | 0.4672865596 |
|  | 6 | 10398494 | 10398754 | PDFSTATSENSEMBLUCSC | 261 | 4 | 33556 | gene;island | 0.112681 | 0.0158238 | Not queried | 4.36 | 1.14e-01 | 0.3123457953 |
|  | 12 | 81331457 | 81331845 | PDFSTATSENSEMBLUCSC | 389 | 7 | 10649 | island | 0.085153 | 0.0064375 | Not queried | 4.36 | 3.79e-02 | 0.2121545571 |
|  | 5 | 501863 | 502074 | PDFSTATSENSEMBLUCSC | 212 | 4 | 31510 | gene;island | 0.045455 | 0.5151867 | Not queried | 4.36 | 5.71e-02 | 0.2315877671 |
|  | 10 | 131927345 | 131927554 | PDFSTATSENSEMBLUCSC | 210 | 4 | 6272 | island | 0.256337 | 0.5959698 | Not queried | 4.36 | 4.86e-01 | 0.5640816327 |
|  | 8 | 17270347 | 17270608 | PDFSTATSENSEMBLUCSC | 262 | 4 | 39216 | gene;island | 0.055807 | 0.5020039 | Not queried | 4.36 | 5.71e-02 | 0.2315877671 |
|  | 20 | 22559305 | 22559456 | PDFSTATSENSEMBLUCSC | 152 | 4 | 25632 | tss;island | 0.077749 | 0.6203961 | Not queried | 4.36 | 1.14e-01 | 0.3123457953 |
|  | 1 | 29587088 | 29587490 | PDFSTATSENSEMBLUCSC | 403 | 5 | 377 | gene;island | 0.270422 | 0.6472787 | Not queried | 4.35 | 9.52e-02 | 0.3104222821 |
|  | 3 | 141087187 | 141087363 | PDFSTATSENSEMBLUCSC | 177 | 5 | 27973 | gene | 0.964923 | 0.5638574 | Not queried | 4.35 | 1.59e-02 | 0.1830867741 |
|  | 15 | 40674878 | 40674910 | PDFSTATSENSEMBLUCSC | 33 | 4 | 13588 | tss | 0.298435 | 0.7076900 | Not queried | 4.34 | 3.43e-01 | 0.4672865596 |
|  | 1 | 38941882 | 38942644 | PDFSTATSENSEMBLUCSC | 763 | 7 | 3087 | island | 0.446559 | 0.0342856 | Not queried | 4.33 | 3.79e-02 | 0.2121545571 |
|  | 17 | 35300448 | 35300874 | PDFSTATSENSEMBLUCSC | 427 | 5 | 18238 | island | 0.053014 | 0.6592834 | Not queried | 4.32 | 1.51e-01 | 0.3618000441 |
|  | 12 | 125412894 | 125413164 | PDFSTATSENSEMBLUCSC | 271 | 4 | 10979 | island | 0.035964 | 0.3342672 | Not queried | 4.31 | 2.00e-01 | 0.3826488233 |
|  | 5 | 140105626 | 140105729 | PDFSTATSENSEMBLUCSC | 104 | 6 | 32351 | tss | 0.006287 | 0.1908755 | Not queried | 4.31 | 2.60e-02 | 0.1830867741 |
|  | 10 | 85985296 | 85985448 | PDFSTATSENSEMBLUCSC | 153 | 4 | 5193 | tss | 0.070780 | 0.7874621 | Not queried | 4.31 | 2.00e-01 | 0.3826488233 |
|  | 17 | 3375469 | 3375601 | PDFSTATSENSEMBLUCSC | 133 | 5 | 17011 | tss;island | 0.452920 | 0.7306629 | Not queried | 4.31 | 1.59e-02 | 0.1830867741 |
|  | 11 | 72929368 | 72929553 | PDFSTATSENSEMBLUCSC | 186 | 4 | 6819 | gene | 0.291141 | 0.0132261 | Not queried | 4.30 | 2.86e-02 | 0.1830867741 |
|  | 1 | 27961746 | 27962037 | PDFSTATSENSEMBLUCSC | 292 | 5 | 1416 | tss | 0.052100 | 0.5620780 | Not queried | 4.30 | 5.56e-02 | 0.2315877671 |
|  | 17 | 154410 | 154671 | PDFSTATSENSEMBLUCSC | 262 | 4 | 16288 | gene;island | 0.044994 | 0.5240917 | Not queried | 4.29 | 2.86e-02 | 0.1830867741 |
|  | 21 | 34398085 | 34398226 | PDFSTATSENSEMBLUCSC | 142 | 6 | 26516 | tss | 0.009087 | 0.1323411 | Not queried | 4.29 | 8.66e-03 | 0.1595382395 |
|  | 5 | 158531132 | 158531647 | PDFSTATSENSEMBLUCSC | 516 | 5 | 33351 | island | 0.907852 | 0.4984707 | Not queried | 4.29 | 3.17e-02 | 0.1866936846 |
|  | 14 | 57274550 | 57274900 | PDFSTATSENSEMBLUCSC | 351 | 5 | 12091 | gene;island | 0.095191 | 0.0301228 | Not queried | 4.28 | 3.10e-01 | 0.4672865596 |
|  | 1 | 6265071 | 6265417 | PDFSTATSENSEMBLUCSC | 347 | 4 | 1200 | tss;island | 0.405909 | 0.0141605 | Not queried | 4.28 | 1.14e-01 | 0.3123457953 |
|  | 20 | 1294311 | 1294376 | PDFSTATSENSEMBLUCSC | 66 | 4 | 25537 | tss | 0.004419 | 0.0185784 | Not queried | 4.28 | 4.86e-01 | 0.5640816327 |
|  | 2 | 127413363 | 127413530 | PDFSTATSENSEMBLUCSC | 168 | 5 | 23608 | tss;island | 0.054603 | 0.6023570 | Not queried | 4.28 | 4.21e-01 | 0.5404252807 |
|  | 5 | 79378709 | 79378955 | PDFSTATSENSEMBLUCSC | 247 | 4 | 31662 | gene | 0.497981 | 0.0187632 | Not queried | 4.27 | 6.86e-01 | 0.7223604052 |
|  | 8 | 63997977 | 63998463 | PDFSTATSENSEMBLUCSC | 487 | 5 | 39320 | gene | 0.055443 | 0.0119221 | Not queried | 4.27 | 7.94e-03 | 0.1589602024 |
|  | 14 | 92790072 | 92790364 | PDFSTATSENSEMBLUCSC | 293 | 4 | 12181 | gene;island | 0.005971 | 0.1646884 | Not queried | 4.27 | 3.43e-01 | 0.4672865596 |
|  | 14 | 69256677 | 69256977 | PDFSTATSENSEMBLUCSC | 301 | 5 | 12121 | gene;island | 0.004557 | 0.0146637 | Not queried | 4.27 | 2.22e-01 | 0.3935758299 |
|  | 17 | 80330638 | 80330901 | PDFSTATSENSEMBLUCSC | 264 | 4 | 18988 | island | 0.050327 | 0.5391216 | Not queried | 4.26 | 2.00e-01 | 0.3826488233 |
|  | 1 | 210110998 | 210111162 | PDFSTATSENSEMBLUCSC | 165 | 5 | 2235 | tss;island | 0.060473 | 0.0089126 | Not queried | 4.26 | 1.51e-01 | 0.3618000441 |
|  | 15 | 60296809 | 60296996 | PDFSTATSENSEMBLUCSC | 188 | 4 | 13368 | gene;island | 0.413742 | 0.0286120 | Not queried | 4.26 | 2.86e-02 | 0.1830867741 |
|  | 1 | 91182534 | 91182777 | PDFSTATSENSEMBLUCSC | 244 | 4 | 625 | gene | 0.142601 | 0.5012195 | Not queried | 4.26 | 2.00e-01 | 0.3826488233 |
|  | 2 | 105470558 | 105470794 | PDFSTATSENSEMBLUCSC | 237 | 4 | 23550 | tss;island | 0.125208 | 0.0189009 | Not queried | 4.26 | 6.86e-01 | 0.7223604052 |
|  | 2 | 191207820 | 191208171 | PDFSTATSENSEMBLUCSC | 352 | 4 | 23785 | tss | 0.490906 | 0.0193727 | Not queried | 4.26 | 1.14e-01 | 0.3123457953 |
|  | 3 | 50541011 | 50541195 | PDFSTATSENSEMBLUCSC | 185 | 6 | 28358 | tss;island | 0.128567 | 0.0055968 | Not queried | 4.26 | 2.60e-02 | 0.1830867741 |
|  | 20 | 1294301 | 1294376 | PDFSTATSENSEMBLUCSC | 76 | 5 | 25328 | gene;island | 0.003802 | 0.0192056 | Not queried | 4.26 | 2.22e-01 | 0.3935758299 |
|  | 7 | 44104860 | 44105434 | PDFSTATSENSEMBLUCSC | 575 | 6 | 38454 | island | 0.124768 | 0.5430846 | Not queried | 4.25 | 4.33e-03 | 0.1459191215 |
|  | 7 | 44104860 | 44105166 | PDFSTATSENSEMBLUCSC | 307 | 4 | 36989 | gene | 0.124768 | 0.6634237 | Not queried | 4.25 | 2.86e-02 | 0.1830867741 |
|  | 14 | 23305548 | 23305957 | PDFSTATSENSEMBLUCSC | 410 | 6 | 12703 | island | 0.093865 | 0.6877662 | Not queried | 4.25 | 2.16e-03 | 0.0934794372 |
|  | 3 | 42947263 | 42947771 | PDFSTATSENSEMBLUCSC | 509 | 7 | 29023 | island | 0.026679 | 0.1609467 | Not queried | 4.25 | 2.59e-01 | 0.4381244265 |
|  | 2 | 66667039 | 66667101 | PDFSTATSENSEMBLUCSC | 63 | 5 | 22711 | gene | 0.012587 | 0.1830191 | Not queried | 4.24 | 9.52e-02 | 0.3104222821 |
|  | 3 | 126007325 | 126007563 | PDFSTATSENSEMBLUCSC | 239 | 4 | 29429 | island | 0.026836 | 0.5248609 | Not queried | 4.24 | 1.14e-01 | 0.3123457953 |
|  | 2 | 71558461 | 71558589 | PDFSTATSENSEMBLUCSC | 129 | 4 | 23429 | tss;island | 0.019450 | 0.5041656 | Not queried | 4.24 | 5.71e-02 | 0.2315877671 |
|  | 6 | 27259010 | 27259237 | PDFSTATSENSEMBLUCSC | 228 | 5 | 35553 | island | 0.522824 | 0.0400349 | Not queried | 4.24 | 2.22e-01 | 0.3935758299 |
|  | 3 | 189348936 | 189349129 | PDFSTATSENSEMBLUCSC | 194 | 4 | 28782 | tss | 0.427152 | 0.9075960 | Not queried | 4.23 | 5.71e-02 | 0.2315877671 |
|  | 7 | 27170241 | 27171528 | PDFSTATSENSEMBLUCSC | 1288 | 22 | 38345 | island | 0.873619 | 0.2479343 | Not queried | 4.23 | 8.09e-04 | 0.0698560014 |
|  | 16 | 28995843 | 28996053 | PDFSTATSENSEMBLUCSC | 211 | 6 | 15164 | tss | 0.056829 | 0.4669797 | Not queried | 4.23 | 4.11e-02 | 0.2180389943 |
|  | 17 | 9929698 | 9929867 | PDFSTATSENSEMBLUCSC | 170 | 4 | 17133 | tss | 0.040047 | 0.6140944 | Not queried | 4.23 | 5.71e-02 | 0.2315877671 |
|  | 11 | 129817418 | 129817717 | PDFSTATSENSEMBLUCSC | 300 | 5 | 6970 | gene | 0.498635 | 0.0540366 | Not queried | 4.23 | 7.94e-03 | 0.1589602024 |
|  | 7 | 150652948 | 150653195 | PDFSTATSENSEMBLUCSC | 248 | 5 | 37984 | tss | 0.026037 | 0.1906653 | Not queried | 4.22 | 9.52e-02 | 0.3104222821 |
|  | 12 | 133022423 | 133022955 | PDFSTATSENSEMBLUCSC | 533 | 6 | 11058 | island | 0.942182 | 0.4663202 | Not queried | 4.22 | 2.60e-02 | 0.1830867741 |
|  | 17 | 7323540 | 7323660 | PDFSTATSENSEMBLUCSC | 121 | 4 | 17080 | tss | 0.019639 | 0.2766157 | Not queried | 4.21 | 2.86e-02 | 0.1830867741 |
|  | 17 | 202588 | 202759 | PDFSTATSENSEMBLUCSC | 172 | 4 | 16978 | tss;island | 0.030016 | 0.3091529 | Not queried | 4.21 | 1.14e-01 | 0.3123457953 |
|  | 6 | 100911949 | 100912168 | PDFSTATSENSEMBLUCSC | 220 | 4 | 35140 | tss;island | 0.063734 | 0.4268395 | Not queried | 4.21 | 2.86e-02 | 0.1830867741 |
|  | 1 | 50574383 | 50574591 | PDFSTATSENSEMBLUCSC | 209 | 4 | 524 | gene;tss | 0.203875 | 0.3750304 | Not queried | 4.20 | 4.86e-01 | 0.5640816327 |
|  | 6 | 31237664 | 31238036 | PDFSTATSENSEMBLUCSC | 373 | 5 | 33865 | gene;island | 0.820402 | 0.4674876 | Not queried | 4.20 | 9.52e-02 | 0.3104222821 |
|  | 10 | 18629497 | 18629677 | PDFSTATSENSEMBLUCSC | 181 | 6 | 4522 | gene | 0.466567 | 0.9369457 | Not queried | 4.20 | 1.32e-01 | 0.3397986247 |
|  | 12 | 119419106 | 119419331 | PDFSTATSENSEMBLUCSC | 226 | 4 | 10049 | tss;island | 0.018381 | 0.0047018 | Not queried | 4.19 | 2.00e-01 | 0.3826488233 |
|  | 9 | 133589177 | 133589654 | PDFSTATSENSEMBLUCSC | 478 | 4 | 40961 | island | 0.620226 | 0.0945145 | Not queried | 4.19 | 4.86e-01 | 0.5640816327 |
|  | 11 | 75139390 | 75139736 | PDFSTATSENSEMBLUCSC | 347 | 4 | 6835 | gene;island | 0.497799 | 0.0584431 | Not queried | 4.19 | 4.86e-01 | 0.5640816327 |
|  | 14 | 61748243 | 61748440 | PDFSTATSENSEMBLUCSC | 198 | 4 | 12105 | gene;island | 0.045643 | 0.4363914 | Not queried | 4.18 | 2.86e-02 | 0.1830867741 |
|  | 17 | 46657346 | 46657656 | PDFSTATSENSEMBLUCSC | 311 | 4 | 17461 | tss | 0.098104 | 0.6394960 | Not queried | 4.18 | 2.00e-01 | 0.3826488233 |
|  | 4 | 113970617 | 113970745 | PDFSTATSENSEMBLUCSC | 129 | 4 | 30555 | tss | 0.657963 | 0.0342533 | Not queried | 4.17 | 1.14e-01 | 0.3123457953 |
|  | 1 | 210501936 | 210502156 | PDFSTATSENSEMBLUCSC | 221 | 5 | 991 | gene;tss;island | 0.044014 | 0.0102948 | Not queried | 4.17 | 3.10e-01 | 0.4672865596 |
|  | 1 | 100111713 | 100111949 | PDFSTATSENSEMBLUCSC | 237 | 4 | 648 | gene | 0.006698 | 0.3070426 | Not queried | 4.17 | 3.43e-01 | 0.4672865596 |
|  | 17 | 3819339 | 3819429 | PDFSTATSENSEMBLUCSC | 91 | 4 | 16349 | gene | 0.011213 | 0.1554554 | Not queried | 4.17 | 2.86e-02 | 0.1830867741 |
|  | 17 | 46657274 | 46657656 | PDFSTATSENSEMBLUCSC | 383 | 5 | 18482 | island | 0.065422 | 0.5492457 | Not queried | 4.17 | 3.10e-01 | 0.4672865596 |
|  | 19 | 13134771 | 13134830 | PDFSTATSENSEMBLUCSC | 60 | 4 | 19876 | gene;island | 0.056978 | 0.3671905 | Not queried | 4.16 | 1.14e-01 | 0.3123457953 |
|  | 21 | 34444104 | 34444382 | PDFSTATSENSEMBLUCSC | 279 | 4 | 26402 | gene;island | 0.009564 | 0.0260145 | Not queried | 4.16 | 2.00e-01 | 0.3826488233 |
|  | 17 | 7323540 | 7323730 | PDFSTATSENSEMBLUCSC | 191 | 5 | 17946 | island | 0.021585 | 0.2974361 | Not queried | 4.16 | 1.59e-02 | 0.1830867741 |
|  | 5 | 142065671 | 142065819 | PDFSTATSENSEMBLUCSC | 149 | 5 | 32398 | tss | 0.027041 | 0.3670222 | Not queried | 4.16 | 1.51e-01 | 0.3618000441 |
|  | 19 | 41768003 | 41768357 | PDFSTATSENSEMBLUCSC | 355 | 5 | 20803 | tss | 0.012012 | 0.3758975 | Not queried | 4.16 | 5.48e-01 | 0.6179718485 |
|  | 6 | 84562778 | 84562930 | PDFSTATSENSEMBLUCSC | 153 | 7 | 35097 | tss | 0.017981 | 0.2438455 | Not queried | 4.16 | 1.75e-02 | 0.1830867741 |
|  | 1 | 3477168 | 3477442 | PDFSTATSENSEMBLUCSC | 275 | 4 | 130 | gene | 0.025479 | 0.2553422 | Not queried | 4.16 | 2.00e-01 | 0.3826488233 |
|  | 3 | 191048361 | 191048439 | PDFSTATSENSEMBLUCSC | 79 | 4 | 28789 | tss | 0.001126 | 0.0197534 | Not queried | 4.16 | 2.00e-01 | 0.3826488233 |
|  | 3 | 195622522 | 195622598 | PDFSTATSENSEMBLUCSC | 77 | 4 | 28096 | gene;tss;island | 0.014158 | 0.0028555 | Not queried | 4.15 | 5.71e-02 | 0.2315877671 |
|  | 11 | 102826469 | 102826680 | PDFSTATSENSEMBLUCSC | 212 | 4 | 7650 | tss | 0.130413 | 0.8811823 | Not queried | 4.15 | 2.00e-01 | 0.3826488233 |
|  | 2 | 38304720 | 38304804 | PDFSTATSENSEMBLUCSC | 85 | 4 | 23310 | tss;island | 0.012217 | 0.3227624 | Not queried | 4.15 | 1.14e-01 | 0.3123457953 |
|  | 5 | 142066121 | 142066398 | PDFSTATSENSEMBLUCSC | 278 | 4 | 31844 | gene;tss | 0.016096 | 0.3024416 | Not queried | 4.15 | 2.00e-01 | 0.3826488233 |
|  | 10 | 27541298 | 27541418 | PDFSTATSENSEMBLUCSC | 121 | 4 | 5001 | tss | 0.945614 | 0.5740699 | Not queried | 4.14 | 3.43e-01 | 0.4672865596 |
|  | 6 | 29599012 | 29599390 | PDFSTATSENSEMBLUCSC | 379 | 10 | 33681 | gene;island | 0.522468 | 0.9516596 | GABBR1 (margin: GABBR1; SUMO2P1) | 4.14 | 7.58e-05 | 0.0209443807 |
|  | 7 | 99775422 | 99775558 | PDFSTATSENSEMBLUCSC | 137 | 7 | 37776 | tss;island | 0.150881 | 0.8188137 | Not queried | 4.14 | 4.08e-03 | 0.1459191215 |
|  | 2 | 63275868 | 63276268 | PDFSTATSENSEMBLUCSC | 401 | 6 | 23385 | tss;island | 0.423178 | 0.0401711 | Not queried | 4.14 | 1.32e-01 | 0.3397986247 |
|  | 6 | 136871667 | 136872119 | PDFSTATSENSEMBLUCSC | 453 | 10 | 36501 | island | 0.115338 | 0.0100143 | Not queried | 4.14 | 1.50e-03 | 0.0934794372 |
|  | 16 | 29296614 | 29296797 | PDFSTATSENSEMBLUCSC | 184 | 4 | 15801 | island | 0.544783 | 0.1144939 | Not queried | 4.14 | 2.00e-01 | 0.3826488233 |
|  | 6 | 33739406 | 33739653 | PDFSTATSENSEMBLUCSC | 248 | 4 | 34298 | gene;island | 0.070155 | 0.5359286 | Not queried | 4.14 | 2.00e-01 | 0.3826488233 |
|  | 19 | 15344046 | 15344364 | PDFSTATSENSEMBLUCSC | 319 | 4 | 20558 | tss;island | 0.038474 | 0.3137285 | Not queried | 4.14 | 2.00e-01 | 0.3826488233 |
|  | 2 | 38892846 | 38892958 | PDFSTATSENSEMBLUCSC | 113 | 4 | 23312 | tss | 0.048212 | 0.6300073 | Not queried | 4.13 | 3.43e-01 | 0.4672865596 |
|  | 1 | 156261200 | 156261403 | PDFSTATSENSEMBLUCSC | 204 | 5 | 820 | gene;island | 0.042534 | 0.5784208 | Not queried | 4.13 | 7.94e-03 | 0.1589602024 |
|  | 1 | 91190891 | 91191254 | PDFSTATSENSEMBLUCSC | 364 | 5 | 3462 | island | 0.132433 | 0.4287169 | Not queried | 4.12 | 9.52e-02 | 0.3104222821 |
|  | 17 | 77776428 | 77776716 | PDFSTATSENSEMBLUCSC | 289 | 4 | 18843 | island | 0.010428 | 0.0216421 | Not queried | 4.12 | 4.86e-01 | 0.5640816327 |
|  | 15 | 40674878 | 40675029 | PDFSTATSENSEMBLUCSC | 152 | 6 | 14035 | island | 0.028424 | 0.3568408 | Not queried | 4.12 | 5.89e-01 | 0.6570484697 |
|  | 1 | 202778443 | 202778869 | PDFSTATSENSEMBLUCSC | 427 | 4 | 2181 | tss;island | 0.513659 | 0.9258786 | Not queried | 4.11 | 2.00e-01 | 0.3826488233 |
|  | 17 | 46659751 | 46660002 | PDFSTATSENSEMBLUCSC | 252 | 4 | 18483 | island | 0.097834 | 0.4481147 | Not queried | 4.11 | 3.43e-01 | 0.4672865596 |
|  | 19 | 55691730 | 55692003 | PDFSTATSENSEMBLUCSC | 274 | 7 | 21072 | tss;island | 0.185237 | 0.0351534 | Not queried | 4.10 | 1.28e-01 | 0.3397986247 |
|  | 3 | 139653328 | 139653573 | PDFSTATSENSEMBLUCSC | 246 | 4 | 28634 | tss;island | 0.006524 | 0.0674136 | Not queried | 4.10 | 2.86e-02 | 0.1830867741 |
|  | 8 | 80523191 | 80523461 | PDFSTATSENSEMBLUCSC | 271 | 6 | 40408 | island | 0.071058 | 0.5490984 | Not queried | 4.10 | 1.52e-02 | 0.1830867741 |
|  | 4 | 4860431 | 4860698 | PDFSTATSENSEMBLUCSC | 268 | 9 | 30270 | tss;island | 0.117571 | 0.6122316 | Not queried | 4.10 | 1.06e-02 | 0.1810746987 |
|  | 8 | 144120335 | 144120648 | PDFSTATSENSEMBLUCSC | 314 | 5 | 39943 | tss | 0.047488 | 0.5588885 | Not queried | 4.10 | 7.94e-03 | 0.1589602024 |
|  | 19 | 19648890 | 19649144 | PDFSTATSENSEMBLUCSC | 255 | 8 | 21821 | island | 0.335186 | 0.0328667 | Not queried | 4.09 | 3.79e-02 | 0.2121545571 |
|  | 17 | 72931569 | 72931857 | PDFSTATSENSEMBLUCSC | 289 | 10 | 17637 | tss | 0.057973 | 0.3615983 | Not queried | 4.09 | 1.50e-03 | 0.0934794372 |
|  | 10 | 95848649 | 95848841 | PDFSTATSENSEMBLUCSC | 193 | 4 | 4685 | gene | 0.015329 | 0.1791836 | Not queried | 4.09 | 2.86e-02 | 0.1830867741 |
|  | 17 | 42733527 | 42733729 | PDFSTATSENSEMBLUCSC | 203 | 8 | 17412 | tss;island | 0.354247 | 0.0630491 | Not queried | 4.09 | 6.99e-03 | 0.1589602024 |
|  | 11 | 94883242 | 94883516 | PDFSTATSENSEMBLUCSC | 275 | 4 | 8746 | island | 0.183046 | 0.7557259 | Not queried | 4.08 | 5.71e-02 | 0.2315877671 |
|  | 1 | 231176985 | 231177201 | PDFSTATSENSEMBLUCSC | 217 | 4 | 2320 | tss;island | 0.030851 | 0.4045533 | Not queried | 4.08 | 2.86e-02 | 0.1830867741 |
|  | 15 | 30114814 | 30114912 | PDFSTATSENSEMBLUCSC | 99 | 4 | 13547 | tss | 0.356001 | 0.0381178 | Not queried | 4.08 | 2.00e-01 | 0.3826488233 |
|  | 12 | 119212160 | 119212319 | PDFSTATSENSEMBLUCSC | 160 | 4 | 10871 | island | 0.007981 | 0.3596814 | Not queried | 4.08 | 5.71e-02 | 0.2315877671 |
|  | 11 | 64405764 | 64406051 | PDFSTATSENSEMBLUCSC | 288 | 4 | 6691 | gene;island | 0.774279 | 0.0307032 | Not queried | 4.07 | 1.14e-01 | 0.3123457953 |
|  | 11 | 61519687 | 61520258 | PDFSTATSENSEMBLUCSC | 572 | 6 | 8331 | island | 0.230066 | 0.0163608 | Not queried | 4.07 | 3.94e-01 | 0.5236526859 |
|  | 2 | 200468445 | 200469026 | PDFSTATSENSEMBLUCSC | 582 | 5 | 24994 | island | 0.441209 | 0.8163402 | Not queried | 4.07 | 1.51e-01 | 0.3618000441 |
|  | 12 | 110211321 | 110211413 | PDFSTATSENSEMBLUCSC | 93 | 5 | 9995 | tss | 0.015344 | 0.3419507 | Not queried | 4.07 | 7.94e-03 | 0.1589602024 |
|  | 13 | 98869706 | 98870144 | PDFSTATSENSEMBLUCSC | 439 | 5 | 11198 | gene | 0.628782 | 0.9535963 | Not queried | 4.07 | 9.52e-02 | 0.3104222821 |
|  | 10 | 50341989 | 50342145 | PDFSTATSENSEMBLUCSC | 157 | 4 | 5730 | island | 0.414634 | 0.9031739 | Not queried | 4.07 | 2.86e-02 | 0.1830867741 |
|  | 4 | 144621898 | 144621971 | PDFSTATSENSEMBLUCSC | 74 | 5 | 30615 | tss;island | 0.038986 | 0.2926702 | Not queried | 4.06 | 3.17e-02 | 0.1866936846 |
|  | 2 | 108602784 | 108602979 | PDFSTATSENSEMBLUCSC | 196 | 6 | 23562 | tss | 0.030141 | 0.5087433 | Not queried | 4.06 | 8.66e-03 | 0.1595382395 |
|  | 5 | 140613740 | 140614106 | PDFSTATSENSEMBLUCSC | 367 | 8 | 33226 | island | 0.021037 | 0.2591885 | Not queried | 4.06 | 3.28e-01 | 0.4672865596 |
|  | 15 | 79164541 | 79164980 | PDFSTATSENSEMBLUCSC | 440 | 4 | 13839 | tss;island | 0.295594 | 0.0197964 | Not queried | 4.06 | 4.86e-01 | 0.5640816327 |
|  | 13 | 21900392 | 21900591 | PDFSTATSENSEMBLUCSC | 200 | 4 | 11589 | island | 0.096902 | 0.7359108 | Not queried | 4.05 | 2.86e-02 | 0.1830867741 |
|  | 5 | 140480507 | 140480967 | PDFSTATSENSEMBLUCSC | 461 | 6 | 31807 | gene;island | 0.312893 | 0.7993564 | Not queried | 4.05 | 8.66e-03 | 0.1595382395 |
|  | 4 | 25090198 | 25090665 | PDFSTATSENSEMBLUCSC | 468 | 6 | 30958 | island | 0.444469 | 0.0081244 | Not queried | 4.04 | 4.11e-02 | 0.2180389943 |
|  | 6 | 108492642 | 108492769 | PDFSTATSENSEMBLUCSC | 128 | 4 | 34439 | gene;island | 0.018495 | 0.1343185 | Not queried | 4.04 | 2.86e-02 | 0.1830867741 |
|  | 4 | 46391259 | 46391929 | PDFSTATSENSEMBLUCSC | 671 | 13 | 29995 | gene;island | 0.009602 | 0.1010069 | Not queried | 4.03 | 1.40e-02 | 0.1830867741 |
|  | 4 | 46391414 | 46391929 | PDFSTATSENSEMBLUCSC | 516 | 11 | 30372 | tss | 0.009602 | 0.1010069 | Not queried | 4.03 | 1.28e-02 | 0.1830867741 |
|  | 17 | 77311577 | 77311812 | PDFSTATSENSEMBLUCSC | 236 | 4 | 16839 | gene | 0.080093 | 0.4243768 | Not queried | 4.03 | 2.00e-01 | 0.3826488233 |
|  | 1 | 159869927 | 159870326 | PDFSTATSENSEMBLUCSC | 400 | 7 | 2032 | tss;island | 0.940019 | 0.4066345 | Not queried | 4.03 | 4.08e-03 | 0.1459191215 |
|  | 19 | 38794554 | 38794845 | PDFSTATSENSEMBLUCSC | 292 | 6 | 20014 | gene | 0.864332 | 0.2723031 | Not queried | 4.02 | 2.60e-02 | 0.1830867741 |
|  | 10 | 29834018 | 29834266 | PDFSTATSENSEMBLUCSC | 249 | 4 | 4547 | gene | 0.299761 | 0.8506115 | Not queried | 4.02 | 2.86e-02 | 0.1830867741 |
|  | 5 | 140749735 | 140749834 | PDFSTATSENSEMBLUCSC | 100 | 4 | 31824 | gene;tss;island | 0.047133 | 0.4521275 | Not queried | 4.02 | 2.00e-01 | 0.3826488233 |
|  | 3 | 3885955 | 3886273 | PDFSTATSENSEMBLUCSC | 319 | 4 | 27640 | gene | 0.008473 | 0.1282886 | Not queried | 4.02 | 4.86e-01 | 0.5640816327 |
|  | 18 | 42530692 | 42531132 | PDFSTATSENSEMBLUCSC | 441 | 8 | 19096 | gene | 0.880490 | 0.5371571 | Not queried | 4.02 | 4.99e-02 | 0.2315877671 |
|  | 7 | 31726494 | 31726704 | PDFSTATSENSEMBLUCSC | 211 | 5 | 37523 | tss | 0.566325 | 0.7704594 | Not queried | 4.01 | 5.56e-02 | 0.2315877671 |
|  | 19 | 39785679 | 39786136 | PDFSTATSENSEMBLUCSC | 458 | 4 | 20768 | tss | 0.294433 | 0.6238974 | Not queried | 4.01 | 3.43e-01 | 0.4672865596 |
|  | 8 | 23567310 | 23567941 | PDFSTATSENSEMBLUCSC | 632 | 7 | 40157 | island | 0.052989 | 0.2589032 | Not queried | 4.01 | 2.33e-03 | 0.0974014615 |
|  | 17 | 78700906 | 78701257 | PDFSTATSENSEMBLUCSC | 352 | 4 | 16871 | gene;island | 0.332682 | 0.9167238 | Not queried | 4.01 | 2.86e-02 | 0.1830867741 |
|  | 1 | 47900197 | 47900320 | PDFSTATSENSEMBLUCSC | 124 | 4 | 1610 | tss;island | 0.601565 | 0.1296102 | Not queried | 4.00 | 2.86e-02 | 0.1830867741 |
|  | 2 | 241497412 | 241497846 | PDFSTATSENSEMBLUCSC | 435 | 8 | 23981 | tss;island | 0.526381 | 0.0780420 | Not queried | 4.00 | 6.50e-02 | 0.2477578846 |
|  | 8 | 97169621 | 97170097 | PDFSTATSENSEMBLUCSC | 477 | 5 | 39366 | gene;island | 0.224333 | 0.0128583 | Not queried | 4.00 | 3.10e-01 | 0.4672865596 |
|  | 4 | 71337658 | 71337873 | PDFSTATSENSEMBLUCSC | 216 | 4 | 30016 | gene | 0.374758 | 0.6244061 | Not queried | 4.00 | 6.86e-01 | 0.7223604052 |
|  | 1 | 29460817 | 29461070 | PDFSTATSENSEMBLUCSC | 254 | 4 | 2959 | island | 0.868522 | 0.4591063 | Not queried | 3.99 | 1.14e-01 | 0.3123457953 |
|  | 2 | 43903470 | 43904011 | PDFSTATSENSEMBLUCSC | 542 | 8 | 22660 | gene;tss | 0.487040 | 0.0415703 | Not queried | 3.99 | 6.99e-03 | 0.1589602024 |
|  | 19 | 34174665 | 34175419 | PDFSTATSENSEMBLUCSC | 755 | 19 | 20688 | tss | 0.029174 | 0.4286653 | CHST8 (margin: CHST8) | 3.98 | 6.75e-05 | 0.0209443807 |
|  | 2 | 223163725 | 223163809 | PDFSTATSENSEMBLUCSC | 85 | 4 | 23916 | tss | 0.034811 | 0.2862013 | Not queried | 3.98 | 2.86e-02 | 0.1830867741 |
|  | 12 | 52585786 | 52585892 | PDFSTATSENSEMBLUCSC | 107 | 4 | 9724 | tss | 0.015878 | 0.3925901 | Not queried | 3.98 | 2.00e-01 | 0.3826488233 |
|  | 12 | 25055108 | 25055676 | PDFSTATSENSEMBLUCSC | 569 | 8 | 9083 | gene;island | 0.169279 | 0.7062306 | Not queried | 3.98 | 1.86e-03 | 0.0934794372 |
|  | 17 | 9940004 | 9940321 | PDFSTATSENSEMBLUCSC | 318 | 4 | 16441 | gene | 0.486281 | 0.8820323 | Not queried | 3.98 | 2.00e-01 | 0.3826488233 |
|  | 11 | 20617937 | 20618399 | PDFSTATSENSEMBLUCSC | 463 | 8 | 8128 | island | 0.021672 | 0.0939667 | Not queried | 3.98 | 4.99e-02 | 0.2315877671 |
|  | 10 | 88728073 | 88728361 | PDFSTATSENSEMBLUCSC | 289 | 6 | 5909 | island | 0.160606 | 0.7113797 | Not queried | 3.97 | 4.33e-03 | 0.1459191215 |
|  | 19 | 3369702 | 3369820 | PDFSTATSENSEMBLUCSC | 119 | 4 | 19714 | gene;island | 0.014301 | 0.3843609 | Not queried | 3.96 | 1.14e-01 | 0.3123457953 |
|  | 3 | 128779498 | 128779601 | PDFSTATSENSEMBLUCSC | 104 | 6 | 28576 | tss | 0.021824 | 0.2683000 | Not queried | 3.96 | 2.16e-03 | 0.0934794372 |
|  | 13 | 102068989 | 102069296 | PDFSTATSENSEMBLUCSC | 308 | 8 | 11519 | tss;island | 0.217888 | 0.0194661 | Not queried | 3.96 | 2.34e-01 | 0.4128374386 |
|  | 2 | 154336045 | 154336401 | PDFSTATSENSEMBLUCSC | 357 | 5 | 23669 | tss;island | 0.035778 | 0.6328532 | Not queried | 3.95 | 3.10e-01 | 0.4672865596 |
|  | 10 | 94825547 | 94825848 | PDFSTATSENSEMBLUCSC | 302 | 4 | 4679 | gene;island | 0.117326 | 0.0189496 | Not queried | 3.95 | 1.14e-01 | 0.3123457953 |
|  | 5 | 1174426 | 1174666 | PDFSTATSENSEMBLUCSC | 241 | 4 | 32611 | island | 0.064733 | 0.5128787 | Not queried | 3.95 | 1.14e-01 | 0.3123457953 |
|  | 11 | 61519687 | 61520084 | PDFSTATSENSEMBLUCSC | 398 | 5 | 7337 | tss | 0.168782 | 0.0221014 | Not queried | 3.95 | 8.41e-01 | 0.8635565145 |
|  | 17 | 40834743 | 40835003 | PDFSTATSENSEMBLUCSC | 261 | 4 | 16585 | gene;tss;island | 0.009290 | 0.0676510 | Not queried | 3.95 | 5.71e-02 | 0.2315877671 |
|  | 10 | 636076 | 636390 | PDFSTATSENSEMBLUCSC | 315 | 5 | 4444 | gene | 0.046241 | 0.1281579 | Not queried | 3.95 | 2.22e-01 | 0.3935758299 |
|  | 6 | 31513198 | 31513306 | PDFSTATSENSEMBLUCSC | 109 | 5 | 33896 | gene;tss;island | 0.023645 | 0.4710397 | Not queried | 3.95 | 2.22e-01 | 0.3935758299 |
|  | 6 | 32073324 | 32073700 | PDFSTATSENSEMBLUCSC | 377 | 5 | 34048 | gene | 0.479689 | 0.6848821 | Not queried | 3.94 | 2.22e-01 | 0.3935758299 |
|  | 22 | 30476089 | 30476525 | PDFSTATSENSEMBLUCSC | 437 | 11 | 27404 | island | 0.115289 | 0.6566366 | Not queried | 3.94 | 1.86e-03 | 0.0934794372 |
|  | 20 | 21378045 | 21378278 | PDFSTATSENSEMBLUCSC | 234 | 8 | 26040 | island | 0.400387 | 0.0290619 | Not queried | 3.94 | 1.04e-02 | 0.1806165940 |
|  | 10 | 115998843 | 115999009 | PDFSTATSENSEMBLUCSC | 167 | 4 | 5376 | tss | 0.008597 | 0.0021103 | Not queried | 3.93 | 6.86e-01 | 0.7223604052 |
|  | 4 | 94748617 | 94748860 | PDFSTATSENSEMBLUCSC | 244 | 4 | 30500 | tss;island | 0.480003 | 0.0576001 | Not queried | 3.93 | 2.86e-02 | 0.1830867741 |
|  | 10 | 105212236 | 105212813 | PDFSTATSENSEMBLUCSC | 578 | 11 | 5338 | tss | 0.031576 | 0.5254705 | Not queried | 3.93 | 1.04e-02 | 0.1806165940 |
|  | 6 | 32846924 | 32847845 | PDFSTATSENSEMBLUCSC | 922 | 27 | 34160 | gene;island | 0.968773 | 0.4912706 | PPP1R2P1 (margin: PPP1R2P1) | 3.92 | 1.74e-05 | 0.0080291795 |
|  | 14 | 104314434 | 104314734 | PDFSTATSENSEMBLUCSC | 301 | 6 | 12654 | tss;island | 0.020896 | 0.0010466 | Not queried | 3.92 | 1.32e-01 | 0.3397986247 |
|  | 20 | 1471884 | 1472098 | PDFSTATSENSEMBLUCSC | 215 | 4 | 25329 | gene | 0.046458 | 0.4230188 | Not queried | 3.91 | 2.86e-02 | 0.1830867741 |
|  | 2 | 220299484 | 220299672 | PDFSTATSENSEMBLUCSC | 189 | 7 | 23906 | tss | 0.208188 | 0.0410133 | Not queried | 3.91 | 9.73e-02 | 0.3123457953 |
|  | 21 | 43198283 | 43198797 | PDFSTATSENSEMBLUCSC | 515 | 6 | 26694 | island | 0.069966 | 0.4170170 | Not queried | 3.91 | 1.32e-01 | 0.3397986247 |
|  | 1 | 233462575 | 233462803 | PDFSTATSENSEMBLUCSC | 229 | 5 | 2326 | tss;island | 0.655854 | 0.5343208 | Not queried | 3.90 | 3.10e-01 | 0.4672865596 |
|  | 15 | 90437628 | 90437891 | PDFSTATSENSEMBLUCSC | 264 | 5 | 13893 | tss | 0.172879 | 0.0138283 | Not queried | 3.90 | 1.51e-01 | 0.3618000441 |
|  | 17 | 50237205 | 50237844 | PDFSTATSENSEMBLUCSC | 640 | 9 | 17515 | tss;island | 0.017996 | 0.3111019 | Not queried | 3.90 | 1.88e-02 | 0.1830867741 |
|  | 10 | 119295619 | 119295875 | PDFSTATSENSEMBLUCSC | 257 | 4 | 4783 | gene;island | 0.510507 | 0.0397518 | Not queried | 3.90 | 2.00e-01 | 0.3826488233 |
|  | 12 | 54321992 | 54322396 | PDFSTATSENSEMBLUCSC | 405 | 4 | 10476 | island | 0.010275 | 0.0763197 | Not queried | 3.89 | 5.71e-02 | 0.2315877671 |
|  | 1 | 62660556 | 62660861 | PDFSTATSENSEMBLUCSC | 306 | 4 | 562 | gene | 0.032150 | 0.3760301 | Not queried | 3.89 | 3.43e-01 | 0.4672865596 |
|  | 5 | 138628911 | 138629210 | PDFSTATSENSEMBLUCSC | 300 | 8 | 31772 | gene;tss;island | 0.012451 | 0.0015043 | Not queried | 3.89 | 2.34e-01 | 0.4128374386 |
|  | 5 | 175108269 | 175108429 | PDFSTATSENSEMBLUCSC | 161 | 4 | 31907 | gene;tss | 0.030800 | 0.4051231 | Not queried | 3.89 | 2.00e-01 | 0.3826488233 |
|  | 18 | 49868101 | 49868552 | PDFSTATSENSEMBLUCSC | 452 | 7 | 19116 | gene;island | 0.055344 | 0.5090845 | Not queried | 3.88 | 2.62e-02 | 0.1830867741 |
|  | 7 | 23287222 | 23287560 | PDFSTATSENSEMBLUCSC | 339 | 4 | 36914 | gene;island | 0.152555 | 0.0151860 | Not queried | 3.88 | 2.86e-02 | 0.1830867741 |
|  | 18 | 5891948 | 5892245 | PDFSTATSENSEMBLUCSC | 298 | 4 | 19376 | island | 0.114238 | 0.6425463 | Not queried | 3.88 | 2.86e-02 | 0.1830867741 |
|  | 17 | 151914 | 152350 | PDFSTATSENSEMBLUCSC | 437 | 6 | 16287 | gene;island | 0.067967 | 0.5745881 | Not queried | 3.88 | 8.66e-03 | 0.1595382395 |
|  | 19 | 48897863 | 48898160 | PDFSTATSENSEMBLUCSC | 298 | 4 | 22214 | island | 0.090583 | 0.0176275 | Not queried | 3.88 | 1.14e-01 | 0.3123457953 |
|  | 1 | 74663583 | 74663827 | PDFSTATSENSEMBLUCSC | 245 | 5 | 583 | gene | 0.079821 | 0.0021672 | Not queried | 3.88 | 1.51e-01 | 0.3618000441 |
|  | 11 | 843897 | 844686 | PDFSTATSENSEMBLUCSC | 790 | 11 | 6393 | gene;island | 0.022883 | 0.4512571 | Not queried | 3.88 | 2.70e-01 | 0.4563244066 |
|  | 7 | 157179773 | 157180124 | PDFSTATSENSEMBLUCSC | 352 | 6 | 37320 | gene | 0.971951 | 0.5703864 | Not queried | 3.87 | 1.32e-01 | 0.3397986247 |
|  | 15 | 79724739 | 79724802 | PDFSTATSENSEMBLUCSC | 64 | 4 | 13845 | tss;island | 0.047337 | 0.3531323 | Not queried | 3.87 | 2.86e-02 | 0.1830867741 |
|  | 20 | 22566877 | 22567170 | PDFSTATSENSEMBLUCSC | 294 | 5 | 25634 | tss;island | 0.030750 | 0.3472412 | Not queried | 3.86 | 7.94e-03 | 0.1589602024 |
|  | 1 | 1369466 | 1369948 | PDFSTATSENSEMBLUCSC | 483 | 6 | 1152 | tss;island | 0.030071 | 0.3479041 | Not queried | 3.86 | 2.16e-03 | 0.0934794372 |
|  | 2 | 26401640 | 26402098 | PDFSTATSENSEMBLUCSC | 459 | 6 | 22607 | gene;island | 0.215096 | 0.0246505 | Not queried | 3.86 | 3.94e-01 | 0.5236526859 |
|  | 8 | 86350278 | 86350592 | PDFSTATSENSEMBLUCSC | 315 | 6 | 39794 | tss;island | 0.022782 | 0.3454082 | Not queried | 3.85 | 6.49e-02 | 0.2477578846 |
|  | 2 | 237076164 | 237076497 | PDFSTATSENSEMBLUCSC | 334 | 4 | 23089 | gene;island | 0.025000 | 0.2260679 | Not queried | 3.85 | 2.00e-01 | 0.3826488233 |
|  | 7 | 95402189 | 95402528 | PDFSTATSENSEMBLUCSC | 340 | 4 | 37099 | gene;island | 0.037608 | 0.3221802 | Not queried | 3.84 | 2.86e-02 | 0.1830867741 |
|  | 11 | 65816655 | 65816985 | PDFSTATSENSEMBLUCSC | 331 | 6 | 7457 | tss | 0.099287 | 0.7064180 | Not queried | 3.84 | 2.60e-02 | 0.1830867741 |
|  | 21 | 45705175 | 45705742 | PDFSTATSENSEMBLUCSC | 568 | 10 | 26582 | tss;island | 0.535180 | 0.9210059 | Not queried | 3.84 | 2.32e-02 | 0.1830867741 |
|  | 16 | 1583810 | 1584118 | PDFSTATSENSEMBLUCSC | 309 | 6 | 14574 | gene;tss;island | 0.019600 | 0.2081214 | Not queried | 3.83 | 4.33e-03 | 0.1459191215 |
|  | 6 | 159290283 | 159290589 | PDFSTATSENSEMBLUCSC | 307 | 4 | 36600 | island | 0.007667 | 0.0783309 | Not queried | 3.83 | 2.00e-01 | 0.3826488233 |
|  | 11 | 1474386 | 1474841 | PDFSTATSENSEMBLUCSC | 456 | 5 | 6421 | gene;island | 0.135048 | 0.5391844 | Not queried | 3.83 | 5.56e-02 | 0.2315877671 |
|  | 11 | 43602845 | 43602965 | PDFSTATSENSEMBLUCSC | 121 | 7 | 8217 | island | 0.023412 | 0.0109467 | Not queried | 3.83 | 7.28e-02 | 0.2713481487 |
|  | 13 | 113776873 | 113776964 | PDFSTATSENSEMBLUCSC | 92 | 4 | 11957 | island | 0.256018 | 0.7088707 | Not queried | 3.82 | 2.00e-01 | 0.3826488233 |
|  | 5 | 79330669 | 79331135 | PDFSTATSENSEMBLUCSC | 467 | 7 | 32918 | island | 0.522758 | 0.0234624 | Not queried | 3.82 | 2.62e-02 | 0.1830867741 |
|  | 5 | 79330669 | 79330959 | PDFSTATSENSEMBLUCSC | 291 | 5 | 32148 | tss | 0.522758 | 0.0234624 | Not queried | 3.82 | 2.22e-01 | 0.3935758299 |
|  | 19 | 17791074 | 17791354 | PDFSTATSENSEMBLUCSC | 281 | 4 | 19919 | gene;island | 0.027428 | 0.0091032 | Not queried | 3.81 | 4.86e-01 | 0.5640816327 |
|  | 15 | 30114295 | 30114912 | PDFSTATSENSEMBLUCSC | 618 | 7 | 13977 | island | 0.088496 | 0.0138696 | Not queried | 3.81 | 3.79e-02 | 0.2121545571 |
|  | 15 | 45028083 | 45028270 | PDFSTATSENSEMBLUCSC | 188 | 4 | 13642 | tss | 0.014021 | 0.1522310 | Not queried | 3.81 | 1.14e-01 | 0.3123457953 |
|  | 2 | 223166909 | 223167617 | PDFSTATSENSEMBLUCSC | 709 | 8 | 23050 | gene;island | 0.123163 | 0.0159047 | Not queried | 3.80 | 4.99e-02 | 0.2315877671 |
|  | 1 | 54821853 | 54822298 | PDFSTATSENSEMBLUCSC | 446 | 6 | 548 | gene;island | 0.131434 | 0.5678403 | Not queried | 3.80 | 2.60e-02 | 0.1830867741 |
|  | 5 | 76934327 | 76934696 | PDFSTATSENSEMBLUCSC | 370 | 6 | 32902 | island | 0.330684 | 0.0473785 | Not queried | 3.80 | 6.49e-02 | 0.2477578846 |
|  | 7 | 76029648 | 76029881 | PDFSTATSENSEMBLUCSC | 234 | 5 | 37057 | gene;island | 0.808568 | 0.9832735 | Not queried | 3.80 | 9.52e-02 | 0.3104222821 |
|  | 14 | 53258566 | 53258684 | PDFSTATSENSEMBLUCSC | 119 | 5 | 12400 | tss;island | 0.651294 | 0.0157519 | Not queried | 3.80 | 5.48e-01 | 0.6179718485 |
|  | 6 | 29705815 | 29706048 | PDFSTATSENSEMBLUCSC | 234 | 4 | 33689 | gene | 0.253532 | 0.0267591 | Not queried | 3.80 | 6.86e-01 | 0.7223604052 |
|  | 8 | 110986685 | 110987019 | PDFSTATSENSEMBLUCSC | 335 | 5 | 40542 | island | 0.386988 | 0.0063599 | Not queried | 3.79 | 3.17e-02 | 0.1866936846 |
|  | 6 | 33143721 | 33143807 | PDFSTATSENSEMBLUCSC | 87 | 4 | 34204 | gene | 0.261299 | 0.6283601 | Not queried | 3.79 | 3.43e-01 | 0.4672865596 |
|  | 5 | 132155233 | 132155555 | PDFSTATSENSEMBLUCSC | 323 | 5 | 33101 | island | 0.471365 | 0.0611205 | Not queried | 3.79 | 1.51e-01 | 0.3618000441 |
|  | 11 | 65408429 | 65408566 | PDFSTATSENSEMBLUCSC | 138 | 4 | 6714 | gene;island | 0.010906 | 0.2196516 | Not queried | 3.78 | 3.43e-01 | 0.4672865596 |
|  | 6 | 127796989 | 127797286 | PDFSTATSENSEMBLUCSC | 298 | 4 | 34470 | gene;island | 0.523373 | 0.8893552 | Not queried | 3.78 | 1.14e-01 | 0.3123457953 |
|  | 6 | 83073156 | 83073335 | PDFSTATSENSEMBLUCSC | 180 | 4 | 34400 | gene;tss;island | 0.018892 | 0.2987068 | Not queried | 3.78 | 2.86e-02 | 0.1830867741 |
|  | 1 | 3620638 | 3621048 | PDFSTATSENSEMBLUCSC | 411 | 5 | 140 | gene;island | 0.582126 | 0.1294325 | Not queried | 3.78 | 7.94e-03 | 0.1589602024 |
|  | 7 | 4832112 | 4832535 | PDFSTATSENSEMBLUCSC | 424 | 5 | 38201 | island | 0.968040 | 0.5590610 | Not queried | 3.77 | 1.59e-02 | 0.1830867741 |
|  | 11 | 76380921 | 76381236 | PDFSTATSENSEMBLUCSC | 316 | 8 | 6837 | gene;island | 0.028251 | 0.4276290 | Not queried | 3.77 | 6.99e-03 | 0.1589602024 |
|  | 11 | 76381044 | 76381236 | PDFSTATSENSEMBLUCSC | 193 | 6 | 7576 | tss | 0.020703 | 0.4276290 | Not queried | 3.77 | 1.52e-02 | 0.1830867741 |
|  | 6 | 5994956 | 5995150 | PDFSTATSENSEMBLUCSC | 195 | 4 | 35402 | island | 0.350249 | 0.0319115 | Not queried | 3.77 | 5.71e-02 | 0.2315877671 |
|  | 1 | 201857840 | 201857955 | PDFSTATSENSEMBLUCSC | 116 | 6 | 942 | gene | 0.249450 | 0.0292499 | Not queried | 3.77 | 1.52e-02 | 0.1830867741 |
|  | 18 | 5197327 | 5197559 | PDFSTATSENSEMBLUCSC | 233 | 6 | 19166 | tss;island | 0.034881 | 0.5083288 | Not queried | 3.77 | 4.11e-02 | 0.2180389943 |
|  | 5 | 1416793 | 1417003 | PDFSTATSENSEMBLUCSC | 211 | 4 | 31538 | gene;island | 0.639431 | 0.9423936 | Not queried | 3.76 | 2.86e-02 | 0.1830867741 |
|  | 7 | 27170412 | 27171528 | PDFSTATSENSEMBLUCSC | 1117 | 18 | 37491 | tss | 0.872610 | 0.2539824 | Not queried | 3.76 | 1.42e-02 | 0.1830867741 |
|  | 3 | 128205143 | 128205496 | PDFSTATSENSEMBLUCSC | 354 | 5 | 27936 | gene;island | 0.029275 | 0.1053049 | Not queried | 3.76 | 1.51e-01 | 0.3618000441 |
|  | 5 | 135415693 | 135416613 | PDFSTATSENSEMBLUCSC | 921 | 16 | 33146 | island | 0.843647 | 0.4093294 | VTRNA2-1 (margin: VTRNA2-1) | 3.75 | 7.07e-05 | 0.0209443807 |
|  | 5 | 135416331 | 135416613 | PDFSTATSENSEMBLUCSC | 283 | 9 | 32310 | tss | 0.921520 | 0.4695498 | Not queried | 3.75 | 4.00e-02 | 0.2180389943 |
|  | 17 | 8647944 | 8648193 | PDFSTATSENSEMBLUCSC | 250 | 5 | 18007 | island | 0.721660 | 0.9789607 | Not queried | 3.75 | 6.90e-01 | 0.7223604052 |
|  | 7 | 54610022 | 54610320 | PDFSTATSENSEMBLUCSC | 299 | 4 | 37017 | gene | 0.040272 | 0.2893149 | Not queried | 3.75 | 2.00e-01 | 0.3826488233 |
|  | 18 | 3447454 | 3447713 | PDFSTATSENSEMBLUCSC | 260 | 4 | 19040 | gene;island | 0.018586 | 0.0957538 | Not queried | 3.74 | 3.43e-01 | 0.4672865596 |
|  | 7 | 138720786 | 138721065 | PDFSTATSENSEMBLUCSC | 280 | 9 | 37927 | tss;island | 0.018056 | 0.3537297 | Not queried | 3.74 | 3.99e-03 | 0.1459191215 |
|  | 5 | 43000303 | 43000890 | PDFSTATSENSEMBLUCSC | 588 | 6 | 32764 | island | 0.297950 | 0.7019960 | Not queried | 3.74 | 1.32e-01 | 0.3397986247 |
|  | 8 | 80523191 | 80523364 | PDFSTATSENSEMBLUCSC | 174 | 4 | 39780 | tss | 0.081583 | 0.4500296 | Not queried | 3.74 | 2.00e-01 | 0.3826488233 |
|  | 16 | 18995157 | 18995532 | PDFSTATSENSEMBLUCSC | 376 | 5 | 15112 | tss | 0.044428 | 0.0113492 | Not queried | 3.74 | 2.22e-01 | 0.3935758299 |
|  | 5 | 176520024 | 176520502 | PDFSTATSENSEMBLUCSC | 479 | 5 | 31916 | gene;island | 0.628298 | 0.9408619 | Not queried | 3.74 | 5.56e-02 | 0.2315877671 |
|  | 17 | 39969264 | 39969397 | PDFSTATSENSEMBLUCSC | 134 | 4 | 16572 | gene;tss;island | 0.154001 | 0.6875490 | Not queried | 3.73 | 1.14e-01 | 0.3123457953 |
|  | 10 | 129535138 | 129535509 | PDFSTATSENSEMBLUCSC | 372 | 6 | 5436 | tss | 0.013752 | 0.1889653 | Not queried | 3.73 | 2.60e-02 | 0.1830867741 |
|  | 7 | 48019205 | 48019713 | PDFSTATSENSEMBLUCSC | 509 | 5 | 38481 | island | 0.038628 | 0.6851785 | Not queried | 3.73 | 4.21e-01 | 0.5404252807 |
|  | 10 | 105212056 | 105212813 | PDFSTATSENSEMBLUCSC | 758 | 12 | 6102 | island | 0.027339 | 0.5248855 | Not queried | 3.73 | 8.29e-03 | 0.1595382395 |
|  | 19 | 39694477 | 39694831 | PDFSTATSENSEMBLUCSC | 355 | 4 | 20026 | gene | 0.053589 | 0.1006625 | Not queried | 3.73 | 4.86e-01 | 0.5640816327 |
|  | 5 | 140501321 | 140501812 | PDFSTATSENSEMBLUCSC | 492 | 10 | 33211 | island | 0.144654 | 0.5006071 | Not queried | 3.72 | 2.88e-02 | 0.1834529198 |
|  | 17 | 7117125 | 7117310 | PDFSTATSENSEMBLUCSC | 186 | 4 | 16383 | gene;island | 0.069625 | 0.5451692 | Not queried | 3.72 | 2.00e-01 | 0.3826488233 |
|  | 5 | 76936361 | 76936793 | PDFSTATSENSEMBLUCSC | 433 | 4 | 32903 | island | 0.345499 | 0.0349317 | Not queried | 3.72 | 1.14e-01 | 0.3123457953 |
|  | 2 | 120189867 | 120190297 | PDFSTATSENSEMBLUCSC | 431 | 4 | 22846 | gene;island | 0.042452 | 0.3765866 | Not queried | 3.72 | 2.00e-01 | 0.3826488233 |
|  | 5 | 112849156 | 112849285 | PDFSTATSENSEMBLUCSC | 130 | 4 | 32220 | tss | 0.005403 | 0.1567673 | Not queried | 3.71 | 4.86e-01 | 0.5640816327 |
|  | 19 | 45720771 | 45721076 | PDFSTATSENSEMBLUCSC | 306 | 4 | 20089 | gene;island | 0.010775 | 0.2216448 | Not queried | 3.71 | 1.14e-01 | 0.3123457953 |
|  | 1 | 85358968 | 85359134 | PDFSTATSENSEMBLUCSC | 167 | 4 | 1717 | tss;island | 0.060814 | 0.0067531 | Not queried | 3.71 | 5.71e-02 | 0.2315877671 |
|  | 2 | 223163573 | 223163809 | PDFSTATSENSEMBLUCSC | 237 | 5 | 23048 | gene;island | 0.035590 | 0.4007198 | Not queried | 3.71 | 7.94e-03 | 0.1589602024 |
|  | 10 | 91597207 | 91597593 | PDFSTATSENSEMBLUCSC | 387 | 4 | 5927 | island | 0.043749 | 0.4905347 | Not queried | 3.70 | 2.86e-02 | 0.1830867741 |
|  | 1 | 65731689 | 65731886 | PDFSTATSENSEMBLUCSC | 198 | 4 | 568 | gene;island | 0.013867 | 0.1030364 | Not queried | 3.70 | 2.00e-01 | 0.3826488233 |
|  | 7 | 1083072 | 1083473 | PDFSTATSENSEMBLUCSC | 402 | 4 | 36740 | gene;island | 0.940180 | 0.6756889 | Not queried | 3.70 | 1.14e-01 | 0.3123457953 |
|  | 11 | 637032 | 637175 | PDFSTATSENSEMBLUCSC | 144 | 7 | 7021 | tss;island | 0.022499 | 0.1574262 | Not queried | 3.70 | 2.62e-02 | 0.1830867741 |
|  | 17 | 56232260 | 56232499 | PDFSTATSENSEMBLUCSC | 240 | 4 | 17532 | tss | 0.008439 | 0.1930153 | Not queried | 3.70 | 3.43e-01 | 0.4672865596 |
|  | 6 | 30510238 | 30510459 | PDFSTATSENSEMBLUCSC | 222 | 4 | 33773 | gene | 0.011301 | 0.2836384 | Not queried | 3.70 | 5.71e-02 | 0.2315877671 |
|  | 12 | 106533840 | 106533903 | PDFSTATSENSEMBLUCSC | 64 | 5 | 9966 | tss | 0.248463 | 0.0290661 | Not queried | 3.70 | 2.22e-01 | 0.3935758299 |
|  | 1 | 1293868 | 1294140 | PDFSTATSENSEMBLUCSC | 273 | 4 | 2429 | island | 0.061033 | 0.4190615 | Not queried | 3.69 | 2.86e-02 | 0.1830867741 |
|  | 17 | 9929528 | 9929867 | PDFSTATSENSEMBLUCSC | 340 | 5 | 16440 | gene | 0.053440 | 0.6254988 | Not queried | 3.69 | 1.51e-01 | 0.3618000441 |
|  | 5 | 36607333 | 36607417 | PDFSTATSENSEMBLUCSC | 85 | 4 | 31587 | gene;tss | 0.017997 | 0.1031753 | Not queried | 3.69 | 5.71e-02 | 0.2315877671 |
|  | 11 | 111169284 | 111169473 | PDFSTATSENSEMBLUCSC | 190 | 4 | 6897 | gene;tss;island | 0.751269 | 0.9659356 | Not queried | 3.69 | 5.71e-02 | 0.2315877671 |
|  | 6 | 31025933 | 31026287 | PDFSTATSENSEMBLUCSC | 355 | 6 | 33849 | gene | 0.076510 | 0.4839170 | Not queried | 3.68 | 6.49e-02 | 0.2477578846 |
|  | 1 | 182921660 | 182921857 | PDFSTATSENSEMBLUCSC | 198 | 4 | 912 | gene;island | 0.876215 | 0.3363280 | Not queried | 3.68 | 2.00e-01 | 0.3826488233 |
|  | 3 | 101498198 | 101498279 | PDFSTATSENSEMBLUCSC | 82 | 4 | 28480 | tss | 0.007655 | 0.0014265 | Not queried | 3.68 | 1.14e-01 | 0.3123457953 |
|  | 17 | 79031376 | 79031712 | PDFSTATSENSEMBLUCSC | 337 | 5 | 16890 | gene | 0.028216 | 0.3014919 | Not queried | 3.68 | 3.10e-01 | 0.4672865596 |
|  | 15 | 31621629 | 31621843 | PDFSTATSENSEMBLUCSC | 215 | 4 | 13273 | gene;island | 0.142527 | 0.4588002 | Not queried | 3.68 | 6.86e-01 | 0.7223604052 |
|  | 5 | 63461216 | 63461930 | PDFSTATSENSEMBLUCSC | 715 | 10 | 32828 | island | 0.005984 | 0.2497589 | Not queried | 3.68 | 2.32e-02 | 0.1830867741 |
|  | 6 | 33138421 | 33138669 | PDFSTATSENSEMBLUCSC | 249 | 4 | 34200 | gene | 0.245873 | 0.8021846 | Not queried | 3.68 | 2.00e-01 | 0.3826488233 |
|  | 8 | 216453 | 216788 | PDFSTATSENSEMBLUCSC | 336 | 5 | 40001 | island | 0.078027 | 0.8129365 | Not queried | 3.68 | 3.17e-02 | 0.1866936846 |
|  | 12 | 81331457 | 81331660 | PDFSTATSENSEMBLUCSC | 204 | 4 | 9227 | gene | 0.071927 | 0.0057282 | Not queried | 3.67 | 2.86e-02 | 0.1830867741 |
|  | 3 | 157813327 | 157813810 | PDFSTATSENSEMBLUCSC | 484 | 6 | 29616 | island | 0.483687 | 0.0332860 | Not queried | 3.67 | 6.49e-02 | 0.2477578846 |
|  | 1 | 46951291 | 46951683 | PDFSTATSENSEMBLUCSC | 393 | 6 | 3219 | island | 0.259827 | 0.0539460 | Not queried | 3.66 | 3.10e-01 | 0.4672865596 |
|  | 2 | 236772900 | 236773260 | PDFSTATSENSEMBLUCSC | 361 | 4 | 23083 | gene;island | 0.250828 | 0.5793988 | Not queried | 3.66 | 1.14e-01 | 0.3123457953 |
|  | 5 | 131892242 | 131892523 | PDFSTATSENSEMBLUCSC | 282 | 5 | 32272 | tss | 0.007403 | 0.4551062 | Not queried | 3.66 | 4.21e-01 | 0.5404252807 |
|  | 6 | 31794278 | 31794519 | PDFSTATSENSEMBLUCSC | 242 | 4 | 34840 | tss;island | 0.031943 | 0.4542175 | Not queried | 3.66 | 2.00e-01 | 0.3826488233 |
|  | 2 | 45237689 | 45237929 | PDFSTATSENSEMBLUCSC | 241 | 4 | 23335 | tss;island | 0.033517 | 0.3418514 | Not queried | 3.65 | 5.71e-02 | 0.2315877671 |
|  | 1 | 52521668 | 52521894 | PDFSTATSENSEMBLUCSC | 227 | 4 | 1628 | tss;island | 0.002389 | 0.0017682 | Not queried | 3.65 | 8.86e-01 | 0.8941250130 |
|  | 1 | 202183264 | 202183605 | PDFSTATSENSEMBLUCSC | 342 | 5 | 945 | gene;island | 0.155809 | 0.4957891 | Not queried | 3.65 | 2.22e-01 | 0.3935758299 |
|  | 10 | 119302050 | 119302318 | PDFSTATSENSEMBLUCSC | 269 | 8 | 4785 | gene;island | 0.295870 | 0.0127514 | Not queried | 3.65 | 2.81e-02 | 0.1830867741 |
|  | 17 | 68128754 | 68128923 | PDFSTATSENSEMBLUCSC | 170 | 4 | 16743 | gene | 0.339581 | 0.8093192 | Not queried | 3.64 | 2.00e-01 | 0.3826488233 |
|  | 2 | 219906245 | 219906468 | PDFSTATSENSEMBLUCSC | 224 | 7 | 23889 | tss | 0.010265 | 0.0977714 | Not queried | 3.64 | 1.28e-01 | 0.3397986247 |
|  | 11 | 5617703 | 5617926 | PDFSTATSENSEMBLUCSC | 224 | 5 | 6485 | gene | 0.019773 | 0.0367787 | Not queried | 3.64 | 1.51e-01 | 0.3618000441 |
|  | 4 | 1043421 | 1043631 | PDFSTATSENSEMBLUCSC | 211 | 5 | 30762 | island | 0.485973 | 0.7521422 | Not queried | 3.63 | 1.59e-02 | 0.1830867741 |
|  | 10 | 42863508 | 42863594 | PDFSTATSENSEMBLUCSC | 87 | 4 | 5039 | tss | 0.275602 | 0.0267964 | Not queried | 3.63 | 2.00e-01 | 0.3826488233 |
|  | 20 | 25039658 | 25039812 | PDFSTATSENSEMBLUCSC | 155 | 5 | 25644 | tss;island | 0.036501 | 0.3214981 | Not queried | 3.63 | 3.17e-02 | 0.1866936846 |
|  | 22 | 42095154 | 42095536 | PDFSTATSENSEMBLUCSC | 383 | 7 | 27529 | island | 0.484775 | 0.0925817 | Not queried | 3.63 | 2.62e-02 | 0.1830867741 |
|  | 17 | 72931569 | 72932048 | PDFSTATSENSEMBLUCSC | 480 | 12 | 18731 | island | 0.071701 | 0.4133572 | Not queried | 3.63 | 8.58e-04 | 0.0725921168 |
|  | 10 | 100995599 | 100995821 | PDFSTATSENSEMBLUCSC | 223 | 4 | 6005 | island | 0.108431 | 0.0220037 | Not queried | 3.62 | 2.86e-02 | 0.1830867741 |
|  | 1 | 32801964 | 32802312 | PDFSTATSENSEMBLUCSC | 349 | 8 | 1458 | tss;island | 0.063611 | 0.3766846 | Not queried | 3.62 | 1.61e-01 | 0.3814043454 |
|  | 17 | 78161467 | 78161572 | PDFSTATSENSEMBLUCSC | 106 | 5 | 17722 | tss | 0.458785 | 0.9001853 | Not queried | 3.62 | 9.52e-02 | 0.3104222821 |
|  | 7 | 153584582 | 153584873 | PDFSTATSENSEMBLUCSC | 292 | 6 | 37301 | gene | 0.297042 | 0.6974766 | Not queried | 3.62 | 2.40e-01 | 0.4133265075 |
|  | 11 | 7695165 | 7695528 | PDFSTATSENSEMBLUCSC | 364 | 7 | 7121 | tss;island | 0.047491 | 0.3776778 | Not queried | 3.62 | 1.65e-01 | 0.3826488233 |
|  | 6 | 33134045 | 33134325 | PDFSTATSENSEMBLUCSC | 281 | 4 | 34196 | gene | 0.031177 | 0.2592363 | Not queried | 3.61 | 1.14e-01 | 0.3123457953 |
|  | 13 | 28493913 | 28494177 | PDFSTATSENSEMBLUCSC | 265 | 8 | 11633 | island | 0.551103 | 0.0625737 | Not queried | 3.61 | 2.81e-02 | 0.1830867741 |
|  | 11 | 46615333 | 46615693 | PDFSTATSENSEMBLUCSC | 361 | 4 | 8252 | island | 0.010758 | 0.0071118 | Not queried | 3.61 | 6.86e-01 | 0.7223604052 |
|  | 8 | 28174814 | 28175021 | PDFSTATSENSEMBLUCSC | 208 | 5 | 39261 | gene | 0.770122 | 0.4701871 | Not queried | 3.61 | 9.52e-02 | 0.3104222821 |
|  | 7 | 12610355 | 12610833 | PDFSTATSENSEMBLUCSC | 479 | 6 | 36896 | gene | 0.033826 | 0.0871635 | Not queried | 3.60 | 4.85e-01 | 0.5640816327 |
|  | 7 | 55640396 | 55640714 | PDFSTATSENSEMBLUCSC | 319 | 4 | 37604 | tss;island | 0.046584 | 0.0211315 | Not queried | 3.60 | 3.43e-01 | 0.4672865596 |
|  | 19 | 39523309 | 39523626 | PDFSTATSENSEMBLUCSC | 318 | 5 | 20764 | tss | 0.566968 | 0.5661579 | Not queried | 3.60 | 5.48e-01 | 0.6179718485 |
|  | 2 | 115419537 | 115419846 | PDFSTATSENSEMBLUCSC | 310 | 4 | 22838 | gene;island | 0.043575 | 0.4855960 | Not queried | 3.60 | 5.71e-02 | 0.2315877671 |
|  | 19 | 35940425 | 35940593 | PDFSTATSENSEMBLUCSC | 169 | 4 | 20703 | tss | 0.628681 | 0.9454819 | Not queried | 3.60 | 2.00e-01 | 0.3826488233 |
|  | 3 | 15311021 | 15311269 | PDFSTATSENSEMBLUCSC | 249 | 4 | 27690 | gene | 0.429156 | 0.0694086 | Not queried | 3.60 | 2.86e-02 | 0.1830867741 |
|  | 3 | 42304966 | 42305237 | PDFSTATSENSEMBLUCSC | 272 | 5 | 27731 | gene;island | 0.142586 | 0.0180864 | Not queried | 3.60 | 3.17e-02 | 0.1866936846 |
|  | 11 | 63258509 | 63258803 | PDFSTATSENSEMBLUCSC | 295 | 8 | 8369 | island | 0.008959 | 0.0854652 | Not queried | 3.60 | 4.66e-03 | 0.1498346615 |
|  | 19 | 47617042 | 47617301 | PDFSTATSENSEMBLUCSC | 260 | 7 | 20902 | tss;island | 0.015233 | 0.0040454 | Not queried | 3.60 | 2.59e-01 | 0.4381244265 |
|  | 12 | 103889772 | 103890274 | PDFSTATSENSEMBLUCSC | 503 | 9 | 9952 | tss | 0.172701 | 0.0242318 | Not queried | 3.59 | 7.77e-03 | 0.1589602024 |
|  | 13 | 28562798 | 28562902 | PDFSTATSENSEMBLUCSC | 105 | 7 | 11367 | tss | 0.781919 | 0.9703374 | Not queried | 3.59 | 3.79e-02 | 0.2121545571 |
|  | 8 | 35092687 | 35092876 | PDFSTATSENSEMBLUCSC | 190 | 4 | 39643 | tss;island | 0.057986 | 0.7298806 | Not queried | 3.59 | 3.43e-01 | 0.4672865596 |
|  | 7 | 150652702 | 150653195 | PDFSTATSENSEMBLUCSC | 494 | 8 | 37279 | gene;island | 0.038011 | 0.2229411 | Not queried | 3.59 | 1.95e-01 | 0.3826488233 |
|  | 6 | 33397511 | 33397959 | PDFSTATSENSEMBLUCSC | 449 | 5 | 34289 | gene;island | 0.027653 | 0.0728588 | Not queried | 3.59 | 2.22e-01 | 0.3935758299 |
|  | 7 | 153584416 | 153584873 | PDFSTATSENSEMBLUCSC | 458 | 7 | 39053 | island | 0.214607 | 0.5445993 | Not queried | 3.59 | 1.28e-01 | 0.3397986247 |
|  | 10 | 15761854 | 15762312 | PDFSTATSENSEMBLUCSC | 459 | 8 | 4964 | tss | 0.054852 | 0.4355350 | Not queried | 3.59 | 2.07e-02 | 0.1830867741 |
|  | 5 | 66124395 | 66124588 | PDFSTATSENSEMBLUCSC | 194 | 5 | 32102 | tss | 0.002604 | 0.0124195 | Not queried | 3.59 | 3.10e-01 | 0.4672865596 |
|  | 5 | 140723331 | 140723583 | PDFSTATSENSEMBLUCSC | 253 | 6 | 32373 | tss | 0.349565 | 0.0177258 | Not queried | 3.59 | 9.31e-02 | 0.3104222821 |
|  | 1 | 39284267 | 39284609 | PDFSTATSENSEMBLUCSC | 343 | 4 | 3092 | island | 0.016437 | 0.2738193 | Not queried | 3.58 | 3.43e-01 | 0.4672865596 |
|  | 15 | 90437601 | 90437891 | PDFSTATSENSEMBLUCSC | 291 | 6 | 14443 | island | 0.169234 | 0.0110290 | Not queried | 3.58 | 3.10e-01 | 0.4672865596 |
|  | 11 | 121986961 | 121987203 | PDFSTATSENSEMBLUCSC | 243 | 7 | 6948 | gene;tss | 0.025171 | 0.4237142 | Not queried | 3.58 | 7.28e-02 | 0.2713481487 |
|  | 17 | 27346732 | 27347260 | PDFSTATSENSEMBLUCSC | 529 | 6 | 18164 | island | 0.011890 | 0.1769034 | Not queried | 3.58 | 6.49e-02 | 0.2477578846 |
|  | 12 | 117797056 | 117797385 | PDFSTATSENSEMBLUCSC | 330 | 4 | 9338 | gene;island | 0.030130 | 0.4064809 | Not queried | 3.58 | 1.14e-01 | 0.3123457953 |
|  | 8 | 27468981 | 27469338 | PDFSTATSENSEMBLUCSC | 358 | 5 | 39257 | gene;tss;island | 0.020508 | 0.1857063 | Not queried | 3.57 | 9.52e-02 | 0.3104222821 |
|  | 2 | 45159434 | 45159663 | PDFSTATSENSEMBLUCSC | 230 | 4 | 24259 | island | 0.022680 | 0.3294366 | Not queried | 3.57 | 5.71e-02 | 0.2315877671 |
|  | 12 | 115122028 | 115122304 | PDFSTATSENSEMBLUCSC | 277 | 6 | 10036 | tss;island | 0.164730 | 0.0410793 | Not queried | 3.57 | 1.32e-01 | 0.3397986247 |
|  | 1 | 91195159 | 91195683 | PDFSTATSENSEMBLUCSC | 525 | 5 | 3465 | island | 0.228807 | 0.0715302 | Not queried | 3.57 | 3.10e-01 | 0.4672865596 |
|  | 1 | 175568216 | 175568710 | PDFSTATSENSEMBLUCSC | 495 | 4 | 895 | gene;island | 0.012066 | 0.0303908 | Not queried | 3.57 | 2.86e-02 | 0.1830867741 |
|  | 6 | 33386967 | 33387205 | PDFSTATSENSEMBLUCSC | 239 | 6 | 34908 | tss;island | 0.647067 | 0.9553141 | Not queried | 3.57 | 1.32e-01 | 0.3397986247 |
|  | 1 | 91182534 | 91183051 | PDFSTATSENSEMBLUCSC | 518 | 7 | 3458 | island | 0.221207 | 0.5677069 | Not queried | 3.56 | 1.28e-01 | 0.3397986247 |
|  | 22 | 38218945 | 38219400 | PDFSTATSENSEMBLUCSC | 456 | 5 | 27475 | island | 0.061730 | 0.4372888 | Not queried | 3.56 | 1.51e-01 | 0.3618000441 |
|  | 1 | 94884039 | 94884189 | PDFSTATSENSEMBLUCSC | 151 | 4 | 642 | gene;island | 0.005226 | 0.0000791 | Not queried | 3.56 | 1.14e-01 | 0.3123457953 |
|  | 19 | 45927563 | 45927696 | PDFSTATSENSEMBLUCSC | 134 | 4 | 20871 | tss;island | 0.007057 | 0.0768458 | Not queried | 3.56 | 1.14e-01 | 0.3123457953 |
|  | 6 | 28477957 | 28478579 | PDFSTATSENSEMBLUCSC | 623 | 8 | 33632 | gene | 0.079288 | 0.4603745 | Not queried | 3.56 | 4.99e-02 | 0.2315877671 |
|  | 7 | 20826128 | 20826345 | PDFSTATSENSEMBLUCSC | 218 | 4 | 36910 | gene;island | 0.009959 | 0.1200229 | Not queried | 3.56 | 5.71e-02 | 0.2315877671 |
|  | 6 | 31631638 | 31631801 | PDFSTATSENSEMBLUCSC | 164 | 7 | 33931 | gene;island | 0.388209 | 0.9096620 | Not queried | 3.56 | 1.75e-02 | 0.1830867741 |
|  | 19 | 58545001 | 58545333 | PDFSTATSENSEMBLUCSC | 333 | 8 | 21131 | tss;island | 0.099317 | 0.4381719 | Not queried | 3.55 | 1.05e-01 | 0.3123457953 |
|  | 17 | 72438885 | 72439217 | PDFSTATSENSEMBLUCSC | 333 | 4 | 16757 | gene | 0.078851 | 0.2521795 | Not queried | 3.55 | 4.86e-01 | 0.5640816327 |
|  | 17 | 8702698 | 8702896 | PDFSTATSENSEMBLUCSC | 199 | 6 | 17122 | tss | 0.615246 | 0.9487782 | Not queried | 3.55 | 1.52e-02 | 0.1830867741 |
|  | 1 | 11752109 | 11752494 | PDFSTATSENSEMBLUCSC | 386 | 4 | 226 | gene;tss;island | 0.018022 | 0.2666639 | Not queried | 3.55 | 3.43e-01 | 0.4672865596 |
|  | 15 | 26327718 | 26328092 | PDFSTATSENSEMBLUCSC | 375 | 4 | 13962 | island | 0.027222 | 0.3772468 | Not queried | 3.54 | 2.00e-01 | 0.3826488233 |
|  | 3 | 55508379 | 55508686 | PDFSTATSENSEMBLUCSC | 308 | 4 | 27837 | gene;island | 0.488037 | 0.9305234 | Not queried | 3.54 | 5.71e-02 | 0.2315877671 |
|  | 3 | 49236800 | 49236918 | PDFSTATSENSEMBLUCSC | 119 | 5 | 28324 | tss | 0.749150 | 0.2325625 | Not queried | 3.54 | 3.17e-02 | 0.1866936846 |
|  | 4 | 85402870 | 85403409 | PDFSTATSENSEMBLUCSC | 540 | 5 | 31158 | island | 0.060851 | 0.4270387 | Not queried | 3.54 | 1.51e-01 | 0.3618000441 |
|  | 1 | 1897585 | 1897959 | PDFSTATSENSEMBLUCSC | 375 | 4 | 51 | gene;island | 0.147175 | 0.4683700 | Not queried | 3.53 | 2.86e-02 | 0.1830867741 |
|  | 13 | 49549440 | 49549665 | PDFSTATSENSEMBLUCSC | 226 | 4 | 11437 | tss;island | 0.006744 | 0.1433550 | Not queried | 3.53 | 2.00e-01 | 0.3826488233 |
|  | 8 | 22560922 | 22561238 | PDFSTATSENSEMBLUCSC | 317 | 5 | 40142 | island | 0.098048 | 0.3303944 | Not queried | 3.53 | 1.59e-02 | 0.1830867741 |
|  | 19 | 18548929 | 18549274 | PDFSTATSENSEMBLUCSC | 346 | 7 | 20619 | tss | 0.019897 | 0.1919573 | Not queried | 3.53 | 1.75e-02 | 0.1830867741 |
|  | 7 | 1708566 | 1708892 | PDFSTATSENSEMBLUCSC | 327 | 4 | 38122 | island | 0.437782 | 0.0691259 | Not queried | 3.52 | 3.43e-01 | 0.4672865596 |
|  | 1 | 21835945 | 21836326 | PDFSTATSENSEMBLUCSC | 382 | 4 | 297 | gene;island | 0.246608 | 0.0329425 | Not queried | 3.52 | 3.43e-01 | 0.4672865596 |
|  | 2 | 219905959 | 219906468 | PDFSTATSENSEMBLUCSC | 510 | 10 | 25103 | island | 0.008367 | 0.1113302 | Not queried | 3.52 | 2.88e-02 | 0.1834529198 |
|  | 3 | 62304470 | 62304779 | PDFSTATSENSEMBLUCSC | 310 | 5 | 28424 | tss;island | 0.028231 | 0.2808430 | Not queried | 3.52 | 7.94e-03 | 0.1589602024 |
|  | 7 | 2059796 | 2060179 | PDFSTATSENSEMBLUCSC | 384 | 6 | 36786 | gene;island | 0.503158 | 0.9258758 | Not queried | 3.52 | 1.32e-01 | 0.3397986247 |
|  | 3 | 65342546 | 65342971 | PDFSTATSENSEMBLUCSC | 426 | 5 | 27852 | gene;island | 0.081258 | 0.3936234 | Not queried | 3.51 | 5.56e-02 | 0.2315877671 |
|  | 8 | 21881998 | 21882104 | PDFSTATSENSEMBLUCSC | 107 | 4 | 39578 | tss;island | 0.015063 | 0.2540440 | Not queried | 3.51 | 1.14e-01 | 0.3123457953 |
|  | 4 | 1800154 | 1800448 | PDFSTATSENSEMBLUCSC | 295 | 4 | 29875 | gene;island | 0.233785 | 0.6175343 | Not queried | 3.51 | 3.43e-01 | 0.4672865596 |
|  | 7 | 27183133 | 27184853 | PDFSTATSENSEMBLUCSC | 1721 | 47 | 38347 | island | 0.452023 | 0.9059644 | HOXA-AS3; HOXA3; HOXA5; HOXA6 (margin: HOXA-AS2; HOXA-AS3; HOXA3; HOXA5; HOXA6; HOXA7) | 3.50 | 3.47e-11 | 0.0000000719 |
|  | 1 | 32708234 | 32708485 | PDFSTATSENSEMBLUCSC | 252 | 5 | 1456 | tss;island | 0.055494 | 0.4538287 | Not queried | 3.50 | 1.51e-01 | 0.3618000441 |
|  | 8 | 142517341 | 142517553 | PDFSTATSENSEMBLUCSC | 213 | 5 | 39930 | tss | 0.033302 | 0.1309394 | Not queried | 3.50 | 1.51e-01 | 0.3618000441 |
|  | 8 | 17271067 | 17271119 | PDFSTATSENSEMBLUCSC | 53 | 4 | 39566 | tss;island | 0.094621 | 0.5346114 | Not queried | 3.50 | 2.86e-02 | 0.1830867741 |
|  | 2 | 137748366 | 137748460 | PDFSTATSENSEMBLUCSC | 95 | 4 | 23651 | tss | 0.023032 | 0.1586009 | Not queried | 3.50 | 2.00e-01 | 0.3826488233 |
|  | 16 | 84681507 | 84681729 | PDFSTATSENSEMBLUCSC | 223 | 4 | 15393 | tss;island | 0.264722 | 0.6276138 | Not queried | 3.49 | 6.86e-01 | 0.7223604052 |
|  | 19 | 42806146 | 42806265 | PDFSTATSENSEMBLUCSC | 120 | 7 | 20823 | tss | 0.037594 | 0.2489556 | Not queried | 3.49 | 1.65e-01 | 0.3826488233 |
|  | 10 | 695844 | 696063 | PDFSTATSENSEMBLUCSC | 220 | 6 | 4449 | gene | 0.438396 | 0.7636201 | Not queried | 3.49 | 1.32e-01 | 0.3397986247 |
|  | 1 | 150947832 | 150948030 | PDFSTATSENSEMBLUCSC | 199 | 5 | 1916 | tss;island | 0.732467 | 0.0300704 | Not queried | 3.49 | 1.51e-01 | 0.3618000441 |
|  | 5 | 83017000 | 83017184 | PDFSTATSENSEMBLUCSC | 185 | 4 | 32160 | tss;island | 0.728577 | 0.4729371 | Not queried | 3.49 | 8.86e-01 | 0.8941250130 |
|  | 7 | 149569715 | 149570040 | PDFSTATSENSEMBLUCSC | 326 | 8 | 37972 | tss | 0.016180 | 0.5613354 | Not queried | 3.48 | 2.81e-02 | 0.1830867741 |
|  | 11 | 46366643 | 46367100 | PDFSTATSENSEMBLUCSC | 458 | 8 | 6598 | gene;island | 0.065114 | 0.3209312 | Not queried | 3.48 | 6.99e-03 | 0.1589602024 |
|  | 1 | 47915553 | 47915951 | PDFSTATSENSEMBLUCSC | 399 | 6 | 3241 | island | 0.018629 | 0.3780236 | Not queried | 3.48 | 9.31e-02 | 0.3104222821 |
|  | 15 | 79382548 | 79383167 | PDFSTATSENSEMBLUCSC | 620 | 7 | 13447 | gene;island | 0.018426 | 0.0168678 | Not queried | 3.48 | 3.18e-01 | 0.4672865596 |
|  | 17 | 38465281 | 38465422 | PDFSTATSENSEMBLUCSC | 142 | 5 | 17329 | tss | 0.008191 | 0.3783177 | Not queried | 3.48 | 3.17e-02 | 0.1866936846 |
|  | 16 | 1480790 | 1480992 | PDFSTATSENSEMBLUCSC | 203 | 4 | 15542 | island | 0.537876 | 0.0792839 | Not queried | 3.48 | 3.43e-01 | 0.4672865596 |
|  | 2 | 20190016 | 20190349 | PDFSTATSENSEMBLUCSC | 334 | 9 | 23207 | tss | 0.097668 | 0.0297289 | Not queried | 3.47 | 6.25e-02 | 0.2477578846 |
|  | 1 | 147782116 | 147782558 | PDFSTATSENSEMBLUCSC | 443 | 5 | 3702 | island | 0.488496 | 0.8835660 | Not queried | 3.47 | 7.94e-03 | 0.1589602024 |
|  | 6 | 78176473 | 78176847 | PDFSTATSENSEMBLUCSC | 375 | 6 | 36270 | island | 0.203778 | 0.0244126 | Not queried | 3.47 | 4.11e-02 | 0.2180389943 |
|  | 19 | 18548845 | 18549274 | PDFSTATSENSEMBLUCSC | 430 | 8 | 21779 | island | 0.017662 | 0.1421337 | Not queried | 3.46 | 4.99e-02 | 0.2315877671 |
|  | 11 | 31822243 | 31822526 | PDFSTATSENSEMBLUCSC | 284 | 4 | 6557 | gene;island | 0.703707 | 0.3298915 | Not queried | 3.46 | 3.43e-01 | 0.4672865596 |
|  | 17 | 78865087 | 78865755 | PDFSTATSENSEMBLUCSC | 669 | 7 | 16881 | gene;island | 0.064827 | 0.6768798 | Not queried | 3.46 | 9.73e-02 | 0.3123457953 |
|  | 1 | 145473005 | 145473392 | PDFSTATSENSEMBLUCSC | 388 | 6 | 725 | gene;island | 0.004405 | 0.0458573 | Not queried | 3.46 | 1.52e-02 | 0.1830867741 |
|  | 1 | 165325030 | 165325231 | PDFSTATSENSEMBLUCSC | 202 | 4 | 2063 | tss | 0.110275 | 0.0171501 | Not queried | 3.46 | 5.71e-02 | 0.2315877671 |
|  | 13 | 21520203 | 21520519 | PDFSTATSENSEMBLUCSC | 317 | 4 | 11582 | island | 0.301211 | 0.0285169 | Not queried | 3.46 | 1.14e-01 | 0.3123457953 |
|  | 17 | 78161467 | 78161706 | PDFSTATSENSEMBLUCSC | 240 | 6 | 16859 | gene;island | 0.469575 | 0.8872667 | Not queried | 3.46 | 6.49e-02 | 0.2477578846 |
|  | 12 | 103311088 | 103311761 | PDFSTATSENSEMBLUCSC | 674 | 9 | 10726 | island | 0.020166 | 0.1515947 | Not queried | 3.45 | 1.88e-02 | 0.1830867741 |
|  | 2 | 176952696 | 176952930 | PDFSTATSENSEMBLUCSC | 235 | 4 | 24890 | island | 0.046260 | 0.2868037 | Not queried | 3.45 | 2.00e-01 | 0.3826488233 |
|  | 1 | 50893469 | 50893984 | PDFSTATSENSEMBLUCSC | 516 | 5 | 3254 | island | 0.497596 | 0.0477150 | Not queried | 3.45 | 1.51e-01 | 0.3618000441 |
|  | 4 | 118006405 | 118006832 | PDFSTATSENSEMBLUCSC | 428 | 9 | 31264 | island | 0.206485 | 0.0434089 | Not queried | 3.44 | 1.90e-01 | 0.3826488233 |
|  | 3 | 52001778 | 52002169 | PDFSTATSENSEMBLUCSC | 392 | 4 | 28368 | tss | 0.003916 | 0.0111635 | Not queried | 3.44 | 5.71e-02 | 0.2315877671 |
|  | 11 | 7041194 | 7041570 | PDFSTATSENSEMBLUCSC | 377 | 8 | 6494 | gene | 0.183882 | 0.0441096 | Not queried | 3.44 | 6.99e-03 | 0.1589602024 |
|  | 8 | 65291287 | 65291703 | PDFSTATSENSEMBLUCSC | 417 | 8 | 39733 | tss | 0.063410 | 0.3499809 | Not queried | 3.44 | 2.07e-02 | 0.1830867741 |
|  | 11 | 115374969 | 115375226 | PDFSTATSENSEMBLUCSC | 258 | 4 | 6913 | gene | 0.094766 | 0.5002125 | Not queried | 3.44 | 1.14e-01 | 0.3123457953 |
|  | 20 | 54824316 | 54824646 | PDFSTATSENSEMBLUCSC | 331 | 4 | 25460 | gene;island | 0.032035 | 0.3896165 | Not queried | 3.43 | 3.43e-01 | 0.4672865596 |
|  | 2 | 1286216 | 1286641 | PDFSTATSENSEMBLUCSC | 426 | 5 | 22528 | gene;island | 0.549962 | 0.6042107 | Not queried | 3.43 | 1.51e-01 | 0.3618000441 |
|  | 15 | 74421523 | 74421935 | PDFSTATSENSEMBLUCSC | 413 | 12 | 13789 | tss;island | 0.058561 | 0.0090383 | ISLR2; RP11-247C2.2 (margin: ISLR2; RP11-247C2.2) | 3.43 | 2.74e-04 | 0.0455223165 |
|  | 14 | 65005951 | 65006359 | PDFSTATSENSEMBLUCSC | 409 | 10 | 12458 | tss;island | 0.091199 | 0.5319735 | Not queried | 3.43 | 1.65e-01 | 0.3826488233 |
|  | 9 | 137030463 | 137030823 | PDFSTATSENSEMBLUCSC | 361 | 5 | 40853 | tss;island | 0.018871 | 0.2175678 | Not queried | 3.43 | 4.21e-01 | 0.5404252807 |
|  | 21 | 39644216 | 39644354 | PDFSTATSENSEMBLUCSC | 139 | 4 | 26426 | gene;tss | 0.271289 | 0.7190326 | Not queried | 3.43 | 1.14e-01 | 0.3123457953 |
|  | 7 | 149569715 | 149570367 | PDFSTATSENSEMBLUCSC | 653 | 13 | 37268 | gene;island | 0.016894 | 0.4575367 | Not queried | 3.43 | 2.35e-03 | 0.0974014615 |
|  | 19 | 51529846 | 51529999 | PDFSTATSENSEMBLUCSC | 154 | 4 | 20189 | gene | 0.231626 | 0.4876721 | Not queried | 3.43 | 4.86e-01 | 0.5640816327 |
|  | 1 | 154377364 | 154377621 | PDFSTATSENSEMBLUCSC | 258 | 6 | 1963 | tss | 0.007779 | 0.1079220 | Not queried | 3.43 | 4.11e-02 | 0.2180389943 |
|  | 19 | 46526333 | 46526675 | PDFSTATSENSEMBLUCSC | 343 | 7 | 20889 | tss | 0.038033 | 0.4326887 | Not queried | 3.43 | 2.62e-02 | 0.1830867741 |
|  | 2 | 208635549 | 208636363 | PDFSTATSENSEMBLUCSC | 815 | 10 | 25044 | island | 0.952530 | 0.2397046 | Not queried | 3.42 | 3.53e-01 | 0.4803603739 |
|  | 6 | 56112392 | 56112696 | PDFSTATSENSEMBLUCSC | 305 | 8 | 35044 | tss | 0.017206 | 0.3490909 | Not queried | 3.42 | 1.30e-01 | 0.3397986247 |
|  | 15 | 25425326 | 25425615 | PDFSTATSENSEMBLUCSC | 290 | 4 | 13527 | tss | 0.137366 | 0.3158142 | Not queried | 3.42 | 3.43e-01 | 0.4672865596 |
|  | 5 | 87968528 | 87968749 | PDFSTATSENSEMBLUCSC | 222 | 4 | 31677 | gene;island | 0.572392 | 0.1786603 | Not queried | 3.41 | 2.86e-02 | 0.1830867741 |
|  | 6 | 35732234 | 35732480 | PDFSTATSENSEMBLUCSC | 247 | 4 | 36075 | island | 0.296577 | 0.0243639 | Not queried | 3.41 | 2.00e-01 | 0.3826488233 |
|  | 4 | 40751717 | 40751845 | PDFSTATSENSEMBLUCSC | 129 | 4 | 30360 | tss | 0.381311 | 0.0460266 | Not queried | 3.41 | 1.14e-01 | 0.3123457953 |
|  | 6 | 90597340 | 90597591 | PDFSTATSENSEMBLUCSC | 252 | 4 | 36326 | island | 0.266265 | 0.5854380 | Not queried | 3.41 | 5.71e-02 | 0.2315877671 |
|  | 20 | 61637360 | 61637856 | PDFSTATSENSEMBLUCSC | 497 | 4 | 25496 | gene;island | 0.643545 | 0.0642121 | Not queried | 3.41 | 1.14e-01 | 0.3123457953 |
|  | 2 | 177001256 | 177001426 | PDFSTATSENSEMBLUCSC | 171 | 4 | 24906 | island | 0.111067 | 0.5733336 | Not queried | 3.40 | 2.86e-02 | 0.1830867741 |
|  | 20 | 44540794 | 44541153 | PDFSTATSENSEMBLUCSC | 360 | 5 | 25783 | tss | 0.019293 | 0.2967710 | Not queried | 3.40 | 3.10e-01 | 0.4672865596 |
|  | 7 | 96649956 | 96650668 | PDFSTATSENSEMBLUCSC | 713 | 8 | 37105 | gene;island | 0.015460 | 0.0216470 | Not queried | 3.40 | 4.42e-01 | 0.5640816327 |
|  | 15 | 42500502 | 42500843 | PDFSTATSENSEMBLUCSC | 342 | 6 | 13614 | tss | 0.016822 | 0.2240995 | Not queried | 3.40 | 1.80e-01 | 0.3826488233 |
|  | 12 | 8332808 | 8333192 | PDFSTATSENSEMBLUCSC | 385 | 5 | 9049 | gene | 0.041202 | 0.3114807 | Not queried | 3.40 | 3.17e-02 | 0.1866936846 |
|  | 10 | 124638756 | 124639410 | PDFSTATSENSEMBLUCSC | 655 | 16 | 4799 | gene;tss;island | 0.009031 | 0.0898760 | Not queried | 3.40 | 4.67e-04 | 0.0548331102 |
|  | 3 | 46792023 | 46792462 | PDFSTATSENSEMBLUCSC | 440 | 5 | 29060 | island | 0.535143 | 0.7289656 | Not queried | 3.39 | 5.48e-01 | 0.6179718485 |
|  | 21 | 40984760 | 40984886 | PDFSTATSENSEMBLUCSC | 127 | 5 | 26552 | tss | 0.069451 | 0.2562267 | Not queried | 3.39 | 3.17e-02 | 0.1866936846 |
|  | 10 | 95361132 | 95361386 | PDFSTATSENSEMBLUCSC | 255 | 5 | 5239 | tss;island | 0.315542 | 0.0832746 | Not queried | 3.39 | 4.21e-01 | 0.5404252807 |
|  | 12 | 25538177 | 25538380 | PDFSTATSENSEMBLUCSC | 204 | 4 | 10292 | island | 0.106589 | 0.3004271 | Not queried | 3.39 | 6.86e-01 | 0.7223604052 |
|  | 20 | 13200929 | 13200992 | PDFSTATSENSEMBLUCSC | 64 | 10 | 25601 | tss | 0.034365 | 0.1740090 | ISM1 (margin: ISM1) | 3.39 | 7.58e-05 | 0.0209443807 |
|  | 20 | 13200685 | 13200992 | PDFSTATSENSEMBLUCSC | 308 | 12 | 26007 | island | 0.026418 | 0.1161884 | ISM1 (margin: ISM1) | 3.39 | 1.03e-04 | 0.0236793538 |
|  | 13 | 36705446 | 36705622 | PDFSTATSENSEMBLUCSC | 177 | 5 | 11665 | island | 0.019439 | 0.0707086 | Not queried | 3.39 | 1.59e-02 | 0.1830867741 |
|  | 6 | 94129412 | 94129697 | PDFSTATSENSEMBLUCSC | 286 | 8 | 35125 | tss | 0.032464 | 0.1662164 | Not queried | 3.39 | 4.66e-03 | 0.1498346615 |
|  | 19 | 19383613 | 19384165 | PDFSTATSENSEMBLUCSC | 553 | 5 | 21810 | island | 0.577667 | 0.0594986 | Not queried | 3.39 | 5.56e-02 | 0.2315877671 |
|  | 11 | 22359188 | 22359658 | PDFSTATSENSEMBLUCSC | 471 | 7 | 7202 | tss;island | 0.105813 | 0.0552101 | Not queried | 3.39 | 3.18e-01 | 0.4672865596 |
|  | 11 | 65816463 | 65816985 | PDFSTATSENSEMBLUCSC | 523 | 10 | 8497 | island | 0.105831 | 0.5137191 | Not queried | 3.38 | 2.18e-01 | 0.3935758299 |
|  | 22 | 23522307 | 23522511 | PDFSTATSENSEMBLUCSC | 205 | 7 | 27008 | tss | 0.578523 | 0.0270550 | Not queried | 3.38 | 3.79e-02 | 0.2121545571 |
|  | 11 | 2029309 | 2029688 | PDFSTATSENSEMBLUCSC | 380 | 4 | 7948 | island | 0.405414 | 0.7116371 | Not queried | 3.38 | 4.86e-01 | 0.5640816327 |
|  | 4 | 2956627 | 2957353 | PDFSTATSENSEMBLUCSC | 727 | 6 | 29897 | gene;island | 0.807849 | 0.9760357 | Not queried | 3.38 | 4.85e-01 | 0.5640816327 |
|  | 12 | 29650692 | 29651041 | PDFSTATSENSEMBLUCSC | 350 | 6 | 9623 | tss | 0.317257 | 0.6312738 | Not queried | 3.38 | 2.16e-03 | 0.0934794372 |
|  | 4 | 81118188 | 81118588 | PDFSTATSENSEMBLUCSC | 401 | 4 | 30033 | gene;tss;island | 0.010490 | 0.0983330 | Not queried | 3.38 | 1.14e-01 | 0.3123457953 |
|  | 1 | 85512957 | 85513315 | PDFSTATSENSEMBLUCSC | 359 | 4 | 604 | gene;island | 0.615441 | 0.9507791 | Not queried | 3.38 | 2.86e-02 | 0.1830867741 |
|  | 13 | 113776873 | 113777109 | PDFSTATSENSEMBLUCSC | 237 | 8 | 11548 | tss | 0.130587 | 0.4045890 | Not queried | 3.38 | 6.50e-02 | 0.2477578846 |
|  | 10 | 95517382 | 95517562 | PDFSTATSENSEMBLUCSC | 181 | 5 | 5242 | tss | 0.026427 | 0.0031403 | Not queried | 3.37 | 2.22e-01 | 0.3935758299 |
|  | 11 | 43602845 | 43602920 | PDFSTATSENSEMBLUCSC | 76 | 6 | 7254 | tss | 0.021060 | 0.0074671 | Not queried | 3.37 | 9.31e-02 | 0.3104222821 |
|  | 5 | 167719478 | 167719742 | PDFSTATSENSEMBLUCSC | 265 | 4 | 31884 | gene;island | 0.255506 | 0.0245031 | Not queried | 3.37 | 5.71e-02 | 0.2315877671 |
|  | 2 | 218898491 | 218898788 | PDFSTATSENSEMBLUCSC | 298 | 4 | 25078 | island | 0.448037 | 0.9318038 | Not queried | 3.37 | 5.71e-02 | 0.2315877671 |
|  | 11 | 111385338 | 111385496 | PDFSTATSENSEMBLUCSC | 159 | 5 | 7687 | tss | 0.022049 | 0.0137907 | Not queried | 3.37 | 6.90e-01 | 0.7223604052 |
|  | 14 | 61188077 | 61188431 | PDFSTATSENSEMBLUCSC | 355 | 4 | 12104 | gene;island | 0.008757 | 0.3057612 | Not queried | 3.36 | 1.14e-01 | 0.3123457953 |
|  | 2 | 74153086 | 74153317 | PDFSTATSENSEMBLUCSC | 232 | 4 | 23447 | tss;island | 0.799919 | 0.9688409 | Not queried | 3.36 | 3.43e-01 | 0.4672865596 |
|  | 10 | 43846281 | 43846705 | PDFSTATSENSEMBLUCSC | 425 | 5 | 5694 | island | 0.772136 | 0.2703340 | Not queried | 3.36 | 2.22e-01 | 0.3935758299 |
|  | 4 | 104640489 | 104640833 | PDFSTATSENSEMBLUCSC | 345 | 5 | 30078 | gene;island | 0.047137 | 0.3812198 | Not queried | 3.36 | 2.22e-01 | 0.3935758299 |
|  | 19 | 57018848 | 57019069 | PDFSTATSENSEMBLUCSC | 222 | 6 | 21099 | tss;island | 0.096676 | 0.5049068 | Not queried | 3.36 | 2.60e-02 | 0.1830867741 |
|  | 13 | 61989701 | 61990025 | PDFSTATSENSEMBLUCSC | 325 | 8 | 11470 | tss | 0.105035 | 0.5444479 | Not queried | 3.36 | 2.81e-02 | 0.1830867741 |
|  | 6 | 166418799 | 166419138 | PDFSTATSENSEMBLUCSC | 340 | 4 | 36624 | island | 0.102080 | 0.4007129 | Not queried | 3.36 | 2.00e-01 | 0.3826488233 |
|  | 12 | 106974832 | 106975246 | PDFSTATSENSEMBLUCSC | 415 | 5 | 10751 | island | 0.036412 | 0.2969122 | Not queried | 3.36 | 9.52e-02 | 0.3104222821 |
|  | 16 | 49732224 | 49732623 | PDFSTATSENSEMBLUCSC | 400 | 4 | 14745 | gene;island | 0.390415 | 0.0339782 | Not queried | 3.35 | 5.71e-02 | 0.2315877671 |
|  | 5 | 92929319 | 92929679 | PDFSTATSENSEMBLUCSC | 361 | 5 | 31690 | gene;island | 0.008903 | 0.4009911 | Not queried | 3.35 | 5.56e-02 | 0.2315877671 |
|  | 12 | 25102274 | 25102521 | PDFSTATSENSEMBLUCSC | 248 | 5 | 10290 | island | 0.002678 | 0.0136372 | Not queried | 3.35 | 7.94e-03 | 0.1589602024 |
|  | 16 | 4421445 | 4421654 | PDFSTATSENSEMBLUCSC | 210 | 5 | 14638 | gene;tss;island | 0.160927 | 0.8951612 | Not queried | 3.35 | 3.17e-02 | 0.1866936846 |
|  | 1 | 60392140 | 60392392 | PDFSTATSENSEMBLUCSC | 253 | 4 | 560 | gene | 0.019618 | 0.1847350 | Not queried | 3.35 | 2.86e-02 | 0.1830867741 |
|  | 1 | 60392140 | 60392567 | PDFSTATSENSEMBLUCSC | 428 | 6 | 3326 | island | 0.019618 | 0.1797915 | Not queried | 3.35 | 1.52e-02 | 0.1830867741 |
|  | 1 | 153599479 | 153599831 | PDFSTATSENSEMBLUCSC | 353 | 5 | 762 | gene;tss | 0.299869 | 0.8668449 | Not queried | 3.35 | 7.94e-03 | 0.1589602024 |
|  | 8 | 120428418 | 120428692 | PDFSTATSENSEMBLUCSC | 275 | 4 | 40558 | island | 0.013354 | 0.1398503 | Not queried | 3.35 | 1.14e-01 | 0.3123457953 |
|  | 6 | 32410247 | 32410459 | PDFSTATSENSEMBLUCSC | 213 | 6 | 34113 | gene | 0.733020 | 0.9822975 | Not queried | 3.34 | 1.80e-01 | 0.3826488233 |
|  | 11 | 31825605 | 31825969 | PDFSTATSENSEMBLUCSC | 365 | 5 | 6560 | gene;island | 0.018203 | 0.1383361 | Not queried | 3.34 | 1.51e-01 | 0.3618000441 |
|  | 17 | 9694619 | 9695137 | PDFSTATSENSEMBLUCSC | 519 | 6 | 17130 | tss | 0.137268 | 0.5104770 | Not queried | 3.34 | 9.31e-02 | 0.3104222821 |
|  | 6 | 166891617 | 166891959 | PDFSTATSENSEMBLUCSC | 343 | 5 | 34546 | gene | 0.453685 | 0.9082311 | Not queried | 3.34 | 1.51e-01 | 0.3618000441 |
|  | 22 | 50968250 | 50968343 | PDFSTATSENSEMBLUCSC | 94 | 5 | 26931 | gene | 0.015722 | 0.0041809 | Not queried | 3.34 | 5.48e-01 | 0.6179718485 |
|  | 8 | 21771059 | 21771668 | PDFSTATSENSEMBLUCSC | 610 | 7 | 40119 | island | 0.003647 | 0.0185264 | Not queried | 3.33 | 3.79e-02 | 0.2121545571 |
|  | 8 | 9763552 | 9764073 | PDFSTATSENSEMBLUCSC | 522 | 5 | 40066 | island | 0.251502 | 0.0021723 | Not queried | 3.33 | 1.51e-01 | 0.3618000441 |
|  | 10 | 72141375 | 72141625 | PDFSTATSENSEMBLUCSC | 251 | 4 | 4618 | gene;island | 0.499075 | 0.1608556 | Not queried | 3.33 | 3.43e-01 | 0.4672865596 |
|  | 1 | 202172778 | 202172912 | PDFSTATSENSEMBLUCSC | 135 | 4 | 944 | gene | 0.339670 | 0.0278806 | Not queried | 3.33 | 3.43e-01 | 0.4672865596 |
|  | 5 | 178422071 | 178422415 | PDFSTATSENSEMBLUCSC | 345 | 6 | 33477 | island | 0.459334 | 0.0622419 | Not queried | 3.33 | 4.11e-02 | 0.2180389943 |
|  | 7 | 27134109 | 27134369 | PDFSTATSENSEMBLUCSC | 261 | 4 | 36926 | gene;island | 0.518766 | 0.9322212 | Not queried | 3.33 | 3.43e-01 | 0.4672865596 |
|  | 20 | 11871282 | 11871396 | PDFSTATSENSEMBLUCSC | 115 | 5 | 25600 | tss | 0.248713 | 0.0188459 | Not queried | 3.33 | 9.52e-02 | 0.3104222821 |
|  | 20 | 11871282 | 11871743 | PDFSTATSENSEMBLUCSC | 462 | 7 | 26006 | island | 0.248713 | 0.0188459 | Not queried | 3.33 | 9.73e-02 | 0.3123457953 |
|  | 10 | 30721971 | 30722286 | PDFSTATSENSEMBLUCSC | 316 | 4 | 5012 | tss;island | 0.532196 | 0.0362266 | Not queried | 3.32 | 3.43e-01 | 0.4672865596 |
|  | 6 | 25652381 | 25652407 | PDFSTATSENSEMBLUCSC | 27 | 6 | 34674 | tss | 0.495935 | 0.9140510 | Not queried | 3.32 | 2.16e-03 | 0.0934794372 |
|  | 11 | 124747075 | 124747263 | PDFSTATSENSEMBLUCSC | 189 | 4 | 6955 | gene;island | 0.672895 | 0.3462173 | Not queried | 3.32 | 1.14e-01 | 0.3123457953 |
|  | 22 | 31317764 | 31318240 | PDFSTATSENSEMBLUCSC | 477 | 8 | 27070 | tss | 0.003926 | 0.3924808 | Not queried | 3.32 | 1.48e-02 | 0.1830867741 |
|  | 8 | 65291287 | 65291730 | PDFSTATSENSEMBLUCSC | 444 | 9 | 40342 | island | 0.075002 | 0.3601889 | Not queried | 3.32 | 4.00e-02 | 0.2180389943 |
|  | 19 | 36247867 | 36248077 | PDFSTATSENSEMBLUCSC | 211 | 4 | 20713 | tss;island | 0.023944 | 0.3909136 | Not queried | 3.32 | 1.14e-01 | 0.3123457953 |
|  | 10 | 124907409 | 124907708 | PDFSTATSENSEMBLUCSC | 300 | 6 | 6217 | island | 0.271887 | 0.0190546 | Not queried | 3.32 | 1.52e-02 | 0.1830867741 |
|  | 13 | 20138761 | 20139220 | PDFSTATSENSEMBLUCSC | 460 | 5 | 11559 | island | 0.137979 | 0.5330424 | Not queried | 3.31 | 6.90e-01 | 0.7223604052 |
|  | 6 | 26988141 | 26988289 | PDFSTATSENSEMBLUCSC | 149 | 7 | 34710 | tss | 0.027023 | 0.4341912 | Not queried | 3.31 | 1.11e-02 | 0.1821650572 |
|  | 5 | 178422128 | 178422415 | PDFSTATSENSEMBLUCSC | 288 | 5 | 32546 | tss | 0.376135 | 0.0575165 | Not queried | 3.31 | 9.52e-02 | 0.3104222821 |
|  | 6 | 28129313 | 28129498 | PDFSTATSENSEMBLUCSC | 186 | 7 | 34734 | tss | 0.017455 | 0.2015239 | Not queried | 3.31 | 1.11e-02 | 0.1821650572 |
|  | 1 | 220864119 | 220864321 | PDFSTATSENSEMBLUCSC | 203 | 4 | 1019 | gene;island | 0.136764 | 0.0162523 | Not queried | 3.31 | 1.14e-01 | 0.3123457953 |
|  | 11 | 61283865 | 61284134 | PDFSTATSENSEMBLUCSC | 270 | 4 | 6629 | gene;island | 0.009199 | 0.1344169 | Not queried | 3.30 | 1.14e-01 | 0.3123457953 |
|  | 20 | 62795341 | 62795807 | PDFSTATSENSEMBLUCSC | 467 | 5 | 25915 | tss | 0.152386 | 0.6383937 | Not queried | 3.30 | 3.17e-02 | 0.1866936846 |
|  | 17 | 77758370 | 77758850 | PDFSTATSENSEMBLUCSC | 481 | 7 | 16843 | gene;island | 0.682096 | 0.9346099 | Not queried | 3.30 | 5.83e-04 | 0.0548331102 |
|  | 22 | 30901707 | 30901886 | PDFSTATSENSEMBLUCSC | 180 | 4 | 27064 | tss | 0.067262 | 0.5977188 | Not queried | 3.30 | 5.71e-02 | 0.2315877671 |
|  | 5 | 149546331 | 149546471 | PDFSTATSENSEMBLUCSC | 141 | 5 | 33304 | island | 0.678275 | 0.9646446 | Not queried | 3.29 | 2.22e-01 | 0.3935758299 |
|  | 4 | 74719046 | 74719289 | PDFSTATSENSEMBLUCSC | 244 | 4 | 30022 | gene | 0.417780 | 0.0427439 | Not queried | 3.29 | 1.14e-01 | 0.3123457953 |
|  | 4 | 55095282 | 55095529 | PDFSTATSENSEMBLUCSC | 248 | 4 | 30004 | gene | 0.216729 | 0.0019075 | Not queried | 3.29 | 1.14e-01 | 0.3123457953 |
|  | 6 | 10886946 | 10887367 | PDFSTATSENSEMBLUCSC | 422 | 9 | 35442 | island | 0.036495 | 0.0037025 | Not queried | 3.29 | 1.06e-02 | 0.1810746987 |
|  | 7 | 27281216 | 27281964 | PDFSTATSENSEMBLUCSC | 749 | 14 | 37500 | tss;island | 0.032387 | 0.5377164 | Not queried | 3.29 | 9.71e-04 | 0.0805458728 |
|  | 10 | 42863444 | 42863594 | PDFSTATSENSEMBLUCSC | 151 | 5 | 5680 | island | 0.258469 | 0.0270393 | Not queried | 3.29 | 9.52e-02 | 0.3104222821 |
|  | 6 | 6007259 | 6007769 | PDFSTATSENSEMBLUCSC | 511 | 6 | 35407 | island | 0.166015 | 0.0106798 | Not queried | 3.29 | 6.49e-02 | 0.2477578846 |
|  | 19 | 43969650 | 43970034 | PDFSTATSENSEMBLUCSC | 385 | 6 | 22083 | island | 0.837238 | 0.4221695 | Not queried | 3.28 | 6.49e-02 | 0.2477578846 |
|  | 18 | 31020418 | 31021065 | PDFSTATSENSEMBLUCSC | 648 | 8 | 19227 | tss;island | 0.098612 | 0.3882054 | Not queried | 3.28 | 6.50e-02 | 0.2477578846 |
|  | 7 | 43151725 | 43152125 | PDFSTATSENSEMBLUCSC | 401 | 6 | 37555 | tss | 0.033469 | 0.5502725 | Not queried | 3.28 | 3.10e-01 | 0.4672865596 |
|  | 2 | 80530701 | 80530948 | PDFSTATSENSEMBLUCSC | 248 | 4 | 22755 | gene;island | 0.013212 | 0.1801262 | Not queried | 3.28 | 3.43e-01 | 0.4672865596 |
|  | 8 | 6662963 | 6663401 | PDFSTATSENSEMBLUCSC | 439 | 4 | 40052 | island | 0.196506 | 0.4973700 | Not queried | 3.28 | 2.00e-01 | 0.3826488233 |
|  | 22 | 29875578 | 29875991 | PDFSTATSENSEMBLUCSC | 414 | 5 | 27049 | tss;island | 0.033919 | 0.2298651 | Not queried | 3.28 | 5.56e-02 | 0.2315877671 |
|  | 2 | 162283434 | 162284206 | PDFSTATSENSEMBLUCSC | 773 | 8 | 24823 | island | 0.120954 | 0.0208398 | Not queried | 3.28 | 3.79e-02 | 0.2121545571 |
|  | 2 | 55450577 | 55450874 | PDFSTATSENSEMBLUCSC | 298 | 4 | 22686 | gene;island | 0.273282 | 0.0446650 | Not queried | 3.28 | 1.14e-01 | 0.3123457953 |
|  | 3 | 178984841 | 178984973 | PDFSTATSENSEMBLUCSC | 133 | 4 | 28730 | tss | 0.125843 | 0.0300821 | Not queried | 3.27 | 2.86e-02 | 0.1830867741 |
|  | 17 | 48473757 | 48474099 | PDFSTATSENSEMBLUCSC | 343 | 4 | 16686 | gene;island | 0.008887 | 0.0933201 | Not queried | 3.27 | 2.86e-02 | 0.1830867741 |
|  | 13 | 113633379 | 113633600 | PDFSTATSENSEMBLUCSC | 222 | 4 | 11546 | tss | 0.024793 | 0.2773686 | Not queried | 3.27 | 5.71e-02 | 0.2315877671 |
|  | 19 | 40732691 | 40732902 | PDFSTATSENSEMBLUCSC | 212 | 8 | 20784 | tss | 0.249021 | 0.6067431 | Not queried | 3.27 | 4.66e-03 | 0.1498346615 |
|  | 5 | 138861832 | 138861941 | PDFSTATSENSEMBLUCSC | 110 | 4 | 31777 | gene | 0.036097 | 0.3106130 | Not queried | 3.27 | 2.00e-01 | 0.3826488233 |
|  | 2 | 223162666 | 223162800 | PDFSTATSENSEMBLUCSC | 135 | 4 | 23915 | tss | 0.349657 | 0.0130669 | Not queried | 3.27 | 1.14e-01 | 0.3123457953 |
|  | 17 | 45949677 | 45949878 | PDFSTATSENSEMBLUCSC | 202 | 5 | 18464 | island | 0.554480 | 0.8047263 | Not queried | 3.27 | 1.51e-01 | 0.3618000441 |
|  | 10 | 119293800 | 119294332 | PDFSTATSENSEMBLUCSC | 533 | 5 | 4782 | gene;island | 0.158614 | 0.5220398 | Not queried | 3.26 | 5.48e-01 | 0.6179718485 |
|  | 10 | 124906421 | 124906930 | PDFSTATSENSEMBLUCSC | 510 | 6 | 5416 | tss;island | 0.092780 | 0.0125162 | Not queried | 3.26 | 3.10e-01 | 0.4672865596 |
|  | 15 | 66947392 | 66947617 | PDFSTATSENSEMBLUCSC | 226 | 4 | 14245 | island | 0.625199 | 0.2084743 | Not queried | 3.26 | 2.86e-02 | 0.1830867741 |
|  | 6 | 110736772 | 110737053 | PDFSTATSENSEMBLUCSC | 282 | 5 | 35169 | tss | 0.041832 | 0.0527786 | Not queried | 3.26 | 4.21e-01 | 0.5404252807 |
|  | 10 | 72648543 | 72648693 | PDFSTATSENSEMBLUCSC | 151 | 4 | 5140 | tss | 0.004727 | 0.0078073 | Not queried | 3.26 | 2.00e-01 | 0.3826488233 |
|  | 2 | 220299484 | 220299900 | PDFSTATSENSEMBLUCSC | 417 | 9 | 25124 | island | 0.100604 | 0.0410133 | Not queried | 3.26 | 3.40e-01 | 0.4672865596 |
|  | 7 | 1329387 | 1329592 | PDFSTATSENSEMBLUCSC | 206 | 5 | 38097 | island | 0.012470 | 0.4757445 | Not queried | 3.25 | 9.52e-02 | 0.3104222821 |
|  | 8 | 68864769 | 68864964 | PDFSTATSENSEMBLUCSC | 196 | 4 | 39328 | gene;island | 0.012225 | 0.2578596 | Not queried | 3.25 | 4.86e-01 | 0.5640816327 |
|  | 19 | 40732518 | 40732902 | PDFSTATSENSEMBLUCSC | 385 | 9 | 22017 | island | 0.100507 | 0.5155647 | Not queried | 3.25 | 1.42e-02 | 0.1830867741 |
|  | 6 | 30135449 | 30135671 | PDFSTATSENSEMBLUCSC | 223 | 4 | 33737 | gene | 0.056603 | 0.4247008 | Not queried | 3.25 | 2.00e-01 | 0.3826488233 |
|  | 4 | 13538730 | 13539255 | PDFSTATSENSEMBLUCSC | 526 | 7 | 30932 | island | 0.175480 | 0.2001467 | Not queried | 3.25 | 3.18e-01 | 0.4672865596 |
|  | 1 | 82268588 | 82268801 | PDFSTATSENSEMBLUCSC | 214 | 4 | 598 | gene;island | 0.015403 | 0.2946156 | Not queried | 3.25 | 3.43e-01 | 0.4672865596 |
|  | 2 | 220282914 | 220283214 | PDFSTATSENSEMBLUCSC | 301 | 6 | 25123 | island | 0.080477 | 0.4228122 | Not queried | 3.25 | 2.40e-01 | 0.4133265075 |
|  | 11 | 94134446 | 94135029 | PDFSTATSENSEMBLUCSC | 584 | 10 | 8737 | island | 0.349225 | 0.0665760 | Not queried | 3.25 | 6.30e-02 | 0.2477578846 |
|  | 11 | 20690628 | 20690957 | PDFSTATSENSEMBLUCSC | 330 | 7 | 7200 | tss | 0.017589 | 0.2448600 | Not queried | 3.24 | 2.62e-02 | 0.1830867741 |
|  | 6 | 30078954 | 30079662 | PDFSTATSENSEMBLUCSC | 709 | 21 | 33724 | gene | 0.094032 | 0.4893740 | Not queried | 3.24 | 1.48e-03 | 0.0934794372 |
|  | 2 | 103236078 | 103236300 | PDFSTATSENSEMBLUCSC | 223 | 4 | 24574 | island | 0.044063 | 0.2343419 | Not queried | 3.24 | 2.86e-02 | 0.1830867741 |
|  | 3 | 139346734 | 139346955 | PDFSTATSENSEMBLUCSC | 222 | 4 | 27970 | gene | 0.124782 | 0.4512945 | Not queried | 3.23 | 1.14e-01 | 0.3123457953 |
|  | 19 | 56988778 | 56989102 | PDFSTATSENSEMBLUCSC | 325 | 6 | 21098 | tss | 0.067756 | 0.2394204 | Not queried | 3.23 | 6.49e-02 | 0.2477578846 |
|  | 4 | 5570187 | 5570306 | PDFSTATSENSEMBLUCSC | 120 | 4 | 29920 | gene;island | 0.756328 | 0.9633936 | Not queried | 3.23 | 2.86e-02 | 0.1830867741 |
|  | 11 | 93063516 | 93063692 | PDFSTATSENSEMBLUCSC | 177 | 4 | 7619 | tss | 0.010522 | 0.0789882 | Not queried | 3.23 | 2.86e-02 | 0.1830867741 |
|  | 1 | 35220224 | 35220710 | PDFSTATSENSEMBLUCSC | 487 | 6 | 1478 | tss | 0.235004 | 0.4929670 | Not queried | 3.23 | 4.85e-01 | 0.5640816327 |
|  | 14 | 92414151 | 92414425 | PDFSTATSENSEMBLUCSC | 275 | 5 | 12562 | tss;island | 0.086907 | 0.5081642 | Not queried | 3.22 | 7.94e-03 | 0.1589602024 |
|  | 22 | 26875499 | 26875823 | PDFSTATSENSEMBLUCSC | 325 | 6 | 26822 | gene;island | 0.068704 | 0.0128369 | Not queried | 3.22 | 6.49e-02 | 0.2477578846 |
|  | 6 | 26103952 | 26104165 | PDFSTATSENSEMBLUCSC | 214 | 5 | 34685 | tss | 0.000660 | 0.0158735 | Not queried | 3.22 | 3.17e-02 | 0.1866936846 |
|  | 8 | 19460097 | 19460243 | PDFSTATSENSEMBLUCSC | 147 | 5 | 39571 | tss | 0.525373 | 0.9112071 | Not queried | 3.22 | 3.17e-02 | 0.1866936846 |
|  | 1 | 153581494 | 153581793 | PDFSTATSENSEMBLUCSC | 300 | 5 | 761 | gene | 0.428992 | 0.8743217 | Not queried | 3.22 | 5.56e-02 | 0.2315877671 |
|  | 5 | 140514711 | 140515207 | PDFSTATSENSEMBLUCSC | 497 | 6 | 33212 | island | 0.040026 | 0.3680801 | Not queried | 3.22 | 1.52e-02 | 0.1830867741 |
|  | 17 | 185102 | 185263 | PDFSTATSENSEMBLUCSC | 162 | 4 | 16292 | gene;island | 0.282092 | 0.7210152 | Not queried | 3.22 | 1.14e-01 | 0.3123457953 |
|  | 11 | 17229487 | 17229731 | PDFSTATSENSEMBLUCSC | 245 | 5 | 8095 | island | 0.000279 | 0.0038103 | Not queried | 3.22 | 1.51e-01 | 0.3618000441 |
|  | 7 | 145813915 | 145814306 | PDFSTATSENSEMBLUCSC | 392 | 5 | 37257 | gene;island | 0.023150 | 0.1366396 | Not queried | 3.22 | 7.94e-03 | 0.1589602024 |
|  | 6 | 28226885 | 28227482 | PDFSTATSENSEMBLUCSC | 598 | 11 | 35585 | island | 0.760262 | 0.1261724 | Not queried | 3.22 | 1.01e-01 | 0.3123457953 |
|  | 13 | 47472050 | 47472429 | PDFSTATSENSEMBLUCSC | 380 | 12 | 11429 | tss | 0.392696 | 0.7830259 | Not queried | 3.21 | 2.05e-02 | 0.1830867741 |
|  | 6 | 56111812 | 56112696 | PDFSTATSENSEMBLUCSC | 885 | 13 | 36219 | island | 0.020025 | 0.1330502 | Not queried | 3.21 | 5.01e-02 | 0.2315877671 |
|  | 8 | 143580770 | 143581070 | PDFSTATSENSEMBLUCSC | 301 | 4 | 39463 | gene;island | 0.912833 | 0.4944840 | Not queried | 3.21 | 2.00e-01 | 0.3826488233 |
|  | 4 | 81187601 | 81187906 | PDFSTATSENSEMBLUCSC | 306 | 5 | 31136 | island | 0.013711 | 0.1160797 | Not queried | 3.21 | 1.59e-02 | 0.1830867741 |
|  | 17 | 79420145 | 79420436 | PDFSTATSENSEMBLUCSC | 292 | 5 | 16911 | gene;island | 0.059488 | 0.3692773 | Not queried | 3.21 | 1.51e-01 | 0.3618000441 |
|  | 6 | 110798012 | 110798323 | PDFSTATSENSEMBLUCSC | 312 | 5 | 35170 | tss;island | 0.525172 | 0.6558192 | Not queried | 3.21 | 5.48e-01 | 0.6179718485 |
|  | 8 | 93114150 | 93114437 | PDFSTATSENSEMBLUCSC | 288 | 4 | 40449 | island | 0.071322 | 0.3781465 | Not queried | 3.21 | 2.00e-01 | 0.3826488233 |
|  | 4 | 55093834 | 55094011 | PDFSTATSENSEMBLUCSC | 178 | 4 | 30400 | tss | 0.204852 | 0.0073430 | Not queried | 3.21 | 2.00e-01 | 0.3826488233 |
|  | 7 | 149389669 | 149389941 | PDFSTATSENSEMBLUCSC | 273 | 4 | 38999 | island | 0.629756 | 0.9147959 | Not queried | 3.21 | 5.71e-02 | 0.2315877671 |
|  | 13 | 113104883 | 113105341 | PDFSTATSENSEMBLUCSC | 459 | 5 | 11920 | island | 0.336563 | 0.8075437 | Not queried | 3.21 | 1.51e-01 | 0.3618000441 |
|  | 6 | 99290284 | 99290737 | PDFSTATSENSEMBLUCSC | 454 | 5 | 36345 | island | 0.028818 | 0.2134832 | Not queried | 3.20 | 1.59e-02 | 0.1830867741 |
|  | 19 | 496239 | 496528 | PDFSTATSENSEMBLUCSC | 290 | 6 | 21163 | island | 0.023423 | 0.2507969 | Not queried | 3.20 | 6.49e-02 | 0.2477578846 |
|  | 22 | 45148218 | 45148385 | PDFSTATSENSEMBLUCSC | 168 | 9 | 27215 | tss | 0.011300 | 0.1620448 | Not queried | 3.20 | 4.94e-04 | 0.0548331102 |
|  | 5 | 72746710 | 72746990 | PDFSTATSENSEMBLUCSC | 281 | 4 | 32874 | island | 0.211885 | 0.0129545 | Not queried | 3.20 | 2.00e-01 | 0.3826488233 |
|  | 15 | 74421739 | 74421935 | PDFSTATSENSEMBLUCSC | 197 | 7 | 13416 | gene | 0.049488 | 0.0062248 | Not queried | 3.20 | 6.99e-03 | 0.1589602024 |
|  | 7 | 99766376 | 99766665 | PDFSTATSENSEMBLUCSC | 290 | 5 | 37774 | tss | 0.154572 | 0.6764655 | Not queried | 3.20 | 3.10e-01 | 0.4672865596 |
|  | 22 | 42095154 | 42095443 | PDFSTATSENSEMBLUCSC | 290 | 6 | 27179 | tss | 0.457559 | 0.0896919 | Not queried | 3.20 | 6.49e-02 | 0.2477578846 |
|  | 18 | 12287173 | 12287561 | PDFSTATSENSEMBLUCSC | 389 | 4 | 19402 | island | 0.150209 | 0.4381731 | Not queried | 3.20 | 1.14e-01 | 0.3123457953 |
|  | 7 | 123563992 | 123564379 | PDFSTATSENSEMBLUCSC | 388 | 4 | 37872 | tss | 0.032230 | 0.3956091 | Not queried | 3.20 | 5.71e-02 | 0.2315877671 |
|  | 20 | 23030343 | 23030533 | PDFSTATSENSEMBLUCSC | 191 | 5 | 25636 | tss | 0.007082 | 0.0172442 | Not queried | 3.20 | 2.22e-01 | 0.3935758299 |
|  | 7 | 16460965 | 16461244 | PDFSTATSENSEMBLUCSC | 280 | 5 | 37451 | tss | 0.235574 | 0.0266022 | Not queried | 3.20 | 1.51e-01 | 0.3618000441 |
|  | 17 | 41739130 | 41739326 | PDFSTATSENSEMBLUCSC | 197 | 4 | 17392 | tss | 0.172413 | 0.4886118 | Not queried | 3.19 | 3.43e-01 | 0.4672865596 |
|  | 14 | 90168304 | 90168492 | PDFSTATSENSEMBLUCSC | 189 | 4 | 13067 | island | 0.528003 | 0.1400913 | Not queried | 3.19 | 3.43e-01 | 0.4672865596 |
|  | 2 | 97171137 | 97171449 | PDFSTATSENSEMBLUCSC | 313 | 5 | 22781 | gene;island | 0.012691 | 0.2584532 | Not queried | 3.19 | 4.21e-01 | 0.5404252807 |
|  | 8 | 77585222 | 77585448 | PDFSTATSENSEMBLUCSC | 227 | 4 | 39340 | gene | 0.297623 | 0.0376278 | Not queried | 3.19 | 2.00e-01 | 0.3826488233 |
|  | 19 | 12946231 | 12946465 | PDFSTATSENSEMBLUCSC | 235 | 6 | 20515 | tss | 0.143776 | 0.0392447 | Not queried | 3.19 | 3.10e-01 | 0.4672865596 |
|  | 15 | 58723657 | 58724041 | PDFSTATSENSEMBLUCSC | 385 | 4 | 13698 | tss | 0.446383 | 0.8760834 | Not queried | 3.18 | 3.43e-01 | 0.4672865596 |
|  | 2 | 133426129 | 133426418 | PDFSTATSENSEMBLUCSC | 290 | 4 | 22878 | gene;island | 0.061793 | 0.3077727 | Not queried | 3.18 | 2.86e-02 | 0.1830867741 |
|  | 12 | 104697193 | 104697631 | PDFSTATSENSEMBLUCSC | 439 | 12 | 9273 | gene;island | 0.074641 | 0.0233017 | Not queried | 3.18 | 2.42e-02 | 0.1830867741 |
|  | 10 | 135039948 | 135040250 | PDFSTATSENSEMBLUCSC | 303 | 5 | 6345 | island | 0.070367 | 0.4247312 | Not queried | 3.18 | 2.22e-01 | 0.3935758299 |
|  | 7 | 1783946 | 1784169 | PDFSTATSENSEMBLUCSC | 224 | 4 | 36766 | gene;island | 0.445826 | 0.8944046 | Not queried | 3.18 | 1.14e-01 | 0.3123457953 |
|  | 6 | 29079939 | 29080437 | PDFSTATSENSEMBLUCSC | 499 | 5 | 33650 | gene | 0.066728 | 0.4689336 | Not queried | 3.18 | 9.52e-02 | 0.3104222821 |
|  | 17 | 58216297 | 58216651 | PDFSTATSENSEMBLUCSC | 355 | 4 | 18618 | island | 0.443435 | 0.0811534 | Not queried | 3.17 | 3.43e-01 | 0.4672865596 |
|  | 15 | 89438795 | 89438964 | PDFSTATSENSEMBLUCSC | 170 | 4 | 13882 | tss | 0.021979 | 0.2056080 | Not queried | 3.17 | 4.86e-01 | 0.5640816327 |
|  | 22 | 38218945 | 38219336 | PDFSTATSENSEMBLUCSC | 392 | 4 | 27126 | tss | 0.157393 | 0.5301157 | Not queried | 3.17 | 3.43e-01 | 0.4672865596 |
|  | 6 | 29570507 | 29570781 | PDFSTATSENSEMBLUCSC | 275 | 5 | 33663 | gene | 0.251345 | 0.4621757 | Not queried | 3.17 | 1.51e-01 | 0.3618000441 |
|  | 5 | 172655841 | 172656166 | PDFSTATSENSEMBLUCSC | 326 | 6 | 33405 | island | 0.136688 | 0.0172213 | Not queried | 3.17 | 2.40e-01 | 0.4133265075 |
|  | 2 | 63274857 | 63275178 | PDFSTATSENSEMBLUCSC | 322 | 4 | 23384 | tss | 0.181711 | 0.0354269 | Not queried | 3.17 | 2.00e-01 | 0.3826488233 |
|  | 5 | 149535646 | 149535765 | PDFSTATSENSEMBLUCSC | 120 | 4 | 32425 | tss | 0.022575 | 0.2264305 | Not queried | 3.17 | 2.00e-01 | 0.3826488233 |
|  | 1 | 207277018 | 207277581 | PDFSTATSENSEMBLUCSC | 564 | 8 | 2219 | tss | 0.563654 | 0.9144842 | Not queried | 3.17 | 1.61e-01 | 0.3814043454 |
|  | 12 | 113909932 | 113910034 | PDFSTATSENSEMBLUCSC | 103 | 6 | 10030 | tss;island | 0.009513 | 0.1641742 | Not queried | 3.17 | 1.80e-01 | 0.3826488233 |
|  | 1 | 32281665 | 32281812 | PDFSTATSENSEMBLUCSC | 148 | 4 | 1451 | tss | 0.446511 | 0.0678296 | Not queried | 3.17 | 5.71e-02 | 0.2315877671 |
|  | 5 | 162930177 | 162930325 | PDFSTATSENSEMBLUCSC | 149 | 5 | 33365 | island | 0.017820 | 0.0056229 | Not queried | 3.16 | 2.22e-01 | 0.3935758299 |
|  | 11 | 62621178 | 62621406 | PDFSTATSENSEMBLUCSC | 229 | 4 | 6649 | gene;tss;island | 0.013512 | 0.0073728 | Not queried | 3.16 | 6.86e-01 | 0.7223604052 |
|  | 7 | 27183369 | 27184737 | PDFSTATSENSEMBLUCSC | 1369 | 41 | 37492 | tss | 0.448084 | 0.8855557 | HOXA-AS3; HOXA3; HOXA5; HOXA6 (margin: HOXA-AS2; HOXA-AS3; HOXA3; HOXA5; HOXA6; HOXA7) | 3.16 | 1.30e-09 | 0.0000017973 |
|  | 2 | 220174201 | 220174251 | PDFSTATSENSEMBLUCSC | 51 | 4 | 23904 | tss;island | 0.061714 | 0.2258709 | Not queried | 3.16 | 3.43e-01 | 0.4672865596 |
|  | 17 | 1202015 | 1202252 | PDFSTATSENSEMBLUCSC | 238 | 4 | 16312 | gene | 0.457696 | 0.0888748 | Not queried | 3.16 | 3.43e-01 | 0.4672865596 |
|  | 15 | 69744390 | 69745085 | PDFSTATSENSEMBLUCSC | 696 | 12 | 13764 | tss | 0.016066 | 0.0044421 | Not queried | 3.16 | 1.11e-03 | 0.0833132385 |
|  | 7 | 29186153 | 29186347 | PDFSTATSENSEMBLUCSC | 195 | 7 | 37510 | tss | 0.033840 | 0.0193550 | Not queried | 3.16 | 2.59e-01 | 0.4381244265 |
|  | 10 | 695844 | 695900 | PDFSTATSENSEMBLUCSC | 57 | 5 | 4910 | tss | 0.487210 | 0.8927664 | Not queried | 3.15 | 9.52e-02 | 0.3104222821 |
|  | 22 | 45809244 | 45810043 | PDFSTATSENSEMBLUCSC | 800 | 16 | 27223 | tss;island | 0.256033 | 0.0246571 | Not queried | 3.15 | 3.92e-03 | 0.1459191215 |
|  | 13 | 100626995 | 100627366 | PDFSTATSENSEMBLUCSC | 372 | 4 | 11846 | island | 0.038939 | 0.1531425 | Not queried | 3.15 | 2.86e-02 | 0.1830867741 |
|  | 3 | 181441345 | 181442137 | PDFSTATSENSEMBLUCSC | 793 | 9 | 28044 | gene;island | 0.073266 | 0.5428204 | Not queried | 3.15 | 2.44e-02 | 0.1830867741 |
|  | 1 | 98510865 | 98511258 | PDFSTATSENSEMBLUCSC | 394 | 5 | 3513 | island | 0.013544 | 0.1609140 | Not queried | 3.15 | 1.51e-01 | 0.3618000441 |
|  | 5 | 140800398 | 140800983 | PDFSTATSENSEMBLUCSC | 586 | 9 | 31834 | gene;island | 0.177756 | 0.0230619 | Not queried | 3.15 | 1.88e-02 | 0.1830867741 |
|  | 16 | 87447700 | 87448114 | PDFSTATSENSEMBLUCSC | 415 | 4 | 14890 | gene;island | 0.533056 | 0.9457984 | Not queried | 3.14 | 2.00e-01 | 0.3826488233 |
|  | 19 | 58446745 | 58446988 | PDFSTATSENSEMBLUCSC | 244 | 8 | 21129 | tss | 0.130236 | 0.0201309 | Not queried | 3.14 | 2.07e-02 | 0.1830867741 |
|  | 6 | 32977400 | 32977708 | PDFSTATSENSEMBLUCSC | 309 | 4 | 34886 | tss | 0.496310 | 0.3063218 | Not queried | 3.14 | 4.86e-01 | 0.5640816327 |
|  | 2 | 97505275 | 97505787 | PDFSTATSENSEMBLUCSC | 513 | 5 | 22787 | gene;island | 0.666745 | 0.9779356 | Not queried | 3.14 | 1.51e-01 | 0.3618000441 |
|  | 11 | 118842126 | 118842413 | PDFSTATSENSEMBLUCSC | 288 | 7 | 7742 | tss | 0.955158 | 0.5619925 | Not queried | 3.14 | 1.65e-01 | 0.3826488233 |
|  | 17 | 80084554 | 80084751 | PDFSTATSENSEMBLUCSC | 198 | 4 | 16939 | gene;island | 0.519052 | 0.9751829 | Not queried | 3.14 | 2.00e-01 | 0.3826488233 |
|  | 17 | 73893198 | 73893209 | PDFSTATSENSEMBLUCSC | 12 | 4 | 17665 | tss | 0.012444 | 0.1373937 | Not queried | 3.14 | 5.71e-02 | 0.2315877671 |
|  | 20 | 30073209 | 30073576 | PDFSTATSENSEMBLUCSC | 368 | 6 | 25660 | tss;island | 0.463661 | 0.0683297 | Not queried | 3.13 | 1.52e-02 | 0.1830867741 |
|  | 11 | 94501303 | 94501467 | PDFSTATSENSEMBLUCSC | 165 | 7 | 7632 | tss | 0.034937 | 0.0058885 | Not queried | 3.13 | 7.28e-02 | 0.2713481487 |
|  | 11 | 2176877 | 2177018 | PDFSTATSENSEMBLUCSC | 142 | 4 | 6451 | gene;island | 0.350977 | 0.7937380 | Not queried | 3.13 | 2.00e-01 | 0.3826488233 |
|  | 11 | 119227592 | 119227876 | PDFSTATSENSEMBLUCSC | 285 | 4 | 6939 | gene;island | 0.016618 | 0.0716363 | Not queried | 3.13 | 3.43e-01 | 0.4672865596 |
|  | 2 | 109647956 | 109648208 | PDFSTATSENSEMBLUCSC | 253 | 4 | 24604 | island | 0.015818 | 0.2917285 | Not queried | 3.13 | 3.43e-01 | 0.4672865596 |
|  | 1 | 42384310 | 42384474 | PDFSTATSENSEMBLUCSC | 165 | 4 | 463 | gene | 0.781784 | 0.4284415 | Not queried | 3.13 | 5.71e-02 | 0.2315877671 |
|  | 5 | 181726 | 182028 | PDFSTATSENSEMBLUCSC | 303 | 4 | 31496 | gene;island | 0.386328 | 0.6009033 | Not queried | 3.13 | 4.86e-01 | 0.5640816327 |
|  | 5 | 175224563 | 175224721 | PDFSTATSENSEMBLUCSC | 159 | 4 | 31908 | gene;island | 0.017810 | 0.0899433 | Not queried | 3.13 | 1.14e-01 | 0.3123457953 |
|  | 5 | 41509849 | 41510116 | PDFSTATSENSEMBLUCSC | 268 | 5 | 31599 | gene;island | 0.044750 | 0.5973028 | Not queried | 3.13 | 1.51e-01 | 0.3618000441 |
|  | 2 | 176986261 | 176986659 | PDFSTATSENSEMBLUCSC | 399 | 4 | 23736 | tss;island | 0.572285 | 0.2527560 | Not queried | 3.12 | 3.43e-01 | 0.4672865596 |
|  | 12 | 106533667 | 106533903 | PDFSTATSENSEMBLUCSC | 237 | 6 | 10747 | island | 0.270738 | 0.0476903 | Not queried | 3.12 | 1.80e-01 | 0.3826488233 |
|  | 17 | 59534637 | 59534998 | PDFSTATSENSEMBLUCSC | 362 | 5 | 16720 | gene;island | 0.043397 | 0.0056407 | Not queried | 3.12 | 3.17e-02 | 0.1866936846 |
|  | 17 | 46871708 | 46871905 | PDFSTATSENSEMBLUCSC | 198 | 5 | 17473 | tss | 0.451132 | 0.8696867 | Not queried | 3.12 | 9.52e-02 | 0.3104222821 |
|  | 19 | 50553682 | 50553907 | PDFSTATSENSEMBLUCSC | 226 | 5 | 20971 | tss | 0.013263 | 0.1089239 | Not queried | 3.12 | 9.52e-02 | 0.3104222821 |
|  | 3 | 42705828 | 42706106 | PDFSTATSENSEMBLUCSC | 279 | 4 | 27735 | gene | 0.346372 | 0.0373939 | Not queried | 3.12 | 3.43e-01 | 0.4672865596 |
|  | 2 | 43398079 | 43398339 | PDFSTATSENSEMBLUCSC | 261 | 6 | 24248 | island | 0.497167 | 0.9489853 | Not queried | 3.12 | 1.32e-01 | 0.3397986247 |
|  | 11 | 16632311 | 16632724 | PDFSTATSENSEMBLUCSC | 414 | 4 | 8091 | island | 0.214565 | 0.3810090 | Not queried | 3.11 | 3.43e-01 | 0.4672865596 |
|  | 3 | 183146663 | 183146882 | PDFSTATSENSEMBLUCSC | 220 | 4 | 28746 | tss;island | 0.007313 | 0.0801416 | Not queried | 3.11 | 5.71e-02 | 0.2315877671 |
|  | 19 | 51231557 | 51232007 | PDFSTATSENSEMBLUCSC | 451 | 5 | 22322 | island | 0.364422 | 0.6508143 | Not queried | 3.11 | 3.10e-01 | 0.4672865596 |
|  | 1 | 6639570 | 6639762 | PDFSTATSENSEMBLUCSC | 193 | 4 | 179 | gene | 0.022654 | 0.2939859 | Not queried | 3.11 | 3.43e-01 | 0.4672865596 |
|  | 13 | 92051400 | 92051786 | PDFSTATSENSEMBLUCSC | 387 | 4 | 11189 | gene;island | 0.082483 | 0.5059692 | Not queried | 3.11 | 2.00e-01 | 0.3826488233 |
|  | 5 | 145713607 | 145713882 | PDFSTATSENSEMBLUCSC | 276 | 6 | 33279 | island | 0.015629 | 0.3816110 | Not queried | 3.11 | 1.32e-01 | 0.3397986247 |
|  | 2 | 232765174 | 232765416 | PDFSTATSENSEMBLUCSC | 243 | 4 | 25189 | island | 0.707781 | 0.0735826 | Not queried | 3.11 | 4.86e-01 | 0.5640816327 |
|  | 7 | 1022471 | 1022806 | PDFSTATSENSEMBLUCSC | 336 | 6 | 37392 | tss | 0.351482 | 0.8417946 | Not queried | 3.11 | 4.11e-02 | 0.2180389943 |
|  | 1 | 15256054 | 15256222 | PDFSTATSENSEMBLUCSC | 169 | 5 | 240 | gene;tss | 0.050273 | 0.4664632 | Not queried | 3.10 | 1.51e-01 | 0.3618000441 |
|  | 2 | 105853494 | 105853796 | PDFSTATSENSEMBLUCSC | 303 | 4 | 24583 | island | 0.881636 | 0.5757354 | Not queried | 3.10 | 5.71e-02 | 0.2315877671 |
|  | 6 | 29574700 | 29575145 | PDFSTATSENSEMBLUCSC | 446 | 7 | 33667 | gene | 0.168012 | 0.3890642 | Not queried | 3.10 | 1.75e-02 | 0.1830867741 |
|  | 8 | 110704026 | 110704289 | PDFSTATSENSEMBLUCSC | 264 | 5 | 39874 | tss | 0.007865 | 0.0410854 | Not queried | 3.10 | 1.51e-01 | 0.3618000441 |
|  | 17 | 79881468 | 79881543 | PDFSTATSENSEMBLUCSC | 76 | 5 | 16929 | gene;tss;island | 0.232049 | 0.0342844 | Not queried | 3.10 | 7.94e-03 | 0.1589602024 |
|  | 2 | 71503484 | 71503883 | PDFSTATSENSEMBLUCSC | 400 | 7 | 24396 | island | 0.079394 | 0.0065999 | Not queried | 3.09 | 6.99e-03 | 0.1589602024 |
|  | 8 | 145625605 | 145625814 | PDFSTATSENSEMBLUCSC | 210 | 4 | 39981 | tss | 0.853023 | 0.9818844 | Not queried | 3.09 | 2.86e-02 | 0.1830867741 |
|  | 17 | 76037035 | 76037562 | PDFSTATSENSEMBLUCSC | 528 | 6 | 16812 | gene;island | 0.659963 | 0.9328694 | Not queried | 3.09 | 2.16e-03 | 0.0934794372 |
|  | 2 | 242808427 | 242808907 | PDFSTATSENSEMBLUCSC | 481 | 5 | 25312 | island | 0.138288 | 0.5336316 | Not queried | 3.09 | 2.22e-01 | 0.3935758299 |
|  | 7 | 93519220 | 93519621 | PDFSTATSENSEMBLUCSC | 402 | 4 | 37090 | gene;island | 0.017189 | 0.1457377 | Not queried | 3.09 | 2.00e-01 | 0.3826488233 |
|  | 1 | 855425 | 855649 | PDFSTATSENSEMBLUCSC | 225 | 4 | 1133 | tss;island | 0.332196 | 0.7084453 | Not queried | 3.09 | 5.71e-02 | 0.2315877671 |
|  | 1 | 40782713 | 40783264 | PDFSTATSENSEMBLUCSC | 552 | 10 | 3120 | island | 0.020692 | 0.1950846 | Not queried | 3.09 | 1.85e-02 | 0.1830867741 |
|  | 1 | 180201002 | 180201319 | PDFSTATSENSEMBLUCSC | 318 | 4 | 905 | gene;island | 0.247612 | 0.0161127 | Not queried | 3.09 | 3.43e-01 | 0.4672865596 |
|  | 3 | 44626492 | 44626860 | PDFSTATSENSEMBLUCSC | 369 | 4 | 27740 | gene | 0.019088 | 0.1860929 | Not queried | 3.09 | 1.14e-01 | 0.3123457953 |
|  | 1 | 7111184 | 7111367 | PDFSTATSENSEMBLUCSC | 184 | 4 | 185 | gene | 0.466286 | 0.6631403 | Not queried | 3.09 | 2.00e-01 | 0.3826488233 |
|  | 4 | 41869086 | 41869611 | PDFSTATSENSEMBLUCSC | 526 | 7 | 31006 | island | 0.067774 | 0.3364103 | Not queried | 3.09 | 1.75e-02 | 0.1830867741 |
|  | 16 | 56677634 | 56677855 | PDFSTATSENSEMBLUCSC | 222 | 4 | 14763 | gene | 0.080407 | 0.4252559 | Not queried | 3.08 | 6.86e-01 | 0.7223604052 |
|  | 6 | 87647370 | 87647644 | PDFSTATSENSEMBLUCSC | 275 | 5 | 34408 | gene;island | 0.057945 | 0.3851937 | Not queried | 3.08 | 2.22e-01 | 0.3935758299 |
|  | 12 | 81110706 | 81111012 | PDFSTATSENSEMBLUCSC | 307 | 5 | 10648 | island | 0.096599 | 0.5430759 | Not queried | 3.08 | 9.52e-02 | 0.3104222821 |
|  | 14 | 77279317 | 77279508 | PDFSTATSENSEMBLUCSC | 192 | 4 | 12525 | tss | 0.019999 | 0.1231417 | Not queried | 3.08 | 3.43e-01 | 0.4672865596 |
|  | 19 | 16939845 | 16940140 | PDFSTATSENSEMBLUCSC | 296 | 4 | 20576 | tss | 0.003853 | 0.0096927 | Not queried | 3.08 | 4.86e-01 | 0.5640816327 |
|  | 22 | 26875552 | 26875823 | PDFSTATSENSEMBLUCSC | 272 | 5 | 27033 | tss | 0.083770 | 0.0218832 | Not queried | 3.08 | 2.22e-01 | 0.3935758299 |
|  | 1 | 166916762 | 166917267 | PDFSTATSENSEMBLUCSC | 506 | 5 | 872 | gene;island | 0.136823 | 0.0219466 | Not queried | 3.08 | 9.52e-02 | 0.3104222821 |
|  | 4 | 13530923 | 13531213 | PDFSTATSENSEMBLUCSC | 291 | 6 | 30931 | island | 0.116466 | 0.5206456 | Not queried | 3.08 | 2.60e-02 | 0.1830867741 |
|  | 19 | 50789808 | 50789973 | PDFSTATSENSEMBLUCSC | 166 | 4 | 20172 | gene;island | 0.227766 | 0.7366940 | Not queried | 3.08 | 2.86e-02 | 0.1830867741 |
|  | 16 | 89163343 | 89163800 | PDFSTATSENSEMBLUCSC | 458 | 5 | 14927 | gene;island | 0.597626 | 0.9784122 | Not queried | 3.07 | 5.56e-02 | 0.2315877671 |
|  | 10 | 530635 | 531584 | PDFSTATSENSEMBLUCSC | 950 | 12 | 4441 | gene;island | 0.234958 | 0.5569539 | Not queried | 3.07 | 2.32e-03 | 0.0974014615 |
|  | 7 | 23636744 | 23636910 | PDFSTATSENSEMBLUCSC | 167 | 5 | 37474 | tss | 0.017983 | 0.0185138 | Not queried | 3.07 | 5.48e-01 | 0.6179718485 |
|  | 1 | 227505845 | 227506021 | PDFSTATSENSEMBLUCSC | 177 | 6 | 2292 | tss;island | 0.020768 | 0.1657895 | Not queried | 3.07 | 2.60e-02 | 0.1830867741 |
|  | 17 | 20687681 | 20687912 | PDFSTATSENSEMBLUCSC | 232 | 4 | 18110 | island | 0.609177 | 0.3486135 | Not queried | 3.07 | 4.86e-01 | 0.5640816327 |
|  | 6 | 168435914 | 168436099 | PDFSTATSENSEMBLUCSC | 186 | 4 | 34558 | gene;island | 0.202853 | 0.5737327 | Not queried | 3.07 | 3.43e-01 | 0.4672865596 |
|  | 16 | 86546628 | 86546979 | PDFSTATSENSEMBLUCSC | 352 | 5 | 14884 | gene;island | 0.371148 | 0.0472291 | Not queried | 3.07 | 2.22e-01 | 0.3935758299 |
|  | 17 | 33760230 | 33760527 | PDFSTATSENSEMBLUCSC | 298 | 5 | 17270 | tss | 0.264130 | 0.0293236 | Not queried | 3.06 | 1.51e-01 | 0.3618000441 |
|  | 10 | 88149210 | 88149632 | PDFSTATSENSEMBLUCSC | 423 | 4 | 5903 | island | 0.028078 | 0.3121494 | Not queried | 3.06 | 1.14e-01 | 0.3123457953 |
|  | 19 | 3398706 | 3399015 | PDFSTATSENSEMBLUCSC | 310 | 5 | 19715 | gene;island | 0.941854 | 0.7375422 | Not queried | 3.06 | 7.94e-03 | 0.1589602024 |
|  | 2 | 235860416 | 235860554 | PDFSTATSENSEMBLUCSC | 139 | 5 | 23963 | tss;island | 0.003347 | 0.0218814 | Not queried | 3.06 | 3.10e-01 | 0.4672865596 |
|  | 17 | 64188403 | 64188644 | PDFSTATSENSEMBLUCSC | 242 | 4 | 17595 | tss;island | 0.021051 | 0.0071959 | Not queried | 3.06 | 4.86e-01 | 0.5640816327 |
|  | 2 | 3642710 | 3642967 | PDFSTATSENSEMBLUCSC | 258 | 5 | 22553 | gene | 0.969974 | 0.7826497 | Not queried | 3.06 | 7.94e-03 | 0.1589602024 |
|  | 1 | 1104257 | 1104526 | PDFSTATSENSEMBLUCSC | 270 | 7 | 2410 | island | 0.129184 | 0.5517690 | Not queried | 3.05 | 2.59e-01 | 0.4381244265 |
|  | 20 | 51589616 | 51589985 | PDFSTATSENSEMBLUCSC | 370 | 4 | 25457 | gene;island | 0.025588 | 0.0997219 | Not queried | 3.05 | 1.14e-01 | 0.3123457953 |
|  | 12 | 99548329 | 99548550 | PDFSTATSENSEMBLUCSC | 222 | 5 | 9262 | gene | 0.612051 | 0.8946646 | Not queried | 3.05 | 7.94e-03 | 0.1589602024 |
|  | 2 | 198650603 | 198651076 | PDFSTATSENSEMBLUCSC | 474 | 6 | 23802 | tss;island | 0.014038 | 0.0302761 | Not queried | 3.05 | 5.89e-01 | 0.6570484697 |
|  | 2 | 219861012 | 219861150 | PDFSTATSENSEMBLUCSC | 139 | 5 | 25101 | island | 0.012309 | 0.0036412 | Not queried | 3.04 | 4.21e-01 | 0.5404252807 |
|  | 1 | 65991461 | 65991765 | PDFSTATSENSEMBLUCSC | 305 | 4 | 571 | gene;island | 0.055427 | 0.3235456 | Not queried | 3.04 | 2.00e-01 | 0.3826488233 |
|  | 17 | 75789279 | 75789529 | PDFSTATSENSEMBLUCSC | 251 | 5 | 18812 | island | 0.506372 | 0.7702039 | Not queried | 3.04 | 2.22e-01 | 0.3935758299 |
|  | 6 | 149772707 | 149772892 | PDFSTATSENSEMBLUCSC | 186 | 4 | 34505 | gene;island | 0.626625 | 0.9490709 | Not queried | 3.04 | 5.71e-02 | 0.2315877671 |
|  | 19 | 53832311 | 53832670 | PDFSTATSENSEMBLUCSC | 360 | 5 | 22374 | island | 0.558452 | 0.1341545 | Not queried | 3.04 | 9.52e-02 | 0.3104222821 |
|  | 14 | 105116395 | 105116681 | PDFSTATSENSEMBLUCSC | 287 | 4 | 13210 | island | 0.008708 | 0.0560845 | Not queried | 3.04 | 3.43e-01 | 0.4672865596 |
|  | 10 | 13043878 | 13043986 | PDFSTATSENSEMBLUCSC | 109 | 6 | 4953 | tss;island | 0.014056 | 0.1157171 | Not queried | 3.04 | 2.60e-02 | 0.1830867741 |
|  | 2 | 172950067 | 172950171 | PDFSTATSENSEMBLUCSC | 105 | 6 | 23717 | tss | 0.007012 | 0.0017025 | Not queried | 3.04 | 2.16e-03 | 0.0934794372 |
|  | 17 | 79495268 | 79495348 | PDFSTATSENSEMBLUCSC | 81 | 5 | 17740 | tss | 0.881945 | 0.5287471 | Not queried | 3.04 | 9.52e-02 | 0.3104222821 |
|  | 17 | 79495268 | 79495664 | PDFSTATSENSEMBLUCSC | 397 | 7 | 18927 | island | 0.881945 | 0.5287471 | Not queried | 3.04 | 9.73e-02 | 0.3123457953 |
|  | 11 | 70672740 | 70672986 | PDFSTATSENSEMBLUCSC | 247 | 7 | 6797 | gene;island | 0.030969 | 0.3579024 | Not queried | 3.04 | 1.28e-01 | 0.3397986247 |
|  | 2 | 182850059 | 182850441 | PDFSTATSENSEMBLUCSC | 383 | 4 | 23764 | tss | 0.031851 | 0.6128378 | Not queried | 3.03 | 6.86e-01 | 0.7223604052 |
|  | 1 | 205181033 | 205181261 | PDFSTATSENSEMBLUCSC | 229 | 4 | 2202 | tss;island | 0.045024 | 0.0068030 | Not queried | 3.03 | 3.43e-01 | 0.4672865596 |
|  | 17 | 8702369 | 8702896 | PDFSTATSENSEMBLUCSC | 528 | 9 | 18008 | island | 0.675907 | 0.9567168 | Not queried | 3.03 | 7.70e-02 | 0.2825346647 |
|  | 3 | 191048308 | 191048439 | PDFSTATSENSEMBLUCSC | 132 | 5 | 28084 | gene;island | 0.001807 | 0.0055487 | Not queried | 3.03 | 1.51e-01 | 0.3618000441 |
|  | 10 | 116164598 | 116164955 | PDFSTATSENSEMBLUCSC | 358 | 6 | 5377 | tss;island | 0.531697 | 0.2301978 | Not queried | 3.03 | 3.10e-01 | 0.4672865596 |
|  | 8 | 41504566 | 41505042 | PDFSTATSENSEMBLUCSC | 477 | 7 | 40247 | island | 0.272223 | 0.1138229 | Not queried | 3.03 | 2.59e-01 | 0.4381244265 |
|  | 8 | 41504884 | 41505042 | PDFSTATSENSEMBLUCSC | 159 | 5 | 39671 | tss | 0.272223 | 0.0607937 | Not queried | 3.03 | 6.90e-01 | 0.7223604052 |
|  | 15 | 96875591 | 96875830 | PDFSTATSENSEMBLUCSC | 240 | 8 | 13493 | gene;tss;island | 0.101365 | 0.2722256 | Not queried | 3.03 | 2.81e-02 | 0.1830867741 |
|  | 18 | 77793182 | 77793554 | PDFSTATSENSEMBLUCSC | 373 | 5 | 19340 | tss;island | 0.003679 | 0.0067487 | Not queried | 3.03 | 4.21e-01 | 0.5404252807 |
|  | 10 | 114043123 | 114043435 | PDFSTATSENSEMBLUCSC | 313 | 5 | 5364 | tss | 0.037647 | 0.2884384 | Not queried | 3.03 | 3.10e-01 | 0.4672865596 |
|  | 2 | 88427581 | 88427827 | PDFSTATSENSEMBLUCSC | 247 | 4 | 23499 | tss | 0.150518 | 0.4325552 | Not queried | 3.03 | 1.14e-01 | 0.3123457953 |
|  | 6 | 32018906 | 32019556 | PDFSTATSENSEMBLUCSC | 651 | 5 | 34017 | gene | 0.048981 | 0.2209213 | Not queried | 3.03 | 4.21e-01 | 0.5404252807 |
|  | 1 | 200992902 | 200993311 | PDFSTATSENSEMBLUCSC | 410 | 5 | 2163 | tss | 0.020736 | 0.0098914 | Not queried | 3.03 | 5.48e-01 | 0.6179718485 |
|  | 19 | 19383613 | 19383976 | PDFSTATSENSEMBLUCSC | 364 | 4 | 19949 | gene | 0.522827 | 0.1735715 | Not queried | 3.03 | 2.00e-01 | 0.3826488233 |
|  | 4 | 52942997 | 52943247 | PDFSTATSENSEMBLUCSC | 251 | 4 | 30001 | gene;island | 0.024595 | 0.1708308 | Not queried | 3.03 | 2.00e-01 | 0.3826488233 |
|  | 6 | 146350041 | 146350748 | PDFSTATSENSEMBLUCSC | 708 | 8 | 34500 | gene;island | 0.030115 | 0.4550148 | Not queried | 3.03 | 3.79e-02 | 0.2121545571 |
|  | 11 | 20690628 | 20691429 | PDFSTATSENSEMBLUCSC | 802 | 12 | 8131 | island | 0.007322 | 0.0429836 | Not queried | 3.03 | 6.81e-03 | 0.1589602024 |
|  | 17 | 46641708 | 46642104 | PDFSTATSENSEMBLUCSC | 397 | 4 | 16652 | gene;island | 0.910287 | 0.5354375 | Not queried | 3.03 | 2.00e-01 | 0.3826488233 |
|  | 1 | 63249693 | 63249793 | PDFSTATSENSEMBLUCSC | 101 | 4 | 1667 | tss | 0.000480 | 0.0145815 | Not queried | 3.02 | 3.43e-01 | 0.4672865596 |
|  | 3 | 55517496 | 55517853 | PDFSTATSENSEMBLUCSC | 358 | 4 | 27838 | gene;island | 0.007897 | 0.1784096 | Not queried | 3.02 | 3.43e-01 | 0.4672865596 |
|  | 12 | 119616616 | 119616913 | PDFSTATSENSEMBLUCSC | 298 | 5 | 9340 | gene | 0.034877 | 0.0067880 | Not queried | 3.02 | 1.51e-01 | 0.3618000441 |
|  | 17 | 73084263 | 73084494 | PDFSTATSENSEMBLUCSC | 232 | 4 | 16764 | gene;island | 0.057081 | 0.3457477 | Not queried | 3.02 | 1.14e-01 | 0.3123457953 |
|  | 16 | 3355295 | 3355553 | PDFSTATSENSEMBLUCSC | 259 | 4 | 15646 | island | 0.139602 | 0.0173491 | Not queried | 3.02 | 2.86e-02 | 0.1830867741 |
|  | 22 | 32767005 | 32767162 | PDFSTATSENSEMBLUCSC | 158 | 4 | 27091 | tss | 0.613961 | 0.8685027 | Not queried | 3.02 | 4.86e-01 | 0.5640816327 |
|  | 5 | 172661803 | 172662130 | PDFSTATSENSEMBLUCSC | 328 | 4 | 31904 | gene | 0.042999 | 0.0104252 | Not queried | 3.01 | 1.14e-01 | 0.3123457953 |
|  | 12 | 124990897 | 124991139 | PDFSTATSENSEMBLUCSC | 243 | 4 | 9399 | gene | 0.214400 | 0.7424805 | Not queried | 3.01 | 3.43e-01 | 0.4672865596 |
|  | 1 | 223316634 | 223316927 | PDFSTATSENSEMBLUCSC | 294 | 4 | 2269 | tss | 0.003709 | 0.0143718 | Not queried | 3.01 | 4.86e-01 | 0.5640816327 |
|  | 7 | 1022471 | 1023156 | PDFSTATSENSEMBLUCSC | 686 | 11 | 38063 | island | 0.457979 | 0.8603505 | Not queried | 3.01 | 1.04e-02 | 0.1806165940 |
|  | 7 | 1022841 | 1023156 | PDFSTATSENSEMBLUCSC | 316 | 5 | 36730 | gene | 0.704845 | 0.9108346 | Not queried | 3.01 | 2.22e-01 | 0.3935758299 |
|  | 17 | 16119344 | 16119456 | PDFSTATSENSEMBLUCSC | 113 | 4 | 17154 | tss;island | 0.119434 | 0.0278802 | Not queried | 3.01 | 2.00e-01 | 0.3826488233 |
|  | 13 | 38443950 | 38444227 | PDFSTATSENSEMBLUCSC | 278 | 6 | 11401 | tss | 0.040362 | 0.1381480 | Not queried | 3.01 | 9.31e-02 | 0.3104222821 |
|  | 14 | 89028798 | 89029224 | PDFSTATSENSEMBLUCSC | 427 | 6 | 12545 | tss;island | 0.078839 | 0.5668370 | Not queried | 3.01 | 2.40e-01 | 0.4133265075 |
|  | 2 | 27505396 | 27505593 | PDFSTATSENSEMBLUCSC | 198 | 5 | 23262 | tss | 0.046195 | 0.2696030 | Not queried | 3.01 | 7.94e-03 | 0.1589602024 |
|  | 1 | 247578859 | 247579312 | PDFSTATSENSEMBLUCSC | 454 | 4 | 2368 | tss | 0.020402 | 0.4114587 | Not queried | 3.00 | 3.43e-01 | 0.4672865596 |
|  | 19 | 50666238 | 50666552 | PDFSTATSENSEMBLUCSC | 315 | 6 | 22300 | island | 0.090369 | 0.5465316 | Not queried | 3.00 | 8.66e-03 | 0.1595382395 |
|  | 19 | 50666238 | 50666484 | PDFSTATSENSEMBLUCSC | 247 | 4 | 20171 | gene | 0.158800 | 0.5465316 | Not queried | 3.00 | 5.71e-02 | 0.2315877671 |
|  | 1 | 200011684 | 200011988 | PDFSTATSENSEMBLUCSC | 305 | 6 | 934 | gene;island | 0.102862 | 0.0108638 | Not queried | 3.00 | 1.32e-01 | 0.3397986247 |
|  | 6 | 126071099 | 126071538 | PDFSTATSENSEMBLUCSC | 440 | 5 | 34466 | gene;island | 0.041152 | 0.0103129 | Not queried | 3.00 | 1.59e-02 | 0.1830867741 |
|  | 1 | 1475644 | 1475742 | PDFSTATSENSEMBLUCSC | 99 | 4 | 2444 | island | 0.007740 | 0.0472580 | Not queried | 3.00 | 2.00e-01 | 0.3826488233 |
|  | 8 | 103750821 | 103751006 | PDFSTATSENSEMBLUCSC | 186 | 4 | 40513 | island | 0.560228 | 0.1280501 | Not queried | 3.00 | 1.14e-01 | 0.3123457953 |
|  | 7 | 127744210 | 127744617 | PDFSTATSENSEMBLUCSC | 408 | 4 | 38890 | island | 0.025671 | 0.0980616 | Not queried | 2.99 | 4.86e-01 | 0.5640816327 |
|  | 12 | 109162390 | 109162748 | PDFSTATSENSEMBLUCSC | 359 | 6 | 10773 | island | 0.277721 | 0.0295959 | Not queried | 2.99 | 4.33e-03 | 0.1459191215 |
|  | 7 | 100199952 | 100200009 | PDFSTATSENSEMBLUCSC | 58 | 4 | 37144 | gene | 0.572182 | 0.3226436 | Not queried | 2.99 | 2.00e-01 | 0.3826488233 |
|  | 19 | 12306136 | 12306497 | PDFSTATSENSEMBLUCSC | 362 | 4 | 21597 | island | 0.255375 | 0.0366453 | Not queried | 2.99 | 2.86e-02 | 0.1830867741 |
|  | 7 | 50861418 | 50862266 | PDFSTATSENSEMBLUCSC | 849 | 20 | 37598 | tss;island | 0.067392 | 0.6083027 | Not queried | 2.99 | 1.59e-03 | 0.0934794372 |
|  | 1 | 186649153 | 186649530 | PDFSTATSENSEMBLUCSC | 378 | 6 | 925 | gene | 0.063801 | 0.0065812 | Not queried | 2.99 | 2.60e-02 | 0.1830867741 |
|  | 6 | 30076659 | 30076980 | PDFSTATSENSEMBLUCSC | 322 | 4 | 33723 | gene | 0.156978 | 0.3289770 | Not queried | 2.99 | 4.86e-01 | 0.5640816327 |
|  | 20 | 5891730 | 5892091 | PDFSTATSENSEMBLUCSC | 362 | 7 | 25989 | island | 0.094335 | 0.0099436 | Not queried | 2.99 | 2.62e-02 | 0.1830867741 |
|  | 17 | 4545074 | 4545157 | PDFSTATSENSEMBLUCSC | 84 | 4 | 17027 | tss | 0.334800 | 0.0497522 | Not queried | 2.99 | 2.86e-02 | 0.1830867741 |
|  | 1 | 17746286 | 17746597 | PDFSTATSENSEMBLUCSC | 312 | 6 | 266 | gene;island | 0.953764 | 0.4738686 | Not queried | 2.99 | 9.31e-02 | 0.3104222821 |
|  | 10 | 1531243 | 1531529 | PDFSTATSENSEMBLUCSC | 287 | 4 | 4468 | gene | 0.227601 | 0.0275502 | Not queried | 2.99 | 5.71e-02 | 0.2315877671 |
|  | 5 | 135265753 | 135265937 | PDFSTATSENSEMBLUCSC | 185 | 5 | 32308 | tss | 0.015248 | 0.0950287 | Not queried | 2.98 | 1.51e-01 | 0.3618000441 |
|  | 7 | 43797839 | 43798207 | PDFSTATSENSEMBLUCSC | 369 | 7 | 37558 | tss;island | 0.009984 | 0.0625796 | Not queried | 2.98 | 1.75e-02 | 0.1830867741 |
|  | 20 | 51588780 | 51588860 | PDFSTATSENSEMBLUCSC | 81 | 4 | 25828 | tss;island | 0.010483 | 0.0685069 | Not queried | 2.98 | 2.86e-02 | 0.1830867741 |
|  | 10 | 114134878 | 114135259 | PDFSTATSENSEMBLUCSC | 382 | 5 | 4761 | gene;tss | 0.009330 | 0.0635108 | Not queried | 2.98 | 1.59e-02 | 0.1830867741 |
|  | 3 | 124931627 | 124931746 | PDFSTATSENSEMBLUCSC | 120 | 4 | 28550 | tss | 0.337487 | 0.0442416 | Not queried | 2.98 | 2.00e-01 | 0.3826488233 |
|  | 3 | 170746029 | 170746291 | PDFSTATSENSEMBLUCSC | 263 | 6 | 29661 | island | 0.089067 | 0.0207267 | Not queried | 2.98 | 1.80e-01 | 0.3826488233 |
|  | 7 | 32111001 | 32111453 | PDFSTATSENSEMBLUCSC | 453 | 7 | 37524 | tss | 0.104076 | 0.4828719 | Not queried | 2.98 | 3.18e-01 | 0.4672865596 |
|  | 11 | 18230754 | 18231021 | PDFSTATSENSEMBLUCSC | 268 | 5 | 6526 | gene | 0.537989 | 0.9017393 | Not queried | 2.98 | 1.51e-01 | 0.3618000441 |
|  | 8 | 144242029 | 144242448 | PDFSTATSENSEMBLUCSC | 420 | 6 | 39944 | tss;island | 0.040099 | 0.2454499 | Not queried | 2.98 | 1.80e-01 | 0.3826488233 |
|  | 1 | 118727658 | 118728226 | PDFSTATSENSEMBLUCSC | 569 | 10 | 3655 | island | 0.093216 | 0.6192023 | Not queried | 2.97 | 3.55e-02 | 0.2067926195 |
|  | 14 | 74705942 | 74706113 | PDFSTATSENSEMBLUCSC | 172 | 6 | 12505 | tss | 0.075209 | 0.4016325 | Not queried | 2.97 | 8.66e-03 | 0.1595382395 |
|  | 2 | 160654866 | 160655081 | PDFSTATSENSEMBLUCSC | 216 | 4 | 23682 | tss;island | 0.503513 | 0.1355615 | Not queried | 2.97 | 3.43e-01 | 0.4672865596 |
|  | 11 | 117296936 | 117297328 | PDFSTATSENSEMBLUCSC | 393 | 4 | 8847 | island | 0.027087 | 0.5855665 | Not queried | 2.97 | 6.86e-01 | 0.7223604052 |
|  | 3 | 153839743 | 153840718 | PDFSTATSENSEMBLUCSC | 976 | 11 | 28000 | gene;island | 0.165598 | 0.5299559 | Not queried | 2.97 | 6.52e-02 | 0.2483354067 |
|  | 22 | 45148218 | 45148489 | PDFSTATSENSEMBLUCSC | 272 | 10 | 26896 | gene | 0.012492 | 0.1469768 | Not queried | 2.97 | 1.05e-03 | 0.0821403610 |
|  | 8 | 16859295 | 16859671 | PDFSTATSENSEMBLUCSC | 377 | 5 | 39214 | gene | 0.070643 | 0.4472376 | Not queried | 2.96 | 2.22e-01 | 0.3935758299 |
|  | 18 | 23713407 | 23714084 | PDFSTATSENSEMBLUCSC | 678 | 10 | 19440 | island | 0.842550 | 0.5055680 | Not queried | 2.96 | 4.87e-04 | 0.0548331102 |
|  | 13 | 20805380 | 20805679 | PDFSTATSENSEMBLUCSC | 300 | 8 | 11330 | tss | 0.770574 | 0.3469432 | Not queried | 2.96 | 4.99e-02 | 0.2315877671 |
|  | 7 | 1198522 | 1198977 | PDFSTATSENSEMBLUCSC | 456 | 5 | 36752 | gene;island | 0.060816 | 0.4282235 | Not queried | 2.96 | 3.10e-01 | 0.4672865596 |
|  | 11 | 126872615 | 126872863 | PDFSTATSENSEMBLUCSC | 249 | 4 | 8936 | island | 0.562546 | 0.0689540 | Not queried | 2.96 | 4.86e-01 | 0.5640816327 |
|  | 20 | 30605656 | 30606095 | PDFSTATSENSEMBLUCSC | 440 | 5 | 25387 | gene;island | 0.046617 | 0.0289826 | Not queried | 2.96 | 2.22e-01 | 0.3935758299 |
|  | 16 | 8806966 | 8807043 | PDFSTATSENSEMBLUCSC | 78 | 5 | 14653 | gene | 0.032738 | 0.2556769 | Not queried | 2.96 | 5.56e-02 | 0.2315877671 |
|  | 8 | 24813866 | 24814137 | PDFSTATSENSEMBLUCSC | 272 | 4 | 40162 | island | 0.010504 | 0.0307879 | Not queried | 2.96 | 4.86e-01 | 0.5640816327 |
|  | 1 | 59042745 | 59043370 | PDFSTATSENSEMBLUCSC | 626 | 10 | 3314 | island | 0.369821 | 0.4224662 | Not queried | 2.96 | 2.80e-01 | 0.4672865596 |
|  | 17 | 33787402 | 33787739 | PDFSTATSENSEMBLUCSC | 338 | 4 | 18215 | island | 0.044184 | 0.3216334 | Not queried | 2.95 | 2.00e-01 | 0.3826488233 |
|  | 18 | 67069959 | 67070461 | PDFSTATSENSEMBLUCSC | 503 | 6 | 19123 | gene;island | 0.057877 | 0.3145806 | Not queried | 2.95 | 3.10e-01 | 0.4672865596 |
|  | 15 | 78933630 | 78933902 | PDFSTATSENSEMBLUCSC | 273 | 5 | 13838 | tss;island | 0.173589 | 0.0264398 | Not queried | 2.95 | 5.48e-01 | 0.6179718485 |
|  | 7 | 114055074 | 114055210 | PDFSTATSENSEMBLUCSC | 137 | 6 | 37192 | gene | 0.286109 | 0.6217738 | Not queried | 2.95 | 9.31e-02 | 0.3104222821 |
|  | 2 | 89065319 | 89065605 | PDFSTATSENSEMBLUCSC | 287 | 7 | 24507 | island | 0.372448 | 0.0377819 | Not queried | 2.95 | 5.30e-02 | 0.2315877671 |
|  | 13 | 20805094 | 20805679 | PDFSTATSENSEMBLUCSC | 586 | 10 | 11096 | gene;island | 0.761518 | 0.4580431 | Not queried | 2.94 | 1.05e-01 | 0.3123457953 |
|  | 6 | 31800885 | 31801070 | PDFSTATSENSEMBLUCSC | 186 | 4 | 35850 | island | 0.410065 | 0.6323439 | Not queried | 2.94 | 4.86e-01 | 0.5640816327 |
|  | 17 | 47113407 | 47113596 | PDFSTATSENSEMBLUCSC | 190 | 4 | 16671 | gene;island | 0.303094 | 0.5897038 | Not queried | 2.94 | 3.43e-01 | 0.4672865596 |
|  | 16 | 49499006 | 49499385 | PDFSTATSENSEMBLUCSC | 380 | 4 | 15908 | island | 0.321423 | 0.6207172 | Not queried | 2.94 | 4.86e-01 | 0.5640816327 |
|  | 6 | 32025886 | 32026350 | PDFSTATSENSEMBLUCSC | 465 | 10 | 34022 | gene | 0.133744 | 0.6148950 | Not queried | 2.94 | 4.33e-02 | 0.2287547702 |
|  | 10 | 129534107 | 129534411 | PDFSTATSENSEMBLUCSC | 305 | 4 | 5435 | tss | 0.201693 | 0.0115884 | Not queried | 2.94 | 6.86e-01 | 0.7223604052 |
|  | 8 | 49647579 | 49647833 | PDFSTATSENSEMBLUCSC | 255 | 6 | 39301 | gene | 0.251334 | 0.0274494 | Not queried | 2.94 | 6.49e-02 | 0.2477578846 |
|  | 7 | 4848683 | 4848939 | PDFSTATSENSEMBLUCSC | 257 | 4 | 36857 | gene;island | 0.310457 | 0.0189786 | Not queried | 2.94 | 3.43e-01 | 0.4672865596 |
|  | 10 | 125651370 | 125651726 | PDFSTATSENSEMBLUCSC | 357 | 8 | 6221 | island | 0.025220 | 0.4460377 | Not queried | 2.94 | 2.07e-02 | 0.1830867741 |
|  | 8 | 57350735 | 57351185 | PDFSTATSENSEMBLUCSC | 451 | 7 | 40313 | island | 0.644513 | 0.5476801 | Not queried | 2.93 | 3.83e-01 | 0.5168893301 |
|  | 19 | 496239 | 496428 | PDFSTATSENSEMBLUCSC | 190 | 5 | 20273 | tss | 0.021964 | 0.0819689 | Not queried | 2.93 | 1.51e-01 | 0.3618000441 |
|  | 20 | 44540777 | 44541153 | PDFSTATSENSEMBLUCSC | 377 | 6 | 26209 | island | 0.016897 | 0.2302050 | Not queried | 2.93 | 3.10e-01 | 0.4672865596 |
|  | 2 | 47150 | 47452 | PDFSTATSENSEMBLUCSC | 303 | 4 | 23151 | tss;island | 0.303830 | 0.0592925 | Not queried | 2.93 | 8.86e-01 | 0.8941250130 |
|  | 19 | 30866192 | 30866365 | PDFSTATSENSEMBLUCSC | 174 | 4 | 19970 | gene;island | 0.259554 | 0.0451556 | Not queried | 2.93 | 2.86e-02 | 0.1830867741 |
|  | 16 | 69141250 | 69141437 | PDFSTATSENSEMBLUCSC | 188 | 5 | 15326 | tss | 0.011495 | 0.2011220 | Not queried | 2.93 | 1.51e-01 | 0.3618000441 |
|  | 4 | 111397134 | 111397220 | PDFSTATSENSEMBLUCSC | 87 | 5 | 30548 | tss | 0.459156 | 0.8564763 | Not queried | 2.93 | 9.52e-02 | 0.3104222821 |
|  | 11 | 1463541 | 1463662 | PDFSTATSENSEMBLUCSC | 122 | 4 | 6419 | gene;island | 0.018674 | 0.2592572 | Not queried | 2.93 | 1.14e-01 | 0.3123457953 |
|  | 16 | 85832091 | 85832378 | PDFSTATSENSEMBLUCSC | 288 | 5 | 14881 | gene;tss;island | 0.048875 | 0.0074871 | Not queried | 2.93 | 3.10e-01 | 0.4672865596 |
|  | 17 | 73892982 | 73893209 | PDFSTATSENSEMBLUCSC | 228 | 6 | 18767 | island | 0.012444 | 0.0802483 | Not queried | 2.93 | 6.49e-02 | 0.2477578846 |
|  | 16 | 18995157 | 18995637 | PDFSTATSENSEMBLUCSC | 481 | 6 | 15732 | island | 0.032336 | 0.0099943 | Not queried | 2.93 | 1.80e-01 | 0.3826488233 |
|  | 15 | 67813132 | 67813371 | PDFSTATSENSEMBLUCSC | 240 | 5 | 13756 | tss;island | 0.003570 | 0.0046130 | Not queried | 2.93 | 4.21e-01 | 0.5404252807 |
|  | 5 | 32710456 | 32710882 | PDFSTATSENSEMBLUCSC | 427 | 6 | 32008 | tss;island | 0.288472 | 0.0219898 | Not queried | 2.93 | 9.31e-02 | 0.3104222821 |
|  | 12 | 71314587 | 71314752 | PDFSTATSENSEMBLUCSC | 166 | 4 | 9869 | tss | 0.017652 | 0.0912462 | Not queried | 2.92 | 1.14e-01 | 0.3123457953 |
|  | 14 | 105616988 | 105617308 | PDFSTATSENSEMBLUCSC | 321 | 4 | 12256 | gene;island | 0.408094 | 0.6868628 | Not queried | 2.92 | 3.43e-01 | 0.4672865596 |
|  | 1 | 243646235 | 243646402 | PDFSTATSENSEMBLUCSC | 168 | 4 | 1112 | gene;island | 0.061032 | 0.4198476 | Not queried | 2.92 | 1.14e-01 | 0.3123457953 |
|  | 2 | 105478656 | 105479054 | PDFSTATSENSEMBLUCSC | 399 | 4 | 24579 | island | 0.128924 | 0.0292300 | Not queried | 2.92 | 4.86e-01 | 0.5640816327 |
|  | 4 | 77341841 | 77342104 | PDFSTATSENSEMBLUCSC | 264 | 4 | 31117 | island | 0.518399 | 0.9023114 | Not queried | 2.92 | 1.14e-01 | 0.3123457953 |
|  | 14 | 23834860 | 23834995 | PDFSTATSENSEMBLUCSC | 136 | 6 | 12302 | tss | 0.011527 | 0.1023300 | Not queried | 2.92 | 6.49e-02 | 0.2477578846 |
|  | 1 | 153651886 | 153652247 | PDFSTATSENSEMBLUCSC | 362 | 4 | 765 | gene;island | 0.110120 | 0.3605126 | Not queried | 2.92 | 3.43e-01 | 0.4672865596 |
|  | 14 | 59931922 | 59932321 | PDFSTATSENSEMBLUCSC | 400 | 11 | 12892 | island | 0.006991 | 0.0349439 | Not queried | 2.92 | 2.33e-02 | 0.1830867741 |
|  | 14 | 59932101 | 59932321 | PDFSTATSENSEMBLUCSC | 221 | 9 | 12430 | tss | 0.006991 | 0.0349439 | Not queried | 2.92 | 6.25e-02 | 0.2477578846 |
|  | 22 | 31317764 | 31318546 | PDFSTATSENSEMBLUCSC | 783 | 12 | 27420 | island | 0.031392 | 0.4054548 | Not queried | 2.92 | 1.00e-02 | 0.1806165940 |
|  | 15 | 60294120 | 60294517 | PDFSTATSENSEMBLUCSC | 398 | 4 | 14188 | island | 0.061486 | 0.3139806 | Not queried | 2.91 | 3.43e-01 | 0.4672865596 |
|  | 3 | 64211155 | 64211659 | PDFSTATSENSEMBLUCSC | 505 | 5 | 28433 | tss | 0.510164 | 0.5489353 | Not queried | 2.91 | 3.10e-01 | 0.4672865596 |
|  | 20 | 43439387 | 43439688 | PDFSTATSENSEMBLUCSC | 302 | 5 | 25764 | tss;island | 0.008111 | 0.1287547 | Not queried | 2.91 | 5.48e-01 | 0.6179718485 |
|  | 4 | 81186795 | 81187198 | PDFSTATSENSEMBLUCSC | 404 | 5 | 30461 | tss;island | 0.004693 | 0.0385084 | Not queried | 2.91 | 5.56e-02 | 0.2315877671 |
|  | 12 | 81110749 | 81111012 | PDFSTATSENSEMBLUCSC | 264 | 4 | 9226 | gene | 0.148809 | 0.6113221 | Not queried | 2.91 | 1.14e-01 | 0.3123457953 |
|  | 19 | 15121204 | 15121531 | PDFSTATSENSEMBLUCSC | 328 | 7 | 20552 | tss | 0.451378 | 0.8462509 | Not queried | 2.91 | 1.11e-02 | 0.1821650572 |
|  | 2 | 176948693 | 176949017 | PDFSTATSENSEMBLUCSC | 325 | 7 | 23731 | tss | 0.251150 | 0.8614819 | Not queried | 2.91 | 2.62e-02 | 0.1830867741 |
|  | 2 | 176948544 | 176949017 | PDFSTATSENSEMBLUCSC | 474 | 9 | 24889 | island | 0.251150 | 0.8357278 | Not queried | 2.91 | 2.44e-02 | 0.1830867741 |
|  | 10 | 652260 | 652597 | PDFSTATSENSEMBLUCSC | 338 | 4 | 4445 | gene;island | 0.160202 | 0.6129444 | Not queried | 2.91 | 1.14e-01 | 0.3123457953 |
|  | 7 | 6655711 | 6655973 | PDFSTATSENSEMBLUCSC | 263 | 4 | 36887 | gene;island | 0.014053 | 0.2923783 | Not queried | 2.91 | 1.14e-01 | 0.3123457953 |
|  | 2 | 109065127 | 109065510 | PDFSTATSENSEMBLUCSC | 384 | 6 | 23564 | tss | 0.011806 | 0.0668655 | Not queried | 2.90 | 5.89e-01 | 0.6570484697 |
|  | 13 | 38443634 | 38444227 | PDFSTATSENSEMBLUCSC | 594 | 9 | 11678 | island | 0.031350 | 0.1410408 | Not queried | 2.90 | 7.70e-02 | 0.2825346647 |
|  | 22 | 24823389 | 24823519 | PDFSTATSENSEMBLUCSC | 131 | 5 | 27019 | tss | 0.016175 | 0.0569364 | Not queried | 2.90 | 9.52e-02 | 0.3104222821 |
|  | 19 | 46526100 | 46526675 | PDFSTATSENSEMBLUCSC | 576 | 10 | 22168 | island | 0.038626 | 0.4421039 | Not queried | 2.90 | 1.47e-02 | 0.1830867741 |
|  | 7 | 95063967 | 95064225 | PDFSTATSENSEMBLUCSC | 259 | 4 | 37097 | gene | 0.015171 | 0.1734971 | Not queried | 2.90 | 3.43e-01 | 0.4672865596 |
|  | 17 | 6796745 | 6797119 | PDFSTATSENSEMBLUCSC | 375 | 5 | 16378 | gene;island | 0.286622 | 0.5248991 | Not queried | 2.90 | 9.52e-02 | 0.3104222821 |
|  | 10 | 118900324 | 118900492 | PDFSTATSENSEMBLUCSC | 169 | 4 | 6173 | island | 0.042582 | 0.1807114 | Not queried | 2.90 | 1.14e-01 | 0.3123457953 |
|  | 1 | 2288167 | 2288463 | PDFSTATSENSEMBLUCSC | 297 | 4 | 77 | gene | 0.361261 | 0.5813857 | Not queried | 2.90 | 4.86e-01 | 0.5640816327 |
|  | 10 | 134751598 | 134751800 | PDFSTATSENSEMBLUCSC | 203 | 4 | 4882 | gene | 0.491085 | 0.8769495 | Not queried | 2.89 | 2.00e-01 | 0.3826488233 |
|  | 1 | 146649635 | 146649851 | PDFSTATSENSEMBLUCSC | 217 | 4 | 729 | gene;island | 0.962896 | 0.6791366 | Not queried | 2.89 | 5.71e-02 | 0.2315877671 |
|  | 3 | 29322342 | 29322474 | PDFSTATSENSEMBLUCSC | 133 | 4 | 28199 | tss | 0.018897 | 0.0039822 | Not queried | 2.89 | 5.71e-02 | 0.2315877671 |
|  | 5 | 63985282 | 63985489 | PDFSTATSENSEMBLUCSC | 208 | 4 | 32090 | tss;island | 0.144657 | 0.4276363 | Not queried | 2.89 | 3.43e-01 | 0.4672865596 |
|  | 5 | 110847639 | 110848106 | PDFSTATSENSEMBLUCSC | 468 | 4 | 31709 | gene | 0.005538 | 0.0187524 | Not queried | 2.89 | 4.86e-01 | 0.5640816327 |
|  | 2 | 30144547 | 30144838 | PDFSTATSENSEMBLUCSC | 292 | 6 | 23283 | tss | 0.012349 | 0.0045441 | Not queried | 2.89 | 1.32e-01 | 0.3397986247 |
|  | 2 | 1879820 | 1880131 | PDFSTATSENSEMBLUCSC | 312 | 4 | 22539 | gene | 0.415716 | 0.7664347 | Not queried | 2.89 | 3.43e-01 | 0.4672865596 |
|  | 2 | 3642098 | 3642967 | PDFSTATSENSEMBLUCSC | 870 | 11 | 24035 | island | 0.960428 | 0.7937236 | Not queried | 2.89 | 1.86e-03 | 0.0934794372 |
|  | 4 | 165898825 | 165898967 | PDFSTATSENSEMBLUCSC | 143 | 6 | 30666 | tss | 0.440492 | 0.7202763 | Not queried | 2.88 | 2.16e-03 | 0.0934794372 |
|  | 17 | 29248604 | 29248954 | PDFSTATSENSEMBLUCSC | 351 | 6 | 18183 | island | 0.009756 | 0.0623391 | Not queried | 2.88 | 4.11e-02 | 0.2180389943 |
|  | 11 | 1706894 | 1707163 | PDFSTATSENSEMBLUCSC | 270 | 4 | 6428 | gene;tss | 0.070491 | 0.3407529 | Not queried | 2.88 | 1.14e-01 | 0.3123457953 |
|  | 1 | 15250209 | 15250388 | PDFSTATSENSEMBLUCSC | 180 | 4 | 239 | gene;tss;island | 0.058313 | 0.2994698 | Not queried | 2.88 | 4.86e-01 | 0.5640816327 |
|  | 13 | 112668130 | 112668501 | PDFSTATSENSEMBLUCSC | 372 | 6 | 11906 | island | 0.541126 | 0.8977076 | Not queried | 2.88 | 1.80e-01 | 0.3826488233 |
|  | 11 | 1328419 | 1328622 | PDFSTATSENSEMBLUCSC | 204 | 4 | 6415 | gene;island | 0.926341 | 0.5773456 | Not queried | 2.88 | 2.00e-01 | 0.3826488233 |
|  | 19 | 16394366 | 16394774 | PDFSTATSENSEMBLUCSC | 409 | 5 | 21716 | island | 0.048301 | 0.3150751 | Not queried | 2.88 | 3.10e-01 | 0.4672865596 |
|  | 16 | 56224504 | 56224901 | PDFSTATSENSEMBLUCSC | 398 | 5 | 14755 | gene | 0.044629 | 0.0041589 | Not queried | 2.88 | 3.17e-02 | 0.1866936846 |
|  | 8 | 33457483 | 33457822 | PDFSTATSENSEMBLUCSC | 340 | 6 | 39642 | tss | 0.096896 | 0.0117201 | Not queried | 2.88 | 9.31e-02 | 0.3104222821 |
|  | 6 | 30122947 | 30123226 | PDFSTATSENSEMBLUCSC | 280 | 5 | 33730 | gene | 0.154383 | 0.4952480 | Not queried | 2.88 | 1.51e-01 | 0.3618000441 |
|  | 10 | 5708674 | 5708881 | PDFSTATSENSEMBLUCSC | 208 | 6 | 4927 | tss;island | 0.003619 | 0.0273606 | Not queried | 2.88 | 6.49e-02 | 0.2477578846 |
|  | 6 | 112575705 | 112576119 | PDFSTATSENSEMBLUCSC | 415 | 6 | 36423 | island | 0.142186 | 0.5024326 | Not queried | 2.87 | 2.60e-02 | 0.1830867741 |
|  | 6 | 30460244 | 30460798 | PDFSTATSENSEMBLUCSC | 555 | 7 | 33771 | gene;island | 0.532756 | 0.9459607 | Not queried | 2.87 | 2.09e-01 | 0.3935758299 |
|  | 16 | 8806359 | 8806756 | PDFSTATSENSEMBLUCSC | 398 | 8 | 14652 | gene;tss | 0.067463 | 0.3118311 | Not queried | 2.87 | 2.07e-02 | 0.1830867741 |
|  | 12 | 7167584 | 7167900 | PDFSTATSENSEMBLUCSC | 317 | 4 | 9543 | tss | 0.262939 | 0.7941673 | Not queried | 2.87 | 1.14e-01 | 0.3123457953 |
|  | 14 | 34420292 | 34420683 | PDFSTATSENSEMBLUCSC | 392 | 8 | 12345 | tss | 0.015686 | 0.0059612 | Not queried | 2.87 | 1.04e-02 | 0.1806165940 |
|  | 1 | 22140769 | 22141400 | PDFSTATSENSEMBLUCSC | 632 | 7 | 301 | gene;island | 0.050378 | 0.5128944 | Not queried | 2.87 | 3.83e-01 | 0.5168893301 |
|  | 1 | 24645802 | 24645853 | PDFSTATSENSEMBLUCSC | 52 | 4 | 1358 | tss | 0.009698 | 0.1234896 | Not queried | 2.87 | 3.43e-01 | 0.4672865596 |
|  | 6 | 100442105 | 100442151 | PDFSTATSENSEMBLUCSC | 47 | 4 | 35138 | tss | 0.350790 | 0.0420067 | Not queried | 2.87 | 3.43e-01 | 0.4672865596 |
|  | 4 | 74718978 | 74719289 | PDFSTATSENSEMBLUCSC | 312 | 5 | 31096 | island | 0.314276 | 0.0591095 | Not queried | 2.87 | 2.22e-01 | 0.3935758299 |
|  | 7 | 32110988 | 32111453 | PDFSTATSENSEMBLUCSC | 466 | 8 | 38393 | island | 0.080096 | 0.4801985 | Not queried | 2.86 | 3.28e-01 | 0.4672865596 |
|  | 4 | 62382932 | 62383240 | PDFSTATSENSEMBLUCSC | 309 | 4 | 30012 | gene;island | 0.551287 | 0.0363811 | Not queried | 2.86 | 5.71e-02 | 0.2315877671 |
|  | 1 | 22470218 | 22470343 | PDFSTATSENSEMBLUCSC | 126 | 4 | 1331 | tss;island | 0.015056 | 0.2099592 | Not queried | 2.86 | 4.86e-01 | 0.5640816327 |
|  | 13 | 79181285 | 79181613 | PDFSTATSENSEMBLUCSC | 329 | 4 | 11795 | island | 0.047554 | 0.2648847 | Not queried | 2.86 | 2.00e-01 | 0.3826488233 |
|  | 5 | 16784775 | 16785135 | PDFSTATSENSEMBLUCSC | 361 | 5 | 31570 | gene | 0.184098 | 0.6081389 | Not queried | 2.86 | 1.51e-01 | 0.3618000441 |
|  | 20 | 24451769 | 24452131 | PDFSTATSENSEMBLUCSC | 363 | 6 | 25376 | gene;island | 0.005000 | 0.0492387 | Not queried | 2.86 | 6.49e-02 | 0.2477578846 |
|  | 14 | 90042201 | 90042364 | PDFSTATSENSEMBLUCSC | 164 | 4 | 12171 | gene;tss | 0.979529 | 0.8646607 | Not queried | 2.86 | 2.86e-02 | 0.1830867741 |
|  | 3 | 49236800 | 49236946 | PDFSTATSENSEMBLUCSC | 147 | 6 | 27778 | gene;island | 0.788691 | 0.3136714 | Not queried | 2.86 | 6.49e-02 | 0.2477578846 |
|  | 4 | 74734714 | 74735101 | PDFSTATSENSEMBLUCSC | 388 | 9 | 30430 | tss | 0.020878 | 0.3862605 | Not queried | 2.86 | 1.06e-02 | 0.1810746987 |
|  | 2 | 200329486 | 200329872 | PDFSTATSENSEMBLUCSC | 387 | 7 | 24992 | island | 0.017430 | 0.1067467 | Not queried | 2.86 | 7.28e-02 | 0.2713481487 |
|  | 10 | 77542302 | 77542488 | PDFSTATSENSEMBLUCSC | 187 | 7 | 5177 | tss | 0.896352 | 0.3628286 | Not queried | 2.86 | 1.28e-01 | 0.3397986247 |
|  | 19 | 49655176 | 49655517 | PDFSTATSENSEMBLUCSC | 342 | 4 | 20145 | gene;island | 0.329051 | 0.0447399 | Not queried | 2.86 | 5.71e-02 | 0.2315877671 |
|  | 2 | 220362124 | 220362242 | PDFSTATSENSEMBLUCSC | 119 | 4 | 23907 | tss | 0.044168 | 0.4139588 | Not queried | 2.86 | 2.00e-01 | 0.3826488233 |
|  | 12 | 25102311 | 25102521 | PDFSTATSENSEMBLUCSC | 211 | 4 | 9608 | tss | 0.002829 | 0.0189065 | Not queried | 2.86 | 2.86e-02 | 0.1830867741 |
|  | 17 | 41985144 | 41985408 | PDFSTATSENSEMBLUCSC | 265 | 4 | 17396 | tss | 0.244841 | 0.0133478 | Not queried | 2.86 | 2.00e-01 | 0.3826488233 |
|  | 13 | 25670047 | 25670238 | PDFSTATSENSEMBLUCSC | 192 | 9 | 11349 | tss | 0.531597 | 0.1361886 | PABPC3 (margin: PABPC3) | 2.86 | 4.11e-05 | 0.0155042818 |
|  | 17 | 46799479 | 46800028 | PDFSTATSENSEMBLUCSC | 550 | 9 | 17469 | tss;island | 0.472348 | 0.0913897 | Not queried | 2.86 | 1.90e-01 | 0.3826488233 |
|  | 6 | 33132943 | 33133083 | PDFSTATSENSEMBLUCSC | 141 | 4 | 34194 | gene;island | 0.441884 | 0.7029882 | Not queried | 2.85 | 4.86e-01 | 0.5640816327 |
|  | 6 | 153452724 | 153452954 | PDFSTATSENSEMBLUCSC | 231 | 4 | 35310 | tss;island | 0.388419 | 0.0516849 | Not queried | 2.85 | 1.14e-01 | 0.3123457953 |
|  | 5 | 123987423 | 123987747 | PDFSTATSENSEMBLUCSC | 325 | 5 | 31733 | gene;island | 0.006901 | 0.0099487 | Not queried | 2.85 | 3.10e-01 | 0.4672865596 |
|  | 5 | 68710808 | 68710912 | PDFSTATSENSEMBLUCSC | 105 | 6 | 32110 | tss;island | 0.022447 | 0.1705138 | Not queried | 2.85 | 3.10e-01 | 0.4672865596 |
|  | 3 | 27766009 | 27766382 | PDFSTATSENSEMBLUCSC | 374 | 6 | 28953 | island | 0.210773 | 0.0464318 | Not queried | 2.85 | 2.40e-01 | 0.4133265075 |
|  | 11 | 63871228 | 63871343 | PDFSTATSENSEMBLUCSC | 116 | 5 | 6663 | gene;tss | 0.169922 | 0.6777335 | Not queried | 2.85 | 9.52e-02 | 0.3104222821 |
|  | 20 | 36889208 | 36889389 | PDFSTATSENSEMBLUCSC | 182 | 7 | 25732 | tss | 0.091445 | 0.3858719 | Not queried | 2.84 | 1.75e-02 | 0.1830867741 |
|  | 7 | 56297490 | 56297714 | PDFSTATSENSEMBLUCSC | 225 | 5 | 38513 | island | 0.109067 | 0.0906536 | Not queried | 2.84 | 2.22e-01 | 0.3935758299 |
|  | 14 | 65569751 | 65570008 | PDFSTATSENSEMBLUCSC | 258 | 4 | 12467 | tss;island | 0.001185 | 0.0030038 | Not queried | 2.84 | 4.86e-01 | 0.5640816327 |
|  | 2 | 242716388 | 242716801 | PDFSTATSENSEMBLUCSC | 414 | 4 | 23145 | gene | 0.438128 | 0.8711536 | Not queried | 2.84 | 1.14e-01 | 0.3123457953 |
|  | 1 | 86622395 | 86622737 | PDFSTATSENSEMBLUCSC | 343 | 6 | 1725 | tss;island | 0.007640 | 0.0263222 | Not queried | 2.84 | 3.10e-01 | 0.4672865596 |
|  | 16 | 1146277 | 1146545 | PDFSTATSENSEMBLUCSC | 269 | 4 | 14988 | tss | 0.512512 | 0.8782966 | Not queried | 2.83 | 1.14e-01 | 0.3123457953 |
|  | 8 | 117950244 | 117950454 | PDFSTATSENSEMBLUCSC | 211 | 5 | 39882 | tss | 0.362681 | 0.0836967 | Not queried | 2.83 | 5.56e-02 | 0.2315877671 |
|  | 1 | 214162545 | 214162854 | PDFSTATSENSEMBLUCSC | 310 | 5 | 1004 | gene;island | 0.035173 | 0.0694327 | Not queried | 2.83 | 6.90e-01 | 0.7223604052 |
|  | 8 | 12990898 | 12991196 | PDFSTATSENSEMBLUCSC | 299 | 7 | 39560 | tss | 0.107552 | 0.0085484 | Not queried | 2.83 | 2.62e-02 | 0.1830867741 |
|  | 5 | 76925380 | 76925623 | PDFSTATSENSEMBLUCSC | 244 | 4 | 31655 | gene;island | 0.185293 | 0.0471344 | Not queried | 2.83 | 3.43e-01 | 0.4672865596 |
|  | 11 | 20181877 | 20182122 | PDFSTATSENSEMBLUCSC | 246 | 7 | 7195 | tss | 0.189189 | 0.0959135 | Not queried | 2.83 | 5.35e-01 | 0.6176455124 |
|  | 11 | 624457 | 624995 | PDFSTATSENSEMBLUCSC | 539 | 5 | 6386 | gene | 0.497702 | 0.8881315 | Not queried | 2.83 | 5.56e-02 | 0.2315877671 |
|  | 7 | 106685015 | 106685033 | PDFSTATSENSEMBLUCSC | 19 | 4 | 37823 | tss;island | 0.000958 | 0.0033418 | Not queried | 2.83 | 5.71e-02 | 0.2315877671 |
|  | 11 | 116371188 | 116371487 | PDFSTATSENSEMBLUCSC | 300 | 4 | 8832 | island | 0.045321 | 0.2673187 | Not queried | 2.83 | 3.43e-01 | 0.4672865596 |
|  | 6 | 168426063 | 168426413 | PDFSTATSENSEMBLUCSC | 351 | 5 | 34557 | gene | 0.127598 | 0.6312924 | Not queried | 2.83 | 3.10e-01 | 0.4672865596 |
|  | 17 | 46871582 | 46871905 | PDFSTATSENSEMBLUCSC | 324 | 6 | 16667 | gene | 0.493072 | 0.6916913 | Not queried | 2.83 | 3.10e-01 | 0.4672865596 |
|  | 19 | 4531638 | 4531961 | PDFSTATSENSEMBLUCSC | 324 | 4 | 19735 | gene;island | 0.413688 | 0.0506504 | Not queried | 2.83 | 1.14e-01 | 0.3123457953 |
|  | 7 | 95064691 | 95064926 | PDFSTATSENSEMBLUCSC | 236 | 6 | 37728 | tss;island | 0.049465 | 0.0224180 | Not queried | 2.83 | 5.89e-01 | 0.6570484697 |
|  | 1 | 200860363 | 200860682 | PDFSTATSENSEMBLUCSC | 320 | 4 | 4069 | island | 0.005878 | 0.1128146 | Not queried | 2.82 | 3.43e-01 | 0.4672865596 |
|  | 1 | 209848845 | 209849185 | PDFSTATSENSEMBLUCSC | 341 | 5 | 986 | gene;island | 0.026664 | 0.1864591 | Not queried | 2.82 | 7.94e-03 | 0.1589602024 |
|  | 22 | 50968250 | 50968516 | PDFSTATSENSEMBLUCSC | 267 | 6 | 27629 | island | 0.015136 | 0.0079119 | Not queried | 2.82 | 4.85e-01 | 0.5640816327 |
|  | 16 | 28948092 | 28948319 | PDFSTATSENSEMBLUCSC | 228 | 4 | 14708 | gene;island | 0.798091 | 0.3111220 | Not queried | 2.82 | 6.86e-01 | 0.7223604052 |
|  | 7 | 88425077 | 88425148 | PDFSTATSENSEMBLUCSC | 72 | 5 | 37693 | tss | 0.687849 | 0.9054183 | Not queried | 2.82 | 4.21e-01 | 0.5404252807 |
|  | 11 | 122852412 | 122852698 | PDFSTATSENSEMBLUCSC | 287 | 8 | 7775 | tss | 0.043201 | 0.1965441 | Not queried | 2.82 | 1.61e-01 | 0.3814043454 |
|  | 1 | 2040968 | 2041228 | PDFSTATSENSEMBLUCSC | 261 | 4 | 60 | gene;island | 0.411277 | 0.7409765 | Not queried | 2.82 | 2.00e-01 | 0.3826488233 |
|  | 17 | 1548881 | 1549434 | PDFSTATSENSEMBLUCSC | 554 | 6 | 17821 | island | 0.248519 | 0.8381442 | Not queried | 2.82 | 1.32e-01 | 0.3397986247 |
|  | 13 | 25670047 | 25670327 | PDFSTATSENSEMBLUCSC | 281 | 11 | 11609 | island | 0.505984 | 0.1361886 | PABPC3 (margin: PABPC3) | 2.82 | 8.51e-05 | 0.0220396863 |
|  | 4 | 85414016 | 85414194 | PDFSTATSENSEMBLUCSC | 179 | 4 | 31159 | island | 0.219917 | 0.0340369 | Not queried | 2.82 | 2.00e-01 | 0.3826488233 |
|  | 22 | 51038379 | 51038723 | PDFSTATSENSEMBLUCSC | 345 | 4 | 27262 | tss;island | 0.304639 | 0.0552868 | Not queried | 2.81 | 4.86e-01 | 0.5640816327 |
|  | 3 | 36902435 | 36902745 | PDFSTATSENSEMBLUCSC | 311 | 4 | 27715 | gene | 0.650937 | 0.9386729 | Not queried | 2.81 | 2.86e-02 | 0.1830867741 |
|  | 3 | 147128998 | 147129278 | PDFSTATSENSEMBLUCSC | 281 | 4 | 27989 | gene;island | 0.488049 | 0.1165188 | Not queried | 2.81 | 2.00e-01 | 0.3826488233 |
|  | 6 | 100442002 | 100442151 | PDFSTATSENSEMBLUCSC | 150 | 5 | 36361 | island | 0.269554 | 0.0396720 | Not queried | 2.81 | 4.21e-01 | 0.5404252807 |
|  | 7 | 27127448 | 27127752 | PDFSTATSENSEMBLUCSC | 305 | 4 | 38334 | island | 0.566560 | 0.1958705 | Not queried | 2.81 | 2.00e-01 | 0.3826488233 |
|  | 1 | 18956762 | 18957023 | PDFSTATSENSEMBLUCSC | 262 | 4 | 1295 | tss;island | 0.030912 | 0.1469318 | Not queried | 2.81 | 1.14e-01 | 0.3123457953 |
|  | 11 | 17498514 | 17498619 | PDFSTATSENSEMBLUCSC | 106 | 6 | 7176 | tss | 0.049513 | 0.1155710 | Not queried | 2.81 | 9.31e-02 | 0.3104222821 |
|  | 8 | 133573739 | 133574073 | PDFSTATSENSEMBLUCSC | 335 | 5 | 39919 | tss | 0.003771 | 0.1937665 | Not queried | 2.81 | 2.22e-01 | 0.3935758299 |
|  | 7 | 158379132 | 158379348 | PDFSTATSENSEMBLUCSC | 217 | 4 | 37379 | gene;island | 0.158827 | 0.6910585 | Not queried | 2.81 | 2.00e-01 | 0.3826488233 |
|  | 19 | 54876728 | 54876795 | PDFSTATSENSEMBLUCSC | 68 | 4 | 21060 | tss | 0.083578 | 0.3616424 | Not queried | 2.81 | 2.00e-01 | 0.3826488233 |
|  | 6 | 10695206 | 10695389 | PDFSTATSENSEMBLUCSC | 184 | 5 | 33565 | gene | 0.012966 | 0.0029640 | Not queried | 2.81 | 5.56e-02 | 0.2315877671 |
|  | 13 | 20717346 | 20717597 | PDFSTATSENSEMBLUCSC | 252 | 4 | 11093 | gene;island | 0.714056 | 0.9342137 | Not queried | 2.81 | 5.71e-02 | 0.2315877671 |
|  | 15 | 93652578 | 93653073 | PDFSTATSENSEMBLUCSC | 496 | 6 | 14482 | island | 0.138207 | 0.4800835 | Not queried | 2.80 | 4.11e-02 | 0.2180389943 |
|  | 6 | 4775063 | 4775222 | PDFSTATSENSEMBLUCSC | 160 | 4 | 33541 | gene;island | 0.058020 | 0.1985458 | Not queried | 2.80 | 4.86e-01 | 0.5640816327 |
|  | 11 | 368351 | 369192 | PDFSTATSENSEMBLUCSC | 842 | 24 | 7009 | tss | 0.033820 | 0.3225201 | B4GALNT4 (margin: B4GALNT4) | 2.80 | 2.74e-04 | 0.0455223165 |
|  | 7 | 103630013 | 103630093 | PDFSTATSENSEMBLUCSC | 81 | 4 | 37813 | tss;island | 0.011619 | 0.1414593 | Not queried | 2.80 | 1.14e-01 | 0.3123457953 |
|  | 1 | 229406360 | 229406711 | PDFSTATSENSEMBLUCSC | 352 | 6 | 2310 | tss;island | 0.618205 | 0.3115503 | Not queried | 2.80 | 6.99e-01 | 0.7290267580 |
|  | 4 | 4867387 | 4867638 | PDFSTATSENSEMBLUCSC | 252 | 4 | 30873 | island | 0.289721 | 0.0479401 | Not queried | 2.80 | 2.86e-02 | 0.1830867741 |
|  | 17 | 79225573 | 79225974 | PDFSTATSENSEMBLUCSC | 402 | 4 | 16902 | gene | 0.385128 | 0.0801736 | Not queried | 2.80 | 3.43e-01 | 0.4672865596 |
|  | 11 | 31837104 | 31837607 | PDFSTATSENSEMBLUCSC | 504 | 7 | 6563 | gene;island | 0.058747 | 0.0338451 | Not queried | 2.80 | 2.59e-01 | 0.4381244265 |
|  | 8 | 18541446 | 18541627 | PDFSTATSENSEMBLUCSC | 182 | 5 | 39219 | gene | 0.046108 | 0.2514736 | Not queried | 2.80 | 3.10e-01 | 0.4672865596 |
|  | 6 | 33943520 | 33943929 | PDFSTATSENSEMBLUCSC | 410 | 5 | 36048 | island | 0.070865 | 0.2483905 | Not queried | 2.80 | 9.52e-02 | 0.3104222821 |
|  | 2 | 38978654 | 38978896 | PDFSTATSENSEMBLUCSC | 243 | 6 | 23313 | tss | 0.019159 | 0.0215297 | Not queried | 2.80 | 5.89e-01 | 0.6570484697 |
|  | 4 | 155663689 | 155664311 | PDFSTATSENSEMBLUCSC | 623 | 7 | 30646 | tss | 0.038651 | 0.1386183 | Not queried | 2.79 | 9.73e-02 | 0.3123457953 |
|  | 19 | 41698814 | 41699065 | PDFSTATSENSEMBLUCSC | 252 | 9 | 20802 | tss;island | 0.060469 | 0.3086603 | Not queried | 2.79 | 1.13e-01 | 0.3123457953 |
|  | 1 | 28994850 | 28995195 | PDFSTATSENSEMBLUCSC | 346 | 6 | 1435 | tss | 0.002796 | 0.0017698 | Not queried | 2.79 | 3.94e-01 | 0.5236526859 |
|  | 22 | 45809244 | 45809454 | PDFSTATSENSEMBLUCSC | 211 | 6 | 26903 | gene | 0.583575 | 0.2374703 | Not queried | 2.79 | 1.32e-01 | 0.3397986247 |
|  | 3 | 10857688 | 10857800 | PDFSTATSENSEMBLUCSC | 113 | 8 | 28153 | tss;island | 0.255645 | 0.5182897 | Not queried | 2.79 | 8.30e-02 | 0.3028612233 |
|  | 14 | 104940229 | 104940658 | PDFSTATSENSEMBLUCSC | 430 | 5 | 13206 | island | 0.355911 | 0.7924069 | Not queried | 2.79 | 7.94e-03 | 0.1589602024 |
|  | 12 | 14720805 | 14721288 | PDFSTATSENSEMBLUCSC | 484 | 9 | 9584 | tss | 0.887066 | 0.4882384 | Not queried | 2.79 | 2.22e-01 | 0.3935758299 |
|  | 7 | 121950924 | 121951055 | PDFSTATSENSEMBLUCSC | 132 | 4 | 38872 | island | 0.094822 | 0.4335543 | Not queried | 2.79 | 3.43e-01 | 0.4672865596 |
|  | 4 | 41694736 | 41694847 | PDFSTATSENSEMBLUCSC | 112 | 4 | 29992 | gene | 0.661637 | 0.9405007 | Not queried | 2.79 | 3.43e-01 | 0.4672865596 |
|  | 6 | 134213992 | 134214307 | PDFSTATSENSEMBLUCSC | 316 | 4 | 34480 | gene;island | 0.058514 | 0.4697679 | Not queried | 2.79 | 3.43e-01 | 0.4672865596 |
|  | 1 | 16553267 | 16553549 | PDFSTATSENSEMBLUCSC | 283 | 5 | 2740 | island | 0.758189 | 0.5187543 | Not queried | 2.79 | 5.48e-01 | 0.6179718485 |
|  | 20 | 2731102 | 2731560 | PDFSTATSENSEMBLUCSC | 459 | 4 | 25337 | gene;island | 0.038043 | 0.2859799 | Not queried | 2.79 | 1.14e-01 | 0.3123457953 |
|  | 11 | 31821298 | 31821764 | PDFSTATSENSEMBLUCSC | 467 | 6 | 6556 | gene;island | 0.036663 | 0.3133859 | Not queried | 2.79 | 6.49e-02 | 0.2477578846 |
|  | 20 | 36888790 | 36889389 | PDFSTATSENSEMBLUCSC | 600 | 10 | 26153 | island | 0.061722 | 0.2492586 | Not queried | 2.78 | 6.30e-02 | 0.2477578846 |
|  | 11 | 65123257 | 65123609 | PDFSTATSENSEMBLUCSC | 353 | 4 | 6706 | gene;island | 0.670690 | 0.2804171 | Not queried | 2.78 | 2.00e-01 | 0.3826488233 |
|  | 13 | 114880761 | 114881194 | PDFSTATSENSEMBLUCSC | 434 | 6 | 11316 | gene;island | 0.556425 | 0.9015543 | Not queried | 2.78 | 1.32e-01 | 0.3397986247 |
|  | 8 | 116681611 | 116681744 | PDFSTATSENSEMBLUCSC | 134 | 4 | 39878 | tss;island | 0.026443 | 0.0053167 | Not queried | 2.78 | 2.00e-01 | 0.3826488233 |
|  | 15 | 91423337 | 91423574 | PDFSTATSENSEMBLUCSC | 238 | 4 | 13477 | gene;island | 0.959332 | 0.6790939 | Not queried | 2.78 | 2.00e-01 | 0.3826488233 |
|  | 10 | 118897836 | 118898082 | PDFSTATSENSEMBLUCSC | 247 | 6 | 5388 | tss;island | 0.124845 | 0.0112025 | Not queried | 2.78 | 4.11e-02 | 0.2180389943 |
|  | 11 | 67183148 | 67183616 | PDFSTATSENSEMBLUCSC | 469 | 5 | 6753 | gene | 0.081083 | 0.5308419 | Not queried | 2.78 | 1.51e-01 | 0.3618000441 |
|  | 4 | 183064851 | 183065224 | PDFSTATSENSEMBLUCSC | 374 | 4 | 30191 | gene;island | 0.090929 | 0.0183712 | Not queried | 2.78 | 2.86e-02 | 0.1830867741 |
|  | 1 | 154474344 | 154474530 | PDFSTATSENSEMBLUCSC | 187 | 4 | 778 | gene;tss;island | 0.269743 | 0.0431317 | Not queried | 2.78 | 2.86e-02 | 0.1830867741 |
|  | 12 | 104697193 | 104697545 | PDFSTATSENSEMBLUCSC | 353 | 10 | 9959 | tss | 0.061978 | 0.0239481 | Not queried | 2.78 | 1.43e-01 | 0.3618000441 |
|  | 2 | 238777587 | 238777806 | PDFSTATSENSEMBLUCSC | 220 | 4 | 23093 | gene | 0.007723 | 0.0412056 | Not queried | 2.77 | 2.86e-02 | 0.1830867741 |
|  | 7 | 28996196 | 28996639 | PDFSTATSENSEMBLUCSC | 444 | 5 | 36947 | gene;island | 0.390597 | 0.0428360 | Not queried | 2.77 | 3.17e-02 | 0.1866936846 |
|  | 18 | 12254365 | 12254690 | PDFSTATSENSEMBLUCSC | 326 | 9 | 19060 | gene | 0.045075 | 0.3233203 | CIDEA (margin: RP11-64C12.9; CIDEA) | 2.77 | 1.65e-04 | 0.0296603652 |
|  | 10 | 43047134 | 43047371 | PDFSTATSENSEMBLUCSC | 238 | 4 | 4565 | gene;island | 0.550448 | 0.9114319 | Not queried | 2.77 | 2.00e-01 | 0.3826488233 |
|  | 7 | 88424985 | 88425148 | PDFSTATSENSEMBLUCSC | 164 | 6 | 37079 | gene | 0.571694 | 0.8756798 | Not queried | 2.77 | 3.10e-01 | 0.4672865596 |
|  | 12 | 131303016 | 131303247 | PDFSTATSENSEMBLUCSC | 232 | 4 | 9418 | gene;island | 0.647196 | 0.3889138 | Not queried | 2.77 | 3.43e-01 | 0.4672865596 |
|  | 13 | 88324432 | 88324711 | PDFSTATSENSEMBLUCSC | 280 | 4 | 11494 | tss | 0.020837 | 0.1192512 | Not queried | 2.77 | 2.86e-02 | 0.1830867741 |
|  | 7 | 95401692 | 95401856 | PDFSTATSENSEMBLUCSC | 165 | 5 | 38683 | island | 0.014207 | 0.0297380 | Not queried | 2.77 | 9.52e-02 | 0.3104222821 |
|  | 1 | 240656217 | 240656737 | PDFSTATSENSEMBLUCSC | 521 | 6 | 1106 | gene;island | 0.470493 | 0.8323197 | Not queried | 2.76 | 2.16e-03 | 0.0934794372 |
|  | 21 | 44088823 | 44089142 | PDFSTATSENSEMBLUCSC | 320 | 4 | 26439 | gene;island | 0.703637 | 0.9571283 | Not queried | 2.76 | 2.00e-01 | 0.3826488233 |
|  | 1 | 204951031 | 204951336 | PDFSTATSENSEMBLUCSC | 306 | 4 | 961 | gene;island | 0.244757 | 0.4479910 | Not queried | 2.76 | 4.86e-01 | 0.5640816327 |
|  | 1 | 50886782 | 50886969 | PDFSTATSENSEMBLUCSC | 188 | 4 | 525 | gene;island | 0.270326 | 0.0509012 | Not queried | 2.76 | 4.86e-01 | 0.5640816327 |
|  | 1 | 247171403 | 247171838 | PDFSTATSENSEMBLUCSC | 436 | 7 | 2365 | tss | 0.102150 | 0.0149454 | Not queried | 2.76 | 1.28e-01 | 0.3397986247 |
|  | 10 | 101874829 | 101875138 | PDFSTATSENSEMBLUCSC | 310 | 5 | 6018 | island | 0.631686 | 0.9168938 | Not queried | 2.76 | 4.21e-01 | 0.5404252807 |
|  | 17 | 4544925 | 4545157 | PDFSTATSENSEMBLUCSC | 233 | 5 | 17873 | island | 0.215835 | 0.0428863 | Not queried | 2.76 | 1.59e-02 | 0.1830867741 |
|  | 15 | 51385812 | 51385970 | PDFSTATSENSEMBLUCSC | 159 | 4 | 13348 | gene;island | 0.001523 | 0.0055494 | Not queried | 2.76 | 4.86e-01 | 0.5640816327 |
|  | 12 | 34500585 | 34500813 | PDFSTATSENSEMBLUCSC | 229 | 4 | 10322 | island | 0.546290 | 0.8447644 | Not queried | 2.75 | 2.86e-02 | 0.1830867741 |
|  | 7 | 1748302 | 1748766 | PDFSTATSENSEMBLUCSC | 465 | 9 | 37405 | tss | 0.359380 | 0.7241534 | Not queried | 2.75 | 1.88e-02 | 0.1830867741 |
|  | 11 | 67033594 | 67033801 | PDFSTATSENSEMBLUCSC | 208 | 5 | 7484 | tss;island | 0.007747 | 0.0028289 | Not queried | 2.75 | 5.48e-01 | 0.6179718485 |
|  | 7 | 44279049 | 44279273 | PDFSTATSENSEMBLUCSC | 225 | 4 | 36992 | gene;island | 0.186311 | 0.4695392 | Not queried | 2.75 | 4.86e-01 | 0.5640816327 |
|  | 1 | 59369792 | 59369954 | PDFSTATSENSEMBLUCSC | 163 | 4 | 3321 | island | 0.020760 | 0.0586502 | Not queried | 2.75 | 6.86e-01 | 0.7223604052 |
|  | 5 | 57755923 | 57756026 | PDFSTATSENSEMBLUCSC | 104 | 4 | 32076 | tss | 0.004790 | 0.0113263 | Not queried | 2.75 | 3.43e-01 | 0.4672865596 |
|  | 16 | 29625133 | 29625259 | PDFSTATSENSEMBLUCSC | 127 | 5 | 15166 | tss;island | 0.004801 | 0.0106568 | Not queried | 2.75 | 1.51e-01 | 0.3618000441 |
|  | 20 | 30618874 | 30619244 | PDFSTATSENSEMBLUCSC | 371 | 5 | 25388 | gene;island | 0.319723 | 0.3822562 | Not queried | 2.75 | 1.00e+00 | 1.0000000000 |
|  | 10 | 38691306 | 38691563 | PDFSTATSENSEMBLUCSC | 258 | 4 | 4561 | gene;island | 0.335967 | 0.0346915 | Not queried | 2.74 | 1.14e-01 | 0.3123457953 |
|  | 3 | 185911519 | 185911885 | PDFSTATSENSEMBLUCSC | 367 | 4 | 28072 | gene;island | 0.034889 | 0.3041790 | Not queried | 2.74 | 2.86e-02 | 0.1830867741 |
|  | 13 | 46425866 | 46426263 | PDFSTATSENSEMBLUCSC | 398 | 9 | 11424 | tss | 0.689481 | 0.1173224 | Not queried | 2.74 | 4.89e-01 | 0.5677586445 |
|  | 1 | 35395541 | 35395837 | PDFSTATSENSEMBLUCSC | 297 | 4 | 3030 | island | 0.102812 | 0.0070113 | Not queried | 2.74 | 2.00e-01 | 0.3826488233 |
|  | 10 | 27541213 | 27541418 | PDFSTATSENSEMBLUCSC | 206 | 5 | 5627 | island | 0.982419 | 0.5989667 | Not queried | 2.74 | 3.10e-01 | 0.4672865596 |
|  | 15 | 52404887 | 52405162 | PDFSTATSENSEMBLUCSC | 276 | 8 | 14147 | island | 0.615563 | 0.1819962 | Not queried | 2.74 | 3.82e-01 | 0.5168893301 |
|  | 15 | 51973591 | 51973920 | PDFSTATSENSEMBLUCSC | 330 | 4 | 13350 | gene | 0.409269 | 0.0912680 | Not queried | 2.74 | 2.86e-02 | 0.1830867741 |
|  | 2 | 132202485 | 132202537 | PDFSTATSENSEMBLUCSC | 53 | 5 | 23633 | tss | 0.923644 | 0.6439036 | Not queried | 2.74 | 5.56e-02 | 0.2315877671 |
|  | 13 | 43148017 | 43148436 | PDFSTATSENSEMBLUCSC | 420 | 5 | 11146 | gene;island | 0.022899 | 0.1409463 | Not queried | 2.74 | 3.10e-01 | 0.4672865596 |
|  | 16 | 8768425 | 8768700 | PDFSTATSENSEMBLUCSC | 276 | 5 | 15685 | island | 0.035745 | 0.0065167 | Not queried | 2.74 | 9.52e-02 | 0.3104222821 |
|  | 16 | 1060367 | 1060765 | PDFSTATSENSEMBLUCSC | 399 | 5 | 15506 | island | 0.096046 | 0.1694025 | Not queried | 2.74 | 9.52e-02 | 0.3104222821 |
|  | 1 | 7913667 | 7914143 | PDFSTATSENSEMBLUCSC | 477 | 5 | 1218 | tss | 0.165310 | 0.8959520 | Not queried | 2.74 | 3.17e-02 | 0.1866936846 |
|  | 8 | 10586678 | 10587055 | PDFSTATSENSEMBLUCSC | 378 | 7 | 39199 | gene;island | 0.066544 | 0.4429420 | Not queried | 2.73 | 4.08e-03 | 0.1459191215 |
|  | 1 | 159174001 | 159174162 | PDFSTATSENSEMBLUCSC | 162 | 4 | 2029 | tss | 0.101874 | 0.4821425 | Not queried | 2.73 | 5.71e-02 | 0.2315877671 |
|  | 6 | 101846650 | 101847058 | PDFSTATSENSEMBLUCSC | 409 | 12 | 36370 | island | 0.241133 | 0.6061345 | Not queried | 2.73 | 5.19e-02 | 0.2315877671 |
|  | 1 | 10856466 | 10856739 | PDFSTATSENSEMBLUCSC | 274 | 5 | 2675 | island | 0.134248 | 0.0502371 | Not queried | 2.73 | 6.90e-01 | 0.7223604052 |
|  | 17 | 79920395 | 79920755 | PDFSTATSENSEMBLUCSC | 361 | 5 | 18962 | island | 0.602677 | 0.9091583 | Not queried | 2.73 | 3.10e-01 | 0.4672865596 |
|  | 7 | 158656429 | 158656677 | PDFSTATSENSEMBLUCSC | 249 | 4 | 37380 | gene;island | 0.010792 | 0.0016173 | Not queried | 2.73 | 4.86e-01 | 0.5640816327 |
|  | 20 | 37353032 | 37353096 | PDFSTATSENSEMBLUCSC | 65 | 4 | 25738 | tss | 0.079979 | 0.0161984 | Not queried | 2.73 | 2.00e-01 | 0.3826488233 |
|  | 14 | 104928289 | 104928675 | PDFSTATSENSEMBLUCSC | 387 | 4 | 13205 | island | 0.176725 | 0.0204336 | Not queried | 2.73 | 3.43e-01 | 0.4672865596 |
|  | 2 | 239228826 | 239229182 | PDFSTATSENSEMBLUCSC | 357 | 4 | 23974 | tss | 0.004390 | 0.0552233 | Not queried | 2.73 | 3.43e-01 | 0.4672865596 |
|  | 17 | 46667587 | 46667812 | PDFSTATSENSEMBLUCSC | 226 | 6 | 17462 | tss;island | 0.012609 | 0.0743953 | Not queried | 2.73 | 1.52e-02 | 0.1830867741 |
|  | 22 | 31318349 | 31318546 | PDFSTATSENSEMBLUCSC | 198 | 4 | 26839 | gene | 0.063605 | 0.4078735 | Not queried | 2.73 | 2.00e-01 | 0.3826488233 |
|  | 19 | 48948963 | 48949463 | PDFSTATSENSEMBLUCSC | 501 | 6 | 22217 | island | 0.007990 | 0.0017458 | Not queried | 2.73 | 3.94e-01 | 0.5236526859 |
|  | 11 | 30038615 | 30038797 | PDFSTATSENSEMBLUCSC | 183 | 9 | 7218 | tss;island | 0.116341 | 0.4439297 | Not queried | 2.73 | 1.88e-02 | 0.1830867741 |
|  | 12 | 120763487 | 120763869 | PDFSTATSENSEMBLUCSC | 383 | 5 | 9350 | gene | 0.010707 | 0.0435773 | Not queried | 2.73 | 5.56e-02 | 0.2315877671 |
|  | 15 | 24123232 | 24123421 | PDFSTATSENSEMBLUCSC | 190 | 4 | 13949 | island | 0.333782 | 0.1185486 | Not queried | 2.72 | 2.00e-01 | 0.3826488233 |
|  | 4 | 8136996 | 8137155 | PDFSTATSENSEMBLUCSC | 160 | 5 | 29947 | gene | 0.446253 | 0.5755021 | Not queried | 2.72 | 4.21e-01 | 0.5404252807 |
|  | 14 | 105936300 | 105936715 | PDFSTATSENSEMBLUCSC | 416 | 4 | 12265 | gene;island | 0.755449 | 0.9825063 | Not queried | 2.72 | 2.00e-01 | 0.3826488233 |
|  | 16 | 56554066 | 56554313 | PDFSTATSENSEMBLUCSC | 248 | 5 | 15245 | tss | 0.230353 | 0.9056949 | Not queried | 2.72 | 2.22e-01 | 0.3935758299 |
|  | 1 | 26233332 | 26233709 | PDFSTATSENSEMBLUCSC | 378 | 11 | 1380 | tss;island | 0.419663 | 0.5929551 | Not queried | 2.72 | 1.71e-01 | 0.3826488233 |
|  | 11 | 393938 | 394289 | PDFSTATSENSEMBLUCSC | 352 | 5 | 7847 | island | 0.443709 | 0.6670112 | Not queried | 2.72 | 1.51e-01 | 0.3618000441 |
|  | 1 | 110210265 | 110211023 | PDFSTATSENSEMBLUCSC | 759 | 10 | 3569 | island | 0.016292 | 0.1647880 | Not queried | 2.72 | 1.47e-02 | 0.1830867741 |
|  | 10 | 125651526 | 125651726 | PDFSTATSENSEMBLUCSC | 201 | 6 | 5420 | tss | 0.019852 | 0.4641369 | Not queried | 2.72 | 6.49e-02 | 0.2477578846 |
|  | 19 | 18902117 | 18902221 | PDFSTATSENSEMBLUCSC | 105 | 4 | 20628 | tss | 0.105101 | 0.3071746 | Not queried | 2.72 | 3.43e-01 | 0.4672865596 |
|  | 6 | 31513571 | 31513886 | PDFSTATSENSEMBLUCSC | 316 | 4 | 33897 | gene;tss;island | 0.321999 | 0.7930431 | Not queried | 2.72 | 2.00e-01 | 0.3826488233 |
|  | 16 | 53468112 | 53468205 | PDFSTATSENSEMBLUCSC | 94 | 4 | 15235 | tss | 0.107969 | 0.1996397 | Not queried | 2.72 | 8.86e-01 | 0.8941250130 |
|  | 3 | 21792248 | 21792684 | PDFSTATSENSEMBLUCSC | 437 | 4 | 27700 | gene | 0.042981 | 0.0226073 | Not queried | 2.71 | 6.86e-01 | 0.7223604052 |
|  | 20 | 35169260 | 35169609 | PDFSTATSENSEMBLUCSC | 350 | 5 | 25716 | tss;island | 0.091356 | 0.2278898 | Not queried | 2.71 | 4.21e-01 | 0.5404252807 |
|  | 10 | 23479993 | 23480316 | PDFSTATSENSEMBLUCSC | 324 | 6 | 4987 | tss;island | 0.061272 | 0.3489761 | Not queried | 2.71 | 4.11e-02 | 0.2180389943 |
|  | 6 | 29527870 | 29528119 | PDFSTATSENSEMBLUCSC | 250 | 4 | 34748 | tss | 0.481707 | 0.6903221 | Not queried | 2.71 | 4.86e-01 | 0.5640816327 |
|  | 2 | 176961552 | 176961907 | PDFSTATSENSEMBLUCSC | 356 | 4 | 24892 | island | 0.003977 | 0.0278223 | Not queried | 2.71 | 3.43e-01 | 0.4672865596 |
|  | 11 | 130029961 | 130030459 | PDFSTATSENSEMBLUCSC | 499 | 4 | 6976 | gene;island | 0.015465 | 0.1246543 | Not queried | 2.71 | 3.43e-01 | 0.4672865596 |
|  | 13 | 88324432 | 88324879 | PDFSTATSENSEMBLUCSC | 448 | 5 | 11809 | island | 0.022028 | 0.1153146 | Not queried | 2.71 | 1.59e-02 | 0.1830867741 |
|  | 19 | 54850444 | 54850645 | PDFSTATSENSEMBLUCSC | 202 | 4 | 21059 | tss | 0.672483 | 0.2119643 | Not queried | 2.71 | 1.14e-01 | 0.3123457953 |
|  | 4 | 7637856 | 7638280 | PDFSTATSENSEMBLUCSC | 425 | 6 | 29940 | gene | 0.426288 | 0.5453953 | Not queried | 2.71 | 1.32e-01 | 0.3397986247 |
|  | 20 | 23030102 | 23030533 | PDFSTATSENSEMBLUCSC | 432 | 7 | 26048 | island | 0.010473 | 0.0450709 | Not queried | 2.71 | 1.65e-01 | 0.3826488233 |
|  | 14 | 52455805 | 52456142 | PDFSTATSENSEMBLUCSC | 338 | 4 | 12394 | tss;island | 0.007331 | 0.0059271 | Not queried | 2.71 | 4.86e-01 | 0.5640816327 |
|  | 7 | 154684327 | 154684562 | PDFSTATSENSEMBLUCSC | 236 | 4 | 37304 | gene;island | 0.588014 | 0.7324579 | Not queried | 2.71 | 8.86e-01 | 0.8941250130 |
|  | 7 | 136553088 | 136553327 | PDFSTATSENSEMBLUCSC | 240 | 7 | 37922 | tss;island | 0.004973 | 0.0208930 | Not queried | 2.71 | 3.18e-01 | 0.4672865596 |
|  | 2 | 38300537 | 38300885 | PDFSTATSENSEMBLUCSC | 349 | 4 | 22650 | gene;island | 0.945788 | 0.3753242 | Not queried | 2.70 | 1.14e-01 | 0.3123457953 |
|  | 8 | 19459996 | 19460243 | PDFSTATSENSEMBLUCSC | 248 | 6 | 39221 | gene;island | 0.529596 | 0.9155166 | Not queried | 2.70 | 1.32e-01 | 0.3397986247 |
|  | 10 | 70321243 | 70321959 | PDFSTATSENSEMBLUCSC | 717 | 10 | 4610 | gene;island | 0.022200 | 0.1731352 | Not queried | 2.70 | 4.36e-01 | 0.5589686471 |
|  | 5 | 523430 | 523794 | PDFSTATSENSEMBLUCSC | 365 | 4 | 31511 | gene;island | 0.033056 | 0.0291063 | Not queried | 2.70 | 6.86e-01 | 0.7223604052 |
|  | 1 | 78354053 | 78354406 | PDFSTATSENSEMBLUCSC | 354 | 4 | 3400 | island | 0.120070 | 0.4958310 | Not queried | 2.70 | 2.86e-02 | 0.1830867741 |
|  | 1 | 205819406 | 205819609 | PDFSTATSENSEMBLUCSC | 204 | 4 | 2210 | tss | 0.406962 | 0.0321711 | Not queried | 2.70 | 3.43e-01 | 0.4672865596 |
|  | 1 | 229569608 | 229570288 | PDFSTATSENSEMBLUCSC | 681 | 14 | 4310 | island | 0.068560 | 0.3194111 | ACTA1 (margin: RP5-1068B5.1; ACTA1; RP5-1068B5.3; NUP133) | 2.70 | 3.38e-04 | 0.0499764971 |
|  | 16 | 30485383 | 30485966 | PDFSTATSENSEMBLUCSC | 584 | 6 | 14721 | gene;island | 0.816019 | 0.9133375 | Not queried | 2.70 | 2.40e-01 | 0.4133265075 |
|  | 11 | 123430575 | 123430949 | PDFSTATSENSEMBLUCSC | 375 | 4 | 6950 | gene | 0.016057 | 0.2595990 | Not queried | 2.70 | 3.43e-01 | 0.4672865596 |
|  | 7 | 42267269 | 42267747 | PDFSTATSENSEMBLUCSC | 479 | 7 | 36976 | gene;island | 0.007495 | 0.0583376 | Not queried | 2.70 | 1.75e-02 | 0.1830867741 |
|  | 16 | 55794456 | 55794910 | PDFSTATSENSEMBLUCSC | 455 | 5 | 15938 | island | 0.302379 | 0.2139369 | Not queried | 2.70 | 3.10e-01 | 0.4672865596 |
|  | 17 | 46114615 | 46114933 | PDFSTATSENSEMBLUCSC | 319 | 6 | 17450 | tss | 0.027797 | 0.0824910 | Not queried | 2.70 | 3.10e-01 | 0.4672865596 |
|  | 12 | 70132987 | 70133067 | PDFSTATSENSEMBLUCSC | 81 | 5 | 9213 | gene;tss;island | 0.006055 | 0.0037489 | Not queried | 2.70 | 3.10e-01 | 0.4672865596 |
|  | 11 | 7041752 | 7041987 | PDFSTATSENSEMBLUCSC | 236 | 6 | 6495 | gene | 0.032949 | 0.2007410 | Not queried | 2.69 | 4.33e-03 | 0.1459191215 |
|  | 12 | 46767665 | 46768016 | PDFSTATSENSEMBLUCSC | 352 | 6 | 9658 | tss;island | 0.376921 | 0.7629516 | Not queried | 2.69 | 4.85e-01 | 0.5640816327 |
|  | 17 | 35289351 | 35289875 | PDFSTATSENSEMBLUCSC | 525 | 5 | 18234 | island | 0.034741 | 0.1103071 | Not queried | 2.69 | 3.10e-01 | 0.4672865596 |
|  | 20 | 54919037 | 54919410 | PDFSTATSENSEMBLUCSC | 374 | 6 | 26271 | island | 0.012426 | 0.0733906 | Not queried | 2.69 | 5.89e-01 | 0.6570484697 |
|  | 7 | 111368367 | 111368847 | PDFSTATSENSEMBLUCSC | 481 | 4 | 37188 | gene;island | 0.978743 | 0.8440788 | Not queried | 2.69 | 5.71e-02 | 0.2315877671 |
|  | 3 | 49395574 | 49395745 | PDFSTATSENSEMBLUCSC | 172 | 4 | 27781 | gene | 0.009032 | 0.0535444 | Not queried | 2.69 | 2.00e-01 | 0.3826488233 |
|  | 2 | 51259693 | 51260003 | PDFSTATSENSEMBLUCSC | 311 | 6 | 23349 | tss | 0.013117 | 0.0048512 | Not queried | 2.69 | 2.40e-01 | 0.4133265075 |
|  | 7 | 157453775 | 157454207 | PDFSTATSENSEMBLUCSC | 433 | 8 | 37337 | gene;island | 0.717591 | 0.9562647 | Not queried | 2.69 | 1.04e-02 | 0.1806165940 |
|  | 20 | 62151993 | 62152128 | PDFSTATSENSEMBLUCSC | 136 | 4 | 25896 | tss | 0.001100 | 0.0047149 | Not queried | 2.69 | 2.00e-01 | 0.3826488233 |
|  | 16 | 83171068 | 83171314 | PDFSTATSENSEMBLUCSC | 247 | 4 | 14860 | gene | 0.339118 | 0.0320173 | Not queried | 2.68 | 8.86e-01 | 0.8941250130 |
|  | 2 | 75147006 | 75147288 | PDFSTATSENSEMBLUCSC | 283 | 4 | 24453 | island | 0.158684 | 0.0252248 | Not queried | 2.68 | 1.14e-01 | 0.3123457953 |
|  | 17 | 72442928 | 72443179 | PDFSTATSENSEMBLUCSC | 252 | 5 | 16758 | gene;island | 0.129639 | 0.4876485 | Not queried | 2.68 | 1.59e-02 | 0.1830867741 |
|  | 8 | 26724744 | 26725033 | PDFSTATSENSEMBLUCSC | 290 | 5 | 40177 | island | 0.009539 | 0.0474909 | Not queried | 2.68 | 1.51e-01 | 0.3618000441 |
|  | 12 | 103311462 | 103311761 | PDFSTATSENSEMBLUCSC | 300 | 6 | 9951 | tss | 0.015379 | 0.0560934 | Not queried | 2.68 | 9.31e-02 | 0.3104222821 |
|  | 5 | 72594461 | 72594851 | PDFSTATSENSEMBLUCSC | 391 | 4 | 32868 | island | 0.034780 | 0.2155144 | Not queried | 2.68 | 2.00e-01 | 0.3826488233 |
|  | 13 | 28493913 | 28494161 | PDFSTATSENSEMBLUCSC | 249 | 6 | 11365 | tss | 0.447738 | 0.0738360 | Not queried | 2.68 | 1.80e-01 | 0.3826488233 |
|  | 4 | 21305805 | 21306051 | PDFSTATSENSEMBLUCSC | 247 | 4 | 29965 | gene;tss | 0.079444 | 0.4025088 | Not queried | 2.68 | 3.43e-01 | 0.4672865596 |
|  | 6 | 32005786 | 32006079 | PDFSTATSENSEMBLUCSC | 294 | 4 | 34860 | tss | 0.080074 | 0.4246125 | Not queried | 2.68 | 3.43e-01 | 0.4672865596 |
|  | 19 | 49016564 | 49016934 | PDFSTATSENSEMBLUCSC | 371 | 6 | 20917 | tss;island | 0.214004 | 0.0141683 | Not queried | 2.68 | 3.10e-01 | 0.4672865596 |
|  | 6 | 167263025 | 167263454 | PDFSTATSENSEMBLUCSC | 430 | 6 | 34555 | gene | 0.834707 | 0.3913949 | Not queried | 2.68 | 2.60e-02 | 0.1830867741 |
|  | 6 | 32810551 | 32810833 | PDFSTATSENSEMBLUCSC | 283 | 5 | 34150 | gene;island | 0.089046 | 0.2779585 | Not queried | 2.68 | 2.22e-01 | 0.3935758299 |
|  | 12 | 132923339 | 132923603 | PDFSTATSENSEMBLUCSC | 265 | 4 | 11045 | island | 0.042006 | 0.1285374 | Not queried | 2.68 | 2.00e-01 | 0.3826488233 |
|  | 5 | 114516141 | 114516422 | PDFSTATSENSEMBLUCSC | 282 | 6 | 33019 | island | 0.031740 | 0.0354266 | Not queried | 2.67 | 6.99e-01 | 0.7290267580 |
|  | 10 | 91405265 | 91405300 | PDFSTATSENSEMBLUCSC | 36 | 4 | 5216 | tss;island | 0.116460 | 0.0142254 | Not queried | 2.67 | 2.00e-01 | 0.3826488233 |
|  | 2 | 3699195 | 3699353 | PDFSTATSENSEMBLUCSC | 159 | 4 | 24039 | island | 0.190095 | 0.0393927 | Not queried | 2.67 | 1.14e-01 | 0.3123457953 |
|  | 1 | 156051324 | 156051681 | PDFSTATSENSEMBLUCSC | 358 | 4 | 813 | gene | 0.009125 | 0.0559349 | Not queried | 2.67 | 6.86e-01 | 0.7223604052 |
|  | 6 | 106433792 | 106434623 | PDFSTATSENSEMBLUCSC | 832 | 8 | 36378 | island | 0.156155 | 0.0268787 | Not queried | 2.67 | 4.42e-01 | 0.5640816327 |
|  | 1 | 29240533 | 29240705 | PDFSTATSENSEMBLUCSC | 173 | 4 | 374 | gene;tss;island | 0.672687 | 0.2620181 | Not queried | 2.67 | 4.86e-01 | 0.5640816327 |
|  | 11 | 18230491 | 18230799 | PDFSTATSENSEMBLUCSC | 309 | 7 | 7181 | tss | 0.547286 | 0.8258070 | Not queried | 2.67 | 4.56e-01 | 0.5640816327 |
|  | 11 | 46354803 | 46355283 | PDFSTATSENSEMBLUCSC | 481 | 4 | 6597 | gene | 0.012620 | 0.2538289 | Not queried | 2.67 | 2.00e-01 | 0.3826488233 |
|  | 7 | 54956202 | 54956598 | PDFSTATSENSEMBLUCSC | 397 | 5 | 38501 | island | 0.016709 | 0.1706567 | Not queried | 2.67 | 5.56e-02 | 0.2315877671 |
|  | 16 | 8768488 | 8768700 | PDFSTATSENSEMBLUCSC | 213 | 4 | 14651 | gene | 0.036473 | 0.0082157 | Not queried | 2.67 | 2.86e-02 | 0.1830867741 |
|  | 19 | 34174665 | 34175554 | PDFSTATSENSEMBLUCSC | 890 | 21 | 19974 | gene;island | 0.029174 | 0.4286653 | CHST8 (margin: CHST8) | 2.67 | 9.39e-05 | 0.0229076809 |
|  | 16 | 88038949 | 88039257 | PDFSTATSENSEMBLUCSC | 309 | 4 | 14897 | gene;island | 0.739312 | 0.5072577 | Not queried | 2.67 | 6.86e-01 | 0.7223604052 |
|  | 20 | 327607 | 327811 | PDFSTATSENSEMBLUCSC | 205 | 4 | 25321 | gene;island | 0.015880 | 0.0028658 | Not queried | 2.67 | 2.00e-01 | 0.3826488233 |
|  | 16 | 89390609 | 89390968 | PDFSTATSENSEMBLUCSC | 360 | 4 | 14938 | gene | 0.025052 | 0.3469676 | Not queried | 2.66 | 6.86e-01 | 0.7223604052 |
|  | 19 | 15162515 | 15162971 | PDFSTATSENSEMBLUCSC | 457 | 4 | 20553 | tss | 0.050238 | 0.1819012 | Not queried | 2.66 | 2.00e-01 | 0.3826488233 |
|  | 1 | 229569877 | 229570288 | PDFSTATSENSEMBLUCSC | 412 | 11 | 2311 | tss | 0.062650 | 0.3022816 | Not queried | 2.66 | 6.63e-03 | 0.1589602024 |
|  | 4 | 74734714 | 74735149 | PDFSTATSENSEMBLUCSC | 436 | 10 | 31097 | island | 0.018095 | 0.3467930 | Not queried | 2.66 | 2.88e-02 | 0.1834529198 |
|  | 7 | 27186993 | 27187691 | PDFSTATSENSEMBLUCSC | 699 | 11 | 38349 | island | 0.027389 | 0.2370978 | Not queried | 2.66 | 4.00e-02 | 0.2180389943 |
|  | 4 | 780856 | 781272 | PDFSTATSENSEMBLUCSC | 417 | 4 | 29838 | gene;island | 0.089342 | 0.4721470 | Not queried | 2.66 | 1.14e-01 | 0.3123457953 |
|  | 12 | 110271439 | 110271625 | PDFSTATSENSEMBLUCSC | 187 | 4 | 9996 | tss;island | 0.470323 | 0.1238520 | Not queried | 2.66 | 2.86e-02 | 0.1830867741 |
|  | 10 | 133000130 | 133000471 | PDFSTATSENSEMBLUCSC | 342 | 6 | 4843 | gene | 0.280006 | 0.6512461 | Not queried | 2.66 | 1.80e-01 | 0.3826488233 |
|  | 15 | 96887824 | 96888024 | PDFSTATSENSEMBLUCSC | 201 | 5 | 14489 | island | 0.019642 | 0.0482741 | Not queried | 2.66 | 3.10e-01 | 0.4672865596 |
|  | 7 | 34873912 | 34874196 | PDFSTATSENSEMBLUCSC | 285 | 4 | 36962 | gene | 0.383490 | 0.7952754 | Not queried | 2.66 | 2.00e-01 | 0.3826488233 |
|  | 7 | 106505688 | 106505961 | PDFSTATSENSEMBLUCSC | 274 | 5 | 38825 | island | 0.011752 | 0.0043983 | Not queried | 2.66 | 5.48e-01 | 0.6179718485 |
|  | 6 | 85482570 | 85483055 | PDFSTATSENSEMBLUCSC | 486 | 5 | 36298 | island | 0.321699 | 0.2147192 | Not queried | 2.66 | 4.21e-01 | 0.5404252807 |
|  | 6 | 37616410 | 37616803 | PDFSTATSENSEMBLUCSC | 394 | 5 | 34323 | gene;island | 0.178338 | 0.0757104 | Not queried | 2.65 | 3.10e-01 | 0.4672865596 |
|  | 1 | 3394709 | 3395053 | PDFSTATSENSEMBLUCSC | 345 | 4 | 126 | gene;island | 0.439035 | 0.7344071 | Not queried | 2.65 | 2.00e-01 | 0.3826488233 |
|  | 6 | 28481660 | 28482191 | PDFSTATSENSEMBLUCSC | 532 | 6 | 33634 | gene | 0.117375 | 0.4553744 | Not queried | 2.65 | 3.10e-01 | 0.4672865596 |
|  | 8 | 105479058 | 105479420 | PDFSTATSENSEMBLUCSC | 363 | 7 | 40524 | island | 0.024733 | 0.4072998 | Not queried | 2.65 | 2.62e-02 | 0.1830867741 |
|  | 6 | 32297615 | 32297861 | PDFSTATSENSEMBLUCSC | 247 | 5 | 34101 | gene | 0.825668 | 0.9801835 | Not queried | 2.65 | 9.52e-02 | 0.3104222821 |
|  | 11 | 44087367 | 44087867 | PDFSTATSENSEMBLUCSC | 501 | 10 | 8221 | island | 0.012367 | 0.0717464 | Not queried | 2.65 | 3.55e-02 | 0.2067926195 |
|  | 6 | 31275551 | 31275881 | PDFSTATSENSEMBLUCSC | 331 | 11 | 35762 | island | 0.476808 | 0.6845095 | Not queried | 2.65 | 7.59e-02 | 0.2811835917 |
|  | 17 | 35014412 | 35014676 | PDFSTATSENSEMBLUCSC | 265 | 4 | 18232 | island | 0.026460 | 0.2203379 | Not queried | 2.65 | 6.86e-01 | 0.7223604052 |
|  | 21 | 42688468 | 42688650 | PDFSTATSENSEMBLUCSC | 183 | 5 | 26555 | tss | 0.026122 | 0.0504250 | Not queried | 2.65 | 4.21e-01 | 0.5404252807 |
|  | 19 | 29455596 | 29455913 | PDFSTATSENSEMBLUCSC | 318 | 4 | 20668 | tss | 0.029829 | 0.3270932 | Not queried | 2.65 | 4.86e-01 | 0.5640816327 |
|  | 11 | 8102311 | 8102510 | PDFSTATSENSEMBLUCSC | 200 | 4 | 6496 | gene;tss;island | 0.012618 | 0.0338738 | Not queried | 2.65 | 2.00e-01 | 0.3826488233 |
|  | 5 | 142077712 | 142078020 | PDFSTATSENSEMBLUCSC | 309 | 4 | 32400 | tss | 0.022167 | 0.2579818 | Not queried | 2.65 | 8.86e-01 | 0.8941250130 |
|  | 8 | 80803196 | 80803526 | PDFSTATSENSEMBLUCSC | 331 | 5 | 40410 | island | 0.005640 | 0.0244856 | Not queried | 2.65 | 3.17e-02 | 0.1866936846 |
|  | 1 | 55504649 | 55505189 | PDFSTATSENSEMBLUCSC | 541 | 10 | 1651 | tss | 0.423977 | 0.0515203 | Not queried | 2.64 | 1.65e-01 | 0.3826488233 |
|  | 22 | 36877697 | 36877737 | PDFSTATSENSEMBLUCSC | 41 | 4 | 27108 | tss | 0.000899 | 0.0047143 | Not queried | 2.64 | 3.43e-01 | 0.4672865596 |
|  | 11 | 118842126 | 118842650 | PDFSTATSENSEMBLUCSC | 525 | 10 | 8864 | island | 0.943276 | 0.5611051 | Not queried | 2.64 | 1.23e-01 | 0.3348527312 |
|  | 5 | 154070144 | 154070385 | PDFSTATSENSEMBLUCSC | 242 | 4 | 33335 | island | 0.976051 | 0.7699479 | Not queried | 2.64 | 3.43e-01 | 0.4672865596 |
|  | 4 | 175135048 | 175135997 | PDFSTATSENSEMBLUCSC | 950 | 9 | 31432 | island | 0.020970 | 0.2021104 | Not queried | 2.64 | 1.88e-02 | 0.1830867741 |
|  | 4 | 187646301 | 187646446 | PDFSTATSENSEMBLUCSC | 146 | 4 | 30709 | tss;island | 0.085068 | 0.0107697 | Not queried | 2.64 | 3.43e-01 | 0.4672865596 |
|  | 14 | 69951720 | 69952104 | PDFSTATSENSEMBLUCSC | 385 | 5 | 12125 | gene;island | 0.260396 | 0.5286518 | Not queried | 2.64 | 9.52e-02 | 0.3104222821 |
|  | 11 | 18230491 | 18231021 | PDFSTATSENSEMBLUCSC | 531 | 10 | 8107 | island | 0.542638 | 0.7781336 | Not queried | 2.64 | 2.47e-01 | 0.4242889032 |
|  | 3 | 52828583 | 52828680 | PDFSTATSENSEMBLUCSC | 98 | 4 | 28393 | tss | 0.377794 | 0.6396259 | Not queried | 2.64 | 3.43e-01 | 0.4672865596 |
|  | 12 | 129337674 | 129337910 | PDFSTATSENSEMBLUCSC | 237 | 5 | 10116 | tss;island | 0.025085 | 0.4109776 | Not queried | 2.64 | 2.22e-01 | 0.3935758299 |
|  | 20 | 61866902 | 61867533 | PDFSTATSENSEMBLUCSC | 632 | 10 | 26346 | island | 0.234251 | 0.6747862 | Not queried | 2.64 | 8.92e-02 | 0.3104222821 |
|  | 20 | 61866902 | 61867218 | PDFSTATSENSEMBLUCSC | 317 | 8 | 25891 | tss | 0.234251 | 0.6747862 | Not queried | 2.64 | 1.30e-01 | 0.3397986247 |
|  | 16 | 3079708 | 3079953 | PDFSTATSENSEMBLUCSC | 246 | 4 | 14622 | gene;island | 0.022235 | 0.1763880 | Not queried | 2.64 | 1.14e-01 | 0.3123457953 |
|  | 11 | 2189562 | 2189856 | PDFSTATSENSEMBLUCSC | 295 | 6 | 6452 | gene;island | 0.151828 | 0.4996859 | Not queried | 2.64 | 6.49e-02 | 0.2477578846 |
|  | 3 | 168863860 | 168864313 | PDFSTATSENSEMBLUCSC | 454 | 7 | 28020 | gene;island | 0.019983 | 0.1209651 | Not queried | 2.64 | 1.65e-01 | 0.3826488233 |
|  | 10 | 102279690 | 102279954 | PDFSTATSENSEMBLUCSC | 265 | 7 | 5294 | tss;island | 0.488329 | 0.1328599 | Not queried | 2.63 | 5.35e-01 | 0.6176455124 |
|  | 19 | 18496756 | 18496953 | PDFSTATSENSEMBLUCSC | 198 | 4 | 20618 | tss | 0.259132 | 0.0287304 | Not queried | 2.63 | 1.14e-01 | 0.3123457953 |
|  | 5 | 151304770 | 151305143 | PDFSTATSENSEMBLUCSC | 374 | 6 | 32445 | tss;island | 0.019517 | 0.2324845 | Not queried | 2.63 | 2.40e-01 | 0.4133265075 |
|  | 17 | 37754160 | 37754424 | PDFSTATSENSEMBLUCSC | 265 | 4 | 18277 | island | 0.085326 | 0.5357091 | Not queried | 2.63 | 2.00e-01 | 0.3826488233 |
|  | 9 | 98784231 | 98784441 | PDFSTATSENSEMBLUCSC | 211 | 4 | 40824 | tss;island | 0.026267 | 0.2310009 | Not queried | 2.63 | 4.86e-01 | 0.5640816327 |
|  | 1 | 155100382 | 155100511 | PDFSTATSENSEMBLUCSC | 130 | 4 | 791 | gene | 0.031607 | 0.3116428 | Not queried | 2.63 | 2.00e-01 | 0.3826488233 |
|  | 5 | 180486604 | 180486879 | PDFSTATSENSEMBLUCSC | 276 | 4 | 31949 | gene;island | 0.303824 | 0.0626534 | Not queried | 2.63 | 1.14e-01 | 0.3123457953 |
|  | 14 | 96670628 | 96671104 | PDFSTATSENSEMBLUCSC | 477 | 10 | 12592 | tss | 0.060094 | 0.4271878 | Not queried | 2.63 | 8.92e-02 | 0.3104222821 |
|  | 5 | 142065539 | 142065819 | PDFSTATSENSEMBLUCSC | 281 | 6 | 31843 | gene | 0.034353 | 0.2259026 | Not queried | 2.63 | 2.40e-01 | 0.4133265075 |
|  | 2 | 63274476 | 63275178 | PDFSTATSENSEMBLUCSC | 703 | 7 | 24335 | island | 0.151371 | 0.0343217 | Not queried | 2.62 | 2.59e-01 | 0.4381244265 |
|  | 1 | 118727904 | 118728226 | PDFSTATSENSEMBLUCSC | 323 | 7 | 1875 | tss | 0.054611 | 0.5945480 | Not queried | 2.62 | 7.28e-02 | 0.2713481487 |
|  | 14 | 64319089 | 64319306 | PDFSTATSENSEMBLUCSC | 218 | 4 | 12452 | tss;island | 0.422250 | 0.4365582 | Not queried | 2.62 | 4.86e-01 | 0.5640816327 |
|  | 10 | 50339928 | 50340173 | PDFSTATSENSEMBLUCSC | 246 | 4 | 4582 | gene;island | 0.639948 | 0.9081891 | Not queried | 2.62 | 2.86e-02 | 0.1830867741 |
|  | 2 | 132558939 | 132559214 | PDFSTATSENSEMBLUCSC | 276 | 4 | 22872 | gene | 0.328854 | 0.5087209 | Not queried | 2.62 | 4.86e-01 | 0.5640816327 |
|  | 11 | 63828569 | 63828762 | PDFSTATSENSEMBLUCSC | 194 | 4 | 6661 | gene | 0.011875 | 0.0894900 | Not queried | 2.62 | 1.14e-01 | 0.3123457953 |
|  | 19 | 12946093 | 12946465 | PDFSTATSENSEMBLUCSC | 373 | 8 | 21625 | island | 0.186375 | 0.0413733 | Not queried | 2.62 | 3.82e-01 | 0.5168893301 |
|  | 7 | 45927735 | 45927947 | PDFSTATSENSEMBLUCSC | 213 | 5 | 37586 | tss | 0.093613 | 0.4889669 | Not queried | 2.62 | 4.21e-01 | 0.5404252807 |
|  | 7 | 50800426 | 50800620 | PDFSTATSENSEMBLUCSC | 195 | 4 | 37013 | gene;tss | 0.249887 | 0.6573329 | Not queried | 2.62 | 1.14e-01 | 0.3123457953 |
|  | 10 | 125853676 | 125853893 | PDFSTATSENSEMBLUCSC | 218 | 4 | 6225 | island | 0.094549 | 0.3784362 | Not queried | 2.62 | 1.14e-01 | 0.3123457953 |
|  | 6 | 32549139 | 32549631 | PDFSTATSENSEMBLUCSC | 493 | 5 | 34121 | gene;island | 0.649069 | 0.9371347 | Not queried | 2.62 | 3.10e-01 | 0.4672865596 |
|  | 11 | 2193041 | 2193293 | PDFSTATSENSEMBLUCSC | 253 | 4 | 7063 | tss | 0.220433 | 0.6300158 | Not queried | 2.62 | 3.43e-01 | 0.4672865596 |
|  | 17 | 32906394 | 32906622 | PDFSTATSENSEMBLUCSC | 229 | 4 | 17260 | tss | 0.004474 | 0.0176603 | Not queried | 2.62 | 2.00e-01 | 0.3826488233 |
|  | 6 | 33160847 | 33161589 | PDFSTATSENSEMBLUCSC | 743 | 22 | 34890 | tss;island | 0.093490 | 0.4242105 | Not queried | 2.61 | 1.41e-02 | 0.1830867741 |
|  | 12 | 64784483 | 64784686 | PDFSTATSENSEMBLUCSC | 204 | 5 | 9841 | tss;island | 0.019824 | 0.1090484 | Not queried | 2.61 | 9.52e-02 | 0.3104222821 |
|  | 19 | 49699879 | 49700021 | PDFSTATSENSEMBLUCSC | 143 | 4 | 20147 | gene;island | 0.565025 | 0.8611668 | Not queried | 2.61 | 2.86e-02 | 0.1830867741 |
|  | 5 | 168728149 | 168728270 | PDFSTATSENSEMBLUCSC | 122 | 4 | 32480 | tss | 0.080306 | 0.0079159 | Not queried | 2.61 | 2.00e-01 | 0.3826488233 |
|  | 12 | 103344336 | 103344685 | PDFSTATSENSEMBLUCSC | 350 | 5 | 10727 | island | 0.011664 | 0.1041506 | Not queried | 2.61 | 9.52e-02 | 0.3104222821 |
|  | 8 | 1960217 | 1960466 | PDFSTATSENSEMBLUCSC | 250 | 4 | 40046 | island | 0.227819 | 0.6685937 | Not queried | 2.61 | 2.00e-01 | 0.3826488233 |
|  | 19 | 37825307 | 37825446 | PDFSTATSENSEMBLUCSC | 140 | 6 | 20736 | tss;island | 0.415411 | 0.2107143 | Not queried | 2.61 | 3.10e-01 | 0.4672865596 |
|  | 10 | 29698152 | 29698470 | PDFSTATSENSEMBLUCSC | 319 | 8 | 5008 | tss;island | 0.011361 | 0.0050132 | Not queried | 2.61 | 3.82e-01 | 0.5168893301 |
|  | 11 | 2160540 | 2161586 | PDFSTATSENSEMBLUCSC | 1047 | 32 | 6447 | gene;tss;island | 0.026258 | 0.1457218 | IGF2-AS; IGF2; INS-IGF2 (margin: IGF2-AS; IGF2; AC132217.4; INS-IGF2; MIR483) | 2.61 | 5.59e-06 | 0.0028957612 |
|  | 16 | 1070957 | 1071402 | PDFSTATSENSEMBLUCSC | 446 | 5 | 15508 | island | 0.417148 | 0.5679185 | Not queried | 2.61 | 4.21e-01 | 0.5404252807 |
|  | 3 | 121264538 | 121264849 | PDFSTATSENSEMBLUCSC | 312 | 5 | 27912 | gene | 0.004760 | 0.0106002 | Not queried | 2.61 | 3.10e-01 | 0.4672865596 |
|  | 11 | 94501303 | 94501718 | PDFSTATSENSEMBLUCSC | 416 | 9 | 8742 | island | 0.027385 | 0.0058885 | Not queried | 2.61 | 4.00e-02 | 0.2180389943 |
|  | 11 | 27015656 | 27015991 | PDFSTATSENSEMBLUCSC | 336 | 5 | 6541 | gene | 0.120775 | 0.4554488 | Not queried | 2.61 | 2.22e-01 | 0.3935758299 |
|  | 5 | 140305947 | 140306249 | PDFSTATSENSEMBLUCSC | 303 | 8 | 32357 | tss | 0.121438 | 0.0561376 | Not queried | 2.60 | 1.95e-01 | 0.3826488233 |
|  | 11 | 70257035 | 70257280 | PDFSTATSENSEMBLUCSC | 246 | 4 | 6789 | gene;island | 0.629318 | 0.0752362 | Not queried | 2.60 | 4.86e-01 | 0.5640816327 |
|  | 3 | 147124363 | 147124827 | PDFSTATSENSEMBLUCSC | 465 | 6 | 28651 | tss;island | 0.008399 | 0.0478803 | Not queried | 2.60 | 4.11e-02 | 0.2180389943 |
|  | 3 | 64670459 | 64670640 | PDFSTATSENSEMBLUCSC | 182 | 4 | 27850 | gene;island | 0.442801 | 0.2592019 | Not queried | 2.60 | 6.86e-01 | 0.7223604052 |
|  | 6 | 149285724 | 149285963 | PDFSTATSENSEMBLUCSC | 240 | 4 | 34503 | gene | 0.944393 | 0.6258820 | Not queried | 2.60 | 1.14e-01 | 0.3123457953 |
|  | 10 | 1517083 | 1517340 | PDFSTATSENSEMBLUCSC | 258 | 5 | 4467 | gene;island | 0.139948 | 0.3999584 | Not queried | 2.60 | 3.10e-01 | 0.4672865596 |
|  | 5 | 36606102 | 36606446 | PDFSTATSENSEMBLUCSC | 345 | 5 | 32019 | tss | 0.015053 | 0.0034310 | Not queried | 2.59 | 3.10e-01 | 0.4672865596 |
|  | 2 | 45159894 | 45160554 | PDFSTATSENSEMBLUCSC | 661 | 8 | 24260 | island | 0.036907 | 0.4659900 | Not queried | 2.59 | 6.50e-02 | 0.2477578846 |
|  | 19 | 11530021 | 11530065 | PDFSTATSENSEMBLUCSC | 45 | 4 | 20471 | tss | 0.005982 | 0.0409693 | Not queried | 2.59 | 1.14e-01 | 0.3123457953 |
|  | 2 | 182549353 | 182549604 | PDFSTATSENSEMBLUCSC | 252 | 5 | 24939 | island | 0.096290 | 0.4937362 | Not queried | 2.59 | 6.90e-01 | 0.7223604052 |
|  | 11 | 74442547 | 74442900 | PDFSTATSENSEMBLUCSC | 354 | 4 | 7561 | tss;island | 0.029475 | 0.1355857 | Not queried | 2.59 | 3.43e-01 | 0.4672865596 |
|  | 1 | 247171234 | 247171838 | PDFSTATSENSEMBLUCSC | 605 | 10 | 4411 | island | 0.054074 | 0.0148760 | Not queried | 2.59 | 7.53e-02 | 0.2795801356 |
|  | 1 | 110672769 | 110673319 | PDFSTATSENSEMBLUCSC | 551 | 6 | 3575 | island | 0.058464 | 0.2687691 | Not queried | 2.59 | 1.80e-01 | 0.3826488233 |
|  | 6 | 70991156 | 70991283 | PDFSTATSENSEMBLUCSC | 128 | 4 | 34382 | gene;island | 0.375437 | 0.6584076 | Not queried | 2.59 | 4.86e-01 | 0.5640816327 |
|  | 7 | 1280040 | 1280640 | PDFSTATSENSEMBLUCSC | 601 | 5 | 38094 | island | 0.014360 | 0.1141960 | Not queried | 2.58 | 2.22e-01 | 0.3935758299 |
|  | 14 | 69726197 | 69726620 | PDFSTATSENSEMBLUCSC | 424 | 8 | 12479 | tss | 0.274038 | 0.0357326 | Not queried | 2.58 | 3.79e-02 | 0.2121545571 |
|  | 3 | 126242864 | 126243125 | PDFSTATSENSEMBLUCSC | 262 | 7 | 28561 | tss | 0.162355 | 0.0332051 | Not queried | 2.58 | 5.30e-02 | 0.2315877671 |
|  | 18 | 13801010 | 13801271 | PDFSTATSENSEMBLUCSC | 262 | 4 | 19411 | island | 0.023820 | 0.0448497 | Not queried | 2.58 | 4.86e-01 | 0.5640816327 |
|  | 12 | 54393966 | 54394212 | PDFSTATSENSEMBLUCSC | 247 | 5 | 9157 | gene;island | 0.029785 | 0.1950794 | Not queried | 2.58 | 1.51e-01 | 0.3618000441 |
|  | 3 | 46933782 | 46933972 | PDFSTATSENSEMBLUCSC | 191 | 4 | 27757 | gene;island | 0.024316 | 0.0453671 | Not queried | 2.58 | 3.43e-01 | 0.4672865596 |
|  | 20 | 35972924 | 35973098 | PDFSTATSENSEMBLUCSC | 175 | 5 | 25726 | tss;island | 0.171898 | 0.4578843 | Not queried | 2.58 | 2.22e-01 | 0.3935758299 |
|  | 14 | 105104840 | 105105198 | PDFSTATSENSEMBLUCSC | 359 | 4 | 13209 | island | 0.369464 | 0.7762412 | Not queried | 2.58 | 3.43e-01 | 0.4672865596 |
|  | 12 | 93836052 | 93836653 | PDFSTATSENSEMBLUCSC | 602 | 9 | 9915 | tss | 0.004833 | 0.0034309 | Not queried | 2.58 | 2.22e-01 | 0.3935758299 |
|  | 16 | 68270129 | 68270387 | PDFSTATSENSEMBLUCSC | 259 | 5 | 16041 | island | 0.018694 | 0.2096431 | Not queried | 2.58 | 3.17e-02 | 0.1866936846 |
|  | 14 | 101525784 | 101526140 | PDFSTATSENSEMBLUCSC | 357 | 6 | 12630 | tss | 0.199593 | 0.5623148 | Not queried | 2.58 | 4.11e-02 | 0.2180389943 |
|  | 8 | 124219425 | 124219725 | PDFSTATSENSEMBLUCSC | 301 | 4 | 39419 | gene;island | 0.562113 | 0.8482687 | Not queried | 2.58 | 2.00e-01 | 0.3826488233 |
|  | 2 | 63276478 | 63277327 | PDFSTATSENSEMBLUCSC | 850 | 21 | 23386 | tss;island | 0.230741 | 0.0236931 | Not queried | 2.57 | 4.90e-02 | 0.2315877671 |
|  | 1 | 119543883 | 119544329 | PDFSTATSENSEMBLUCSC | 447 | 5 | 3661 | island | 0.089517 | 0.0697744 | Not queried | 2.57 | 6.90e-01 | 0.7223604052 |
|  | 6 | 30458519 | 30458730 | PDFSTATSENSEMBLUCSC | 212 | 4 | 33769 | gene;island | 0.032566 | 0.0752740 | Not queried | 2.57 | 4.86e-01 | 0.5640816327 |
|  | 7 | 157982161 | 157982589 | PDFSTATSENSEMBLUCSC | 429 | 5 | 37361 | gene;island | 0.050835 | 0.2413452 | Not queried | 2.57 | 9.52e-02 | 0.3104222821 |
|  | 13 | 79170923 | 79171230 | PDFSTATSENSEMBLUCSC | 308 | 4 | 11793 | island | 0.156723 | 0.0616192 | Not queried | 2.57 | 2.00e-01 | 0.3826488233 |
|  | 6 | 29631227 | 29631447 | PDFSTATSENSEMBLUCSC | 221 | 4 | 33685 | gene | 0.407657 | 0.7034022 | Not queried | 2.57 | 2.00e-01 | 0.3826488233 |
|  | 15 | 68724690 | 68725002 | PDFSTATSENSEMBLUCSC | 313 | 4 | 13759 | tss;island | 0.056614 | 0.2550204 | Not queried | 2.57 | 3.43e-01 | 0.4672865596 |
|  | 15 | 68851385 | 68851827 | PDFSTATSENSEMBLUCSC | 443 | 5 | 14259 | island | 0.007837 | 0.0063835 | Not queried | 2.57 | 3.10e-01 | 0.4672865596 |
|  | 11 | 320732 | 320889 | PDFSTATSENSEMBLUCSC | 158 | 4 | 6370 | gene | 0.050066 | 0.3162403 | Not queried | 2.57 | 1.14e-01 | 0.3123457953 |
|  | 8 | 67344553 | 67344665 | PDFSTATSENSEMBLUCSC | 113 | 6 | 39742 | tss | 0.011661 | 0.0852933 | Not queried | 2.57 | 1.32e-01 | 0.3397986247 |
|  | 7 | 24797102 | 24797884 | PDFSTATSENSEMBLUCSC | 783 | 11 | 37478 | tss | 0.176079 | 0.8381729 | Not queried | 2.57 | 2.43e-01 | 0.4165716700 |
|  | 12 | 50344211 | 50344475 | PDFSTATSENSEMBLUCSC | 265 | 5 | 9698 | tss | 0.108656 | 0.4196181 | Not queried | 2.57 | 5.56e-02 | 0.2315877671 |
|  | 17 | 49243257 | 49243640 | PDFSTATSENSEMBLUCSC | 384 | 5 | 16695 | gene;tss;island | 0.012667 | 0.0184425 | Not queried | 2.57 | 5.48e-01 | 0.6179718485 |
|  | 3 | 30647378 | 30647988 | PDFSTATSENSEMBLUCSC | 611 | 11 | 28200 | tss | 0.009934 | 0.0583963 | Not queried | 2.57 | 5.57e-02 | 0.2315877671 |
|  | 8 | 23385905 | 23386187 | PDFSTATSENSEMBLUCSC | 283 | 5 | 39602 | tss;island | 0.004843 | 0.4735073 | Not queried | 2.57 | 1.51e-01 | 0.3618000441 |
|  | 2 | 175205716 | 175205852 | PDFSTATSENSEMBLUCSC | 137 | 4 | 24875 | island | 0.031393 | 0.1844833 | Not queried | 2.57 | 3.43e-01 | 0.4672865596 |
|  | 1 | 3100143 | 3100497 | PDFSTATSENSEMBLUCSC | 355 | 4 | 106 | gene;island | 0.073123 | 0.5601795 | Not queried | 2.57 | 1.14e-01 | 0.3123457953 |
|  | 19 | 14229237 | 14229581 | PDFSTATSENSEMBLUCSC | 345 | 4 | 20540 | tss;island | 0.035709 | 0.2267326 | Not queried | 2.56 | 2.00e-01 | 0.3826488233 |
|  | 11 | 64059307 | 64059594 | PDFSTATSENSEMBLUCSC | 288 | 4 | 6678 | gene;island | 0.003536 | 0.0054689 | Not queried | 2.56 | 2.00e-01 | 0.3826488233 |
|  | 3 | 193922037 | 193922406 | PDFSTATSENSEMBLUCSC | 370 | 5 | 29784 | island | 0.050964 | 0.4858026 | Not queried | 2.56 | 5.48e-01 | 0.6179718485 |
|  | 5 | 140718283 | 140718405 | PDFSTATSENSEMBLUCSC | 123 | 4 | 31816 | gene;island | 0.012394 | 0.0646590 | Not queried | 2.56 | 4.86e-01 | 0.5640816327 |
|  | 5 | 171057072 | 171057339 | PDFSTATSENSEMBLUCSC | 268 | 5 | 33383 | island | 0.314930 | 0.1177605 | Not queried | 2.56 | 3.10e-01 | 0.4672865596 |
|  | 3 | 147142102 | 147142415 | PDFSTATSENSEMBLUCSC | 314 | 5 | 29576 | island | 0.036478 | 0.2752495 | Not queried | 2.56 | 4.21e-01 | 0.5404252807 |
|  | 16 | 51187388 | 51187807 | PDFSTATSENSEMBLUCSC | 420 | 5 | 15920 | island | 0.126862 | 0.0519300 | Not queried | 2.56 | 1.00e+00 | 1.0000000000 |
|  | 6 | 30458998 | 30459595 | PDFSTATSENSEMBLUCSC | 598 | 8 | 33770 | gene;island | 0.077496 | 0.5664437 | Not queried | 2.56 | 3.79e-02 | 0.2121545571 |
|  | 3 | 46506104 | 46506374 | PDFSTATSENSEMBLUCSC | 271 | 4 | 27749 | gene | 0.240380 | 0.0190717 | Not queried | 2.56 | 2.00e-01 | 0.3826488233 |
|  | 11 | 320732 | 320956 | PDFSTATSENSEMBLUCSC | 225 | 7 | 7842 | island | 0.028990 | 0.2756238 | Not queried | 2.56 | 9.73e-02 | 0.3123457953 |
|  | 2 | 55277876 | 55278269 | PDFSTATSENSEMBLUCSC | 394 | 4 | 23358 | tss;island | 0.008082 | 0.0033385 | Not queried | 2.56 | 2.00e-01 | 0.3826488233 |
|  | 13 | 111089028 | 111089384 | PDFSTATSENSEMBLUCSC | 357 | 4 | 11229 | gene;island | 0.524251 | 0.8644930 | Not queried | 2.55 | 2.00e-01 | 0.3826488233 |
|  | 19 | 15122135 | 15122224 | PDFSTATSENSEMBLUCSC | 90 | 4 | 19898 | gene;island | 0.026975 | 0.1113256 | Not queried | 2.55 | 2.86e-02 | 0.1830867741 |
|  | 10 | 95326801 | 95327325 | PDFSTATSENSEMBLUCSC | 525 | 6 | 4682 | gene;island | 0.165107 | 0.0466165 | Not queried | 2.55 | 1.32e-01 | 0.3397986247 |
|  | 4 | 4387697 | 4387917 | PDFSTATSENSEMBLUCSC | 221 | 6 | 30267 | tss;island | 0.143151 | 0.0130273 | Not queried | 2.55 | 1.32e-01 | 0.3397986247 |
|  | 16 | 85878940 | 85879145 | PDFSTATSENSEMBLUCSC | 206 | 4 | 16166 | island | 0.650906 | 0.8498712 | Not queried | 2.55 | 3.43e-01 | 0.4672865596 |
|  | 22 | 44287876 | 44288155 | PDFSTATSENSEMBLUCSC | 280 | 6 | 27206 | tss | 0.019935 | 0.2105981 | Not queried | 2.55 | 2.60e-02 | 0.1830867741 |
|  | 8 | 21771252 | 21771668 | PDFSTATSENSEMBLUCSC | 417 | 6 | 39577 | tss | 0.003702 | 0.0234736 | Not queried | 2.55 | 4.11e-02 | 0.2180389943 |
|  | 12 | 678853 | 679287 | PDFSTATSENSEMBLUCSC | 435 | 4 | 8996 | gene;island | 0.009031 | 0.0195039 | Not queried | 2.55 | 4.86e-01 | 0.5640816327 |
|  | 17 | 78417789 | 78418076 | PDFSTATSENSEMBLUCSC | 288 | 6 | 18876 | island | 0.223605 | 0.6615421 | Not queried | 2.55 | 8.66e-03 | 0.1595382395 |
|  | 13 | 40746370 | 40746648 | PDFSTATSENSEMBLUCSC | 279 | 4 | 11685 | island | 0.386967 | 0.8056389 | Not queried | 2.55 | 1.14e-01 | 0.3123457953 |
|  | 15 | 75639163 | 75639433 | PDFSTATSENSEMBLUCSC | 271 | 4 | 14328 | island | 0.079444 | 0.4186265 | Not queried | 2.55 | 3.43e-01 | 0.4672865596 |
|  | 5 | 158689508 | 158689727 | PDFSTATSENSEMBLUCSC | 220 | 4 | 32461 | tss;island | 0.169879 | 0.6508837 | Not queried | 2.55 | 3.43e-01 | 0.4672865596 |
|  | 11 | 120973085 | 120973274 | PDFSTATSENSEMBLUCSC | 190 | 4 | 7766 | tss | 0.368009 | 0.7408096 | Not queried | 2.55 | 3.43e-01 | 0.4672865596 |
|  | 17 | 11501999 | 11502254 | PDFSTATSENSEMBLUCSC | 256 | 4 | 16445 | gene;island | 0.037860 | 0.2753104 | Not queried | 2.54 | 2.00e-01 | 0.3826488233 |
|  | 10 | 102756795 | 102756923 | PDFSTATSENSEMBLUCSC | 129 | 6 | 5301 | tss | 0.003667 | 0.0055906 | Not queried | 2.54 | 2.40e-01 | 0.4133265075 |
|  | 1 | 221052350 | 221052729 | PDFSTATSENSEMBLUCSC | 380 | 7 | 2264 | tss | 0.003116 | 0.0211087 | Not queried | 2.54 | 5.30e-02 | 0.2315877671 |
|  | 7 | 126893237 | 126893362 | PDFSTATSENSEMBLUCSC | 126 | 4 | 37875 | tss;island | 0.067972 | 0.1003687 | Not queried | 2.54 | 6.86e-01 | 0.7223604052 |
|  | 3 | 10149803 | 10150139 | PDFSTATSENSEMBLUCSC | 337 | 9 | 28871 | island | 0.968652 | 0.9123461 | Not queried | 2.54 | 6.25e-02 | 0.2477578846 |
|  | 3 | 10149918 | 10150139 | PDFSTATSENSEMBLUCSC | 222 | 7 | 28142 | tss | 0.954410 | 0.9123461 | Not queried | 2.54 | 1.28e-01 | 0.3397986247 |
|  | 15 | 100533202 | 100533336 | PDFSTATSENSEMBLUCSC | 135 | 4 | 13504 | gene;island | 0.046239 | 0.3359695 | Not queried | 2.54 | 2.00e-01 | 0.3826488233 |
|  | 6 | 85474028 | 85474595 | PDFSTATSENSEMBLUCSC | 568 | 10 | 35101 | tss;island | 0.463522 | 0.0601850 | Not queried | 2.54 | 5.24e-02 | 0.2315877671 |
|  | 12 | 1725788 | 1726076 | PDFSTATSENSEMBLUCSC | 289 | 6 | 9485 | tss | 0.112196 | 0.6017566 | Not queried | 2.54 | 3.10e-01 | 0.4672865596 |
|  | 1 | 155265026 | 155265378 | PDFSTATSENSEMBLUCSC | 353 | 4 | 800 | gene;island | 0.491717 | 0.1030736 | Not queried | 2.54 | 3.43e-01 | 0.4672865596 |
|  | 17 | 9478950 | 9479224 | PDFSTATSENSEMBLUCSC | 275 | 5 | 16436 | gene | 0.003881 | 0.0094686 | Not queried | 2.54 | 2.22e-01 | 0.3935758299 |
|  | 10 | 126694147 | 126694517 | PDFSTATSENSEMBLUCSC | 371 | 4 | 4810 | gene | 0.436017 | 0.6500064 | Not queried | 2.54 | 2.00e-01 | 0.3826488233 |
|  | 17 | 11501503 | 11501663 | PDFSTATSENSEMBLUCSC | 161 | 8 | 17137 | tss;island | 0.093546 | 0.2188429 | Not queried | 2.54 | 1.61e-01 | 0.3814043454 |
|  | 2 | 63283806 | 63284132 | PDFSTATSENSEMBLUCSC | 327 | 5 | 22705 | gene;island | 0.007541 | 0.0058027 | Not queried | 2.53 | 8.41e-01 | 0.8635565145 |
|  | 14 | 103388656 | 103389022 | PDFSTATSENSEMBLUCSC | 367 | 6 | 13180 | island | 0.610187 | 0.8982798 | Not queried | 2.53 | 1.80e-01 | 0.3826488233 |
|  | 14 | 103388656 | 103388859 | PDFSTATSENSEMBLUCSC | 204 | 4 | 12647 | tss | 0.725962 | 0.9010361 | Not queried | 2.53 | 3.43e-01 | 0.4672865596 |
|  | 2 | 45029543 | 45030072 | PDFSTATSENSEMBLUCSC | 530 | 6 | 24257 | island | 0.039261 | 0.2692720 | Not queried | 2.53 | 9.31e-02 | 0.3104222821 |
|  | 12 | 106751060 | 106751428 | PDFSTATSENSEMBLUCSC | 369 | 7 | 9968 | tss;island | 0.002288 | 0.0037429 | Not queried | 2.53 | 2.59e-01 | 0.4381244265 |
|  | 19 | 34918900 | 34919411 | PDFSTATSENSEMBLUCSC | 512 | 8 | 21886 | island | 0.002442 | 0.0028556 | Not queried | 2.53 | 6.45e-01 | 0.7177393779 |
|  | 16 | 67427183 | 67427391 | PDFSTATSENSEMBLUCSC | 209 | 4 | 14798 | gene;island | 0.064904 | 0.3693196 | Not queried | 2.53 | 1.14e-01 | 0.3123457953 |
|  | 13 | 113633379 | 113634042 | PDFSTATSENSEMBLUCSC | 664 | 8 | 11263 | gene;island | 0.039385 | 0.4668478 | Not queried | 2.53 | 4.99e-02 | 0.2315877671 |
|  | 17 | 53342593 | 53342740 | PDFSTATSENSEMBLUCSC | 148 | 4 | 16700 | gene;island | 0.020060 | 0.0092028 | Not queried | 2.53 | 6.86e-01 | 0.7223604052 |
|  | 13 | 58204028 | 58204379 | PDFSTATSENSEMBLUCSC | 352 | 7 | 11765 | island | 0.052198 | 0.3784814 | Not queried | 2.53 | 1.75e-02 | 0.1830867741 |
|  | 11 | 64051699 | 64051814 | PDFSTATSENSEMBLUCSC | 116 | 6 | 6673 | gene;tss;island | 0.060043 | 0.0136480 | Not queried | 2.53 | 1.80e-01 | 0.3826488233 |
|  | 8 | 65292217 | 65292498 | PDFSTATSENSEMBLUCSC | 282 | 4 | 40343 | island | 0.523981 | 0.1036686 | Not queried | 2.53 | 8.86e-01 | 0.8941250130 |
|  | 11 | 14993378 | 14993818 | PDFSTATSENSEMBLUCSC | 441 | 6 | 6520 | gene | 0.097303 | 0.4085192 | Not queried | 2.53 | 9.31e-02 | 0.3104222821 |
|  | 16 | 49316845 | 49317169 | PDFSTATSENSEMBLUCSC | 325 | 4 | 15226 | tss | 0.017759 | 0.2232500 | Not queried | 2.53 | 2.00e-01 | 0.3826488233 |
|  | 2 | 105484357 | 105484823 | PDFSTATSENSEMBLUCSC | 467 | 4 | 24580 | island | 0.488615 | 0.1408323 | Not queried | 2.53 | 3.43e-01 | 0.4672865596 |
|  | 18 | 21977708 | 21978221 | PDFSTATSENSEMBLUCSC | 514 | 9 | 19436 | island | 0.041679 | 0.0109703 | Not queried | 2.53 | 3.15e-02 | 0.1866936846 |
|  | 17 | 16256937 | 16257223 | PDFSTATSENSEMBLUCSC | 287 | 9 | 17156 | tss;island | 0.316072 | 0.0244363 | Not queried | 2.52 | 5.03e-02 | 0.2315877671 |
|  | 1 | 40204285 | 40204479 | PDFSTATSENSEMBLUCSC | 195 | 4 | 1528 | tss | 0.012815 | 0.0552693 | Not queried | 2.52 | 3.43e-01 | 0.4672865596 |
|  | 12 | 6421437 | 6421806 | PDFSTATSENSEMBLUCSC | 370 | 6 | 9024 | gene;tss;island | 0.595732 | 0.8786544 | Not queried | 2.52 | 3.94e-01 | 0.5236526859 |
|  | 8 | 144820683 | 144820908 | PDFSTATSENSEMBLUCSC | 226 | 4 | 40685 | island | 0.253137 | 0.6071820 | Not queried | 2.52 | 2.00e-01 | 0.3826488233 |
|  | 15 | 88798331 | 88798666 | PDFSTATSENSEMBLUCSC | 336 | 4 | 13464 | gene;island | 0.015429 | 0.2349007 | Not queried | 2.52 | 3.43e-01 | 0.4672865596 |
|  | 12 | 79258313 | 79258438 | PDFSTATSENSEMBLUCSC | 126 | 4 | 9225 | gene;tss | 0.007463 | 0.0437722 | Not queried | 2.52 | 4.86e-01 | 0.5640816327 |
|  | 7 | 1018384 | 1018603 | PDFSTATSENSEMBLUCSC | 220 | 4 | 38062 | island | 0.787156 | 0.9650794 | Not queried | 2.52 | 1.14e-01 | 0.3123457953 |
|  | 11 | 134176598 | 134176888 | PDFSTATSENSEMBLUCSC | 291 | 5 | 6996 | gene | 0.179390 | 0.6400339 | Not queried | 2.52 | 3.10e-01 | 0.4672865596 |
|  | 10 | 124895638 | 124895886 | PDFSTATSENSEMBLUCSC | 249 | 4 | 4802 | gene | 0.014682 | 0.0035043 | Not queried | 2.52 | 2.00e-01 | 0.3826488233 |
|  | 1 | 152487844 | 152488368 | PDFSTATSENSEMBLUCSC | 525 | 8 | 758 | gene;island | 0.097672 | 0.4485084 | Not queried | 2.52 | 4.42e-01 | 0.5640816327 |
|  | 6 | 26183508 | 26183899 | PDFSTATSENSEMBLUCSC | 392 | 9 | 34691 | tss | 0.019488 | 0.0182204 | Not queried | 2.52 | 2.58e-01 | 0.4381244265 |
|  | 12 | 69753119 | 69753455 | PDFSTATSENSEMBLUCSC | 337 | 5 | 9860 | tss | 0.003325 | 0.0019630 | Not queried | 2.51 | 5.48e-01 | 0.6179718485 |
|  | 8 | 68864012 | 68864549 | PDFSTATSENSEMBLUCSC | 538 | 9 | 39751 | tss;island | 0.024682 | 0.1646819 | Not queried | 2.51 | 1.85e-03 | 0.0934794372 |
|  | 6 | 10886946 | 10887047 | PDFSTATSENSEMBLUCSC | 102 | 7 | 34633 | tss | 0.021164 | 0.0037025 | Not queried | 2.51 | 3.79e-02 | 0.2121545571 |
|  | 22 | 37252282 | 37252652 | PDFSTATSENSEMBLUCSC | 371 | 4 | 27460 | island | 0.012910 | 0.0412111 | Not queried | 2.51 | 3.43e-01 | 0.4672865596 |
|  | 11 | 57519282 | 57519659 | PDFSTATSENSEMBLUCSC | 378 | 8 | 7310 | tss;island | 0.167328 | 0.6865073 | Not queried | 2.51 | 2.34e-01 | 0.4128374386 |
|  | 11 | 46366643 | 46366884 | PDFSTATSENSEMBLUCSC | 242 | 6 | 7276 | tss | 0.076800 | 0.4784348 | Not queried | 2.51 | 4.11e-02 | 0.2180389943 |
|  | 1 | 205290869 | 205291215 | PDFSTATSENSEMBLUCSC | 347 | 5 | 4126 | island | 0.048680 | 0.2106659 | Not queried | 2.51 | 9.52e-02 | 0.3104222821 |
|  | 8 | 1792023 | 1792249 | PDFSTATSENSEMBLUCSC | 227 | 4 | 39178 | gene | 0.441138 | 0.8302538 | Not queried | 2.50 | 1.14e-01 | 0.3123457953 |
|  | 15 | 96873850 | 96874052 | PDFSTATSENSEMBLUCSC | 203 | 9 | 13492 | gene;tss;island | 0.060289 | 0.2298941 | Not queried | 2.50 | 7.70e-02 | 0.2825346647 |
|  | 6 | 39759941 | 39760092 | PDFSTATSENSEMBLUCSC | 152 | 4 | 34958 | tss;island | 0.146395 | 0.0268831 | Not queried | 2.50 | 5.71e-02 | 0.2315877671 |
|  | 11 | 64115989 | 64116279 | PDFSTATSENSEMBLUCSC | 291 | 4 | 6685 | gene;island | 0.768955 | 0.9692663 | Not queried | 2.50 | 5.71e-02 | 0.2315877671 |
|  | 16 | 1146194 | 1146545 | PDFSTATSENSEMBLUCSC | 352 | 5 | 15516 | island | 0.638660 | 0.8113193 | Not queried | 2.50 | 5.56e-02 | 0.2315877671 |
|  | 5 | 3595728 | 3595977 | PDFSTATSENSEMBLUCSC | 250 | 4 | 31977 | tss;island | 0.496714 | 0.1095521 | Not queried | 2.50 | 6.86e-01 | 0.7223604052 |
|  | 6 | 7911240 | 7911480 | PDFSTATSENSEMBLUCSC | 241 | 4 | 34617 | tss;island | 0.002912 | 0.0178085 | Not queried | 2.50 | 4.86e-01 | 0.5640816327 |
|  | 1 | 221052350 | 221052886 | PDFSTATSENSEMBLUCSC | 537 | 8 | 4221 | island | 0.003058 | 0.0189370 | Not queried | 2.50 | 6.50e-02 | 0.2477578846 |
|  | 18 | 7011217 | 7011463 | PDFSTATSENSEMBLUCSC | 247 | 4 | 19051 | gene;island | 0.492370 | 0.8785936 | Not queried | 2.50 | 2.00e-01 | 0.3826488233 |
|  | 4 | 174452835 | 174453287 | PDFSTATSENSEMBLUCSC | 453 | 5 | 30181 | gene;island | 0.017926 | 0.2769595 | Not queried | 2.50 | 2.22e-01 | 0.3935758299 |
|  | 16 | 46603015 | 46603321 | PDFSTATSENSEMBLUCSC | 307 | 6 | 15218 | tss | 0.789595 | 0.6006238 | Not queried | 2.50 | 5.89e-01 | 0.6570484697 |
|  | 1 | 207669922 | 207670205 | PDFSTATSENSEMBLUCSC | 284 | 4 | 978 | gene;island | 0.151272 | 0.0244846 | Not queried | 2.50 | 3.43e-01 | 0.4672865596 |
|  | 6 | 167136206 | 167136432 | PDFSTATSENSEMBLUCSC | 227 | 4 | 34554 | gene | 0.220989 | 0.6049366 | Not queried | 2.50 | 3.43e-01 | 0.4672865596 |
|  | 8 | 65488640 | 65489065 | PDFSTATSENSEMBLUCSC | 426 | 5 | 39322 | gene;island | 0.111812 | 0.0106453 | Not queried | 2.50 | 1.51e-01 | 0.3618000441 |
|  | 4 | 1407592 | 1407989 | PDFSTATSENSEMBLUCSC | 398 | 4 | 30786 | island | 0.413352 | 0.6378061 | Not queried | 2.49 | 3.43e-01 | 0.4672865596 |
|  | 7 | 157414294 | 157414580 | PDFSTATSENSEMBLUCSC | 287 | 4 | 37333 | gene;island | 0.789301 | 0.9586676 | Not queried | 2.49 | 1.14e-01 | 0.3123457953 |
|  | 2 | 220412406 | 220412606 | PDFSTATSENSEMBLUCSC | 201 | 4 | 23038 | gene;island | 0.529309 | 0.7342977 | Not queried | 2.49 | 2.00e-01 | 0.3826488233 |
|  | 19 | 13976610 | 13976956 | PDFSTATSENSEMBLUCSC | 347 | 5 | 21660 | island | 0.009371 | 0.0214785 | Not queried | 2.49 | 2.22e-01 | 0.3935758299 |
|  | 10 | 81679675 | 81679852 | PDFSTATSENSEMBLUCSC | 178 | 4 | 5184 | tss | 0.658993 | 0.8854888 | Not queried | 2.49 | 2.00e-01 | 0.3826488233 |
|  | 12 | 123757860 | 123758062 | PDFSTATSENSEMBLUCSC | 203 | 4 | 10099 | tss;island | 0.979664 | 0.7697336 | Not queried | 2.49 | 6.86e-01 | 0.7223604052 |
|  | 3 | 194014481 | 194014928 | PDFSTATSENSEMBLUCSC | 448 | 6 | 29786 | island | 0.423510 | 0.8401943 | Not queried | 2.49 | 1.80e-01 | 0.3826488233 |
|  | 22 | 51221675 | 51221773 | PDFSTATSENSEMBLUCSC | 99 | 4 | 26935 | gene;tss;island | 0.167406 | 0.0157013 | Not queried | 2.49 | 3.43e-01 | 0.4672865596 |
|  | 20 | 31780882 | 31781393 | PDFSTATSENSEMBLUCSC | 512 | 7 | 25680 | tss | 0.013543 | 0.0182476 | Not queried | 2.49 | 4.56e-01 | 0.5640816327 |
|  | 2 | 161127463 | 161127884 | PDFSTATSENSEMBLUCSC | 422 | 5 | 24815 | island | 0.837120 | 0.9550362 | Not queried | 2.49 | 5.56e-02 | 0.2315877671 |
|  | 19 | 14550846 | 14550999 | PDFSTATSENSEMBLUCSC | 154 | 4 | 19892 | gene;tss;island | 0.114997 | 0.0074450 | Not queried | 2.49 | 6.86e-01 | 0.7223604052 |
|  | 8 | 11559039 | 11559488 | PDFSTATSENSEMBLUCSC | 450 | 4 | 40085 | island | 0.050359 | 0.0249222 | Not queried | 2.49 | 2.00e-01 | 0.3826488233 |
|  | 17 | 46656543 | 46656690 | PDFSTATSENSEMBLUCSC | 148 | 4 | 17460 | tss;island | 0.525402 | 0.2772957 | Not queried | 2.49 | 6.86e-01 | 0.7223604052 |
|  | 2 | 112917127 | 112917424 | PDFSTATSENSEMBLUCSC | 298 | 4 | 22827 | gene;island | 0.477260 | 0.6303392 | Not queried | 2.49 | 3.43e-01 | 0.4672865596 |
|  | 2 | 223164459 | 223164925 | PDFSTATSENSEMBLUCSC | 467 | 8 | 23049 | gene;tss;island | 0.212004 | 0.0306191 | Not queried | 2.49 | 3.79e-02 | 0.2121545571 |
|  | 7 | 4850075 | 4850345 | PDFSTATSENSEMBLUCSC | 271 | 4 | 36858 | gene;island | 0.522189 | 0.8764419 | Not queried | 2.49 | 5.71e-02 | 0.2315877671 |
|  | 18 | 21977798 | 21978221 | PDFSTATSENSEMBLUCSC | 424 | 8 | 19212 | tss | 0.069647 | 0.0181397 | Not queried | 2.49 | 3.79e-02 | 0.2121545571 |
|  | 4 | 8412369 | 8412795 | PDFSTATSENSEMBLUCSC | 427 | 4 | 29952 | gene | 0.518274 | 0.7071051 | Not queried | 2.49 | 6.86e-01 | 0.7223604052 |
|  | 11 | 101918304 | 101918710 | PDFSTATSENSEMBLUCSC | 407 | 4 | 6876 | gene | 0.060978 | 0.2725801 | Not queried | 2.48 | 2.00e-01 | 0.3826488233 |
|  | 6 | 170571686 | 170572016 | PDFSTATSENSEMBLUCSC | 331 | 8 | 35354 | tss | 0.451802 | 0.5009088 | Not queried | 2.48 | 1.95e-01 | 0.3826488233 |
|  | 2 | 157291759 | 157292127 | PDFSTATSENSEMBLUCSC | 369 | 6 | 23674 | tss;island | 0.090812 | 0.6223004 | Not queried | 2.48 | 3.10e-01 | 0.4672865596 |
|  | 4 | 187629232 | 187629520 | PDFSTATSENSEMBLUCSC | 289 | 8 | 30219 | gene | 0.656204 | 0.8961757 | Not queried | 2.48 | 4.99e-02 | 0.2315877671 |
|  | 8 | 145925708 | 145926089 | PDFSTATSENSEMBLUCSC | 382 | 5 | 40746 | island | 0.378131 | 0.2338769 | Not queried | 2.48 | 2.22e-01 | 0.3935758299 |
|  | 12 | 50677228 | 50677281 | PDFSTATSENSEMBLUCSC | 54 | 4 | 9128 | gene | 0.006374 | 0.0046811 | Not queried | 2.48 | 2.00e-01 | 0.3826488233 |
|  | 15 | 47476133 | 47476482 | PDFSTATSENSEMBLUCSC | 350 | 6 | 14118 | island | 0.061104 | 0.1778840 | Not queried | 2.48 | 1.80e-01 | 0.3826488233 |
|  | 1 | 164290179 | 164290275 | PDFSTATSENSEMBLUCSC | 97 | 4 | 3924 | island | 0.070561 | 0.2172307 | Not queried | 2.48 | 6.86e-01 | 0.7223604052 |
|  | 19 | 11199851 | 11199965 | PDFSTATSENSEMBLUCSC | 115 | 6 | 20464 | tss | 0.001274 | 0.0037361 | Not queried | 2.47 | 4.85e-01 | 0.5640816327 |
|  | 3 | 35720994 | 35721284 | PDFSTATSENSEMBLUCSC | 291 | 5 | 27712 | gene;tss | 0.169669 | 0.0167726 | Not queried | 2.47 | 6.90e-01 | 0.7223604052 |
|  | 6 | 112375186 | 112375333 | PDFSTATSENSEMBLUCSC | 148 | 6 | 35180 | tss | 0.283487 | 0.6562395 | Not queried | 2.47 | 1.52e-02 | 0.1830867741 |
|  | 5 | 98104747 | 98105150 | PDFSTATSENSEMBLUCSC | 404 | 5 | 32981 | island | 0.017657 | 0.0046316 | Not queried | 2.47 | 2.22e-01 | 0.3935758299 |
|  | 22 | 17602561 | 17602681 | PDFSTATSENSEMBLUCSC | 121 | 4 | 26941 | tss;island | 0.044335 | 0.2601731 | Not queried | 2.47 | 1.14e-01 | 0.3123457953 |
|  | 1 | 89357908 | 89358043 | PDFSTATSENSEMBLUCSC | 136 | 4 | 1737 | tss;island | 0.878085 | 0.9789418 | Not queried | 2.47 | 1.14e-01 | 0.3123457953 |
|  | 1 | 7022041 | 7022284 | PDFSTATSENSEMBLUCSC | 244 | 4 | 183 | gene;island | 0.197626 | 0.5446307 | Not queried | 2.47 | 2.00e-01 | 0.3826488233 |
|  | 16 | 202311 | 202542 | PDFSTATSENSEMBLUCSC | 232 | 4 | 14961 | tss;island | 0.203292 | 0.4525171 | Not queried | 2.47 | 4.86e-01 | 0.5640816327 |
|  | 2 | 74669349 | 74669516 | PDFSTATSENSEMBLUCSC | 168 | 5 | 23454 | tss;island | 0.559135 | 0.7124505 | Not queried | 2.47 | 2.22e-01 | 0.3935758299 |
|  | 16 | 3414983 | 3415311 | PDFSTATSENSEMBLUCSC | 329 | 5 | 15647 | island | 0.007182 | 0.1895461 | Not queried | 2.47 | 1.51e-01 | 0.3618000441 |
|  | 16 | 67232180 | 67232557 | PDFSTATSENSEMBLUCSC | 378 | 4 | 14793 | gene;tss;island | 0.694923 | 0.4717267 | Not queried | 2.47 | 2.00e-01 | 0.3826488233 |
|  | 2 | 54682758 | 54683048 | PDFSTATSENSEMBLUCSC | 291 | 4 | 23356 | tss;island | 0.017815 | 0.1039624 | Not queried | 2.47 | 2.86e-02 | 0.1830867741 |
|  | 1 | 161697052 | 161697281 | PDFSTATSENSEMBLUCSC | 230 | 4 | 855 | gene;island | 0.004079 | 0.0008139 | Not queried | 2.47 | 2.00e-01 | 0.3826488233 |
|  | 17 | 40086714 | 40086869 | PDFSTATSENSEMBLUCSC | 156 | 5 | 17349 | tss | 0.006188 | 0.0047420 | Not queried | 2.46 | 2.22e-01 | 0.3935758299 |
|  | 2 | 31806042 | 31806551 | PDFSTATSENSEMBLUCSC | 510 | 6 | 23289 | tss | 0.779428 | 0.4528895 | Not queried | 2.46 | 3.10e-01 | 0.4672865596 |
|  | 6 | 25652381 | 25652815 | PDFSTATSENSEMBLUCSC | 435 | 10 | 35499 | island | 0.465571 | 0.8122825 | Not queried | 2.46 | 2.18e-01 | 0.3935758299 |
|  | 6 | 50818685 | 50819236 | PDFSTATSENSEMBLUCSC | 552 | 4 | 36200 | island | 0.007350 | 0.0307307 | Not queried | 2.46 | 2.00e-01 | 0.3826488233 |
|  | 6 | 10384975 | 10385489 | PDFSTATSENSEMBLUCSC | 515 | 6 | 35423 | island | 0.209734 | 0.0222228 | Not queried | 2.46 | 3.10e-01 | 0.4672865596 |
|  | 17 | 78388520 | 78388593 | PDFSTATSENSEMBLUCSC | 74 | 4 | 16867 | gene;tss;island | 0.043786 | 0.1509505 | Not queried | 2.46 | 2.86e-02 | 0.1830867741 |
|  | 11 | 122852284 | 122852698 | PDFSTATSENSEMBLUCSC | 415 | 9 | 8904 | island | 0.049781 | 0.2107363 | Not queried | 2.46 | 2.97e-01 | 0.4672865596 |
|  | 16 | 88590325 | 88590467 | PDFSTATSENSEMBLUCSC | 143 | 5 | 14908 | gene;island | 0.559529 | 0.9614388 | Not queried | 2.46 | 5.56e-02 | 0.2315877671 |
|  | 7 | 5938193 | 5938293 | PDFSTATSENSEMBLUCSC | 101 | 6 | 37428 | tss;island | 0.002997 | 0.0069024 | Not queried | 2.46 | 1.32e-01 | 0.3397986247 |
|  | 16 | 58497714 | 58497815 | PDFSTATSENSEMBLUCSC | 102 | 4 | 14771 | gene;tss;island | 0.000790 | 0.0047393 | Not queried | 2.46 | 6.86e-01 | 0.7223604052 |
|  | 1 | 117753591 | 117753741 | PDFSTATSENSEMBLUCSC | 151 | 4 | 1870 | tss | 0.211275 | 0.5609836 | Not queried | 2.46 | 3.43e-01 | 0.4672865596 |
|  | 6 | 44355135 | 44355287 | PDFSTATSENSEMBLUCSC | 153 | 6 | 35014 | tss;island | 0.001748 | 0.0053898 | Not queried | 2.46 | 9.31e-02 | 0.3104222821 |
|  | 7 | 150211761 | 150211939 | PDFSTATSENSEMBLUCSC | 179 | 5 | 37981 | tss | 0.012873 | 0.0170158 | Not queried | 2.46 | 6.90e-01 | 0.7223604052 |
|  | 12 | 85305677 | 85306021 | PDFSTATSENSEMBLUCSC | 345 | 4 | 9231 | gene;island | 0.019523 | 0.1345911 | Not queried | 2.46 | 4.86e-01 | 0.5640816327 |
|  | 5 | 50265272 | 50265767 | PDFSTATSENSEMBLUCSC | 496 | 5 | 32786 | island | 0.037838 | 0.1620725 | Not queried | 2.46 | 1.51e-01 | 0.3618000441 |
|  | 11 | 69933927 | 69934128 | PDFSTATSENSEMBLUCSC | 202 | 5 | 6786 | gene;island | 0.051572 | 0.7081570 | Not queried | 2.46 | 9.52e-02 | 0.3104222821 |
|  | 11 | 44972672 | 44973218 | PDFSTATSENSEMBLUCSC | 547 | 10 | 7263 | tss | 0.118308 | 0.4868528 | Not queried | 2.46 | 2.18e-01 | 0.3935758299 |
|  | 19 | 54024649 | 54024834 | PDFSTATSENSEMBLUCSC | 186 | 4 | 20205 | gene;island | 0.231225 | 0.0556079 | Not queried | 2.46 | 2.00e-01 | 0.3826488233 |
|  | 16 | 56224504 | 56225059 | PDFSTATSENSEMBLUCSC | 556 | 6 | 15242 | tss;island | 0.029157 | 0.0056247 | Not queried | 2.45 | 2.60e-02 | 0.1830867741 |
|  | 12 | 58011764 | 58011875 | PDFSTATSENSEMBLUCSC | 112 | 4 | 10567 | island | 0.419482 | 0.8086863 | Not queried | 2.45 | 2.00e-01 | 0.3826488233 |
|  | 1 | 197887955 | 197888512 | PDFSTATSENSEMBLUCSC | 558 | 6 | 931 | gene;island | 0.197446 | 0.0170297 | Not queried | 2.45 | 9.31e-02 | 0.3104222821 |
|  | 5 | 178684416 | 178684658 | PDFSTATSENSEMBLUCSC | 243 | 4 | 31931 | gene;island | 0.532573 | 0.8428093 | Not queried | 2.45 | 2.00e-01 | 0.3826488233 |
|  | 15 | 101926307 | 101926872 | PDFSTATSENSEMBLUCSC | 566 | 5 | 13509 | gene | 0.433577 | 0.5801094 | Not queried | 2.45 | 2.22e-01 | 0.3935758299 |
|  | 1 | 50798944 | 50799316 | PDFSTATSENSEMBLUCSC | 373 | 4 | 3251 | island | 0.011136 | 0.0525112 | Not queried | 2.45 | 2.00e-01 | 0.3826488233 |
|  | 7 | 49812836 | 49813111 | PDFSTATSENSEMBLUCSC | 276 | 7 | 37592 | tss;island | 0.018124 | 0.0761940 | Not queried | 2.45 | 7.28e-02 | 0.2713481487 |
|  | 13 | 112728799 | 112728964 | PDFSTATSENSEMBLUCSC | 166 | 4 | 11914 | island | 0.012198 | 0.0425340 | Not queried | 2.45 | 2.00e-01 | 0.3826488233 |
|  | 13 | 25946657 | 25947159 | PDFSTATSENSEMBLUCSC | 503 | 4 | 11109 | gene;island | 0.316825 | 0.6119701 | Not queried | 2.45 | 4.86e-01 | 0.5640816327 |
|  | 11 | 108408907 | 108408981 | PDFSTATSENSEMBLUCSC | 75 | 5 | 6889 | gene | 0.982541 | 0.9188900 | Not queried | 2.45 | 5.56e-02 | 0.2315877671 |
|  | 2 | 145281942 | 145282262 | PDFSTATSENSEMBLUCSC | 321 | 4 | 24771 | island | 0.052275 | 0.0216455 | Not queried | 2.44 | 4.86e-01 | 0.5640816327 |
|  | 5 | 134363324 | 134363973 | PDFSTATSENSEMBLUCSC | 650 | 7 | 33129 | island | 0.250561 | 0.0843992 | Not queried | 2.44 | 3.18e-01 | 0.4672865596 |
|  | 11 | 44972574 | 44973218 | PDFSTATSENSEMBLUCSC | 645 | 11 | 8229 | island | 0.084422 | 0.4760670 | Not queried | 2.44 | 2.17e-01 | 0.3935758299 |
|  | 10 | 124894943 | 124895886 | PDFSTATSENSEMBLUCSC | 944 | 13 | 6215 | island | 0.043127 | 0.0112991 | Not queried | 2.44 | 1.69e-01 | 0.3826488233 |
|  | 10 | 124894943 | 124895500 | PDFSTATSENSEMBLUCSC | 558 | 9 | 5415 | tss | 0.108226 | 0.0219478 | Not queried | 2.44 | 2.97e-01 | 0.4672865596 |
|  | 19 | 33764284 | 33764550 | PDFSTATSENSEMBLUCSC | 267 | 4 | 21873 | island | 0.281618 | 0.6851568 | Not queried | 2.44 | 3.43e-01 | 0.4672865596 |
|  | 6 | 8102871 | 8103092 | PDFSTATSENSEMBLUCSC | 222 | 8 | 34619 | tss | 0.029157 | 0.0058591 | Not queried | 2.44 | 3.28e-01 | 0.4672865596 |
|  | 1 | 37499309 | 37499649 | PDFSTATSENSEMBLUCSC | 341 | 4 | 431 | gene;island | 0.039568 | 0.2075014 | Not queried | 2.44 | 2.86e-02 | 0.1830867741 |
|  | 5 | 139944046 | 139944115 | PDFSTATSENSEMBLUCSC | 70 | 4 | 31787 | gene | 0.004161 | 0.0006765 | Not queried | 2.44 | 4.86e-01 | 0.5640816327 |
|  | 5 | 1201359 | 1201713 | PDFSTATSENSEMBLUCSC | 355 | 10 | 32613 | island | 0.463140 | 0.4898382 | Not queried | 2.44 | 6.84e-01 | 0.7223604052 |
|  | 6 | 106428957 | 106429769 | PDFSTATSENSEMBLUCSC | 813 | 9 | 36377 | island | 0.148291 | 0.0416221 | Not queried | 2.44 | 6.25e-02 | 0.2477578846 |
|  | 8 | 19796299 | 19796591 | PDFSTATSENSEMBLUCSC | 293 | 6 | 40114 | island | 0.179155 | 0.0274353 | Not queried | 2.44 | 3.10e-01 | 0.4672865596 |
|  | 2 | 176993017 | 176993320 | PDFSTATSENSEMBLUCSC | 304 | 6 | 23738 | tss;island | 0.024281 | 0.2123277 | Not queried | 2.44 | 4.85e-01 | 0.5640816327 |
|  | 19 | 4769146 | 4769240 | PDFSTATSENSEMBLUCSC | 95 | 4 | 19738 | gene | 0.028770 | 0.1599287 | Not queried | 2.44 | 2.86e-02 | 0.1830867741 |
|  | 2 | 114048829 | 114049041 | PDFSTATSENSEMBLUCSC | 213 | 5 | 22835 | gene;island | 0.067248 | 0.4391131 | Not queried | 2.44 | 5.56e-02 | 0.2315877671 |
|  | 15 | 75470777 | 75471218 | PDFSTATSENSEMBLUCSC | 442 | 6 | 14325 | island | 0.138255 | 0.0159467 | Not queried | 2.44 | 8.66e-03 | 0.1595382395 |
|  | 17 | 4337082 | 4337202 | PDFSTATSENSEMBLUCSC | 121 | 5 | 17023 | tss | 0.011567 | 0.0665275 | Not queried | 2.43 | 9.52e-02 | 0.3104222821 |
|  | 18 | 52626476 | 52626848 | PDFSTATSENSEMBLUCSC | 373 | 5 | 19285 | tss;island | 0.086313 | 0.0207855 | Not queried | 2.43 | 1.51e-01 | 0.3618000441 |
|  | 11 | 26353484 | 26353811 | PDFSTATSENSEMBLUCSC | 328 | 8 | 8140 | island | 0.024426 | 0.0526714 | Not queried | 2.43 | 6.50e-02 | 0.2477578846 |
|  | 12 | 8332640 | 8333192 | PDFSTATSENSEMBLUCSC | 553 | 9 | 10233 | island | 0.032281 | 0.1930658 | Not queried | 2.43 | 4.00e-02 | 0.2180389943 |
|  | 19 | 39694477 | 39695237 | PDFSTATSENSEMBLUCSC | 761 | 9 | 21990 | island | 0.156392 | 0.3870774 | Not queried | 2.43 | 2.58e-01 | 0.4381244265 |
|  | 3 | 124931535 | 124931746 | PDFSTATSENSEMBLUCSC | 212 | 5 | 29419 | island | 0.292674 | 0.0510194 | Not queried | 2.43 | 3.10e-01 | 0.4672865596 |
|  | 4 | 187476326 | 187477065 | PDFSTATSENSEMBLUCSC | 740 | 11 | 31483 | island | 0.071298 | 0.3663095 | Not queried | 2.43 | 8.33e-03 | 0.1595382395 |
|  | 4 | 187476543 | 187477065 | PDFSTATSENSEMBLUCSC | 523 | 9 | 30707 | tss | 0.071298 | 0.3663095 | Not queried | 2.43 | 3.15e-02 | 0.1866936846 |
|  | 20 | 591076 | 591400 | PDFSTATSENSEMBLUCSC | 325 | 6 | 25528 | tss;island | 0.134742 | 0.5015485 | Not queried | 2.43 | 1.80e-01 | 0.3826488233 |
|  | 2 | 176977220 | 176977458 | PDFSTATSENSEMBLUCSC | 239 | 4 | 24898 | island | 0.051615 | 0.3324358 | Not queried | 2.43 | 2.00e-01 | 0.3826488233 |
|  | 20 | 52789956 | 52790141 | PDFSTATSENSEMBLUCSC | 186 | 4 | 25458 | gene;island | 0.090433 | 0.0126912 | Not queried | 2.43 | 1.14e-01 | 0.3123457953 |
|  | 1 | 27874258 | 27874574 | PDFSTATSENSEMBLUCSC | 317 | 5 | 356 | gene;island | 0.598947 | 0.9431139 | Not queried | 2.42 | 3.17e-02 | 0.1866936846 |
|  | 15 | 101629063 | 101629375 | PDFSTATSENSEMBLUCSC | 313 | 4 | 14514 | island | 0.084007 | 0.1845655 | Not queried | 2.42 | 6.86e-01 | 0.7223604052 |
|  | 15 | 74659901 | 74660566 | PDFSTATSENSEMBLUCSC | 666 | 7 | 13792 | tss;island | 0.439354 | 0.7610741 | Not queried | 2.42 | 1.75e-02 | 0.1830867741 |
|  | 16 | 67562424 | 67562672 | PDFSTATSENSEMBLUCSC | 249 | 4 | 15305 | tss;island | 0.030328 | 0.1496989 | Not queried | 2.42 | 4.86e-01 | 0.5640816327 |
|  | 18 | 3498854 | 3499253 | PDFSTATSENSEMBLUCSC | 400 | 4 | 19044 | gene;island | 0.424270 | 0.3375687 | Not queried | 2.42 | 8.86e-01 | 0.8941250130 |
|  | 7 | 27153636 | 27153944 | PDFSTATSENSEMBLUCSC | 309 | 5 | 37489 | tss | 0.678560 | 0.9649139 | Not queried | 2.42 | 3.10e-01 | 0.4672865596 |
|  | 20 | 45179226 | 45179413 | PDFSTATSENSEMBLUCSC | 188 | 5 | 25793 | tss | 0.510187 | 0.4486714 | Not queried | 2.42 | 8.41e-01 | 0.8635565145 |
|  | 14 | 96180576 | 96181044 | PDFSTATSENSEMBLUCSC | 469 | 9 | 12590 | tss | 0.043242 | 0.1738057 | Not queried | 2.42 | 7.70e-02 | 0.2825346647 |
|  | 15 | 37403088 | 37403304 | PDFSTATSENSEMBLUCSC | 217 | 5 | 14015 | island | 0.047988 | 0.5030541 | Not queried | 2.42 | 2.22e-01 | 0.3935758299 |
|  | 6 | 134209935 | 134210257 | PDFSTATSENSEMBLUCSC | 323 | 6 | 35232 | tss | 0.039457 | 0.3151910 | Not queried | 2.41 | 4.85e-01 | 0.5640816327 |
|  | 10 | 134361889 | 134362170 | PDFSTATSENSEMBLUCSC | 282 | 6 | 4865 | gene;island | 0.720573 | 0.9379951 | Not queried | 2.41 | 2.60e-02 | 0.1830867741 |
|  | 2 | 72376056 | 72376239 | PDFSTATSENSEMBLUCSC | 184 | 4 | 23432 | tss;island | 0.003111 | 0.0111729 | Not queried | 2.41 | 2.86e-02 | 0.1830867741 |
|  | 11 | 94134646 | 94135029 | PDFSTATSENSEMBLUCSC | 384 | 8 | 7629 | tss | 0.244520 | 0.0665760 | Not queried | 2.41 | 1.61e-01 | 0.3814043454 |
|  | 17 | 7486551 | 7486961 | PDFSTATSENSEMBLUCSC | 411 | 9 | 17090 | tss | 0.365900 | 0.5155269 | Not queried | 2.41 | 2.22e-01 | 0.3935758299 |
|  | 14 | 104690040 | 104690244 | PDFSTATSENSEMBLUCSC | 205 | 5 | 13204 | island | 0.202987 | 0.0334864 | Not queried | 2.41 | 9.52e-02 | 0.3104222821 |
|  | 11 | 101454626 | 101454996 | PDFSTATSENSEMBLUCSC | 371 | 8 | 8756 | island | 0.060003 | 0.3457013 | Not queried | 2.41 | 1.30e-01 | 0.3397986247 |
|  | 10 | 43697817 | 43698141 | PDFSTATSENSEMBLUCSC | 325 | 5 | 4568 | gene;island | 0.005352 | 0.0190274 | Not queried | 2.41 | 5.48e-01 | 0.6179718485 |
|  | 5 | 148928885 | 148929784 | PDFSTATSENSEMBLUCSC | 900 | 8 | 31858 | gene;island | 0.085766 | 0.0040202 | Not queried | 2.41 | 2.79e-01 | 0.4672865596 |
|  | 2 | 178976981 | 178977094 | PDFSTATSENSEMBLUCSC | 114 | 5 | 23753 | tss | 0.020174 | 0.0060979 | Not queried | 2.41 | 8.41e-01 | 0.8635565145 |
|  | 1 | 223538174 | 223538379 | PDFSTATSENSEMBLUCSC | 206 | 4 | 2271 | tss;island | 0.040684 | 0.2244590 | Not queried | 2.41 | 5.71e-02 | 0.2315877671 |
|  | 11 | 32457124 | 32457386 | PDFSTATSENSEMBLUCSC | 263 | 7 | 7226 | tss;island | 0.090051 | 0.0133918 | Not queried | 2.41 | 1.75e-02 | 0.1830867741 |
|  | 8 | 1501458 | 1501670 | PDFSTATSENSEMBLUCSC | 213 | 4 | 39168 | gene;island | 0.543488 | 0.8934609 | Not queried | 2.41 | 5.71e-02 | 0.2315877671 |
|  | 3 | 147131069 | 147131332 | PDFSTATSENSEMBLUCSC | 264 | 4 | 27990 | gene;island | 0.161157 | 0.0274946 | Not queried | 2.40 | 3.43e-01 | 0.4672865596 |
|  | 11 | 118478236 | 118478329 | PDFSTATSENSEMBLUCSC | 94 | 4 | 6931 | gene;island | 0.042988 | 0.2391585 | Not queried | 2.40 | 3.43e-01 | 0.4672865596 |
|  | 12 | 98850691 | 98851021 | PDFSTATSENSEMBLUCSC | 331 | 6 | 10703 | island | 0.015007 | 0.1627761 | Not queried | 2.40 | 3.10e-01 | 0.4672865596 |
|  | 2 | 84743142 | 84743743 | PDFSTATSENSEMBLUCSC | 602 | 12 | 24469 | island | 0.065255 | 0.0184985 | Not queried | 2.40 | 2.84e-02 | 0.1830867741 |
|  | 20 | 37353032 | 37353158 | PDFSTATSENSEMBLUCSC | 127 | 7 | 26159 | island | 0.047291 | 0.0187041 | Not queried | 2.40 | 3.79e-02 | 0.2121545571 |
|  | 11 | 695873 | 695896 | PDFSTATSENSEMBLUCSC | 24 | 4 | 6388 | gene;tss;island | 0.001820 | 0.0042941 | Not queried | 2.40 | 2.00e-01 | 0.3826488233 |
|  | 10 | 92631135 | 92631706 | PDFSTATSENSEMBLUCSC | 572 | 12 | 5219 | tss | 0.010630 | 0.0026497 | Not queried | 2.40 | 1.14e-01 | 0.3123457953 |
|  | 20 | 43160245 | 43160301 | PDFSTATSENSEMBLUCSC | 57 | 4 | 25762 | tss;island | 0.011777 | 0.0866389 | Not queried | 2.40 | 1.14e-01 | 0.3123457953 |
|  | 4 | 174438930 | 174440103 | PDFSTATSENSEMBLUCSC | 1174 | 10 | 31426 | island | 0.105196 | 0.3370831 | Not queried | 2.40 | 2.88e-02 | 0.1834529198 |
|  | 2 | 14772312 | 14772734 | PDFSTATSENSEMBLUCSC | 423 | 7 | 23195 | tss;island | 0.060575 | 0.1856801 | Not queried | 2.40 | 1.28e-01 | 0.3397986247 |
|  | 4 | 155663529 | 155664311 | PDFSTATSENSEMBLUCSC | 783 | 8 | 31386 | island | 0.031154 | 0.1181505 | Not queried | 2.40 | 1.61e-01 | 0.3814043454 |
|  | 14 | 23834710 | 23834995 | PDFSTATSENSEMBLUCSC | 286 | 8 | 12718 | island | 0.011672 | 0.0480606 | Not queried | 2.40 | 2.07e-02 | 0.1830867741 |
|  | 6 | 166908379 | 166908811 | PDFSTATSENSEMBLUCSC | 433 | 5 | 34548 | gene | 0.346421 | 0.8092010 | Not queried | 2.40 | 3.10e-01 | 0.4672865596 |
|  | 7 | 158109339 | 158109694 | PDFSTATSENSEMBLUCSC | 356 | 4 | 37367 | gene;island | 0.586084 | 0.8617274 | Not queried | 2.39 | 2.00e-01 | 0.3826488233 |
|  | 6 | 133563764 | 133564066 | PDFSTATSENSEMBLUCSC | 303 | 4 | 34477 | gene;island | 0.046992 | 0.1380449 | Not queried | 2.39 | 3.43e-01 | 0.4672865596 |
|  | 11 | 44337982 | 44338421 | PDFSTATSENSEMBLUCSC | 440 | 5 | 8226 | island | 0.323426 | 0.0418472 | Not queried | 2.39 | 5.56e-02 | 0.2315877671 |
|  | 17 | 62084205 | 62084359 | PDFSTATSENSEMBLUCSC | 155 | 5 | 16731 | gene | 0.557219 | 0.8686408 | Not queried | 2.39 | 5.48e-01 | 0.6179718485 |
|  | 5 | 1076587 | 1076892 | PDFSTATSENSEMBLUCSC | 306 | 5 | 31518 | gene;island | 0.896603 | 0.6215861 | Not queried | 2.39 | 2.22e-01 | 0.3935758299 |
|  | 1 | 19600673 | 19601069 | PDFSTATSENSEMBLUCSC | 397 | 9 | 1301 | tss;island | 0.473330 | 0.1177154 | Not queried | 2.39 | 3.15e-02 | 0.1866936846 |
|  | 11 | 62314820 | 62315119 | PDFSTATSENSEMBLUCSC | 300 | 4 | 7345 | tss;island | 0.420437 | 0.6351212 | Not queried | 2.39 | 1.14e-01 | 0.3123457953 |
|  | 4 | 55524145 | 55524400 | PDFSTATSENSEMBLUCSC | 256 | 4 | 30005 | gene;island | 0.014639 | 0.0619903 | Not queried | 2.39 | 1.14e-01 | 0.3123457953 |
|  | 1 | 247275735 | 247276096 | PDFSTATSENSEMBLUCSC | 362 | 5 | 2367 | tss | 0.238521 | 0.6272460 | Not queried | 2.39 | 1.51e-01 | 0.3618000441 |
|  | 12 | 5603131 | 5603261 | PDFSTATSENSEMBLUCSC | 131 | 6 | 9019 | gene;tss;island | 0.185839 | 0.4083781 | Not queried | 2.39 | 1.32e-01 | 0.3397986247 |
|  | 5 | 172672684 | 172673349 | PDFSTATSENSEMBLUCSC | 666 | 8 | 33410 | island | 0.070328 | 0.2505509 | Not queried | 2.39 | 2.34e-01 | 0.4128374386 |
|  | 10 | 27609163 | 27609333 | PDFSTATSENSEMBLUCSC | 171 | 4 | 5629 | island | 0.628307 | 0.9364240 | Not queried | 2.39 | 4.86e-01 | 0.5640816327 |
|  | 3 | 33260732 | 33261204 | PDFSTATSENSEMBLUCSC | 473 | 7 | 28212 | tss;island | 0.076077 | 0.0778799 | Not queried | 2.39 | 7.10e-01 | 0.7390730975 |
|  | 2 | 242749925 | 242750102 | PDFSTATSENSEMBLUCSC | 178 | 5 | 23994 | tss | 0.691703 | 0.9657436 | Not queried | 2.39 | 7.94e-03 | 0.1589602024 |
|  | 6 | 31276418 | 31276797 | PDFSTATSENSEMBLUCSC | 380 | 7 | 35764 | island | 0.580301 | 0.0343728 | Not queried | 2.39 | 7.28e-02 | 0.2713481487 |
|  | 22 | 28010359 | 28010598 | PDFSTATSENSEMBLUCSC | 240 | 4 | 27380 | island | 0.114863 | 0.4472081 | Not queried | 2.38 | 2.86e-02 | 0.1830867741 |
|  | 3 | 52091484 | 52091829 | PDFSTATSENSEMBLUCSC | 346 | 4 | 28371 | tss;island | 0.022669 | 0.0156699 | Not queried | 2.38 | 3.43e-01 | 0.4672865596 |
|  | 5 | 78407418 | 78407566 | PDFSTATSENSEMBLUCSC | 149 | 6 | 32145 | tss | 0.441072 | 0.7441151 | Not queried | 2.38 | 1.52e-02 | 0.1830867741 |
|  | 11 | 134262988 | 134263349 | PDFSTATSENSEMBLUCSC | 362 | 5 | 7001 | gene | 0.139983 | 0.4378882 | Not queried | 2.38 | 2.22e-01 | 0.3935758299 |
|  | 3 | 195384522 | 195384752 | PDFSTATSENSEMBLUCSC | 231 | 6 | 28805 | tss;island | 0.147007 | 0.0122928 | Not queried | 2.38 | 3.10e-01 | 0.4672865596 |
|  | 12 | 107711872 | 107712210 | PDFSTATSENSEMBLUCSC | 339 | 6 | 10761 | island | 0.015648 | 0.0044481 | Not queried | 2.38 | 4.85e-01 | 0.5640816327 |
|  | 6 | 94129234 | 94129697 | PDFSTATSENSEMBLUCSC | 464 | 10 | 36331 | island | 0.032464 | 0.1218326 | Not queried | 2.38 | 2.88e-03 | 0.1170421274 |
|  | 8 | 104512858 | 104513186 | PDFSTATSENSEMBLUCSC | 329 | 4 | 40521 | island | 0.032200 | 0.0153260 | Not queried | 2.38 | 3.43e-01 | 0.4672865596 |
|  | 12 | 14720726 | 14721288 | PDFSTATSENSEMBLUCSC | 563 | 10 | 10264 | island | 0.571683 | 0.2938349 | Not queried | 2.38 | 1.90e-01 | 0.3826488233 |
|  | 1 | 34642000 | 34642519 | PDFSTATSENSEMBLUCSC | 520 | 8 | 1477 | tss | 0.631119 | 0.4891831 | Not queried | 2.38 | 3.82e-01 | 0.5168893301 |
|  | 22 | 31536476 | 31536784 | PDFSTATSENSEMBLUCSC | 309 | 5 | 27075 | tss | 0.030401 | 0.1194275 | Not queried | 2.38 | 4.21e-01 | 0.5404252807 |
|  | 17 | 32483560 | 32484259 | PDFSTATSENSEMBLUCSC | 700 | 13 | 18200 | island | 0.040385 | 0.2445831 | Not queried | 2.38 | 2.23e-01 | 0.3936710914 |
|  | 3 | 27765232 | 27765546 | PDFSTATSENSEMBLUCSC | 315 | 5 | 28952 | island | 0.075664 | 0.0414450 | Not queried | 2.38 | 5.48e-01 | 0.6179718485 |
|  | 7 | 1135747 | 1136023 | PDFSTATSENSEMBLUCSC | 277 | 4 | 36750 | gene;island | 0.814985 | 0.9573961 | Not queried | 2.38 | 2.00e-01 | 0.3826488233 |
|  | 2 | 234776807 | 234777085 | PDFSTATSENSEMBLUCSC | 279 | 5 | 25212 | island | 0.013930 | 0.0526091 | Not queried | 2.38 | 3.10e-01 | 0.4672865596 |
|  | 16 | 1968576 | 1968709 | PDFSTATSENSEMBLUCSC | 134 | 4 | 15009 | tss;island | 0.097408 | 0.4936292 | Not queried | 2.37 | 3.43e-01 | 0.4672865596 |
|  | 3 | 121902442 | 121902622 | PDFSTATSENSEMBLUCSC | 181 | 7 | 29396 | island | 0.295385 | 0.4866874 | Not queried | 2.37 | 1.28e-01 | 0.3397986247 |
|  | 2 | 224904626 | 224904837 | PDFSTATSENSEMBLUCSC | 212 | 5 | 23924 | tss;island | 0.021635 | 0.1649503 | Not queried | 2.37 | 1.51e-01 | 0.3618000441 |
|  | 12 | 103889516 | 103890274 | PDFSTATSENSEMBLUCSC | 759 | 11 | 10731 | island | 0.150574 | 0.0164043 | Not queried | 2.37 | 2.81e-02 | 0.1830867741 |
|  | 12 | 50297477 | 50297945 | PDFSTATSENSEMBLUCSC | 469 | 7 | 10406 | island | 0.037908 | 0.1294793 | Not queried | 2.37 | 2.09e-01 | 0.3935758299 |
|  | 8 | 145625497 | 145625814 | PDFSTATSENSEMBLUCSC | 318 | 5 | 39520 | gene;island | 0.789853 | 0.9802872 | Not queried | 2.37 | 3.17e-02 | 0.1866936846 |
|  | 7 | 155601876 | 155602259 | PDFSTATSENSEMBLUCSC | 384 | 5 | 37312 | gene;island | 0.025247 | 0.0113338 | Not queried | 2.37 | 1.51e-01 | 0.3618000441 |
|  | 6 | 50808627 | 50808813 | PDFSTATSENSEMBLUCSC | 187 | 4 | 34366 | gene;island | 0.067105 | 0.0523690 | Not queried | 2.37 | 6.86e-01 | 0.7223604052 |
|  | 1 | 805338 | 805554 | PDFSTATSENSEMBLUCSC | 217 | 4 | 1 | gene;island | 0.284768 | 0.6287204 | Not queried | 2.37 | 4.86e-01 | 0.5640816327 |
|  | 18 | 44337664 | 44338147 | PDFSTATSENSEMBLUCSC | 484 | 6 | 19259 | tss;island | 0.029478 | 0.0645835 | Not queried | 2.37 | 3.10e-01 | 0.4672865596 |
|  | 1 | 917642 | 917892 | PDFSTATSENSEMBLUCSC | 251 | 5 | 1136 | tss;island | 0.050058 | 0.2125513 | Not queried | 2.36 | 6.90e-01 | 0.7223604052 |
|  | 11 | 20691126 | 20691429 | PDFSTATSENSEMBLUCSC | 304 | 5 | 6538 | gene | 0.006784 | 0.0337557 | Not queried | 2.36 | 3.17e-02 | 0.1866936846 |
|  | 2 | 23850319 | 23850586 | PDFSTATSENSEMBLUCSC | 268 | 4 | 22595 | gene;island | 0.949451 | 0.7738259 | Not queried | 2.36 | 2.86e-02 | 0.1830867741 |
|  | 17 | 7342585 | 7342894 | PDFSTATSENSEMBLUCSC | 310 | 4 | 17949 | island | 0.046318 | 0.3328364 | Not queried | 2.36 | 1.14e-01 | 0.3123457953 |
|  | 11 | 61322742 | 61323413 | PDFSTATSENSEMBLUCSC | 672 | 6 | 6630 | gene;island | 0.063580 | 0.3341927 | Not queried | 2.36 | 3.10e-01 | 0.4672865596 |
|  | 8 | 20054372 | 20054699 | PDFSTATSENSEMBLUCSC | 328 | 8 | 39575 | tss | 0.046857 | 0.0176910 | Not queried | 2.36 | 3.28e-01 | 0.4672865596 |
|  | 5 | 1268644 | 1269309 | PDFSTATSENSEMBLUCSC | 666 | 8 | 31531 | gene;island | 0.961077 | 0.7684635 | Not queried | 2.36 | 6.50e-02 | 0.2477578846 |
|  | 12 | 121342197 | 121342435 | PDFSTATSENSEMBLUCSC | 239 | 5 | 10068 | tss;island | 0.011835 | 0.0081462 | Not queried | 2.36 | 4.21e-01 | 0.5404252807 |
|  | 13 | 84456127 | 84456486 | PDFSTATSENSEMBLUCSC | 360 | 6 | 11186 | gene | 0.137638 | 0.0308672 | Not queried | 2.36 | 1.80e-01 | 0.3826488233 |
|  | 10 | 23983496 | 23983538 | PDFSTATSENSEMBLUCSC | 43 | 4 | 4989 | tss | 0.019765 | 0.1594757 | Not queried | 2.36 | 2.00e-01 | 0.3826488233 |
|  | 6 | 168613133 | 168613387 | PDFSTATSENSEMBLUCSC | 255 | 4 | 36660 | island | 0.542335 | 0.7643234 | Not queried | 2.36 | 6.86e-01 | 0.7223604052 |
|  | 19 | 15121204 | 15121596 | PDFSTATSENSEMBLUCSC | 393 | 9 | 21691 | island | 0.447423 | 0.8079015 | Not queried | 2.36 | 9.39e-02 | 0.3104222821 |
|  | 6 | 31622318 | 31622439 | PDFSTATSENSEMBLUCSC | 122 | 6 | 34824 | tss;island | 0.913873 | 0.7537636 | Not queried | 2.35 | 1.80e-01 | 0.3826488233 |
|  | 17 | 37307911 | 37308278 | PDFSTATSENSEMBLUCSC | 368 | 6 | 17303 | tss;island | 0.024714 | 0.0053116 | Not queried | 2.35 | 3.94e-01 | 0.5236526859 |
|  | 15 | 26026152 | 26026414 | PDFSTATSENSEMBLUCSC | 263 | 6 | 13260 | gene | 0.360181 | 0.6919758 | Not queried | 2.35 | 9.31e-02 | 0.3104222821 |
|  | 3 | 64672542 | 64672750 | PDFSTATSENSEMBLUCSC | 209 | 4 | 27851 | gene;island | 0.031098 | 0.0095976 | Not queried | 2.35 | 4.86e-01 | 0.5640816327 |
|  | 2 | 26468086 | 26468406 | PDFSTATSENSEMBLUCSC | 321 | 5 | 22609 | gene;tss;island | 0.003437 | 0.0052212 | Not queried | 2.35 | 4.21e-01 | 0.5404252807 |
|  | 15 | 99789622 | 99789777 | PDFSTATSENSEMBLUCSC | 156 | 4 | 13502 | gene | 0.007294 | 0.0375466 | Not queried | 2.35 | 5.71e-02 | 0.2315877671 |
|  | 7 | 158279694 | 158279891 | PDFSTATSENSEMBLUCSC | 198 | 4 | 37376 | gene;island | 0.373791 | 0.4885707 | Not queried | 2.35 | 6.86e-01 | 0.7223604052 |
|  | 2 | 242497960 | 242498098 | PDFSTATSENSEMBLUCSC | 139 | 4 | 23991 | tss;island | 0.183523 | 0.4949677 | Not queried | 2.35 | 1.14e-01 | 0.3123457953 |
|  | 16 | 85362963 | 85363209 | PDFSTATSENSEMBLUCSC | 247 | 4 | 16154 | island | 0.145055 | 0.4400410 | Not queried | 2.35 | 2.00e-01 | 0.3826488233 |
|  | 19 | 13616871 | 13617094 | PDFSTATSENSEMBLUCSC | 224 | 4 | 19882 | gene;island | 0.155332 | 0.0282120 | Not queried | 2.35 | 1.14e-01 | 0.3123457953 |
|  | 1 | 16073336 | 16073520 | PDFSTATSENSEMBLUCSC | 185 | 4 | 251 | gene;island | 0.736475 | 0.9614973 | Not queried | 2.35 | 4.86e-01 | 0.5640816327 |
|  | 7 | 157366933 | 157367159 | PDFSTATSENSEMBLUCSC | 227 | 5 | 37328 | gene;island | 0.342701 | 0.7258505 | Not queried | 2.35 | 1.51e-01 | 0.3618000441 |
|  | 6 | 32178603 | 32178775 | PDFSTATSENSEMBLUCSC | 173 | 4 | 34091 | gene | 0.545648 | 0.8555073 | Not queried | 2.34 | 4.86e-01 | 0.5640816327 |
|  | 7 | 152456579 | 152456815 | PDFSTATSENSEMBLUCSC | 237 | 5 | 38007 | tss;island | 0.003538 | 0.0073717 | Not queried | 2.34 | 4.21e-01 | 0.5404252807 |
|  | 16 | 420490 | 420755 | PDFSTATSENSEMBLUCSC | 266 | 4 | 15453 | island | 0.000211 | 0.0074683 | Not queried | 2.34 | 3.43e-01 | 0.4672865596 |
|  | 8 | 72459908 | 72460135 | PDFSTATSENSEMBLUCSC | 228 | 4 | 40379 | island | 0.051699 | 0.2022811 | Not queried | 2.34 | 2.00e-01 | 0.3826488233 |
|  | 2 | 620044 | 620310 | PDFSTATSENSEMBLUCSC | 267 | 4 | 24008 | island | 0.259969 | 0.6291734 | Not queried | 2.34 | 2.00e-01 | 0.3826488233 |
|  | 9 | 117267242 | 117267672 | PDFSTATSENSEMBLUCSC | 431 | 5 | 40780 | gene;island | 0.008081 | 0.0349022 | Not queried | 2.34 | 6.90e-01 | 0.7223604052 |
|  | 17 | 79503642 | 79503877 | PDFSTATSENSEMBLUCSC | 236 | 4 | 16914 | gene;island | 0.024893 | 0.1643360 | Not queried | 2.34 | 8.86e-01 | 0.8941250130 |
|  | 12 | 85306323 | 85306574 | PDFSTATSENSEMBLUCSC | 252 | 4 | 9232 | gene | 0.014529 | 0.0856980 | Not queried | 2.34 | 5.71e-02 | 0.2315877671 |
|  | 2 | 177012117 | 177012706 | PDFSTATSENSEMBLUCSC | 590 | 7 | 24907 | island | 0.191349 | 0.1433182 | Not queried | 2.34 | 6.20e-01 | 0.6899391537 |
|  | 4 | 166299786 | 166300051 | PDFSTATSENSEMBLUCSC | 266 | 6 | 30670 | tss;island | 0.007627 | 0.0129834 | Not queried | 2.34 | 3.94e-01 | 0.5236526859 |
|  | 11 | 44116911 | 44117063 | PDFSTATSENSEMBLUCSC | 153 | 5 | 7260 | tss | 0.048107 | 0.0281401 | Not queried | 2.34 | 6.90e-01 | 0.7223604052 |
|  | 15 | 99193929 | 99194172 | PDFSTATSENSEMBLUCSC | 244 | 4 | 13495 | gene;island | 0.036045 | 0.0138735 | Not queried | 2.34 | 2.00e-01 | 0.3826488233 |
|  | 12 | 30948636 | 30948794 | PDFSTATSENSEMBLUCSC | 159 | 5 | 10308 | island | 0.019159 | 0.0896267 | Not queried | 2.34 | 3.10e-01 | 0.4672865596 |
|  | 20 | 62574023 | 62574273 | PDFSTATSENSEMBLUCSC | 251 | 5 | 25514 | gene;tss;island | 0.550443 | 0.8352042 | Not queried | 2.34 | 5.48e-01 | 0.6179718485 |
|  | 2 | 233390712 | 233390859 | PDFSTATSENSEMBLUCSC | 148 | 4 | 23950 | tss | 0.028899 | 0.3447050 | Not queried | 2.34 | 4.86e-01 | 0.5640816327 |
|  | 1 | 186649153 | 186649985 | PDFSTATSENSEMBLUCSC | 833 | 13 | 4047 | island | 0.046332 | 0.0072640 | Not queried | 2.34 | 2.87e-03 | 0.1170421274 |
|  | 11 | 64058550 | 64059057 | PDFSTATSENSEMBLUCSC | 508 | 6 | 8401 | island | 0.125574 | 0.2654192 | Not queried | 2.34 | 3.10e-01 | 0.4672865596 |
|  | 19 | 1275121 | 1275466 | PDFSTATSENSEMBLUCSC | 346 | 6 | 20299 | tss;island | 0.043809 | 0.0054162 | Not queried | 2.34 | 1.32e-01 | 0.3397986247 |
|  | 3 | 38388371 | 38388808 | PDFSTATSENSEMBLUCSC | 438 | 5 | 27719 | gene | 0.035975 | 0.0311888 | Not queried | 2.33 | 1.00e+00 | 1.0000000000 |
|  | 15 | 65368982 | 65369137 | PDFSTATSENSEMBLUCSC | 156 | 4 | 13738 | tss | 0.777180 | 0.6133794 | Not queried | 2.33 | 1.14e-01 | 0.3123457953 |
|  | 15 | 33602758 | 33602824 | PDFSTATSENSEMBLUCSC | 67 | 4 | 13555 | tss;island | 0.035994 | 0.1110814 | Not queried | 2.33 | 5.71e-02 | 0.2315877671 |
|  | 21 | 47064079 | 47064462 | PDFSTATSENSEMBLUCSC | 384 | 4 | 26762 | island | 0.066667 | 0.3104941 | Not queried | 2.33 | 4.86e-01 | 0.5640816327 |
|  | 17 | 7348316 | 7348490 | PDFSTATSENSEMBLUCSC | 175 | 8 | 17950 | island | 0.007997 | 0.0185285 | Not queried | 2.33 | 4.99e-02 | 0.2315877671 |
|  | 1 | 91182128 | 91182305 | PDFSTATSENSEMBLUCSC | 178 | 4 | 624 | gene;island | 0.240231 | 0.4773231 | Not queried | 2.33 | 6.86e-01 | 0.7223604052 |
|  | 11 | 114069864 | 114070210 | PDFSTATSENSEMBLUCSC | 347 | 6 | 6911 | gene;island | 0.537097 | 0.7947402 | Not queried | 2.33 | 1.32e-01 | 0.3397986247 |
|  | 5 | 1201359 | 1201698 | PDFSTATSENSEMBLUCSC | 340 | 9 | 31967 | tss | 0.463418 | 0.5544233 | Not queried | 2.33 | 7.30e-01 | 0.7597603748 |
|  | 2 | 119599459 | 119600002 | PDFSTATSENSEMBLUCSC | 544 | 6 | 24644 | island | 0.063512 | 0.0173191 | Not queried | 2.33 | 1.32e-01 | 0.3397986247 |
|  | 7 | 45927735 | 45928326 | PDFSTATSENSEMBLUCSC | 592 | 8 | 38477 | island | 0.030099 | 0.2631327 | Not queried | 2.33 | 1.95e-01 | 0.3826488233 |
|  | 5 | 78365647 | 78365801 | PDFSTATSENSEMBLUCSC | 155 | 5 | 31660 | gene;tss;island | 0.546893 | 0.1859017 | Not queried | 2.33 | 9.52e-02 | 0.3104222821 |
|  | 3 | 170746029 | 170746265 | PDFSTATSENSEMBLUCSC | 237 | 5 | 28717 | tss | 0.035161 | 0.0080118 | Not queried | 2.33 | 2.22e-01 | 0.3935758299 |
|  | 1 | 156182601 | 156182774 | PDFSTATSENSEMBLUCSC | 174 | 4 | 2010 | tss | 0.001203 | 0.0076596 | Not queried | 2.33 | 3.43e-01 | 0.4672865596 |
|  | 8 | 25041651 | 25041796 | PDFSTATSENSEMBLUCSC | 146 | 4 | 39606 | tss;island | 0.048215 | 0.2439939 | Not queried | 2.33 | 4.86e-01 | 0.5640816327 |
|  | 4 | 110223830 | 110224288 | PDFSTATSENSEMBLUCSC | 459 | 10 | 30542 | tss | 0.042562 | 0.2290007 | Not queried | 2.32 | 3.55e-02 | 0.2067926195 |
|  | 4 | 5710319 | 5710411 | PDFSTATSENSEMBLUCSC | 93 | 6 | 29921 | gene;tss;island | 0.066752 | 0.0141588 | Not queried | 2.32 | 6.49e-02 | 0.2477578846 |
|  | 2 | 132202427 | 132202537 | PDFSTATSENSEMBLUCSC | 111 | 6 | 24732 | island | 0.938573 | 0.6792436 | Not queried | 2.32 | 1.32e-01 | 0.3397986247 |
|  | 10 | 1228893 | 1229080 | PDFSTATSENSEMBLUCSC | 188 | 4 | 4457 | gene;island | 0.438950 | 0.8390931 | Not queried | 2.32 | 5.71e-02 | 0.2315877671 |
|  | 12 | 7260546 | 7260888 | PDFSTATSENSEMBLUCSC | 343 | 8 | 9044 | gene;tss | 0.167431 | 0.0119845 | Not queried | 2.32 | 1.04e-02 | 0.1806165940 |
|  | 5 | 1876168 | 1876397 | PDFSTATSENSEMBLUCSC | 230 | 5 | 32643 | island | 0.041730 | 0.2670145 | Not queried | 2.32 | 3.10e-01 | 0.4672865596 |
|  | 11 | 67350153 | 67350663 | PDFSTATSENSEMBLUCSC | 511 | 7 | 7497 | tss;island | 0.584071 | 0.4779931 | Not queried | 2.32 | 2.09e-01 | 0.3935758299 |
|  | 19 | 37997675 | 37997831 | PDFSTATSENSEMBLUCSC | 157 | 8 | 20740 | tss | 0.005637 | 0.0018479 | Not queried | 2.32 | 6.50e-02 | 0.2477578846 |
|  | 5 | 15499895 | 15500270 | PDFSTATSENSEMBLUCSC | 376 | 4 | 31994 | tss;island | 0.008922 | 0.0569322 | Not queried | 2.32 | 2.00e-01 | 0.3826488233 |
|  | 6 | 29817963 | 29818300 | PDFSTATSENSEMBLUCSC | 338 | 8 | 35638 | island | 0.046132 | 0.0905028 | Not queried | 2.32 | 6.50e-02 | 0.2477578846 |
|  | 7 | 156812806 | 156813602 | PDFSTATSENSEMBLUCSC | 797 | 8 | 39094 | island | 0.373166 | 0.0829976 | Not queried | 2.32 | 4.99e-02 | 0.2315877671 |
|  | 17 | 36997420 | 36997740 | PDFSTATSENSEMBLUCSC | 321 | 7 | 18261 | island | 0.565503 | 0.9234333 | Not queried | 2.32 | 1.65e-01 | 0.3826488233 |
|  | 1 | 60280716 | 60280868 | PDFSTATSENSEMBLUCSC | 153 | 4 | 559 | gene;island | 0.024878 | 0.1522178 | Not queried | 2.32 | 3.43e-01 | 0.4672865596 |
|  | 2 | 38763130 | 38763533 | PDFSTATSENSEMBLUCSC | 404 | 4 | 24230 | island | 0.022687 | 0.0095600 | Not queried | 2.31 | 4.86e-01 | 0.5640816327 |
|  | 6 | 29795815 | 29796124 | PDFSTATSENSEMBLUCSC | 310 | 9 | 33696 | gene;island | 0.056361 | 0.4262899 | Not queried | 2.31 | 5.03e-02 | 0.2315877671 |
|  | 1 | 228246632 | 228247135 | PDFSTATSENSEMBLUCSC | 504 | 4 | 1046 | gene;island | 0.219116 | 0.0530374 | Not queried | 2.31 | 2.86e-02 | 0.1830867741 |
|  | 1 | 47184738 | 47185236 | PDFSTATSENSEMBLUCSC | 499 | 5 | 1605 | tss | 0.002003 | 0.0076806 | Not queried | 2.31 | 9.52e-02 | 0.3104222821 |
|  | 1 | 31845923 | 31846110 | PDFSTATSENSEMBLUCSC | 188 | 6 | 1445 | tss | 0.012407 | 0.0580292 | Not queried | 2.31 | 4.33e-03 | 0.1459191215 |
|  | 3 | 129694392 | 129694683 | PDFSTATSENSEMBLUCSC | 292 | 4 | 27945 | gene;island | 0.061146 | 0.1730350 | Not queried | 2.31 | 3.43e-01 | 0.4672865596 |
|  | 15 | 91478847 | 91479184 | PDFSTATSENSEMBLUCSC | 338 | 4 | 13480 | gene;island | 0.025551 | 0.2712620 | Not queried | 2.31 | 2.00e-01 | 0.3826488233 |
|  | 4 | 170678860 | 170679082 | PDFSTATSENSEMBLUCSC | 223 | 5 | 30176 | gene | 0.005115 | 0.0012807 | Not queried | 2.31 | 9.52e-02 | 0.3104222821 |
|  | 22 | 50968297 | 50968516 | PDFSTATSENSEMBLUCSC | 220 | 5 | 27258 | tss | 0.014551 | 0.0041809 | Not queried | 2.31 | 4.21e-01 | 0.5404252807 |
|  | 8 | 20054372 | 20054703 | PDFSTATSENSEMBLUCSC | 332 | 9 | 40115 | island | 0.042902 | 0.0076583 | Not queried | 2.31 | 2.97e-01 | 0.4672865596 |
|  | 19 | 41531634 | 41531976 | PDFSTATSENSEMBLUCSC | 343 | 4 | 22044 | island | 0.249320 | 0.4956716 | Not queried | 2.31 | 3.43e-01 | 0.4672865596 |
|  | 17 | 56232260 | 56232674 | PDFSTATSENSEMBLUCSC | 415 | 5 | 18594 | island | 0.014781 | 0.3302219 | Not queried | 2.31 | 4.21e-01 | 0.5404252807 |
|  | 1 | 89357317 | 89357426 | PDFSTATSENSEMBLUCSC | 110 | 4 | 1736 | tss | 0.001187 | 0.0039948 | Not queried | 2.31 | 4.86e-01 | 0.5640816327 |
|  | 2 | 131594925 | 131595321 | PDFSTATSENSEMBLUCSC | 397 | 4 | 24724 | island | 0.170714 | 0.0410445 | Not queried | 2.30 | 1.14e-01 | 0.3123457953 |
|  | 6 | 161695210 | 161695561 | PDFSTATSENSEMBLUCSC | 352 | 6 | 35328 | tss | 0.221149 | 0.0227276 | Not queried | 2.30 | 3.94e-01 | 0.5236526859 |
|  | 2 | 131046122 | 131046551 | PDFSTATSENSEMBLUCSC | 430 | 4 | 24718 | island | 0.438622 | 0.7789022 | Not queried | 2.30 | 2.86e-02 | 0.1830867741 |
|  | 19 | 12203029 | 12203349 | PDFSTATSENSEMBLUCSC | 321 | 5 | 21592 | island | 0.023803 | 0.0064804 | Not queried | 2.30 | 9.52e-02 | 0.3104222821 |
|  | 19 | 50370992 | 50371215 | PDFSTATSENSEMBLUCSC | 224 | 4 | 20966 | tss;island | 0.001660 | 0.0058215 | Not queried | 2.30 | 2.86e-02 | 0.1830867741 |
|  | 6 | 29580340 | 29580367 | PDFSTATSENSEMBLUCSC | 28 | 4 | 33671 | gene | 0.674273 | 0.9120232 | Not queried | 2.30 | 1.14e-01 | 0.3123457953 |
|  | 1 | 15271830 | 15272326 | PDFSTATSENSEMBLUCSC | 497 | 6 | 241 | gene;tss | 0.254002 | 0.5285203 | Not queried | 2.30 | 2.40e-01 | 0.4133265075 |
|  | 3 | 49967462 | 49967713 | PDFSTATSENSEMBLUCSC | 252 | 6 | 28340 | tss | 0.240927 | 0.4677453 | Not queried | 2.30 | 8.18e-01 | 0.8469867211 |
|  | 22 | 30938383 | 30938784 | PDFSTATSENSEMBLUCSC | 402 | 4 | 27414 | island | 0.086682 | 0.3833000 | Not queried | 2.30 | 3.43e-01 | 0.4672865596 |
|  | 5 | 57755773 | 57756026 | PDFSTATSENSEMBLUCSC | 254 | 8 | 32815 | island | 0.008161 | 0.0140553 | Not queried | 2.30 | 1.30e-01 | 0.3397986247 |
|  | 11 | 32452038 | 32452839 | PDFSTATSENSEMBLUCSC | 802 | 8 | 6566 | gene;island | 0.128850 | 0.6609106 | Not queried | 2.30 | 4.99e-02 | 0.2315877671 |
|  | 10 | 15761192 | 15762312 | PDFSTATSENSEMBLUCSC | 1121 | 12 | 5571 | island | 0.054852 | 0.1961785 | Not queried | 2.30 | 5.19e-02 | 0.2315877671 |
|  | 6 | 170190914 | 170191082 | PDFSTATSENSEMBLUCSC | 169 | 4 | 34580 | gene;island | 0.605697 | 0.7699543 | Not queried | 2.30 | 4.86e-01 | 0.5640816327 |
|  | 12 | 53183773 | 53184086 | PDFSTATSENSEMBLUCSC | 314 | 5 | 9140 | gene;island | 0.025678 | 0.1393825 | Not queried | 2.30 | 9.52e-02 | 0.3104222821 |
|  | 10 | 133956824 | 133957095 | PDFSTATSENSEMBLUCSC | 272 | 4 | 4856 | gene;island | 0.081871 | 0.3600594 | Not queried | 2.30 | 3.43e-01 | 0.4672865596 |
|  | 3 | 58563609 | 58563693 | PDFSTATSENSEMBLUCSC | 85 | 5 | 28419 | tss | 0.051822 | 0.1739978 | Not queried | 2.30 | 1.51e-01 | 0.3618000441 |
|  | 1 | 155035008 | 155035530 | PDFSTATSENSEMBLUCSC | 523 | 5 | 1978 | tss;island | 0.024216 | 0.0451894 | Not queried | 2.29 | 3.10e-01 | 0.4672865596 |
|  | 12 | 51236582 | 51236693 | PDFSTATSENSEMBLUCSC | 112 | 4 | 9708 | tss | 0.510146 | 0.8258437 | Not queried | 2.29 | 1.14e-01 | 0.3123457953 |
|  | 10 | 99695981 | 99696260 | PDFSTATSENSEMBLUCSC | 280 | 5 | 4701 | gene | 0.448856 | 0.7980857 | Not queried | 2.29 | 4.21e-01 | 0.5404252807 |
|  | 11 | 128562003 | 128562408 | PDFSTATSENSEMBLUCSC | 406 | 7 | 7802 | tss;island | 0.048567 | 0.0955358 | Not queried | 2.29 | 2.09e-01 | 0.3935758299 |
|  | 12 | 132637734 | 132638107 | PDFSTATSENSEMBLUCSC | 374 | 4 | 11025 | island | 0.553749 | 0.7826907 | Not queried | 2.29 | 3.43e-01 | 0.4672865596 |
|  | 17 | 42081810 | 42082036 | PDFSTATSENSEMBLUCSC | 227 | 8 | 17397 | tss;island | 0.166181 | 0.0580383 | Not queried | 2.29 | 1.05e-01 | 0.3123457953 |
|  | 4 | 37961862 | 37962205 | PDFSTATSENSEMBLUCSC | 344 | 4 | 29975 | gene | 0.829249 | 0.9779467 | Not queried | 2.29 | 3.43e-01 | 0.4672865596 |
|  | 10 | 17271006 | 17271303 | PDFSTATSENSEMBLUCSC | 298 | 5 | 4518 | gene;island | 0.001654 | 0.0007974 | Not queried | 2.29 | 3.10e-01 | 0.4672865596 |
|  | 1 | 113265410 | 113265883 | PDFSTATSENSEMBLUCSC | 474 | 7 | 695 | gene;island | 0.187455 | 0.0490725 | Not queried | 2.29 | 5.30e-02 | 0.2315877671 |
|  | 5 | 19988592 | 19988944 | PDFSTATSENSEMBLUCSC | 353 | 9 | 32000 | tss | 0.022448 | 0.1554178 | Not queried | 2.29 | 1.90e-01 | 0.3826488233 |
|  | 6 | 46459694 | 46460003 | PDFSTATSENSEMBLUCSC | 310 | 6 | 36186 | island | 0.177104 | 0.0623318 | Not queried | 2.29 | 2.40e-01 | 0.4133265075 |
|  | 4 | 7755730 | 7755806 | PDFSTATSENSEMBLUCSC | 77 | 4 | 30293 | tss | 0.073800 | 0.2668002 | Not queried | 2.29 | 3.43e-01 | 0.4672865596 |
|  | 17 | 13505433 | 13505799 | PDFSTATSENSEMBLUCSC | 367 | 5 | 17144 | tss;island | 0.011624 | 0.0193536 | Not queried | 2.29 | 5.56e-02 | 0.2315877671 |
|  | 11 | 132948042 | 132948244 | PDFSTATSENSEMBLUCSC | 203 | 4 | 6984 | gene;island | 0.275655 | 0.7923465 | Not queried | 2.29 | 3.43e-01 | 0.4672865596 |
|  | 10 | 73479696 | 73479848 | PDFSTATSENSEMBLUCSC | 153 | 4 | 5144 | tss | 0.409236 | 0.8161270 | Not queried | 2.29 | 2.86e-02 | 0.1830867741 |
|  | 19 | 48908102 | 48908557 | PDFSTATSENSEMBLUCSC | 456 | 5 | 20123 | gene;island | 0.544515 | 0.8693372 | Not queried | 2.28 | 2.22e-01 | 0.3935758299 |
|  | 5 | 87974369 | 87974547 | PDFSTATSENSEMBLUCSC | 179 | 7 | 31680 | gene;tss;island | 0.491968 | 0.0506315 | Not queried | 2.28 | 4.08e-03 | 0.1459191215 |
|  | 2 | 113956255 | 113956634 | PDFSTATSENSEMBLUCSC | 380 | 4 | 22833 | gene;island | 0.742591 | 0.4761905 | Not queried | 2.28 | 2.00e-01 | 0.3826488233 |
|  | 1 | 41889271 | 41889625 | PDFSTATSENSEMBLUCSC | 355 | 4 | 3139 | island | 0.494341 | 0.8918063 | Not queried | 2.28 | 3.43e-01 | 0.4672865596 |
|  | 6 | 144607911 | 144608270 | PDFSTATSENSEMBLUCSC | 360 | 4 | 36537 | island | 0.016330 | 0.0639548 | Not queried | 2.28 | 3.43e-01 | 0.4672865596 |
|  | 20 | 1716270 | 1716632 | PDFSTATSENSEMBLUCSC | 363 | 4 | 25939 | island | 0.960920 | 0.7525547 | Not queried | 2.28 | 2.00e-01 | 0.3826488233 |
|  | 4 | 7940375 | 7940579 | PDFSTATSENSEMBLUCSC | 205 | 4 | 29943 | gene;island | 0.147430 | 0.0361974 | Not queried | 2.28 | 4.86e-01 | 0.5640816327 |
|  | 19 | 12167119 | 12167313 | PDFSTATSENSEMBLUCSC | 195 | 4 | 21590 | island | 0.660318 | 0.2585724 | Not queried | 2.28 | 1.14e-01 | 0.3123457953 |
|  | 1 | 6419606 | 6419906 | PDFSTATSENSEMBLUCSC | 301 | 4 | 170 | gene;tss | 0.006649 | 0.1346306 | Not queried | 2.28 | 4.86e-01 | 0.5640816327 |
|  | 13 | 112692119 | 112692382 | PDFSTATSENSEMBLUCSC | 264 | 4 | 11907 | island | 0.177470 | 0.5072312 | Not queried | 2.28 | 3.43e-01 | 0.4672865596 |
|  | 12 | 107349499 | 107349539 | PDFSTATSENSEMBLUCSC | 41 | 6 | 9972 | tss | 0.004174 | 0.0055862 | Not queried | 2.28 | 3.94e-01 | 0.5236526859 |
|  | 7 | 77428232 | 77428337 | PDFSTATSENSEMBLUCSC | 106 | 4 | 37061 | gene | 0.001741 | 0.0046484 | Not queried | 2.28 | 2.00e-01 | 0.3826488233 |
|  | 13 | 47126300 | 47126551 | PDFSTATSENSEMBLUCSC | 252 | 4 | 11427 | tss;island | 0.439919 | 0.2704923 | Not queried | 2.28 | 4.86e-01 | 0.5640816327 |
|  | 6 | 33241226 | 33241770 | PDFSTATSENSEMBLUCSC | 545 | 5 | 34245 | gene;island | 0.063201 | 0.4635804 | Not queried | 2.28 | 9.52e-02 | 0.3104222821 |
|  | 19 | 49631042 | 49631297 | PDFSTATSENSEMBLUCSC | 256 | 4 | 20143 | gene;island | 0.047248 | 0.0142515 | Not queried | 2.28 | 6.86e-01 | 0.7223604052 |
|  | 11 | 18477379 | 18477753 | PDFSTATSENSEMBLUCSC | 375 | 4 | 6530 | gene | 0.891719 | 0.7360134 | Not queried | 2.28 | 4.86e-01 | 0.5640816327 |
|  | 5 | 132361924 | 132362235 | PDFSTATSENSEMBLUCSC | 312 | 4 | 31749 | gene | 0.007913 | 0.0022990 | Not queried | 2.27 | 5.71e-02 | 0.2315877671 |
|  | 6 | 1384491 | 1384780 | PDFSTATSENSEMBLUCSC | 290 | 4 | 35366 | island | 0.012896 | 0.0898859 | Not queried | 2.27 | 3.43e-01 | 0.4672865596 |
|  | 1 | 7023495 | 7023864 | PDFSTATSENSEMBLUCSC | 370 | 4 | 184 | gene;island | 0.119176 | 0.0446165 | Not queried | 2.27 | 8.86e-01 | 0.8941250130 |
|  | 2 | 1746509 | 1746846 | PDFSTATSENSEMBLUCSC | 338 | 4 | 22536 | gene;island | 0.019176 | 0.0044970 | Not queried | 2.27 | 2.00e-01 | 0.3826488233 |
|  | 3 | 11034749 | 11035079 | PDFSTATSENSEMBLUCSC | 331 | 4 | 27664 | gene;island | 0.057142 | 0.2868259 | Not queried | 2.27 | 2.00e-01 | 0.3826488233 |
|  | 15 | 74420511 | 74420890 | PDFSTATSENSEMBLUCSC | 380 | 4 | 13414 | gene;tss;island | 0.216305 | 0.0161838 | Not queried | 2.27 | 3.43e-01 | 0.4672865596 |
|  | 12 | 123351548 | 123351868 | PDFSTATSENSEMBLUCSC | 321 | 5 | 9374 | gene;island | 0.967463 | 0.9092432 | Not queried | 2.27 | 9.52e-02 | 0.3104222821 |
|  | 11 | 18477153 | 18477753 | PDFSTATSENSEMBLUCSC | 601 | 9 | 8111 | island | 0.852075 | 0.5435392 | Not queried | 2.27 | 2.97e-01 | 0.4672865596 |
|  | 5 | 131892242 | 131892723 | PDFSTATSENSEMBLUCSC | 482 | 7 | 33093 | island | 0.007403 | 0.0300324 | Not queried | 2.27 | 1.65e-01 | 0.3826488233 |
|  | 14 | 101530343 | 101530856 | PDFSTATSENSEMBLUCSC | 514 | 6 | 12633 | tss;island | 0.183086 | 0.3710628 | Not queried | 2.27 | 5.89e-01 | 0.6570484697 |
|  | 22 | 33196328 | 33196627 | PDFSTATSENSEMBLUCSC | 300 | 5 | 26850 | gene;tss;island | 0.166764 | 0.5415209 | Not queried | 2.27 | 1.51e-01 | 0.3618000441 |
|  | 12 | 107349499 | 107349641 | PDFSTATSENSEMBLUCSC | 143 | 7 | 10757 | island | 0.004750 | 0.0057917 | Not queried | 2.27 | 5.35e-01 | 0.6176455124 |
|  | 16 | 49316845 | 49317262 | PDFSTATSENSEMBLUCSC | 418 | 5 | 15907 | island | 0.031534 | 0.0940601 | Not queried | 2.27 | 3.10e-01 | 0.4672865596 |
|  | 10 | 134043618 | 134043897 | PDFSTATSENSEMBLUCSC | 280 | 5 | 4858 | gene;island | 0.021277 | 0.1727682 | Not queried | 2.27 | 3.17e-02 | 0.1866936846 |
|  | 11 | 63775071 | 63775612 | PDFSTATSENSEMBLUCSC | 542 | 5 | 6658 | gene;island | 0.115649 | 0.0902568 | Not queried | 2.27 | 5.48e-01 | 0.6179718485 |
|  | 12 | 54070891 | 54071194 | PDFSTATSENSEMBLUCSC | 304 | 7 | 9746 | tss;island | 0.012344 | 0.1199990 | Not queried | 2.27 | 1.11e-02 | 0.1821650572 |
|  | 5 | 170736021 | 170736277 | PDFSTATSENSEMBLUCSC | 257 | 7 | 32486 | tss | 0.358178 | 0.1378274 | Not queried | 2.27 | 1.28e-01 | 0.3397986247 |
|  | 6 | 108488751 | 108489064 | PDFSTATSENSEMBLUCSC | 314 | 4 | 34438 | gene;island | 0.046108 | 0.3524716 | Not queried | 2.27 | 3.43e-01 | 0.4672865596 |
|  | 10 | 97850047 | 97850343 | PDFSTATSENSEMBLUCSC | 297 | 5 | 5971 | island | 0.002785 | 0.0053435 | Not queried | 2.27 | 5.48e-01 | 0.6179718485 |
|  | 11 | 44087367 | 44087808 | PDFSTATSENSEMBLUCSC | 442 | 9 | 7259 | tss | 0.015183 | 0.0375473 | Not queried | 2.27 | 7.70e-02 | 0.2825346647 |
|  | 17 | 32905966 | 32906622 | PDFSTATSENSEMBLUCSC | 657 | 7 | 18201 | island | 0.005154 | 0.0092495 | Not queried | 2.27 | 5.35e-01 | 0.6176455124 |
|  | 1 | 201617847 | 201618284 | PDFSTATSENSEMBLUCSC | 438 | 6 | 940 | gene;island | 0.172463 | 0.5816383 | Not queried | 2.27 | 2.40e-01 | 0.4133265075 |
|  | 20 | 17549599 | 17549955 | PDFSTATSENSEMBLUCSC | 357 | 5 | 25608 | tss;island | 0.187214 | 0.0751218 | Not queried | 2.27 | 1.51e-01 | 0.3618000441 |
|  | 1 | 221060753 | 221061254 | PDFSTATSENSEMBLUCSC | 502 | 6 | 4224 | island | 0.079963 | 0.3133908 | Not queried | 2.26 | 8.66e-03 | 0.1595382395 |
|  | 17 | 74868561 | 74868725 | PDFSTATSENSEMBLUCSC | 165 | 4 | 16798 | gene;tss;island | 0.010349 | 0.0260256 | Not queried | 2.26 | 4.86e-01 | 0.5640816327 |
|  | 2 | 3642098 | 3642634 | PDFSTATSENSEMBLUCSC | 537 | 6 | 23163 | tss | 0.959802 | 0.7949537 | Not queried | 2.26 | 9.31e-02 | 0.3104222821 |
|  | 5 | 122434853 | 122435202 | PDFSTATSENSEMBLUCSC | 350 | 4 | 31732 | gene;island | 0.053461 | 0.1759999 | Not queried | 2.26 | 6.86e-01 | 0.7223604052 |
|  | 6 | 26183508 | 26184245 | PDFSTATSENSEMBLUCSC | 738 | 12 | 35521 | island | 0.046651 | 0.0192424 | Not queried | 2.26 | 1.60e-01 | 0.3814043454 |
|  | 6 | 108436115 | 108436519 | PDFSTATSENSEMBLUCSC | 405 | 4 | 36390 | island | 0.009737 | 0.0458079 | Not queried | 2.26 | 2.00e-01 | 0.3826488233 |
|  | 1 | 47902085 | 47902371 | PDFSTATSENSEMBLUCSC | 287 | 5 | 518 | gene;island | 0.011032 | 0.0056193 | Not queried | 2.26 | 2.22e-01 | 0.3935758299 |
|  | 2 | 30144376 | 30144838 | PDFSTATSENSEMBLUCSC | 463 | 7 | 24199 | island | 0.013654 | 0.0051273 | Not queried | 2.26 | 2.09e-01 | 0.3935758299 |
|  | 1 | 98515041 | 98515272 | PDFSTATSENSEMBLUCSC | 232 | 4 | 3515 | island | 0.014148 | 0.0064386 | Not queried | 2.26 | 3.43e-01 | 0.4672865596 |
|  | 19 | 3671903 | 3672411 | PDFSTATSENSEMBLUCSC | 509 | 4 | 19719 | gene;island | 0.051896 | 0.1345629 | Not queried | 2.26 | 2.00e-01 | 0.3826488233 |
|  | 1 | 156215418 | 156215727 | PDFSTATSENSEMBLUCSC | 310 | 4 | 818 | gene;island | 0.024103 | 0.0231470 | Not queried | 2.26 | 8.86e-01 | 0.8941250130 |
|  | 4 | 174443242 | 174443567 | PDFSTATSENSEMBLUCSC | 326 | 4 | 31427 | island | 0.120550 | 0.3213410 | Not queried | 2.26 | 1.14e-01 | 0.3123457953 |
|  | 11 | 65409760 | 65410126 | PDFSTATSENSEMBLUCSC | 367 | 4 | 6715 | gene;island | 0.966808 | 0.7309103 | Not queried | 2.26 | 4.86e-01 | 0.5640816327 |
|  | 6 | 32784097 | 32784676 | PDFSTATSENSEMBLUCSC | 580 | 5 | 34135 | gene | 0.006897 | 0.0022263 | Not queried | 2.26 | 2.22e-01 | 0.3935758299 |
|  | 7 | 100493710 | 100493849 | PDFSTATSENSEMBLUCSC | 140 | 4 | 37795 | tss | 0.004864 | 0.0301463 | Not queried | 2.26 | 1.14e-01 | 0.3123457953 |
|  | 5 | 135364827 | 135365012 | PDFSTATSENSEMBLUCSC | 186 | 4 | 31761 | gene;island | 0.299426 | 0.5659787 | Not queried | 2.25 | 2.00e-01 | 0.3826488233 |
|  | 17 | 43047405 | 43047971 | PDFSTATSENSEMBLUCSC | 567 | 5 | 18418 | island | 0.009422 | 0.0102568 | Not queried | 2.25 | 4.21e-01 | 0.5404252807 |
|  | 4 | 164087672 | 164088022 | PDFSTATSENSEMBLUCSC | 351 | 4 | 30166 | gene | 0.004986 | 0.0020024 | Not queried | 2.25 | 2.00e-01 | 0.3826488233 |
|  | 3 | 157824148 | 157824283 | PDFSTATSENSEMBLUCSC | 136 | 5 | 28682 | tss;island | 0.039689 | 0.0074809 | Not queried | 2.25 | 2.22e-01 | 0.3935758299 |
|  | 6 | 10419835 | 10420079 | PDFSTATSENSEMBLUCSC | 245 | 7 | 34625 | tss | 0.380276 | 0.0641276 | Not queried | 2.25 | 2.09e-01 | 0.3935758299 |
|  | 1 | 3663164 | 3664084 | PDFSTATSENSEMBLUCSC | 921 | 10 | 2570 | island | 0.047230 | 0.0078903 | Not queried | 2.25 | 7.53e-02 | 0.2795801356 |
|  | 11 | 15095017 | 15095070 | PDFSTATSENSEMBLUCSC | 54 | 5 | 7168 | tss | 0.202698 | 0.0258988 | Not queried | 2.25 | 5.56e-02 | 0.2315877671 |
|  | 2 | 84743142 | 84743573 | PDFSTATSENSEMBLUCSC | 432 | 9 | 23475 | tss | 0.046898 | 0.0185170 | Not queried | 2.25 | 7.70e-02 | 0.2825346647 |
|  | 20 | 10200138 | 10200617 | PDFSTATSENSEMBLUCSC | 480 | 5 | 25357 | gene;island | 0.007760 | 0.0133065 | Not queried | 2.25 | 6.90e-01 | 0.7223604052 |
|  | 7 | 1967621 | 1967976 | PDFSTATSENSEMBLUCSC | 356 | 5 | 36773 | gene;island | 0.926044 | 0.5498361 | Not queried | 2.25 | 5.56e-02 | 0.2315877671 |
|  | 7 | 27153580 | 27153944 | PDFSTATSENSEMBLUCSC | 365 | 7 | 36930 | gene;island | 0.839740 | 0.9500709 | Not queried | 2.25 | 2.59e-01 | 0.4381244265 |
|  | 2 | 133175192 | 133175397 | PDFSTATSENSEMBLUCSC | 206 | 4 | 22875 | gene;island | 0.313894 | 0.7541624 | Not queried | 2.25 | 2.00e-01 | 0.3826488233 |
|  | 3 | 32859377 | 32859445 | PDFSTATSENSEMBLUCSC | 69 | 5 | 28208 | tss | 0.097327 | 0.0230958 | Not queried | 2.25 | 7.94e-03 | 0.1589602024 |
|  | 12 | 568798 | 569221 | PDFSTATSENSEMBLUCSC | 424 | 5 | 9479 | tss;island | 0.134860 | 0.0519740 | Not queried | 2.25 | 3.10e-01 | 0.4672865596 |
|  | 16 | 58498456 | 58498754 | PDFSTATSENSEMBLUCSC | 299 | 7 | 15274 | tss | 0.120966 | 0.0118378 | Not queried | 2.24 | 7.28e-02 | 0.2713481487 |
|  | 19 | 14016603 | 14016822 | PDFSTATSENSEMBLUCSC | 220 | 7 | 19887 | gene | 0.022725 | 0.1228958 | Not queried | 2.24 | 8.05e-01 | 0.8347790563 |
|  | 6 | 160679974 | 160680213 | PDFSTATSENSEMBLUCSC | 240 | 8 | 35327 | tss | 0.333539 | 0.6611111 | Not queried | 2.24 | 4.99e-02 | 0.2315877671 |
|  | 8 | 144367072 | 144367553 | PDFSTATSENSEMBLUCSC | 482 | 4 | 40651 | island | 0.512824 | 0.8200622 | Not queried | 2.24 | 3.43e-01 | 0.4672865596 |
|  | 20 | 44098223 | 44098409 | PDFSTATSENSEMBLUCSC | 187 | 7 | 26200 | island | 0.126234 | 0.1518501 | Not queried | 2.24 | 3.18e-01 | 0.4672865596 |
|  | 4 | 187111841 | 187112145 | PDFSTATSENSEMBLUCSC | 305 | 5 | 30705 | tss;island | 0.006144 | 0.0014688 | Not queried | 2.24 | 9.52e-02 | 0.3104222821 |
|  | 7 | 15726348 | 15726821 | PDFSTATSENSEMBLUCSC | 474 | 7 | 37450 | tss | 0.007130 | 0.0097541 | Not queried | 2.24 | 3.83e-01 | 0.5168893301 |
|  | 20 | 2796729 | 2796872 | PDFSTATSENSEMBLUCSC | 144 | 4 | 25552 | tss | 0.254124 | 0.5918625 | Not queried | 2.24 | 3.43e-01 | 0.4672865596 |
|  | 14 | 99736387 | 99736559 | PDFSTATSENSEMBLUCSC | 173 | 4 | 12202 | gene;island | 0.015627 | 0.0243118 | Not queried | 2.24 | 6.86e-01 | 0.7223604052 |
|  | 3 | 30647378 | 30648098 | PDFSTATSENSEMBLUCSC | 721 | 13 | 28957 | island | 0.014097 | 0.0504448 | Not queried | 2.24 | 1.39e-01 | 0.3560599333 |
|  | 3 | 120003547 | 120003884 | PDFSTATSENSEMBLUCSC | 338 | 5 | 29387 | island | 0.406864 | 0.5333307 | Not queried | 2.24 | 2.22e-01 | 0.3935758299 |
|  | 16 | 979898 | 980146 | PDFSTATSENSEMBLUCSC | 249 | 4 | 14555 | gene;island | 0.633402 | 0.9114010 | Not queried | 2.24 | 1.14e-01 | 0.3123457953 |
|  | 15 | 26108391 | 26108412 | PDFSTATSENSEMBLUCSC | 22 | 5 | 13538 | tss | 0.007271 | 0.0340858 | Not queried | 2.23 | 7.94e-03 | 0.1589602024 |
|  | 22 | 29426511 | 29426832 | PDFSTATSENSEMBLUCSC | 322 | 4 | 26829 | gene;island | 0.007202 | 0.0621209 | Not queried | 2.23 | 3.43e-01 | 0.4672865596 |
|  | 12 | 8995557 | 8995965 | PDFSTATSENSEMBLUCSC | 409 | 5 | 9050 | gene | 0.741236 | 0.9407724 | Not queried | 2.23 | 4.21e-01 | 0.5404252807 |
|  | 5 | 95768997 | 95769079 | PDFSTATSENSEMBLUCSC | 83 | 6 | 32189 | tss | 0.403526 | 0.1761539 | Not queried | 2.23 | 9.31e-02 | 0.3104222821 |
|  | 15 | 80543353 | 80543685 | PDFSTATSENSEMBLUCSC | 333 | 5 | 14374 | island | 0.216735 | 0.6076228 | Not queried | 2.23 | 3.10e-01 | 0.4672865596 |
|  | 19 | 49259215 | 49259452 | PDFSTATSENSEMBLUCSC | 238 | 4 | 20927 | tss;island | 0.023174 | 0.2019228 | Not queried | 2.23 | 3.43e-01 | 0.4672865596 |
|  | 4 | 57410776 | 57411219 | PDFSTATSENSEMBLUCSC | 444 | 5 | 31069 | island | 0.009018 | 0.0422662 | Not queried | 2.23 | 3.10e-01 | 0.4672865596 |
|  | 6 | 31459698 | 31460088 | PDFSTATSENSEMBLUCSC | 391 | 6 | 35773 | island | 0.027191 | 0.0120101 | Not queried | 2.23 | 3.10e-01 | 0.4672865596 |
|  | 15 | 65368982 | 65369213 | PDFSTATSENSEMBLUCSC | 232 | 5 | 14226 | island | 0.867640 | 0.6429829 | Not queried | 2.23 | 2.22e-01 | 0.3935758299 |
|  | 11 | 118436807 | 118437133 | PDFSTATSENSEMBLUCSC | 327 | 9 | 7735 | tss | 0.003320 | 0.0102620 | Not queried | 2.23 | 6.05e-01 | 0.6747992494 |
|  | 1 | 848379 | 848609 | PDFSTATSENSEMBLUCSC | 231 | 4 | 2378 | island | 0.217945 | 0.4279101 | Not queried | 2.23 | 2.00e-01 | 0.3826488233 |
|  | 6 | 108444791 | 108444920 | PDFSTATSENSEMBLUCSC | 130 | 4 | 36392 | island | 0.541542 | 0.3118909 | Not queried | 2.23 | 3.43e-01 | 0.4672865596 |
|  | 7 | 156795767 | 156796145 | PDFSTATSENSEMBLUCSC | 379 | 5 | 39090 | island | 0.343458 | 0.1553366 | Not queried | 2.23 | 2.22e-01 | 0.3935758299 |
|  | 9 | 99382084 | 99382235 | PDFSTATSENSEMBLUCSC | 152 | 4 | 40917 | island | 0.012614 | 0.0267356 | Not queried | 2.23 | 1.14e-01 | 0.3123457953 |
|  | 11 | 811545 | 811669 | PDFSTATSENSEMBLUCSC | 125 | 4 | 7030 | tss | 0.801168 | 0.9515020 | Not queried | 2.23 | 2.00e-01 | 0.3826488233 |
|  | 1 | 113216774 | 113217043 | PDFSTATSENSEMBLUCSC | 270 | 5 | 1837 | tss | 0.025774 | 0.0117046 | Not queried | 2.23 | 3.10e-01 | 0.4672865596 |
|  | 1 | 95392925 | 95393138 | PDFSTATSENSEMBLUCSC | 214 | 4 | 1767 | tss;island | 0.088006 | 0.0397724 | Not queried | 2.23 | 2.00e-01 | 0.3826488233 |
|  | 11 | 58830191 | 58830610 | PDFSTATSENSEMBLUCSC | 420 | 4 | 8300 | island | 0.795788 | 0.5427661 | Not queried | 2.23 | 3.43e-01 | 0.4672865596 |
|  | 17 | 38375119 | 38375510 | PDFSTATSENSEMBLUCSC | 392 | 7 | 17327 | tss;island | 0.007784 | 0.0015793 | Not queried | 2.23 | 5.30e-02 | 0.2315877671 |
|  | 2 | 162272107 | 162272604 | PDFSTATSENSEMBLUCSC | 498 | 6 | 23687 | tss | 0.036863 | 0.0521284 | Not queried | 2.23 | 3.94e-01 | 0.5236526859 |
|  | 6 | 167031273 | 167031411 | PDFSTATSENSEMBLUCSC | 139 | 4 | 34549 | gene;island | 0.848252 | 0.9542949 | Not queried | 2.22 | 3.43e-01 | 0.4672865596 |
|  | 16 | 959749 | 960144 | PDFSTATSENSEMBLUCSC | 396 | 4 | 14550 | gene;island | 0.817712 | 0.9584398 | Not queried | 2.22 | 1.14e-01 | 0.3123457953 |
|  | 1 | 173639044 | 173639135 | PDFSTATSENSEMBLUCSC | 92 | 4 | 2097 | tss;island | 0.464771 | 0.1812384 | Not queried | 2.22 | 3.43e-01 | 0.4672865596 |
|  | 8 | 599963 | 600555 | PDFSTATSENSEMBLUCSC | 593 | 6 | 40003 | island | 0.753005 | 0.9664128 | Not queried | 2.22 | 2.60e-02 | 0.1830867741 |
|  | 13 | 36871646 | 36871962 | PDFSTATSENSEMBLUCSC | 317 | 6 | 11129 | gene | 0.050261 | 0.2166580 | Not queried | 2.22 | 9.31e-02 | 0.3104222821 |
|  | 16 | 2653222 | 2653306 | PDFSTATSENSEMBLUCSC | 85 | 7 | 15032 | tss | 0.009520 | 0.0076578 | Not queried | 2.22 | 2.09e-01 | 0.3935758299 |
|  | 11 | 115024362 | 115024626 | PDFSTATSENSEMBLUCSC | 265 | 4 | 8826 | island | 0.569389 | 0.8451969 | Not queried | 2.22 | 2.00e-01 | 0.3826488233 |
|  | 19 | 51524658 | 51524828 | PDFSTATSENSEMBLUCSC | 171 | 4 | 20991 | tss;island | 0.126573 | 0.0176172 | Not queried | 2.22 | 8.86e-01 | 0.8941250130 |
|  | 19 | 41317837 | 41318235 | PDFSTATSENSEMBLUCSC | 399 | 4 | 22043 | island | 0.028557 | 0.1319943 | Not queried | 2.22 | 8.86e-01 | 0.8941250130 |
|  | 3 | 189838996 | 189839473 | PDFSTATSENSEMBLUCSC | 478 | 7 | 28081 | gene;tss;island | 0.876566 | 0.6098152 | Not queried | 2.22 | 3.83e-01 | 0.5168893301 |
|  | 3 | 184972080 | 184972153 | PDFSTATSENSEMBLUCSC | 74 | 4 | 28766 | tss;island | 0.283849 | 0.4331436 | Not queried | 2.22 | 8.86e-01 | 0.8941250130 |
|  | 7 | 141438205 | 141438468 | PDFSTATSENSEMBLUCSC | 264 | 4 | 37252 | gene | 0.004090 | 0.0022067 | Not queried | 2.22 | 4.86e-01 | 0.5640816327 |
|  | 20 | 61809557 | 61809841 | PDFSTATSENSEMBLUCSC | 285 | 7 | 25889 | tss | 0.063572 | 0.2519511 | Not queried | 2.22 | 1.65e-01 | 0.3826488233 |
|  | 11 | 107730432 | 107730551 | PDFSTATSENSEMBLUCSC | 120 | 4 | 7665 | tss;island | 0.004257 | 0.0131558 | Not queried | 2.22 | 3.43e-01 | 0.4672865596 |
|  | 19 | 49926586 | 49927309 | PDFSTATSENSEMBLUCSC | 724 | 7 | 22264 | island | 0.044329 | 0.1391527 | Not queried | 2.22 | 5.30e-02 | 0.2315877671 |
|  | 19 | 11494980 | 11495295 | PDFSTATSENSEMBLUCSC | 316 | 4 | 21566 | island | 0.381964 | 0.0512136 | Not queried | 2.22 | 4.86e-01 | 0.5640816327 |
|  | 6 | 40345376 | 40345751 | PDFSTATSENSEMBLUCSC | 376 | 4 | 34961 | tss | 0.415097 | 0.6647332 | Not queried | 2.22 | 3.43e-01 | 0.4672865596 |
|  | 8 | 143781297 | 143781406 | PDFSTATSENSEMBLUCSC | 110 | 4 | 39933 | tss;island | 0.085669 | 0.0879656 | Not queried | 2.22 | 6.86e-01 | 0.7223604052 |
|  | 3 | 154042288 | 154042778 | PDFSTATSENSEMBLUCSC | 491 | 9 | 28668 | tss | 0.012358 | 0.0276448 | Not queried | 2.22 | 2.58e-01 | 0.4381244265 |
|  | 10 | 102890886 | 102891080 | PDFSTATSENSEMBLUCSC | 195 | 8 | 5303 | tss;island | 0.054699 | 0.0314488 | Not queried | 2.22 | 2.79e-01 | 0.4672865596 |
|  | 2 | 234847554 | 234847856 | PDFSTATSENSEMBLUCSC | 303 | 4 | 23079 | gene;island | 0.156535 | 0.0136238 | Not queried | 2.22 | 3.43e-01 | 0.4672865596 |
|  | 19 | 44952417 | 44952618 | PDFSTATSENSEMBLUCSC | 202 | 4 | 20083 | gene | 0.038431 | 0.1263621 | Not queried | 2.21 | 3.43e-01 | 0.4672865596 |
|  | 1 | 42618891 | 42619087 | PDFSTATSENSEMBLUCSC | 197 | 4 | 1546 | tss | 0.353901 | 0.6021706 | Not queried | 2.21 | 2.00e-01 | 0.3826488233 |
|  | 11 | 66045351 | 66045718 | PDFSTATSENSEMBLUCSC | 368 | 9 | 8501 | island | 0.003566 | 0.0054310 | Not queried | 2.21 | 1.13e-01 | 0.3123457953 |
|  | 19 | 12228348 | 12228618 | PDFSTATSENSEMBLUCSC | 271 | 5 | 21593 | island | 0.761009 | 0.9413005 | Not queried | 2.21 | 1.51e-01 | 0.3618000441 |
|  | 6 | 53530538 | 53530628 | PDFSTATSENSEMBLUCSC | 91 | 5 | 35041 | tss | 0.567449 | 0.8614295 | Not queried | 2.21 | 3.17e-02 | 0.1866936846 |
|  | 16 | 680872 | 681057 | PDFSTATSENSEMBLUCSC | 186 | 4 | 15465 | island | 0.446212 | 0.0477619 | Not queried | 2.21 | 3.43e-01 | 0.4672865596 |
|  | 3 | 138067350 | 138067409 | PDFSTATSENSEMBLUCSC | 60 | 4 | 28622 | tss | 0.007718 | 0.0015335 | Not queried | 2.21 | 3.43e-01 | 0.4672865596 |
|  | 10 | 71892694 | 71892787 | PDFSTATSENSEMBLUCSC | 94 | 6 | 5130 | tss | 0.006243 | 0.1497056 | Not queried | 2.21 | 9.31e-02 | 0.3104222821 |
|  | 11 | 624457 | 625147 | PDFSTATSENSEMBLUCSC | 691 | 8 | 7869 | island | 0.541067 | 0.8818389 | Not queried | 2.21 | 8.30e-02 | 0.3028612233 |
|  | 17 | 6797466 | 6797771 | PDFSTATSENSEMBLUCSC | 306 | 4 | 16379 | gene;island | 0.037121 | 0.2010040 | Not queried | 2.21 | 2.00e-01 | 0.3826488233 |
|  | 17 | 46114574 | 46114933 | PDFSTATSENSEMBLUCSC | 360 | 7 | 16649 | gene;island | 0.033107 | 0.1354282 | Not queried | 2.21 | 3.18e-01 | 0.4672865596 |
|  | 15 | 102264738 | 102264772 | PDFSTATSENSEMBLUCSC | 35 | 5 | 13936 | tss;island | 0.000653 | 0.0029676 | Not queried | 2.21 | 1.51e-01 | 0.3618000441 |
|  | 1 | 95006322 | 95006977 | PDFSTATSENSEMBLUCSC | 656 | 6 | 643 | gene;island | 0.644840 | 0.7562703 | Not queried | 2.21 | 4.85e-01 | 0.5640816327 |
|  | 4 | 77172617 | 77172841 | PDFSTATSENSEMBLUCSC | 225 | 6 | 30447 | tss | 0.008970 | 0.0038725 | Not queried | 2.21 | 1.32e-01 | 0.3397986247 |
|  | 8 | 67344553 | 67344720 | PDFSTATSENSEMBLUCSC | 168 | 7 | 40354 | island | 0.007639 | 0.0787184 | Not queried | 2.21 | 1.28e-01 | 0.3397986247 |
|  | 18 | 48085671 | 48085844 | PDFSTATSENSEMBLUCSC | 174 | 4 | 19275 | tss;island | 0.028170 | 0.1581170 | Not queried | 2.21 | 1.14e-01 | 0.3123457953 |
|  | 3 | 48700375 | 48700498 | PDFSTATSENSEMBLUCSC | 124 | 6 | 28305 | tss | 0.079189 | 0.0178739 | Not queried | 2.21 | 1.80e-01 | 0.3826488233 |
|  | 3 | 40428713 | 40429014 | PDFSTATSENSEMBLUCSC | 302 | 4 | 27727 | gene | 0.050220 | 0.3417998 | Not queried | 2.21 | 2.00e-01 | 0.3826488233 |
|  | 8 | 42234724 | 42234975 | PDFSTATSENSEMBLUCSC | 252 | 5 | 39678 | tss | 0.166024 | 0.4783922 | Not queried | 2.20 | 8.41e-01 | 0.8635565145 |
|  | 4 | 154216535 | 154217214 | PDFSTATSENSEMBLUCSC | 680 | 6 | 30154 | gene;island | 0.694154 | 0.9461747 | Not queried | 2.20 | 4.11e-02 | 0.2180389943 |
|  | 16 | 66400320 | 66400411 | PDFSTATSENSEMBLUCSC | 92 | 4 | 15279 | tss | 0.224469 | 0.5372098 | Not queried | 2.20 | 2.00e-01 | 0.3826488233 |
|  | 16 | 745663 | 745765 | PDFSTATSENSEMBLUCSC | 103 | 4 | 14539 | gene;island | 0.090690 | 0.0249632 | Not queried | 2.20 | 2.86e-02 | 0.1830867741 |
|  | 11 | 117947459 | 117947657 | PDFSTATSENSEMBLUCSC | 199 | 5 | 7725 | tss | 0.444355 | 0.6383892 | Not queried | 2.20 | 1.51e-01 | 0.3618000441 |
|  | 16 | 58498456 | 58498818 | PDFSTATSENSEMBLUCSC | 363 | 8 | 14772 | gene;island | 0.085940 | 0.0137775 | Not queried | 2.20 | 1.30e-01 | 0.3397986247 |
|  | 12 | 2788627 | 2788847 | PDFSTATSENSEMBLUCSC | 221 | 5 | 9005 | gene;island | 0.693635 | 0.8569039 | Not queried | 2.20 | 4.21e-01 | 0.5404252807 |
|  | 4 | 54457737 | 54458246 | PDFSTATSENSEMBLUCSC | 510 | 6 | 30397 | tss | 0.121072 | 0.0624863 | Not queried | 2.20 | 2.40e-01 | 0.4133265075 |
|  | 1 | 94703343 | 94703507 | PDFSTATSENSEMBLUCSC | 165 | 5 | 1763 | tss | 0.105000 | 0.0394629 | Not queried | 2.20 | 4.21e-01 | 0.5404252807 |
|  | 17 | 36997420 | 36997627 | PDFSTATSENSEMBLUCSC | 208 | 4 | 16542 | gene | 0.560683 | 0.9525755 | Not queried | 2.20 | 4.86e-01 | 0.5640816327 |
|  | 4 | 47033126 | 47033274 | PDFSTATSENSEMBLUCSC | 149 | 5 | 30376 | tss | 0.071627 | 0.0497760 | Not queried | 2.20 | 6.90e-01 | 0.7223604052 |
|  | 4 | 40751717 | 40752151 | PDFSTATSENSEMBLUCSC | 435 | 6 | 30997 | island | 0.219481 | 0.0409574 | Not queried | 2.20 | 6.49e-02 | 0.2477578846 |
|  | 7 | 44579245 | 44579475 | PDFSTATSENSEMBLUCSC | 231 | 5 | 36993 | gene | 0.274819 | 0.6754242 | Not queried | 2.20 | 1.51e-01 | 0.3618000441 |
|  | 1 | 201368738 | 201369031 | PDFSTATSENSEMBLUCSC | 294 | 8 | 2166 | tss | 0.197711 | 0.0313311 | Not queried | 2.20 | 6.99e-03 | 0.1589602024 |
|  | 6 | 32365491 | 32366096 | PDFSTATSENSEMBLUCSC | 606 | 7 | 34107 | gene | 0.125887 | 0.0142202 | Not queried | 2.19 | 1.28e-01 | 0.3397986247 |
|  | 6 | 11111894 | 11112237 | PDFSTATSENSEMBLUCSC | 344 | 5 | 33569 | gene | 0.863359 | 0.5301021 | Not queried | 2.19 | 1.51e-01 | 0.3618000441 |
|  | 18 | 63417605 | 63417783 | PDFSTATSENSEMBLUCSC | 179 | 4 | 19122 | gene;tss;island | 0.031992 | 0.1461462 | Not queried | 2.19 | 4.86e-01 | 0.5640816327 |
|  | 12 | 50349173 | 50349522 | PDFSTATSENSEMBLUCSC | 350 | 4 | 9126 | gene;island | 0.144249 | 0.5020661 | Not queried | 2.19 | 3.43e-01 | 0.4672865596 |
|  | 11 | 417540 | 417643 | PDFSTATSENSEMBLUCSC | 104 | 4 | 7011 | tss;island | 0.597724 | 0.2951342 | Not queried | 2.19 | 3.43e-01 | 0.4672865596 |
|  | 12 | 132896199 | 132896939 | PDFSTATSENSEMBLUCSC | 741 | 9 | 9447 | gene;island | 0.541334 | 0.8478458 | Not queried | 2.19 | 1.36e-01 | 0.3491249184 |
|  | 16 | 84029457 | 84029838 | PDFSTATSENSEMBLUCSC | 382 | 5 | 14862 | gene;island | 0.025733 | 0.0268502 | Not queried | 2.19 | 6.90e-01 | 0.7223604052 |
|  | 19 | 58520847 | 58521273 | PDFSTATSENSEMBLUCSC | 427 | 6 | 22490 | island | 0.102892 | 0.0366759 | Not queried | 2.19 | 9.31e-02 | 0.3104222821 |
|  | 3 | 184243086 | 184243411 | PDFSTATSENSEMBLUCSC | 326 | 4 | 29729 | island | 0.003756 | 0.0582942 | Not queried | 2.19 | 4.86e-01 | 0.5640816327 |
|  | 13 | 111642443 | 111642660 | PDFSTATSENSEMBLUCSC | 218 | 4 | 11893 | island | 0.390952 | 0.7327555 | Not queried | 2.19 | 2.86e-02 | 0.1830867741 |
|  | 1 | 2324205 | 2324396 | PDFSTATSENSEMBLUCSC | 192 | 4 | 81 | gene;tss;island | 0.960961 | 0.8453853 | Not queried | 2.19 | 3.43e-01 | 0.4672865596 |
|  | 11 | 92702373 | 92702663 | PDFSTATSENSEMBLUCSC | 291 | 7 | 7617 | tss;island | 0.210741 | 0.5556101 | Not queried | 2.19 | 3.79e-02 | 0.2121545571 |
|  | 11 | 20044428 | 20044639 | PDFSTATSENSEMBLUCSC | 212 | 4 | 6536 | gene | 0.530973 | 0.8696106 | Not queried | 2.19 | 4.86e-01 | 0.5640816327 |
|  | 1 | 854766 | 855046 | PDFSTATSENSEMBLUCSC | 281 | 7 | 2379 | island | 0.316510 | 0.7244037 | Not queried | 2.19 | 5.83e-04 | 0.0548331102 |
|  | 10 | 86001154 | 86001418 | PDFSTATSENSEMBLUCSC | 265 | 6 | 5896 | island | 0.204161 | 0.4056413 | Not queried | 2.19 | 2.40e-01 | 0.4133265075 |
|  | 17 | 46698881 | 46699155 | PDFSTATSENSEMBLUCSC | 275 | 4 | 16661 | gene;island | 0.252180 | 0.6846486 | Not queried | 2.19 | 2.00e-01 | 0.3826488233 |
|  | 12 | 59313279 | 59313517 | PDFSTATSENSEMBLUCSC | 239 | 4 | 9198 | gene;island | 0.005035 | 0.0023348 | Not queried | 2.19 | 6.86e-01 | 0.7223604052 |
|  | 19 | 3869086 | 3869599 | PDFSTATSENSEMBLUCSC | 514 | 6 | 20353 | tss;island | 0.190991 | 0.4252011 | Not queried | 2.18 | 5.89e-01 | 0.6570484697 |
|  | 7 | 98248852 | 98249103 | PDFSTATSENSEMBLUCSC | 252 | 5 | 37114 | gene;island | 0.055753 | 0.0361054 | Not queried | 2.18 | 4.21e-01 | 0.5404252807 |
|  | 8 | 88884985 | 88885254 | PDFSTATSENSEMBLUCSC | 270 | 4 | 39355 | gene;island | 0.180071 | 0.5007843 | Not queried | 2.18 | 4.86e-01 | 0.5640816327 |
|  | 20 | 62688573 | 62688896 | PDFSTATSENSEMBLUCSC | 324 | 4 | 25516 | gene;island | 0.137279 | 0.3888901 | Not queried | 2.18 | 2.00e-01 | 0.3826488233 |
|  | 22 | 19701734 | 19701871 | PDFSTATSENSEMBLUCSC | 138 | 4 | 26963 | tss;island | 0.021544 | 0.0058340 | Not queried | 2.18 | 1.14e-01 | 0.3123457953 |
|  | 12 | 130918514 | 130918994 | PDFSTATSENSEMBLUCSC | 481 | 5 | 9414 | gene | 0.534178 | 0.7017579 | Not queried | 2.18 | 3.10e-01 | 0.4672865596 |
|  | 19 | 35329860 | 35330243 | PDFSTATSENSEMBLUCSC | 384 | 4 | 21892 | island | 0.162774 | 0.4734748 | Not queried | 2.18 | 1.14e-01 | 0.3123457953 |
|  | 20 | 55904633 | 55904875 | PDFSTATSENSEMBLUCSC | 243 | 7 | 26279 | island | 0.629064 | 0.8873675 | Not queried | 2.18 | 1.65e-01 | 0.3826488233 |
|  | 12 | 106976448 | 106976843 | PDFSTATSENSEMBLUCSC | 396 | 7 | 9970 | tss;island | 0.016580 | 0.0508069 | Not queried | 2.18 | 3.79e-02 | 0.2121545571 |
|  | 1 | 25868969 | 25869196 | PDFSTATSENSEMBLUCSC | 228 | 4 | 1370 | tss;island | 0.076452 | 0.5139389 | Not queried | 2.18 | 2.00e-01 | 0.3826488233 |
|  | 8 | 145133526 | 145133899 | PDFSTATSENSEMBLUCSC | 374 | 4 | 39509 | gene | 0.011102 | 0.0186937 | Not queried | 2.18 | 6.86e-01 | 0.7223604052 |
|  | 6 | 5851644 | 5851994 | PDFSTATSENSEMBLUCSC | 351 | 5 | 35401 | island | 0.087955 | 0.2642338 | Not queried | 2.18 | 2.22e-01 | 0.3935758299 |
|  | 6 | 29601398 | 29601705 | PDFSTATSENSEMBLUCSC | 308 | 6 | 34750 | tss;island | 0.546702 | 0.8102783 | Not queried | 2.18 | 3.10e-01 | 0.4672865596 |
|  | 6 | 24646254 | 24646782 | PDFSTATSENSEMBLUCSC | 529 | 7 | 34670 | tss | 0.018025 | 0.0054877 | Not queried | 2.18 | 3.18e-01 | 0.4672865596 |
|  | 8 | 48100081 | 48100186 | PDFSTATSENSEMBLUCSC | 106 | 4 | 40269 | island | 0.015737 | 0.0141942 | Not queried | 2.18 | 8.86e-01 | 0.8941250130 |
|  | 8 | 22422657 | 22423091 | PDFSTATSENSEMBLUCSC | 435 | 8 | 39234 | gene;tss;island | 0.013601 | 0.0729163 | Not queried | 2.18 | 1.61e-01 | 0.3814043454 |
|  | 1 | 8085604 | 8085795 | PDFSTATSENSEMBLUCSC | 192 | 4 | 192 | gene;island | 0.142693 | 0.0182648 | Not queried | 2.18 | 1.14e-01 | 0.3123457953 |
|  | 6 | 17282284 | 17282859 | PDFSTATSENSEMBLUCSC | 576 | 10 | 33587 | gene;tss;island | 0.107877 | 0.2304046 | Not queried | 2.18 | 5.79e-01 | 0.6514968922 |
|  | 3 | 44626029 | 44626860 | PDFSTATSENSEMBLUCSC | 832 | 11 | 29036 | island | 0.015951 | 0.0419183 | Not queried | 2.18 | 4.73e-02 | 0.2315877671 |
|  | 8 | 25905478 | 25905811 | PDFSTATSENSEMBLUCSC | 334 | 4 | 40171 | island | 0.335054 | 0.6178983 | Not queried | 2.18 | 3.43e-01 | 0.4672865596 |
|  | 6 | 32725075 | 32725438 | PDFSTATSENSEMBLUCSC | 364 | 5 | 34131 | gene | 0.382679 | 0.4704989 | Not queried | 2.18 | 4.21e-01 | 0.5404252807 |
|  | 16 | 11350740 | 11351330 | PDFSTATSENSEMBLUCSC | 591 | 10 | 15092 | tss;island | 0.012176 | 0.0438953 | Not queried | 2.18 | 5.24e-02 | 0.2315877671 |
|  | 14 | 105444724 | 105445253 | PDFSTATSENSEMBLUCSC | 530 | 8 | 12663 | tss | 0.015087 | 0.0803697 | Not queried | 2.18 | 1.30e-01 | 0.3397986247 |
|  | 15 | 69323876 | 69324156 | PDFSTATSENSEMBLUCSC | 281 | 5 | 13397 | gene;island | 0.127457 | 0.0375231 | Not queried | 2.18 | 5.56e-02 | 0.2315877671 |
|  | 17 | 6347867 | 6348200 | PDFSTATSENSEMBLUCSC | 334 | 6 | 16372 | gene | 0.346826 | 0.0201331 | Not queried | 2.18 | 4.85e-01 | 0.5640816327 |
|  | 5 | 87971794 | 87972265 | PDFSTATSENSEMBLUCSC | 472 | 7 | 31679 | gene;island | 0.025241 | 0.0108808 | Not queried | 2.18 | 2.09e-01 | 0.3935758299 |
|  | 7 | 24796981 | 24797884 | PDFSTATSENSEMBLUCSC | 904 | 12 | 38318 | island | 0.131219 | 0.6970204 | Not queried | 2.18 | 2.42e-01 | 0.4149845107 |
|  | 3 | 64430634 | 64431038 | PDFSTATSENSEMBLUCSC | 405 | 5 | 29261 | island | 0.036546 | 0.1710696 | Not queried | 2.18 | 9.52e-02 | 0.3104222821 |
|  | 6 | 105400884 | 105401339 | PDFSTATSENSEMBLUCSC | 456 | 5 | 36373 | island | 0.389981 | 0.0503490 | Not queried | 2.17 | 9.52e-02 | 0.3104222821 |
|  | 10 | 101282572 | 101282883 | PDFSTATSENSEMBLUCSC | 312 | 5 | 6009 | island | 0.616440 | 0.8450342 | Not queried | 2.17 | 1.51e-01 | 0.3618000441 |
|  | 15 | 26108084 | 26108412 | PDFSTATSENSEMBLUCSC | 329 | 7 | 13959 | island | 0.007448 | 0.0354872 | Not queried | 2.17 | 1.75e-02 | 0.1830867741 |
|  | 6 | 99292170 | 99292438 | PDFSTATSENSEMBLUCSC | 269 | 4 | 36347 | island | 0.103296 | 0.0234357 | Not queried | 2.17 | 3.43e-01 | 0.4672865596 |
|  | 16 | 68270252 | 68270387 | PDFSTATSENSEMBLUCSC | 136 | 4 | 15319 | tss | 0.043555 | 0.1525232 | Not queried | 2.17 | 1.14e-01 | 0.3123457953 |
|  | 5 | 140305947 | 140306458 | PDFSTATSENSEMBLUCSC | 512 | 10 | 31804 | gene;island | 0.121438 | 0.0936216 | Not queried | 2.17 | 2.80e-01 | 0.4672865596 |
|  | 10 | 119312691 | 119313075 | PDFSTATSENSEMBLUCSC | 385 | 4 | 6183 | island | 0.264122 | 0.0543216 | Not queried | 2.17 | 3.43e-01 | 0.4672865596 |
|  | 8 | 144387646 | 144387935 | PDFSTATSENSEMBLUCSC | 290 | 4 | 40653 | island | 0.563774 | 0.8541781 | Not queried | 2.17 | 4.86e-01 | 0.5640816327 |
|  | 8 | 1094484 | 1094904 | PDFSTATSENSEMBLUCSC | 421 | 6 | 40012 | island | 0.422182 | 0.0680751 | Not queried | 2.17 | 1.52e-02 | 0.1830867741 |
|  | 17 | 46507549 | 46507895 | PDFSTATSENSEMBLUCSC | 347 | 8 | 18472 | island | 0.093462 | 0.0687748 | Not queried | 2.17 | 5.05e-01 | 0.5852592101 |
|  | 5 | 41870796 | 41871189 | PDFSTATSENSEMBLUCSC | 394 | 9 | 32038 | tss | 0.017950 | 0.2441021 | Not queried | 2.17 | 2.97e-01 | 0.4672865596 |
|  | 1 | 3157287 | 3157510 | PDFSTATSENSEMBLUCSC | 224 | 4 | 111 | gene;island | 0.614771 | 0.8318624 | Not queried | 2.17 | 5.71e-02 | 0.2315877671 |
|  | 2 | 170335723 | 170335894 | PDFSTATSENSEMBLUCSC | 172 | 5 | 23702 | tss;island | 0.055545 | 0.4272432 | Not queried | 2.17 | 1.51e-01 | 0.3618000441 |
|  | 5 | 113697861 | 113697946 | PDFSTATSENSEMBLUCSC | 86 | 4 | 32222 | tss | 0.126186 | 0.0508916 | Not queried | 2.17 | 6.86e-01 | 0.7223604052 |
|  | 5 | 135265753 | 135266135 | PDFSTATSENSEMBLUCSC | 383 | 6 | 33142 | island | 0.038047 | 0.0800113 | Not queried | 2.17 | 3.10e-01 | 0.4672865596 |
|  | 20 | 34359544 | 34359917 | PDFSTATSENSEMBLUCSC | 374 | 9 | 25711 | tss | 0.004329 | 0.0094883 | Not queried | 2.17 | 1.61e-01 | 0.3826488233 |
|  | 2 | 164593221 | 164593498 | PDFSTATSENSEMBLUCSC | 278 | 4 | 23691 | tss;island | 0.032324 | 0.1757761 | Not queried | 2.17 | 1.14e-01 | 0.3123457953 |
|  | 3 | 8811437 | 8811758 | PDFSTATSENSEMBLUCSC | 322 | 4 | 28122 | tss | 0.872177 | 0.7026152 | Not queried | 2.17 | 3.43e-01 | 0.4672865596 |
|  | 3 | 52811297 | 52811637 | PDFSTATSENSEMBLUCSC | 341 | 4 | 28390 | tss | 0.360600 | 0.6940198 | Not queried | 2.17 | 3.43e-01 | 0.4672865596 |
|  | 12 | 133186599 | 133187174 | PDFSTATSENSEMBLUCSC | 576 | 5 | 11066 | island | 0.469969 | 0.1667485 | Not queried | 2.17 | 4.21e-01 | 0.5404252807 |
|  | 5 | 140186484 | 140186983 | PDFSTATSENSEMBLUCSC | 500 | 6 | 31795 | gene;island | 0.201562 | 0.6490287 | Not queried | 2.16 | 3.10e-01 | 0.4672865596 |
|  | 1 | 34632573 | 34632658 | PDFSTATSENSEMBLUCSC | 86 | 4 | 1476 | tss;island | 0.032235 | 0.1315593 | Not queried | 2.16 | 3.43e-01 | 0.4672865596 |
|  | 4 | 57522493 | 57523055 | PDFSTATSENSEMBLUCSC | 563 | 9 | 30010 | gene;island | 0.008356 | 0.0514101 | Not queried | 2.16 | 4.00e-02 | 0.2180389943 |
|  | 4 | 57522689 | 57523055 | PDFSTATSENSEMBLUCSC | 367 | 7 | 30411 | tss | 0.008356 | 0.0514101 | Not queried | 2.16 | 7.28e-02 | 0.2713481487 |
|  | 1 | 9775755 | 9776133 | PDFSTATSENSEMBLUCSC | 379 | 4 | 204 | gene;island | 0.961975 | 0.9002175 | Not queried | 2.16 | 2.00e-01 | 0.3826488233 |
|  | 11 | 67183078 | 67183616 | PDFSTATSENSEMBLUCSC | 539 | 8 | 8545 | island | 0.018898 | 0.2600868 | Not queried | 2.16 | 1.30e-01 | 0.3397986247 |
|  | 1 | 155948550 | 155949012 | PDFSTATSENSEMBLUCSC | 463 | 5 | 2008 | tss;island | 0.014550 | 0.0119415 | Not queried | 2.16 | 4.21e-01 | 0.5404252807 |
|  | 20 | 44993182 | 44993436 | PDFSTATSENSEMBLUCSC | 255 | 7 | 25790 | tss | 0.116040 | 0.0172185 | Not queried | 2.16 | 1.28e-01 | 0.3397986247 |
|  | 7 | 150210742 | 150211152 | PDFSTATSENSEMBLUCSC | 411 | 5 | 37980 | tss | 0.544294 | 0.6900520 | Not queried | 2.16 | 2.22e-01 | 0.3935758299 |
|  | 6 | 41343210 | 41343830 | PDFSTATSENSEMBLUCSC | 621 | 6 | 36120 | island | 0.018414 | 0.0392064 | Not queried | 2.16 | 8.18e-01 | 0.8469867211 |
|  | 11 | 31827593 | 31828040 | PDFSTATSENSEMBLUCSC | 448 | 7 | 6561 | gene;island | 0.183850 | 0.0346008 | Not queried | 2.16 | 1.28e-01 | 0.3397986247 |
|  | 17 | 10741502 | 10741610 | PDFSTATSENSEMBLUCSC | 109 | 4 | 17136 | tss | 0.696469 | 0.9147984 | Not queried | 2.16 | 3.43e-01 | 0.4672865596 |
|  | 10 | 112256517 | 112256825 | PDFSTATSENSEMBLUCSC | 309 | 4 | 5356 | tss;island | 0.472212 | 0.8236765 | Not queried | 2.16 | 2.00e-01 | 0.3826488233 |
|  | 14 | 37666810 | 37666994 | PDFSTATSENSEMBLUCSC | 185 | 5 | 12361 | tss;island | 0.033375 | 0.1746561 | Not queried | 2.16 | 2.22e-01 | 0.3935758299 |
|  | 5 | 137225191 | 137225509 | PDFSTATSENSEMBLUCSC | 319 | 5 | 31763 | gene;island | 0.002248 | 0.0026534 | Not queried | 2.16 | 2.22e-01 | 0.3935758299 |
|  | 15 | 53097649 | 53097840 | PDFSTATSENSEMBLUCSC | 192 | 4 | 14154 | island | 0.020573 | 0.1154236 | Not queried | 2.16 | 1.14e-01 | 0.3123457953 |
|  | 10 | 134838014 | 134838656 | PDFSTATSENSEMBLUCSC | 643 | 7 | 6326 | island | 0.261896 | 0.3737896 | Not queried | 2.16 | 2.59e-01 | 0.4381244265 |
|  | 19 | 52599190 | 52599329 | PDFSTATSENSEMBLUCSC | 140 | 5 | 21006 | tss | 0.037107 | 0.0091362 | Not queried | 2.16 | 4.21e-01 | 0.5404252807 |
|  | 11 | 61335071 | 61335269 | PDFSTATSENSEMBLUCSC | 199 | 4 | 6631 | gene;island | 0.074132 | 0.0231800 | Not queried | 2.15 | 2.00e-01 | 0.3826488233 |
|  | 19 | 57610536 | 57610870 | PDFSTATSENSEMBLUCSC | 335 | 4 | 22456 | island | 0.175656 | 0.5281522 | Not queried | 2.15 | 3.43e-01 | 0.4672865596 |
|  | 3 | 123167507 | 123167770 | PDFSTATSENSEMBLUCSC | 264 | 4 | 28545 | tss;island | 0.220817 | 0.0230532 | Not queried | 2.15 | 4.86e-01 | 0.5640816327 |
|  | 14 | 36983404 | 36983949 | PDFSTATSENSEMBLUCSC | 546 | 7 | 12357 | tss;island | 0.022031 | 0.1993156 | Not queried | 2.15 | 1.65e-01 | 0.3826488233 |
|  | 5 | 135266509 | 135266896 | PDFSTATSENSEMBLUCSC | 388 | 5 | 31760 | gene;island | 0.185549 | 0.1299760 | Not queried | 2.15 | 2.22e-01 | 0.3935758299 |
|  | 4 | 53727714 | 53728038 | PDFSTATSENSEMBLUCSC | 325 | 5 | 30393 | tss;island | 0.905061 | 0.7210437 | Not queried | 2.15 | 5.48e-01 | 0.6179718485 |
|  | 16 | 2907517 | 2907859 | PDFSTATSENSEMBLUCSC | 343 | 4 | 14616 | gene;island | 0.524893 | 0.2270008 | Not queried | 2.15 | 5.71e-02 | 0.2315877671 |
|  | 22 | 29834388 | 29834697 | PDFSTATSENSEMBLUCSC | 310 | 5 | 26832 | gene | 0.131315 | 0.5786169 | Not queried | 2.15 | 2.22e-01 | 0.3935758299 |
|  | 19 | 37959744 | 37959961 | PDFSTATSENSEMBLUCSC | 218 | 7 | 20739 | tss | 0.019369 | 0.0075376 | Not queried | 2.15 | 7.28e-02 | 0.2713481487 |
|  | 2 | 128051810 | 128051925 | PDFSTATSENSEMBLUCSC | 116 | 5 | 23611 | tss | 0.008757 | 0.0062341 | Not queried | 2.15 | 2.22e-01 | 0.3935758299 |
|  | 1 | 55088984 | 55089372 | PDFSTATSENSEMBLUCSC | 389 | 5 | 549 | gene | 0.580170 | 0.6898542 | Not queried | 2.15 | 4.21e-01 | 0.5404252807 |
|  | 16 | 2198395 | 2198504 | PDFSTATSENSEMBLUCSC | 110 | 4 | 15018 | tss;island | 0.008353 | 0.0490011 | Not queried | 2.15 | 2.00e-01 | 0.3826488233 |
|  | 8 | 99961768 | 99962118 | PDFSTATSENSEMBLUCSC | 351 | 4 | 39375 | gene;island | 0.286597 | 0.5322123 | Not queried | 2.15 | 3.43e-01 | 0.4672865596 |
|  | 8 | 41519026 | 41519399 | PDFSTATSENSEMBLUCSC | 374 | 4 | 39290 | gene;tss | 0.900895 | 0.9607751 | Not queried | 2.15 | 1.14e-01 | 0.3123457953 |
|  | 10 | 72163475 | 72163814 | PDFSTATSENSEMBLUCSC | 340 | 5 | 5134 | tss | 0.011915 | 0.0024626 | Not queried | 2.15 | 9.52e-02 | 0.3104222821 |
|  | 10 | 128077307 | 128077376 | PDFSTATSENSEMBLUCSC | 70 | 6 | 5433 | tss;island | 0.402423 | 0.0986186 | Not queried | 2.15 | 4.11e-02 | 0.2180389943 |
|  | 1 | 112531263 | 112531653 | PDFSTATSENSEMBLUCSC | 391 | 4 | 689 | gene | 0.022813 | 0.3138943 | Not queried | 2.15 | 1.14e-01 | 0.3123457953 |
|  | 14 | 101830869 | 101831332 | PDFSTATSENSEMBLUCSC | 464 | 4 | 13155 | island | 0.369489 | 0.7105475 | Not queried | 2.15 | 2.00e-01 | 0.3826488233 |
|  | 11 | 84634409 | 84634735 | PDFSTATSENSEMBLUCSC | 327 | 5 | 6851 | gene | 0.446178 | 0.8272360 | Not queried | 2.15 | 2.22e-01 | 0.3935758299 |
|  | 3 | 50283852 | 50284305 | PDFSTATSENSEMBLUCSC | 454 | 4 | 27797 | gene;tss | 0.145245 | 0.0196805 | Not queried | 2.15 | 4.86e-01 | 0.5640816327 |
|  | 4 | 15376072 | 15376215 | PDFSTATSENSEMBLUCSC | 144 | 8 | 29958 | gene | 0.648992 | 0.9507108 | Not queried | 2.15 | 4.99e-02 | 0.2315877671 |
|  | 4 | 122685352 | 122685675 | PDFSTATSENSEMBLUCSC | 324 | 6 | 30113 | gene;tss;island | 0.224906 | 0.2559040 | Not queried | 2.15 | 5.89e-01 | 0.6570484697 |
|  | 7 | 121955208 | 121955894 | PDFSTATSENSEMBLUCSC | 687 | 6 | 38873 | island | 0.012057 | 0.0554851 | Not queried | 2.15 | 9.31e-02 | 0.3104222821 |
|  | 5 | 72715731 | 72716122 | PDFSTATSENSEMBLUCSC | 392 | 5 | 32872 | island | 0.016098 | 0.0253329 | Not queried | 2.15 | 2.22e-01 | 0.3935758299 |
|  | 2 | 239228826 | 239229395 | PDFSTATSENSEMBLUCSC | 570 | 7 | 25244 | island | 0.004386 | 0.0025213 | Not queried | 2.14 | 9.02e-01 | 0.9091904204 |
|  | 16 | 86600309 | 86600499 | PDFSTATSENSEMBLUCSC | 191 | 4 | 15404 | tss;island | 0.022933 | 0.0776261 | Not queried | 2.14 | 1.14e-01 | 0.3123457953 |
|  | 7 | 14028690 | 14029085 | PDFSTATSENSEMBLUCSC | 396 | 6 | 36898 | gene;island | 0.005513 | 0.0199296 | Not queried | 2.14 | 3.94e-01 | 0.5236526859 |
|  | 4 | 183061830 | 183062004 | PDFSTATSENSEMBLUCSC | 175 | 5 | 30188 | gene;island | 0.087876 | 0.5096772 | Not queried | 2.14 | 5.56e-02 | 0.2315877671 |
|  | 11 | 59578351 | 59578540 | PDFSTATSENSEMBLUCSC | 190 | 5 | 7319 | tss | 0.006566 | 0.0735545 | Not queried | 2.14 | 2.22e-01 | 0.3935758299 |
|  | 11 | 1282842 | 1283165 | PDFSTATSENSEMBLUCSC | 324 | 4 | 6407 | gene | 0.742977 | 0.9184042 | Not queried | 2.14 | 2.86e-02 | 0.1830867741 |
|  | 1 | 165324679 | 165325231 | PDFSTATSENSEMBLUCSC | 553 | 7 | 3927 | island | 0.105957 | 0.0264925 | Not queried | 2.14 | 2.59e-01 | 0.4381244265 |
|  | 4 | 2366485 | 2366831 | PDFSTATSENSEMBLUCSC | 347 | 4 | 29889 | gene;island | 0.487525 | 0.2605101 | Not queried | 2.14 | 2.86e-02 | 0.1830867741 |
|  | 6 | 84562778 | 84563098 | PDFSTATSENSEMBLUCSC | 321 | 9 | 36292 | island | 0.017981 | 0.1777476 | Not queried | 2.14 | 3.15e-02 | 0.1866936846 |
|  | 14 | 91977240 | 91977306 | PDFSTATSENSEMBLUCSC | 67 | 4 | 12561 | tss;island | 0.003445 | 0.0018551 | Not queried | 2.14 | 5.71e-02 | 0.2315877671 |
|  | 10 | 50604330 | 50604569 | PDFSTATSENSEMBLUCSC | 240 | 4 | 5733 | island | 0.012674 | 0.0541104 | Not queried | 2.14 | 1.14e-01 | 0.3123457953 |
|  | 10 | 102987993 | 102988529 | PDFSTATSENSEMBLUCSC | 537 | 5 | 4726 | gene;tss;island | 0.068803 | 0.1683915 | Not queried | 2.14 | 4.21e-01 | 0.5404252807 |
|  | 13 | 36705475 | 36705622 | PDFSTATSENSEMBLUCSC | 148 | 4 | 11391 | tss | 0.022629 | 0.0750983 | Not queried | 2.14 | 5.71e-02 | 0.2315877671 |
|  | 12 | 132862800 | 132863118 | PDFSTATSENSEMBLUCSC | 319 | 5 | 9443 | gene;island | 0.063178 | 0.1351692 | Not queried | 2.13 | 2.22e-01 | 0.3935758299 |
|  | 17 | 40936498 | 40936820 | PDFSTATSENSEMBLUCSC | 323 | 5 | 16588 | gene;island | 0.021050 | 0.1561701 | Not queried | 2.13 | 4.21e-01 | 0.5404252807 |
|  | 1 | 160312929 | 160313057 | PDFSTATSENSEMBLUCSC | 129 | 5 | 2038 | tss | 0.006777 | 0.0017536 | Not queried | 2.13 | 4.21e-01 | 0.5404252807 |
|  | 19 | 12780830 | 12781150 | PDFSTATSENSEMBLUCSC | 321 | 4 | 19861 | gene;tss;island | 0.007535 | 0.0520090 | Not queried | 2.13 | 2.00e-01 | 0.3826488233 |
|  | 5 | 113697330 | 113697649 | PDFSTATSENSEMBLUCSC | 320 | 4 | 32221 | tss;island | 0.015022 | 0.0053236 | Not queried | 2.13 | 2.00e-01 | 0.3826488233 |
|  | 6 | 32171786 | 32172151 | PDFSTATSENSEMBLUCSC | 366 | 8 | 34088 | gene | 0.255991 | 0.4761910 | Not queried | 2.13 | 4.66e-03 | 0.1498346615 |
|  | 18 | 51750950 | 51751391 | PDFSTATSENSEMBLUCSC | 442 | 7 | 19522 | island | 0.005750 | 0.0024286 | Not queried | 2.13 | 7.28e-02 | 0.2713481487 |
|  | 2 | 131512651 | 131513216 | PDFSTATSENSEMBLUCSC | 566 | 12 | 23629 | tss;island | 0.124054 | 0.3834580 | Not queried | 2.13 | 2.42e-01 | 0.4149845107 |
|  | 6 | 10883834 | 10884314 | PDFSTATSENSEMBLUCSC | 481 | 6 | 35441 | island | 0.636838 | 0.4105571 | Not queried | 2.13 | 3.10e-01 | 0.4672865596 |
|  | 2 | 237010048 | 237010430 | PDFSTATSENSEMBLUCSC | 383 | 5 | 23088 | gene;island | 0.468082 | 0.8218771 | Not queried | 2.13 | 5.56e-02 | 0.2315877671 |
|  | 3 | 50242325 | 50242754 | PDFSTATSENSEMBLUCSC | 430 | 5 | 29138 | island | 0.008018 | 0.0428411 | Not queried | 2.13 | 5.48e-01 | 0.6179718485 |
|  | 14 | 102094825 | 102095139 | PDFSTATSENSEMBLUCSC | 315 | 4 | 13161 | island | 0.040883 | 0.0430369 | Not queried | 2.13 | 8.86e-01 | 0.8941250130 |
|  | 17 | 74137399 | 74137522 | PDFSTATSENSEMBLUCSC | 124 | 5 | 17672 | tss | 0.000236 | 0.0029575 | Not queried | 2.13 | 4.21e-01 | 0.5404252807 |
|  | 18 | 32957329 | 32957683 | PDFSTATSENSEMBLUCSC | 355 | 8 | 19237 | tss;island | 0.058621 | 0.4845186 | Not queried | 2.13 | 1.30e-01 | 0.3397986247 |
|  | 1 | 25228582 | 25229192 | PDFSTATSENSEMBLUCSC | 611 | 9 | 329 | gene;island | 0.734494 | 0.9772239 | Not queried | 2.12 | 6.25e-02 | 0.2477578846 |
|  | 7 | 56515510 | 56515846 | PDFSTATSENSEMBLUCSC | 337 | 5 | 37022 | gene;island | 0.521952 | 0.2003555 | Not queried | 2.12 | 3.17e-02 | 0.1866936846 |
|  | 10 | 129535138 | 129535968 | PDFSTATSENSEMBLUCSC | 831 | 11 | 6249 | island | 0.047058 | 0.2171388 | Not queried | 2.12 | 4.01e-01 | 0.5327651490 |
|  | 5 | 39425374 | 39425549 | PDFSTATSENSEMBLUCSC | 176 | 6 | 32033 | tss | 0.126382 | 0.0315732 | Not queried | 2.12 | 3.94e-01 | 0.5236526859 |
|  | 14 | 37641134 | 37641726 | PDFSTATSENSEMBLUCSC | 593 | 8 | 12069 | gene;island | 0.118342 | 0.2721263 | Not queried | 2.12 | 1.05e-01 | 0.3123457953 |
|  | 7 | 130126153 | 130126637 | PDFSTATSENSEMBLUCSC | 485 | 6 | 37223 | gene | 0.026178 | 0.0716954 | Not queried | 2.12 | 1.32e-01 | 0.3397986247 |
|  | 2 | 73429084 | 73429371 | PDFSTATSENSEMBLUCSC | 288 | 7 | 23437 | tss | 0.157638 | 0.0404895 | Not queried | 2.12 | 2.09e-01 | 0.3935758299 |
|  | 6 | 30644681 | 30644832 | PDFSTATSENSEMBLUCSC | 152 | 4 | 33799 | gene;island | 0.841471 | 0.6575656 | Not queried | 2.12 | 4.86e-01 | 0.5640816327 |
|  | 1 | 19639298 | 19639494 | PDFSTATSENSEMBLUCSC | 197 | 4 | 282 | gene;tss;island | 0.032820 | 0.2153276 | Not queried | 2.12 | 6.86e-01 | 0.7223604052 |
|  | 5 | 139040820 | 139041014 | PDFSTATSENSEMBLUCSC | 195 | 4 | 31778 | gene;island | 0.012660 | 0.0129514 | Not queried | 2.12 | 8.86e-01 | 0.8941250130 |
|  | 5 | 140473857 | 140474578 | PDFSTATSENSEMBLUCSC | 722 | 9 | 33209 | island | 0.032266 | 0.4031879 | Not queried | 2.12 | 4.00e-02 | 0.2180389943 |
|  | 15 | 72489416 | 72489828 | PDFSTATSENSEMBLUCSC | 413 | 4 | 13408 | gene;island | 0.140460 | 0.4055419 | Not queried | 2.12 | 6.86e-01 | 0.7223604052 |
|  | 7 | 138552716 | 138552825 | PDFSTATSENSEMBLUCSC | 110 | 4 | 37239 | gene;island | 0.820632 | 0.9727796 | Not queried | 2.12 | 2.00e-01 | 0.3826488233 |
|  | 19 | 15083795 | 15083916 | PDFSTATSENSEMBLUCSC | 122 | 5 | 20551 | tss | 0.373523 | 0.4972341 | Not queried | 2.12 | 4.21e-01 | 0.5404252807 |
|  | 10 | 71336773 | 71337104 | PDFSTATSENSEMBLUCSC | 332 | 4 | 5807 | island | 0.013686 | 0.0167100 | Not queried | 2.12 | 6.86e-01 | 0.7223604052 |
|  | 15 | 73076635 | 73077046 | PDFSTATSENSEMBLUCSC | 412 | 6 | 13780 | tss;island | 0.312048 | 0.0622330 | Not queried | 2.12 | 1.32e-01 | 0.3397986247 |
|  | 2 | 172950067 | 172950626 | PDFSTATSENSEMBLUCSC | 560 | 10 | 24861 | island | 0.016256 | 0.0039084 | Not queried | 2.12 | 2.32e-02 | 0.1830867741 |
|  | 4 | 188916496 | 188916875 | PDFSTATSENSEMBLUCSC | 380 | 10 | 30710 | tss | 0.304918 | 0.0596189 | Not queried | 2.12 | 1.47e-02 | 0.1830867741 |
|  | 8 | 110656982 | 110657197 | PDFSTATSENSEMBLUCSC | 216 | 6 | 39400 | gene;island | 0.002576 | 0.0016554 | Not queried | 2.12 | 4.85e-01 | 0.5640816327 |
|  | 7 | 157467883 | 157468443 | PDFSTATSENSEMBLUCSC | 561 | 7 | 37339 | gene | 0.556969 | 0.5872824 | Not queried | 2.12 | 2.59e-01 | 0.4381244265 |
|  | 11 | 118401536 | 118401801 | PDFSTATSENSEMBLUCSC | 266 | 4 | 7734 | tss | 0.002707 | 0.0037170 | Not queried | 2.12 | 3.43e-01 | 0.4672865596 |
|  | 17 | 46800992 | 46801314 | PDFSTATSENSEMBLUCSC | 323 | 4 | 16663 | gene;tss;island | 0.019784 | 0.1061188 | Not queried | 2.11 | 2.00e-01 | 0.3826488233 |
|  | 10 | 73479514 | 73479848 | PDFSTATSENSEMBLUCSC | 335 | 5 | 4622 | gene | 0.324592 | 0.7857140 | Not queried | 2.11 | 3.17e-02 | 0.1866936846 |
|  | 17 | 43222106 | 43222258 | PDFSTATSENSEMBLUCSC | 153 | 4 | 18426 | island | 0.213571 | 0.5630675 | Not queried | 2.11 | 2.00e-01 | 0.3826488233 |
|  | 8 | 120868172 | 120868512 | PDFSTATSENSEMBLUCSC | 341 | 6 | 39888 | tss;island | 0.002912 | 0.0004384 | Not queried | 2.11 | 2.40e-01 | 0.4133265075 |
|  | 11 | 64703415 | 64703818 | PDFSTATSENSEMBLUCSC | 404 | 5 | 7409 | tss | 0.430469 | 0.6263208 | Not queried | 2.11 | 3.10e-01 | 0.4672865596 |
|  | 19 | 1401576 | 1401781 | PDFSTATSENSEMBLUCSC | 206 | 4 | 20304 | tss | 0.102313 | 0.0227106 | Not queried | 2.11 | 3.43e-01 | 0.4672865596 |
|  | 5 | 134872610 | 134872659 | PDFSTATSENSEMBLUCSC | 50 | 4 | 32306 | tss;island | 0.077866 | 0.4191282 | Not queried | 2.11 | 5.71e-02 | 0.2315877671 |
|  | 18 | 77631625 | 77631756 | PDFSTATSENSEMBLUCSC | 132 | 4 | 19143 | gene;island | 0.538380 | 0.8230429 | Not queried | 2.11 | 5.71e-02 | 0.2315877671 |
|  | 2 | 176943669 | 176943914 | PDFSTATSENSEMBLUCSC | 246 | 4 | 24886 | island | 0.326741 | 0.0608433 | Not queried | 2.11 | 3.43e-01 | 0.4672865596 |
|  | 15 | 65689058 | 65689433 | PDFSTATSENSEMBLUCSC | 376 | 6 | 13380 | gene;island | 0.031253 | 0.0230401 | Not queried | 2.11 | 6.99e-01 | 0.7290267580 |
|  | 2 | 155554688 | 155555053 | PDFSTATSENSEMBLUCSC | 366 | 6 | 23671 | tss | 0.042251 | 0.1259365 | Not queried | 2.11 | 2.60e-02 | 0.1830867741 |
|  | 19 | 34918900 | 34919248 | PDFSTATSENSEMBLUCSC | 349 | 7 | 20692 | tss | 0.003519 | 0.0013785 | Not queried | 2.11 | 8.05e-01 | 0.8347790563 |
|  | 10 | 334430 | 334773 | PDFSTATSENSEMBLUCSC | 344 | 5 | 4432 | gene | 0.423948 | 0.5293270 | Not queried | 2.11 | 1.51e-01 | 0.3618000441 |
|  | 2 | 235859315 | 235859792 | PDFSTATSENSEMBLUCSC | 478 | 4 | 23962 | tss;island | 0.094492 | 0.3375256 | Not queried | 2.11 | 4.86e-01 | 0.5640816327 |
|  | 14 | 101520369 | 101520662 | PDFSTATSENSEMBLUCSC | 294 | 4 | 12628 | tss | 0.480577 | 0.7517739 | Not queried | 2.11 | 2.00e-01 | 0.3826488233 |
|  | 1 | 223316476 | 223316927 | PDFSTATSENSEMBLUCSC | 452 | 6 | 4234 | island | 0.001549 | 0.0034145 | Not queried | 2.11 | 1.80e-01 | 0.3826488233 |
|  | 1 | 209848306 | 209848577 | PDFSTATSENSEMBLUCSC | 272 | 5 | 2231 | tss;island | 0.038318 | 0.2054520 | Not queried | 2.11 | 1.51e-01 | 0.3618000441 |
|  | 19 | 58695006 | 58695164 | PDFSTATSENSEMBLUCSC | 159 | 4 | 20258 | gene;island | 0.016512 | 0.0065461 | Not queried | 2.11 | 3.43e-01 | 0.4672865596 |
|  | 8 | 11560299 | 11560851 | PDFSTATSENSEMBLUCSC | 553 | 12 | 39552 | tss;island | 0.047480 | 0.2135525 | Not queried | 2.11 | 2.42e-02 | 0.1830867741 |
|  | 19 | 49198900 | 49199217 | PDFSTATSENSEMBLUCSC | 318 | 4 | 20923 | tss | 0.334459 | 0.5102684 | Not queried | 2.10 | 3.43e-01 | 0.4672865596 |
|  | 17 | 78157897 | 78158248 | PDFSTATSENSEMBLUCSC | 352 | 4 | 16858 | gene;island | 0.212289 | 0.5356309 | Not queried | 2.10 | 2.00e-01 | 0.3826488233 |
|  | 6 | 25930894 | 25931205 | PDFSTATSENSEMBLUCSC | 312 | 5 | 34676 | tss | 0.591929 | 0.9180470 | Not queried | 2.10 | 3.10e-01 | 0.4672865596 |
|  | 5 | 54518469 | 54519307 | PDFSTATSENSEMBLUCSC | 839 | 7 | 32800 | island | 0.047573 | 0.0241057 | Not queried | 2.10 | 4.56e-01 | 0.5640816327 |
|  | 2 | 54342584 | 54342961 | PDFSTATSENSEMBLUCSC | 378 | 4 | 22682 | gene;island | 0.001841 | 0.0029276 | Not queried | 2.10 | 4.86e-01 | 0.5640816327 |
|  | 10 | 129533998 | 129534411 | PDFSTATSENSEMBLUCSC | 414 | 5 | 6248 | island | 0.298415 | 0.0194293 | Not queried | 2.10 | 6.90e-01 | 0.7223604052 |
|  | 14 | 102504851 | 102505062 | PDFSTATSENSEMBLUCSC | 212 | 4 | 12222 | gene | 0.795198 | 0.9821636 | Not queried | 2.10 | 3.43e-01 | 0.4672865596 |
|  | 12 | 131443231 | 131443486 | PDFSTATSENSEMBLUCSC | 256 | 6 | 9421 | gene | 0.826439 | 0.9643836 | Not queried | 2.10 | 9.31e-02 | 0.3104222821 |
|  | 3 | 69915189 | 69915449 | PDFSTATSENSEMBLUCSC | 261 | 4 | 27856 | gene | 0.086543 | 0.2457305 | Not queried | 2.10 | 4.86e-01 | 0.5640816327 |
|  | 12 | 52845930 | 52846088 | PDFSTATSENSEMBLUCSC | 159 | 4 | 9728 | tss | 0.096368 | 0.3825011 | Not queried | 2.10 | 2.00e-01 | 0.3826488233 |
|  | 12 | 132865182 | 132865474 | PDFSTATSENSEMBLUCSC | 293 | 4 | 9445 | gene;island | 0.471402 | 0.7936891 | Not queried | 2.10 | 1.14e-01 | 0.3123457953 |
|  | 4 | 94749865 | 94750061 | PDFSTATSENSEMBLUCSC | 197 | 6 | 30501 | tss | 0.062675 | 0.0182313 | Not queried | 2.10 | 4.11e-02 | 0.2180389943 |
|  | 1 | 24307153 | 24307535 | PDFSTATSENSEMBLUCSC | 383 | 5 | 1354 | tss;island | 0.005897 | 0.0249386 | Not queried | 2.10 | 6.90e-01 | 0.7223604052 |
|  | 5 | 42951935 | 42952369 | PDFSTATSENSEMBLUCSC | 435 | 4 | 32762 | island | 0.022741 | 0.0562757 | Not queried | 2.10 | 2.86e-02 | 0.1830867741 |
|  | 3 | 157821919 | 157822072 | PDFSTATSENSEMBLUCSC | 154 | 4 | 28011 | gene;island | 0.036300 | 0.2387128 | Not queried | 2.10 | 6.86e-01 | 0.7223604052 |
|  | 5 | 34838922 | 34839241 | PDFSTATSENSEMBLUCSC | 320 | 8 | 32013 | tss | 0.020926 | 0.0091559 | Not queried | 2.10 | 1.95e-01 | 0.3826488233 |
|  | 22 | 19949873 | 19950040 | PDFSTATSENSEMBLUCSC | 168 | 6 | 26970 | tss | 0.296361 | 0.6026653 | Not queried | 2.10 | 8.18e-01 | 0.8469867211 |
|  | 6 | 74103959 | 74104137 | PDFSTATSENSEMBLUCSC | 179 | 4 | 35069 | tss;island | 0.607796 | 0.9115497 | Not queried | 2.10 | 3.43e-01 | 0.4672865596 |
|  | 1 | 220921216 | 220921670 | PDFSTATSENSEMBLUCSC | 455 | 8 | 2262 | tss | 0.051032 | 0.2709414 | Not queried | 2.10 | 6.50e-02 | 0.2477578846 |
|  | 3 | 42846907 | 42847116 | PDFSTATSENSEMBLUCSC | 210 | 4 | 28249 | tss;island | 0.789597 | 0.9417809 | Not queried | 2.09 | 6.86e-01 | 0.7223604052 |
|  | 3 | 172167445 | 172167810 | PDFSTATSENSEMBLUCSC | 366 | 5 | 29667 | island | 0.234779 | 0.2208060 | Not queried | 2.09 | 6.90e-01 | 0.7223604052 |
|  | 2 | 131673771 | 131674221 | PDFSTATSENSEMBLUCSC | 451 | 7 | 23631 | tss | 0.175992 | 0.2857955 | Not queried | 2.09 | 3.79e-02 | 0.2121545571 |
|  | 19 | 55314879 | 55315034 | PDFSTATSENSEMBLUCSC | 156 | 4 | 21065 | tss | 0.298899 | 0.4717482 | Not queried | 2.09 | 8.86e-01 | 0.8941250130 |
|  | 6 | 32062160 | 32062554 | PDFSTATSENSEMBLUCSC | 395 | 5 | 34045 | gene;island | 0.054270 | 0.2293675 | Not queried | 2.09 | 5.56e-02 | 0.2315877671 |
|  | 1 | 2415199 | 2415453 | PDFSTATSENSEMBLUCSC | 255 | 4 | 86 | gene;island | 0.227781 | 0.5409351 | Not queried | 2.09 | 2.00e-01 | 0.3826488233 |
|  | 4 | 74864596 | 74864725 | PDFSTATSENSEMBLUCSC | 130 | 4 | 30432 | tss;island | 0.091118 | 0.3762809 | Not queried | 2.09 | 4.86e-01 | 0.5640816327 |
|  | 6 | 28497278 | 28497370 | PDFSTATSENSEMBLUCSC | 93 | 4 | 33635 | gene | 0.385030 | 0.7025517 | Not queried | 2.09 | 2.00e-01 | 0.3826488233 |
|  | 14 | 95826570 | 95826997 | PDFSTATSENSEMBLUCSC | 428 | 5 | 13110 | island | 0.608986 | 0.4342987 | Not queried | 2.09 | 3.10e-01 | 0.4672865596 |
|  | 11 | 1774722 | 1775039 | PDFSTATSENSEMBLUCSC | 318 | 4 | 6431 | gene;island | 0.676555 | 0.9147124 | Not queried | 2.09 | 1.14e-01 | 0.3123457953 |
|  | 1 | 51433840 | 51434014 | PDFSTATSENSEMBLUCSC | 175 | 4 | 1618 | tss;island | 0.005299 | 0.0027346 | Not queried | 2.09 | 8.86e-01 | 0.8941250130 |
|  | 1 | 154377364 | 154378025 | PDFSTATSENSEMBLUCSC | 662 | 12 | 3789 | island | 0.005943 | 0.0192423 | Not queried | 2.09 | 1.73e-02 | 0.1830867741 |
|  | 19 | 19322527 | 19322902 | PDFSTATSENSEMBLUCSC | 376 | 8 | 21808 | island | 0.008900 | 0.1008369 | Not queried | 2.09 | 1.05e-01 | 0.3123457953 |
|  | 5 | 83678796 | 83679364 | PDFSTATSENSEMBLUCSC | 569 | 6 | 31671 | gene;island | 0.005226 | 0.0994709 | Not queried | 2.09 | 4.85e-01 | 0.5640816327 |
|  | 18 | 44099053 | 44099340 | PDFSTATSENSEMBLUCSC | 288 | 4 | 19257 | tss | 0.016006 | 0.0704488 | Not queried | 2.09 | 6.86e-01 | 0.7223604052 |
|  | 17 | 46507690 | 46507895 | PDFSTATSENSEMBLUCSC | 206 | 7 | 17455 | tss | 0.122736 | 0.0931558 | Not queried | 2.09 | 5.35e-01 | 0.6176455124 |
|  | 19 | 49926748 | 49927309 | PDFSTATSENSEMBLUCSC | 562 | 6 | 20944 | tss | 0.046810 | 0.1458620 | Not queried | 2.09 | 1.32e-01 | 0.3397986247 |
|  | 20 | 34359544 | 34359976 | PDFSTATSENSEMBLUCSC | 433 | 10 | 26131 | island | 0.003258 | 0.0092285 | Not queried | 2.09 | 1.23e-01 | 0.3348527312 |
|  | 2 | 182521874 | 182521926 | PDFSTATSENSEMBLUCSC | 53 | 6 | 23762 | tss;island | 0.009361 | 0.0030597 | Not queried | 2.09 | 2.60e-02 | 0.1830867741 |
|  | 6 | 30295772 | 30296049 | PDFSTATSENSEMBLUCSC | 278 | 4 | 33760 | gene;tss;island | 0.851052 | 0.9796837 | Not queried | 2.09 | 4.86e-01 | 0.5640816327 |
|  | 1 | 18691778 | 18692019 | PDFSTATSENSEMBLUCSC | 242 | 5 | 271 | gene;island | 0.176437 | 0.4759527 | Not queried | 2.09 | 3.10e-01 | 0.4672865596 |
|  | 15 | 76016056 | 76016306 | PDFSTATSENSEMBLUCSC | 251 | 4 | 13818 | tss | 0.111841 | 0.3393808 | Not queried | 2.08 | 6.86e-01 | 0.7223604052 |
|  | 10 | 131767960 | 131768149 | PDFSTATSENSEMBLUCSC | 190 | 4 | 6268 | island | 0.273958 | 0.0385239 | Not queried | 2.08 | 4.86e-01 | 0.5640816327 |
|  | 19 | 49340489 | 49340765 | PDFSTATSENSEMBLUCSC | 277 | 4 | 20138 | gene;tss;island | 0.052480 | 0.0293126 | Not queried | 2.08 | 4.86e-01 | 0.5640816327 |
|  | 1 | 41827960 | 41828276 | PDFSTATSENSEMBLUCSC | 317 | 4 | 3137 | island | 0.011489 | 0.0509377 | Not queried | 2.08 | 6.86e-01 | 0.7223604052 |
|  | 7 | 138483046 | 138483314 | PDFSTATSENSEMBLUCSC | 269 | 4 | 37238 | gene;tss | 0.318246 | 0.7162874 | Not queried | 2.08 | 2.00e-01 | 0.3826488233 |
|  | 1 | 111682944 | 111683426 | PDFSTATSENSEMBLUCSC | 483 | 6 | 681 | gene;tss;island | 0.010113 | 0.0026909 | Not queried | 2.08 | 2.40e-01 | 0.4133265075 |
|  | 19 | 47742558 | 47742961 | PDFSTATSENSEMBLUCSC | 404 | 4 | 22189 | island | 0.038870 | 0.1506262 | Not queried | 2.08 | 5.71e-02 | 0.2315877671 |
|  | 6 | 152127812 | 152128024 | PDFSTATSENSEMBLUCSC | 213 | 4 | 34513 | gene;tss;island | 0.473621 | 0.6669812 | Not queried | 2.08 | 2.86e-02 | 0.1830867741 |
|  | 14 | 65016517 | 65016602 | PDFSTATSENSEMBLUCSC | 86 | 5 | 12460 | tss;island | 0.031029 | 0.0530443 | Not queried | 2.08 | 9.52e-02 | 0.3104222821 |
|  | 10 | 99338056 | 99338240 | PDFSTATSENSEMBLUCSC | 185 | 4 | 4698 | gene;island | 0.870931 | 0.9648793 | Not queried | 2.08 | 3.43e-01 | 0.4672865596 |
|  | 11 | 67442067 | 67442408 | PDFSTATSENSEMBLUCSC | 342 | 6 | 6764 | gene | 0.288648 | 0.6266378 | Not queried | 2.08 | 9.31e-02 | 0.3104222821 |
|  | 3 | 23987430 | 23987467 | PDFSTATSENSEMBLUCSC | 38 | 4 | 27703 | gene;tss;island | 0.002651 | 0.0093602 | Not queried | 2.08 | 3.43e-01 | 0.4672865596 |
|  | 4 | 118006747 | 118006832 | PDFSTATSENSEMBLUCSC | 86 | 6 | 30559 | tss | 0.155771 | 0.0382333 | Not queried | 2.08 | 2.40e-01 | 0.4133265075 |
|  | 7 | 55086091 | 55086288 | PDFSTATSENSEMBLUCSC | 198 | 4 | 37603 | tss;island | 0.355390 | 0.1283766 | Not queried | 2.07 | 5.71e-02 | 0.2315877671 |
|  | 3 | 4508494 | 4508875 | PDFSTATSENSEMBLUCSC | 382 | 4 | 27642 | gene | 0.000764 | 0.0011242 | Not queried | 2.07 | 6.86e-01 | 0.7223604052 |
|  | 14 | 104195181 | 104195666 | PDFSTATSENSEMBLUCSC | 486 | 5 | 12243 | gene;island | 0.918334 | 0.9612446 | Not queried | 2.07 | 5.48e-01 | 0.6179718485 |
|  | 19 | 44220122 | 44220337 | PDFSTATSENSEMBLUCSC | 216 | 4 | 22093 | island | 0.743933 | 0.5106224 | Not queried | 2.07 | 3.43e-01 | 0.4672865596 |
|  | 19 | 9609335 | 9609704 | PDFSTATSENSEMBLUCSC | 370 | 8 | 20425 | tss | 0.084098 | 0.3939870 | Not queried | 2.07 | 1.95e-01 | 0.3826488233 |
|  | 14 | 102026086 | 102026555 | PDFSTATSENSEMBLUCSC | 470 | 11 | 13158 | island | 0.038088 | 0.1289377 | Not queried | 2.07 | 4.10e-03 | 0.1459191215 |
|  | 17 | 747015 | 747496 | PDFSTATSENSEMBLUCSC | 482 | 6 | 16298 | gene;island | 0.334713 | 0.6323557 | Not queried | 2.07 | 4.11e-02 | 0.2180389943 |
|  | 19 | 58728390 | 58728865 | PDFSTATSENSEMBLUCSC | 476 | 5 | 22498 | island | 0.777132 | 0.4538132 | Not queried | 2.07 | 2.22e-01 | 0.3935758299 |
|  | 1 | 201368613 | 201369031 | PDFSTATSENSEMBLUCSC | 419 | 9 | 4075 | island | 0.142288 | 0.0323819 | Not queried | 2.07 | 7.77e-03 | 0.1589602024 |
|  | 7 | 1902804 | 1903159 | PDFSTATSENSEMBLUCSC | 356 | 4 | 36770 | gene;island | 0.726993 | 0.9525028 | Not queried | 2.07 | 8.86e-01 | 0.8941250130 |
|  | 19 | 56988702 | 56989102 | PDFSTATSENSEMBLUCSC | 401 | 7 | 22446 | island | 0.077458 | 0.2055821 | Not queried | 2.07 | 3.79e-02 | 0.2121545571 |
|  | 19 | 42509801 | 42510093 | PDFSTATSENSEMBLUCSC | 293 | 4 | 20063 | gene;island | 0.184139 | 0.4754262 | Not queried | 2.07 | 3.43e-01 | 0.4672865596 |
|  | 7 | 75188876 | 75189413 | PDFSTATSENSEMBLUCSC | 538 | 7 | 37047 | gene;island | 0.794281 | 0.9381548 | Not queried | 2.07 | 5.30e-02 | 0.2315877671 |
|  | 15 | 51973460 | 51973920 | PDFSTATSENSEMBLUCSC | 461 | 6 | 14140 | island | 0.409269 | 0.0959105 | Not queried | 2.07 | 5.89e-01 | 0.6570484697 |
|  | 11 | 118401536 | 118401878 | PDFSTATSENSEMBLUCSC | 343 | 5 | 8858 | island | 0.002953 | 0.0039406 | Not queried | 2.07 | 4.21e-01 | 0.5404252807 |
|  | 12 | 82152229 | 82152625 | PDFSTATSENSEMBLUCSC | 397 | 5 | 9229 | gene;island | 0.020127 | 0.1590574 | Not queried | 2.07 | 9.52e-02 | 0.3104222821 |
|  | 2 | 63281069 | 63281383 | PDFSTATSENSEMBLUCSC | 315 | 4 | 22704 | gene;island | 0.037587 | 0.1677395 | Not queried | 2.06 | 2.00e-01 | 0.3826488233 |
|  | 1 | 60280322 | 60280503 | PDFSTATSENSEMBLUCSC | 182 | 4 | 1659 | tss;island | 0.007889 | 0.0337162 | Not queried | 2.06 | 2.86e-02 | 0.1830867741 |
|  | 16 | 725416 | 725581 | PDFSTATSENSEMBLUCSC | 166 | 4 | 14975 | tss | 0.010630 | 0.0335754 | Not queried | 2.06 | 3.43e-01 | 0.4672865596 |
|  | 4 | 186435443 | 186435667 | PDFSTATSENSEMBLUCSC | 225 | 6 | 30210 | gene | 0.628932 | 0.8811745 | Not queried | 2.06 | 9.31e-02 | 0.3104222821 |
|  | 1 | 2100169 | 2100435 | PDFSTATSENSEMBLUCSC | 267 | 4 | 66 | gene | 0.517585 | 0.7332220 | Not queried | 2.06 | 3.43e-01 | 0.4672865596 |
|  | 7 | 157669880 | 157670171 | PDFSTATSENSEMBLUCSC | 292 | 4 | 37349 | gene;island | 0.039631 | 0.2512375 | Not queried | 2.06 | 2.00e-01 | 0.3826488233 |
|  | 8 | 102236522 | 102236831 | PDFSTATSENSEMBLUCSC | 310 | 4 | 40505 | island | 0.522079 | 0.7943313 | Not queried | 2.06 | 3.43e-01 | 0.4672865596 |
|  | 1 | 162792092 | 162792613 | PDFSTATSENSEMBLUCSC | 522 | 6 | 3921 | island | 0.275288 | 0.0272740 | Not queried | 2.06 | 4.85e-01 | 0.5640816327 |
|  | 1 | 78511600 | 78512129 | PDFSTATSENSEMBLUCSC | 530 | 5 | 595 | gene | 0.019036 | 0.0589176 | Not queried | 2.06 | 9.52e-02 | 0.3104222821 |
|  | 18 | 13611370 | 13611489 | PDFSTATSENSEMBLUCSC | 120 | 5 | 19062 | gene;tss | 0.004980 | 0.0571277 | Not queried | 2.06 | 2.22e-01 | 0.3935758299 |
|  | 17 | 61514690 | 61515010 | PDFSTATSENSEMBLUCSC | 321 | 4 | 16725 | gene;island | 0.761078 | 0.9509752 | Not queried | 2.06 | 6.86e-01 | 0.7223604052 |
|  | 8 | 41583018 | 41583523 | PDFSTATSENSEMBLUCSC | 506 | 5 | 39292 | gene;island | 0.462623 | 0.7435698 | Not queried | 2.06 | 3.17e-02 | 0.1866936846 |
|  | 1 | 24645802 | 24646392 | PDFSTATSENSEMBLUCSC | 591 | 13 | 2858 | island | 0.013740 | 0.0648931 | Not queried | 2.06 | 1.20e-02 | 0.1830867741 |
|  | 1 | 43250691 | 43251253 | PDFSTATSENSEMBLUCSC | 563 | 6 | 3151 | island | 0.450425 | 0.0769018 | Not queried | 2.06 | 4.85e-01 | 0.5640816327 |
|  | 17 | 46669455 | 46669674 | PDFSTATSENSEMBLUCSC | 220 | 7 | 17463 | tss | 0.169650 | 0.6050237 | Not queried | 2.06 | 2.09e-01 | 0.3935758299 |
|  | 12 | 91347805 | 91348242 | PDFSTATSENSEMBLUCSC | 438 | 4 | 9240 | gene;island | 0.535316 | 0.7498978 | Not queried | 2.06 | 3.43e-01 | 0.4672865596 |
|  | 7 | 18126902 | 18127112 | PDFSTATSENSEMBLUCSC | 211 | 4 | 38280 | island | 0.345258 | 0.7608913 | Not queried | 2.06 | 4.86e-01 | 0.5640816327 |
|  | 10 | 102881114 | 102881545 | PDFSTATSENSEMBLUCSC | 432 | 6 | 4723 | gene;island | 0.021384 | 0.2057506 | Not queried | 2.06 | 6.49e-02 | 0.2477578846 |
|  | 14 | 101908561 | 101909021 | PDFSTATSENSEMBLUCSC | 461 | 6 | 13156 | island | 0.238603 | 0.5907211 | Not queried | 2.06 | 3.10e-01 | 0.4672865596 |
|  | 6 | 33135753 | 33136061 | PDFSTATSENSEMBLUCSC | 309 | 7 | 34198 | gene | 0.596140 | 0.8168719 | Not queried | 2.06 | 1.28e-01 | 0.3397986247 |
|  | 14 | 103674159 | 103674342 | PDFSTATSENSEMBLUCSC | 184 | 4 | 13184 | island | 0.474697 | 0.1587206 | Not queried | 2.06 | 2.00e-01 | 0.3826488233 |
|  | 5 | 134871645 | 134872315 | PDFSTATSENSEMBLUCSC | 671 | 18 | 32305 | tss | 0.010954 | 0.0427345 | Not queried | 2.06 | 5.76e-03 | 0.1589602024 |
|  | 10 | 729479 | 729956 | PDFSTATSENSEMBLUCSC | 478 | 6 | 4451 | gene | 0.444563 | 0.7609090 | Not queried | 2.06 | 6.49e-02 | 0.2477578846 |
|  | 5 | 140514811 | 140515207 | PDFSTATSENSEMBLUCSC | 397 | 5 | 31808 | gene | 0.056817 | 0.2486675 | Not queried | 2.06 | 5.56e-02 | 0.2315877671 |
|  | 8 | 22960461 | 22960619 | PDFSTATSENSEMBLUCSC | 159 | 4 | 39239 | gene | 0.042317 | 0.2128241 | Not queried | 2.06 | 3.43e-01 | 0.4672865596 |
|  | 6 | 33180841 | 33181565 | PDFSTATSENSEMBLUCSC | 725 | 14 | 35985 | island | 0.030135 | 0.0746058 | Not queried | 2.06 | 2.74e-02 | 0.1830867741 |
|  | 4 | 619162 | 619314 | PDFSTATSENSEMBLUCSC | 153 | 4 | 30226 | tss | 0.312025 | 0.4527222 | Not queried | 2.06 | 6.86e-01 | 0.7223604052 |
|  | 10 | 134735893 | 134736248 | PDFSTATSENSEMBLUCSC | 356 | 5 | 6318 | island | 0.841673 | 0.9070316 | Not queried | 2.06 | 3.10e-01 | 0.4672865596 |
|  | 6 | 117197952 | 117198281 | PDFSTATSENSEMBLUCSC | 330 | 8 | 35194 | tss | 0.075964 | 0.0177783 | Not queried | 2.06 | 1.61e-01 | 0.3814043454 |
|  | 10 | 132909113 | 132909470 | PDFSTATSENSEMBLUCSC | 358 | 5 | 4840 | gene | 0.264510 | 0.5990727 | Not queried | 2.06 | 2.22e-01 | 0.3935758299 |
|  | 15 | 53086630 | 53087599 | PDFSTATSENSEMBLUCSC | 970 | 9 | 14153 | island | 0.410182 | 0.1773317 | Not queried | 2.06 | 3.87e-01 | 0.5209559659 |
|  | 4 | 8307757 | 8308063 | PDFSTATSENSEMBLUCSC | 307 | 4 | 29950 | gene;island | 0.784095 | 0.9373300 | Not queried | 2.06 | 2.00e-01 | 0.3826488233 |
|  | 1 | 21996199 | 21996376 | PDFSTATSENSEMBLUCSC | 178 | 4 | 1326 | tss;island | 0.016573 | 0.0893301 | Not queried | 2.06 | 3.43e-01 | 0.4672865596 |
|  | 2 | 45161107 | 45161510 | PDFSTATSENSEMBLUCSC | 404 | 4 | 24261 | island | 0.032286 | 0.0802245 | Not queried | 2.06 | 6.86e-01 | 0.7223604052 |
|  | 16 | 28075183 | 28075295 | PDFSTATSENSEMBLUCSC | 113 | 4 | 15153 | tss;island | 0.031364 | 0.1247061 | Not queried | 2.06 | 2.00e-01 | 0.3826488233 |
|  | 17 | 79077169 | 79077463 | PDFSTATSENSEMBLUCSC | 295 | 4 | 16893 | gene;island | 0.540369 | 0.8194218 | Not queried | 2.06 | 2.00e-01 | 0.3826488233 |
|  | 4 | 113970617 | 113970935 | PDFSTATSENSEMBLUCSC | 319 | 6 | 30103 | gene | 0.657963 | 0.1285238 | Not queried | 2.06 | 3.10e-01 | 0.4672865596 |
|  | 13 | 102068234 | 102068650 | PDFSTATSENSEMBLUCSC | 417 | 5 | 11212 | gene;island | 0.038962 | 0.2876562 | Not queried | 2.06 | 1.51e-01 | 0.3618000441 |
|  | 16 | 80966033 | 80966491 | PDFSTATSENSEMBLUCSC | 459 | 6 | 16118 | island | 0.071428 | 0.0118588 | Not queried | 2.05 | 9.31e-02 | 0.3104222821 |
|  | 22 | 45125218 | 45125666 | PDFSTATSENSEMBLUCSC | 449 | 4 | 26895 | gene;island | 0.478851 | 0.1826172 | Not queried | 2.05 | 2.00e-01 | 0.3826488233 |
|  | 15 | 90294471 | 90294776 | PDFSTATSENSEMBLUCSC | 306 | 8 | 14441 | island | 0.024987 | 0.0524074 | Not queried | 2.05 | 5.74e-01 | 0.6460388787 |
|  | 15 | 90294568 | 90294776 | PDFSTATSENSEMBLUCSC | 209 | 6 | 13891 | tss | 0.035780 | 0.1210127 | Not queried | 2.05 | 5.89e-01 | 0.6570484697 |
|  | 3 | 39323202 | 39323539 | PDFSTATSENSEMBLUCSC | 338 | 5 | 28233 | tss | 0.108439 | 0.3780588 | Not queried | 2.05 | 2.22e-01 | 0.3935758299 |
|  | 11 | 128761146 | 128761211 | PDFSTATSENSEMBLUCSC | 66 | 6 | 7807 | tss;island | 0.010309 | 0.0048350 | Not queried | 2.05 | 1.80e-01 | 0.3826488233 |
|  | 10 | 104211296 | 104211488 | PDFSTATSENSEMBLUCSC | 193 | 6 | 6089 | island | 0.362178 | 0.4686381 | Not queried | 2.05 | 6.99e-01 | 0.7290267580 |
|  | 8 | 144824625 | 144824658 | PDFSTATSENSEMBLUCSC | 34 | 4 | 40686 | island | 0.524899 | 0.8216730 | Not queried | 2.05 | 2.00e-01 | 0.3826488233 |
|  | 7 | 4869981 | 4870162 | PDFSTATSENSEMBLUCSC | 182 | 4 | 36859 | gene | 0.425787 | 0.1323347 | Not queried | 2.05 | 3.43e-01 | 0.4672865596 |
|  | 19 | 863054 | 863244 | PDFSTATSENSEMBLUCSC | 191 | 4 | 19635 | gene;island | 0.276542 | 0.0273721 | Not queried | 2.05 | 3.43e-01 | 0.4672865596 |
|  | 22 | 32600722 | 32601274 | PDFSTATSENSEMBLUCSC | 553 | 4 | 27089 | tss | 0.409130 | 0.6777338 | Not queried | 2.05 | 4.86e-01 | 0.5640816327 |
|  | 12 | 115104274 | 115104983 | PDFSTATSENSEMBLUCSC | 710 | 7 | 10844 | island | 0.028962 | 0.4652791 | Not queried | 2.05 | 2.09e-01 | 0.3935758299 |
|  | 5 | 38445522 | 38445614 | PDFSTATSENSEMBLUCSC | 93 | 4 | 32029 | tss | 0.684528 | 0.9023920 | Not queried | 2.05 | 2.00e-01 | 0.3826488233 |
|  | 19 | 54106789 | 54106910 | PDFSTATSENSEMBLUCSC | 122 | 4 | 21032 | tss | 0.179169 | 0.7259045 | Not queried | 2.05 | 6.86e-01 | 0.7223604052 |
|  | 1 | 239550177 | 239550578 | PDFSTATSENSEMBLUCSC | 402 | 6 | 4371 | island | 0.016908 | 0.0594496 | Not queried | 2.05 | 1.80e-01 | 0.3826488233 |
|  | 5 | 140562562 | 140563400 | PDFSTATSENSEMBLUCSC | 839 | 8 | 31813 | gene;island | 0.226750 | 0.2349964 | Not queried | 2.05 | 7.98e-01 | 0.8290400976 |
|  | 17 | 34838488 | 34838831 | PDFSTATSENSEMBLUCSC | 344 | 4 | 18226 | island | 0.372556 | 0.1393394 | Not queried | 2.05 | 4.86e-01 | 0.5640816327 |
|  | 2 | 182545485 | 182545934 | PDFSTATSENSEMBLUCSC | 450 | 9 | 23763 | tss;island | 0.126450 | 0.0137518 | Not queried | 2.05 | 9.39e-02 | 0.3104222821 |
|  | 22 | 47081634 | 47082044 | PDFSTATSENSEMBLUCSC | 411 | 4 | 26915 | gene;island | 0.811746 | 0.9707444 | Not queried | 2.05 | 4.86e-01 | 0.5640816327 |
|  | 12 | 85673200 | 85673347 | PDFSTATSENSEMBLUCSC | 148 | 4 | 9900 | tss;island | 0.046019 | 0.0974175 | Not queried | 2.05 | 3.43e-01 | 0.4672865596 |
|  | 11 | 130029478 | 130029615 | PDFSTATSENSEMBLUCSC | 138 | 4 | 7817 | tss | 0.231744 | 0.1369278 | Not queried | 2.05 | 4.86e-01 | 0.5640816327 |
|  | 19 | 57049530 | 57049857 | PDFSTATSENSEMBLUCSC | 328 | 4 | 21100 | tss;island | 0.009678 | 0.0335468 | Not queried | 2.05 | 2.00e-01 | 0.3826488233 |
|  | 7 | 4921827 | 4922196 | PDFSTATSENSEMBLUCSC | 370 | 4 | 36864 | gene;island | 0.139399 | 0.2545897 | Not queried | 2.05 | 2.00e-01 | 0.3826488233 |
|  | 19 | 45975694 | 45976195 | PDFSTATSENSEMBLUCSC | 502 | 5 | 20098 | gene;island | 0.039785 | 0.2341775 | Not queried | 2.05 | 1.51e-01 | 0.3618000441 |
|  | 14 | 68067023 | 68067294 | PDFSTATSENSEMBLUCSC | 272 | 4 | 12473 | tss | 0.024361 | 0.0270307 | Not queried | 2.04 | 8.86e-01 | 0.8941250130 |
|  | 15 | 45406534 | 45406939 | PDFSTATSENSEMBLUCSC | 406 | 6 | 13326 | gene | 0.024136 | 0.0708683 | Not queried | 2.04 | 1.80e-01 | 0.3826488233 |
|  | 6 | 168533375 | 168533689 | PDFSTATSENSEMBLUCSC | 315 | 4 | 36659 | island | 0.380227 | 0.0730777 | Not queried | 2.04 | 3.43e-01 | 0.4672865596 |
|  | 2 | 242754333 | 242754756 | PDFSTATSENSEMBLUCSC | 424 | 5 | 23147 | gene;island | 0.669520 | 0.9485087 | Not queried | 2.04 | 1.51e-01 | 0.3618000441 |
|  | 14 | 93170534 | 93170970 | PDFSTATSENSEMBLUCSC | 437 | 4 | 12183 | gene | 0.008838 | 0.0040834 | Not queried | 2.04 | 3.43e-01 | 0.4672865596 |
|  | 3 | 187870621 | 187871034 | PDFSTATSENSEMBLUCSC | 414 | 7 | 28780 | tss;island | 0.400495 | 0.6164564 | Not queried | 2.04 | 2.59e-01 | 0.4381244265 |
|  | 22 | 46731652 | 46731750 | PDFSTATSENSEMBLUCSC | 99 | 4 | 26912 | gene;island | 0.003472 | 0.0010393 | Not queried | 2.04 | 3.43e-01 | 0.4672865596 |
|  | 22 | 21386885 | 21387058 | PDFSTATSENSEMBLUCSC | 174 | 7 | 26991 | tss | 0.187027 | 0.0339212 | Not queried | 2.04 | 5.35e-01 | 0.6176455124 |
|  | 13 | 20735139 | 20735532 | PDFSTATSENSEMBLUCSC | 394 | 8 | 11569 | island | 0.005249 | 0.0018646 | Not queried | 2.04 | 4.99e-02 | 0.2315877671 |
|  | 8 | 144069457 | 144069888 | PDFSTATSENSEMBLUCSC | 432 | 6 | 39470 | gene | 0.458425 | 0.8325015 | Not queried | 2.04 | 1.80e-01 | 0.3826488233 |
|  | 6 | 28601269 | 28601519 | PDFSTATSENSEMBLUCSC | 251 | 14 | 35592 | island | 0.813475 | 0.9510254 | Not queried | 2.04 | 4.97e-02 | 0.2315877671 |
|  | 17 | 42431627 | 42432165 | PDFSTATSENSEMBLUCSC | 539 | 5 | 16617 | gene;island | 0.004949 | 0.0270820 | Not queried | 2.04 | 4.21e-01 | 0.5404252807 |
|  | 17 | 6358363 | 6358599 | PDFSTATSENSEMBLUCSC | 237 | 4 | 16373 | gene;island | 0.279115 | 0.4731571 | Not queried | 2.04 | 2.00e-01 | 0.3826488233 |
|  | 20 | 26188908 | 26188997 | PDFSTATSENSEMBLUCSC | 90 | 7 | 26067 | island | 0.166931 | 0.3922957 | Not queried | 2.04 | 1.11e-02 | 0.1821650572 |
|  | 13 | 112322853 | 112323195 | PDFSTATSENSEMBLUCSC | 343 | 4 | 11902 | island | 0.547088 | 0.8224090 | Not queried | 2.04 | 2.00e-01 | 0.3826488233 |
|  | 12 | 121570677 | 121571068 | PDFSTATSENSEMBLUCSC | 392 | 4 | 9362 | gene | 0.051569 | 0.0277278 | Not queried | 2.04 | 4.86e-01 | 0.5640816327 |
|  | 11 | 118135094 | 118135407 | PDFSTATSENSEMBLUCSC | 314 | 7 | 7729 | tss | 0.120378 | 0.4720755 | Not queried | 2.04 | 1.00e+00 | 1.0000000000 |
|  | 8 | 146024438 | 146024786 | PDFSTATSENSEMBLUCSC | 349 | 4 | 39530 | gene;island | 0.005985 | 0.0132274 | Not queried | 2.04 | 6.86e-01 | 0.7223604052 |
|  | 2 | 171784945 | 171785124 | PDFSTATSENSEMBLUCSC | 180 | 4 | 23710 | tss;island | 0.743451 | 0.6884419 | Not queried | 2.03 | 8.86e-01 | 0.8941250130 |
|  | 22 | 24823389 | 24823554 | PDFSTATSENSEMBLUCSC | 166 | 6 | 27361 | island | 0.018442 | 0.0497011 | Not queried | 2.03 | 4.11e-02 | 0.2180389943 |
|  | 3 | 62861071 | 62861311 | PDFSTATSENSEMBLUCSC | 241 | 6 | 28428 | tss | 0.040649 | 0.0337872 | Not queried | 2.03 | 6.99e-01 | 0.7290267580 |
|  | 16 | 88747591 | 88747919 | PDFSTATSENSEMBLUCSC | 329 | 4 | 14911 | gene;island | 0.464832 | 0.1795408 | Not queried | 2.03 | 1.14e-01 | 0.3123457953 |
|  | 7 | 99766203 | 99766665 | PDFSTATSENSEMBLUCSC | 463 | 6 | 38753 | island | 0.152203 | 0.5441352 | Not queried | 2.03 | 3.10e-01 | 0.4672865596 |
|  | 2 | 176981919 | 176982342 | PDFSTATSENSEMBLUCSC | 424 | 4 | 22936 | gene;island | 0.071679 | 0.3791765 | Not queried | 2.03 | 1.14e-01 | 0.3123457953 |
|  | 19 | 18496756 | 18497201 | PDFSTATSENSEMBLUCSC | 446 | 8 | 21778 | island | 0.353694 | 0.0562590 | Not queried | 2.03 | 2.79e-01 | 0.4672865596 |
|  | 11 | 61447487 | 61447938 | PDFSTATSENSEMBLUCSC | 452 | 6 | 8330 | island | 0.039408 | 0.0050749 | Not queried | 2.03 | 6.49e-02 | 0.2477578846 |
|  | 1 | 153643479 | 153643822 | PDFSTATSENSEMBLUCSC | 344 | 8 | 1949 | tss | 0.004012 | 0.0065817 | Not queried | 2.03 | 5.74e-01 | 0.6460388787 |
|  | 22 | 43583726 | 43583855 | PDFSTATSENSEMBLUCSC | 130 | 4 | 27203 | tss;island | 0.002907 | 0.0084545 | Not queried | 2.03 | 2.00e-01 | 0.3826488233 |
|  | 1 | 181452618 | 181452839 | PDFSTATSENSEMBLUCSC | 222 | 4 | 4019 | island | 0.204332 | 0.2828037 | Not queried | 2.03 | 6.86e-01 | 0.7223604052 |
|  | 3 | 49170599 | 49170849 | PDFSTATSENSEMBLUCSC | 251 | 5 | 28320 | tss | 0.152634 | 0.0723897 | Not queried | 2.03 | 6.90e-01 | 0.7223604052 |
|  | 2 | 119600348 | 119600627 | PDFSTATSENSEMBLUCSC | 280 | 5 | 22840 | gene;island | 0.085682 | 0.1225383 | Not queried | 2.03 | 8.41e-01 | 0.8635565145 |
|  | 22 | 21386798 | 21387058 | PDFSTATSENSEMBLUCSC | 261 | 8 | 27330 | island | 0.128035 | 0.0262249 | Not queried | 2.03 | 4.42e-01 | 0.5640816327 |
|  | 6 | 34524095 | 34524290 | PDFSTATSENSEMBLUCSC | 196 | 4 | 34920 | tss | 0.203285 | 0.5893199 | Not queried | 2.03 | 2.86e-02 | 0.1830867741 |
|  | 17 | 79304498 | 79304683 | PDFSTATSENSEMBLUCSC | 186 | 7 | 17737 | tss | 0.305353 | 0.5774052 | Not queried | 2.03 | 7.28e-02 | 0.2713481487 |
|  | 3 | 123603306 | 123603618 | PDFSTATSENSEMBLUCSC | 313 | 6 | 28547 | tss;island | 0.016252 | 0.0340170 | Not queried | 2.03 | 1.80e-01 | 0.3826488233 |
|  | 1 | 45452509 | 45452605 | PDFSTATSENSEMBLUCSC | 97 | 4 | 1578 | tss | 0.275807 | 0.0569409 | Not queried | 2.02 | 1.00e+00 | 1.0000000000 |
|  | 10 | 118386951 | 118387518 | PDFSTATSENSEMBLUCSC | 568 | 5 | 4776 | gene;island | 0.402633 | 0.0766412 | Not queried | 2.02 | 3.10e-01 | 0.4672865596 |
|  | 4 | 8087178 | 8087448 | PDFSTATSENSEMBLUCSC | 271 | 4 | 29946 | gene;island | 0.145530 | 0.5275719 | Not queried | 2.02 | 5.71e-02 | 0.2315877671 |
|  | 2 | 237120069 | 237120313 | PDFSTATSENSEMBLUCSC | 245 | 4 | 23090 | gene;island | 0.416691 | 0.6071160 | Not queried | 2.02 | 3.43e-01 | 0.4672865596 |
|  | 16 | 84423876 | 84424282 | PDFSTATSENSEMBLUCSC | 407 | 4 | 14869 | gene | 0.690273 | 0.8976316 | Not queried | 2.02 | 3.43e-01 | 0.4672865596 |
|  | 19 | 37997675 | 37998171 | PDFSTATSENSEMBLUCSC | 497 | 11 | 21957 | island | 0.014403 | 0.0023956 | Not queried | 2.02 | 4.73e-02 | 0.2315877671 |
|  | 5 | 1394296 | 1394633 | PDFSTATSENSEMBLUCSC | 338 | 5 | 31536 | gene | 0.061907 | 0.5325649 | Not queried | 2.02 | 2.22e-01 | 0.3935758299 |
|  | 1 | 43855376 | 43855471 | PDFSTATSENSEMBLUCSC | 96 | 5 | 478 | gene | 0.002739 | 0.0069994 | Not queried | 2.02 | 5.48e-01 | 0.6179718485 |
|  | 15 | 95869918 | 95870475 | PDFSTATSENSEMBLUCSC | 558 | 9 | 14484 | island | 0.059046 | 0.1767330 | Not queried | 2.02 | 7.70e-02 | 0.2825346647 |
|  | 6 | 169951905 | 169952299 | PDFSTATSENSEMBLUCSC | 395 | 4 | 34569 | gene | 0.942031 | 0.9821104 | Not queried | 2.02 | 1.14e-01 | 0.3123457953 |
|  | 19 | 49622361 | 49622717 | PDFSTATSENSEMBLUCSC | 357 | 6 | 20941 | tss;island | 0.006717 | 0.0059772 | Not queried | 2.02 | 3.94e-01 | 0.5236526859 |
|  | 14 | 34420102 | 34420683 | PDFSTATSENSEMBLUCSC | 582 | 10 | 12772 | island | 0.021651 | 0.0072902 | Not queried | 2.02 | 4.33e-02 | 0.2287547702 |
|  | 5 | 3605783 | 3606081 | PDFSTATSENSEMBLUCSC | 299 | 5 | 32668 | island | 0.179798 | 0.0666601 | Not queried | 2.02 | 2.22e-01 | 0.3935758299 |
|  | 3 | 26663551 | 26663885 | PDFSTATSENSEMBLUCSC | 335 | 6 | 28194 | tss;island | 0.109079 | 0.3296150 | Not queried | 2.02 | 4.85e-01 | 0.5640816327 |
|  | 5 | 122426096 | 122426400 | PDFSTATSENSEMBLUCSC | 305 | 4 | 31729 | gene;island | 0.214461 | 0.0502888 | Not queried | 2.02 | 2.00e-01 | 0.3826488233 |
|  | 2 | 27712555 | 27713092 | PDFSTATSENSEMBLUCSC | 538 | 11 | 24187 | island | 0.005316 | 0.0167768 | Not queried | 2.02 | 7.59e-02 | 0.2811835917 |
|  | 1 | 16259191 | 16259499 | PDFSTATSENSEMBLUCSC | 309 | 4 | 256 | gene;island | 0.799862 | 0.9738319 | Not queried | 2.02 | 8.86e-01 | 0.8941250130 |
|  | 1 | 76540438 | 76540641 | PDFSTATSENSEMBLUCSC | 204 | 4 | 591 | gene;island | 0.035708 | 0.0105034 | Not queried | 2.02 | 2.00e-01 | 0.3826488233 |
|  | 20 | 61809557 | 61809932 | PDFSTATSENSEMBLUCSC | 376 | 9 | 26343 | island | 0.063572 | 0.0769251 | Not queried | 2.02 | 4.36e-01 | 0.5589686471 |
|  | 6 | 160388924 | 160389126 | PDFSTATSENSEMBLUCSC | 203 | 4 | 35325 | tss;island | 0.960219 | 0.7794676 | Not queried | 2.02 | 6.86e-01 | 0.7223604052 |
|  | 3 | 133392794 | 133393240 | PDFSTATSENSEMBLUCSC | 447 | 4 | 29490 | island | 0.271582 | 0.4833957 | Not queried | 2.02 | 4.86e-01 | 0.5640816327 |
|  | 16 | 56691551 | 56691849 | PDFSTATSENSEMBLUCSC | 299 | 7 | 15250 | tss | 0.006276 | 0.0305222 | Not queried | 2.01 | 7.28e-02 | 0.2713481487 |
|  | 5 | 1093580 | 1093797 | PDFSTATSENSEMBLUCSC | 218 | 4 | 31523 | gene;island | 0.963393 | 0.8794946 | Not queried | 2.01 | 1.14e-01 | 0.3123457953 |
|  | 18 | 42643730 | 42643936 | PDFSTATSENSEMBLUCSC | 207 | 5 | 19097 | gene;island | 0.974794 | 0.9127336 | Not queried | 2.01 | 9.52e-02 | 0.3104222821 |
|  | 1 | 155294935 | 155295267 | PDFSTATSENSEMBLUCSC | 333 | 4 | 805 | gene;tss;island | 0.038679 | 0.0238238 | Not queried | 2.01 | 6.86e-01 | 0.7223604052 |
|  | 14 | 57261601 | 57262177 | PDFSTATSENSEMBLUCSC | 577 | 5 | 12874 | island | 0.119282 | 0.2884768 | Not queried | 2.01 | 9.52e-02 | 0.3104222821 |
|  | 5 | 87963342 | 87963592 | PDFSTATSENSEMBLUCSC | 251 | 4 | 31676 | gene | 0.026117 | 0.0913248 | Not queried | 2.01 | 3.43e-01 | 0.4672865596 |
|  | 1 | 161993675 | 161994363 | PDFSTATSENSEMBLUCSC | 689 | 10 | 2056 | tss | 0.044262 | 0.0081811 | Not queried | 2.01 | 6.84e-03 | 0.1589602024 |
|  | 2 | 3704363 | 3704793 | PDFSTATSENSEMBLUCSC | 431 | 7 | 23164 | tss | 0.074164 | 0.8879860 | Not queried | 2.01 | 1.65e-01 | 0.3826488233 |
|  | 14 | 106857188 | 106857655 | PDFSTATSENSEMBLUCSC | 468 | 4 | 13247 | island | 0.874849 | 0.9697678 | Not queried | 2.01 | 2.00e-01 | 0.3826488233 |
|  | 5 | 1342124 | 1342200 | PDFSTATSENSEMBLUCSC | 77 | 4 | 31535 | gene;island | 0.059199 | 0.2345177 | Not queried | 2.01 | 1.14e-01 | 0.3123457953 |
|  | 12 | 49371987 | 49372474 | PDFSTATSENSEMBLUCSC | 488 | 9 | 10380 | island | 0.139749 | 0.0417224 | Not queried | 2.01 | 2.58e-01 | 0.4381244265 |
|  | 2 | 224866411 | 224866636 | PDFSTATSENSEMBLUCSC | 226 | 6 | 23054 | gene | 0.638052 | 0.8973144 | Not queried | 2.01 | 1.80e-01 | 0.3826488233 |
|  | 16 | 66955873 | 66956242 | PDFSTATSENSEMBLUCSC | 370 | 5 | 14784 | gene;island | 0.672210 | 0.8948975 | Not queried | 2.01 | 5.48e-01 | 0.6179718485 |
|  | 1 | 55007670 | 55008516 | PDFSTATSENSEMBLUCSC | 847 | 11 | 3299 | island | 0.045250 | 0.0204079 | Not queried | 2.00 | 1.04e-03 | 0.0821403610 |
|  | 2 | 9533573 | 9533887 | PDFSTATSENSEMBLUCSC | 315 | 5 | 22563 | gene;island | 0.768865 | 0.9294884 | Not queried | 2.00 | 9.52e-02 | 0.3104222821 |
|  | 15 | 39871808 | 39872186 | PDFSTATSENSEMBLUCSC | 379 | 7 | 13576 | tss;island | 0.386703 | 0.7069689 | Not queried | 2.00 | 7.28e-02 | 0.2713481487 |
|  | 12 | 46123553 | 46123850 | PDFSTATSENSEMBLUCSC | 298 | 4 | 10345 | island | 0.009375 | 0.0072977 | Not queried | 2.00 | 4.86e-01 | 0.5640816327 |
|  | 5 | 140797172 | 140797286 | PDFSTATSENSEMBLUCSC | 115 | 4 | 31833 | gene;island | 0.025098 | 0.0903849 | Not queried | 2.00 | 4.86e-01 | 0.5640816327 |
|  | 17 | 75277207 | 75277435 | PDFSTATSENSEMBLUCSC | 229 | 5 | 17695 | tss;island | 0.003099 | 0.0012535 | Not queried | 2.00 | 5.48e-01 | 0.6179718485 |
|  | 10 | 101292403 | 101292766 | PDFSTATSENSEMBLUCSC | 364 | 10 | 6012 | island | 0.015943 | 0.0062590 | Not queried | 2.00 | 1.85e-02 | 0.1830867741 |
|  | 6 | 29580896 | 29581228 | PDFSTATSENSEMBLUCSC | 333 | 7 | 33672 | gene | 0.189252 | 0.6283002 | Not queried | 2.00 | 2.62e-02 | 0.1830867741 |
|  | 10 | 86001241 | 86001560 | PDFSTATSENSEMBLUCSC | 320 | 7 | 5194 | tss | 0.031742 | 0.1157977 | Not queried | 2.00 | 3.83e-01 | 0.5168893301 |
|  | 19 | 38924045 | 38924330 | PDFSTATSENSEMBLUCSC | 286 | 7 | 20755 | tss | 0.083490 | 0.3009042 | Not queried | 2.00 | 3.18e-01 | 0.4672865596 |
|  | 7 | 96632294 | 96632841 | PDFSTATSENSEMBLUCSC | 548 | 5 | 37101 | gene;island | 0.666707 | 0.1175437 | Not queried | 2.00 | 1.51e-01 | 0.3618000441 |
|  | 10 | 77161525 | 77161705 | PDFSTATSENSEMBLUCSC | 181 | 7 | 4642 | gene;tss;island | 0.019673 | 0.1064301 | Not queried | 2.00 | 1.11e-02 | 0.1821650572 |
|  | 4 | 188916496 | 188916943 | PDFSTATSENSEMBLUCSC | 448 | 11 | 31491 | island | 0.330975 | 0.0666352 | Not queried | 2.00 | 1.92e-02 | 0.1830867741 |
|  | 4 | 55093612 | 55094011 | PDFSTATSENSEMBLUCSC | 400 | 6 | 31054 | island | 0.204852 | 0.0101228 | Not queried | 2.00 | 1.80e-01 | 0.3826488233 |
|  | 1 | 16345293 | 16345491 | PDFSTATSENSEMBLUCSC | 199 | 4 | 1278 | tss | 0.360077 | 0.6272561 | Not queried | 2.00 | 5.71e-02 | 0.2315877671 |
|  | 19 | 407758 | 408233 | PDFSTATSENSEMBLUCSC | 476 | 4 | 19625 | gene;island | 0.617548 | 0.9301426 | Not queried | 2.00 | 1.14e-01 | 0.3123457953 |
|  | 19 | 16830613 | 16830749 | PDFSTATSENSEMBLUCSC | 137 | 4 | 20575 | tss | 0.574857 | 0.0942908 | Not queried | 2.00 | 3.43e-01 | 0.4672865596 |
|  | 1 | 24514218 | 24514557 | PDFSTATSENSEMBLUCSC | 340 | 5 | 1357 | tss;island | 0.026862 | 0.0259515 | Not queried | 2.00 | 5.48e-01 | 0.6179718485 |
|  | 1 | 45769308 | 45769557 | PDFSTATSENSEMBLUCSC | 250 | 5 | 1582 | tss | 0.002697 | 0.0055029 | Not queried | 1.99 | 6.90e-01 | 0.7223604052 |
|  | 2 | 210636350 | 210636560 | PDFSTATSENSEMBLUCSC | 211 | 5 | 23852 | tss | 0.159382 | 0.0201491 | Not queried | 1.99 | 5.56e-02 | 0.2315877671 |
|  | 17 | 22191704 | 22191979 | PDFSTATSENSEMBLUCSC | 276 | 4 | 18131 | island | 0.330859 | 0.6620744 | Not queried | 1.99 | 2.00e-01 | 0.3826488233 |
|  | 1 | 22919873 | 22920169 | PDFSTATSENSEMBLUCSC | 297 | 4 | 307 | gene;island | 0.141827 | 0.6711594 | Not queried | 1.99 | 1.14e-01 | 0.3123457953 |
|  | 16 | 54318044 | 54318314 | PDFSTATSENSEMBLUCSC | 271 | 4 | 14751 | gene;island | 0.033533 | 0.2020581 | Not queried | 1.99 | 4.86e-01 | 0.5640816327 |
|  | 6 | 26987575 | 26988289 | PDFSTATSENSEMBLUCSC | 715 | 11 | 33615 | gene;island | 0.141098 | 0.4341912 | Not queried | 1.99 | 1.71e-01 | 0.3826488233 |
|  | 22 | 38863905 | 38864244 | PDFSTATSENSEMBLUCSC | 340 | 5 | 27490 | island | 0.048483 | 0.0116921 | Not queried | 1.99 | 6.90e-01 | 0.7223604052 |
|  | 7 | 45066738 | 45067057 | PDFSTATSENSEMBLUCSC | 320 | 5 | 36997 | gene;tss | 0.022754 | 0.0232528 | Not queried | 1.99 | 4.21e-01 | 0.5404252807 |
|  | 12 | 54409207 | 54409770 | PDFSTATSENSEMBLUCSC | 564 | 12 | 9753 | tss;island | 0.005438 | 0.0109554 | Not queried | 1.99 | 4.43e-01 | 0.5640816327 |
|  | 7 | 50633725 | 50634252 | PDFSTATSENSEMBLUCSC | 528 | 6 | 37595 | tss | 0.146953 | 0.4032683 | Not queried | 1.99 | 1.32e-01 | 0.3397986247 |
|  | 22 | 21398553 | 21398740 | PDFSTATSENSEMBLUCSC | 188 | 4 | 26992 | tss;island | 0.209213 | 0.6496377 | Not queried | 1.99 | 3.43e-01 | 0.4672865596 |
|  | 14 | 29234890 | 29235469 | PDFSTATSENSEMBLUCSC | 580 | 11 | 12335 | tss;island | 0.079030 | 0.2028200 | Not queried | 1.99 | 8.47e-01 | 0.8692219522 |
|  | 16 | 86912525 | 86912782 | PDFSTATSENSEMBLUCSC | 258 | 4 | 16178 | island | 0.045312 | 0.1958081 | Not queried | 1.99 | 2.00e-01 | 0.3826488233 |
|  | 1 | 8086588 | 8086959 | PDFSTATSENSEMBLUCSC | 372 | 4 | 1220 | tss;island | 0.116567 | 0.3539901 | Not queried | 1.99 | 4.86e-01 | 0.5640816327 |
|  | 1 | 180600890 | 180601141 | PDFSTATSENSEMBLUCSC | 252 | 6 | 2126 | tss;island | 0.003498 | 0.0087014 | Not queried | 1.99 | 3.94e-01 | 0.5236526859 |
|  | 1 | 26880767 | 26881009 | PDFSTATSENSEMBLUCSC | 243 | 4 | 343 | gene;tss | 0.837664 | 0.9603613 | Not queried | 1.99 | 2.86e-02 | 0.1830867741 |
|  | 19 | 42806146 | 42806556 | PDFSTATSENSEMBLUCSC | 411 | 10 | 20067 | gene;island | 0.029879 | 0.1383191 | Not queried | 1.99 | 3.53e-01 | 0.4803603739 |
|  | 22 | 38054505 | 38054892 | PDFSTATSENSEMBLUCSC | 388 | 6 | 27466 | island | 0.004561 | 0.0111429 | Not queried | 1.99 | 2.40e-01 | 0.4133265075 |
|  | 17 | 36003216 | 36003443 | PDFSTATSENSEMBLUCSC | 228 | 5 | 16536 | gene | 0.002751 | 0.0049066 | Not queried | 1.99 | 2.22e-01 | 0.3935758299 |
|  | 1 | 228403127 | 228403515 | PDFSTATSENSEMBLUCSC | 389 | 5 | 1050 | gene;island | 0.906939 | 0.9502267 | Not queried | 1.99 | 2.22e-01 | 0.3935758299 |
|  | 5 | 95768915 | 95769079 | PDFSTATSENSEMBLUCSC | 165 | 7 | 32974 | island | 0.294900 | 0.1921766 | Not queried | 1.99 | 3.18e-01 | 0.4672865596 |
|  | 10 | 103985730 | 103986128 | PDFSTATSENSEMBLUCSC | 399 | 7 | 5318 | tss | 0.005845 | 0.0159470 | Not queried | 1.99 | 2.09e-01 | 0.3935758299 |
|  | 7 | 1407330 | 1407571 | PDFSTATSENSEMBLUCSC | 242 | 6 | 38100 | island | 0.398310 | 0.6138212 | Not queried | 1.99 | 2.40e-01 | 0.4133265075 |
|  | 1 | 1959442 | 1959832 | PDFSTATSENSEMBLUCSC | 391 | 6 | 52 | gene;island | 0.568534 | 0.8268992 | Not queried | 1.99 | 1.52e-02 | 0.1830867741 |
|  | 21 | 45232531 | 45232601 | PDFSTATSENSEMBLUCSC | 71 | 4 | 26579 | tss | 0.546439 | 0.8086574 | Not queried | 1.99 | 2.00e-01 | 0.3826488233 |
|  | 14 | 102026230 | 102026555 | PDFSTATSENSEMBLUCSC | 326 | 10 | 12635 | tss | 0.039890 | 0.1202916 | Not queried | 1.99 | 1.47e-02 | 0.1830867741 |
|  | 14 | 77227545 | 77227645 | PDFSTATSENSEMBLUCSC | 101 | 4 | 12524 | tss;island | 0.486226 | 0.1703028 | Not queried | 1.98 | 5.71e-02 | 0.2315877671 |
|  | 2 | 186603398 | 186603639 | PDFSTATSENSEMBLUCSC | 242 | 4 | 24947 | island | 0.040808 | 0.0985260 | Not queried | 1.98 | 4.86e-01 | 0.5640816327 |
|  | 19 | 1238119 | 1238538 | PDFSTATSENSEMBLUCSC | 420 | 6 | 20294 | tss;island | 0.075356 | 0.0346337 | Not queried | 1.98 | 9.37e-01 | 0.9445195058 |
|  | 6 | 112575212 | 112575482 | PDFSTATSENSEMBLUCSC | 271 | 5 | 34450 | gene;island | 0.031979 | 0.1157297 | Not queried | 1.98 | 2.22e-01 | 0.3935758299 |
|  | 11 | 86085623 | 86086101 | PDFSTATSENSEMBLUCSC | 479 | 7 | 8711 | island | 0.102191 | 0.0550710 | Not queried | 1.98 | 6.20e-01 | 0.6899391537 |
|  | 10 | 101290958 | 101291397 | PDFSTATSENSEMBLUCSC | 440 | 4 | 6011 | island | 0.124484 | 0.3033880 | Not queried | 1.98 | 6.86e-01 | 0.7223604052 |
|  | 1 | 117664976 | 117665186 | PDFSTATSENSEMBLUCSC | 211 | 4 | 1869 | tss;island | 0.520311 | 0.2272376 | Not queried | 1.98 | 4.86e-01 | 0.5640816327 |
|  | 17 | 56355299 | 56355431 | PDFSTATSENSEMBLUCSC | 133 | 4 | 16709 | gene;island | 0.632258 | 0.8865600 | Not queried | 1.98 | 2.00e-01 | 0.3826488233 |
|  | 6 | 27463112 | 27463347 | PDFSTATSENSEMBLUCSC | 236 | 4 | 35558 | island | 0.015235 | 0.0404941 | Not queried | 1.98 | 2.86e-02 | 0.1830867741 |
|  | 5 | 1253833 | 1254048 | PDFSTATSENSEMBLUCSC | 216 | 4 | 31529 | gene;island | 0.838629 | 0.4377513 | Not queried | 1.98 | 4.86e-01 | 0.5640816327 |
|  | 10 | 81154142 | 81154192 | PDFSTATSENSEMBLUCSC | 51 | 4 | 4652 | gene | 0.495478 | 0.7937731 | Not queried | 1.98 | 2.86e-02 | 0.1830867741 |
|  | 10 | 15130387 | 15130767 | PDFSTATSENSEMBLUCSC | 381 | 4 | 4511 | gene | 0.048964 | 0.0280726 | Not queried | 1.98 | 1.14e-01 | 0.3123457953 |
|  | 18 | 55102849 | 55102925 | PDFSTATSENSEMBLUCSC | 77 | 5 | 19532 | island | 0.020801 | 0.0969011 | Not queried | 1.98 | 1.51e-01 | 0.3618000441 |
|  | 11 | 107460901 | 107461225 | PDFSTATSENSEMBLUCSC | 325 | 4 | 7662 | tss;island | 0.022791 | 0.0273348 | Not queried | 1.98 | 6.86e-01 | 0.7223604052 |
|  | 22 | 19712623 | 19712750 | PDFSTATSENSEMBLUCSC | 128 | 4 | 27302 | island | 0.659141 | 0.8430542 | Not queried | 1.98 | 4.86e-01 | 0.5640816327 |
|  | 6 | 33156779 | 33157222 | PDFSTATSENSEMBLUCSC | 444 | 10 | 34213 | gene;island | 0.224646 | 0.5127815 | Not queried | 1.98 | 1.43e-01 | 0.3618000441 |
|  | 1 | 211667297 | 211667751 | PDFSTATSENSEMBLUCSC | 455 | 5 | 2239 | tss | 0.634581 | 0.7423326 | Not queried | 1.98 | 4.21e-01 | 0.5404252807 |
|  | 11 | 3187271 | 3188016 | PDFSTATSENSEMBLUCSC | 746 | 24 | 7092 | tss;island | 0.393021 | 0.7006352 | Not queried | 1.98 | 2.30e-02 | 0.1830867741 |
|  | 18 | 44259787 | 44260091 | PDFSTATSENSEMBLUCSC | 305 | 4 | 19104 | gene;island | 0.688430 | 0.9017504 | Not queried | 1.98 | 1.14e-01 | 0.3123457953 |
|  | 7 | 151421620 | 151422150 | PDFSTATSENSEMBLUCSC | 531 | 6 | 37296 | gene;island | 0.580765 | 0.7888995 | Not queried | 1.98 | 3.94e-01 | 0.5236526859 |
|  | 17 | 27045043 | 27045176 | PDFSTATSENSEMBLUCSC | 134 | 5 | 16500 | gene | 0.020503 | 0.0742646 | Not queried | 1.98 | 9.52e-02 | 0.3104222821 |
|  | 14 | 45431486 | 45431885 | PDFSTATSENSEMBLUCSC | 400 | 5 | 12073 | gene | 0.011616 | 0.0033594 | Not queried | 1.98 | 1.51e-01 | 0.3618000441 |
|  | 7 | 134464242 | 134464411 | PDFSTATSENSEMBLUCSC | 170 | 4 | 37231 | gene | 0.504183 | 0.8016365 | Not queried | 1.98 | 3.43e-01 | 0.4672865596 |
|  | 2 | 120282191 | 120282327 | PDFSTATSENSEMBLUCSC | 137 | 5 | 23595 | tss | 0.860998 | 0.9604909 | Not queried | 1.97 | 4.21e-01 | 0.5404252807 |
|  | 5 | 115297690 | 115298079 | PDFSTATSENSEMBLUCSC | 390 | 5 | 32232 | tss | 0.551634 | 0.0414134 | Not queried | 1.97 | 4.21e-01 | 0.5404252807 |
|  | 6 | 29815787 | 29815902 | PDFSTATSENSEMBLUCSC | 116 | 4 | 35637 | island | 0.059363 | 0.0146186 | Not queried | 1.97 | 3.43e-01 | 0.4672865596 |
|  | 20 | 46415306 | 46415859 | PDFSTATSENSEMBLUCSC | 554 | 12 | 25802 | tss;island | 0.022024 | 0.2907706 | Not queried | 1.97 | 1.78e-01 | 0.3826488233 |
|  | 1 | 156022836 | 156023044 | PDFSTATSENSEMBLUCSC | 209 | 4 | 812 | gene;island | 0.003933 | 0.0039620 | Not queried | 1.97 | 4.86e-01 | 0.5640816327 |
|  | 2 | 27256009 | 27256285 | PDFSTATSENSEMBLUCSC | 277 | 4 | 22619 | gene;island | 0.003140 | 0.0023572 | Not queried | 1.97 | 3.43e-01 | 0.4672865596 |
|  | 18 | 44099028 | 44099340 | PDFSTATSENSEMBLUCSC | 313 | 5 | 19103 | gene | 0.019416 | 0.0864335 | Not queried | 1.97 | 5.48e-01 | 0.6179718485 |
|  | 11 | 6341842 | 6341908 | PDFSTATSENSEMBLUCSC | 67 | 7 | 7106 | tss | 0.012216 | 0.0335103 | Not queried | 1.97 | 1.65e-01 | 0.3826488233 |
|  | 1 | 118166013 | 118166402 | PDFSTATSENSEMBLUCSC | 390 | 4 | 712 | gene | 0.601917 | 0.8251253 | Not queried | 1.97 | 2.00e-01 | 0.3826488233 |
|  | 5 | 149545907 | 149546087 | PDFSTATSENSEMBLUCSC | 181 | 4 | 32426 | tss;island | 0.720236 | 0.9427034 | Not queried | 1.97 | 2.00e-01 | 0.3826488233 |
|  | 11 | 77791290 | 77791508 | PDFSTATSENSEMBLUCSC | 219 | 4 | 7589 | tss | 0.761491 | 0.3736713 | Not queried | 1.97 | 6.86e-01 | 0.7223604052 |
|  | 2 | 74875227 | 74875548 | PDFSTATSENSEMBLUCSC | 322 | 8 | 23465 | tss;island | 0.531092 | 0.3321873 | Not queried | 1.97 | 2.79e-01 | 0.4672865596 |
|  | 19 | 18902081 | 18902221 | PDFSTATSENSEMBLUCSC | 141 | 6 | 21794 | island | 0.232504 | 0.4916602 | Not queried | 1.97 | 1.80e-01 | 0.3826488233 |
|  | 11 | 130029478 | 130029684 | PDFSTATSENSEMBLUCSC | 207 | 5 | 8957 | island | 0.118856 | 0.1073045 | Not queried | 1.97 | 5.48e-01 | 0.6179718485 |
|  | 8 | 85094742 | 85094923 | PDFSTATSENSEMBLUCSC | 182 | 4 | 39787 | tss;island | 0.053784 | 0.0123299 | Not queried | 1.97 | 3.43e-01 | 0.4672865596 |
|  | 17 | 27045043 | 27045302 | PDFSTATSENSEMBLUCSC | 260 | 6 | 17218 | tss;island | 0.019216 | 0.0696603 | Not queried | 1.97 | 1.80e-01 | 0.3826488233 |
|  | 19 | 11465809 | 11466020 | PDFSTATSENSEMBLUCSC | 212 | 4 | 20469 | tss | 0.030276 | 0.0216669 | Not queried | 1.97 | 4.86e-01 | 0.5640816327 |
|  | 17 | 29032782 | 29033187 | PDFSTATSENSEMBLUCSC | 406 | 4 | 18179 | island | 0.092352 | 0.2551416 | Not queried | 1.97 | 6.86e-01 | 0.7223604052 |
|  | 6 | 33161377 | 33161589 | PDFSTATSENSEMBLUCSC | 213 | 5 | 34217 | gene | 0.460392 | 0.5272291 | Not queried | 1.97 | 2.22e-01 | 0.3935758299 |
|  | 11 | 10326100 | 10326342 | PDFSTATSENSEMBLUCSC | 243 | 4 | 7142 | tss;island | 0.028337 | 0.0766347 | Not queried | 1.96 | 2.00e-01 | 0.3826488233 |
|  | 2 | 108602784 | 108603244 | PDFSTATSENSEMBLUCSC | 461 | 9 | 24599 | island | 0.081418 | 0.4101483 | Not queried | 1.96 | 1.88e-02 | 0.1830867741 |
|  | 16 | 62069806 | 62070365 | PDFSTATSENSEMBLUCSC | 560 | 11 | 15985 | island | 0.237570 | 0.0805091 | Not queried | 1.96 | 1.93e-01 | 0.3826488233 |
|  | 19 | 38210837 | 38210932 | PDFSTATSENSEMBLUCSC | 96 | 4 | 20744 | tss | 0.032341 | 0.0107201 | Not queried | 1.96 | 1.14e-01 | 0.3123457953 |
|  | 6 | 30126161 | 30126535 | PDFSTATSENSEMBLUCSC | 375 | 4 | 33733 | gene | 0.371910 | 0.8792458 | Not queried | 1.96 | 6.86e-01 | 0.7223604052 |
|  | 11 | 73498584 | 73499134 | PDFSTATSENSEMBLUCSC | 551 | 5 | 8644 | island | 0.019097 | 0.0090291 | Not queried | 1.96 | 1.00e+00 | 1.0000000000 |
|  | 14 | 103739856 | 103740085 | PDFSTATSENSEMBLUCSC | 230 | 4 | 13185 | island | 0.038005 | 0.2042497 | Not queried | 1.96 | 1.14e-01 | 0.3123457953 |
|  | 3 | 197463810 | 197464306 | PDFSTATSENSEMBLUCSC | 497 | 7 | 28823 | tss | 0.001835 | 0.0016086 | Not queried | 1.96 | 4.56e-01 | 0.5640816327 |
|  | 11 | 1854174 | 1854368 | PDFSTATSENSEMBLUCSC | 195 | 4 | 7046 | tss | 0.018490 | 0.3608698 | Not queried | 1.96 | 6.86e-01 | 0.7223604052 |
|  | 10 | 104535792 | 104536121 | PDFSTATSENSEMBLUCSC | 330 | 9 | 4738 | gene | 0.277101 | 0.0968229 | Not queried | 1.96 | 4.36e-01 | 0.5589686471 |
|  | 2 | 29337946 | 29338258 | PDFSTATSENSEMBLUCSC | 313 | 9 | 23282 | tss | 0.015845 | 0.0578293 | Not queried | 1.96 | 1.06e-02 | 0.1810746987 |
|  | 2 | 133428422 | 133428993 | PDFSTATSENSEMBLUCSC | 572 | 7 | 22879 | gene | 0.183745 | 0.4667998 | Not queried | 1.96 | 6.20e-01 | 0.6899391537 |
|  | 11 | 3601010 | 3601214 | PDFSTATSENSEMBLUCSC | 205 | 4 | 7998 | island | 0.460884 | 0.7276309 | Not queried | 1.96 | 2.00e-01 | 0.3826488233 |
|  | 15 | 50716705 | 50716929 | PDFSTATSENSEMBLUCSC | 225 | 4 | 13347 | gene;island | 0.006775 | 0.0033423 | Not queried | 1.96 | 6.86e-01 | 0.7223604052 |
|  | 6 | 33360495 | 33360836 | PDFSTATSENSEMBLUCSC | 342 | 5 | 34272 | gene;island | 0.076133 | 0.0206651 | Not queried | 1.96 | 1.00e+00 | 1.0000000000 |
|  | 10 | 126289835 | 126290192 | PDFSTATSENSEMBLUCSC | 358 | 4 | 4807 | gene | 0.045040 | 0.2204544 | Not queried | 1.96 | 4.86e-01 | 0.5640816327 |
|  | 13 | 27333803 | 27334259 | PDFSTATSENSEMBLUCSC | 457 | 4 | 11113 | gene;island | 0.028303 | 0.0644026 | Not queried | 1.95 | 4.86e-01 | 0.5640816327 |
|  | 14 | 93798983 | 93799331 | PDFSTATSENSEMBLUCSC | 349 | 5 | 12188 | gene | 0.003617 | 0.0013198 | Not queried | 1.95 | 4.21e-01 | 0.5404252807 |
|  | 22 | 18484303 | 18484586 | PDFSTATSENSEMBLUCSC | 284 | 4 | 26782 | gene;island | 0.001622 | 0.0108866 | Not queried | 1.95 | 3.43e-01 | 0.4672865596 |
|  | 7 | 93520074 | 93520566 | PDFSTATSENSEMBLUCSC | 493 | 13 | 37715 | tss | 0.076074 | 0.0150092 | Not queried | 1.95 | 2.95e-02 | 0.1866936846 |
|  | 11 | 64067685 | 64067858 | PDFSTATSENSEMBLUCSC | 174 | 6 | 7394 | tss | 0.019558 | 0.0845352 | Not queried | 1.95 | 1.80e-01 | 0.3826488233 |
|  | 6 | 31324916 | 31324972 | PDFSTATSENSEMBLUCSC | 57 | 5 | 33870 | gene | 0.002524 | 0.0057183 | Not queried | 1.95 | 6.90e-01 | 0.7223604052 |
|  | 11 | 113184936 | 113185247 | PDFSTATSENSEMBLUCSC | 312 | 8 | 7702 | tss | 0.014096 | 0.0644177 | Not queried | 1.95 | 3.82e-01 | 0.5168893301 |
|  | 12 | 10875874 | 10876242 | PDFSTATSENSEMBLUCSC | 369 | 9 | 10242 | island | 0.003221 | 0.0016369 | Not queried | 1.95 | 7.70e-02 | 0.2825346647 |
|  | 1 | 21044685 | 21044945 | PDFSTATSENSEMBLUCSC | 261 | 6 | 1318 | tss;island | 0.026846 | 0.2123271 | Not queried | 1.95 | 1.32e-01 | 0.3397986247 |
|  | 4 | 84377120 | 84377400 | PDFSTATSENSEMBLUCSC | 281 | 4 | 30046 | gene | 0.002189 | 0.0028095 | Not queried | 1.95 | 8.86e-01 | 0.8941250130 |
|  | 10 | 119293083 | 119293550 | PDFSTATSENSEMBLUCSC | 468 | 5 | 4781 | gene;island | 0.013958 | 0.1169555 | Not queried | 1.95 | 6.90e-01 | 0.7223604052 |
|  | 5 | 68485086 | 68485282 | PDFSTATSENSEMBLUCSC | 197 | 4 | 32106 | tss | 0.008264 | 0.1440488 | Not queried | 1.95 | 4.86e-01 | 0.5640816327 |
|  | 1 | 854824 | 855046 | PDFSTATSENSEMBLUCSC | 223 | 6 | 1132 | tss | 0.327425 | 0.6559519 | Not queried | 1.95 | 2.16e-03 | 0.0934794372 |
|  | 16 | 89557017 | 89557420 | PDFSTATSENSEMBLUCSC | 404 | 8 | 15422 | tss;island | 0.005774 | 0.0020228 | Not queried | 1.95 | 1.95e-01 | 0.3826488233 |
|  | 5 | 168195173 | 168195460 | PDFSTATSENSEMBLUCSC | 288 | 4 | 31886 | gene | 0.142621 | 0.3706011 | Not queried | 1.95 | 2.00e-01 | 0.3826488233 |
|  | 5 | 27038707 | 27038836 | PDFSTATSENSEMBLUCSC | 130 | 4 | 32003 | tss | 0.240399 | 0.5459505 | Not queried | 1.95 | 3.43e-01 | 0.4672865596 |
|  | 11 | 134336336 | 134336476 | PDFSTATSENSEMBLUCSC | 141 | 4 | 8988 | island | 0.122970 | 0.2076573 | Not queried | 1.95 | 2.00e-01 | 0.3826488233 |
|  | 3 | 62363922 | 62364390 | PDFSTATSENSEMBLUCSC | 469 | 6 | 29251 | island | 0.068207 | 0.1487169 | Not queried | 1.95 | 1.80e-01 | 0.3826488233 |
|  | 3 | 71633826 | 71634044 | PDFSTATSENSEMBLUCSC | 219 | 4 | 28442 | tss;island | 0.015010 | 0.0028965 | Not queried | 1.95 | 2.00e-01 | 0.3826488233 |
|  | 7 | 117864720 | 117865112 | PDFSTATSENSEMBLUCSC | 393 | 4 | 37202 | gene | 0.612353 | 0.9218055 | Not queried | 1.95 | 2.00e-01 | 0.3826488233 |
|  | 7 | 155325552 | 155326102 | PDFSTATSENSEMBLUCSC | 551 | 6 | 37310 | gene;island | 0.233230 | 0.0355060 | Not queried | 1.95 | 2.40e-01 | 0.4133265075 |
|  | 1 | 145826877 | 145827062 | PDFSTATSENSEMBLUCSC | 186 | 4 | 728 | gene;island | 0.002695 | 0.0018718 | Not queried | 1.95 | 2.00e-01 | 0.3826488233 |
|  | 7 | 65878734 | 65879115 | PDFSTATSENSEMBLUCSC | 382 | 4 | 38534 | island | 0.044069 | 0.0066514 | Not queried | 1.94 | 3.43e-01 | 0.4672865596 |
|  | 12 | 115105220 | 115105710 | PDFSTATSENSEMBLUCSC | 491 | 5 | 10845 | island | 0.039170 | 0.1356963 | Not queried | 1.94 | 8.41e-01 | 0.8635565145 |
|  | 6 | 117198428 | 117198743 | PDFSTATSENSEMBLUCSC | 316 | 4 | 34453 | gene | 0.121119 | 0.4602089 | Not queried | 1.94 | 5.71e-02 | 0.2315877671 |
|  | 3 | 127174705 | 127175109 | PDFSTATSENSEMBLUCSC | 405 | 5 | 29443 | island | 0.019081 | 0.2724310 | Not queried | 1.94 | 1.51e-01 | 0.3618000441 |
|  | 11 | 94502349 | 94502879 | PDFSTATSENSEMBLUCSC | 531 | 8 | 6869 | gene;island | 0.178995 | 0.0266060 | Not queried | 1.94 | 1.95e-01 | 0.3826488233 |
|  | 1 | 71513494 | 71513789 | PDFSTATSENSEMBLUCSC | 296 | 6 | 1693 | tss;island | 0.214708 | 0.0274660 | Not queried | 1.94 | 2.40e-01 | 0.4133265075 |
|  | 1 | 214557246 | 214557497 | PDFSTATSENSEMBLUCSC | 252 | 5 | 1006 | gene;island | 0.496582 | 0.8339201 | Not queried | 1.94 | 5.56e-02 | 0.2315877671 |
|  | 6 | 31683051 | 31683131 | PDFSTATSENSEMBLUCSC | 81 | 5 | 34831 | tss | 0.278949 | 0.6078311 | Not queried | 1.94 | 9.52e-02 | 0.3104222821 |
|  | 2 | 48132755 | 48133053 | PDFSTATSENSEMBLUCSC | 299 | 5 | 24286 | island | 0.003085 | 0.0065989 | Not queried | 1.94 | 4.21e-01 | 0.5404252807 |
|  | 8 | 82024462 | 82024860 | PDFSTATSENSEMBLUCSC | 399 | 6 | 39783 | tss;island | 0.008255 | 0.0099803 | Not queried | 1.94 | 3.94e-01 | 0.5236526859 |
|  | 8 | 143823734 | 143824001 | PDFSTATSENSEMBLUCSC | 268 | 5 | 40642 | island | 0.450400 | 0.7522281 | Not queried | 1.94 | 9.52e-02 | 0.3104222821 |
|  | 7 | 4838902 | 4839249 | PDFSTATSENSEMBLUCSC | 348 | 5 | 36856 | gene | 0.725797 | 0.9106134 | Not queried | 1.94 | 3.10e-01 | 0.4672865596 |
|  | 2 | 220378996 | 220379260 | PDFSTATSENSEMBLUCSC | 265 | 9 | 23036 | gene;island | 0.009555 | 0.0227615 | Not queried | 1.94 | 9.39e-02 | 0.3104222821 |
|  | 1 | 230492737 | 230493133 | PDFSTATSENSEMBLUCSC | 397 | 5 | 1072 | gene;island | 0.542553 | 0.8466562 | Not queried | 1.94 | 2.22e-01 | 0.3935758299 |
|  | 7 | 129414627 | 129414894 | PDFSTATSENSEMBLUCSC | 268 | 11 | 37894 | tss | 0.531533 | 0.7829856 | Not queried | 1.94 | 1.04e-02 | 0.1806165940 |
|  | 6 | 10882927 | 10883105 | PDFSTATSENSEMBLUCSC | 179 | 4 | 34632 | tss;island | 0.370985 | 0.1954796 | Not queried | 1.94 | 8.86e-01 | 0.8941250130 |
|  | 2 | 89065319 | 89065379 | PDFSTATSENSEMBLUCSC | 61 | 4 | 23504 | tss | 0.205164 | 0.1159613 | Not queried | 1.94 | 3.43e-01 | 0.4672865596 |
|  | 17 | 4802692 | 4802906 | PDFSTATSENSEMBLUCSC | 215 | 6 | 17033 | tss | 0.021502 | 0.0106465 | Not queried | 1.94 | 2.40e-01 | 0.4133265075 |
|  | 7 | 157502370 | 157502620 | PDFSTATSENSEMBLUCSC | 251 | 4 | 37340 | gene;island | 0.248548 | 0.4086604 | Not queried | 1.94 | 4.86e-01 | 0.5640816327 |
|  | 10 | 100991959 | 100992222 | PDFSTATSENSEMBLUCSC | 264 | 4 | 4705 | gene;island | 0.175816 | 0.0444393 | Not queried | 1.94 | 1.14e-01 | 0.3123457953 |
|  | 3 | 150690183 | 150690456 | PDFSTATSENSEMBLUCSC | 274 | 6 | 28664 | tss | 0.472651 | 0.7477817 | Not queried | 1.94 | 1.80e-01 | 0.3826488233 |
|  | 8 | 98290148 | 98290601 | PDFSTATSENSEMBLUCSC | 454 | 9 | 40476 | island | 0.172124 | 0.0517428 | Not queried | 1.93 | 2.97e-01 | 0.4672865596 |
|  | 19 | 37157776 | 37157995 | PDFSTATSENSEMBLUCSC | 220 | 8 | 20729 | tss | 0.008310 | 0.0030423 | Not queried | 1.93 | 2.34e-01 | 0.4128374386 |
|  | 17 | 75243296 | 75243679 | PDFSTATSENSEMBLUCSC | 384 | 4 | 18803 | island | 0.016991 | 0.0172077 | Not queried | 1.93 | 1.00e+00 | 1.0000000000 |
|  | 17 | 62658750 | 62658919 | PDFSTATSENSEMBLUCSC | 170 | 4 | 17585 | tss;island | 0.055250 | 0.0141636 | Not queried | 1.93 | 1.14e-01 | 0.3123457953 |
|  | 20 | 55904633 | 55904768 | PDFSTATSENSEMBLUCSC | 136 | 5 | 25841 | tss | 0.752291 | 0.9467671 | Not queried | 1.93 | 4.21e-01 | 0.5404252807 |
|  | 11 | 27741916 | 27742454 | PDFSTATSENSEMBLUCSC | 539 | 9 | 6547 | gene;tss;island | 0.154067 | 0.1563225 | Not queried | 1.93 | 2.22e-01 | 0.3935758299 |
|  | 11 | 111847692 | 111847984 | PDFSTATSENSEMBLUCSC | 293 | 5 | 7695 | tss | 0.013809 | 0.0319009 | Not queried | 1.93 | 2.22e-01 | 0.3935758299 |
|  | 13 | 113119625 | 113119864 | PDFSTATSENSEMBLUCSC | 240 | 4 | 11922 | island | 0.808388 | 0.9247676 | Not queried | 1.93 | 3.43e-01 | 0.4672865596 |
|  | 6 | 166907748 | 166908071 | PDFSTATSENSEMBLUCSC | 324 | 4 | 34547 | gene | 0.349993 | 0.6439449 | Not queried | 1.93 | 1.14e-01 | 0.3123457953 |
|  | 7 | 63505584 | 63505871 | PDFSTATSENSEMBLUCSC | 288 | 7 | 38522 | island | 0.108387 | 0.3164707 | Not queried | 1.93 | 3.79e-02 | 0.2121545571 |
|  | 1 | 215256254 | 215256978 | PDFSTATSENSEMBLUCSC | 725 | 9 | 1009 | gene;island | 0.017198 | 0.0082082 | Not queried | 1.93 | 3.40e-01 | 0.4672865596 |
|  | 4 | 1642054 | 1642257 | PDFSTATSENSEMBLUCSC | 204 | 4 | 29873 | gene;island | 0.578090 | 0.8345070 | Not queried | 1.93 | 5.71e-02 | 0.2315877671 |
|  | 1 | 183387128 | 183387564 | PDFSTATSENSEMBLUCSC | 437 | 5 | 916 | gene | 0.055477 | 0.0220820 | Not queried | 1.93 | 1.51e-01 | 0.3618000441 |
|  | 6 | 26020672 | 26020714 | PDFSTATSENSEMBLUCSC | 43 | 4 | 34678 | tss;island | 0.000546 | 0.0020977 | Not queried | 1.93 | 8.86e-01 | 0.8941250130 |
|  | 19 | 58864846 | 58864929 | PDFSTATSENSEMBLUCSC | 84 | 4 | 20261 | gene;island | 0.769321 | 0.9280852 | Not queried | 1.93 | 2.86e-02 | 0.1830867741 |
|  | 6 | 143832933 | 143833251 | PDFSTATSENSEMBLUCSC | 319 | 7 | 35266 | tss | 0.518884 | 0.1022071 | Not queried | 1.93 | 3.83e-01 | 0.5168893301 |
|  | 6 | 710835 | 711405 | PDFSTATSENSEMBLUCSC | 571 | 4 | 35364 | island | 0.007138 | 0.0028602 | Not queried | 1.92 | 2.86e-02 | 0.1830867741 |
|  | 14 | 94088174 | 94088604 | PDFSTATSENSEMBLUCSC | 431 | 4 | 12191 | gene | 0.504780 | 0.8643646 | Not queried | 1.92 | 2.00e-01 | 0.3826488233 |
|  | 21 | 37442104 | 37442476 | PDFSTATSENSEMBLUCSC | 373 | 4 | 26668 | island | 0.035904 | 0.0084622 | Not queried | 1.92 | 2.00e-01 | 0.3826488233 |
|  | 4 | 380157 | 380529 | PDFSTATSENSEMBLUCSC | 373 | 4 | 30717 | island | 0.294166 | 0.6111464 | Not queried | 1.92 | 6.86e-01 | 0.7223604052 |
|  | 13 | 98829521 | 98829666 | PDFSTATSENSEMBLUCSC | 146 | 5 | 11508 | tss | 0.473666 | 0.8191273 | Not queried | 1.92 | 9.52e-02 | 0.3104222821 |
|  | 1 | 2984218 | 2984275 | PDFSTATSENSEMBLUCSC | 58 | 4 | 96 | gene | 0.235662 | 0.6433476 | Not queried | 1.92 | 1.14e-01 | 0.3123457953 |
|  | 15 | 85113863 | 85114479 | PDFSTATSENSEMBLUCSC | 617 | 8 | 14401 | island | 0.059852 | 0.0131265 | Not queried | 1.92 | 1.95e-01 | 0.3826488233 |
|  | 15 | 85114131 | 85114479 | PDFSTATSENSEMBLUCSC | 349 | 6 | 13867 | tss | 0.014012 | 0.0106960 | Not queried | 1.92 | 4.85e-01 | 0.5640816327 |
|  | 2 | 98329691 | 98330020 | PDFSTATSENSEMBLUCSC | 330 | 5 | 23531 | tss | 0.034523 | 0.2067331 | Not queried | 1.92 | 1.51e-01 | 0.3618000441 |
|  | 19 | 2525231 | 2525634 | PDFSTATSENSEMBLUCSC | 404 | 5 | 19690 | gene;island | 0.839606 | 0.9608977 | Not queried | 1.92 | 5.56e-02 | 0.2315877671 |
|  | 19 | 44860658 | 44861241 | PDFSTATSENSEMBLUCSC | 584 | 5 | 22108 | island | 0.533758 | 0.3245240 | Not queried | 1.92 | 5.48e-01 | 0.6179718485 |
|  | 1 | 150254280 | 150254546 | PDFSTATSENSEMBLUCSC | 267 | 5 | 1905 | tss;island | 0.020837 | 0.0341387 | Not queried | 1.92 | 8.41e-01 | 0.8635565145 |
|  | 12 | 89744471 | 89744877 | PDFSTATSENSEMBLUCSC | 407 | 8 | 9238 | gene;island | 0.016830 | 0.0879803 | Not queried | 1.92 | 6.50e-02 | 0.2477578846 |
|  | 11 | 32459428 | 32459954 | PDFSTATSENSEMBLUCSC | 527 | 4 | 6569 | gene;island | 0.279749 | 0.0503974 | Not queried | 1.92 | 6.86e-01 | 0.7223604052 |
|  | 4 | 96761264 | 96761796 | PDFSTATSENSEMBLUCSC | 533 | 5 | 30064 | gene | 0.564679 | 0.8012380 | Not queried | 1.92 | 3.10e-01 | 0.4672865596 |
|  | 16 | 62070072 | 62070365 | PDFSTATSENSEMBLUCSC | 294 | 9 | 15278 | tss | 0.237570 | 0.0947658 | Not queried | 1.92 | 4.89e-01 | 0.5677586445 |
|  | 15 | 43785364 | 43785537 | PDFSTATSENSEMBLUCSC | 174 | 7 | 13316 | gene;tss;island | 0.003032 | 0.0054954 | Not queried | 1.92 | 2.09e-01 | 0.3935758299 |
|  | 17 | 61042718 | 61043072 | PDFSTATSENSEMBLUCSC | 355 | 4 | 18639 | island | 0.539329 | 0.9161967 | Not queried | 1.92 | 6.86e-01 | 0.7223604052 |
|  | 11 | 57267101 | 57267244 | PDFSTATSENSEMBLUCSC | 144 | 4 | 6614 | gene;island | 0.605447 | 0.3352171 | Not queried | 1.92 | 2.86e-02 | 0.1830867741 |
|  | 12 | 68647289 | 68647590 | PDFSTATSENSEMBLUCSC | 302 | 6 | 9853 | tss | 0.751035 | 0.9372766 | Not queried | 1.92 | 3.10e-01 | 0.4672865596 |
|  | 6 | 17102310 | 17102678 | PDFSTATSENSEMBLUCSC | 369 | 6 | 35473 | island | 0.067495 | 0.2304071 | Not queried | 1.92 | 3.10e-01 | 0.4672865596 |
|  | 7 | 120968877 | 120969079 | PDFSTATSENSEMBLUCSC | 203 | 7 | 37861 | tss | 0.071248 | 0.4158600 | Not queried | 1.91 | 1.11e-02 | 0.1821650572 |
|  | 5 | 1286042 | 1286354 | PDFSTATSENSEMBLUCSC | 313 | 4 | 31534 | gene;island | 0.959752 | 0.8553669 | Not queried | 1.91 | 3.43e-01 | 0.4672865596 |
|  | 11 | 65657704 | 65657849 | PDFSTATSENSEMBLUCSC | 146 | 6 | 7450 | tss | 0.238271 | 0.0179440 | Not queried | 1.91 | 1.80e-01 | 0.3826488233 |
|  | 20 | 53092781 | 53093152 | PDFSTATSENSEMBLUCSC | 372 | 4 | 25459 | gene;island | 0.053121 | 0.0114995 | Not queried | 1.91 | 1.14e-01 | 0.3123457953 |
|  | 19 | 54371775 | 54372062 | PDFSTATSENSEMBLUCSC | 288 | 4 | 20208 | gene;tss;island | 0.002634 | 0.0041618 | Not queried | 1.91 | 2.00e-01 | 0.3826488233 |
|  | 13 | 50570018 | 50570250 | PDFSTATSENSEMBLUCSC | 233 | 4 | 11167 | gene;tss;island | 0.005574 | 0.0192182 | Not queried | 1.91 | 2.00e-01 | 0.3826488233 |
|  | 11 | 76092311 | 76092535 | PDFSTATSENSEMBLUCSC | 225 | 4 | 7574 | tss;island | 0.002992 | 0.0030843 | Not queried | 1.91 | 3.43e-01 | 0.4672865596 |
|  | 17 | 79905152 | 79905319 | PDFSTATSENSEMBLUCSC | 168 | 7 | 17762 | tss | 0.669460 | 0.9145035 | Not queried | 1.91 | 1.28e-01 | 0.3397986247 |
|  | 8 | 101661767 | 101662246 | PDFSTATSENSEMBLUCSC | 480 | 9 | 40498 | island | 0.040784 | 0.0071379 | Not queried | 1.91 | 6.25e-02 | 0.2477578846 |
|  | 8 | 101661918 | 101662246 | PDFSTATSENSEMBLUCSC | 329 | 7 | 39844 | tss | 0.084620 | 0.0071379 | Not queried | 1.91 | 2.09e-01 | 0.3935758299 |
|  | 5 | 87985809 | 87986067 | PDFSTATSENSEMBLUCSC | 259 | 4 | 32949 | island | 0.080803 | 0.0226861 | Not queried | 1.91 | 3.43e-01 | 0.4672865596 |
|  | 11 | 330872 | 331179 | PDFSTATSENSEMBLUCSC | 308 | 4 | 7843 | island | 0.557176 | 0.7790861 | Not queried | 1.91 | 3.43e-01 | 0.4672865596 |
|  | 7 | 82792053 | 82792411 | PDFSTATSENSEMBLUCSC | 359 | 9 | 38625 | island | 0.002022 | 0.0099143 | Not queried | 1.91 | 3.87e-01 | 0.5209559659 |
|  | 3 | 113160430 | 113160821 | PDFSTATSENSEMBLUCSC | 392 | 7 | 28506 | tss | 0.506058 | 0.1779857 | Not queried | 1.91 | 6.20e-01 | 0.6899391537 |
|  | 3 | 157155811 | 157156326 | PDFSTATSENSEMBLUCSC | 516 | 4 | 28008 | gene;island | 0.168911 | 0.0284821 | Not queried | 1.91 | 5.71e-02 | 0.2315877671 |
|  | 5 | 100238655 | 100238983 | PDFSTATSENSEMBLUCSC | 329 | 4 | 31702 | gene | 0.000173 | 0.0010354 | Not queried | 1.91 | 3.43e-01 | 0.4672865596 |
|  | 13 | 113540400 | 113540631 | PDFSTATSENSEMBLUCSC | 232 | 5 | 11262 | gene;island | 0.386447 | 0.5351819 | Not queried | 1.91 | 1.00e+00 | 1.0000000000 |
|  | 3 | 132756721 | 132757106 | PDFSTATSENSEMBLUCSC | 386 | 9 | 28599 | tss | 0.046028 | 0.1339320 | Not queried | 1.91 | 1.42e-02 | 0.1830867741 |
|  | 14 | 96180319 | 96181044 | PDFSTATSENSEMBLUCSC | 726 | 11 | 13113 | island | 0.014896 | 0.1478445 | Not queried | 1.91 | 1.93e-01 | 0.3826488233 |
|  | 15 | 26109204 | 26109614 | PDFSTATSENSEMBLUCSC | 411 | 12 | 13540 | tss;island | 0.400495 | 0.7032765 | Not queried | 1.91 | 6.84e-02 | 0.2600243423 |
|  | 22 | 45072455 | 45072724 | PDFSTATSENSEMBLUCSC | 270 | 4 | 26892 | gene | 0.076629 | 0.2372083 | Not queried | 1.91 | 6.86e-01 | 0.7223604052 |
|  | 20 | 1165065 | 1165435 | PDFSTATSENSEMBLUCSC | 371 | 5 | 25933 | island | 0.550017 | 0.4843414 | Not queried | 1.91 | 8.41e-01 | 0.8635565145 |
|  | 10 | 131356942 | 131357256 | PDFSTATSENSEMBLUCSC | 315 | 6 | 4827 | gene | 0.684864 | 0.8683282 | Not queried | 1.91 | 1.32e-01 | 0.3397986247 |
|  | 3 | 62305003 | 62305285 | PDFSTATSENSEMBLUCSC | 283 | 5 | 28425 | tss;island | 0.203835 | 0.1294322 | Not queried | 1.91 | 8.41e-01 | 0.8635565145 |
|  | 15 | 81616310 | 81616489 | PDFSTATSENSEMBLUCSC | 180 | 4 | 13451 | gene | 0.002737 | 0.0032577 | Not queried | 1.91 | 4.86e-01 | 0.5640816327 |
|  | 6 | 39902296 | 39902693 | PDFSTATSENSEMBLUCSC | 398 | 8 | 34960 | tss | 0.160021 | 0.0230110 | Not queried | 1.91 | 5.05e-01 | 0.5852592101 |
|  | 5 | 137475177 | 137475532 | PDFSTATSENSEMBLUCSC | 356 | 4 | 32313 | tss | 0.014759 | 0.1309918 | Not queried | 1.91 | 1.00e+00 | 1.0000000000 |
|  | 17 | 48172598 | 48172668 | PDFSTATSENSEMBLUCSC | 71 | 4 | 17492 | tss;island | 0.005535 | 0.0027489 | Not queried | 1.91 | 1.14e-01 | 0.3123457953 |
|  | 8 | 110703888 | 110704289 | PDFSTATSENSEMBLUCSC | 402 | 6 | 40541 | island | 0.011671 | 0.0872041 | Not queried | 1.90 | 3.10e-01 | 0.4672865596 |
|  | 5 | 139135602 | 139135961 | PDFSTATSENSEMBLUCSC | 360 | 4 | 33178 | island | 0.023920 | 0.0401517 | Not queried | 1.90 | 8.86e-01 | 0.8941250130 |
|  | 7 | 92672812 | 92673176 | PDFSTATSENSEMBLUCSC | 365 | 5 | 38665 | island | 0.482642 | 0.3556937 | Not queried | 1.90 | 5.48e-01 | 0.6179718485 |
|  | 1 | 46767565 | 46767971 | PDFSTATSENSEMBLUCSC | 407 | 4 | 511 | gene;island | 0.005071 | 0.0060037 | Not queried | 1.90 | 4.86e-01 | 0.5640816327 |
|  | 16 | 2198777 | 2199120 | PDFSTATSENSEMBLUCSC | 344 | 4 | 14601 | gene;island | 0.018793 | 0.0731259 | Not queried | 1.90 | 2.86e-02 | 0.1830867741 |
|  | 4 | 4405844 | 4406200 | PDFSTATSENSEMBLUCSC | 357 | 6 | 29916 | gene | 0.529348 | 0.6738702 | Not queried | 1.90 | 3.10e-01 | 0.4672865596 |
|  | 4 | 187509603 | 187510183 | PDFSTATSENSEMBLUCSC | 581 | 5 | 30216 | gene | 0.466449 | 0.8344066 | Not queried | 1.90 | 3.10e-01 | 0.4672865596 |
|  | 20 | 26188919 | 26188997 | PDFSTATSENSEMBLUCSC | 79 | 6 | 25655 | tss | 0.155762 | 0.3508526 | Not queried | 1.90 | 8.66e-03 | 0.1595382395 |
|  | 14 | 77732283 | 77732641 | PDFSTATSENSEMBLUCSC | 359 | 5 | 12150 | gene | 0.203222 | 0.5179161 | Not queried | 1.90 | 2.22e-01 | 0.3935758299 |
|  | 17 | 10560609 | 10560928 | PDFSTATSENSEMBLUCSC | 320 | 5 | 17134 | tss | 0.921918 | 0.9345011 | Not queried | 1.90 | 5.48e-01 | 0.6179718485 |
|  | 2 | 73429084 | 73429539 | PDFSTATSENSEMBLUCSC | 456 | 8 | 24412 | island | 0.124353 | 0.0358634 | Not queried | 1.90 | 1.05e-01 | 0.3123457953 |
|  | 3 | 72788183 | 72788482 | PDFSTATSENSEMBLUCSC | 300 | 4 | 29282 | island | 0.028150 | 0.0474910 | Not queried | 1.90 | 4.86e-01 | 0.5640816327 |
|  | 19 | 41018757 | 41018880 | PDFSTATSENSEMBLUCSC | 124 | 4 | 20043 | gene;island | 0.332625 | 0.1421107 | Not queried | 1.90 | 4.86e-01 | 0.5640816327 |
|  | 19 | 57306631 | 57307081 | PDFSTATSENSEMBLUCSC | 451 | 6 | 20239 | gene;island | 0.190490 | 0.3400953 | Not queried | 1.90 | 5.89e-01 | 0.6570484697 |
|  | 8 | 63997977 | 63998778 | PDFSTATSENSEMBLUCSC | 802 | 11 | 40337 | island | 0.046892 | 0.0121773 | Not queried | 1.90 | 4.10e-03 | 0.1459191215 |
|  | 5 | 149493008 | 149493258 | PDFSTATSENSEMBLUCSC | 251 | 6 | 32424 | tss | 0.336724 | 0.6299059 | Not queried | 1.90 | 2.60e-02 | 0.1830867741 |
|  | 8 | 25909171 | 25909599 | PDFSTATSENSEMBLUCSC | 429 | 6 | 40172 | island | 0.060144 | 0.1074702 | Not queried | 1.90 | 5.89e-01 | 0.6570484697 |
|  | 1 | 211306986 | 211307408 | PDFSTATSENSEMBLUCSC | 423 | 5 | 993 | gene;island | 0.013732 | 0.0366259 | Not queried | 1.90 | 2.22e-01 | 0.3935758299 |
|  | 8 | 24771273 | 24771645 | PDFSTATSENSEMBLUCSC | 373 | 4 | 39245 | gene | 0.354380 | 0.0960490 | Not queried | 1.90 | 1.14e-01 | 0.3123457953 |
|  | 4 | 298681 | 299370 | PDFSTATSENSEMBLUCSC | 690 | 9 | 30715 | island | 0.500183 | 0.3106543 | Not queried | 1.90 | 2.22e-01 | 0.3935758299 |
|  | 22 | 36784250 | 36784627 | PDFSTATSENSEMBLUCSC | 378 | 5 | 27107 | tss;island | 0.005793 | 0.0006386 | Not queried | 1.90 | 4.21e-01 | 0.5404252807 |
|  | 1 | 47696212 | 47696788 | PDFSTATSENSEMBLUCSC | 577 | 6 | 1607 | tss;island | 0.070647 | 0.0150852 | Not queried | 1.90 | 1.32e-01 | 0.3397986247 |
|  | 22 | 46659317 | 46659595 | PDFSTATSENSEMBLUCSC | 279 | 4 | 27232 | tss;island | 0.815085 | 0.9345517 | Not queried | 1.90 | 4.86e-01 | 0.5640816327 |
|  | 14 | 99664107 | 99664344 | PDFSTATSENSEMBLUCSC | 238 | 5 | 12201 | gene | 0.212224 | 0.6629196 | Not queried | 1.89 | 3.10e-01 | 0.4672865596 |
|  | 15 | 100882020 | 100882647 | PDFSTATSENSEMBLUCSC | 628 | 11 | 14507 | island | 0.160128 | 0.0280966 | Not queried | 1.89 | 3.36e-02 | 0.1970167151 |
|  | 4 | 102268799 | 102268957 | PDFSTATSENSEMBLUCSC | 159 | 5 | 30517 | tss | 0.007129 | 0.0078840 | Not queried | 1.89 | 6.90e-01 | 0.7223604052 |
|  | 18 | 47792917 | 47793175 | PDFSTATSENSEMBLUCSC | 259 | 6 | 19274 | tss | 0.009137 | 0.0186194 | Not queried | 1.89 | 8.18e-01 | 0.8469867211 |
|  | 3 | 147140754 | 147140930 | PDFSTATSENSEMBLUCSC | 177 | 4 | 29575 | island | 0.273214 | 0.0395355 | Not queried | 1.89 | 6.86e-01 | 0.7223604052 |
|  | 21 | 43736736 | 43737065 | PDFSTATSENSEMBLUCSC | 330 | 4 | 26566 | tss | 0.413560 | 0.7154416 | Not queried | 1.89 | 5.71e-02 | 0.2315877671 |
|  | 15 | 73735509 | 73735694 | PDFSTATSENSEMBLUCSC | 186 | 4 | 13412 | gene | 0.610617 | 0.7840795 | Not queried | 1.89 | 4.86e-01 | 0.5640816327 |
|  | 10 | 104211319 | 104211488 | PDFSTATSENSEMBLUCSC | 170 | 5 | 5325 | tss | 0.576781 | 0.4559739 | Not queried | 1.89 | 1.00e+00 | 1.0000000000 |
|  | 6 | 150463775 | 150464124 | PDFSTATSENSEMBLUCSC | 350 | 7 | 35296 | tss | 0.054533 | 0.0243810 | Not queried | 1.89 | 2.59e-01 | 0.4381244265 |
|  | 16 | 50099438 | 50099780 | PDFSTATSENSEMBLUCSC | 343 | 4 | 15228 | tss | 0.275058 | 0.0251630 | Not queried | 1.89 | 6.86e-01 | 0.7223604052 |
|  | 4 | 57547347 | 57547699 | PDFSTATSENSEMBLUCSC | 353 | 4 | 30011 | gene | 0.270131 | 0.3905145 | Not queried | 1.89 | 3.43e-01 | 0.4672865596 |
|  | 13 | 113642457 | 113642784 | PDFSTATSENSEMBLUCSC | 328 | 6 | 11265 | gene | 0.691641 | 0.9018924 | Not queried | 1.89 | 2.40e-01 | 0.4133265075 |
|  | 17 | 40932199 | 40932746 | PDFSTATSENSEMBLUCSC | 548 | 9 | 18353 | island | 0.054793 | 0.4925054 | Not queried | 1.89 | 2.58e-01 | 0.4381244265 |
|  | 8 | 62200380 | 62200518 | PDFSTATSENSEMBLUCSC | 139 | 4 | 39727 | tss | 0.037520 | 0.2694527 | Not queried | 1.89 | 6.86e-01 | 0.7223604052 |
|  | 7 | 121956502 | 121956890 | PDFSTATSENSEMBLUCSC | 389 | 5 | 38874 | island | 0.009693 | 0.0339019 | Not queried | 1.89 | 8.41e-01 | 0.8635565145 |
|  | 7 | 20838339 | 20838631 | PDFSTATSENSEMBLUCSC | 293 | 5 | 38296 | island | 0.173723 | 0.0133535 | Not queried | 1.89 | 3.10e-01 | 0.4672865596 |
|  | 22 | 23522307 | 23522748 | PDFSTATSENSEMBLUCSC | 442 | 9 | 27349 | island | 0.172053 | 0.0270550 | Not queried | 1.89 | 2.22e-01 | 0.3935758299 |
|  | 6 | 80657412 | 80657555 | PDFSTATSENSEMBLUCSC | 144 | 7 | 35083 | tss;island | 0.062462 | 0.0179229 | Not queried | 1.88 | 5.30e-02 | 0.2315877671 |
|  | 20 | 30062970 | 30063249 | PDFSTATSENSEMBLUCSC | 280 | 8 | 26071 | island | 0.068107 | 0.0207092 | Not queried | 1.88 | 8.30e-02 | 0.3028612233 |
|  | 4 | 187810446 | 187810783 | PDFSTATSENSEMBLUCSC | 338 | 5 | 31487 | island | 0.573393 | 0.9278655 | Not queried | 1.88 | 4.21e-01 | 0.5404252807 |
|  | 3 | 187387555 | 187388148 | PDFSTATSENSEMBLUCSC | 594 | 8 | 28077 | gene | 0.124969 | 0.4855629 | Not queried | 1.88 | 1.04e-02 | 0.1806165940 |
|  | 3 | 187387555 | 187388281 | PDFSTATSENSEMBLUCSC | 727 | 10 | 29759 | island | 0.116895 | 0.4303736 | Not queried | 1.88 | 1.85e-02 | 0.1830867741 |
|  | 5 | 134735544 | 134735701 | PDFSTATSENSEMBLUCSC | 158 | 7 | 32303 | tss;island | 0.532128 | 0.1718994 | Not queried | 1.88 | 1.11e-02 | 0.1821650572 |
|  | 19 | 7562249 | 7562555 | PDFSTATSENSEMBLUCSC | 307 | 4 | 21450 | island | 0.392704 | 0.2581591 | Not queried | 1.88 | 8.86e-01 | 0.8941250130 |
|  | 1 | 214557726 | 214557928 | PDFSTATSENSEMBLUCSC | 203 | 4 | 1007 | gene;island | 0.831901 | 0.9728915 | Not queried | 1.88 | 1.14e-01 | 0.3123457953 |
|  | 12 | 54343506 | 54343835 | PDFSTATSENSEMBLUCSC | 330 | 5 | 10480 | island | 0.024140 | 0.1336765 | Not queried | 1.88 | 2.22e-01 | 0.3935758299 |
|  | 6 | 152702330 | 152702660 | PDFSTATSENSEMBLUCSC | 331 | 6 | 34516 | gene | 0.578251 | 0.7498369 | Not queried | 1.88 | 2.40e-01 | 0.4133265075 |
|  | 8 | 145028093 | 145028505 | PDFSTATSENSEMBLUCSC | 413 | 9 | 39967 | tss | 0.075565 | 0.3091700 | Not queried | 1.88 | 4.89e-01 | 0.5677586445 |
|  | 6 | 28186797 | 28186923 | PDFSTATSENSEMBLUCSC | 127 | 6 | 34735 | tss | 0.016732 | 0.0505087 | Not queried | 1.88 | 9.31e-02 | 0.3104222821 |
|  | 8 | 53477881 | 53478102 | PDFSTATSENSEMBLUCSC | 222 | 5 | 40293 | island | 0.094092 | 0.0850991 | Not queried | 1.88 | 8.41e-01 | 0.8635565145 |
|  | 5 | 156696185 | 156696520 | PDFSTATSENSEMBLUCSC | 336 | 4 | 31874 | gene;island | 0.112000 | 0.1753423 | Not queried | 1.88 | 1.00e+00 | 1.0000000000 |
|  | 20 | 62037503 | 62037758 | PDFSTATSENSEMBLUCSC | 256 | 4 | 26352 | island | 0.497636 | 0.7951329 | Not queried | 1.88 | 4.86e-01 | 0.5640816327 |
|  | 20 | 9819697 | 9819820 | PDFSTATSENSEMBLUCSC | 124 | 6 | 25593 | tss;island | 0.024536 | 0.0128838 | Not queried | 1.88 | 4.85e-01 | 0.5640816327 |
|  | 10 | 8101307 | 8101566 | PDFSTATSENSEMBLUCSC | 260 | 4 | 4490 | gene;island | 0.918740 | 0.3210888 | Not queried | 1.88 | 3.43e-01 | 0.4672865596 |
|  | 17 | 744675 | 744939 | PDFSTATSENSEMBLUCSC | 265 | 5 | 16297 | gene;island | 0.882868 | 0.9599463 | Not queried | 1.88 | 3.17e-02 | 0.1866936846 |
|  | 10 | 38382849 | 38383250 | PDFSTATSENSEMBLUCSC | 402 | 9 | 5036 | tss | 0.010948 | 0.0041865 | Not queried | 1.88 | 2.58e-01 | 0.4381244265 |
|  | 5 | 2753746 | 2754322 | PDFSTATSENSEMBLUCSC | 577 | 10 | 31550 | gene;island | 0.012552 | 0.0374693 | Not queried | 1.88 | 1.90e-01 | 0.3826488233 |
|  | 8 | 116679763 | 116680127 | PDFSTATSENSEMBLUCSC | 365 | 4 | 39404 | gene;island | 0.086707 | 0.0107011 | Not queried | 1.88 | 2.00e-01 | 0.3826488233 |
|  | 1 | 99126913 | 99127446 | PDFSTATSENSEMBLUCSC | 534 | 6 | 3516 | island | 0.012565 | 0.0519440 | Not queried | 1.88 | 1.80e-01 | 0.3826488233 |
|  | 10 | 101292403 | 101292682 | PDFSTATSENSEMBLUCSC | 280 | 9 | 5282 | tss | 0.012992 | 0.0071613 | Not queried | 1.87 | 6.25e-02 | 0.2477578846 |
|  | 7 | 1095416 | 1095720 | PDFSTATSENSEMBLUCSC | 305 | 6 | 36742 | gene;island | 0.546825 | 0.4516949 | Not queried | 1.87 | 2.40e-01 | 0.4133265075 |
|  | 6 | 99276697 | 99277015 | PDFSTATSENSEMBLUCSC | 319 | 4 | 36341 | island | 0.251051 | 0.0063921 | Not queried | 1.87 | 4.86e-01 | 0.5640816327 |
|  | 1 | 16563727 | 16563954 | PDFSTATSENSEMBLUCSC | 228 | 5 | 1281 | tss | 0.002719 | 0.0040388 | Not queried | 1.87 | 6.90e-01 | 0.7223604052 |
|  | 1 | 236557473 | 236557758 | PDFSTATSENSEMBLUCSC | 286 | 4 | 2343 | tss;island | 0.016643 | 0.0521670 | Not queried | 1.87 | 2.00e-01 | 0.3826488233 |
|  | 8 | 23563925 | 23564294 | PDFSTATSENSEMBLUCSC | 370 | 8 | 39603 | tss | 0.319307 | 0.0744078 | Not queried | 1.87 | 4.99e-02 | 0.2315877671 |
|  | 2 | 232651086 | 232651190 | PDFSTATSENSEMBLUCSC | 105 | 7 | 25188 | island | 0.002624 | 0.0013041 | Not queried | 1.87 | 2.59e-01 | 0.4381244265 |
|  | 4 | 176922556 | 176923081 | PDFSTATSENSEMBLUCSC | 526 | 5 | 30185 | gene;island | 0.015106 | 0.0081440 | Not queried | 1.87 | 4.21e-01 | 0.5404252807 |
|  | 5 | 177659919 | 177660036 | PDFSTATSENSEMBLUCSC | 118 | 4 | 32539 | tss;island | 0.020673 | 0.0906450 | Not queried | 1.87 | 4.86e-01 | 0.5640816327 |
|  | 16 | 1384626 | 1384908 | PDFSTATSENSEMBLUCSC | 283 | 5 | 15529 | island | 0.234448 | 0.0723550 | Not queried | 1.87 | 5.48e-01 | 0.6179718485 |
|  | 8 | 19796299 | 19796561 | PDFSTATSENSEMBLUCSC | 263 | 5 | 39574 | tss | 0.190963 | 0.0303151 | Not queried | 1.87 | 2.22e-01 | 0.3935758299 |
|  | 14 | 85996101 | 85996393 | PDFSTATSENSEMBLUCSC | 293 | 8 | 12542 | tss | 0.057501 | 0.1967738 | Not queried | 1.87 | 2.79e-01 | 0.4672865596 |
|  | 22 | 17083412 | 17083727 | PDFSTATSENSEMBLUCSC | 316 | 4 | 26778 | gene;island | 0.042168 | 0.1337405 | Not queried | 1.87 | 1.14e-01 | 0.3123457953 |
|  | 17 | 72462164 | 72462441 | PDFSTATSENSEMBLUCSC | 278 | 4 | 17627 | tss | 0.026344 | 0.1427795 | Not queried | 1.87 | 6.86e-01 | 0.7223604052 |
|  | 13 | 114793125 | 114793396 | PDFSTATSENSEMBLUCSC | 272 | 4 | 11308 | gene | 0.862385 | 0.5574913 | Not queried | 1.87 | 2.00e-01 | 0.3826488233 |
|  | 4 | 148402399 | 148402899 | PDFSTATSENSEMBLUCSC | 501 | 5 | 30150 | gene;island | 0.090257 | 0.2785738 | Not queried | 1.87 | 2.22e-01 | 0.3935758299 |
|  | 20 | 31870775 | 31870935 | PDFSTATSENSEMBLUCSC | 161 | 4 | 25681 | tss | 0.137513 | 0.0388358 | Not queried | 1.87 | 8.86e-01 | 0.8941250130 |
|  | 5 | 5287980 | 5288296 | PDFSTATSENSEMBLUCSC | 317 | 6 | 31552 | gene | 0.745754 | 0.7781827 | Not queried | 1.87 | 4.85e-01 | 0.5640816327 |
|  | 1 | 206680459 | 206680868 | PDFSTATSENSEMBLUCSC | 410 | 5 | 2212 | tss | 0.006145 | 0.0208515 | Not queried | 1.86 | 4.21e-01 | 0.5404252807 |
|  | 17 | 39822429 | 39822735 | PDFSTATSENSEMBLUCSC | 307 | 4 | 18312 | island | 0.003539 | 0.0023870 | Not queried | 1.86 | 2.00e-01 | 0.3826488233 |
|  | 10 | 97889373 | 97889448 | PDFSTATSENSEMBLUCSC | 76 | 4 | 5253 | tss | 0.486244 | 0.9451948 | Not queried | 1.86 | 3.43e-01 | 0.4672865596 |
|  | 6 | 33215649 | 33215868 | PDFSTATSENSEMBLUCSC | 220 | 6 | 35986 | island | 0.055725 | 0.1468743 | Not queried | 1.86 | 5.89e-01 | 0.6570484697 |
|  | 12 | 52627272 | 52627576 | PDFSTATSENSEMBLUCSC | 305 | 4 | 9135 | gene;island | 0.086764 | 0.3699897 | Not queried | 1.86 | 4.86e-01 | 0.5640816327 |
|  | 11 | 84634581 | 84634735 | PDFSTATSENSEMBLUCSC | 155 | 4 | 7601 | tss | 0.659709 | 0.8904612 | Not queried | 1.86 | 3.43e-01 | 0.4672865596 |
|  | 6 | 1598854 | 1599401 | PDFSTATSENSEMBLUCSC | 548 | 6 | 35370 | island | 0.493415 | 0.8987920 | Not queried | 1.86 | 1.32e-01 | 0.3397986247 |
|  | 14 | 77737495 | 77737800 | PDFSTATSENSEMBLUCSC | 306 | 6 | 13032 | island | 0.361521 | 0.0550492 | Not queried | 1.86 | 3.10e-01 | 0.4672865596 |
|  | 12 | 121416315 | 121416547 | PDFSTATSENSEMBLUCSC | 233 | 6 | 10069 | tss | 0.662103 | 0.8974876 | Not queried | 1.86 | 4.11e-02 | 0.2180389943 |
|  | 6 | 170101455 | 170101549 | PDFSTATSENSEMBLUCSC | 95 | 4 | 34574 | gene;tss;island | 0.010777 | 0.0210551 | Not queried | 1.86 | 5.71e-02 | 0.2315877671 |
|  | 1 | 45792557 | 45792733 | PDFSTATSENSEMBLUCSC | 177 | 5 | 501 | gene | 0.016632 | 0.0053940 | Not queried | 1.86 | 3.17e-02 | 0.1866936846 |
|  | 1 | 45792540 | 45792733 | PDFSTATSENSEMBLUCSC | 194 | 7 | 3198 | island | 0.016632 | 0.0053940 | Not queried | 1.86 | 7.28e-02 | 0.2713481487 |
|  | 20 | 42543034 | 42543446 | PDFSTATSENSEMBLUCSC | 413 | 7 | 25752 | tss;island | 0.032186 | 0.0195695 | Not queried | 1.86 | 2.59e-01 | 0.4381244265 |
|  | 8 | 49231559 | 49231786 | PDFSTATSENSEMBLUCSC | 228 | 4 | 40280 | island | 0.015225 | 0.0496185 | Not queried | 1.86 | 3.43e-01 | 0.4672865596 |
|  | 19 | 13215041 | 13215387 | PDFSTATSENSEMBLUCSC | 347 | 5 | 21644 | island | 0.027408 | 0.0702646 | Not queried | 1.86 | 7.94e-03 | 0.1589602024 |
|  | 4 | 2795105 | 2795501 | PDFSTATSENSEMBLUCSC | 397 | 6 | 29893 | gene;island | 0.007113 | 0.0220613 | Not queried | 1.86 | 6.99e-01 | 0.7290267580 |
|  | 12 | 132905611 | 132906010 | PDFSTATSENSEMBLUCSC | 400 | 4 | 11042 | island | 0.062144 | 0.0387345 | Not queried | 1.86 | 2.00e-01 | 0.3826488233 |
|  | 1 | 186649559 | 186649985 | PDFSTATSENSEMBLUCSC | 427 | 7 | 2146 | tss | 0.021140 | 0.0109766 | Not queried | 1.86 | 1.65e-01 | 0.3826488233 |
|  | 8 | 142452378 | 142452509 | PDFSTATSENSEMBLUCSC | 132 | 4 | 39454 | gene;island | 0.718241 | 0.9007904 | Not queried | 1.86 | 3.43e-01 | 0.4672865596 |
|  | 5 | 34838922 | 34839352 | PDFSTATSENSEMBLUCSC | 431 | 9 | 32726 | island | 0.019203 | 0.0088598 | Not queried | 1.86 | 1.90e-01 | 0.3826488233 |
|  | 17 | 73642404 | 73642628 | PDFSTATSENSEMBLUCSC | 225 | 6 | 17658 | tss | 0.064437 | 0.0204908 | Not queried | 1.86 | 4.85e-01 | 0.5640816327 |
|  | 10 | 71892593 | 71892787 | PDFSTATSENSEMBLUCSC | 195 | 7 | 5812 | island | 0.004685 | 0.0270801 | Not queried | 1.86 | 2.09e-01 | 0.3935758299 |
|  | 17 | 74864041 | 74864302 | PDFSTATSENSEMBLUCSC | 262 | 4 | 17690 | tss;island | 0.112547 | 0.0719962 | Not queried | 1.86 | 6.86e-01 | 0.7223604052 |
|  | 10 | 134755601 | 134755955 | PDFSTATSENSEMBLUCSC | 355 | 4 | 4883 | gene;island | 0.047796 | 0.0604170 | Not queried | 1.86 | 8.86e-01 | 0.8941250130 |
|  | 6 | 33757040 | 33757077 | PDFSTATSENSEMBLUCSC | 38 | 4 | 34913 | tss | 0.006579 | 0.0041303 | Not queried | 1.86 | 2.86e-02 | 0.1830867741 |
|  | 3 | 184529753 | 184529876 | PDFSTATSENSEMBLUCSC | 124 | 5 | 28764 | tss | 0.002187 | 0.0070356 | Not queried | 1.86 | 3.10e-01 | 0.4672865596 |
|  | 10 | 105678128 | 105678266 | PDFSTATSENSEMBLUCSC | 139 | 6 | 5341 | tss | 0.002953 | 0.0052784 | Not queried | 1.86 | 4.11e-02 | 0.2180389943 |
|  | 10 | 75457622 | 75457686 | PDFSTATSENSEMBLUCSC | 65 | 4 | 5161 | tss | 0.855820 | 0.9535250 | Not queried | 1.86 | 2.86e-02 | 0.1830867741 |
|  | 2 | 97202260 | 97202435 | PDFSTATSENSEMBLUCSC | 176 | 4 | 23519 | tss | 0.001626 | 0.0060287 | Not queried | 1.86 | 3.43e-01 | 0.4672865596 |
|  | 17 | 17109651 | 17110353 | PDFSTATSENSEMBLUCSC | 703 | 12 | 17162 | tss | 0.379419 | 0.6042759 | Not queried | 1.86 | 4.43e-01 | 0.5640816327 |
|  | 4 | 122686269 | 122686667 | PDFSTATSENSEMBLUCSC | 399 | 9 | 30114 | gene;island | 0.015380 | 0.0726511 | Not queried | 1.86 | 3.15e-02 | 0.1866936846 |
|  | 13 | 51640297 | 51640442 | PDFSTATSENSEMBLUCSC | 146 | 4 | 11450 | tss | 0.019940 | 0.0855513 | Not queried | 1.86 | 3.43e-01 | 0.4672865596 |
|  | 10 | 28034669 | 28034856 | PDFSTATSENSEMBLUCSC | 188 | 7 | 5634 | island | 0.109395 | 0.3444235 | Not queried | 1.86 | 9.73e-02 | 0.3123457953 |
|  | 17 | 66508512 | 66508605 | PDFSTATSENSEMBLUCSC | 94 | 4 | 16742 | gene;island | 0.002398 | 0.0007576 | Not queried | 1.86 | 5.71e-02 | 0.2315877671 |
|  | 1 | 2046506 | 2046928 | PDFSTATSENSEMBLUCSC | 423 | 5 | 61 | gene;island | 0.621083 | 0.7438763 | Not queried | 1.86 | 8.41e-01 | 0.8635565145 |
|  | 1 | 109941043 | 109941201 | PDFSTATSENSEMBLUCSC | 159 | 5 | 1802 | tss;island | 0.523749 | 0.0596369 | Not queried | 1.86 | 2.22e-01 | 0.3935758299 |
|  | 8 | 42751637 | 42751762 | PDFSTATSENSEMBLUCSC | 126 | 4 | 39299 | gene;tss;island | 0.010777 | 0.0083481 | Not queried | 1.85 | 4.86e-01 | 0.5640816327 |
|  | 5 | 140529499 | 140529819 | PDFSTATSENSEMBLUCSC | 321 | 6 | 32363 | tss;island | 0.086443 | 0.1504914 | Not queried | 1.85 | 8.18e-01 | 0.8469867211 |
|  | 11 | 98891492 | 98891820 | PDFSTATSENSEMBLUCSC | 329 | 9 | 7640 | tss;island | 0.011347 | 0.1185692 | Not queried | 1.85 | 1.42e-02 | 0.1830867741 |
|  | 7 | 150216872 | 150217081 | PDFSTATSENSEMBLUCSC | 210 | 4 | 37275 | gene | 0.500586 | 0.8722164 | Not queried | 1.85 | 3.43e-01 | 0.4672865596 |
|  | 14 | 24803679 | 24804342 | PDFSTATSENSEMBLUCSC | 664 | 10 | 12746 | island | 0.019944 | 0.0923914 | Not queried | 1.85 | 1.15e-02 | 0.1830867741 |
|  | 14 | 24803873 | 24804342 | PDFSTATSENSEMBLUCSC | 470 | 8 | 12328 | tss | 0.019944 | 0.1006235 | Not queried | 1.85 | 6.50e-02 | 0.2477578846 |
|  | 15 | 45422325 | 45422553 | PDFSTATSENSEMBLUCSC | 229 | 4 | 13328 | gene;tss;island | 0.444123 | 0.6630927 | Not queried | 1.85 | 3.43e-01 | 0.4672865596 |
|  | 15 | 43398302 | 43398581 | PDFSTATSENSEMBLUCSC | 280 | 6 | 13622 | tss | 0.006909 | 0.0024242 | Not queried | 1.85 | 3.10e-01 | 0.4672865596 |
|  | 12 | 132976722 | 132977140 | PDFSTATSENSEMBLUCSC | 419 | 4 | 11049 | island | 0.239597 | 0.0575342 | Not queried | 1.85 | 2.00e-01 | 0.3826488233 |
|  | 11 | 43596920 | 43597273 | PDFSTATSENSEMBLUCSC | 354 | 5 | 8214 | island | 0.014357 | 0.0460242 | Not queried | 1.85 | 3.17e-02 | 0.1866936846 |
|  | 1 | 228291486 | 228291705 | PDFSTATSENSEMBLUCSC | 220 | 5 | 2297 | tss;island | 0.981224 | 0.8149215 | Not queried | 1.85 | 2.22e-01 | 0.3935758299 |
|  | 18 | 19476805 | 19477059 | PDFSTATSENSEMBLUCSC | 255 | 5 | 19420 | island | 0.513000 | 0.6348109 | Not queried | 1.85 | 4.21e-01 | 0.5404252807 |
|  | 16 | 79633452 | 79633901 | PDFSTATSENSEMBLUCSC | 450 | 4 | 14851 | gene;island | 0.248118 | 0.0995400 | Not queried | 1.85 | 3.43e-01 | 0.4672865596 |
|  | 19 | 663355 | 663602 | PDFSTATSENSEMBLUCSC | 248 | 5 | 20277 | tss;island | 0.004359 | 0.0106079 | Not queried | 1.85 | 4.21e-01 | 0.5404252807 |
|  | 2 | 207506975 | 207507096 | PDFSTATSENSEMBLUCSC | 122 | 4 | 23838 | tss | 0.096460 | 0.2328940 | Not queried | 1.85 | 3.43e-01 | 0.4672865596 |
|  | 11 | 128556611 | 128556992 | PDFSTATSENSEMBLUCSC | 382 | 4 | 8939 | island | 0.039086 | 0.0155537 | Not queried | 1.85 | 4.86e-01 | 0.5640816327 |
|  | 16 | 88575243 | 88575557 | PDFSTATSENSEMBLUCSC | 315 | 4 | 14907 | gene;island | 0.728737 | 0.9345652 | Not queried | 1.85 | 3.43e-01 | 0.4672865596 |
|  | 10 | 38382849 | 38383525 | PDFSTATSENSEMBLUCSC | 677 | 11 | 5673 | island | 0.017106 | 0.0065924 | Not queried | 1.85 | 3.32e-01 | 0.4672865596 |
|  | 17 | 4648949 | 4649333 | PDFSTATSENSEMBLUCSC | 385 | 4 | 16358 | gene | 0.513959 | 0.5084802 | Not queried | 1.85 | 6.86e-01 | 0.7223604052 |
|  | 6 | 29942951 | 29943841 | PDFSTATSENSEMBLUCSC | 891 | 15 | 33703 | gene;island | 0.125717 | 0.0123264 | Not queried | 1.85 | 9.87e-03 | 0.1806165940 |
|  | 12 | 57410518 | 57410788 | PDFSTATSENSEMBLUCSC | 271 | 4 | 9806 | tss | 0.436808 | 0.7309983 | Not queried | 1.85 | 2.00e-01 | 0.3826488233 |
|  | 1 | 246670726 | 246670940 | PDFSTATSENSEMBLUCSC | 215 | 7 | 2362 | tss;island | 0.010834 | 0.0021820 | Not queried | 1.85 | 9.02e-01 | 0.9091904204 |
|  | 11 | 79148183 | 79148548 | PDFSTATSENSEMBLUCSC | 366 | 4 | 6849 | gene;island | 0.041789 | 0.1015135 | Not queried | 1.85 | 4.86e-01 | 0.5640816327 |
|  | 1 | 47695138 | 47695475 | PDFSTATSENSEMBLUCSC | 338 | 4 | 3230 | island | 0.059152 | 0.0566000 | Not queried | 1.85 | 1.00e+00 | 1.0000000000 |
|  | 6 | 170562191 | 170562720 | PDFSTATSENSEMBLUCSC | 530 | 4 | 36683 | island | 0.463732 | 0.7143391 | Not queried | 1.85 | 2.00e-01 | 0.3826488233 |
|  | 17 | 79393463 | 79393718 | PDFSTATSENSEMBLUCSC | 256 | 5 | 16908 | gene;island | 0.503865 | 0.7851212 | Not queried | 1.85 | 1.51e-01 | 0.3618000441 |
|  | 7 | 157320088 | 157320662 | PDFSTATSENSEMBLUCSC | 575 | 7 | 39106 | island | 0.218449 | 0.4338374 | Not queried | 1.85 | 2.59e-01 | 0.4381244265 |
|  | 5 | 76939333 | 76939558 | PDFSTATSENSEMBLUCSC | 226 | 4 | 32904 | island | 0.032823 | 0.1096047 | Not queried | 1.85 | 2.86e-02 | 0.1830867741 |
|  | 19 | 7911120 | 7911362 | PDFSTATSENSEMBLUCSC | 243 | 5 | 20409 | tss | 0.403862 | 0.5895586 | Not queried | 1.85 | 3.10e-01 | 0.4672865596 |
|  | 19 | 41106155 | 41106351 | PDFSTATSENSEMBLUCSC | 197 | 4 | 20046 | gene;tss;island | 0.008111 | 0.0120234 | Not queried | 1.85 | 4.86e-01 | 0.5640816327 |
|  | 19 | 42891265 | 42891428 | PDFSTATSENSEMBLUCSC | 164 | 4 | 20071 | gene;island | 0.025582 | 0.1804435 | Not queried | 1.85 | 3.43e-01 | 0.4672865596 |
|  | 21 | 15645649 | 15645988 | PDFSTATSENSEMBLUCSC | 340 | 4 | 26487 | tss | 0.368855 | 0.7494067 | Not queried | 1.85 | 2.00e-01 | 0.3826488233 |
|  | 16 | 86411222 | 86411456 | PDFSTATSENSEMBLUCSC | 235 | 5 | 16169 | island | 0.427156 | 0.6985670 | Not queried | 1.84 | 5.48e-01 | 0.6179718485 |
|  | 7 | 2774414 | 2774654 | PDFSTATSENSEMBLUCSC | 241 | 4 | 36815 | gene;island | 0.236782 | 0.5207650 | Not queried | 1.84 | 5.71e-02 | 0.2315877671 |
|  | 11 | 72982929 | 72983097 | PDFSTATSENSEMBLUCSC | 169 | 4 | 6820 | gene;tss | 0.888310 | 0.4418872 | Not queried | 1.84 | 4.86e-01 | 0.5640816327 |
|  | 16 | 85171054 | 85171295 | PDFSTATSENSEMBLUCSC | 242 | 4 | 16150 | island | 0.019453 | 0.1229229 | Not queried | 1.84 | 3.43e-01 | 0.4672865596 |
|  | 17 | 79868964 | 79869136 | PDFSTATSENSEMBLUCSC | 173 | 4 | 16927 | gene;island | 0.006008 | 0.0043972 | Not queried | 1.84 | 8.86e-01 | 0.8941250130 |
|  | 14 | 70233599 | 70233918 | PDFSTATSENSEMBLUCSC | 320 | 10 | 12126 | gene;island | 0.003792 | 0.0084055 | Not queried | 1.84 | 6.30e-02 | 0.2477578846 |
|  | 6 | 33140660 | 33141305 | PDFSTATSENSEMBLUCSC | 646 | 9 | 34201 | gene | 0.021133 | 0.3734163 | Not queried | 1.84 | 1.13e-01 | 0.3123457953 |
|  | 1 | 47184735 | 47185236 | PDFSTATSENSEMBLUCSC | 502 | 6 | 3227 | island | 0.002800 | 0.0084091 | Not queried | 1.84 | 1.80e-01 | 0.3826488233 |
|  | 19 | 10735482 | 10735594 | PDFSTATSENSEMBLUCSC | 113 | 4 | 19817 | gene;tss;island | 0.920014 | 0.6214295 | Not queried | 1.84 | 4.86e-01 | 0.5640816327 |
|  | 15 | 53075421 | 53075877 | PDFSTATSENSEMBLUCSC | 457 | 6 | 13357 | gene;island | 0.326869 | 0.0422130 | Not queried | 1.84 | 4.85e-01 | 0.5640816327 |
|  | 10 | 124907409 | 124907600 | PDFSTATSENSEMBLUCSC | 192 | 5 | 5417 | tss | 0.094004 | 0.0099539 | Not queried | 1.84 | 3.17e-02 | 0.1866936846 |
|  | 8 | 41518004 | 41518139 | PDFSTATSENSEMBLUCSC | 136 | 4 | 39289 | gene | 0.799872 | 0.8989271 | Not queried | 1.84 | 3.43e-01 | 0.4672865596 |
|  | 6 | 167276275 | 167276650 | PDFSTATSENSEMBLUCSC | 376 | 5 | 35339 | tss;island | 0.036784 | 0.0179026 | Not queried | 1.84 | 4.21e-01 | 0.5404252807 |
|  | 10 | 72201484 | 72201741 | PDFSTATSENSEMBLUCSC | 258 | 6 | 5135 | tss | 0.287560 | 0.2084869 | Not queried | 1.84 | 4.85e-01 | 0.5640816327 |
|  | 17 | 42091713 | 42091909 | PDFSTATSENSEMBLUCSC | 197 | 4 | 16603 | gene;island | 0.237335 | 0.0087139 | Not queried | 1.84 | 1.14e-01 | 0.3123457953 |
|  | 19 | 57352014 | 57352074 | PDFSTATSENSEMBLUCSC | 61 | 4 | 20242 | gene | 0.952955 | 0.7325450 | Not queried | 1.84 | 3.43e-01 | 0.4672865596 |
|  | 7 | 157333405 | 157333949 | PDFSTATSENSEMBLUCSC | 545 | 5 | 37324 | gene | 0.600708 | 0.8835025 | Not queried | 1.84 | 1.51e-01 | 0.3618000441 |
|  | 6 | 27101633 | 27101941 | PDFSTATSENSEMBLUCSC | 309 | 4 | 34712 | tss;island | 0.004137 | 0.0033723 | Not queried | 1.84 | 6.86e-01 | 0.7223604052 |
|  | 6 | 99278991 | 99279108 | PDFSTATSENSEMBLUCSC | 118 | 4 | 36342 | island | 0.081619 | 0.2129319 | Not queried | 1.84 | 4.86e-01 | 0.5640816327 |
|  | 8 | 42911287 | 42911370 | PDFSTATSENSEMBLUCSC | 84 | 5 | 39684 | tss | 0.000901 | 0.0032045 | Not queried | 1.83 | 9.52e-02 | 0.3104222821 |
|  | 6 | 26458189 | 26458489 | PDFSTATSENSEMBLUCSC | 301 | 4 | 33612 | gene | 0.010731 | 0.0060300 | Not queried | 1.83 | 6.86e-01 | 0.7223604052 |
|  | 5 | 115297690 | 115298231 | PDFSTATSENSEMBLUCSC | 542 | 6 | 33029 | island | 0.382904 | 0.0580617 | Not queried | 1.83 | 3.10e-01 | 0.4672865596 |
|  | 14 | 102554826 | 102554977 | PDFSTATSENSEMBLUCSC | 152 | 4 | 12226 | gene;tss;island | 0.074109 | 0.2476712 | Not queried | 1.83 | 3.43e-01 | 0.4672865596 |
|  | 3 | 150690183 | 150690585 | PDFSTATSENSEMBLUCSC | 403 | 7 | 27996 | gene | 0.497193 | 0.8496373 | Not queried | 1.83 | 3.18e-01 | 0.4672865596 |
|  | 17 | 41832753 | 41833324 | PDFSTATSENSEMBLUCSC | 572 | 6 | 16598 | gene;island | 0.083519 | 0.1316803 | Not queried | 1.83 | 4.85e-01 | 0.5640816327 |
|  | 6 | 99283553 | 99284027 | PDFSTATSENSEMBLUCSC | 475 | 4 | 34420 | gene;island | 0.118845 | 0.1668224 | Not queried | 1.83 | 4.86e-01 | 0.5640816327 |
|  | 13 | 111522755 | 111522985 | PDFSTATSENSEMBLUCSC | 231 | 5 | 11540 | tss | 0.645199 | 0.9104279 | Not queried | 1.83 | 9.52e-02 | 0.3104222821 |
|  | 7 | 8473234 | 8473578 | PDFSTATSENSEMBLUCSC | 345 | 6 | 37439 | tss;island | 0.027300 | 0.0896942 | Not queried | 1.83 | 1.32e-01 | 0.3397986247 |
|  | 11 | 50257496 | 50257979 | PDFSTATSENSEMBLUCSC | 484 | 7 | 8279 | island | 0.664115 | 0.8059195 | Not queried | 1.83 | 1.28e-01 | 0.3397986247 |
|  | 11 | 50257633 | 50257979 | PDFSTATSENSEMBLUCSC | 347 | 5 | 7296 | tss | 0.614782 | 0.7275173 | Not queried | 1.83 | 1.51e-01 | 0.3618000441 |
|  | 11 | 2593857 | 2594153 | PDFSTATSENSEMBLUCSC | 297 | 4 | 6463 | gene;island | 0.324374 | 0.4709794 | Not queried | 1.83 | 4.86e-01 | 0.5640816327 |
|  | 5 | 153863417 | 153863826 | PDFSTATSENSEMBLUCSC | 410 | 4 | 33333 | island | 0.197720 | 0.0629747 | Not queried | 1.82 | 8.86e-01 | 0.8941250130 |
|  | 17 | 75283800 | 75283979 | PDFSTATSENSEMBLUCSC | 180 | 4 | 16803 | gene | 0.648650 | 0.8778721 | Not queried | 1.82 | 2.86e-02 | 0.1830867741 |
|  | 11 | 89867637 | 89867809 | PDFSTATSENSEMBLUCSC | 173 | 5 | 7615 | tss | 0.011756 | 0.0035716 | Not queried | 1.82 | 2.22e-01 | 0.3935758299 |
|  | 7 | 63505584 | 63505811 | PDFSTATSENSEMBLUCSC | 228 | 6 | 37613 | tss | 0.069717 | 0.2963209 | Not queried | 1.82 | 9.31e-02 | 0.3104222821 |
|  | 1 | 202927535 | 202927977 | PDFSTATSENSEMBLUCSC | 443 | 5 | 2186 | tss | 0.019869 | 0.0232619 | Not queried | 1.82 | 4.21e-01 | 0.5404252807 |
|  | 15 | 89910632 | 89910860 | PDFSTATSENSEMBLUCSC | 229 | 4 | 13886 | tss;island | 0.104032 | 0.3103732 | Not queried | 1.82 | 2.00e-01 | 0.3826488233 |
|  | 2 | 210636350 | 210636844 | PDFSTATSENSEMBLUCSC | 495 | 8 | 25053 | island | 0.107441 | 0.0227075 | Not queried | 1.82 | 3.79e-02 | 0.2121545571 |
|  | 3 | 49907414 | 49907692 | PDFSTATSENSEMBLUCSC | 279 | 6 | 28338 | tss | 0.034503 | 0.1669478 | Not queried | 1.82 | 4.85e-01 | 0.5640816327 |
|  | 8 | 144965352 | 144965577 | PDFSTATSENSEMBLUCSC | 226 | 4 | 40696 | island | 0.130054 | 0.3492451 | Not queried | 1.82 | 2.00e-01 | 0.3826488233 |
|  | 12 | 12965884 | 12966464 | PDFSTATSENSEMBLUCSC | 581 | 9 | 10256 | island | 0.005232 | 0.0024193 | Not queried | 1.82 | 4.36e-01 | 0.5589686471 |
|  | 19 | 20162642 | 20163138 | PDFSTATSENSEMBLUCSC | 497 | 7 | 21833 | island | 0.017399 | 0.0054683 | Not queried | 1.82 | 4.56e-01 | 0.5640816327 |
|  | 20 | 2361431 | 2361548 | PDFSTATSENSEMBLUCSC | 118 | 5 | 25543 | tss | 0.465697 | 0.7532331 | Not queried | 1.82 | 1.51e-01 | 0.3618000441 |
|  | 10 | 88024529 | 88024673 | PDFSTATSENSEMBLUCSC | 145 | 7 | 4659 | gene;island | 0.139245 | 0.3890921 | Not queried | 1.82 | 2.09e-01 | 0.3935758299 |
|  | 22 | 32599511 | 32599648 | PDFSTATSENSEMBLUCSC | 138 | 5 | 26846 | gene;tss | 0.537350 | 0.5458295 | Not queried | 1.82 | 6.90e-01 | 0.7223604052 |
|  | 6 | 30883762 | 30884124 | PDFSTATSENSEMBLUCSC | 363 | 4 | 33839 | gene;island | 0.673190 | 0.9228009 | Not queried | 1.82 | 1.14e-01 | 0.3123457953 |
|  | 17 | 79412583 | 79412788 | PDFSTATSENSEMBLUCSC | 206 | 4 | 16910 | gene;island | 0.535508 | 0.7217751 | Not queried | 1.82 | 4.86e-01 | 0.5640816327 |
|  | 17 | 70120182 | 70120541 | PDFSTATSENSEMBLUCSC | 360 | 4 | 16744 | gene;island | 0.670321 | 0.8574427 | Not queried | 1.82 | 1.14e-01 | 0.3123457953 |
|  | 10 | 98946673 | 98946943 | PDFSTATSENSEMBLUCSC | 271 | 4 | 5258 | tss;island | 0.499068 | 0.7029724 | Not queried | 1.82 | 4.86e-01 | 0.5640816327 |
|  | 7 | 2565944 | 2566263 | PDFSTATSENSEMBLUCSC | 320 | 5 | 36805 | gene;island | 0.819087 | 0.9591462 | Not queried | 1.82 | 3.17e-02 | 0.1866936846 |
|  | 11 | 133938676 | 133939064 | PDFSTATSENSEMBLUCSC | 389 | 6 | 8974 | island | 0.025519 | 0.0133647 | Not queried | 1.82 | 2.40e-01 | 0.4133265075 |
|  | 6 | 32632106 | 32632338 | PDFSTATSENSEMBLUCSC | 233 | 6 | 34125 | gene;island | 0.238172 | 0.4229016 | Not queried | 1.82 | 2.40e-01 | 0.4133265075 |
|  | 11 | 57232331 | 57232742 | PDFSTATSENSEMBLUCSC | 412 | 5 | 6613 | gene;island | 0.805244 | 0.5297770 | Not queried | 1.82 | 3.10e-01 | 0.4672865596 |
|  | 19 | 2427708 | 2428122 | PDFSTATSENSEMBLUCSC | 415 | 5 | 21284 | island | 0.006919 | 0.0075437 | Not queried | 1.82 | 5.48e-01 | 0.6179718485 |
|  | 17 | 48072565 | 48072833 | PDFSTATSENSEMBLUCSC | 269 | 4 | 18537 | island | 0.010590 | 0.0540234 | Not queried | 1.82 | 3.43e-01 | 0.4672865596 |
|  | 20 | 56803778 | 56804327 | PDFSTATSENSEMBLUCSC | 550 | 4 | 26287 | island | 0.048081 | 0.0946797 | Not queried | 1.82 | 4.86e-01 | 0.5640816327 |
|  | 15 | 25434030 | 25434533 | PDFSTATSENSEMBLUCSC | 504 | 7 | 13531 | tss | 0.271933 | 0.3835864 | Not queried | 1.81 | 3.18e-01 | 0.4672865596 |
|  | 1 | 14027378 | 14027581 | PDFSTATSENSEMBLUCSC | 204 | 4 | 236 | gene;island | 0.014714 | 0.0119940 | Not queried | 1.81 | 6.86e-01 | 0.7223604052 |
|  | 17 | 79632335 | 79632662 | PDFSTATSENSEMBLUCSC | 328 | 4 | 17744 | tss | 0.924178 | 0.5626375 | Not queried | 1.81 | 4.86e-01 | 0.5640816327 |
|  | 20 | 30406997 | 30407145 | PDFSTATSENSEMBLUCSC | 149 | 5 | 25667 | tss | 0.508836 | 0.8150419 | Not queried | 1.81 | 2.22e-01 | 0.3935758299 |
|  | 17 | 75451779 | 75452100 | PDFSTATSENSEMBLUCSC | 322 | 5 | 16810 | gene | 0.658531 | 0.9279821 | Not queried | 1.81 | 5.48e-01 | 0.6179718485 |
|  | 16 | 3989076 | 3989432 | PDFSTATSENSEMBLUCSC | 357 | 4 | 15657 | island | 0.074236 | 0.0077266 | Not queried | 1.81 | 8.86e-01 | 0.8941250130 |
|  | 15 | 99250795 | 99251007 | PDFSTATSENSEMBLUCSC | 213 | 4 | 13496 | gene;island | 0.608671 | 0.8591555 | Not queried | 1.81 | 2.00e-01 | 0.3826488233 |
|  | 13 | 31773806 | 31773954 | PDFSTATSENSEMBLUCSC | 149 | 4 | 11379 | tss;island | 0.019906 | 0.3699127 | Not queried | 1.81 | 3.43e-01 | 0.4672865596 |
|  | 11 | 64067685 | 64067868 | PDFSTATSENSEMBLUCSC | 184 | 7 | 8403 | island | 0.020195 | 0.0838877 | Not queried | 1.81 | 1.28e-01 | 0.3397986247 |
|  | 16 | 88815867 | 88816022 | PDFSTATSENSEMBLUCSC | 156 | 4 | 14916 | gene;island | 0.772644 | 0.9643065 | Not queried | 1.81 | 6.86e-01 | 0.7223604052 |
|  | 6 | 42145851 | 42146143 | PDFSTATSENSEMBLUCSC | 293 | 4 | 34338 | gene;island | 0.420015 | 0.0662211 | Not queried | 1.81 | 4.86e-01 | 0.5640816327 |
|  | 11 | 2183708 | 2183864 | PDFSTATSENSEMBLUCSC | 157 | 4 | 7062 | tss;island | 0.551106 | 0.8090281 | Not queried | 1.81 | 4.86e-01 | 0.5640816327 |
|  | 7 | 140179446 | 140179608 | PDFSTATSENSEMBLUCSC | 163 | 4 | 37938 | tss;island | 0.022343 | 0.0055727 | Not queried | 1.81 | 3.43e-01 | 0.4672865596 |
|  | 18 | 10414355 | 10414556 | PDFSTATSENSEMBLUCSC | 202 | 4 | 19389 | island | 0.700827 | 0.9419741 | Not queried | 1.81 | 3.43e-01 | 0.4672865596 |
|  | 16 | 875881 | 876216 | PDFSTATSENSEMBLUCSC | 336 | 4 | 15492 | island | 0.567528 | 0.8890019 | Not queried | 1.81 | 3.43e-01 | 0.4672865596 |
|  | 1 | 16563580 | 16563954 | PDFSTATSENSEMBLUCSC | 375 | 6 | 2741 | island | 0.002182 | 0.0039208 | Not queried | 1.81 | 5.89e-01 | 0.6570484697 |
|  | 8 | 70983567 | 70983760 | PDFSTATSENSEMBLUCSC | 194 | 4 | 39757 | tss | 0.216746 | 0.0728431 | Not queried | 1.81 | 2.00e-01 | 0.3826488233 |
|  | 11 | 126873139 | 126873689 | PDFSTATSENSEMBLUCSC | 551 | 6 | 8937 | island | 0.047846 | 0.2205410 | Not queried | 1.81 | 6.49e-02 | 0.2477578846 |
|  | 11 | 32458656 | 32459057 | PDFSTATSENSEMBLUCSC | 402 | 5 | 6568 | gene;island | 0.125520 | 0.0461392 | Not queried | 1.81 | 1.00e+00 | 1.0000000000 |
|  | 6 | 30112423 | 30112776 | PDFSTATSENSEMBLUCSC | 354 | 5 | 33727 | gene | 0.552994 | 0.6623090 | Not queried | 1.81 | 4.21e-01 | 0.5404252807 |
|  | 13 | 20735236 | 20735532 | PDFSTATSENSEMBLUCSC | 297 | 7 | 11327 | tss | 0.004664 | 0.0018097 | Not queried | 1.81 | 9.73e-02 | 0.3123457953 |
|  | 19 | 57337294 | 57337648 | PDFSTATSENSEMBLUCSC | 355 | 4 | 20240 | gene;tss | 0.058603 | 0.3060058 | Not queried | 1.81 | 6.86e-01 | 0.7223604052 |
|  | 12 | 113917395 | 113917737 | PDFSTATSENSEMBLUCSC | 343 | 5 | 10835 | island | 0.029910 | 0.0164595 | Not queried | 1.81 | 8.41e-01 | 0.8635565145 |
|  | 3 | 9811714 | 9811990 | PDFSTATSENSEMBLUCSC | 277 | 7 | 28130 | tss;island | 0.445821 | 0.0291129 | Not queried | 1.81 | 6.99e-03 | 0.1589602024 |
|  | 4 | 187422005 | 187422119 | PDFSTATSENSEMBLUCSC | 115 | 5 | 31481 | island | 0.781438 | 0.8954278 | Not queried | 1.81 | 3.10e-01 | 0.4672865596 |
|  | 15 | 23932370 | 23932412 | PDFSTATSENSEMBLUCSC | 43 | 4 | 13254 | gene;island | 0.339253 | 0.5319708 | Not queried | 1.81 | 3.43e-01 | 0.4672865596 |
|  | 6 | 28973318 | 28973524 | PDFSTATSENSEMBLUCSC | 207 | 11 | 34746 | tss | 0.012767 | 0.0253975 | Not queried | 1.80 | 2.70e-01 | 0.4563244066 |
|  | 6 | 10415597 | 10416090 | PDFSTATSENSEMBLUCSC | 494 | 11 | 33561 | gene;tss;island | 0.547082 | 0.3076797 | Not queried | 1.80 | 1.16e-01 | 0.3175184142 |
|  | 6 | 135501674 | 135501969 | PDFSTATSENSEMBLUCSC | 296 | 4 | 35237 | tss;island | 0.008885 | 0.0010404 | Not queried | 1.80 | 8.86e-01 | 0.8941250130 |
|  | 5 | 83679941 | 83680326 | PDFSTATSENSEMBLUCSC | 386 | 4 | 31672 | gene;island | 0.031451 | 0.1010048 | Not queried | 1.80 | 4.86e-01 | 0.5640816327 |
|  | 1 | 228509380 | 228509762 | PDFSTATSENSEMBLUCSC | 383 | 5 | 1056 | gene;island | 0.396701 | 0.5274892 | Not queried | 1.80 | 3.10e-01 | 0.4672865596 |
|  | 2 | 191207820 | 191208232 | PDFSTATSENSEMBLUCSC | 413 | 5 | 24961 | island | 0.084018 | 0.0243049 | Not queried | 1.80 | 1.51e-01 | 0.3618000441 |
|  | 5 | 38259135 | 38259398 | PDFSTATSENSEMBLUCSC | 264 | 4 | 31594 | gene;island | 0.163367 | 0.0420373 | Not queried | 1.80 | 6.86e-01 | 0.7223604052 |
|  | 5 | 1645268 | 1645802 | PDFSTATSENSEMBLUCSC | 535 | 6 | 32638 | island | 0.118910 | 0.3439365 | Not queried | 1.80 | 6.99e-01 | 0.7290267580 |
|  | 1 | 183385831 | 183386237 | PDFSTATSENSEMBLUCSC | 407 | 4 | 915 | gene;island | 0.124721 | 0.3605279 | Not queried | 1.80 | 4.86e-01 | 0.5640816327 |
|  | 12 | 113587240 | 113587690 | PDFSTATSENSEMBLUCSC | 451 | 6 | 10827 | island | 0.079638 | 0.3508295 | Not queried | 1.80 | 4.85e-01 | 0.5640816327 |
|  | 17 | 15244970 | 15245237 | PDFSTATSENSEMBLUCSC | 268 | 7 | 17150 | tss | 0.005577 | 0.0085946 | Not queried | 1.80 | 5.35e-01 | 0.6176455124 |
|  | 4 | 174427388 | 174428191 | PDFSTATSENSEMBLUCSC | 804 | 9 | 31424 | island | 0.017714 | 0.0435752 | Not queried | 1.80 | 2.22e-01 | 0.3935758299 |
|  | 2 | 72079276 | 72079609 | PDFSTATSENSEMBLUCSC | 334 | 6 | 24402 | island | 0.187642 | 0.4266485 | Not queried | 1.80 | 1.32e-01 | 0.3397986247 |
|  | 3 | 184529753 | 184529967 | PDFSTATSENSEMBLUCSC | 215 | 6 | 29734 | island | 0.003610 | 0.0070596 | Not queried | 1.80 | 1.80e-01 | 0.3826488233 |
|  | 18 | 24445134 | 24445318 | PDFSTATSENSEMBLUCSC | 185 | 4 | 19082 | gene;island | 0.189835 | 0.4069941 | Not queried | 1.80 | 3.43e-01 | 0.4672865596 |
|  | 8 | 1365502 | 1365906 | PDFSTATSENSEMBLUCSC | 405 | 4 | 40024 | island | 0.341273 | 0.5012772 | Not queried | 1.80 | 4.86e-01 | 0.5640816327 |
|  | 6 | 31276088 | 31276212 | PDFSTATSENSEMBLUCSC | 125 | 5 | 35763 | island | 0.088168 | 0.1788566 | Not queried | 1.80 | 4.21e-01 | 0.5404252807 |
|  | 8 | 25042340 | 25042630 | PDFSTATSENSEMBLUCSC | 291 | 4 | 39248 | gene;island | 0.005618 | 0.0328772 | Not queried | 1.80 | 2.00e-01 | 0.3826488233 |
|  | 5 | 145316210 | 145316416 | PDFSTATSENSEMBLUCSC | 207 | 5 | 31849 | gene | 0.036536 | 0.0977517 | Not queried | 1.80 | 2.22e-01 | 0.3935758299 |
|  | 11 | 121970613 | 121970768 | PDFSTATSENSEMBLUCSC | 156 | 5 | 7769 | tss | 0.815645 | 0.9406248 | Not queried | 1.80 | 1.51e-01 | 0.3618000441 |
|  | 15 | 31195612 | 31195982 | PDFSTATSENSEMBLUCSC | 371 | 8 | 13549 | tss | 0.033922 | 0.0121498 | Not queried | 1.80 | 2.79e-01 | 0.4672865596 |
|  | 6 | 31589593 | 31589994 | PDFSTATSENSEMBLUCSC | 402 | 5 | 33909 | gene;tss;island | 0.334684 | 0.4499689 | Not queried | 1.80 | 3.10e-01 | 0.4672865596 |
|  | 1 | 181454975 | 181455181 | PDFSTATSENSEMBLUCSC | 207 | 4 | 909 | gene;island | 0.017835 | 0.0698857 | Not queried | 1.80 | 5.71e-02 | 0.2315877671 |
|  | 14 | 94255361 | 94255513 | PDFSTATSENSEMBLUCSC | 153 | 4 | 12576 | tss;island | 0.022678 | 0.0425318 | Not queried | 1.80 | 4.86e-01 | 0.5640816327 |
|  | 1 | 27683345 | 27683501 | PDFSTATSENSEMBLUCSC | 157 | 4 | 352 | gene;island | 0.916649 | 0.9655393 | Not queried | 1.80 | 5.71e-02 | 0.2315877671 |
|  | 11 | 2323801 | 2323938 | PDFSTATSENSEMBLUCSC | 138 | 4 | 6455 | gene;tss | 0.028306 | 0.1069675 | Not queried | 1.80 | 4.86e-01 | 0.5640816327 |
|  | 5 | 134871462 | 134872315 | PDFSTATSENSEMBLUCSC | 854 | 21 | 33138 | island | 0.014745 | 0.0377073 | Not queried | 1.80 | 2.75e-02 | 0.1830867741 |
|  | 15 | 52404977 | 52405162 | PDFSTATSENSEMBLUCSC | 186 | 7 | 13677 | tss | 0.620366 | 0.2554756 | Not queried | 1.80 | 6.20e-01 | 0.6899391537 |
|  | 11 | 43601969 | 43602179 | PDFSTATSENSEMBLUCSC | 211 | 4 | 7253 | tss;island | 0.026131 | 0.0765721 | Not queried | 1.79 | 1.14e-01 | 0.3123457953 |
|  | 11 | 36422377 | 36422615 | PDFSTATSENSEMBLUCSC | 239 | 5 | 6585 | gene | 0.281281 | 0.1246554 | Not queried | 1.79 | 3.10e-01 | 0.4672865596 |
|  | 1 | 151810887 | 151810904 | PDFSTATSENSEMBLUCSC | 18 | 4 | 755 | gene;tss;island | 0.011861 | 0.0478482 | Not queried | 1.79 | 2.86e-02 | 0.1830867741 |
|  | 20 | 44258197 | 44258437 | PDFSTATSENSEMBLUCSC | 241 | 5 | 25431 | gene | 0.091807 | 0.4450900 | Not queried | 1.79 | 4.21e-01 | 0.5404252807 |
|  | 13 | 37248078 | 37248423 | PDFSTATSENSEMBLUCSC | 346 | 4 | 11133 | gene;island | 0.071313 | 0.1571770 | Not queried | 1.79 | 3.43e-01 | 0.4672865596 |
|  | 15 | 65648103 | 65648490 | PDFSTATSENSEMBLUCSC | 388 | 4 | 13379 | gene;island | 0.476456 | 0.1789580 | Not queried | 1.79 | 2.86e-02 | 0.1830867741 |
|  | 2 | 228736089 | 228736324 | PDFSTATSENSEMBLUCSC | 236 | 6 | 23932 | tss | 0.012946 | 0.0406914 | Not queried | 1.79 | 3.94e-01 | 0.5236526859 |
|  | 14 | 101532886 | 101533127 | PDFSTATSENSEMBLUCSC | 242 | 4 | 13154 | island | 0.386514 | 0.6136881 | Not queried | 1.79 | 2.00e-01 | 0.3826488233 |
|  | 1 | 226410871 | 226411270 | PDFSTATSENSEMBLUCSC | 400 | 8 | 2288 | tss;island | 0.050233 | 0.2854047 | Not queried | 1.79 | 2.79e-01 | 0.4672865596 |
|  | 7 | 120968877 | 120969174 | PDFSTATSENSEMBLUCSC | 298 | 8 | 37203 | gene;island | 0.067727 | 0.3832460 | Not queried | 1.79 | 2.07e-02 | 0.1830867741 |
|  | 1 | 119531625 | 119532116 | PDFSTATSENSEMBLUCSC | 492 | 7 | 716 | gene | 0.054377 | 0.1418638 | Not queried | 1.79 | 7.28e-02 | 0.2713481487 |
|  | 2 | 158114069 | 158114300 | PDFSTATSENSEMBLUCSC | 232 | 4 | 23676 | tss | 0.332850 | 0.0522101 | Not queried | 1.79 | 6.86e-01 | 0.7223604052 |
|  | 6 | 26103952 | 26104560 | PDFSTATSENSEMBLUCSC | 609 | 9 | 35514 | island | 0.002758 | 0.0158735 | Not queried | 1.79 | 5.03e-02 | 0.2315877671 |
|  | 11 | 44341361 | 44341831 | PDFSTATSENSEMBLUCSC | 471 | 5 | 8227 | island | 0.059698 | 0.1751281 | Not queried | 1.79 | 4.21e-01 | 0.5404252807 |
|  | 1 | 205290905 | 205291215 | PDFSTATSENSEMBLUCSC | 311 | 4 | 2204 | tss | 0.080803 | 0.1478778 | Not queried | 1.79 | 3.43e-01 | 0.4672865596 |
|  | 17 | 72208213 | 72208504 | PDFSTATSENSEMBLUCSC | 292 | 4 | 16748 | gene;tss;island | 0.476178 | 0.7604797 | Not queried | 1.79 | 4.86e-01 | 0.5640816327 |
|  | 11 | 1104295 | 1104527 | PDFSTATSENSEMBLUCSC | 233 | 4 | 7903 | island | 0.728508 | 0.9542502 | Not queried | 1.79 | 2.00e-01 | 0.3826488233 |
|  | 7 | 158936508 | 158936739 | PDFSTATSENSEMBLUCSC | 232 | 4 | 37385 | gene;island | 0.030126 | 0.0107116 | Not queried | 1.79 | 3.43e-01 | 0.4672865596 |
|  | 6 | 166571782 | 166572045 | PDFSTATSENSEMBLUCSC | 264 | 5 | 34543 | gene;island | 0.697891 | 0.7357168 | Not queried | 1.79 | 5.48e-01 | 0.6179718485 |
|  | 2 | 197457277 | 197457430 | PDFSTATSENSEMBLUCSC | 154 | 6 | 24975 | island | 0.018179 | 0.0434348 | Not queried | 1.79 | 2.40e-01 | 0.4133265075 |
|  | 10 | 134901199 | 134901305 | PDFSTATSENSEMBLUCSC | 107 | 7 | 5452 | tss | 0.076554 | 0.0236159 | Not queried | 1.79 | 3.79e-02 | 0.2121545571 |
|  | 2 | 232790974 | 232791126 | PDFSTATSENSEMBLUCSC | 153 | 6 | 23946 | tss;island | 0.033863 | 0.0153290 | Not queried | 1.79 | 1.32e-01 | 0.3397986247 |
|  | 8 | 124214953 | 124215152 | PDFSTATSENSEMBLUCSC | 200 | 4 | 39418 | gene | 0.474803 | 0.7502735 | Not queried | 1.79 | 4.86e-01 | 0.5640816327 |
|  | 1 | 27189270 | 27189440 | PDFSTATSENSEMBLUCSC | 171 | 4 | 1399 | tss;island | 0.491915 | 0.0938689 | Not queried | 1.78 | 4.86e-01 | 0.5640816327 |
|  | 10 | 72201417 | 72201741 | PDFSTATSENSEMBLUCSC | 325 | 7 | 5819 | island | 0.132182 | 0.1140739 | Not queried | 1.78 | 6.20e-01 | 0.6899391537 |
|  | 11 | 134147143 | 134147634 | PDFSTATSENSEMBLUCSC | 492 | 4 | 6994 | gene;island | 0.015154 | 0.3378675 | Not queried | 1.78 | 4.86e-01 | 0.5640816327 |
|  | 14 | 75389066 | 75389280 | PDFSTATSENSEMBLUCSC | 215 | 4 | 13009 | island | 0.743246 | 0.9406826 | Not queried | 1.78 | 1.14e-01 | 0.3123457953 |
|  | 10 | 85324700 | 85325062 | PDFSTATSENSEMBLUCSC | 363 | 5 | 5892 | island | 0.590998 | 0.8728752 | Not queried | 1.78 | 4.21e-01 | 0.5404252807 |
|  | 1 | 27240319 | 27240514 | PDFSTATSENSEMBLUCSC | 196 | 4 | 347 | gene | 0.457996 | 0.7339096 | Not queried | 1.78 | 2.00e-01 | 0.3826488233 |
|  | 3 | 49907297 | 49907692 | PDFSTATSENSEMBLUCSC | 396 | 7 | 29132 | island | 0.035726 | 0.1155753 | Not queried | 1.78 | 4.56e-01 | 0.5640816327 |
|  | 11 | 2019079 | 2019167 | PDFSTATSENSEMBLUCSC | 89 | 5 | 7054 | tss;island | 0.331107 | 0.6547609 | Not queried | 1.78 | 1.59e-02 | 0.1830867741 |
|  | 22 | 50493126 | 50493212 | PDFSTATSENSEMBLUCSC | 87 | 4 | 27243 | tss | 0.731736 | 0.9456839 | Not queried | 1.78 | 1.14e-01 | 0.3123457953 |
|  | 20 | 44098223 | 44098387 | PDFSTATSENSEMBLUCSC | 165 | 6 | 25776 | tss | 0.140976 | 0.1813798 | Not queried | 1.78 | 3.94e-01 | 0.5236526859 |
|  | 19 | 45873596 | 45873826 | PDFSTATSENSEMBLUCSC | 231 | 4 | 20093 | gene | 0.003621 | 0.0023449 | Not queried | 1.78 | 2.86e-02 | 0.1830867741 |
|  | 17 | 72427538 | 72427693 | PDFSTATSENSEMBLUCSC | 156 | 6 | 18718 | island | 0.026987 | 0.1010059 | Not queried | 1.78 | 2.60e-02 | 0.1830867741 |
|  | 20 | 25566178 | 25566563 | PDFSTATSENSEMBLUCSC | 386 | 7 | 25652 | tss;island | 0.074853 | 0.0423922 | Not queried | 1.78 | 3.18e-01 | 0.4672865596 |
|  | 13 | 98829311 | 98829666 | PDFSTATSENSEMBLUCSC | 356 | 8 | 11197 | gene;island | 0.537625 | 0.8398140 | Not queried | 1.78 | 3.79e-02 | 0.2121545571 |
|  | 11 | 1506936 | 1507347 | PDFSTATSENSEMBLUCSC | 412 | 6 | 6423 | gene | 0.495356 | 0.8137552 | Not queried | 1.78 | 9.31e-02 | 0.3104222821 |
|  | 13 | 112724221 | 112724583 | PDFSTATSENSEMBLUCSC | 363 | 5 | 11246 | gene;island | 0.457268 | 0.2132542 | Not queried | 1.78 | 4.21e-01 | 0.5404252807 |
|  | 7 | 154863176 | 154863381 | PDFSTATSENSEMBLUCSC | 206 | 4 | 37305 | gene;island | 0.256657 | 0.4852402 | Not queried | 1.78 | 3.43e-01 | 0.4672865596 |
|  | 11 | 74870268 | 74870741 | PDFSTATSENSEMBLUCSC | 474 | 5 | 6831 | gene;tss | 0.825803 | 0.9132805 | Not queried | 1.78 | 3.10e-01 | 0.4672865596 |
|  | 1 | 228473704 | 228473980 | PDFSTATSENSEMBLUCSC | 277 | 5 | 1054 | gene;island | 0.178439 | 0.5230743 | Not queried | 1.78 | 9.52e-02 | 0.3104222821 |
|  | 12 | 66123127 | 66123496 | PDFSTATSENSEMBLUCSC | 370 | 4 | 10600 | island | 0.380490 | 0.1222140 | Not queried | 1.78 | 6.86e-01 | 0.7223604052 |
|  | 19 | 43979316 | 43979739 | PDFSTATSENSEMBLUCSC | 424 | 6 | 20074 | gene;island | 0.041944 | 0.1107018 | Not queried | 1.78 | 3.94e-01 | 0.5236526859 |
|  | 13 | 33780205 | 33780362 | PDFSTATSENSEMBLUCSC | 158 | 4 | 11386 | tss | 0.025838 | 0.0096066 | Not queried | 1.78 | 3.43e-01 | 0.4672865596 |
|  | 19 | 4566400 | 4566677 | PDFSTATSENSEMBLUCSC | 278 | 5 | 21381 | island | 0.539492 | 0.5707105 | Not queried | 1.78 | 4.21e-01 | 0.5404252807 |
|  | 7 | 1278142 | 1278508 | PDFSTATSENSEMBLUCSC | 367 | 4 | 38093 | island | 0.074833 | 0.2232519 | Not queried | 1.78 | 4.86e-01 | 0.5640816327 |
|  | 2 | 139537745 | 139537845 | PDFSTATSENSEMBLUCSC | 101 | 4 | 24764 | island | 0.034089 | 0.0799965 | Not queried | 1.78 | 2.86e-02 | 0.1830867741 |
|  | 14 | 104551867 | 104552397 | PDFSTATSENSEMBLUCSC | 531 | 6 | 13199 | island | 0.610496 | 0.8373741 | Not queried | 1.78 | 3.94e-01 | 0.5236526859 |
|  | 14 | 104551867 | 104552034 | PDFSTATSENSEMBLUCSC | 168 | 4 | 12656 | tss | 0.352593 | 0.6474712 | Not queried | 1.78 | 6.86e-01 | 0.7223604052 |
|  | 12 | 107711872 | 107712194 | PDFSTATSENSEMBLUCSC | 323 | 5 | 9975 | tss | 0.010686 | 0.0027662 | Not queried | 1.78 | 5.48e-01 | 0.6179718485 |
|  | 3 | 184032711 | 184033015 | PDFSTATSENSEMBLUCSC | 305 | 4 | 28061 | gene;island | 0.006698 | 0.0018366 | Not queried | 1.78 | 6.86e-01 | 0.7223604052 |
|  | 21 | 44496158 | 44496378 | PDFSTATSENSEMBLUCSC | 221 | 5 | 26573 | tss | 0.006615 | 0.0150632 | Not queried | 1.78 | 4.21e-01 | 0.5404252807 |
|  | 1 | 7887199 | 7887560 | PDFSTATSENSEMBLUCSC | 362 | 5 | 190 | gene;island | 0.757511 | 0.9134019 | Not queried | 1.78 | 4.21e-01 | 0.5404252807 |
|  | 7 | 39989474 | 39989867 | PDFSTATSENSEMBLUCSC | 394 | 6 | 37548 | tss | 0.002798 | 0.0056767 | Not queried | 1.78 | 5.89e-01 | 0.6570484697 |
|  | 1 | 114447334 | 114447605 | PDFSTATSENSEMBLUCSC | 272 | 5 | 699 | gene | 0.006483 | 0.0164463 | Not queried | 1.78 | 1.51e-01 | 0.3618000441 |
|  | 4 | 22389706 | 22390126 | PDFSTATSENSEMBLUCSC | 421 | 5 | 29967 | gene | 0.569250 | 0.8650862 | Not queried | 1.77 | 4.21e-01 | 0.5404252807 |
|  | 14 | 62229147 | 62229330 | PDFSTATSENSEMBLUCSC | 184 | 4 | 12108 | gene | 0.012309 | 0.0190787 | Not queried | 1.77 | 4.86e-01 | 0.5640816327 |
|  | 8 | 33457226 | 33457822 | PDFSTATSENSEMBLUCSC | 597 | 8 | 40215 | island | 0.055377 | 0.0160468 | Not queried | 1.77 | 8.30e-02 | 0.3028612233 |
|  | 22 | 19949873 | 19950166 | PDFSTATSENSEMBLUCSC | 294 | 7 | 26794 | gene | 0.416448 | 0.5344807 | Not queried | 1.77 | 1.00e+00 | 1.0000000000 |
|  | 19 | 15284853 | 15285108 | PDFSTATSENSEMBLUCSC | 256 | 5 | 19901 | gene;island | 0.667393 | 0.8723853 | Not queried | 1.77 | 5.48e-01 | 0.6179718485 |
|  | 11 | 36615733 | 36616048 | PDFSTATSENSEMBLUCSC | 316 | 6 | 7250 | tss | 0.053299 | 0.5450694 | Not queried | 1.77 | 5.89e-01 | 0.6570484697 |
|  | 16 | 88547533 | 88547861 | PDFSTATSENSEMBLUCSC | 329 | 4 | 14903 | gene | 0.856837 | 0.9534248 | Not queried | 1.77 | 3.43e-01 | 0.4672865596 |
|  | 2 | 240302585 | 240302855 | PDFSTATSENSEMBLUCSC | 271 | 4 | 23118 | gene | 0.029434 | 0.1852496 | Not queried | 1.77 | 3.43e-01 | 0.4672865596 |
|  | 17 | 42247565 | 42248062 | PDFSTATSENSEMBLUCSC | 498 | 6 | 17403 | tss | 0.443762 | 0.3065741 | Not queried | 1.77 | 3.94e-01 | 0.5236526859 |
|  | 11 | 44330731 | 44331108 | PDFSTATSENSEMBLUCSC | 378 | 4 | 6590 | gene;island | 0.116237 | 0.0155645 | Not queried | 1.77 | 6.86e-01 | 0.7223604052 |
|  | 15 | 37402488 | 37402723 | PDFSTATSENSEMBLUCSC | 236 | 4 | 14014 | island | 0.444090 | 0.0564171 | Not queried | 1.77 | 6.86e-01 | 0.7223604052 |
|  | 1 | 115396277 | 115396629 | PDFSTATSENSEMBLUCSC | 353 | 4 | 1856 | tss;island | 0.628995 | 0.8334914 | Not queried | 1.77 | 6.86e-01 | 0.7223604052 |
|  | 8 | 63161579 | 63161676 | PDFSTATSENSEMBLUCSC | 98 | 5 | 39318 | gene;island | 0.079013 | 0.0102500 | Not queried | 1.77 | 1.51e-01 | 0.3618000441 |
|  | 7 | 8480586 | 8481036 | PDFSTATSENSEMBLUCSC | 451 | 4 | 36891 | gene;island | 0.226905 | 0.1786723 | Not queried | 1.77 | 6.86e-01 | 0.7223604052 |
|  | 5 | 57755773 | 57755881 | PDFSTATSENSEMBLUCSC | 109 | 4 | 31622 | gene | 0.010178 | 0.0351831 | Not queried | 1.77 | 2.00e-01 | 0.3826488233 |
|  | 3 | 140770213 | 140770683 | PDFSTATSENSEMBLUCSC | 471 | 11 | 28637 | tss | 0.007307 | 0.0294311 | Not queried | 1.77 | 2.17e-01 | 0.3935758299 |
|  | 14 | 106938234 | 106938451 | PDFSTATSENSEMBLUCSC | 218 | 7 | 12676 | tss | 0.942402 | 0.9729162 | Not queried | 1.77 | 9.73e-02 | 0.3123457953 |
|  | 2 | 229046406 | 229046785 | PDFSTATSENSEMBLUCSC | 380 | 4 | 23933 | tss | 0.235960 | 0.2805691 | Not queried | 1.77 | 8.86e-01 | 0.8941250130 |
|  | 16 | 82044738 | 82045078 | PDFSTATSENSEMBLUCSC | 341 | 5 | 14857 | gene | 0.042743 | 0.0189652 | Not queried | 1.77 | 3.10e-01 | 0.4672865596 |
|  | 12 | 133463607 | 133464074 | PDFSTATSENSEMBLUCSC | 468 | 5 | 9469 | gene;island | 0.024006 | 0.0219735 | Not queried | 1.77 | 8.41e-01 | 0.8635565145 |
|  | 7 | 3019159 | 3019382 | PDFSTATSENSEMBLUCSC | 224 | 4 | 36821 | gene | 0.034878 | 0.1160977 | Not queried | 1.77 | 2.00e-01 | 0.3826488233 |
|  | 1 | 179711859 | 179712281 | PDFSTATSENSEMBLUCSC | 423 | 8 | 2120 | tss | 0.158089 | 0.0439092 | Not queried | 1.77 | 3.79e-02 | 0.2121545571 |
|  | 7 | 72935613 | 72935712 | PDFSTATSENSEMBLUCSC | 100 | 4 | 37036 | gene;island | 0.019728 | 0.0438160 | Not queried | 1.77 | 6.86e-01 | 0.7223604052 |
|  | 13 | 115079815 | 115079979 | PDFSTATSENSEMBLUCSC | 165 | 4 | 12002 | island | 0.001078 | 0.0033042 | Not queried | 1.77 | 4.86e-01 | 0.5640816327 |
|  | 18 | 44557635 | 44557819 | PDFSTATSENSEMBLUCSC | 185 | 5 | 19105 | gene;tss;island | 0.362254 | 0.8108857 | Not queried | 1.77 | 9.52e-02 | 0.3104222821 |
|  | 8 | 41166990 | 41167278 | PDFSTATSENSEMBLUCSC | 289 | 6 | 39667 | tss;island | 0.475936 | 0.1491902 | Not queried | 1.77 | 4.11e-02 | 0.2180389943 |
|  | 11 | 63852804 | 63853037 | PDFSTATSENSEMBLUCSC | 234 | 5 | 6662 | gene;island | 0.924688 | 0.9805733 | Not queried | 1.77 | 9.52e-02 | 0.3104222821 |
|  | 19 | 13985513 | 13985688 | PDFSTATSENSEMBLUCSC | 176 | 4 | 19885 | gene | 0.632594 | 0.7821732 | Not queried | 1.77 | 4.86e-01 | 0.5640816327 |
|  | 8 | 61193970 | 61194292 | PDFSTATSENSEMBLUCSC | 323 | 9 | 39724 | tss | 0.064075 | 0.0157552 | Not queried | 1.76 | 3.99e-03 | 0.1459191215 |
|  | 12 | 56882314 | 56882693 | PDFSTATSENSEMBLUCSC | 380 | 7 | 9800 | tss;island | 0.042968 | 0.0839836 | Not queried | 1.76 | 5.35e-01 | 0.6176455124 |
|  | 1 | 20813574 | 20813666 | PDFSTATSENSEMBLUCSC | 93 | 4 | 1313 | tss;island | 0.123202 | 0.0347390 | Not queried | 1.76 | 3.43e-01 | 0.4672865596 |
|  | 12 | 95945082 | 95945384 | PDFSTATSENSEMBLUCSC | 303 | 5 | 10689 | island | 0.673580 | 0.8773202 | Not queried | 1.76 | 9.52e-02 | 0.3104222821 |
|  | 14 | 50328815 | 50328966 | PDFSTATSENSEMBLUCSC | 152 | 4 | 12826 | island | 0.001721 | 0.0095070 | Not queried | 1.76 | 2.00e-01 | 0.3826488233 |
|  | 11 | 108408950 | 108408981 | PDFSTATSENSEMBLUCSC | 32 | 4 | 7673 | tss | 0.983349 | 0.9236018 | Not queried | 1.76 | 1.14e-01 | 0.3123457953 |
|  | 6 | 148881126 | 148881461 | PDFSTATSENSEMBLUCSC | 336 | 4 | 36551 | island | 0.460081 | 0.2497338 | Not queried | 1.76 | 6.86e-01 | 0.7223604052 |
|  | 1 | 3306999 | 3307215 | PDFSTATSENSEMBLUCSC | 217 | 4 | 122 | gene;island | 0.736343 | 0.8683705 | Not queried | 1.76 | 3.43e-01 | 0.4672865596 |
|  | 12 | 125399699 | 125399964 | PDFSTATSENSEMBLUCSC | 266 | 5 | 10110 | tss;island | 0.010623 | 0.0029807 | Not queried | 1.76 | 2.22e-01 | 0.3935758299 |
|  | 12 | 5018715 | 5019066 | PDFSTATSENSEMBLUCSC | 352 | 9 | 9502 | tss | 0.031339 | 0.0201291 | Not queried | 1.76 | 3.40e-01 | 0.4672865596 |
|  | 19 | 58095424 | 58095595 | PDFSTATSENSEMBLUCSC | 172 | 7 | 21120 | tss | 0.083536 | 0.0149028 | Not queried | 1.76 | 3.79e-02 | 0.2121545571 |
|  | 8 | 88886051 | 88886432 | PDFSTATSENSEMBLUCSC | 382 | 9 | 40440 | island | 0.800661 | 0.9569898 | Not queried | 1.76 | 4.00e-02 | 0.2180389943 |
|  | 3 | 50313996 | 50314344 | PDFSTATSENSEMBLUCSC | 349 | 4 | 27799 | gene;island | 0.909612 | 0.5980592 | Not queried | 1.76 | 2.00e-01 | 0.3826488233 |
|  | 17 | 8090466 | 8090696 | PDFSTATSENSEMBLUCSC | 231 | 4 | 17995 | island | 0.007110 | 0.0045152 | Not queried | 1.76 | 4.86e-01 | 0.5640816327 |
|  | 12 | 31744043 | 31744436 | PDFSTATSENSEMBLUCSC | 394 | 4 | 9627 | tss | 0.009055 | 0.0178849 | Not queried | 1.76 | 1.14e-01 | 0.3123457953 |
|  | 12 | 54346784 | 54347242 | PDFSTATSENSEMBLUCSC | 459 | 5 | 10481 | island | 0.035339 | 0.1591735 | Not queried | 1.76 | 8.41e-01 | 0.8635565145 |
|  | 8 | 65489863 | 65489939 | PDFSTATSENSEMBLUCSC | 77 | 5 | 39734 | tss;island | 0.060441 | 0.0119509 | Not queried | 1.76 | 5.48e-01 | 0.6179718485 |
|  | 10 | 102997782 | 102998117 | PDFSTATSENSEMBLUCSC | 336 | 6 | 4728 | gene;island | 0.201032 | 0.0497788 | Not queried | 1.76 | 2.40e-01 | 0.4133265075 |
|  | 8 | 143859669 | 143859990 | PDFSTATSENSEMBLUCSC | 322 | 7 | 39938 | tss;island | 0.368854 | 0.5738676 | Not queried | 1.76 | 1.28e-01 | 0.3397986247 |
|  | 6 | 44310228 | 44310350 | PDFSTATSENSEMBLUCSC | 123 | 6 | 35013 | tss | 0.029705 | 0.1438887 | Not queried | 1.76 | 2.60e-02 | 0.1830867741 |
|  | 17 | 96392 | 96796 | PDFSTATSENSEMBLUCSC | 405 | 6 | 16285 | gene;island | 0.554628 | 0.7280863 | Not queried | 1.76 | 3.10e-01 | 0.4672865596 |
|  | 1 | 160039847 | 160040667 | PDFSTATSENSEMBLUCSC | 821 | 13 | 3887 | island | 0.028228 | 0.1573996 | Not queried | 1.76 | 8.60e-03 | 0.1595382395 |
|  | 1 | 160039962 | 160040667 | PDFSTATSENSEMBLUCSC | 706 | 11 | 2035 | tss | 0.028228 | 0.1870986 | Not queried | 1.76 | 1.04e-02 | 0.1806165940 |
|  | 18 | 11851233 | 11851347 | PDFSTATSENSEMBLUCSC | 115 | 6 | 19184 | tss | 0.005811 | 0.0025104 | Not queried | 1.76 | 3.10e-01 | 0.4672865596 |
|  | 1 | 206786000 | 206786222 | PDFSTATSENSEMBLUCSC | 223 | 6 | 2215 | tss;island | 0.903707 | 0.5346124 | Not queried | 1.76 | 9.31e-02 | 0.3104222821 |
|  | 7 | 29519297 | 29519450 | PDFSTATSENSEMBLUCSC | 154 | 5 | 37511 | tss | 0.250872 | 0.3611472 | Not queried | 1.76 | 6.90e-01 | 0.7223604052 |
|  | 5 | 7826845 | 7827133 | PDFSTATSENSEMBLUCSC | 289 | 5 | 31555 | gene | 0.156455 | 0.3920735 | Not queried | 1.76 | 1.51e-01 | 0.3618000441 |
|  | 18 | 21852313 | 21852436 | PDFSTATSENSEMBLUCSC | 124 | 5 | 19211 | tss | 0.005776 | 0.0151450 | Not queried | 1.76 | 5.48e-01 | 0.6179718485 |
|  | 12 | 81471311 | 81471757 | PDFSTATSENSEMBLUCSC | 447 | 7 | 9894 | tss | 0.025681 | 0.0524423 | Not queried | 1.75 | 3.18e-01 | 0.4672865596 |
|  | 6 | 50803820 | 50803945 | PDFSTATSENSEMBLUCSC | 126 | 4 | 34365 | gene | 0.051853 | 0.2900521 | Not queried | 1.75 | 6.86e-01 | 0.7223604052 |
|  | 7 | 134576049 | 134576386 | PDFSTATSENSEMBLUCSC | 338 | 6 | 37233 | gene | 0.076116 | 0.3859200 | Not queried | 1.75 | 3.94e-01 | 0.5236526859 |
|  | 5 | 168174689 | 168174935 | PDFSTATSENSEMBLUCSC | 247 | 4 | 31885 | gene | 0.417254 | 0.6873336 | Not queried | 1.75 | 4.86e-01 | 0.5640816327 |
|  | 11 | 129939665 | 129939958 | PDFSTATSENSEMBLUCSC | 294 | 8 | 8954 | island | 0.003124 | 0.0133581 | Not queried | 1.75 | 2.34e-01 | 0.4128374386 |
|  | 11 | 129939817 | 129939958 | PDFSTATSENSEMBLUCSC | 142 | 4 | 6972 | gene | 0.001338 | 0.0123683 | Not queried | 1.75 | 3.43e-01 | 0.4672865596 |
|  | 10 | 118033581 | 118034031 | PDFSTATSENSEMBLUCSC | 451 | 4 | 5383 | tss;island | 0.027019 | 0.0415705 | Not queried | 1.75 | 4.86e-01 | 0.5640816327 |
|  | 17 | 42147905 | 42148070 | PDFSTATSENSEMBLUCSC | 166 | 7 | 17400 | tss | 0.004271 | 0.0108313 | Not queried | 1.75 | 3.18e-01 | 0.4672865596 |
|  | 10 | 50816917 | 50817306 | PDFSTATSENSEMBLUCSC | 390 | 6 | 5073 | tss;island | 0.014067 | 0.0353188 | Not queried | 1.75 | 2.40e-01 | 0.4133265075 |
|  | 1 | 3426236 | 3426532 | PDFSTATSENSEMBLUCSC | 297 | 4 | 127 | gene;island | 0.351326 | 0.7245160 | Not queried | 1.75 | 4.86e-01 | 0.5640816327 |
|  | 11 | 66725860 | 66725961 | PDFSTATSENSEMBLUCSC | 102 | 5 | 7482 | tss | 0.045125 | 0.0189259 | Not queried | 1.75 | 4.21e-01 | 0.5404252807 |
|  | 5 | 140165938 | 140166213 | PDFSTATSENSEMBLUCSC | 276 | 4 | 31793 | gene;island | 0.300974 | 0.5882800 | Not queried | 1.75 | 3.43e-01 | 0.4672865596 |
|  | 2 | 172949281 | 172949501 | PDFSTATSENSEMBLUCSC | 221 | 4 | 23716 | tss;island | 0.177888 | 0.0403964 | Not queried | 1.75 | 2.86e-02 | 0.1830867741 |
|  | 3 | 77147070 | 77147364 | PDFSTATSENSEMBLUCSC | 295 | 5 | 27862 | gene;island | 0.373978 | 0.7050930 | Not queried | 1.75 | 3.10e-01 | 0.4672865596 |
|  | 7 | 4901337 | 4901798 | PDFSTATSENSEMBLUCSC | 462 | 7 | 36862 | gene;island | 0.668782 | 0.4343964 | Not queried | 1.75 | 2.09e-01 | 0.3935758299 |
|  | 2 | 85107190 | 85107488 | PDFSTATSENSEMBLUCSC | 299 | 5 | 22759 | gene;island | 0.009965 | 0.0323884 | Not queried | 1.75 | 7.94e-03 | 0.1589602024 |
|  | 2 | 74757141 | 74757221 | PDFSTATSENSEMBLUCSC | 81 | 5 | 22749 | gene;tss;island | 0.002843 | 0.0040924 | Not queried | 1.75 | 3.10e-01 | 0.4672865596 |
|  | 2 | 240031454 | 240031700 | PDFSTATSENSEMBLUCSC | 247 | 4 | 23105 | gene;island | 0.916871 | 0.6861846 | Not queried | 1.75 | 3.43e-01 | 0.4672865596 |
|  | 5 | 134209784 | 134210024 | PDFSTATSENSEMBLUCSC | 241 | 4 | 31753 | gene;island | 0.024524 | 0.0204960 | Not queried | 1.75 | 6.86e-01 | 0.7223604052 |
|  | 11 | 122850490 | 122850741 | PDFSTATSENSEMBLUCSC | 252 | 4 | 6949 | gene;island | 0.097688 | 0.2827586 | Not queried | 1.75 | 3.43e-01 | 0.4672865596 |
|  | 2 | 6121909 | 6122107 | PDFSTATSENSEMBLUCSC | 199 | 4 | 23165 | tss | 0.038584 | 0.0180359 | Not queried | 1.75 | 4.86e-01 | 0.5640816327 |
|  | 12 | 108955167 | 108955348 | PDFSTATSENSEMBLUCSC | 182 | 6 | 9982 | tss;island | 0.000809 | 0.0012391 | Not queried | 1.75 | 1.00e+00 | 1.0000000000 |
|  | 2 | 79220029 | 79220528 | PDFSTATSENSEMBLUCSC | 500 | 5 | 24460 | island | 0.020564 | 0.0581392 | Not queried | 1.75 | 9.52e-02 | 0.3104222821 |
|  | 17 | 61988667 | 61988811 | PDFSTATSENSEMBLUCSC | 145 | 4 | 17576 | tss | 0.454891 | 0.4434571 | Not queried | 1.75 | 6.86e-01 | 0.7223604052 |
|  | 10 | 444914 | 445215 | PDFSTATSENSEMBLUCSC | 302 | 4 | 4437 | gene;island | 0.766901 | 0.8856956 | Not queried | 1.74 | 3.43e-01 | 0.4672865596 |
|  | 16 | 963814 | 963994 | PDFSTATSENSEMBLUCSC | 181 | 4 | 14552 | gene;island | 0.579272 | 0.8196708 | Not queried | 1.74 | 1.14e-01 | 0.3123457953 |
|  | 11 | 6292311 | 6292615 | PDFSTATSENSEMBLUCSC | 305 | 4 | 6489 | gene;island | 0.801710 | 0.9558057 | Not queried | 1.74 | 3.43e-01 | 0.4672865596 |
|  | 19 | 54372324 | 54373043 | PDFSTATSENSEMBLUCSC | 720 | 13 | 20209 | gene;island | 0.009573 | 0.0305724 | Not queried | 1.74 | 1.64e-02 | 0.1830867741 |
|  | 19 | 54372324 | 54372713 | PDFSTATSENSEMBLUCSC | 390 | 9 | 21043 | tss | 0.009573 | 0.0347701 | Not queried | 1.74 | 6.25e-02 | 0.2477578846 |
|  | 15 | 91537812 | 91538344 | PDFSTATSENSEMBLUCSC | 533 | 8 | 13909 | tss | 0.004777 | 0.0113703 | Not queried | 1.74 | 2.34e-01 | 0.4128374386 |
|  | 18 | 76552701 | 76552971 | PDFSTATSENSEMBLUCSC | 271 | 4 | 19592 | island | 0.675918 | 0.9084694 | Not queried | 1.74 | 1.14e-01 | 0.3123457953 |
|  | 22 | 39323204 | 39323648 | PDFSTATSENSEMBLUCSC | 445 | 4 | 27498 | island | 0.286197 | 0.0187000 | Not queried | 1.74 | 3.43e-01 | 0.4672865596 |
|  | 4 | 155661691 | 155661929 | PDFSTATSENSEMBLUCSC | 239 | 4 | 31384 | island | 0.803106 | 0.9241524 | Not queried | 1.74 | 3.43e-01 | 0.4672865596 |
|  | 19 | 15530606 | 15530870 | PDFSTATSENSEMBLUCSC | 265 | 4 | 20561 | tss;island | 0.301177 | 0.6759827 | Not queried | 1.74 | 6.86e-01 | 0.7223604052 |
|  | 12 | 5018715 | 5019091 | PDFSTATSENSEMBLUCSC | 377 | 10 | 10180 | island | 0.028015 | 0.0344179 | Not queried | 1.74 | 4.81e-01 | 0.5640816327 |
|  | 20 | 61051317 | 61052060 | PDFSTATSENSEMBLUCSC | 744 | 18 | 25872 | tss;island | 0.018981 | 0.0336903 | Not queried | 1.74 | 1.43e-01 | 0.3618000441 |
|  | 15 | 90456248 | 90456686 | PDFSTATSENSEMBLUCSC | 439 | 5 | 13894 | tss | 0.035608 | 0.3630170 | Not queried | 1.74 | 2.22e-01 | 0.3935758299 |
|  | 7 | 157291868 | 157292182 | PDFSTATSENSEMBLUCSC | 315 | 4 | 39104 | island | 0.269117 | 0.5820509 | Not queried | 1.74 | 3.43e-01 | 0.4672865596 |
|  | 14 | 23398313 | 23398654 | PDFSTATSENSEMBLUCSC | 342 | 5 | 12019 | gene | 0.000940 | 0.0040751 | Not queried | 1.74 | 1.51e-01 | 0.3618000441 |
|  | 15 | 25513277 | 25513654 | PDFSTATSENSEMBLUCSC | 378 | 4 | 13536 | tss | 0.370627 | 0.2322563 | Not queried | 1.74 | 6.86e-01 | 0.7223604052 |
|  | 17 | 29159410 | 29159713 | PDFSTATSENSEMBLUCSC | 304 | 4 | 16509 | gene;island | 0.000870 | 0.0022094 | Not queried | 1.74 | 4.86e-01 | 0.5640816327 |
|  | 1 | 40204285 | 40204847 | PDFSTATSENSEMBLUCSC | 563 | 6 | 3107 | island | 0.007233 | 0.0123840 | Not queried | 1.74 | 3.94e-01 | 0.5236526859 |
|  | 17 | 62018877 | 62019438 | PDFSTATSENSEMBLUCSC | 562 | 5 | 16730 | gene;island | 0.589905 | 0.8681906 | Not queried | 1.74 | 2.22e-01 | 0.3935758299 |
|  | 6 | 31510729 | 31511014 | PDFSTATSENSEMBLUCSC | 286 | 4 | 34812 | tss;island | 0.919339 | 0.9734423 | Not queried | 1.74 | 3.43e-01 | 0.4672865596 |
|  | 3 | 101546486 | 101546859 | PDFSTATSENSEMBLUCSC | 374 | 6 | 27883 | gene | 0.001327 | 0.0011716 | Not queried | 1.74 | 5.89e-01 | 0.6570484697 |
|  | 4 | 187621790 | 187622164 | PDFSTATSENSEMBLUCSC | 375 | 6 | 30218 | gene | 0.628168 | 0.8237763 | Not queried | 1.74 | 4.11e-02 | 0.2180389943 |
|  | 5 | 130330787 | 130331091 | PDFSTATSENSEMBLUCSC | 305 | 4 | 33076 | island | 0.280539 | 0.5918140 | Not queried | 1.74 | 4.86e-01 | 0.5640816327 |
|  | 6 | 32299502 | 32299676 | PDFSTATSENSEMBLUCSC | 175 | 4 | 34102 | gene | 0.749047 | 0.5423570 | Not queried | 1.73 | 3.43e-01 | 0.4672865596 |
|  | 3 | 50540137 | 50540261 | PDFSTATSENSEMBLUCSC | 125 | 4 | 27811 | gene;island | 0.010417 | 0.0532816 | Not queried | 1.73 | 1.14e-01 | 0.3123457953 |
|  | 14 | 101923155 | 101923702 | PDFSTATSENSEMBLUCSC | 548 | 7 | 13157 | island | 0.048938 | 0.0868360 | Not queried | 1.73 | 2.59e-01 | 0.4381244265 |
|  | 6 | 49681299 | 49681391 | PDFSTATSENSEMBLUCSC | 93 | 5 | 35027 | tss | 0.488188 | 0.2987567 | Not queried | 1.73 | 5.56e-02 | 0.2315877671 |
|  | 11 | 128760367 | 128760609 | PDFSTATSENSEMBLUCSC | 243 | 4 | 7806 | tss;island | 0.079791 | 0.0260460 | Not queried | 1.73 | 2.00e-01 | 0.3826488233 |
|  | 14 | 23589066 | 23589419 | PDFSTATSENSEMBLUCSC | 354 | 4 | 12297 | tss | 0.343921 | 0.8301865 | Not queried | 1.73 | 4.86e-01 | 0.5640816327 |
|  | 5 | 480033 | 480412 | PDFSTATSENSEMBLUCSC | 380 | 4 | 31506 | gene;island | 0.737933 | 0.8110294 | Not queried | 1.73 | 4.86e-01 | 0.5640816327 |
|  | 10 | 1451381 | 1451648 | PDFSTATSENSEMBLUCSC | 268 | 5 | 4464 | gene | 0.674036 | 0.8785177 | Not queried | 1.73 | 2.22e-01 | 0.3935758299 |
|  | 6 | 122793073 | 122793338 | PDFSTATSENSEMBLUCSC | 266 | 5 | 34462 | gene | 0.002256 | 0.0042148 | Not queried | 1.73 | 2.22e-01 | 0.3935758299 |
|  | 5 | 57878953 | 57879417 | PDFSTATSENSEMBLUCSC | 465 | 7 | 31623 | gene | 0.077395 | 0.0245619 | Not queried | 1.73 | 3.79e-02 | 0.2121545571 |
|  | 3 | 52812367 | 52812520 | PDFSTATSENSEMBLUCSC | 154 | 4 | 27825 | gene;tss | 0.367853 | 0.7088191 | Not queried | 1.73 | 2.00e-01 | 0.3826488233 |
|  | 5 | 140614369 | 140614765 | PDFSTATSENSEMBLUCSC | 397 | 4 | 31815 | gene;island | 0.017645 | 0.1027415 | Not queried | 1.73 | 6.86e-01 | 0.7223604052 |
|  | 17 | 17465162 | 17465476 | PDFSTATSENSEMBLUCSC | 315 | 5 | 16464 | gene;island | 0.480938 | 0.7422868 | Not queried | 1.73 | 2.22e-01 | 0.3935758299 |
|  | 13 | 112895431 | 112895794 | PDFSTATSENSEMBLUCSC | 364 | 5 | 11918 | island | 0.317027 | 0.6300337 | Not queried | 1.73 | 6.90e-01 | 0.7223604052 |
|  | 3 | 185826362 | 185826590 | PDFSTATSENSEMBLUCSC | 229 | 4 | 28071 | gene;island | 0.007523 | 0.0032581 | Not queried | 1.73 | 4.86e-01 | 0.5640816327 |
|  | 2 | 176987161 | 176987411 | PDFSTATSENSEMBLUCSC | 251 | 5 | 23737 | tss | 0.438248 | 0.1227829 | Not queried | 1.73 | 2.22e-01 | 0.3935758299 |
|  | 3 | 23244051 | 23244125 | PDFSTATSENSEMBLUCSC | 75 | 4 | 28183 | tss;island | 0.030586 | 0.1331221 | Not queried | 1.73 | 2.00e-01 | 0.3826488233 |
|  | 12 | 13197360 | 13197827 | PDFSTATSENSEMBLUCSC | 468 | 4 | 9063 | gene | 0.003882 | 0.0022492 | Not queried | 1.73 | 4.86e-01 | 0.5640816327 |
|  | 7 | 150413564 | 150413801 | PDFSTATSENSEMBLUCSC | 238 | 4 | 39012 | island | 0.015400 | 0.0482461 | Not queried | 1.73 | 2.86e-02 | 0.1830867741 |
|  | 11 | 67188491 | 67188871 | PDFSTATSENSEMBLUCSC | 381 | 5 | 6754 | gene;island | 0.002737 | 0.0025524 | Not queried | 1.73 | 6.90e-01 | 0.7223604052 |
|  | 12 | 49393027 | 49393408 | PDFSTATSENSEMBLUCSC | 382 | 8 | 10381 | island | 0.018932 | 0.0532970 | Not queried | 1.73 | 1.61e-01 | 0.3814043454 |
|  | 6 | 160783785 | 160784044 | PDFSTATSENSEMBLUCSC | 260 | 4 | 34535 | gene;island | 0.333180 | 0.5998887 | Not queried | 1.73 | 4.86e-01 | 0.5640816327 |
|  | 7 | 150974273 | 150974554 | PDFSTATSENSEMBLUCSC | 282 | 7 | 37999 | tss;island | 0.030855 | 0.0112445 | Not queried | 1.73 | 2.09e-01 | 0.3935758299 |
|  | 11 | 67203360 | 67203661 | PDFSTATSENSEMBLUCSC | 302 | 4 | 6755 | gene;island | 0.250840 | 0.4374744 | Not queried | 1.73 | 4.86e-01 | 0.5640816327 |
|  | 18 | 33877021 | 33877274 | PDFSTATSENSEMBLUCSC | 254 | 4 | 19244 | tss;island | 0.004255 | 0.0130927 | Not queried | 1.73 | 2.00e-01 | 0.3826488233 |
|  | 6 | 105584551 | 105584780 | PDFSTATSENSEMBLUCSC | 230 | 5 | 35145 | tss;island | 0.122871 | 0.0221338 | Not queried | 1.73 | 1.59e-02 | 0.1830867741 |
|  | 11 | 31845148 | 31845639 | PDFSTATSENSEMBLUCSC | 492 | 5 | 8168 | island | 0.035110 | 0.0044182 | Not queried | 1.73 | 5.48e-01 | 0.6179718485 |
|  | 7 | 102985332 | 102985587 | PDFSTATSENSEMBLUCSC | 256 | 4 | 37812 | tss | 0.496223 | 0.6764612 | Not queried | 1.73 | 4.86e-01 | 0.5640816327 |
|  | 8 | 42911287 | 42911648 | PDFSTATSENSEMBLUCSC | 362 | 7 | 40263 | island | 0.002224 | 0.0036584 | Not queried | 1.73 | 3.18e-01 | 0.4672865596 |
|  | 4 | 47034181 | 47034428 | PDFSTATSENSEMBLUCSC | 248 | 4 | 29996 | gene;island | 0.042973 | 0.1116331 | Not queried | 1.72 | 1.14e-01 | 0.3123457953 |
|  | 7 | 92463254 | 92463390 | PDFSTATSENSEMBLUCSC | 137 | 6 | 37710 | tss | 0.005400 | 0.0119505 | Not queried | 1.72 | 3.10e-01 | 0.4672865596 |
|  | 20 | 749264 | 749620 | PDFSTATSENSEMBLUCSC | 357 | 6 | 25529 | tss | 0.055311 | 0.0950342 | Not queried | 1.72 | 4.85e-01 | 0.5640816327 |
|  | 1 | 39546511 | 39546688 | PDFSTATSENSEMBLUCSC | 178 | 4 | 1520 | tss;island | 0.564070 | 0.3614233 | Not queried | 1.72 | 8.86e-01 | 0.8941250130 |
|  | 10 | 82116203 | 82116596 | PDFSTATSENSEMBLUCSC | 394 | 10 | 4654 | gene;tss;island | 0.038268 | 0.0686148 | Not queried | 1.72 | 2.18e-01 | 0.3935758299 |
|  | 11 | 1331124 | 1331497 | PDFSTATSENSEMBLUCSC | 374 | 4 | 6417 | gene;tss;island | 0.017254 | 0.0385344 | Not queried | 1.72 | 2.86e-02 | 0.1830867741 |
|  | 21 | 46424715 | 46424976 | PDFSTATSENSEMBLUCSC | 262 | 6 | 26591 | tss;island | 0.292517 | 0.6818980 | Not queried | 1.72 | 2.40e-01 | 0.4133265075 |
|  | 7 | 116164422 | 116164836 | PDFSTATSENSEMBLUCSC | 415 | 11 | 37850 | tss | 0.034177 | 0.0226336 | Not queried | 1.72 | 3.65e-01 | 0.4973948256 |
|  | 1 | 117529478 | 117530009 | PDFSTATSENSEMBLUCSC | 532 | 5 | 711 | gene | 0.680912 | 0.9573233 | Not queried | 1.72 | 1.00e+00 | 1.0000000000 |
|  | 22 | 44708761 | 44708908 | PDFSTATSENSEMBLUCSC | 148 | 5 | 27212 | tss | 0.202476 | 0.4086259 | Not queried | 1.72 | 4.21e-01 | 0.5404252807 |
|  | 11 | 31839314 | 31839947 | PDFSTATSENSEMBLUCSC | 634 | 9 | 8167 | island | 0.511753 | 0.1100228 | Not queried | 1.72 | 1.90e-01 | 0.3826488233 |
|  | 11 | 31839521 | 31839947 | PDFSTATSENSEMBLUCSC | 427 | 7 | 7224 | tss | 0.511753 | 0.1100228 | Not queried | 1.72 | 2.59e-01 | 0.4381244265 |
|  | 17 | 37856133 | 37856786 | PDFSTATSENSEMBLUCSC | 654 | 9 | 16553 | gene;island | 0.153808 | 0.0328318 | Not queried | 1.72 | 1.00e+00 | 1.0000000000 |
|  | 6 | 39902288 | 39902693 | PDFSTATSENSEMBLUCSC | 406 | 9 | 36113 | island | 0.095115 | 0.0148999 | Not queried | 1.72 | 3.40e-01 | 0.4672865596 |
|  | 6 | 30860866 | 30860960 | PDFSTATSENSEMBLUCSC | 95 | 4 | 33829 | gene | 0.918512 | 0.7731777 | Not queried | 1.72 | 2.00e-01 | 0.3826488233 |
|  | 8 | 11986813 | 11986933 | PDFSTATSENSEMBLUCSC | 121 | 4 | 39209 | gene;tss | 0.596724 | 0.8192435 | Not queried | 1.72 | 2.86e-02 | 0.1830867741 |
|  | 3 | 10334717 | 10334760 | PDFSTATSENSEMBLUCSC | 44 | 4 | 27659 | gene;tss | 0.036199 | 0.1064040 | Not queried | 1.72 | 2.00e-01 | 0.3826488233 |
|  | 19 | 57683687 | 57683885 | PDFSTATSENSEMBLUCSC | 199 | 4 | 22459 | island | 0.095000 | 0.0420101 | Not queried | 1.72 | 4.86e-01 | 0.5640816327 |
|  | 4 | 83483204 | 83483487 | PDFSTATSENSEMBLUCSC | 284 | 6 | 30467 | tss;island | 0.082663 | 0.0320432 | Not queried | 1.72 | 4.85e-01 | 0.5640816327 |
|  | 11 | 67432681 | 67433133 | PDFSTATSENSEMBLUCSC | 453 | 5 | 6763 | gene;island | 0.203495 | 0.5626587 | Not queried | 1.72 | 4.21e-01 | 0.5404252807 |
|  | 19 | 58095424 | 58095659 | PDFSTATSENSEMBLUCSC | 236 | 8 | 22475 | island | 0.078514 | 0.0134158 | Not queried | 1.72 | 6.50e-02 | 0.2477578846 |
|  | 1 | 47082691 | 47082869 | PDFSTATSENSEMBLUCSC | 179 | 5 | 1603 | tss;island | 0.053474 | 0.0486333 | Not queried | 1.72 | 5.48e-01 | 0.6179718485 |
|  | 1 | 60539559 | 60539675 | PDFSTATSENSEMBLUCSC | 117 | 4 | 1660 | tss | 0.047965 | 0.3499515 | Not queried | 1.72 | 1.14e-01 | 0.3123457953 |
|  | 16 | 86588418 | 86588622 | PDFSTATSENSEMBLUCSC | 205 | 4 | 14886 | gene;tss;island | 0.004291 | 0.0203149 | Not queried | 1.72 | 2.00e-01 | 0.3826488233 |
|  | 11 | 17743610 | 17743946 | PDFSTATSENSEMBLUCSC | 337 | 5 | 8104 | island | 0.032096 | 0.0999297 | Not queried | 1.72 | 1.00e+00 | 1.0000000000 |
|  | 5 | 41510742 | 41510851 | PDFSTATSENSEMBLUCSC | 110 | 4 | 32037 | tss | 0.171907 | 0.0554017 | Not queried | 1.72 | 4.86e-01 | 0.5640816327 |
|  | 5 | 174162510 | 174162907 | PDFSTATSENSEMBLUCSC | 398 | 4 | 33417 | island | 0.049606 | 0.0272298 | Not queried | 1.72 | 2.00e-01 | 0.3826488233 |
|  | 6 | 169224886 | 169225041 | PDFSTATSENSEMBLUCSC | 156 | 4 | 36667 | island | 0.580485 | 0.8297044 | Not queried | 1.72 | 3.43e-01 | 0.4672865596 |
|  | 6 | 143999465 | 143999715 | PDFSTATSENSEMBLUCSC | 251 | 4 | 34497 | gene;island | 0.050084 | 0.0072726 | Not queried | 1.72 | 4.86e-01 | 0.5640816327 |
|  | 18 | 12253749 | 12254690 | PDFSTATSENSEMBLUCSC | 942 | 19 | 19401 | island | 0.053031 | 0.1853589 | Not queried | 1.72 | 1.99e-03 | 0.0934794372 |
|  | 4 | 128554125 | 128554327 | PDFSTATSENSEMBLUCSC | 203 | 4 | 30124 | gene | 0.013269 | 0.0048268 | Not queried | 1.72 | 1.14e-01 | 0.3123457953 |
|  | 1 | 203456691 | 203457150 | PDFSTATSENSEMBLUCSC | 460 | 6 | 952 | gene;island | 0.004401 | 0.0182840 | Not queried | 1.72 | 2.40e-01 | 0.4133265075 |
|  | 1 | 3104999 | 3105326 | PDFSTATSENSEMBLUCSC | 328 | 5 | 107 | gene;island | 0.328052 | 0.5736146 | Not queried | 1.72 | 9.52e-02 | 0.3104222821 |
|  | 15 | 89312795 | 89313222 | PDFSTATSENSEMBLUCSC | 428 | 7 | 14421 | island | 0.071192 | 0.4384446 | Not queried | 1.71 | 9.73e-02 | 0.3123457953 |
|  | 5 | 497397 | 497639 | PDFSTATSENSEMBLUCSC | 243 | 4 | 31508 | gene;island | 0.268979 | 0.4297636 | Not queried | 1.71 | 2.86e-02 | 0.1830867741 |
|  | 1 | 47489282 | 47489543 | PDFSTATSENSEMBLUCSC | 262 | 4 | 514 | gene | 0.101010 | 0.0410871 | Not queried | 1.71 | 3.43e-01 | 0.4672865596 |
|  | 1 | 103574469 | 103574619 | PDFSTATSENSEMBLUCSC | 151 | 4 | 1785 | tss | 0.593408 | 0.3742547 | Not queried | 1.71 | 6.86e-01 | 0.7223604052 |
|  | 1 | 116380946 | 116381082 | PDFSTATSENSEMBLUCSC | 137 | 4 | 705 | gene;island | 0.154066 | 0.0367831 | Not queried | 1.71 | 3.43e-01 | 0.4672865596 |
|  | 2 | 10916238 | 10916392 | PDFSTATSENSEMBLUCSC | 155 | 4 | 22570 | gene | 0.722911 | 0.9591740 | Not queried | 1.71 | 4.86e-01 | 0.5640816327 |
|  | 11 | 86085623 | 86085757 | PDFSTATSENSEMBLUCSC | 135 | 4 | 7609 | tss | 0.161909 | 0.0540376 | Not queried | 1.71 | 8.86e-01 | 0.8941250130 |
|  | 5 | 1089507 | 1089800 | PDFSTATSENSEMBLUCSC | 294 | 6 | 31522 | gene;island | 0.933616 | 0.6746886 | Not queried | 1.71 | 2.40e-01 | 0.4133265075 |
|  | 7 | 157700872 | 157701262 | PDFSTATSENSEMBLUCSC | 391 | 5 | 37354 | gene;island | 0.269667 | 0.5465449 | Not queried | 1.71 | 9.52e-02 | 0.3104222821 |
|  | 10 | 134729942 | 134730258 | PDFSTATSENSEMBLUCSC | 317 | 5 | 6317 | island | 0.768864 | 0.4945439 | Not queried | 1.71 | 5.48e-01 | 0.6179718485 |
|  | 17 | 47091978 | 47092272 | PDFSTATSENSEMBLUCSC | 295 | 5 | 16670 | gene;island | 0.742150 | 0.4857384 | Not queried | 1.71 | 5.48e-01 | 0.6179718485 |
|  | 10 | 1095446 | 1095578 | PDFSTATSENSEMBLUCSC | 133 | 4 | 4913 | tss;island | 0.017587 | 0.0054466 | Not queried | 1.71 | 4.86e-01 | 0.5640816327 |
|  | 6 | 142409939 | 142410235 | PDFSTATSENSEMBLUCSC | 297 | 8 | 35260 | tss | 0.161760 | 0.0465878 | Not queried | 1.71 | 1.30e-01 | 0.3397986247 |
|  | 6 | 11232933 | 11233125 | PDFSTATSENSEMBLUCSC | 193 | 4 | 34636 | tss | 0.003450 | 0.0017765 | Not queried | 1.71 | 4.86e-01 | 0.5640816327 |
|  | 17 | 80708279 | 80708632 | PDFSTATSENSEMBLUCSC | 354 | 4 | 16956 | gene;island | 0.935662 | 0.9847000 | Not queried | 1.71 | 5.71e-02 | 0.2315877671 |
|  | 7 | 123673331 | 123673690 | PDFSTATSENSEMBLUCSC | 360 | 6 | 38879 | island | 0.078619 | 0.0428412 | Not queried | 1.71 | 5.89e-01 | 0.6570484697 |
|  | 7 | 123673549 | 123673690 | PDFSTATSENSEMBLUCSC | 142 | 4 | 37873 | tss | 0.077381 | 0.0723958 | Not queried | 1.71 | 8.86e-01 | 0.8941250130 |
|  | 1 | 161993364 | 161994363 | PDFSTATSENSEMBLUCSC | 1000 | 13 | 3915 | island | 0.042535 | 0.0100612 | Not queried | 1.71 | 1.25e-03 | 0.0862637156 |
|  | 17 | 79876118 | 79876287 | PDFSTATSENSEMBLUCSC | 170 | 5 | 17757 | tss | 0.010669 | 0.0031477 | Not queried | 1.70 | 5.48e-01 | 0.6179718485 |
|  | 10 | 103043991 | 103044385 | PDFSTATSENSEMBLUCSC | 395 | 5 | 6065 | island | 0.113561 | 0.0420769 | Not queried | 1.70 | 2.22e-01 | 0.3935758299 |
|  | 13 | 95655083 | 95655579 | PDFSTATSENSEMBLUCSC | 497 | 5 | 11824 | island | 0.150777 | 0.0167115 | Not queried | 1.70 | 5.48e-01 | 0.6179718485 |
|  | 2 | 218712695 | 218712871 | PDFSTATSENSEMBLUCSC | 177 | 4 | 23011 | gene | 0.400442 | 0.5060889 | Not queried | 1.70 | 3.43e-01 | 0.4672865596 |
|  | 11 | 2481857 | 2482594 | PDFSTATSENSEMBLUCSC | 738 | 15 | 7076 | tss | 0.159465 | 0.3971130 | Not queried | 1.70 | 1.16e-01 | 0.3167640946 |
|  | 6 | 100054469 | 100054615 | PDFSTATSENSEMBLUCSC | 147 | 6 | 35137 | tss | 0.485078 | 0.2796131 | Not queried | 1.70 | 3.94e-01 | 0.5236526859 |
|  | 6 | 112575850 | 112576119 | PDFSTATSENSEMBLUCSC | 270 | 5 | 35183 | tss | 0.204222 | 0.4897964 | Not queried | 1.70 | 9.52e-02 | 0.3104222821 |
|  | 7 | 109600399 | 109600733 | PDFSTATSENSEMBLUCSC | 335 | 4 | 37837 | tss | 0.134877 | 0.4096239 | Not queried | 1.70 | 4.86e-01 | 0.5640816327 |
|  | 6 | 33389072 | 33389172 | PDFSTATSENSEMBLUCSC | 101 | 4 | 34283 | gene;island | 0.659542 | 0.8723565 | Not queried | 1.70 | 3.43e-01 | 0.4672865596 |
|  | 8 | 134202337 | 134202370 | PDFSTATSENSEMBLUCSC | 34 | 4 | 39921 | tss | 0.638182 | 0.8561514 | Not queried | 1.70 | 3.43e-01 | 0.4672865596 |
|  | 4 | 20702045 | 20702377 | PDFSTATSENSEMBLUCSC | 333 | 4 | 29964 | gene | 0.004226 | 0.0014937 | Not queried | 1.70 | 4.86e-01 | 0.5640816327 |
|  | 3 | 39425775 | 39426101 | PDFSTATSENSEMBLUCSC | 327 | 4 | 27725 | gene;island | 0.009439 | 0.0187756 | Not queried | 1.70 | 8.86e-01 | 0.8941250130 |
|  | 19 | 50553682 | 50554067 | PDFSTATSENSEMBLUCSC | 386 | 7 | 22299 | island | 0.013263 | 0.0751992 | Not queried | 1.70 | 1.28e-01 | 0.3397986247 |
|  | 2 | 27373014 | 27373412 | PDFSTATSENSEMBLUCSC | 399 | 5 | 22621 | gene;island | 0.164548 | 0.7628913 | Not queried | 1.70 | 3.10e-01 | 0.4672865596 |
|  | 4 | 77355817 | 77356250 | PDFSTATSENSEMBLUCSC | 434 | 5 | 30449 | tss | 0.406627 | 0.5959410 | Not queried | 1.70 | 1.51e-01 | 0.3618000441 |
|  | 17 | 19314299 | 19314408 | PDFSTATSENSEMBLUCSC | 110 | 5 | 17191 | tss | 0.422713 | 0.7339812 | Not queried | 1.70 | 3.17e-02 | 0.1866936846 |
|  | 8 | 1814745 | 1815153 | PDFSTATSENSEMBLUCSC | 409 | 6 | 39180 | gene | 0.474644 | 0.6980425 | Not queried | 1.70 | 2.60e-02 | 0.1830867741 |
|  | 20 | 14318152 | 14318410 | PDFSTATSENSEMBLUCSC | 259 | 6 | 25362 | gene | 0.009958 | 0.0021732 | Not queried | 1.70 | 6.49e-02 | 0.2477578846 |
|  | 3 | 197463746 | 197464306 | PDFSTATSENSEMBLUCSC | 561 | 9 | 28106 | gene;island | 0.006316 | 0.0020195 | Not queried | 1.70 | 5.46e-01 | 0.6179718485 |
|  | 7 | 54609587 | 54610320 | PDFSTATSENSEMBLUCSC | 734 | 12 | 38498 | island | 0.022204 | 0.1106090 | Not queried | 1.70 | 1.28e-01 | 0.3397986247 |
|  | 21 | 46890849 | 46891093 | PDFSTATSENSEMBLUCSC | 245 | 4 | 26469 | gene;island | 0.438902 | 0.6920077 | Not queried | 1.70 | 3.43e-01 | 0.4672865596 |
|  | 19 | 11199851 | 11200146 | PDFSTATSENSEMBLUCSC | 296 | 7 | 21550 | island | 0.002034 | 0.0034037 | Not queried | 1.70 | 3.83e-01 | 0.5168893301 |
|  | 5 | 5303295 | 5303564 | PDFSTATSENSEMBLUCSC | 270 | 5 | 31553 | gene;island | 0.806112 | 0.5990253 | Not queried | 1.70 | 9.52e-02 | 0.3104222821 |
|  | 8 | 1364427 | 1364689 | PDFSTATSENSEMBLUCSC | 263 | 5 | 40023 | island | 0.554491 | 0.6840260 | Not queried | 1.70 | 9.52e-02 | 0.3104222821 |
|  | 10 | 1511024 | 1511277 | PDFSTATSENSEMBLUCSC | 254 | 4 | 4466 | gene | 0.323313 | 0.6060477 | Not queried | 1.70 | 6.86e-01 | 0.7223604052 |
|  | 15 | 65360366 | 65360486 | PDFSTATSENSEMBLUCSC | 121 | 4 | 14225 | island | 0.043904 | 0.2252410 | Not queried | 1.70 | 8.86e-01 | 0.8941250130 |
|  | 1 | 89357197 | 89357426 | PDFSTATSENSEMBLUCSC | 230 | 6 | 3446 | island | 0.002212 | 0.0023029 | Not queried | 1.70 | 5.89e-01 | 0.6570484697 |
|  | 6 | 32489553 | 32489991 | PDFSTATSENSEMBLUCSC | 439 | 6 | 34115 | gene;island | 0.893596 | 0.6980464 | Not queried | 1.70 | 3.94e-01 | 0.5236526859 |
|  | 14 | 101512592 | 101512826 | PDFSTATSENSEMBLUCSC | 235 | 5 | 12627 | tss | 0.355222 | 0.7770768 | Not queried | 1.70 | 2.22e-01 | 0.3935758299 |
|  | 19 | 1438017 | 1438306 | PDFSTATSENSEMBLUCSC | 290 | 10 | 20305 | tss | 0.017648 | 0.0924066 | Not queried | 1.70 | 1.23e-01 | 0.3348527312 |
|  | 5 | 140810051 | 140810137 | PDFSTATSENSEMBLUCSC | 87 | 5 | 32383 | tss | 0.013391 | 0.0041203 | Not queried | 1.70 | 2.22e-01 | 0.3935758299 |
|  | 17 | 80185943 | 80186336 | PDFSTATSENSEMBLUCSC | 394 | 6 | 17769 | tss;island | 0.014302 | 0.0495647 | Not queried | 1.70 | 5.89e-01 | 0.6570484697 |
|  | 7 | 1248693 | 1249199 | PDFSTATSENSEMBLUCSC | 507 | 7 | 38088 | island | 0.487127 | 0.7738135 | Not queried | 1.70 | 1.75e-02 | 0.1830867741 |
|  | 16 | 67876973 | 67877413 | PDFSTATSENSEMBLUCSC | 441 | 4 | 14803 | gene;island | 0.042795 | 0.0451095 | Not queried | 1.70 | 8.86e-01 | 0.8941250130 |
|  | 8 | 98290229 | 98290601 | PDFSTATSENSEMBLUCSC | 373 | 8 | 39829 | tss | 0.174629 | 0.0765677 | Not queried | 1.70 | 3.28e-01 | 0.4672865596 |
|  | 3 | 45838037 | 45838338 | PDFSTATSENSEMBLUCSC | 302 | 8 | 28271 | tss | 0.049079 | 0.0211950 | Not queried | 1.70 | 4.99e-02 | 0.2315877671 |
|  | 3 | 45837898 | 45838338 | PDFSTATSENSEMBLUCSC | 441 | 10 | 29051 | island | 0.056656 | 0.0277113 | Not queried | 1.70 | 8.92e-02 | 0.3104222821 |
|  | 8 | 109095782 | 109096151 | PDFSTATSENSEMBLUCSC | 370 | 8 | 40530 | island | 0.310175 | 0.1083795 | Not queried | 1.70 | 5.05e-01 | 0.5852592101 |
|  | 2 | 142889283 | 142889392 | PDFSTATSENSEMBLUCSC | 110 | 4 | 23653 | tss;island | 0.407117 | 0.7376485 | Not queried | 1.70 | 4.86e-01 | 0.5640816327 |
|  | 1 | 149871404 | 149871658 | PDFSTATSENSEMBLUCSC | 255 | 4 | 734 | gene;island | 0.028577 | 0.0835171 | Not queried | 1.70 | 3.43e-01 | 0.4672865596 |
|  | 17 | 42422247 | 42422442 | PDFSTATSENSEMBLUCSC | 196 | 6 | 17409 | tss | 0.134374 | 0.0191112 | Not queried | 1.70 | 3.10e-01 | 0.4672865596 |
|  | 10 | 3823758 | 3824016 | PDFSTATSENSEMBLUCSC | 259 | 5 | 4475 | gene;island | 0.494849 | 0.5648704 | Not queried | 1.70 | 2.22e-01 | 0.3935758299 |
|  | 19 | 12868527 | 12868938 | PDFSTATSENSEMBLUCSC | 412 | 5 | 19865 | gene;island | 0.040217 | 0.0127706 | Not queried | 1.70 | 6.90e-01 | 0.7223604052 |
|  | 2 | 233320486 | 233320796 | PDFSTATSENSEMBLUCSC | 311 | 4 | 23949 | tss | 0.047108 | 0.1787090 | Not queried | 1.70 | 2.00e-01 | 0.3826488233 |
|  | 7 | 56516076 | 56516504 | PDFSTATSENSEMBLUCSC | 429 | 9 | 37611 | tss;island | 0.508585 | 0.2174767 | Not queried | 1.69 | 4.00e-02 | 0.2180389943 |
|  | 10 | 88391658 | 88392088 | PDFSTATSENSEMBLUCSC | 431 | 4 | 5907 | island | 0.333510 | 0.1284515 | Not queried | 1.69 | 3.43e-01 | 0.4672865596 |
|  | 7 | 158220708 | 158221076 | PDFSTATSENSEMBLUCSC | 369 | 5 | 37370 | gene;island | 0.469444 | 0.7410748 | Not queried | 1.69 | 3.10e-01 | 0.4672865596 |
|  | 4 | 185938720 | 185938989 | PDFSTATSENSEMBLUCSC | 270 | 4 | 30698 | tss;island | 0.015349 | 0.0086446 | Not queried | 1.69 | 8.86e-01 | 0.8941250130 |
|  | 8 | 121824330 | 121824929 | PDFSTATSENSEMBLUCSC | 600 | 9 | 39891 | tss | 0.004932 | 0.0077509 | Not queried | 1.69 | 2.97e-01 | 0.4672865596 |
|  | 19 | 58459097 | 58459358 | PDFSTATSENSEMBLUCSC | 262 | 8 | 21130 | tss | 0.023160 | 0.0070086 | Not queried | 1.69 | 2.81e-02 | 0.1830867741 |
|  | 6 | 43211208 | 43211491 | PDFSTATSENSEMBLUCSC | 284 | 5 | 36158 | island | 0.697656 | 0.3658020 | Not queried | 1.69 | 3.10e-01 | 0.4672865596 |
|  | 2 | 214016774 | 214017273 | PDFSTATSENSEMBLUCSC | 500 | 4 | 23858 | tss;island | 0.009067 | 0.0196127 | Not queried | 1.69 | 2.00e-01 | 0.3826488233 |
|  | 3 | 48471575 | 48471901 | PDFSTATSENSEMBLUCSC | 327 | 5 | 28297 | tss;island | 0.542671 | 0.4882588 | Not queried | 1.69 | 4.21e-01 | 0.5404252807 |
|  | 7 | 4911022 | 4911224 | PDFSTATSENSEMBLUCSC | 203 | 4 | 36863 | gene | 0.209745 | 0.4387077 | Not queried | 1.69 | 4.86e-01 | 0.5640816327 |
|  | 13 | 113637695 | 113637903 | PDFSTATSENSEMBLUCSC | 209 | 4 | 11264 | gene;island | 0.403644 | 0.7215916 | Not queried | 1.69 | 2.86e-02 | 0.1830867741 |
|  | 1 | 111813509 | 111813852 | PDFSTATSENSEMBLUCSC | 344 | 4 | 3590 | island | 0.119226 | 0.4333552 | Not queried | 1.69 | 5.71e-02 | 0.2315877671 |
|  | 7 | 27224092 | 27224795 | PDFSTATSENSEMBLUCSC | 704 | 8 | 36941 | gene | 0.024501 | 0.0435094 | Not queried | 1.69 | 5.74e-01 | 0.6460388787 |
|  | 4 | 146403866 | 146403948 | PDFSTATSENSEMBLUCSC | 83 | 5 | 30620 | tss | 0.054237 | 0.0014999 | Not queried | 1.69 | 2.22e-01 | 0.3935758299 |
|  | 12 | 54785171 | 54785320 | PDFSTATSENSEMBLUCSC | 150 | 4 | 9763 | tss | 0.036611 | 0.0067099 | Not queried | 1.69 | 3.43e-01 | 0.4672865596 |
|  | 2 | 20189711 | 20190349 | PDFSTATSENSEMBLUCSC | 639 | 11 | 24106 | island | 0.073096 | 0.0251274 | Not queried | 1.69 | 1.33e-01 | 0.3420544733 |
|  | 19 | 37959744 | 37960107 | PDFSTATSENSEMBLUCSC | 364 | 8 | 21956 | island | 0.021560 | 0.0092172 | Not queried | 1.69 | 3.79e-02 | 0.2121545571 |
|  | 2 | 3063115 | 3063671 | PDFSTATSENSEMBLUCSC | 557 | 9 | 24025 | island | 0.524099 | 0.8205761 | Not queried | 1.69 | 2.58e-01 | 0.4381244265 |
|  | 12 | 53496842 | 53497147 | PDFSTATSENSEMBLUCSC | 306 | 5 | 9733 | tss | 0.025601 | 0.1481273 | Not queried | 1.69 | 8.41e-01 | 0.8635565145 |
|  | 5 | 140734543 | 140734758 | PDFSTATSENSEMBLUCSC | 216 | 4 | 32375 | tss | 0.167934 | 0.4279464 | Not queried | 1.69 | 4.86e-01 | 0.5640816327 |
|  | 19 | 39694993 | 39695237 | PDFSTATSENSEMBLUCSC | 245 | 5 | 20767 | tss | 0.466815 | 0.7383369 | Not queried | 1.69 | 3.10e-01 | 0.4672865596 |
|  | 5 | 175200401 | 175200576 | PDFSTATSENSEMBLUCSC | 176 | 4 | 33420 | island | 0.095791 | 0.0339945 | Not queried | 1.69 | 2.00e-01 | 0.3826488233 |
|  | 7 | 128784416 | 128784656 | PDFSTATSENSEMBLUCSC | 241 | 6 | 37890 | tss | 0.009356 | 0.0248105 | Not queried | 1.69 | 2.60e-02 | 0.1830867741 |
|  | 8 | 63776508 | 63776762 | PDFSTATSENSEMBLUCSC | 255 | 4 | 39319 | gene | 0.736104 | 0.5185909 | Not queried | 1.69 | 4.86e-01 | 0.5640816327 |
|  | 3 | 171175713 | 171176016 | PDFSTATSENSEMBLUCSC | 304 | 4 | 28031 | gene;island | 0.045041 | 0.1765733 | Not queried | 1.69 | 8.86e-01 | 0.8941250130 |
|  | 2 | 217363243 | 217363465 | PDFSTATSENSEMBLUCSC | 223 | 5 | 23869 | tss | 0.002887 | 0.0018564 | Not queried | 1.69 | 4.21e-01 | 0.5404252807 |
|  | 2 | 63277672 | 63277944 | PDFSTATSENSEMBLUCSC | 273 | 11 | 23387 | tss | 0.005495 | 0.0027708 | Not queried | 1.69 | 1.16e-01 | 0.3175184142 |
|  | 2 | 242751796 | 242752080 | PDFSTATSENSEMBLUCSC | 285 | 4 | 23146 | gene;island | 0.376664 | 0.6077898 | Not queried | 1.69 | 2.00e-01 | 0.3826488233 |
|  | 22 | 19710880 | 19711051 | PDFSTATSENSEMBLUCSC | 172 | 6 | 26788 | gene;tss;island | 0.007788 | 0.0097924 | Not queried | 1.69 | 6.99e-01 | 0.7290267580 |
|  | 2 | 190445477 | 190446181 | PDFSTATSENSEMBLUCSC | 705 | 7 | 24955 | island | 0.016070 | 0.0516899 | Not queried | 1.69 | 2.59e-01 | 0.4381244265 |
|  | 21 | 17961737 | 17962124 | PDFSTATSENSEMBLUCSC | 388 | 4 | 26388 | gene;tss | 0.441118 | 0.7442956 | Not queried | 1.68 | 3.43e-01 | 0.4672865596 |
|  | 4 | 96470887 | 96471143 | PDFSTATSENSEMBLUCSC | 257 | 4 | 30506 | tss;island | 0.007298 | 0.0164219 | Not queried | 1.68 | 5.71e-02 | 0.2315877671 |
|  | 8 | 144790187 | 144790410 | PDFSTATSENSEMBLUCSC | 224 | 5 | 40681 | island | 0.582268 | 0.8182489 | Not queried | 1.68 | 8.41e-01 | 0.8635565145 |
|  | 17 | 14206774 | 14207036 | PDFSTATSENSEMBLUCSC | 263 | 7 | 16450 | gene;tss;island | 0.015863 | 0.0687744 | Not queried | 1.68 | 6.20e-01 | 0.6899391537 |
|  | 7 | 76026853 | 76027101 | PDFSTATSENSEMBLUCSC | 249 | 4 | 37056 | gene;island | 0.024130 | 0.0096519 | Not queried | 1.68 | 3.43e-01 | 0.4672865596 |
|  | 3 | 140769610 | 140770013 | PDFSTATSENSEMBLUCSC | 404 | 4 | 28636 | tss;island | 0.039906 | 0.0588921 | Not queried | 1.68 | 4.86e-01 | 0.5640816327 |
|  | 6 | 29407834 | 29408666 | PDFSTATSENSEMBLUCSC | 833 | 13 | 33656 | gene | 0.184880 | 0.6001960 | Not queried | 1.68 | 8.11e-02 | 0.2974420553 |
|  | 15 | 27019248 | 27019448 | PDFSTATSENSEMBLUCSC | 201 | 4 | 13541 | tss;island | 0.018179 | 0.0469065 | Not queried | 1.68 | 1.14e-01 | 0.3123457953 |
|  | 11 | 46414401 | 46414626 | PDFSTATSENSEMBLUCSC | 226 | 4 | 8251 | island | 0.010284 | 0.0050767 | Not queried | 1.68 | 1.14e-01 | 0.3123457953 |
|  | 10 | 120925308 | 120925632 | PDFSTATSENSEMBLUCSC | 325 | 7 | 5399 | tss | 0.004929 | 0.0074475 | Not queried | 1.68 | 8.05e-01 | 0.8347790563 |
|  | 1 | 53608037 | 53608280 | PDFSTATSENSEMBLUCSC | 244 | 4 | 537 | gene | 0.375922 | 0.5857823 | Not queried | 1.68 | 4.86e-01 | 0.5640816327 |
|  | 15 | 55880894 | 55881246 | PDFSTATSENSEMBLUCSC | 353 | 9 | 14160 | island | 0.182576 | 0.0594019 | Not queried | 1.68 | 2.97e-01 | 0.4672865596 |
|  | 1 | 53392492 | 53392881 | PDFSTATSENSEMBLUCSC | 390 | 6 | 1634 | tss | 0.035837 | 0.3646636 | Not queried | 1.68 | 8.18e-01 | 0.8469867211 |
|  | 7 | 28319343 | 28319676 | PDFSTATSENSEMBLUCSC | 334 | 6 | 37504 | tss | 0.518468 | 0.6638618 | Not queried | 1.68 | 2.40e-01 | 0.4133265075 |
|  | 4 | 187517794 | 187518154 | PDFSTATSENSEMBLUCSC | 361 | 5 | 30217 | gene | 0.739574 | 0.9490570 | Not queried | 1.68 | 5.56e-02 | 0.2315877671 |
|  | 1 | 3605747 | 3606082 | PDFSTATSENSEMBLUCSC | 336 | 5 | 136 | gene;tss;island | 0.725669 | 0.3306354 | Not queried | 1.68 | 5.56e-02 | 0.2315877671 |
|  | 6 | 33144795 | 33145182 | PDFSTATSENSEMBLUCSC | 388 | 5 | 34205 | gene | 0.365399 | 0.7579557 | Not queried | 1.68 | 4.21e-01 | 0.5404252807 |
|  | 11 | 22214333 | 22214726 | PDFSTATSENSEMBLUCSC | 394 | 6 | 8132 | island | 0.011745 | 0.0150928 | Not queried | 1.68 | 3.94e-01 | 0.5236526859 |
|  | 5 | 73980834 | 73980966 | PDFSTATSENSEMBLUCSC | 133 | 5 | 32125 | tss | 0.005882 | 0.0039400 | Not queried | 1.68 | 6.90e-01 | 0.7223604052 |
|  | 19 | 49198900 | 49199234 | PDFSTATSENSEMBLUCSC | 335 | 5 | 22230 | island | 0.182484 | 0.4165967 | Not queried | 1.68 | 2.22e-01 | 0.3935758299 |
|  | 6 | 31845413 | 31845879 | PDFSTATSENSEMBLUCSC | 467 | 6 | 33979 | gene | 0.520646 | 0.6657655 | Not queried | 1.68 | 1.80e-01 | 0.3826488233 |
|  | 1 | 32827707 | 32828191 | PDFSTATSENSEMBLUCSC | 485 | 9 | 395 | gene;tss;island | 0.058992 | 0.1761777 | Not queried | 1.68 | 9.39e-02 | 0.3104222821 |
|  | 16 | 751312 | 751603 | PDFSTATSENSEMBLUCSC | 292 | 4 | 14540 | gene;island | 0.029343 | 0.0961720 | Not queried | 1.68 | 2.00e-01 | 0.3826488233 |
|  | 11 | 1855746 | 1856307 | PDFSTATSENSEMBLUCSC | 562 | 7 | 6432 | gene | 0.170993 | 0.3397184 | Not queried | 1.68 | 5.30e-02 | 0.2315877671 |
|  | 14 | 21493964 | 21494269 | PDFSTATSENSEMBLUCSC | 306 | 6 | 12280 | tss;island | 0.482364 | 0.0849839 | Not queried | 1.68 | 4.85e-01 | 0.5640816327 |
|  | 20 | 30795553 | 30795610 | PDFSTATSENSEMBLUCSC | 58 | 6 | 25673 | tss;island | 0.000803 | 0.0024303 | Not queried | 1.68 | 4.85e-01 | 0.5640816327 |
|  | 5 | 134181484 | 134181707 | PDFSTATSENSEMBLUCSC | 224 | 7 | 31752 | gene;island | 0.003010 | 0.0029341 | Not queried | 1.68 | 7.10e-01 | 0.7390730975 |
|  | 16 | 960911 | 961353 | PDFSTATSENSEMBLUCSC | 443 | 4 | 14551 | gene;island | 0.498968 | 0.8449413 | Not queried | 1.68 | 5.71e-02 | 0.2315877671 |
|  | 16 | 783597 | 783916 | PDFSTATSENSEMBLUCSC | 320 | 4 | 14543 | gene;island | 0.657425 | 0.8308091 | Not queried | 1.67 | 4.86e-01 | 0.5640816327 |
|  | 1 | 228112806 | 228113207 | PDFSTATSENSEMBLUCSC | 402 | 6 | 1044 | gene;island | 0.423476 | 0.4505143 | Not queried | 1.67 | 4.85e-01 | 0.5640816327 |
|  | 7 | 4243286 | 4243734 | PDFSTATSENSEMBLUCSC | 449 | 5 | 36834 | gene | 0.783010 | 0.6980930 | Not queried | 1.67 | 8.41e-01 | 0.8635565145 |
|  | 5 | 140220686 | 140220822 | PDFSTATSENSEMBLUCSC | 137 | 4 | 32354 | tss | 0.814154 | 0.6634196 | Not queried | 1.67 | 2.86e-02 | 0.1830867741 |
|  | 6 | 30080189 | 30080782 | PDFSTATSENSEMBLUCSC | 594 | 10 | 33725 | gene | 0.499614 | 0.8781150 | Not queried | 1.67 | 1.05e-01 | 0.3123457953 |
|  | 2 | 162094696 | 162094867 | PDFSTATSENSEMBLUCSC | 172 | 4 | 24819 | island | 0.012246 | 0.0039023 | Not queried | 1.67 | 3.43e-01 | 0.4672865596 |
|  | 11 | 2950725 | 2950754 | PDFSTATSENSEMBLUCSC | 30 | 4 | 7087 | tss | 0.012418 | 0.0095879 | Not queried | 1.67 | 4.86e-01 | 0.5640816327 |
|  | 3 | 158519410 | 158519645 | PDFSTATSENSEMBLUCSC | 236 | 6 | 28688 | tss | 0.023381 | 0.0110608 | Not queried | 1.67 | 4.85e-01 | 0.5640816327 |
|  | 6 | 29588894 | 29589056 | PDFSTATSENSEMBLUCSC | 163 | 4 | 33674 | gene | 0.295838 | 0.4586713 | Not queried | 1.67 | 2.86e-02 | 0.1830867741 |
|  | 20 | 61162000 | 61162112 | PDFSTATSENSEMBLUCSC | 113 | 6 | 25877 | tss | 0.250623 | 0.4740069 | Not queried | 1.67 | 2.40e-01 | 0.4133265075 |
|  | 19 | 1401118 | 1401781 | PDFSTATSENSEMBLUCSC | 664 | 9 | 21222 | island | 0.010237 | 0.0158888 | Not queried | 1.67 | 7.96e-01 | 0.8275104336 |
|  | 3 | 158390510 | 158390821 | PDFSTATSENSEMBLUCSC | 312 | 7 | 28686 | tss | 0.032922 | 0.2825048 | Not queried | 1.67 | 1.28e-01 | 0.3397986247 |
|  | 6 | 13274151 | 13274354 | PDFSTATSENSEMBLUCSC | 204 | 5 | 33575 | gene | 0.003993 | 0.0197653 | Not queried | 1.67 | 9.52e-02 | 0.3104222821 |
|  | 15 | 74494781 | 74494909 | PDFSTATSENSEMBLUCSC | 129 | 5 | 13420 | gene | 0.392872 | 0.6675646 | Not queried | 1.67 | 2.22e-01 | 0.3935758299 |
|  | 4 | 81124484 | 81124767 | PDFSTATSENSEMBLUCSC | 284 | 4 | 30035 | gene;island | 0.037372 | 0.1415738 | Not queried | 1.67 | 2.00e-01 | 0.3826488233 |
|  | 7 | 156811252 | 156811655 | PDFSTATSENSEMBLUCSC | 404 | 6 | 39093 | island | 0.186143 | 0.0227578 | Not queried | 1.67 | 3.10e-01 | 0.4672865596 |
|  | 1 | 153643319 | 153643822 | PDFSTATSENSEMBLUCSC | 504 | 9 | 3766 | island | 0.006951 | 0.0087503 | Not queried | 1.67 | 7.96e-01 | 0.8275104336 |
|  | 5 | 1257034 | 1257221 | PDFSTATSENSEMBLUCSC | 188 | 5 | 31530 | gene;island | 0.793292 | 0.5466133 | Not queried | 1.67 | 4.21e-01 | 0.5404252807 |
|  | 15 | 45315362 | 45315438 | PDFSTATSENSEMBLUCSC | 77 | 4 | 13325 | gene | 0.004452 | 0.0030882 | Not queried | 1.67 | 3.43e-01 | 0.4672865596 |
|  | 1 | 223899685 | 223899998 | PDFSTATSENSEMBLUCSC | 314 | 7 | 1025 | gene;tss;island | 0.482488 | 0.4827912 | Not queried | 1.66 | 8.05e-01 | 0.8347790563 |
|  | 17 | 21356245 | 21356372 | PDFSTATSENSEMBLUCSC | 128 | 4 | 18121 | island | 0.270824 | 0.5413516 | Not queried | 1.66 | 2.00e-01 | 0.3826488233 |
|  | 7 | 19183563 | 19183880 | PDFSTATSENSEMBLUCSC | 318 | 4 | 38285 | island | 0.006425 | 0.0126180 | Not queried | 1.66 | 3.43e-01 | 0.4672865596 |
|  | 11 | 1221398 | 1221812 | PDFSTATSENSEMBLUCSC | 415 | 4 | 7906 | island | 0.608778 | 0.7938220 | Not queried | 1.66 | 6.86e-01 | 0.7223604052 |
|  | 15 | 79103007 | 79103221 | PDFSTATSENSEMBLUCSC | 215 | 4 | 13441 | gene;island | 0.025552 | 0.0133088 | Not queried | 1.66 | 3.43e-01 | 0.4672865596 |
|  | 19 | 1650224 | 1650452 | PDFSTATSENSEMBLUCSC | 229 | 4 | 19660 | gene;island | 0.498450 | 0.7138461 | Not queried | 1.66 | 4.86e-01 | 0.5640816327 |
|  | 13 | 33780078 | 33780362 | PDFSTATSENSEMBLUCSC | 285 | 5 | 11125 | gene | 0.014214 | 0.0086548 | Not queried | 1.66 | 3.10e-01 | 0.4672865596 |
|  | 1 | 17231486 | 17231793 | PDFSTATSENSEMBLUCSC | 308 | 5 | 2750 | island | 0.004369 | 0.0034223 | Not queried | 1.66 | 8.41e-01 | 0.8635565145 |
|  | 17 | 48785310 | 48785602 | PDFSTATSENSEMBLUCSC | 293 | 5 | 17505 | tss | 0.021007 | 0.0078438 | Not queried | 1.66 | 4.21e-01 | 0.5404252807 |
|  | 5 | 140551968 | 140552163 | PDFSTATSENSEMBLUCSC | 196 | 4 | 32364 | tss | 0.040504 | 0.0426696 | Not queried | 1.66 | 6.86e-01 | 0.7223604052 |
|  | 11 | 66314211 | 66314378 | PDFSTATSENSEMBLUCSC | 168 | 5 | 7473 | tss;island | 0.036790 | 0.0084230 | Not queried | 1.66 | 5.48e-01 | 0.6179718485 |
|  | 11 | 20181725 | 20182122 | PDFSTATSENSEMBLUCSC | 398 | 8 | 8124 | island | 0.185624 | 0.3204307 | Not queried | 1.66 | 1.00e+00 | 1.0000000000 |
|  | 2 | 176988203 | 176988580 | PDFSTATSENSEMBLUCSC | 378 | 5 | 22937 | gene;island | 0.054867 | 0.6988130 | Not queried | 1.66 | 2.22e-01 | 0.3935758299 |
|  | 6 | 31545252 | 31545473 | PDFSTATSENSEMBLUCSC | 222 | 5 | 33903 | gene;island | 0.412472 | 0.6701955 | Not queried | 1.66 | 7.94e-03 | 0.1589602024 |
|  | 10 | 88024553 | 88024673 | PDFSTATSENSEMBLUCSC | 121 | 6 | 5196 | tss | 0.111615 | 0.3737576 | Not queried | 1.66 | 3.94e-01 | 0.5236526859 |
|  | 1 | 3824232 | 3824447 | PDFSTATSENSEMBLUCSC | 216 | 4 | 151 | gene;island | 0.809980 | 0.9650583 | Not queried | 1.66 | 2.00e-01 | 0.3826488233 |
|  | 3 | 120460879 | 120461349 | PDFSTATSENSEMBLUCSC | 471 | 5 | 27910 | gene | 0.002682 | 0.0010189 | Not queried | 1.66 | 5.48e-01 | 0.6179718485 |
|  | 6 | 160679391 | 160680213 | PDFSTATSENSEMBLUCSC | 823 | 12 | 36612 | island | 0.503102 | 0.6917235 | Not queried | 1.66 | 6.84e-02 | 0.2600243423 |
|  | 11 | 67803771 | 67804112 | PDFSTATSENSEMBLUCSC | 342 | 4 | 6767 | gene;island | 0.753358 | 0.9514486 | Not queried | 1.66 | 6.86e-01 | 0.7223604052 |
|  | 7 | 1277521 | 1277790 | PDFSTATSENSEMBLUCSC | 270 | 4 | 38092 | island | 0.292693 | 0.2282559 | Not queried | 1.66 | 1.00e+00 | 1.0000000000 |
|  | 19 | 5507274 | 5507540 | PDFSTATSENSEMBLUCSC | 267 | 5 | 21405 | island | 0.700655 | 0.9439743 | Not queried | 1.66 | 9.52e-02 | 0.3104222821 |
|  | 10 | 135061171 | 135061360 | PDFSTATSENSEMBLUCSC | 190 | 6 | 5457 | tss | 0.583522 | 0.6760661 | Not queried | 1.66 | 1.80e-01 | 0.3826488233 |
|  | 19 | 46932069 | 46932308 | PDFSTATSENSEMBLUCSC | 240 | 4 | 22172 | island | 0.116569 | 0.0253889 | Not queried | 1.66 | 2.00e-01 | 0.3826488233 |
|  | 8 | 61193715 | 61194292 | PDFSTATSENSEMBLUCSC | 578 | 11 | 40329 | island | 0.059467 | 0.0153849 | Not queried | 1.66 | 3.36e-02 | 0.1970167151 |
|  | 20 | 61870691 | 61871067 | PDFSTATSENSEMBLUCSC | 377 | 4 | 25498 | gene;island | 0.665260 | 0.8892425 | Not queried | 1.66 | 2.00e-01 | 0.3826488233 |
|  | 5 | 176778888 | 176779229 | PDFSTATSENSEMBLUCSC | 342 | 9 | 32524 | tss | 0.001469 | 0.0037723 | Not queried | 1.66 | 4.36e-01 | 0.5589686471 |
|  | 8 | 24771114 | 24771645 | PDFSTATSENSEMBLUCSC | 532 | 7 | 39604 | tss;island | 0.299202 | 0.0862646 | Not queried | 1.66 | 6.99e-03 | 0.1589602024 |
|  | 7 | 25893749 | 25894005 | PDFSTATSENSEMBLUCSC | 257 | 4 | 38323 | island | 0.054444 | 0.2865402 | Not queried | 1.66 | 4.86e-01 | 0.5640816327 |
|  | 14 | 70233599 | 70233819 | PDFSTATSENSEMBLUCSC | 221 | 8 | 12483 | tss | 0.005621 | 0.0093011 | Not queried | 1.66 | 1.61e-01 | 0.3814043454 |
|  | 6 | 30955358 | 30955425 | PDFSTATSENSEMBLUCSC | 68 | 5 | 33846 | gene | 0.046663 | 0.1393236 | Not queried | 1.66 | 3.10e-01 | 0.4672865596 |
|  | 20 | 55107833 | 55108228 | PDFSTATSENSEMBLUCSC | 396 | 7 | 25838 | tss | 0.597362 | 0.8263823 | Not queried | 1.66 | 2.59e-01 | 0.4381244265 |
|  | 3 | 126707103 | 126707501 | PDFSTATSENSEMBLUCSC | 399 | 4 | 29438 | island | 0.647708 | 0.8648309 | Not queried | 1.66 | 4.86e-01 | 0.5640816327 |
|  | 18 | 31803454 | 31803836 | PDFSTATSENSEMBLUCSC | 383 | 6 | 19228 | tss;island | 0.015265 | 0.0620067 | Not queried | 1.65 | 3.10e-01 | 0.4672865596 |
|  | 16 | 67219152 | 67219218 | PDFSTATSENSEMBLUCSC | 67 | 4 | 14792 | gene;tss;island | 0.478653 | 0.3030164 | Not queried | 1.65 | 4.86e-01 | 0.5640816327 |
|  | 20 | 61810348 | 61810902 | PDFSTATSENSEMBLUCSC | 555 | 5 | 26344 | island | 0.037364 | 0.0131455 | Not queried | 1.65 | 5.48e-01 | 0.6179718485 |
|  | 1 | 713921 | 714177 | PDFSTATSENSEMBLUCSC | 257 | 6 | 2376 | island | 0.013496 | 0.0097380 | Not queried | 1.65 | 4.85e-01 | 0.5640816327 |
|  | 1 | 3623319 | 3623605 | PDFSTATSENSEMBLUCSC | 287 | 4 | 141 | gene;island | 0.948857 | 0.8790048 | Not queried | 1.65 | 2.00e-01 | 0.3826488233 |
|  | 5 | 170740547 | 170740937 | PDFSTATSENSEMBLUCSC | 391 | 6 | 33380 | island | 0.199828 | 0.4517629 | Not queried | 1.65 | 6.49e-02 | 0.2477578846 |
|  | 11 | 31819148 | 31819464 | PDFSTATSENSEMBLUCSC | 317 | 6 | 6555 | gene;island | 0.425898 | 0.1290673 | Not queried | 1.65 | 6.49e-02 | 0.2477578846 |
|  | 14 | 106938234 | 106938621 | PDFSTATSENSEMBLUCSC | 388 | 10 | 13248 | island | 0.873004 | 0.9687642 | Not queried | 1.65 | 5.24e-02 | 0.2315877671 |
|  | 4 | 80247171 | 80247281 | PDFSTATSENSEMBLUCSC | 111 | 5 | 30456 | tss | 0.825114 | 0.8698521 | Not queried | 1.65 | 5.48e-01 | 0.6179718485 |
|  | 14 | 75642944 | 75643321 | PDFSTATSENSEMBLUCSC | 378 | 4 | 12145 | gene | 0.009359 | 0.0022373 | Not queried | 1.65 | 3.43e-01 | 0.4672865596 |
|  | 16 | 928778 | 929101 | PDFSTATSENSEMBLUCSC | 324 | 4 | 14549 | gene;island | 0.717464 | 0.7674792 | Not queried | 1.65 | 6.86e-01 | 0.7223604052 |
|  | 3 | 135969387 | 135969981 | PDFSTATSENSEMBLUCSC | 595 | 7 | 27959 | gene;island | 0.031146 | 0.0057009 | Not queried | 1.65 | 7.28e-02 | 0.2713481487 |
|  | 6 | 28317687 | 28317796 | PDFSTATSENSEMBLUCSC | 110 | 4 | 33631 | gene | 0.009072 | 0.0046274 | Not queried | 1.65 | 6.86e-01 | 0.7223604052 |
|  | 17 | 7348316 | 7348399 | PDFSTATSENSEMBLUCSC | 84 | 6 | 17084 | tss | 0.008871 | 0.0167352 | Not queried | 1.65 | 3.10e-01 | 0.4672865596 |
|  | 4 | 55095148 | 55095529 | PDFSTATSENSEMBLUCSC | 382 | 8 | 31055 | island | 0.049378 | 0.0099997 | Not queried | 1.65 | 1.30e-01 | 0.3397986247 |
|  | 4 | 81256552 | 81256850 | PDFSTATSENSEMBLUCSC | 299 | 7 | 30462 | tss | 0.006820 | 0.0017050 | Not queried | 1.65 | 6.99e-03 | 0.1589602024 |
|  | 12 | 117176113 | 117176563 | PDFSTATSENSEMBLUCSC | 451 | 6 | 9329 | gene | 0.005023 | 0.0073080 | Not queried | 1.65 | 3.94e-01 | 0.5236526859 |
|  | 7 | 16460779 | 16461244 | PDFSTATSENSEMBLUCSC | 466 | 6 | 38274 | island | 0.239927 | 0.0303239 | Not queried | 1.65 | 3.10e-01 | 0.4672865596 |
|  | 6 | 30882641 | 30882708 | PDFSTATSENSEMBLUCSC | 68 | 4 | 33837 | gene;island | 0.109780 | 0.1702758 | Not queried | 1.65 | 4.86e-01 | 0.5640816327 |
|  | 22 | 30901532 | 30901886 | PDFSTATSENSEMBLUCSC | 355 | 7 | 27413 | island | 0.100078 | 0.5519746 | Not queried | 1.65 | 9.73e-02 | 0.3123457953 |
|  | 1 | 230777685 | 230777875 | PDFSTATSENSEMBLUCSC | 191 | 4 | 2316 | tss;island | 0.154502 | 0.0204816 | Not queried | 1.65 | 6.86e-01 | 0.7223604052 |
|  | 9 | 34370781 | 34370894 | PDFSTATSENSEMBLUCSC | 114 | 4 | 40760 | gene;island | 0.803533 | 0.9665674 | Not queried | 1.65 | 4.86e-01 | 0.5640816327 |
|  | 12 | 54360131 | 54360511 | PDFSTATSENSEMBLUCSC | 381 | 4 | 9156 | gene;island | 0.347041 | 0.4297930 | Not queried | 1.65 | 6.86e-01 | 0.7223604052 |
|  | 5 | 138609219 | 138609700 | PDFSTATSENSEMBLUCSC | 482 | 6 | 32328 | tss | 0.007056 | 0.0251916 | Not queried | 1.65 | 1.80e-01 | 0.3826488233 |
|  | 11 | 93474820 | 93475054 | PDFSTATSENSEMBLUCSC | 235 | 7 | 6862 | gene | 0.021157 | 0.0181498 | Not queried | 1.65 | 5.35e-01 | 0.6176455124 |
|  | 1 | 227746111 | 227746453 | PDFSTATSENSEMBLUCSC | 343 | 4 | 4269 | island | 0.717156 | 0.8536863 | Not queried | 1.65 | 5.71e-02 | 0.2315877671 |
|  | 6 | 29589415 | 29590143 | PDFSTATSENSEMBLUCSC | 729 | 14 | 33675 | gene | 0.298318 | 0.7025327 | Not queried | 1.65 | 4.21e-04 | 0.0548331102 |
|  | 12 | 133179887 | 133180238 | PDFSTATSENSEMBLUCSC | 352 | 4 | 11065 | island | 0.589702 | 0.4101703 | Not queried | 1.64 | 1.14e-01 | 0.3123457953 |
|  | 2 | 222437040 | 222437130 | PDFSTATSENSEMBLUCSC | 91 | 6 | 23913 | tss | 0.009210 | 0.0046018 | Not queried | 1.64 | 3.94e-01 | 0.5236526859 |
|  | 6 | 31783315 | 31783545 | PDFSTATSENSEMBLUCSC | 231 | 11 | 33968 | gene | 0.006629 | 0.0101010 | Not queried | 1.64 | 4.00e-02 | 0.2180389943 |
|  | 8 | 10928985 | 10929210 | PDFSTATSENSEMBLUCSC | 226 | 4 | 39203 | gene;island | 0.680160 | 0.9095087 | Not queried | 1.64 | 2.00e-01 | 0.3826488233 |
|  | 20 | 3643863 | 3644300 | PDFSTATSENSEMBLUCSC | 438 | 6 | 25972 | island | 0.450334 | 0.8022348 | Not queried | 1.64 | 1.80e-01 | 0.3826488233 |
|  | 20 | 3644097 | 3644300 | PDFSTATSENSEMBLUCSC | 204 | 4 | 25566 | tss | 0.401709 | 0.8022348 | Not queried | 1.64 | 3.43e-01 | 0.4672865596 |
|  | 2 | 5836111 | 5836366 | PDFSTATSENSEMBLUCSC | 256 | 5 | 22558 | gene;island | 0.016714 | 0.0041611 | Not queried | 1.64 | 2.22e-01 | 0.3935758299 |
|  | 10 | 16561202 | 16561605 | PDFSTATSENSEMBLUCSC | 404 | 5 | 4515 | gene;island | 0.468438 | 0.5327622 | Not queried | 1.64 | 2.22e-01 | 0.3935758299 |
|  | 10 | 72200612 | 72201008 | PDFSTATSENSEMBLUCSC | 397 | 4 | 4619 | gene;island | 0.011116 | 0.0813750 | Not queried | 1.64 | 6.86e-01 | 0.7223604052 |
|  | 1 | 47489195 | 47489543 | PDFSTATSENSEMBLUCSC | 349 | 5 | 3228 | island | 0.095692 | 0.0367792 | Not queried | 1.64 | 4.21e-01 | 0.5404252807 |
|  | 7 | 151136940 | 151137192 | PDFSTATSENSEMBLUCSC | 253 | 5 | 39040 | island | 0.112871 | 0.0345011 | Not queried | 1.64 | 3.10e-01 | 0.4672865596 |
|  | 3 | 68981852 | 68982098 | PDFSTATSENSEMBLUCSC | 247 | 9 | 28437 | tss;island | 0.058628 | 0.0300646 | Not queried | 1.64 | 1.13e-01 | 0.3123457953 |
|  | 22 | 18531028 | 18531591 | PDFSTATSENSEMBLUCSC | 564 | 6 | 27282 | island | 0.526946 | 0.7784972 | Not queried | 1.64 | 2.40e-01 | 0.4133265075 |
|  | 4 | 2935692 | 2936284 | PDFSTATSENSEMBLUCSC | 593 | 9 | 29896 | gene;island | 0.004902 | 0.0062769 | Not queried | 1.64 | 3.87e-01 | 0.5209559659 |
|  | 17 | 17480829 | 17481059 | PDFSTATSENSEMBLUCSC | 231 | 5 | 16465 | gene;tss | 0.639154 | 0.8295058 | Not queried | 1.64 | 5.48e-01 | 0.6179718485 |
|  | 11 | 33891541 | 33891924 | PDFSTATSENSEMBLUCSC | 384 | 5 | 6579 | gene;tss;island | 0.026037 | 0.0258473 | Not queried | 1.64 | 1.00e+00 | 1.0000000000 |
|  | 7 | 50132541 | 50132764 | PDFSTATSENSEMBLUCSC | 224 | 4 | 37010 | gene | 0.020572 | 0.0158032 | Not queried | 1.64 | 6.86e-01 | 0.7223604052 |
|  | 5 | 72530105 | 72530245 | PDFSTATSENSEMBLUCSC | 141 | 4 | 32867 | island | 0.019250 | 0.0624046 | Not queried | 1.64 | 4.86e-01 | 0.5640816327 |
|  | 2 | 6635591 | 6636516 | PDFSTATSENSEMBLUCSC | 926 | 11 | 24043 | island | 0.772497 | 0.8915564 | Not queried | 1.64 | 2.43e-01 | 0.4165716700 |
|  | 19 | 52772664 | 52772724 | PDFSTATSENSEMBLUCSC | 61 | 4 | 21010 | tss | 0.019215 | 0.0093625 | Not queried | 1.64 | 6.86e-01 | 0.7223604052 |
|  | 5 | 63802106 | 63802412 | PDFSTATSENSEMBLUCSC | 307 | 5 | 32089 | tss | 0.131210 | 0.0181527 | Not queried | 1.64 | 6.90e-01 | 0.7223604052 |
|  | 7 | 97841334 | 97841513 | PDFSTATSENSEMBLUCSC | 180 | 5 | 37743 | tss | 0.938607 | 0.5764505 | Not queried | 1.64 | 5.48e-01 | 0.6179718485 |
|  | 20 | 53091600 | 53092259 | PDFSTATSENSEMBLUCSC | 660 | 6 | 25831 | tss | 0.072733 | 0.0156724 | Not queried | 1.64 | 6.99e-01 | 0.7290267580 |
|  | 10 | 71176719 | 71176853 | PDFSTATSENSEMBLUCSC | 135 | 4 | 5124 | tss | 0.464679 | 0.5799152 | Not queried | 1.64 | 6.86e-01 | 0.7223604052 |
|  | 6 | 111136481 | 111136640 | PDFSTATSENSEMBLUCSC | 160 | 4 | 35171 | tss | 0.003147 | 0.0033969 | Not queried | 1.64 | 4.86e-01 | 0.5640816327 |
|  | 12 | 93096622 | 93096819 | PDFSTATSENSEMBLUCSC | 198 | 4 | 9913 | tss | 0.006525 | 0.0055683 | Not queried | 1.64 | 4.86e-01 | 0.5640816327 |
|  | 6 | 100903561 | 100904055 | PDFSTATSENSEMBLUCSC | 495 | 7 | 34431 | gene;island | 0.090723 | 0.0069902 | Not queried | 1.64 | 3.83e-01 | 0.5168893301 |
|  | 6 | 69345836 | 69346248 | PDFSTATSENSEMBLUCSC | 413 | 5 | 34379 | gene;island | 0.014336 | 0.0271453 | Not queried | 1.64 | 1.51e-01 | 0.3618000441 |
|  | 3 | 140770213 | 140770984 | PDFSTATSENSEMBLUCSC | 772 | 14 | 29543 | island | 0.009634 | 0.0484192 | Not queried | 1.63 | 7.67e-02 | 0.2825346647 |
|  | 10 | 1509675 | 1510077 | PDFSTATSENSEMBLUCSC | 403 | 5 | 4465 | gene;island | 0.451686 | 0.7083001 | Not queried | 1.63 | 5.48e-01 | 0.6179718485 |
|  | 17 | 46688182 | 46688329 | PDFSTATSENSEMBLUCSC | 148 | 4 | 16659 | gene | 0.001361 | 0.0017871 | Not queried | 1.63 | 2.00e-01 | 0.3826488233 |
|  | 8 | 117950244 | 117950504 | PDFSTATSENSEMBLUCSC | 261 | 7 | 40553 | island | 0.362681 | 0.2056712 | Not queried | 1.63 | 3.18e-01 | 0.4672865596 |
|  | 1 | 46958475 | 46958856 | PDFSTATSENSEMBLUCSC | 382 | 4 | 3220 | island | 0.094186 | 0.0872342 | Not queried | 1.63 | 8.86e-01 | 0.8941250130 |
|  | 6 | 159462964 | 159463300 | PDFSTATSENSEMBLUCSC | 337 | 4 | 34532 | gene | 0.787936 | 0.9720642 | Not queried | 1.63 | 6.86e-01 | 0.7223604052 |
|  | 16 | 30023400 | 30023780 | PDFSTATSENSEMBLUCSC | 381 | 4 | 15176 | tss;island | 0.289602 | 0.5575527 | Not queried | 1.63 | 3.43e-01 | 0.4672865596 |
|  | 19 | 51531297 | 51531484 | PDFSTATSENSEMBLUCSC | 188 | 4 | 20992 | tss;island | 0.044374 | 0.0926669 | Not queried | 1.63 | 8.86e-01 | 0.8941250130 |
|  | 6 | 29626990 | 29627395 | PDFSTATSENSEMBLUCSC | 406 | 8 | 33684 | gene | 0.488594 | 0.7604528 | Not queried | 1.63 | 4.99e-02 | 0.2315877671 |
|  | 16 | 55542668 | 55542751 | PDFSTATSENSEMBLUCSC | 84 | 4 | 15240 | tss | 0.541110 | 0.7808951 | Not queried | 1.63 | 1.14e-01 | 0.3123457953 |
|  | 3 | 46924768 | 46925181 | PDFSTATSENSEMBLUCSC | 414 | 4 | 27756 | gene;island | 0.038351 | 0.0082836 | Not queried | 1.63 | 1.00e+00 | 1.0000000000 |
|  | 1 | 110163175 | 110163427 | PDFSTATSENSEMBLUCSC | 253 | 4 | 670 | gene;island | 0.017637 | 0.0475135 | Not queried | 1.63 | 6.86e-01 | 0.7223604052 |
|  | 2 | 187713902 | 187714279 | PDFSTATSENSEMBLUCSC | 378 | 9 | 23775 | tss | 0.059675 | 0.0207510 | Not queried | 1.63 | 5.46e-01 | 0.6179718485 |
|  | 4 | 84376750 | 84376998 | PDFSTATSENSEMBLUCSC | 249 | 5 | 30045 | gene | 0.002863 | 0.0087887 | Not queried | 1.63 | 6.90e-01 | 0.7223604052 |
|  | 11 | 33183108 | 33183473 | PDFSTATSENSEMBLUCSC | 366 | 7 | 7232 | tss | 0.001650 | 0.0008811 | Not queried | 1.63 | 9.73e-02 | 0.3123457953 |
|  | 13 | 114085424 | 114085732 | PDFSTATSENSEMBLUCSC | 309 | 5 | 11285 | gene;island | 0.746358 | 0.8888950 | Not queried | 1.63 | 2.22e-01 | 0.3935758299 |
|  | 8 | 122654023 | 122654328 | PDFSTATSENSEMBLUCSC | 306 | 4 | 39415 | gene;tss;island | 0.001079 | 0.0066885 | Not queried | 1.63 | 1.14e-01 | 0.3123457953 |
|  | 13 | 36871646 | 36872346 | PDFSTATSENSEMBLUCSC | 701 | 14 | 11668 | island | 0.071491 | 0.2733505 | Not queried | 1.63 | 2.74e-02 | 0.1830867741 |
|  | 5 | 3854465 | 3854732 | PDFSTATSENSEMBLUCSC | 268 | 5 | 32670 | island | 0.548181 | 0.8376611 | Not queried | 1.63 | 3.10e-01 | 0.4672865596 |
|  | 2 | 131673771 | 131674687 | PDFSTATSENSEMBLUCSC | 917 | 11 | 24725 | island | 0.253113 | 0.4803829 | Not queried | 1.63 | 1.51e-01 | 0.3627442585 |
|  | 7 | 193229 | 193732 | PDFSTATSENSEMBLUCSC | 504 | 5 | 36701 | gene;island | 0.129964 | 0.1777295 | Not queried | 1.63 | 1.00e+00 | 1.0000000000 |
|  | 1 | 2359927 | 2360074 | PDFSTATSENSEMBLUCSC | 148 | 4 | 2501 | island | 0.537643 | 0.7529460 | Not queried | 1.63 | 5.71e-02 | 0.2315877671 |
|  | 4 | 77997182 | 77997201 | PDFSTATSENSEMBLUCSC | 20 | 4 | 30452 | tss;island | 0.002788 | 0.0024533 | Not queried | 1.63 | 4.86e-01 | 0.5640816327 |
|  | 5 | 140743780 | 140744155 | PDFSTATSENSEMBLUCSC | 376 | 6 | 31823 | gene;island | 0.500021 | 0.7084960 | Not queried | 1.63 | 2.40e-01 | 0.4133265075 |
|  | 15 | 67417607 | 67417964 | PDFSTATSENSEMBLUCSC | 358 | 6 | 13392 | gene;tss | 0.921387 | 0.5672181 | Not queried | 1.63 | 2.40e-01 | 0.4133265075 |
|  | 1 | 177133536 | 177133737 | PDFSTATSENSEMBLUCSC | 202 | 4 | 897 | gene;island | 0.016803 | 0.0407187 | Not queried | 1.63 | 1.14e-01 | 0.3123457953 |
|  | 1 | 60539362 | 60539675 | PDFSTATSENSEMBLUCSC | 314 | 6 | 3327 | island | 0.047965 | 0.3123748 | Not queried | 1.63 | 2.40e-01 | 0.4133265075 |
|  | 10 | 102809954 | 102810157 | PDFSTATSENSEMBLUCSC | 204 | 5 | 6048 | island | 0.009638 | 0.0486468 | Not queried | 1.63 | 2.22e-01 | 0.3935758299 |
|  | 1 | 2984218 | 2984869 | PDFSTATSENSEMBLUCSC | 652 | 10 | 2528 | island | 0.156891 | 0.4039699 | Not queried | 1.63 | 3.15e-01 | 0.4672865596 |
|  | 19 | 52873173 | 52873624 | PDFSTATSENSEMBLUCSC | 452 | 4 | 20194 | gene | 0.068617 | 0.0367662 | Not queried | 1.63 | 3.43e-01 | 0.4672865596 |
|  | 10 | 134977981 | 134978147 | PDFSTATSENSEMBLUCSC | 167 | 5 | 4887 | gene;island | 0.301777 | 0.6562851 | Not queried | 1.62 | 2.22e-01 | 0.3935758299 |
|  | 22 | 30116919 | 30117159 | PDFSTATSENSEMBLUCSC | 241 | 4 | 26834 | gene;island | 0.447388 | 0.2127058 | Not queried | 1.62 | 4.86e-01 | 0.5640816327 |
|  | 12 | 109826364 | 109826496 | PDFSTATSENSEMBLUCSC | 133 | 4 | 9991 | tss | 0.943809 | 0.9513428 | Not queried | 1.62 | 6.86e-01 | 0.7223604052 |
|  | 6 | 32804435 | 32804677 | PDFSTATSENSEMBLUCSC | 243 | 4 | 34145 | gene;island | 0.557786 | 0.6779277 | Not queried | 1.62 | 6.86e-01 | 0.7223604052 |
|  | 12 | 57588031 | 57588243 | PDFSTATSENSEMBLUCSC | 213 | 7 | 9811 | tss | 0.488030 | 0.7961411 | Not queried | 1.62 | 7.28e-02 | 0.2713481487 |
|  | 11 | 130059549 | 130059791 | PDFSTATSENSEMBLUCSC | 243 | 5 | 6977 | gene;island | 0.447162 | 0.2718843 | Not queried | 1.62 | 5.56e-02 | 0.2315877671 |
|  | 4 | 41867415 | 41867562 | PDFSTATSENSEMBLUCSC | 148 | 5 | 31005 | island | 0.020640 | 0.1137083 | Not queried | 1.62 | 4.21e-01 | 0.5404252807 |
|  | 11 | 102187898 | 102188180 | PDFSTATSENSEMBLUCSC | 283 | 7 | 7647 | tss | 0.001289 | 0.0050936 | Not queried | 1.62 | 2.09e-01 | 0.3935758299 |
|  | 11 | 120894635 | 120894801 | PDFSTATSENSEMBLUCSC | 167 | 4 | 7765 | tss | 0.012953 | 0.0042195 | Not queried | 1.62 | 2.00e-01 | 0.3826488233 |
|  | 19 | 49925720 | 49926070 | PDFSTATSENSEMBLUCSC | 351 | 4 | 20149 | gene;island | 0.047504 | 0.2519362 | Not queried | 1.62 | 3.43e-01 | 0.4672865596 |
|  | 12 | 120426529 | 120426784 | PDFSTATSENSEMBLUCSC | 256 | 4 | 10054 | tss;island | 0.010290 | 0.0060968 | Not queried | 1.62 | 6.86e-01 | 0.7223604052 |
|  | 11 | 67442126 | 67442408 | PDFSTATSENSEMBLUCSC | 283 | 4 | 7501 | tss | 0.268444 | 0.4490576 | Not queried | 1.62 | 3.43e-01 | 0.4672865596 |
|  | 17 | 37895771 | 37896175 | PDFSTATSENSEMBLUCSC | 405 | 5 | 16556 | gene | 0.494825 | 0.8634164 | Not queried | 1.62 | 1.51e-01 | 0.3618000441 |
|  | 13 | 103052144 | 103052560 | PDFSTATSENSEMBLUCSC | 417 | 5 | 11215 | gene;island | 0.042030 | 0.1203582 | Not queried | 1.62 | 1.51e-01 | 0.3618000441 |
|  | 3 | 38664022 | 38664451 | PDFSTATSENSEMBLUCSC | 430 | 4 | 27722 | gene | 0.232488 | 0.3453286 | Not queried | 1.62 | 4.86e-01 | 0.5640816327 |
|  | 14 | 74705942 | 74706336 | PDFSTATSENSEMBLUCSC | 395 | 8 | 12997 | island | 0.144956 | 0.3424674 | Not queried | 1.62 | 1.95e-01 | 0.3826488233 |
|  | 13 | 111773936 | 111774127 | PDFSTATSENSEMBLUCSC | 192 | 4 | 11242 | gene | 0.672408 | 0.8373891 | Not queried | 1.62 | 4.86e-01 | 0.5640816327 |
|  | 19 | 41858816 | 41859304 | PDFSTATSENSEMBLUCSC | 489 | 4 | 20057 | gene;island | 0.005006 | 0.0028399 | Not queried | 1.62 | 2.00e-01 | 0.3826488233 |
|  | 18 | 12253749 | 12254566 | PDFSTATSENSEMBLUCSC | 818 | 18 | 19186 | tss | 0.052308 | 0.1777130 | Not queried | 1.62 | 4.16e-03 | 0.1459191215 |
|  | 6 | 39760416 | 39760833 | PDFSTATSENSEMBLUCSC | 418 | 5 | 36112 | island | 0.100659 | 0.0361678 | Not queried | 1.62 | 3.10e-01 | 0.4672865596 |
|  | 20 | 61435829 | 61436024 | PDFSTATSENSEMBLUCSC | 196 | 6 | 25881 | tss;island | 0.001770 | 0.0079167 | Not queried | 1.62 | 3.10e-01 | 0.4672865596 |
|  | 16 | 2264855 | 2264884 | PDFSTATSENSEMBLUCSC | 30 | 4 | 15022 | tss;island | 0.005456 | 0.0046416 | Not queried | 1.62 | 3.43e-01 | 0.4672865596 |
|  | 10 | 135340445 | 135341025 | PDFSTATSENSEMBLUCSC | 581 | 9 | 6367 | island | 0.361049 | 0.6929544 | Not queried | 1.62 | 5.03e-02 | 0.2315877671 |
|  | 10 | 135340445 | 135340850 | PDFSTATSENSEMBLUCSC | 406 | 7 | 5465 | tss | 0.361049 | 0.6929544 | Not queried | 1.62 | 1.28e-01 | 0.3397986247 |
|  | 19 | 48949047 | 48949463 | PDFSTATSENSEMBLUCSC | 417 | 5 | 20125 | gene | 0.007526 | 0.0027882 | Not queried | 1.62 | 6.90e-01 | 0.7223604052 |
|  | 5 | 37834672 | 37835168 | PDFSTATSENSEMBLUCSC | 497 | 7 | 31590 | gene;island | 0.091218 | 0.0228805 | Not queried | 1.62 | 2.09e-01 | 0.3935758299 |
|  | 17 | 3558202 | 3558426 | PDFSTATSENSEMBLUCSC | 225 | 4 | 16342 | gene | 0.938250 | 0.9793887 | Not queried | 1.62 | 6.86e-01 | 0.7223604052 |
|  | 20 | 13765341 | 13765530 | PDFSTATSENSEMBLUCSC | 190 | 5 | 25360 | gene | 0.004460 | 0.0004637 | Not queried | 1.62 | 4.21e-01 | 0.5404252807 |
|  | 1 | 155006911 | 155007323 | PDFSTATSENSEMBLUCSC | 413 | 5 | 788 | gene;tss | 0.319533 | 0.5997103 | Not queried | 1.62 | 5.56e-02 | 0.2315877671 |
|  | 17 | 1837588 | 1837848 | PDFSTATSENSEMBLUCSC | 261 | 6 | 17834 | island | 0.556131 | 0.7298155 | Not queried | 1.62 | 1.52e-02 | 0.1830867741 |
|  | 8 | 12613139 | 12613305 | PDFSTATSENSEMBLUCSC | 167 | 4 | 39558 | tss;island | 0.001694 | 0.0031260 | Not queried | 1.61 | 5.71e-02 | 0.2315877671 |
|  | 17 | 29297380 | 29297873 | PDFSTATSENSEMBLUCSC | 494 | 14 | 17245 | tss;island | 0.003779 | 0.0164551 | Not queried | 1.61 | 8.49e-02 | 0.3096829949 |
|  | 2 | 133174035 | 133174168 | PDFSTATSENSEMBLUCSC | 134 | 6 | 24741 | island | 0.018527 | 0.0587871 | Not queried | 1.61 | 1.32e-01 | 0.3397986247 |
|  | 1 | 33816020 | 33816159 | PDFSTATSENSEMBLUCSC | 140 | 5 | 404 | gene;tss;island | 0.007098 | 0.0105188 | Not queried | 1.61 | 4.21e-01 | 0.5404252807 |
|  | 8 | 22960177 | 22960619 | PDFSTATSENSEMBLUCSC | 443 | 10 | 40145 | island | 0.043281 | 0.1576534 | Not queried | 1.61 | 5.24e-02 | 0.2315877671 |
|  | 8 | 22960177 | 22960427 | PDFSTATSENSEMBLUCSC | 251 | 6 | 39596 | tss | 0.043281 | 0.1576534 | Not queried | 1.61 | 6.49e-02 | 0.2477578846 |
|  | 1 | 86888585 | 86888728 | PDFSTATSENSEMBLUCSC | 144 | 4 | 1727 | tss | 0.429740 | 0.6624540 | Not queried | 1.61 | 3.43e-01 | 0.4672865596 |
|  | 14 | 105635706 | 105635769 | PDFSTATSENSEMBLUCSC | 64 | 4 | 12667 | tss | 0.085190 | 0.0297904 | Not queried | 1.61 | 1.14e-01 | 0.3123457953 |
|  | 11 | 2889809 | 2890725 | PDFSTATSENSEMBLUCSC | 917 | 32 | 7078 | tss | 0.322148 | 0.0521004 | Not queried | 1.61 | 8.37e-03 | 0.1595382395 |
|  | 6 | 33159353 | 33159593 | PDFSTATSENSEMBLUCSC | 241 | 5 | 34215 | gene;island | 0.415787 | 0.4424883 | Not queried | 1.61 | 5.48e-01 | 0.6179718485 |
|  | 5 | 110427798 | 110427863 | PDFSTATSENSEMBLUCSC | 66 | 4 | 32207 | tss | 0.011894 | 0.0192052 | Not queried | 1.61 | 1.00e+00 | 1.0000000000 |
|  | 2 | 38152187 | 38152364 | PDFSTATSENSEMBLUCSC | 178 | 7 | 23308 | tss;island | 0.028567 | 0.0587901 | Not queried | 1.61 | 7.28e-02 | 0.2713481487 |
|  | 6 | 52284632 | 52284958 | PDFSTATSENSEMBLUCSC | 327 | 6 | 35032 | tss | 0.003368 | 0.0052754 | Not queried | 1.61 | 6.99e-01 | 0.7290267580 |
|  | 15 | 89089914 | 89090195 | PDFSTATSENSEMBLUCSC | 282 | 4 | 13880 | tss | 0.033950 | 0.0091001 | Not queried | 1.61 | 6.86e-01 | 0.7223604052 |
|  | 4 | 141264393 | 141264721 | PDFSTATSENSEMBLUCSC | 329 | 5 | 30136 | gene | 0.004265 | 0.0525323 | Not queried | 1.61 | 3.10e-01 | 0.4672865596 |
|  | 2 | 124782117 | 124782263 | PDFSTATSENSEMBLUCSC | 147 | 4 | 23606 | tss;island | 0.324011 | 0.5943983 | Not queried | 1.61 | 6.86e-01 | 0.7223604052 |
|  | 8 | 49890360 | 49890873 | PDFSTATSENSEMBLUCSC | 514 | 5 | 40288 | island | 0.752072 | 0.8940682 | Not queried | 1.61 | 1.51e-01 | 0.3618000441 |
|  | 19 | 35085512 | 35085677 | PDFSTATSENSEMBLUCSC | 166 | 4 | 20693 | tss | 0.508526 | 0.7718167 | Not queried | 1.61 | 4.86e-01 | 0.5640816327 |
|  | 15 | 31195612 | 31196075 | PDFSTATSENSEMBLUCSC | 464 | 9 | 13980 | island | 0.029916 | 0.0096563 | Not queried | 1.61 | 4.36e-01 | 0.5589686471 |
|  | 5 | 178772203 | 178772390 | PDFSTATSENSEMBLUCSC | 188 | 4 | 33483 | island | 0.090758 | 0.0255323 | Not queried | 1.61 | 2.86e-02 | 0.1830867741 |
|  | 12 | 54376019 | 54376241 | PDFSTATSENSEMBLUCSC | 223 | 4 | 10485 | island | 0.287527 | 0.2558260 | Not queried | 1.61 | 6.86e-01 | 0.7223604052 |
|  | 10 | 88296171 | 88296605 | PDFSTATSENSEMBLUCSC | 435 | 4 | 5906 | island | 0.186183 | 0.1675723 | Not queried | 1.61 | 6.86e-01 | 0.7223604052 |
|  | 10 | 132910868 | 132911152 | PDFSTATSENSEMBLUCSC | 285 | 4 | 4841 | gene | 0.451153 | 0.6717339 | Not queried | 1.61 | 4.86e-01 | 0.5640816327 |
|  | 4 | 159093841 | 159094034 | PDFSTATSENSEMBLUCSC | 194 | 4 | 30162 | gene | 0.003620 | 0.0029554 | Not queried | 1.61 | 6.86e-01 | 0.7223604052 |
|  | 10 | 105239085 | 105239205 | PDFSTATSENSEMBLUCSC | 121 | 4 | 5339 | tss | 0.762195 | 0.8893549 | Not queried | 1.61 | 3.43e-01 | 0.4672865596 |
|  | 8 | 18666531 | 18666598 | PDFSTATSENSEMBLUCSC | 68 | 4 | 39220 | gene;tss | 0.285265 | 0.4283312 | Not queried | 1.61 | 4.86e-01 | 0.5640816327 |
|  | 15 | 69853608 | 69854024 | PDFSTATSENSEMBLUCSC | 417 | 4 | 13765 | tss | 0.150136 | 0.3784376 | Not queried | 1.61 | 2.00e-01 | 0.3826488233 |
|  | 4 | 111544235 | 111544463 | PDFSTATSENSEMBLUCSC | 229 | 8 | 30095 | gene;island | 0.069702 | 0.0196600 | Not queried | 1.60 | 4.66e-03 | 0.1498346615 |
|  | 6 | 39895360 | 39895717 | PDFSTATSENSEMBLUCSC | 358 | 5 | 34326 | gene | 0.356723 | 0.6275579 | Not queried | 1.60 | 6.90e-01 | 0.7223604052 |
|  | 19 | 9420290 | 9420631 | PDFSTATSENSEMBLUCSC | 342 | 6 | 21495 | island | 0.001629 | 0.0042662 | Not queried | 1.60 | 5.89e-01 | 0.6570484697 |
|  | 2 | 27712613 | 27713092 | PDFSTATSENSEMBLUCSC | 480 | 10 | 23271 | tss | 0.006120 | 0.0256674 | Not queried | 1.60 | 1.05e-01 | 0.3123457953 |
|  | 8 | 85096835 | 85097429 | PDFSTATSENSEMBLUCSC | 595 | 9 | 39352 | gene;island | 0.023785 | 0.0433944 | Not queried | 1.60 | 1.90e-01 | 0.3826488233 |
|  | 6 | 29797211 | 29797420 | PDFSTATSENSEMBLUCSC | 210 | 4 | 33698 | gene;island | 0.342904 | 0.5516839 | Not queried | 1.60 | 3.43e-01 | 0.4672865596 |
|  | 8 | 144653855 | 144653975 | PDFSTATSENSEMBLUCSC | 121 | 4 | 39484 | gene;island | 0.012274 | 0.0185735 | Not queried | 1.60 | 4.86e-01 | 0.5640816327 |
|  | 12 | 110270919 | 110271031 | PDFSTATSENSEMBLUCSC | 113 | 4 | 9296 | gene;island | 0.266165 | 0.0736258 | Not queried | 1.60 | 5.71e-02 | 0.2315877671 |
|  | 2 | 241807708 | 241808113 | PDFSTATSENSEMBLUCSC | 406 | 6 | 23982 | tss | 0.239073 | 0.3317992 | Not queried | 1.60 | 3.10e-01 | 0.4672865596 |
|  | 2 | 220436894 | 220436948 | PDFSTATSENSEMBLUCSC | 55 | 4 | 23910 | tss;island | 0.471329 | 0.7345231 | Not queried | 1.60 | 5.71e-02 | 0.2315877671 |
|  | 11 | 16631866 | 16632088 | PDFSTATSENSEMBLUCSC | 223 | 4 | 8090 | island | 0.623185 | 0.3920192 | Not queried | 1.60 | 2.00e-01 | 0.3826488233 |
|  | 16 | 55542668 | 55543166 | PDFSTATSENSEMBLUCSC | 499 | 9 | 15936 | island | 0.074553 | 0.5686716 | Not queried | 1.60 | 2.97e-01 | 0.4672865596 |
|  | 16 | 55542921 | 55543166 | PDFSTATSENSEMBLUCSC | 246 | 5 | 14753 | gene | 0.036465 | 0.0477288 | Not queried | 1.60 | 5.48e-01 | 0.6179718485 |
|  | 7 | 48495072 | 48495333 | PDFSTATSENSEMBLUCSC | 262 | 4 | 37008 | gene;island | 0.553786 | 0.7216637 | Not queried | 1.60 | 4.86e-01 | 0.5640816327 |
|  | 19 | 49106849 | 49107139 | PDFSTATSENSEMBLUCSC | 291 | 4 | 20129 | gene;island | 0.893000 | 0.9579269 | Not queried | 1.60 | 1.14e-01 | 0.3123457953 |
|  | 3 | 139257713 | 139257907 | PDFSTATSENSEMBLUCSC | 195 | 5 | 27969 | gene;island | 0.435385 | 0.2028965 | Not queried | 1.60 | 1.00e+00 | 1.0000000000 |
|  | 14 | 23235611 | 23235724 | PDFSTATSENSEMBLUCSC | 114 | 4 | 12287 | tss | 0.007022 | 0.0017416 | Not queried | 1.60 | 2.00e-01 | 0.3826488233 |
|  | 12 | 57569768 | 57569940 | PDFSTATSENSEMBLUCSC | 173 | 4 | 9183 | gene;island | 0.493196 | 0.8487071 | Not queried | 1.60 | 4.86e-01 | 0.5640816327 |
|  | 12 | 12223661 | 12223989 | PDFSTATSENSEMBLUCSC | 329 | 4 | 9567 | tss | 0.507922 | 0.5244277 | Not queried | 1.60 | 8.86e-01 | 0.8941250130 |
|  | 1 | 2273591 | 2273958 | PDFSTATSENSEMBLUCSC | 368 | 4 | 76 | gene;island | 0.243297 | 0.4251328 | Not queried | 1.60 | 6.86e-01 | 0.7223604052 |
|  | 16 | 1598866 | 1599150 | PDFSTATSENSEMBLUCSC | 285 | 4 | 14576 | gene;island | 0.596112 | 0.7375993 | Not queried | 1.60 | 3.43e-01 | 0.4672865596 |
|  | 10 | 95198 | 95398 | PDFSTATSENSEMBLUCSC | 201 | 6 | 4908 | tss | 0.231801 | 0.5262028 | Not queried | 1.60 | 1.32e-01 | 0.3397986247 |
|  | 2 | 223155841 | 223156136 | PDFSTATSENSEMBLUCSC | 296 | 4 | 23044 | gene;island | 0.207621 | 0.0518450 | Not queried | 1.60 | 4.86e-01 | 0.5640816327 |
|  | 3 | 126113327 | 126114064 | PDFSTATSENSEMBLUCSC | 738 | 13 | 29432 | island | 0.077978 | 0.3442094 | Not queried | 1.60 | 4.41e-02 | 0.2315877671 |
|  | 10 | 48413742 | 48413956 | PDFSTATSENSEMBLUCSC | 215 | 4 | 4577 | gene;island | 0.501642 | 0.6338418 | Not queried | 1.60 | 6.86e-01 | 0.7223604052 |
|  | 16 | 891260 | 891400 | PDFSTATSENSEMBLUCSC | 141 | 4 | 15493 | island | 0.816418 | 0.9555346 | Not queried | 1.60 | 1.14e-01 | 0.3123457953 |
|  | 11 | 118134801 | 118134959 | PDFSTATSENSEMBLUCSC | 159 | 4 | 6927 | gene | 0.033305 | 0.0279764 | Not queried | 1.60 | 1.00e+00 | 1.0000000000 |
|  | 14 | 36989287 | 36989588 | PDFSTATSENSEMBLUCSC | 302 | 6 | 12358 | tss;island | 0.032556 | 0.0550841 | Not queried | 1.60 | 5.89e-01 | 0.6570484697 |
|  | 1 | 11795746 | 11796079 | PDFSTATSENSEMBLUCSC | 334 | 10 | 1249 | tss | 0.022372 | 0.2148243 | Not queried | 1.59 | 3.93e-01 | 0.5236526859 |
|  | 3 | 45066580 | 45066971 | PDFSTATSENSEMBLUCSC | 392 | 4 | 28266 | tss | 0.896176 | 0.7056556 | Not queried | 1.59 | 3.43e-01 | 0.4672865596 |
|  | 11 | 63827695 | 63828346 | PDFSTATSENSEMBLUCSC | 652 | 5 | 6660 | gene | 0.211995 | 0.5249339 | Not queried | 1.59 | 2.22e-01 | 0.3935758299 |
|  | 16 | 10674326 | 10674468 | PDFSTATSENSEMBLUCSC | 143 | 5 | 14660 | gene | 0.005134 | 0.0040592 | Not queried | 1.59 | 5.48e-01 | 0.6179718485 |
|  | 16 | 20360206 | 20360497 | PDFSTATSENSEMBLUCSC | 292 | 4 | 14683 | gene;island | 0.299516 | 0.4492679 | Not queried | 1.59 | 3.43e-01 | 0.4672865596 |
|  | 12 | 7863853 | 7864076 | PDFSTATSENSEMBLUCSC | 224 | 5 | 9550 | tss | 0.479461 | 0.7691710 | Not queried | 1.59 | 5.56e-02 | 0.2315877671 |
|  | 10 | 123353500 | 123353956 | PDFSTATSENSEMBLUCSC | 457 | 8 | 5404 | tss | 0.351142 | 0.5732475 | Not queried | 1.59 | 2.79e-01 | 0.4672865596 |
|  | 16 | 103423 | 103592 | PDFSTATSENSEMBLUCSC | 170 | 4 | 14521 | gene | 0.111802 | 0.0308726 | Not queried | 1.59 | 3.43e-01 | 0.4672865596 |
|  | 13 | 35515865 | 35516223 | PDFSTATSENSEMBLUCSC | 359 | 4 | 11389 | tss | 0.016825 | 0.0054476 | Not queried | 1.59 | 2.00e-01 | 0.3826488233 |
|  | 7 | 100199764 | 100200009 | PDFSTATSENSEMBLUCSC | 246 | 6 | 38770 | island | 0.759082 | 0.5270757 | Not queried | 1.59 | 6.99e-01 | 0.7290267580 |
|  | 2 | 209010890 | 209011053 | PDFSTATSENSEMBLUCSC | 164 | 5 | 23847 | tss | 0.244850 | 0.4849821 | Not queried | 1.59 | 2.22e-01 | 0.3935758299 |
|  | 14 | 29254530 | 29255119 | PDFSTATSENSEMBLUCSC | 590 | 6 | 12055 | gene;island | 0.178437 | 0.0726748 | Not queried | 1.59 | 4.85e-01 | 0.5640816327 |
|  | 2 | 171571533 | 171571941 | PDFSTATSENSEMBLUCSC | 409 | 8 | 23709 | tss;island | 0.036043 | 0.0206971 | Not queried | 1.59 | 8.30e-02 | 0.3028612233 |
|  | 14 | 54430382 | 54430831 | PDFSTATSENSEMBLUCSC | 450 | 4 | 12854 | island | 0.008788 | 0.1020782 | Not queried | 1.59 | 6.86e-01 | 0.7223604052 |
|  | 6 | 43230558 | 43231063 | PDFSTATSENSEMBLUCSC | 506 | 5 | 34346 | gene;island | 0.740533 | 0.8945480 | Not queried | 1.59 | 3.10e-01 | 0.4672865596 |
|  | 7 | 98245716 | 98246006 | PDFSTATSENSEMBLUCSC | 291 | 4 | 37747 | tss;island | 0.242813 | 0.2155416 | Not queried | 1.59 | 6.86e-01 | 0.7223604052 |
|  | 6 | 6004686 | 6004897 | PDFSTATSENSEMBLUCSC | 212 | 4 | 33545 | gene;island | 0.094257 | 0.1277059 | Not queried | 1.59 | 1.00e+00 | 1.0000000000 |
|  | 4 | 80246713 | 80247088 | PDFSTATSENSEMBLUCSC | 376 | 4 | 30032 | gene | 0.208028 | 0.4359019 | Not queried | 1.59 | 2.00e-01 | 0.3826488233 |
|  | 20 | 43150917 | 43151161 | PDFSTATSENSEMBLUCSC | 245 | 4 | 25761 | tss;island | 0.001545 | 0.0007211 | Not queried | 1.59 | 3.43e-01 | 0.4672865596 |
|  | 4 | 47033126 | 47033634 | PDFSTATSENSEMBLUCSC | 509 | 8 | 31022 | island | 0.035472 | 0.0195677 | Not queried | 1.59 | 5.74e-01 | 0.6460388787 |
|  | 15 | 42289618 | 42289939 | PDFSTATSENSEMBLUCSC | 322 | 4 | 13306 | gene | 0.545830 | 0.7577666 | Not queried | 1.59 | 3.43e-01 | 0.4672865596 |
|  | 2 | 11924984 | 11925375 | PDFSTATSENSEMBLUCSC | 392 | 4 | 22575 | gene;island | 0.478383 | 0.3126958 | Not queried | 1.59 | 4.86e-01 | 0.5640816327 |
|  | 12 | 4554786 | 4554971 | PDFSTATSENSEMBLUCSC | 186 | 6 | 9498 | tss | 0.696877 | 0.8551511 | Not queried | 1.59 | 2.40e-01 | 0.4133265075 |
|  | 1 | 116184219 | 116184423 | PDFSTATSENSEMBLUCSC | 205 | 4 | 1859 | tss;island | 0.141068 | 0.4281782 | Not queried | 1.59 | 6.86e-01 | 0.7223604052 |
|  | 9 | 124262344 | 124262699 | PDFSTATSENSEMBLUCSC | 356 | 5 | 40835 | tss;island | 0.107304 | 0.0329016 | Not queried | 1.59 | 4.21e-01 | 0.5404252807 |
|  | 17 | 43503394 | 43503638 | PDFSTATSENSEMBLUCSC | 245 | 4 | 17430 | tss;island | 0.248393 | 0.5929308 | Not queried | 1.59 | 3.43e-01 | 0.4672865596 |
|  | 1 | 33722125 | 33722135 | PDFSTATSENSEMBLUCSC | 11 | 4 | 1472 | tss;island | 0.007664 | 0.0042798 | Not queried | 1.59 | 3.43e-01 | 0.4672865596 |
|  | 6 | 32634245 | 32634508 | PDFSTATSENSEMBLUCSC | 264 | 6 | 35922 | island | 0.017910 | 0.0188413 | Not queried | 1.59 | 8.18e-01 | 0.8469867211 |
|  | 5 | 92916862 | 92917125 | PDFSTATSENSEMBLUCSC | 264 | 4 | 32959 | island | 0.011379 | 0.0144358 | Not queried | 1.59 | 6.86e-01 | 0.7223604052 |
|  | 4 | 40752692 | 40752947 | PDFSTATSENSEMBLUCSC | 256 | 4 | 29988 | gene;island | 0.424982 | 0.1691628 | Not queried | 1.59 | 3.43e-01 | 0.4672865596 |
|  | 14 | 89628141 | 89628422 | PDFSTATSENSEMBLUCSC | 282 | 4 | 12169 | gene;island | 0.979459 | 0.9252491 | Not queried | 1.59 | 2.00e-01 | 0.3826488233 |
|  | 2 | 106681831 | 106681998 | PDFSTATSENSEMBLUCSC | 168 | 5 | 23556 | tss;island | 0.390784 | 0.1124596 | Not queried | 1.59 | 4.21e-01 | 0.5404252807 |
|  | 17 | 45908849 | 45908985 | PDFSTATSENSEMBLUCSC | 137 | 4 | 17445 | tss;island | 0.001831 | 0.0034994 | Not queried | 1.59 | 1.14e-01 | 0.3123457953 |
|  | 20 | 23471627 | 23471737 | PDFSTATSENSEMBLUCSC | 111 | 4 | 25640 | tss | 0.420201 | 0.7238814 | Not queried | 1.59 | 1.14e-01 | 0.3123457953 |
|  | 12 | 49110427 | 49110646 | PDFSTATSENSEMBLUCSC | 220 | 4 | 9113 | gene | 0.005141 | 0.0077721 | Not queried | 1.59 | 4.86e-01 | 0.5640816327 |
|  | 1 | 42921582 | 42921899 | PDFSTATSENSEMBLUCSC | 318 | 5 | 465 | gene | 0.013771 | 0.0146973 | Not queried | 1.59 | 8.41e-01 | 0.8635565145 |
|  | 12 | 121454133 | 121454451 | PDFSTATSENSEMBLUCSC | 319 | 8 | 10905 | island | 0.004157 | 0.0018619 | Not queried | 1.59 | 3.28e-01 | 0.4672865596 |
|  | 11 | 117352692 | 117352939 | PDFSTATSENSEMBLUCSC | 248 | 5 | 6920 | gene;island | 0.642081 | 0.8963945 | Not queried | 1.58 | 1.51e-01 | 0.3618000441 |
|  | 4 | 618114 | 618549 | PDFSTATSENSEMBLUCSC | 436 | 5 | 30225 | tss;island | 0.167817 | 0.6198074 | Not queried | 1.58 | 6.90e-01 | 0.7223604052 |
|  | 22 | 19743759 | 19744078 | PDFSTATSENSEMBLUCSC | 320 | 7 | 26965 | tss | 0.374111 | 0.5248242 | Not queried | 1.58 | 3.83e-01 | 0.5168893301 |
|  | 3 | 32509018 | 32509543 | PDFSTATSENSEMBLUCSC | 526 | 9 | 28964 | island | 0.048102 | 0.0359746 | Not queried | 1.58 | 4.89e-01 | 0.5677586445 |
|  | 13 | 110918331 | 110918683 | PDFSTATSENSEMBLUCSC | 353 | 4 | 11227 | gene;island | 0.520169 | 0.8565828 | Not queried | 1.58 | 3.43e-01 | 0.4672865596 |
|  | 1 | 206729034 | 206729258 | PDFSTATSENSEMBLUCSC | 225 | 4 | 971 | gene;tss;island | 0.378528 | 0.6699104 | Not queried | 1.58 | 4.86e-01 | 0.5640816327 |
|  | 7 | 99067116 | 99067368 | PDFSTATSENSEMBLUCSC | 253 | 5 | 38733 | island | 0.305976 | 0.4635624 | Not queried | 1.58 | 3.10e-01 | 0.4672865596 |
|  | 14 | 105044984 | 105045347 | PDFSTATSENSEMBLUCSC | 364 | 4 | 12658 | tss;island | 0.233221 | 0.3713450 | Not queried | 1.58 | 3.43e-01 | 0.4672865596 |
|  | 17 | 39743164 | 39743427 | PDFSTATSENSEMBLUCSC | 264 | 4 | 17341 | tss | 0.405378 | 0.6266914 | Not queried | 1.58 | 1.14e-01 | 0.3123457953 |
|  | 1 | 156889826 | 156890244 | PDFSTATSENSEMBLUCSC | 419 | 5 | 2026 | tss;island | 0.405767 | 0.0745629 | Not queried | 1.58 | 3.10e-01 | 0.4672865596 |
|  | 13 | 84456127 | 84456722 | PDFSTATSENSEMBLUCSC | 596 | 9 | 11806 | island | 0.066354 | 0.0369046 | Not queried | 1.58 | 2.58e-01 | 0.4381244265 |
|  | 20 | 709016 | 709392 | PDFSTATSENSEMBLUCSC | 377 | 4 | 25928 | island | 0.125488 | 0.0311598 | Not queried | 1.58 | 3.43e-01 | 0.4672865596 |
|  | 1 | 12538341 | 12538678 | PDFSTATSENSEMBLUCSC | 338 | 4 | 233 | gene | 0.328798 | 0.4550101 | Not queried | 1.58 | 1.00e+00 | 1.0000000000 |
|  | 16 | 74331461 | 74331660 | PDFSTATSENSEMBLUCSC | 200 | 5 | 14840 | gene;island | 0.125896 | 0.1247919 | Not queried | 1.58 | 8.41e-01 | 0.8635565145 |
|  | 3 | 49756301 | 49757476 | PDFSTATSENSEMBLUCSC | 1176 | 12 | 27788 | gene;island | 0.140171 | 0.3716208 | Not queried | 1.58 | 2.91e-01 | 0.4672865596 |
|  | 14 | 24640947 | 24641201 | PDFSTATSENSEMBLUCSC | 255 | 9 | 12318 | tss;island | 0.035313 | 0.1387179 | Not queried | 1.58 | 1.88e-02 | 0.1830867741 |
|  | 5 | 2008188 | 2008364 | PDFSTATSENSEMBLUCSC | 177 | 4 | 32650 | island | 0.656038 | 0.3570834 | Not queried | 1.58 | 2.00e-01 | 0.3826488233 |
|  | 10 | 111216646 | 111216962 | PDFSTATSENSEMBLUCSC | 317 | 5 | 6122 | island | 0.236717 | 0.0671713 | Not queried | 1.58 | 3.10e-01 | 0.4672865596 |
|  | 19 | 7852051 | 7852238 | PDFSTATSENSEMBLUCSC | 188 | 6 | 20407 | tss;island | 0.337932 | 0.6912196 | Not queried | 1.58 | 3.10e-01 | 0.4672865596 |
|  | 1 | 21044031 | 21044207 | PDFSTATSENSEMBLUCSC | 177 | 4 | 294 | gene | 0.139701 | 0.0230850 | Not queried | 1.58 | 6.86e-01 | 0.7223604052 |
|  | 2 | 203499669 | 203499802 | PDFSTATSENSEMBLUCSC | 134 | 4 | 23826 | tss;island | 0.012250 | 0.0086901 | Not queried | 1.58 | 8.86e-01 | 0.8941250130 |
|  | 17 | 19651881 | 19652056 | PDFSTATSENSEMBLUCSC | 176 | 4 | 17195 | tss;island | 0.421379 | 0.2177605 | Not queried | 1.58 | 3.43e-01 | 0.4672865596 |
|  | 6 | 24646213 | 24646782 | PDFSTATSENSEMBLUCSC | 570 | 8 | 35494 | island | 0.014530 | 0.0047086 | Not queried | 1.58 | 1.95e-01 | 0.3826488233 |
|  | 1 | 2391121 | 2391347 | PDFSTATSENSEMBLUCSC | 227 | 4 | 2503 | island | 0.607213 | 0.6577949 | Not queried | 1.58 | 8.86e-01 | 0.8941250130 |
|  | 12 | 85673860 | 85674022 | PDFSTATSENSEMBLUCSC | 163 | 6 | 9901 | tss | 0.087387 | 0.0305350 | Not queried | 1.58 | 2.40e-01 | 0.4133265075 |
|  | 11 | 70961596 | 70961902 | PDFSTATSENSEMBLUCSC | 307 | 5 | 8616 | island | 0.111143 | 0.0122260 | Not queried | 1.58 | 9.52e-02 | 0.3104222821 |
|  | 17 | 72427538 | 72427637 | PDFSTATSENSEMBLUCSC | 100 | 5 | 17626 | tss | 0.027744 | 0.1058295 | Not queried | 1.58 | 5.56e-02 | 0.2315877671 |
|  | 17 | 1810459 | 1810853 | PDFSTATSENSEMBLUCSC | 395 | 5 | 17832 | island | 0.591030 | 0.9212628 | Not queried | 1.57 | 5.56e-02 | 0.2315877671 |
|  | 8 | 58907018 | 58907091 | PDFSTATSENSEMBLUCSC | 74 | 4 | 39718 | tss | 0.007467 | 0.0201738 | Not queried | 1.57 | 2.00e-01 | 0.3826488233 |
|  | 8 | 110657174 | 110657197 | PDFSTATSENSEMBLUCSC | 24 | 5 | 39873 | tss | 0.001345 | 0.0032085 | Not queried | 1.57 | 8.41e-01 | 0.8635565145 |
|  | 11 | 66059064 | 66059164 | PDFSTATSENSEMBLUCSC | 101 | 4 | 7463 | tss;island | 0.275378 | 0.5311161 | Not queried | 1.57 | 2.00e-01 | 0.3826488233 |
|  | 2 | 236826207 | 236826485 | PDFSTATSENSEMBLUCSC | 279 | 5 | 23084 | gene | 0.444953 | 0.5626523 | Not queried | 1.57 | 6.90e-01 | 0.7223604052 |
|  | 2 | 133402827 | 133403009 | PDFSTATSENSEMBLUCSC | 183 | 4 | 22877 | gene;island | 0.795915 | 0.9414569 | Not queried | 1.57 | 6.86e-01 | 0.7223604052 |
|  | 6 | 170615736 | 170615780 | PDFSTATSENSEMBLUCSC | 45 | 5 | 35356 | tss | 0.001525 | 0.0030563 | Not queried | 1.57 | 3.10e-01 | 0.4672865596 |
|  | 19 | 38210543 | 38210932 | PDFSTATSENSEMBLUCSC | 390 | 6 | 21961 | island | 0.026777 | 0.0107201 | Not queried | 1.57 | 2.60e-02 | 0.1830867741 |
|  | 15 | 79574773 | 79575048 | PDFSTATSENSEMBLUCSC | 276 | 5 | 13843 | tss;island | 0.011688 | 0.0185231 | Not queried | 1.57 | 4.21e-01 | 0.5404252807 |
|  | 20 | 9494838 | 9495199 | PDFSTATSENSEMBLUCSC | 362 | 5 | 25592 | tss | 0.012678 | 0.0075208 | Not queried | 1.57 | 5.48e-01 | 0.6179718485 |
|  | 6 | 127840517 | 127840755 | PDFSTATSENSEMBLUCSC | 239 | 8 | 35217 | tss | 0.053568 | 0.1629892 | Not queried | 1.57 | 3.82e-01 | 0.5168893301 |
|  | 11 | 103908255 | 103908576 | PDFSTATSENSEMBLUCSC | 322 | 4 | 6879 | gene;island | 0.507063 | 0.7906644 | Not queried | 1.57 | 2.00e-01 | 0.3826488233 |
|  | 7 | 5535582 | 5535934 | PDFSTATSENSEMBLUCSC | 353 | 7 | 37423 | tss | 0.723852 | 0.9689442 | Not queried | 1.57 | 1.65e-01 | 0.3826488233 |
|  | 8 | 23563859 | 23564294 | PDFSTATSENSEMBLUCSC | 436 | 9 | 40156 | island | 0.274980 | 0.0783536 | Not queried | 1.57 | 5.03e-02 | 0.2315877671 |
|  | 10 | 38146893 | 38147123 | PDFSTATSENSEMBLUCSC | 231 | 4 | 5033 | tss;island | 0.045949 | 0.0122500 | Not queried | 1.57 | 1.14e-01 | 0.3123457953 |
|  | 17 | 15686294 | 15686473 | PDFSTATSENSEMBLUCSC | 180 | 4 | 18043 | island | 0.053614 | 0.1354625 | Not queried | 1.57 | 4.86e-01 | 0.5640816327 |
|  | 19 | 14591033 | 14591345 | PDFSTATSENSEMBLUCSC | 313 | 5 | 19894 | gene;island | 0.045884 | 0.3373916 | Not queried | 1.57 | 6.90e-01 | 0.7223604052 |
|  | 16 | 89002930 | 89003235 | PDFSTATSENSEMBLUCSC | 306 | 4 | 14925 | gene;island | 0.049320 | 0.1657764 | Not queried | 1.57 | 3.43e-01 | 0.4672865596 |
|  | 7 | 157561593 | 157561925 | PDFSTATSENSEMBLUCSC | 333 | 5 | 37343 | gene;island | 0.474082 | 0.7066988 | Not queried | 1.57 | 6.90e-01 | 0.7223604052 |
|  | 16 | 216450 | 216757 | PDFSTATSENSEMBLUCSC | 308 | 4 | 14523 | gene;island | 0.036276 | 0.1161318 | Not queried | 1.57 | 1.14e-01 | 0.3123457953 |
|  | 6 | 143832855 | 143833251 | PDFSTATSENSEMBLUCSC | 397 | 8 | 36529 | island | 0.302293 | 0.0777072 | Not queried | 1.57 | 3.82e-01 | 0.5168893301 |
|  | 11 | 115374969 | 115375432 | PDFSTATSENSEMBLUCSC | 464 | 8 | 8828 | island | 0.049250 | 0.1215208 | Not queried | 1.57 | 3.82e-01 | 0.5168893301 |
|  | 2 | 109746691 | 109746754 | PDFSTATSENSEMBLUCSC | 64 | 4 | 22823 | gene;tss;island | 0.803016 | 0.5890672 | Not queried | 1.57 | 4.86e-01 | 0.5640816327 |
|  | 1 | 39281613 | 39282003 | PDFSTATSENSEMBLUCSC | 391 | 4 | 3091 | island | 0.039820 | 0.0130205 | Not queried | 1.57 | 4.86e-01 | 0.5640816327 |
|  | 13 | 111367171 | 111367259 | PDFSTATSENSEMBLUCSC | 89 | 5 | 11240 | gene;tss;island | 0.008566 | 0.0216853 | Not queried | 1.57 | 8.41e-01 | 0.8635565145 |
|  | 12 | 6881590 | 6881629 | PDFSTATSENSEMBLUCSC | 40 | 5 | 9527 | tss;island | 0.314840 | 0.4691816 | Not queried | 1.57 | 5.56e-02 | 0.2315877671 |
|  | 19 | 55417361 | 55417500 | PDFSTATSENSEMBLUCSC | 140 | 5 | 21066 | tss | 0.087583 | 0.3331102 | Not queried | 1.57 | 5.48e-01 | 0.6179718485 |
|  | 6 | 30859697 | 30860529 | PDFSTATSENSEMBLUCSC | 833 | 15 | 33828 | gene | 0.955452 | 0.8242029 | Not queried | 1.57 | 3.67e-02 | 0.2121545571 |
|  | 2 | 223290012 | 223290515 | PDFSTATSENSEMBLUCSC | 504 | 4 | 23053 | gene;island | 0.005273 | 0.0147836 | Not queried | 1.57 | 3.43e-01 | 0.4672865596 |
|  | 10 | 75757809 | 75757817 | PDFSTATSENSEMBLUCSC | 9 | 4 | 5167 | tss | 0.005766 | 0.0023344 | Not queried | 1.57 | 6.86e-01 | 0.7223604052 |
|  | 17 | 34062219 | 34062573 | PDFSTATSENSEMBLUCSC | 355 | 5 | 16524 | gene;island | 0.690677 | 0.9130124 | Not queried | 1.57 | 2.22e-01 | 0.3935758299 |
|  | 20 | 45523098 | 45523187 | PDFSTATSENSEMBLUCSC | 90 | 4 | 25798 | tss | 0.005030 | 0.0203687 | Not queried | 1.56 | 3.43e-01 | 0.4672865596 |
|  | 13 | 113140125 | 113140323 | PDFSTATSENSEMBLUCSC | 199 | 4 | 11247 | gene;island | 0.858169 | 0.9404600 | Not queried | 1.56 | 3.43e-01 | 0.4672865596 |
|  | 5 | 65222276 | 65222522 | PDFSTATSENSEMBLUCSC | 247 | 5 | 32840 | island | 0.002963 | 0.0072061 | Not queried | 1.56 | 8.41e-01 | 0.8635565145 |
|  | 8 | 11203882 | 11204132 | PDFSTATSENSEMBLUCSC | 251 | 4 | 39204 | gene;island | 0.636315 | 0.5242486 | Not queried | 1.56 | 6.86e-01 | 0.7223604052 |
|  | 12 | 104609609 | 104610064 | PDFSTATSENSEMBLUCSC | 456 | 4 | 9271 | gene | 0.113607 | 0.0375411 | Not queried | 1.56 | 4.86e-01 | 0.5640816327 |
|  | 12 | 22093960 | 22094750 | PDFSTATSENSEMBLUCSC | 791 | 10 | 10281 | island | 0.092075 | 0.0245975 | Not queried | 1.56 | 7.53e-02 | 0.2795801356 |
|  | 8 | 71581111 | 71581445 | PDFSTATSENSEMBLUCSC | 335 | 4 | 39333 | gene | 0.006272 | 0.0059194 | Not queried | 1.56 | 6.86e-01 | 0.7223604052 |
|  | 1 | 44063475 | 44063744 | PDFSTATSENSEMBLUCSC | 270 | 4 | 482 | gene;island | 0.624604 | 0.9155978 | Not queried | 1.56 | 3.43e-01 | 0.4672865596 |
|  | 5 | 151066268 | 151066486 | PDFSTATSENSEMBLUCSC | 219 | 4 | 31867 | gene | 0.046169 | 0.0125364 | Not queried | 1.56 | 5.71e-02 | 0.2315877671 |
|  | 6 | 10419684 | 10420079 | PDFSTATSENSEMBLUCSC | 396 | 8 | 35432 | island | 0.287516 | 0.0893138 | Not queried | 1.56 | 1.95e-01 | 0.3826488233 |
|  | 2 | 235406275 | 235406540 | PDFSTATSENSEMBLUCSC | 266 | 4 | 23961 | tss;island | 0.511484 | 0.7175599 | Not queried | 1.56 | 6.86e-01 | 0.7223604052 |
|  | 12 | 132900274 | 132901088 | PDFSTATSENSEMBLUCSC | 815 | 7 | 9448 | gene;island | 0.470661 | 0.5935848 | Not queried | 1.56 | 2.59e-01 | 0.4381244265 |
|  | 8 | 2046752 | 2047010 | PDFSTATSENSEMBLUCSC | 259 | 4 | 39188 | gene;island | 0.668643 | 0.8512175 | Not queried | 1.56 | 3.43e-01 | 0.4672865596 |
|  | 19 | 13985408 | 13985688 | PDFSTATSENSEMBLUCSC | 281 | 5 | 21661 | island | 0.504071 | 0.6385437 | Not queried | 1.56 | 5.48e-01 | 0.6179718485 |
|  | 19 | 19322527 | 19322778 | PDFSTATSENSEMBLUCSC | 252 | 6 | 20639 | tss | 0.008801 | 0.0236959 | Not queried | 1.56 | 3.10e-01 | 0.4672865596 |
|  | 4 | 1052372 | 1052717 | PDFSTATSENSEMBLUCSC | 346 | 4 | 30766 | island | 0.391802 | 0.5614881 | Not queried | 1.56 | 3.43e-01 | 0.4672865596 |
|  | 11 | 82444946 | 82445170 | PDFSTATSENSEMBLUCSC | 225 | 4 | 7594 | tss | 0.176924 | 0.3822536 | Not queried | 1.56 | 4.86e-01 | 0.5640816327 |
|  | 2 | 23840537 | 23840763 | PDFSTATSENSEMBLUCSC | 227 | 4 | 22594 | gene | 0.933156 | 0.7497132 | Not queried | 1.56 | 2.00e-01 | 0.3826488233 |
|  | 1 | 151966792 | 151967023 | PDFSTATSENSEMBLUCSC | 232 | 5 | 1935 | tss;island | 0.069073 | 0.0434220 | Not queried | 1.56 | 6.90e-01 | 0.7223604052 |
|  | 1 | 39956641 | 39956807 | PDFSTATSENSEMBLUCSC | 167 | 4 | 1522 | tss;island | 0.190040 | 0.0226243 | Not queried | 1.56 | 1.14e-01 | 0.3123457953 |
|  | 7 | 134855640 | 134855774 | PDFSTATSENSEMBLUCSC | 135 | 8 | 37916 | tss | 0.001306 | 0.0061692 | Not queried | 1.56 | 3.28e-01 | 0.4672865596 |
|  | 12 | 64784070 | 64784252 | PDFSTATSENSEMBLUCSC | 183 | 4 | 9203 | gene;island | 0.330510 | 0.1360359 | Not queried | 1.56 | 6.86e-01 | 0.7223604052 |
|  | 11 | 15136059 | 15136688 | PDFSTATSENSEMBLUCSC | 630 | 9 | 6521 | gene;island | 0.084600 | 0.3242095 | Not queried | 1.56 | 1.90e-01 | 0.3826488233 |
|  | 6 | 168920172 | 168920331 | PDFSTATSENSEMBLUCSC | 160 | 4 | 34561 | gene;island | 0.642838 | 0.7164699 | Not queried | 1.56 | 6.86e-01 | 0.7223604052 |
|  | 4 | 8153998 | 8154405 | PDFSTATSENSEMBLUCSC | 408 | 5 | 29948 | gene;island | 0.849063 | 0.8003628 | Not queried | 1.56 | 6.90e-01 | 0.7223604052 |
|  | 21 | 45773189 | 45773400 | PDFSTATSENSEMBLUCSC | 212 | 4 | 26584 | tss | 0.028778 | 0.2094148 | Not queried | 1.56 | 4.86e-01 | 0.5640816327 |
|  | 4 | 164253207 | 164253656 | PDFSTATSENSEMBLUCSC | 450 | 4 | 30167 | gene | 0.090709 | 0.0216878 | Not queried | 1.56 | 2.00e-01 | 0.3826488233 |
|  | 7 | 50800837 | 50801423 | PDFSTATSENSEMBLUCSC | 587 | 8 | 37014 | gene;tss | 0.458144 | 0.7675301 | Not queried | 1.56 | 2.79e-01 | 0.4672865596 |
|  | 10 | 134049464 | 134049802 | PDFSTATSENSEMBLUCSC | 339 | 5 | 4859 | gene | 0.864461 | 0.8584882 | Not queried | 1.56 | 5.48e-01 | 0.6179718485 |
|  | 11 | 89224288 | 89224799 | PDFSTATSENSEMBLUCSC | 512 | 10 | 6857 | gene;island | 0.071967 | 0.0225441 | Not queried | 1.56 | 1.05e-01 | 0.3123457953 |
|  | 11 | 113953462 | 113953838 | PDFSTATSENSEMBLUCSC | 377 | 4 | 6910 | gene;island | 0.015509 | 0.1320434 | Not queried | 1.56 | 4.86e-01 | 0.5640816327 |
|  | 1 | 223888869 | 223889141 | PDFSTATSENSEMBLUCSC | 273 | 4 | 2273 | tss | 0.542113 | 0.7479012 | Not queried | 1.56 | 3.43e-01 | 0.4672865596 |
|  | 8 | 127569581 | 127569677 | PDFSTATSENSEMBLUCSC | 97 | 4 | 39426 | gene;island | 0.061277 | 0.2761344 | Not queried | 1.55 | 2.00e-01 | 0.3826488233 |
|  | 13 | 98605770 | 98605892 | PDFSTATSENSEMBLUCSC | 123 | 4 | 11507 | tss | 0.595759 | 0.7217355 | Not queried | 1.55 | 6.86e-01 | 0.7223604052 |
|  | 8 | 74659776 | 74660304 | PDFSTATSENSEMBLUCSC | 529 | 8 | 39769 | tss;island | 0.022201 | 0.0175204 | Not queried | 1.55 | 5.74e-01 | 0.6460388787 |
|  | 8 | 23261618 | 23262159 | PDFSTATSENSEMBLUCSC | 542 | 9 | 40151 | island | 0.031657 | 0.1645760 | Not queried | 1.55 | 1.36e-01 | 0.3491249184 |
|  | 14 | 101498206 | 101498317 | PDFSTATSENSEMBLUCSC | 112 | 5 | 12623 | tss | 0.669605 | 0.9089989 | Not queried | 1.55 | 2.22e-01 | 0.3935758299 |
|  | 16 | 2765620 | 2765900 | PDFSTATSENSEMBLUCSC | 281 | 5 | 14610 | gene;island | 0.067689 | 0.0212775 | Not queried | 1.55 | 9.52e-02 | 0.3104222821 |
|  | 4 | 140586921 | 140587179 | PDFSTATSENSEMBLUCSC | 259 | 6 | 30132 | gene | 0.020444 | 0.0117572 | Not queried | 1.55 | 3.94e-01 | 0.5236526859 |
|  | 1 | 236445668 | 236445970 | PDFSTATSENSEMBLUCSC | 303 | 4 | 2342 | tss;island | 0.008587 | 0.0146609 | Not queried | 1.55 | 8.86e-01 | 0.8941250130 |
|  | 13 | 25861182 | 25861849 | PDFSTATSENSEMBLUCSC | 668 | 6 | 11611 | island | 0.002223 | 0.0086433 | Not queried | 1.55 | 2.40e-01 | 0.4133265075 |
|  | 12 | 7023195 | 7023527 | PDFSTATSENSEMBLUCSC | 333 | 9 | 9534 | tss;island | 0.407433 | 0.0179502 | Not queried | 1.55 | 2.97e-01 | 0.4672865596 |
|  | 7 | 157691177 | 157691684 | PDFSTATSENSEMBLUCSC | 508 | 6 | 37351 | gene;island | 0.146390 | 0.2970764 | Not queried | 1.55 | 3.94e-01 | 0.5236526859 |
|  | 14 | 105352845 | 105353169 | PDFSTATSENSEMBLUCSC | 325 | 4 | 12253 | gene;island | 0.441015 | 0.6553293 | Not queried | 1.55 | 2.86e-02 | 0.1830867741 |
|  | 6 | 32025416 | 32025657 | PDFSTATSENSEMBLUCSC | 242 | 4 | 34021 | gene | 0.113787 | 0.3286048 | Not queried | 1.55 | 3.43e-01 | 0.4672865596 |
|  | 16 | 2892518 | 2892994 | PDFSTATSENSEMBLUCSC | 477 | 8 | 15620 | island | 0.525370 | 0.4220178 | Not queried | 1.55 | 5.05e-01 | 0.5852592101 |
|  | 16 | 2892766 | 2892994 | PDFSTATSENSEMBLUCSC | 229 | 6 | 15038 | tss | 0.525370 | 0.1983996 | Not queried | 1.55 | 5.89e-01 | 0.6570484697 |
|  | 10 | 103985730 | 103986268 | PDFSTATSENSEMBLUCSC | 539 | 9 | 6080 | island | 0.014656 | 0.0180248 | Not queried | 1.55 | 2.22e-01 | 0.3935758299 |
|  | 16 | 67678593 | 67678846 | PDFSTATSENSEMBLUCSC | 254 | 5 | 15307 | tss;island | 0.012875 | 0.0097638 | Not queried | 1.55 | 5.48e-01 | 0.6179718485 |
|  | 3 | 69788259 | 69788442 | PDFSTATSENSEMBLUCSC | 184 | 4 | 28441 | tss;island | 0.006195 | 0.0034266 | Not queried | 1.55 | 2.00e-01 | 0.3826488233 |
|  | 6 | 150463775 | 150464265 | PDFSTATSENSEMBLUCSC | 491 | 8 | 36568 | island | 0.046512 | 0.0248429 | Not queried | 1.55 | 2.34e-01 | 0.4128374386 |
|  | 11 | 17565744 | 17565885 | PDFSTATSENSEMBLUCSC | 142 | 4 | 6525 | gene | 0.284984 | 0.0413574 | Not queried | 1.55 | 8.86e-01 | 0.8941250130 |
|  | 12 | 66582582 | 66582805 | PDFSTATSENSEMBLUCSC | 224 | 4 | 9848 | tss;island | 0.331261 | 0.0829447 | Not queried | 1.55 | 3.43e-01 | 0.4672865596 |
|  | 14 | 77737661 | 77737800 | PDFSTATSENSEMBLUCSC | 140 | 4 | 12529 | tss | 0.371830 | 0.2705120 | Not queried | 1.55 | 4.86e-01 | 0.5640816327 |
|  | 8 | 140716970 | 140717372 | PDFSTATSENSEMBLUCSC | 403 | 6 | 40606 | island | 0.046790 | 0.3058538 | Not queried | 1.55 | 3.10e-01 | 0.4672865596 |
|  | 5 | 176873332 | 176873656 | PDFSTATSENSEMBLUCSC | 325 | 6 | 32528 | tss | 0.003212 | 0.0124995 | Not queried | 1.55 | 2.40e-01 | 0.4133265075 |
|  | 4 | 184922382 | 184922646 | PDFSTATSENSEMBLUCSC | 265 | 5 | 30197 | gene | 0.403547 | 0.5988744 | Not queried | 1.55 | 5.48e-01 | 0.6179718485 |
|  | 7 | 87936225 | 87936414 | PDFSTATSENSEMBLUCSC | 190 | 5 | 37691 | tss | 0.036441 | 0.0459499 | Not queried | 1.55 | 4.21e-01 | 0.5404252807 |
|  | 12 | 51236582 | 51236768 | PDFSTATSENSEMBLUCSC | 187 | 6 | 10418 | island | 0.526950 | 0.8258437 | Not queried | 1.55 | 3.10e-01 | 0.4672865596 |
|  | 2 | 88849457 | 88849830 | PDFSTATSENSEMBLUCSC | 374 | 4 | 24503 | island | 0.543745 | 0.6334495 | Not queried | 1.55 | 6.86e-01 | 0.7223604052 |
|  | 7 | 4308120 | 4308449 | PDFSTATSENSEMBLUCSC | 330 | 6 | 36839 | gene;island | 0.367527 | 0.5334327 | Not queried | 1.54 | 2.40e-01 | 0.4133265075 |
|  | 13 | 28674720 | 28675056 | PDFSTATSENSEMBLUCSC | 337 | 5 | 11637 | island | 0.008420 | 0.0209545 | Not queried | 1.54 | 2.22e-01 | 0.3935758299 |
|  | 2 | 71205052 | 71205564 | PDFSTATSENSEMBLUCSC | 513 | 6 | 23424 | tss | 0.044843 | 0.0123538 | Not queried | 1.54 | 4.85e-01 | 0.5640816327 |
|  | 8 | 48100868 | 48100957 | PDFSTATSENSEMBLUCSC | 90 | 4 | 40270 | island | 0.264169 | 0.6908387 | Not queried | 1.54 | 3.43e-01 | 0.4672865596 |
|  | 16 | 88937115 | 88937386 | PDFSTATSENSEMBLUCSC | 272 | 5 | 16226 | island | 0.296307 | 0.0858042 | Not queried | 1.54 | 6.90e-01 | 0.7223604052 |
|  | 7 | 75932840 | 75932977 | PDFSTATSENSEMBLUCSC | 138 | 6 | 37053 | gene;island | 0.004235 | 0.0063283 | Not queried | 1.54 | 3.94e-01 | 0.5236526859 |
|  | 20 | 18568287 | 18568549 | PDFSTATSENSEMBLUCSC | 263 | 7 | 25620 | tss | 0.004565 | 0.0018213 | Not queried | 1.54 | 1.28e-01 | 0.3397986247 |
|  | 20 | 18568287 | 18568595 | PDFSTATSENSEMBLUCSC | 309 | 9 | 26030 | island | 0.004565 | 0.0018213 | Not queried | 1.54 | 1.61e-01 | 0.3826488233 |
|  | 20 | 39995539 | 39995747 | PDFSTATSENSEMBLUCSC | 209 | 7 | 25746 | tss;island | 0.040089 | 0.0268830 | Not queried | 1.54 | 7.10e-01 | 0.7390730975 |
|  | 4 | 89299733 | 89300321 | PDFSTATSENSEMBLUCSC | 589 | 7 | 31173 | island | 0.009337 | 0.0247208 | Not queried | 1.54 | 3.18e-01 | 0.4672865596 |
|  | 7 | 150147988 | 150148073 | PDFSTATSENSEMBLUCSC | 86 | 4 | 37274 | gene | 0.026871 | 0.0792623 | Not queried | 1.54 | 5.71e-02 | 0.2315877671 |
|  | 14 | 81421983 | 81422160 | PDFSTATSENSEMBLUCSC | 178 | 5 | 12161 | gene;island | 0.200536 | 0.5009834 | Not queried | 1.54 | 1.51e-01 | 0.3618000441 |
|  | 1 | 32930375 | 32930544 | PDFSTATSENSEMBLUCSC | 170 | 6 | 1461 | tss;island | 0.022654 | 0.0292741 | Not queried | 1.54 | 2.40e-01 | 0.4133265075 |
|  | 16 | 1728187 | 1728262 | PDFSTATSENSEMBLUCSC | 76 | 5 | 15001 | tss | 0.003376 | 0.0003365 | Not queried | 1.54 | 2.22e-01 | 0.3935758299 |
|  | 17 | 74099707 | 74100207 | PDFSTATSENSEMBLUCSC | 501 | 13 | 17671 | tss;island | 0.006254 | 0.0054015 | Not queried | 1.54 | 5.45e-01 | 0.6179718485 |
|  | 2 | 79739863 | 79739940 | PDFSTATSENSEMBLUCSC | 78 | 4 | 23472 | tss;island | 0.032239 | 0.0943497 | Not queried | 1.54 | 3.43e-01 | 0.4672865596 |
|  | 17 | 80291982 | 80292252 | PDFSTATSENSEMBLUCSC | 271 | 4 | 17773 | tss;island | 0.477117 | 0.6125284 | Not queried | 1.54 | 6.86e-01 | 0.7223604052 |
|  | 4 | 7436701 | 7437156 | PDFSTATSENSEMBLUCSC | 456 | 5 | 30292 | tss | 0.044072 | 0.3291283 | Not queried | 1.54 | 2.22e-01 | 0.3935758299 |
|  | 12 | 48577720 | 48578223 | PDFSTATSENSEMBLUCSC | 504 | 4 | 9111 | gene;island | 0.073045 | 0.0153400 | Not queried | 1.54 | 3.43e-01 | 0.4672865596 |
|  | 12 | 75785089 | 75785295 | PDFSTATSENSEMBLUCSC | 207 | 5 | 9222 | gene;island | 0.002679 | 0.0055901 | Not queried | 1.54 | 1.51e-01 | 0.3618000441 |
|  | 14 | 90526639 | 90526912 | PDFSTATSENSEMBLUCSC | 274 | 4 | 12551 | tss;island | 0.148133 | 0.0178751 | Not queried | 1.54 | 3.43e-01 | 0.4672865596 |
|  | 10 | 21783445 | 21783930 | PDFSTATSENSEMBLUCSC | 486 | 5 | 4525 | gene | 0.004842 | 0.0169939 | Not queried | 1.54 | 9.52e-02 | 0.3104222821 |
|  | 16 | 711015 | 711235 | PDFSTATSENSEMBLUCSC | 221 | 4 | 14535 | gene;island | 0.120263 | 0.3100079 | Not queried | 1.54 | 4.86e-01 | 0.5640816327 |
|  | 3 | 62861055 | 62861311 | PDFSTATSENSEMBLUCSC | 257 | 7 | 29252 | island | 0.045186 | 0.0448052 | Not queried | 1.54 | 5.35e-01 | 0.6176455124 |
|  | 1 | 1998716 | 1999183 | PDFSTATSENSEMBLUCSC | 468 | 4 | 55 | gene | 0.667227 | 0.9229309 | Not queried | 1.54 | 6.86e-01 | 0.7223604052 |
|  | 2 | 112944644 | 112944999 | PDFSTATSENSEMBLUCSC | 356 | 4 | 22828 | gene;island | 0.881126 | 0.9267742 | Not queried | 1.54 | 4.86e-01 | 0.5640816327 |
|  | 1 | 39269995 | 39270310 | PDFSTATSENSEMBLUCSC | 316 | 4 | 3090 | island | 0.032091 | 0.0846199 | Not queried | 1.54 | 2.00e-01 | 0.3826488233 |
|  | 10 | 71811927 | 71812332 | PDFSTATSENSEMBLUCSC | 406 | 9 | 5129 | tss;island | 0.123161 | 0.0313450 | Not queried | 1.54 | 9.39e-02 | 0.3104222821 |
|  | 4 | 111544271 | 111544463 | PDFSTATSENSEMBLUCSC | 193 | 7 | 30549 | tss | 0.070709 | 0.0264424 | Not queried | 1.54 | 1.75e-02 | 0.1830867741 |
|  | 8 | 88886301 | 88886432 | PDFSTATSENSEMBLUCSC | 132 | 6 | 39801 | tss | 0.674475 | 0.9611853 | Not queried | 1.54 | 9.31e-02 | 0.3104222821 |
|  | 7 | 4901628 | 4901798 | PDFSTATSENSEMBLUCSC | 171 | 4 | 37417 | tss | 0.572535 | 0.4919895 | Not queried | 1.54 | 1.00e+00 | 1.0000000000 |
|  | 7 | 544525 | 544976 | PDFSTATSENSEMBLUCSC | 452 | 6 | 36706 | gene | 0.790630 | 0.8970978 | Not queried | 1.54 | 5.89e-01 | 0.6570484697 |
|  | 8 | 108510286 | 108510343 | PDFSTATSENSEMBLUCSC | 58 | 4 | 39865 | tss | 0.015780 | 0.0089436 | Not queried | 1.54 | 6.86e-01 | 0.7223604052 |
|  | 4 | 5890358 | 5890430 | PDFSTATSENSEMBLUCSC | 73 | 6 | 30277 | tss | 0.022719 | 0.0096651 | Not queried | 1.54 | 3.10e-01 | 0.4672865596 |
|  | 15 | 41803428 | 41803850 | PDFSTATSENSEMBLUCSC | 423 | 4 | 13304 | gene;island | 0.298159 | 0.0486081 | Not queried | 1.54 | 3.43e-01 | 0.4672865596 |
|  | 8 | 96037118 | 96037199 | PDFSTATSENSEMBLUCSC | 82 | 5 | 39822 | tss;island | 0.007217 | 0.0097894 | Not queried | 1.54 | 6.90e-01 | 0.7223604052 |
|  | 12 | 6716557 | 6716597 | PDFSTATSENSEMBLUCSC | 41 | 4 | 9518 | tss;island | 0.009914 | 0.0041204 | Not queried | 1.53 | 4.86e-01 | 0.5640816327 |
|  | 11 | 2828214 | 2828778 | PDFSTATSENSEMBLUCSC | 565 | 7 | 6467 | gene;island | 0.231517 | 0.5134463 | Not queried | 1.53 | 9.73e-02 | 0.3123457953 |
|  | 6 | 33165404 | 33165899 | PDFSTATSENSEMBLUCSC | 496 | 9 | 34219 | gene;island | 0.631216 | 0.9201609 | Not queried | 1.53 | 2.22e-01 | 0.3935758299 |
|  | 15 | 68723690 | 68724065 | PDFSTATSENSEMBLUCSC | 376 | 4 | 13396 | gene;island | 0.029782 | 0.0296511 | Not queried | 1.53 | 8.86e-01 | 0.8941250130 |
|  | 11 | 118754280 | 118754535 | PDFSTATSENSEMBLUCSC | 256 | 5 | 7739 | tss | 0.005439 | 0.0040741 | Not queried | 1.53 | 6.90e-01 | 0.7223604052 |
|  | 4 | 3742095 | 3742357 | PDFSTATSENSEMBLUCSC | 263 | 5 | 30852 | island | 0.411660 | 0.6692105 | Not queried | 1.53 | 7.94e-03 | 0.1589602024 |
|  | 19 | 35739448 | 35739762 | PDFSTATSENSEMBLUCSC | 315 | 5 | 21900 | island | 0.007669 | 0.0095460 | Not queried | 1.53 | 3.10e-01 | 0.4672865596 |
|  | 4 | 46911291 | 46911550 | PDFSTATSENSEMBLUCSC | 260 | 5 | 30374 | tss | 0.503274 | 0.8571869 | Not queried | 1.53 | 5.48e-01 | 0.6179718485 |
|  | 7 | 95025611 | 95026248 | PDFSTATSENSEMBLUCSC | 638 | 20 | 38678 | island | 0.109497 | 0.2778207 | Not queried | 1.53 | 3.50e-02 | 0.2050200525 |
|  | 5 | 1409773 | 1410090 | PDFSTATSENSEMBLUCSC | 318 | 4 | 31537 | gene | 0.157161 | 0.3787684 | Not queried | 1.53 | 2.00e-01 | 0.3826488233 |
|  | 19 | 5245891 | 5246048 | PDFSTATSENSEMBLUCSC | 158 | 4 | 19748 | gene;island | 0.544990 | 0.7882884 | Not queried | 1.53 | 2.00e-01 | 0.3826488233 |
|  | 10 | 27547536 | 27547965 | PDFSTATSENSEMBLUCSC | 430 | 6 | 5628 | island | 0.049606 | 0.0373044 | Not queried | 1.53 | 3.10e-01 | 0.4672865596 |
|  | 8 | 23082233 | 23082634 | PDFSTATSENSEMBLUCSC | 402 | 4 | 39240 | gene | 0.006254 | 0.0090790 | Not queried | 1.53 | 4.86e-01 | 0.5640816327 |
|  | 3 | 138725153 | 138725425 | PDFSTATSENSEMBLUCSC | 273 | 7 | 28627 | tss;island | 0.940352 | 0.8682768 | Not queried | 1.53 | 9.02e-01 | 0.9091904204 |
|  | 20 | 43029468 | 43029895 | PDFSTATSENSEMBLUCSC | 428 | 5 | 25759 | tss | 0.261912 | 0.4275095 | Not queried | 1.53 | 4.21e-01 | 0.5404252807 |
|  | 10 | 29827500 | 29827742 | PDFSTATSENSEMBLUCSC | 243 | 7 | 4546 | gene | 0.921847 | 0.9403986 | Not queried | 1.53 | 3.18e-01 | 0.4672865596 |
|  | 17 | 4802692 | 4803053 | PDFSTATSENSEMBLUCSC | 362 | 8 | 16359 | gene;island | 0.021502 | 0.0126353 | Not queried | 1.53 | 1.61e-01 | 0.3814043454 |
|  | 4 | 154710353 | 154711675 | PDFSTATSENSEMBLUCSC | 1323 | 37 | 30643 | tss | 0.020115 | 0.0733571 | Not queried | 1.53 | 2.13e-02 | 0.1830867741 |
|  | 4 | 140374776 | 140375014 | PDFSTATSENSEMBLUCSC | 239 | 4 | 31318 | island | 0.011884 | 0.0087259 | Not queried | 1.53 | 3.43e-01 | 0.4672865596 |
|  | 6 | 27782484 | 27782658 | PDFSTATSENSEMBLUCSC | 175 | 5 | 34720 | tss;island | 0.007316 | 0.0045772 | Not queried | 1.53 | 4.21e-01 | 0.5404252807 |
|  | 11 | 57414401 | 57414626 | PDFSTATSENSEMBLUCSC | 226 | 4 | 6615 | gene;island | 0.312824 | 0.5100594 | Not queried | 1.53 | 8.86e-01 | 0.8941250130 |
|  | 12 | 101603835 | 101603996 | PDFSTATSENSEMBLUCSC | 162 | 4 | 9267 | gene | 0.140394 | 0.0370625 | Not queried | 1.53 | 4.86e-01 | 0.5640816327 |
|  | 6 | 108496716 | 108497141 | PDFSTATSENSEMBLUCSC | 426 | 4 | 34441 | gene;island | 0.063030 | 0.2494617 | Not queried | 1.53 | 4.86e-01 | 0.5640816327 |
|  | 15 | 33360195 | 33360485 | PDFSTATSENSEMBLUCSC | 291 | 6 | 13554 | tss | 0.061093 | 0.0479924 | Not queried | 1.53 | 5.89e-01 | 0.6570484697 |
|  | 5 | 128300967 | 128301185 | PDFSTATSENSEMBLUCSC | 219 | 6 | 32260 | tss | 0.009455 | 0.0214262 | Not queried | 1.53 | 1.80e-01 | 0.3826488233 |
|  | 5 | 1298511 | 1298965 | PDFSTATSENSEMBLUCSC | 455 | 6 | 32625 | island | 0.568223 | 0.6092532 | Not queried | 1.52 | 1.00e+00 | 1.0000000000 |
|  | 1 | 2984245 | 2984869 | PDFSTATSENSEMBLUCSC | 625 | 9 | 1178 | tss | 0.181684 | 0.4235885 | Not queried | 1.52 | 3.87e-01 | 0.5209559659 |
|  | 14 | 73602752 | 73602980 | PDFSTATSENSEMBLUCSC | 229 | 5 | 12495 | tss;island | 0.035022 | 0.0148698 | Not queried | 1.52 | 5.48e-01 | 0.6179718485 |
|  | 10 | 79793353 | 79793501 | PDFSTATSENSEMBLUCSC | 149 | 7 | 5179 | tss | 0.015386 | 0.0052087 | Not queried | 1.52 | 3.83e-01 | 0.5168893301 |
|  | 19 | 51487109 | 51487594 | PDFSTATSENSEMBLUCSC | 486 | 9 | 20988 | tss | 0.295903 | 0.1600472 | Not queried | 1.52 | 4.36e-01 | 0.5589686471 |
|  | 13 | 20532610 | 20532768 | PDFSTATSENSEMBLUCSC | 159 | 5 | 11326 | tss | 0.005521 | 0.0040927 | Not queried | 1.52 | 4.21e-01 | 0.5404252807 |
|  | 3 | 133748115 | 133748809 | PDFSTATSENSEMBLUCSC | 695 | 7 | 27953 | gene;island | 0.016254 | 0.0187591 | Not queried | 1.52 | 6.20e-01 | 0.6899391537 |
|  | 17 | 40428524 | 40428550 | PDFSTATSENSEMBLUCSC | 27 | 4 | 17359 | tss;island | 0.005994 | 0.0179351 | Not queried | 1.52 | 3.43e-01 | 0.4672865596 |
|  | 10 | 5541568 | 5541823 | PDFSTATSENSEMBLUCSC | 256 | 4 | 4925 | tss | 0.201106 | 0.4613629 | Not queried | 1.52 | 3.43e-01 | 0.4672865596 |
|  | 2 | 155554688 | 155555414 | PDFSTATSENSEMBLUCSC | 727 | 9 | 24797 | island | 0.026418 | 0.1034846 | Not queried | 1.52 | 2.44e-02 | 0.1830867741 |
|  | 4 | 90031538 | 90031969 | PDFSTATSENSEMBLUCSC | 432 | 4 | 31177 | island | 0.329812 | 0.0191679 | Not queried | 1.52 | 4.86e-01 | 0.5640816327 |
|  | 11 | 1083178 | 1083509 | PDFSTATSENSEMBLUCSC | 332 | 6 | 6402 | gene;island | 0.351816 | 0.5159970 | Not queried | 1.52 | 1.80e-01 | 0.3826488233 |
|  | 16 | 1128689 | 1128865 | PDFSTATSENSEMBLUCSC | 177 | 5 | 14987 | tss;island | 0.734262 | 0.8492595 | Not queried | 1.52 | 3.10e-01 | 0.4672865596 |
|  | 18 | 5544031 | 5544237 | PDFSTATSENSEMBLUCSC | 207 | 8 | 19171 | tss | 0.022920 | 0.0818604 | Not queried | 1.52 | 2.34e-01 | 0.4128374386 |
|  | 11 | 65547881 | 65548028 | PDFSTATSENSEMBLUCSC | 148 | 6 | 7440 | tss | 0.009439 | 0.0072226 | Not queried | 1.52 | 4.85e-01 | 0.5640816327 |
|  | 20 | 49620366 | 49620679 | PDFSTATSENSEMBLUCSC | 314 | 4 | 25453 | gene;island | 0.173429 | 0.3859913 | Not queried | 1.52 | 2.00e-01 | 0.3826488233 |
|  | 4 | 166795260 | 166795913 | PDFSTATSENSEMBLUCSC | 654 | 9 | 30173 | gene;island | 0.015088 | 0.0478614 | Not queried | 1.52 | 1.90e-01 | 0.3826488233 |
|  | 1 | 2918373 | 2918752 | PDFSTATSENSEMBLUCSC | 380 | 5 | 2525 | island | 0.400248 | 0.5859395 | Not queried | 1.52 | 1.51e-01 | 0.3618000441 |
|  | 19 | 4785298 | 4785605 | PDFSTATSENSEMBLUCSC | 308 | 4 | 21388 | island | 0.068124 | 0.4465156 | Not queried | 1.52 | 2.00e-01 | 0.3826488233 |
|  | 5 | 169724590 | 169724803 | PDFSTATSENSEMBLUCSC | 214 | 5 | 31889 | gene | 0.008420 | 0.0198473 | Not queried | 1.52 | 4.21e-01 | 0.5404252807 |
|  | 7 | 152591099 | 152592038 | PDFSTATSENSEMBLUCSC | 940 | 10 | 39052 | island | 0.275002 | 0.5713940 | Not queried | 1.52 | 3.15e-01 | 0.4672865596 |
|  | 2 | 168572517 | 168572814 | PDFSTATSENSEMBLUCSC | 298 | 4 | 24834 | island | 0.667078 | 0.9296042 | Not queried | 1.52 | 2.00e-01 | 0.3826488233 |
|  | 14 | 73925368 | 73925622 | PDFSTATSENSEMBLUCSC | 255 | 4 | 12496 | tss | 0.021521 | 0.0188485 | Not queried | 1.52 | 3.43e-01 | 0.4672865596 |
|  | 13 | 113663206 | 113663538 | PDFSTATSENSEMBLUCSC | 333 | 4 | 11268 | gene;island | 0.887724 | 0.9583249 | Not queried | 1.52 | 4.86e-01 | 0.5640816327 |
|  | 20 | 22565881 | 22566289 | PDFSTATSENSEMBLUCSC | 409 | 6 | 25633 | tss;island | 0.033777 | 0.0477360 | Not queried | 1.52 | 8.18e-01 | 0.8469867211 |
|  | 4 | 174451954 | 174452159 | PDFSTATSENSEMBLUCSC | 206 | 4 | 30180 | gene;tss;island | 0.049725 | 0.1573947 | Not queried | 1.52 | 4.86e-01 | 0.5640816327 |
|  | 4 | 7069843 | 7070051 | PDFSTATSENSEMBLUCSC | 209 | 6 | 30290 | tss | 0.012311 | 0.0035354 | Not queried | 1.52 | 6.99e-01 | 0.7290267580 |
|  | 13 | 52419332 | 52419483 | PDFSTATSENSEMBLUCSC | 152 | 5 | 11454 | tss | 0.131747 | 0.0570990 | Not queried | 1.51 | 4.21e-01 | 0.5404252807 |
|  | 5 | 1950701 | 1951028 | PDFSTATSENSEMBLUCSC | 328 | 4 | 32648 | island | 0.707081 | 0.8732240 | Not queried | 1.51 | 2.00e-01 | 0.3826488233 |
|  | 5 | 171711430 | 171711562 | PDFSTATSENSEMBLUCSC | 133 | 4 | 32492 | tss;island | 0.002711 | 0.0018095 | Not queried | 1.51 | 4.86e-01 | 0.5640816327 |
|  | 14 | 57263993 | 57264287 | PDFSTATSENSEMBLUCSC | 295 | 4 | 12875 | island | 0.216194 | 0.0787654 | Not queried | 1.51 | 8.86e-01 | 0.8941250130 |
|  | 1 | 77747875 | 77748221 | PDFSTATSENSEMBLUCSC | 347 | 6 | 1705 | tss | 0.039941 | 0.0151330 | Not queried | 1.51 | 3.94e-01 | 0.5236526859 |
|  | 11 | 82444798 | 82445170 | PDFSTATSENSEMBLUCSC | 373 | 5 | 8698 | island | 0.127372 | 0.2931931 | Not queried | 1.51 | 8.41e-01 | 0.8635565145 |
|  | 13 | 95357638 | 95357832 | PDFSTATSENSEMBLUCSC | 195 | 4 | 11821 | island | 0.034597 | 0.0630413 | Not queried | 1.51 | 3.43e-01 | 0.4672865596 |
|  | 16 | 67926607 | 67927170 | PDFSTATSENSEMBLUCSC | 564 | 6 | 15316 | tss | 0.010292 | 0.0147605 | Not queried | 1.51 | 3.94e-01 | 0.5236526859 |
|  | 11 | 75140541 | 75140865 | PDFSTATSENSEMBLUCSC | 325 | 4 | 6836 | gene;island | 0.895614 | 0.9587811 | Not queried | 1.51 | 2.86e-02 | 0.1830867741 |
|  | 12 | 10183167 | 10183267 | PDFSTATSENSEMBLUCSC | 101 | 6 | 9559 | tss | 0.916443 | 0.9743593 | Not queried | 1.51 | 1.32e-01 | 0.3397986247 |
|  | 4 | 94749865 | 94750205 | PDFSTATSENSEMBLUCSC | 341 | 7 | 31186 | island | 0.066581 | 0.0209156 | Not queried | 1.51 | 2.62e-02 | 0.1830867741 |
|  | 22 | 30642820 | 30643063 | PDFSTATSENSEMBLUCSC | 244 | 4 | 27056 | tss | 0.031326 | 0.0074707 | Not queried | 1.51 | 2.00e-01 | 0.3826488233 |
|  | 11 | 788406 | 788714 | PDFSTATSENSEMBLUCSC | 309 | 4 | 6390 | gene;island | 0.871941 | 0.9532300 | Not queried | 1.51 | 3.43e-01 | 0.4672865596 |
|  | 12 | 96882885 | 96883863 | PDFSTATSENSEMBLUCSC | 979 | 9 | 10700 | island | 0.033325 | 0.3290218 | Not queried | 1.51 | 6.66e-01 | 0.7223604052 |
|  | 16 | 82660206 | 82660505 | PDFSTATSENSEMBLUCSC | 300 | 9 | 15385 | tss | 0.396685 | 0.0846439 | Not queried | 1.51 | 1.61e-01 | 0.3826488233 |
|  | 10 | 133947518 | 133947916 | PDFSTATSENSEMBLUCSC | 399 | 4 | 4850 | gene;island | 0.245135 | 0.6195486 | Not queried | 1.51 | 2.00e-01 | 0.3826488233 |
|  | 5 | 170736021 | 170736572 | PDFSTATSENSEMBLUCSC | 552 | 10 | 33379 | island | 0.192912 | 0.1272363 | Not queried | 1.51 | 2.47e-01 | 0.4242889032 |
|  | 1 | 150898437 | 150898761 | PDFSTATSENSEMBLUCSC | 325 | 8 | 1914 | tss;island | 0.007350 | 0.0108492 | Not queried | 1.51 | 4.42e-01 | 0.5640816327 |
|  | 11 | 130416857 | 130417168 | PDFSTATSENSEMBLUCSC | 312 | 5 | 8963 | island | 0.442166 | 0.6254598 | Not queried | 1.51 | 1.51e-01 | 0.3618000441 |
|  | 6 | 39760416 | 39760776 | PDFSTATSENSEMBLUCSC | 361 | 4 | 34959 | tss | 0.095558 | 0.0234253 | Not queried | 1.51 | 3.43e-01 | 0.4672865596 |
|  | 12 | 125223502 | 125223679 | PDFSTATSENSEMBLUCSC | 178 | 4 | 10976 | island | 0.839916 | 0.9159065 | Not queried | 1.51 | 2.00e-01 | 0.3826488233 |
|  | 4 | 1496822 | 1497223 | PDFSTATSENSEMBLUCSC | 402 | 5 | 30787 | island | 0.426106 | 0.6735733 | Not queried | 1.51 | 2.22e-01 | 0.3935758299 |
|  | 6 | 150378738 | 150379047 | PDFSTATSENSEMBLUCSC | 310 | 5 | 36566 | island | 0.449181 | 0.6711823 | Not queried | 1.51 | 5.56e-02 | 0.2315877671 |
|  | 8 | 91803657 | 91803805 | PDFSTATSENSEMBLUCSC | 149 | 5 | 39807 | tss;island | 0.226028 | 0.3880801 | Not queried | 1.51 | 3.10e-01 | 0.4672865596 |
|  | 19 | 51165207 | 51165845 | PDFSTATSENSEMBLUCSC | 639 | 6 | 20181 | gene;island | 0.635574 | 0.7738421 | Not queried | 1.51 | 3.94e-01 | 0.5236526859 |
|  | 9 | 138670609 | 138670828 | PDFSTATSENSEMBLUCSC | 220 | 4 | 40792 | gene;island | 0.436660 | 0.5277164 | Not queried | 1.51 | 6.86e-01 | 0.7223604052 |
|  | 7 | 64734093 | 64734749 | PDFSTATSENSEMBLUCSC | 657 | 7 | 38527 | island | 0.043718 | 0.0092132 | Not queried | 1.51 | 9.73e-02 | 0.3123457953 |
|  | 15 | 55881075 | 55881246 | PDFSTATSENSEMBLUCSC | 172 | 6 | 13684 | tss | 0.306524 | 0.0678728 | Not queried | 1.51 | 3.94e-01 | 0.5236526859 |
|  | 1 | 27817084 | 27817404 | PDFSTATSENSEMBLUCSC | 321 | 4 | 1414 | tss;island | 0.043834 | 0.0201731 | Not queried | 1.51 | 4.86e-01 | 0.5640816327 |
|  | 11 | 120894635 | 120894841 | PDFSTATSENSEMBLUCSC | 207 | 5 | 8897 | island | 0.016789 | 0.0051368 | Not queried | 1.51 | 4.21e-01 | 0.5404252807 |
|  | 10 | 123355268 | 123355748 | PDFSTATSENSEMBLUCSC | 481 | 4 | 4794 | gene;island | 0.429493 | 0.6189388 | Not queried | 1.51 | 5.71e-02 | 0.2315877671 |
|  | 15 | 90319420 | 90319584 | PDFSTATSENSEMBLUCSC | 165 | 4 | 13892 | tss | 0.125766 | 0.2931458 | Not queried | 1.51 | 4.86e-01 | 0.5640816327 |
|  | 4 | 4868723 | 4869172 | PDFSTATSENSEMBLUCSC | 450 | 6 | 30874 | island | 0.041555 | 0.0201637 | Not queried | 1.51 | 9.31e-02 | 0.3104222821 |
|  | 11 | 19367501 | 19368277 | PDFSTATSENSEMBLUCSC | 777 | 8 | 8120 | island | 0.006483 | 0.0167882 | Not queried | 1.51 | 4.99e-02 | 0.2315877671 |
|  | 4 | 183063397 | 183063637 | PDFSTATSENSEMBLUCSC | 241 | 4 | 30190 | gene;island | 0.038671 | 0.0066590 | Not queried | 1.51 | 3.43e-01 | 0.4672865596 |
|  | 17 | 74137336 | 74137522 | PDFSTATSENSEMBLUCSC | 187 | 6 | 18774 | island | 0.003879 | 0.0055274 | Not queried | 1.51 | 5.89e-01 | 0.6570484697 |
|  | 1 | 18806517 | 18806982 | PDFSTATSENSEMBLUCSC | 466 | 4 | 1294 | tss;island | 0.302028 | 0.5349676 | Not queried | 1.51 | 6.86e-01 | 0.7223604052 |
|  | 4 | 54969963 | 54970254 | PDFSTATSENSEMBLUCSC | 292 | 4 | 31052 | island | 0.254845 | 0.1102809 | Not queried | 1.51 | 2.00e-01 | 0.3826488233 |
|  | 19 | 18451112 | 18451326 | PDFSTATSENSEMBLUCSC | 215 | 8 | 20617 | tss | 0.012616 | 0.0372867 | Not queried | 1.51 | 3.79e-02 | 0.2121545571 |
|  | 16 | 84256036 | 84256337 | PDFSTATSENSEMBLUCSC | 302 | 4 | 14866 | gene;island | 0.336196 | 0.5437979 | Not queried | 1.51 | 3.43e-01 | 0.4672865596 |
|  | 18 | 596703 | 596943 | PDFSTATSENSEMBLUCSC | 241 | 5 | 19152 | tss | 0.012391 | 0.0251542 | Not queried | 1.50 | 5.48e-01 | 0.6179718485 |
|  | 2 | 220041926 | 220042205 | PDFSTATSENSEMBLUCSC | 280 | 4 | 23892 | tss;island | 0.001340 | 0.0031188 | Not queried | 1.50 | 6.86e-01 | 0.7223604052 |
|  | 14 | 105767366 | 105767639 | PDFSTATSENSEMBLUCSC | 274 | 7 | 12670 | tss;island | 0.003269 | 0.0061061 | Not queried | 1.50 | 7.10e-01 | 0.7390730975 |
|  | 14 | 35099471 | 35099711 | PDFSTATSENSEMBLUCSC | 241 | 5 | 12347 | tss | 0.119855 | 0.0293947 | Not queried | 1.50 | 5.48e-01 | 0.6179718485 |
|  | 7 | 1272255 | 1272559 | PDFSTATSENSEMBLUCSC | 305 | 8 | 37398 | tss | 0.110116 | 0.1921847 | Not queried | 1.50 | 5.74e-01 | 0.6460388787 |
|  | 6 | 24877462 | 24877729 | PDFSTATSENSEMBLUCSC | 268 | 4 | 33594 | gene | 0.977131 | 0.9280579 | Not queried | 1.50 | 2.86e-02 | 0.1830867741 |
|  | 19 | 13365923 | 13366101 | PDFSTATSENSEMBLUCSC | 179 | 4 | 19880 | gene;island | 0.935064 | 0.9600017 | Not queried | 1.50 | 4.86e-01 | 0.5640816327 |
|  | 4 | 66536186 | 66536772 | PDFSTATSENSEMBLUCSC | 587 | 7 | 30417 | tss;island | 0.007378 | 0.0256086 | Not queried | 1.50 | 7.10e-01 | 0.7390730975 |
|  | 3 | 43147571 | 43148002 | PDFSTATSENSEMBLUCSC | 432 | 8 | 28251 | tss | 0.034224 | 0.0115684 | Not queried | 1.50 | 4.99e-02 | 0.2315877671 |
|  | 7 | 129414567 | 129414894 | PDFSTATSENSEMBLUCSC | 328 | 12 | 38911 | island | 0.539132 | 0.8168730 | Not queried | 1.50 | 1.00e-02 | 0.1806165940 |
|  | 19 | 4153364 | 4153603 | PDFSTATSENSEMBLUCSC | 240 | 4 | 20360 | tss | 0.068926 | 0.3156329 | Not queried | 1.50 | 2.00e-01 | 0.3826488233 |
|  | 10 | 20105179 | 20105641 | PDFSTATSENSEMBLUCSC | 463 | 5 | 5588 | island | 0.032925 | 0.3882682 | Not queried | 1.50 | 6.90e-01 | 0.7223604052 |
|  | 1 | 43424882 | 43425412 | PDFSTATSENSEMBLUCSC | 531 | 6 | 1555 | tss | 0.008334 | 0.0047621 | Not queried | 1.50 | 3.94e-01 | 0.5236526859 |
|  | 3 | 111804830 | 111805077 | PDFSTATSENSEMBLUCSC | 248 | 6 | 28499 | tss | 0.078122 | 0.0212424 | Not queried | 1.50 | 4.11e-02 | 0.2180389943 |
|  | 11 | 67169500 | 67169560 | PDFSTATSENSEMBLUCSC | 61 | 4 | 7490 | tss | 0.019396 | 0.0127071 | Not queried | 1.50 | 6.86e-01 | 0.7223604052 |
|  | 1 | 98511523 | 98511908 | PDFSTATSENSEMBLUCSC | 386 | 5 | 3514 | island | 0.028644 | 0.0774009 | Not queried | 1.50 | 8.41e-01 | 0.8635565145 |
|  | 6 | 31782675 | 31782761 | PDFSTATSENSEMBLUCSC | 87 | 4 | 33967 | gene | 0.003158 | 0.0161408 | Not queried | 1.50 | 8.86e-01 | 0.8941250130 |
|  | 1 | 154965419 | 154965572 | PDFSTATSENSEMBLUCSC | 154 | 4 | 787 | gene;tss | 0.718726 | 0.9572602 | Not queried | 1.50 | 3.43e-01 | 0.4672865596 |
|  | 1 | 228464049 | 228464394 | PDFSTATSENSEMBLUCSC | 346 | 4 | 1052 | gene;island | 0.479038 | 0.7050606 | Not queried | 1.50 | 6.86e-01 | 0.7223604052 |
|  | 13 | 111281037 | 111281479 | PDFSTATSENSEMBLUCSC | 443 | 6 | 11234 | gene;island | 0.879963 | 0.9498020 | Not queried | 1.50 | 1.52e-02 | 0.1830867741 |
|  | 7 | 158319029 | 158319204 | PDFSTATSENSEMBLUCSC | 176 | 4 | 37377 | gene;island | 0.399448 | 0.7051042 | Not queried | 1.50 | 1.14e-01 | 0.3123457953 |
|  | 1 | 228652338 | 228652581 | PDFSTATSENSEMBLUCSC | 244 | 4 | 4302 | island | 0.011689 | 0.0359338 | Not queried | 1.50 | 2.00e-01 | 0.3826488233 |
|  | 16 | 31548640 | 31548908 | PDFSTATSENSEMBLUCSC | 269 | 4 | 15881 | island | 0.544134 | 0.4855602 | Not queried | 1.50 | 8.86e-01 | 0.8941250130 |
|  | 19 | 49944488 | 49944964 | PDFSTATSENSEMBLUCSC | 477 | 8 | 22266 | island | 0.075683 | 0.0192677 | Not queried | 1.50 | 2.81e-02 | 0.1830867741 |
|  | 6 | 161575229 | 161575667 | PDFSTATSENSEMBLUCSC | 439 | 7 | 34537 | gene | 0.848917 | 0.9028214 | Not queried | 1.50 | 2.09e-01 | 0.3935758299 |
|  | 7 | 50628718 | 50629067 | PDFSTATSENSEMBLUCSC | 350 | 4 | 37012 | gene | 0.148880 | 0.3298638 | Not queried | 1.50 | 8.86e-01 | 0.8941250130 |
|  | 6 | 100911687 | 100911746 | PDFSTATSENSEMBLUCSC | 60 | 6 | 35139 | tss | 0.140058 | 0.3819300 | Not queried | 1.50 | 3.10e-01 | 0.4672865596 |
|  | 1 | 47082037 | 47082211 | PDFSTATSENSEMBLUCSC | 175 | 4 | 1602 | tss | 0.006960 | 0.0074439 | Not queried | 1.50 | 6.86e-01 | 0.7223604052 |
|  | 14 | 65347138 | 65347518 | PDFSTATSENSEMBLUCSC | 381 | 4 | 12943 | island | 0.042436 | 0.0236433 | Not queried | 1.50 | 3.43e-01 | 0.4672865596 |
|  | 19 | 47925344 | 47925618 | PDFSTATSENSEMBLUCSC | 275 | 5 | 22194 | island | 0.013596 | 0.0086676 | Not queried | 1.50 | 3.10e-01 | 0.4672865596 |
|  | 11 | 66511804 | 66512052 | PDFSTATSENSEMBLUCSC | 249 | 4 | 7480 | tss;island | 0.005839 | 0.0076777 | Not queried | 1.50 | 8.86e-01 | 0.8941250130 |
|  | 7 | 2499915 | 2500200 | PDFSTATSENSEMBLUCSC | 286 | 6 | 38158 | island | 0.423974 | 0.6433618 | Not queried | 1.50 | 2.40e-01 | 0.4133265075 |
|  | 12 | 48722990 | 48723360 | PDFSTATSENSEMBLUCSC | 371 | 4 | 9112 | gene;island | 0.670567 | 0.8186257 | Not queried | 1.50 | 6.86e-01 | 0.7223604052 |
|  | 21 | 43528734 | 43528949 | PDFSTATSENSEMBLUCSC | 216 | 4 | 26435 | gene;tss;island | 0.489292 | 0.1918909 | Not queried | 1.50 | 6.86e-01 | 0.7223604052 |
|  | 7 | 157567895 | 157568392 | PDFSTATSENSEMBLUCSC | 498 | 6 | 37344 | gene;island | 0.392202 | 0.6570615 | Not queried | 1.50 | 2.16e-03 | 0.0934794372 |
|  | 12 | 52695197 | 52695623 | PDFSTATSENSEMBLUCSC | 427 | 7 | 9726 | tss | 0.426838 | 0.7443484 | Not queried | 1.50 | 7.10e-01 | 0.7390730975 |
|  | 19 | 58458572 | 58459358 | PDFSTATSENSEMBLUCSC | 787 | 12 | 22488 | island | 0.028238 | 0.0136535 | Not queried | 1.50 | 1.73e-02 | 0.1830867741 |
|  | 14 | 37135384 | 37135671 | PDFSTATSENSEMBLUCSC | 288 | 4 | 12068 | gene;island | 0.160627 | 0.0659419 | Not queried | 1.50 | 2.00e-01 | 0.3826488233 |
|  | 16 | 3243304 | 3243552 | PDFSTATSENSEMBLUCSC | 249 | 4 | 15641 | island | 0.959182 | 0.8087573 | Not queried | 1.50 | 2.00e-01 | 0.3826488233 |
|  | 16 | 6068831 | 6069047 | PDFSTATSENSEMBLUCSC | 217 | 4 | 15080 | tss | 0.030967 | 0.0594160 | Not queried | 1.50 | 4.86e-01 | 0.5640816327 |
|  | 2 | 468737 | 469214 | PDFSTATSENSEMBLUCSC | 478 | 5 | 24003 | island | 0.456086 | 0.7848009 | Not queried | 1.50 | 5.48e-01 | 0.6179718485 |
|  | 11 | 64885222 | 64885558 | PDFSTATSENSEMBLUCSC | 337 | 8 | 7415 | tss | 0.006582 | 0.0028725 | Not queried | 1.49 | 1.30e-01 | 0.3397986247 |
|  | 15 | 76004985 | 76005171 | PDFSTATSENSEMBLUCSC | 187 | 4 | 13432 | gene | 0.218176 | 0.3204939 | Not queried | 1.49 | 3.43e-01 | 0.4672865596 |
|  | 1 | 26394201 | 26394593 | PDFSTATSENSEMBLUCSC | 393 | 5 | 1382 | tss | 0.274436 | 0.5619599 | Not queried | 1.49 | 8.41e-01 | 0.8635565145 |
|  | 3 | 50376000 | 50376475 | PDFSTATSENSEMBLUCSC | 476 | 6 | 27806 | gene;tss;island | 0.549080 | 0.7158266 | Not queried | 1.49 | 4.85e-01 | 0.5640816327 |
|  | 19 | 18451410 | 18451650 | PDFSTATSENSEMBLUCSC | 241 | 4 | 19933 | gene | 0.009268 | 0.0056198 | Not queried | 1.49 | 4.86e-01 | 0.5640816327 |
|  | 13 | 20710941 | 20711179 | PDFSTATSENSEMBLUCSC | 239 | 4 | 11566 | island | 0.051780 | 0.0110849 | Not queried | 1.49 | 3.43e-01 | 0.4672865596 |
|  | 6 | 152125861 | 152126441 | PDFSTATSENSEMBLUCSC | 581 | 8 | 34511 | gene;tss;island | 0.615735 | 0.7408235 | Not queried | 1.49 | 3.82e-01 | 0.5168893301 |
|  | 17 | 8027830 | 8027961 | PDFSTATSENSEMBLUCSC | 132 | 4 | 17108 | tss;island | 0.023412 | 0.0149956 | Not queried | 1.49 | 4.86e-01 | 0.5640816327 |
|  | 2 | 44395749 | 44395983 | PDFSTATSENSEMBLUCSC | 235 | 6 | 23331 | tss | 0.018829 | 0.0091933 | Not queried | 1.49 | 3.10e-01 | 0.4672865596 |
|  | 1 | 166890293 | 166890436 | PDFSTATSENSEMBLUCSC | 144 | 4 | 871 | gene;island | 0.272893 | 0.1410157 | Not queried | 1.49 | 2.00e-01 | 0.3826488233 |
|  | 3 | 185655795 | 185656289 | PDFSTATSENSEMBLUCSC | 495 | 6 | 29744 | island | 0.013540 | 0.0231935 | Not queried | 1.49 | 4.85e-01 | 0.5640816327 |
|  | 4 | 54232368 | 54232638 | PDFSTATSENSEMBLUCSC | 271 | 4 | 30394 | tss | 0.003184 | 0.0084904 | Not queried | 1.49 | 4.86e-01 | 0.5640816327 |
|  | 6 | 126070013 | 126070499 | PDFSTATSENSEMBLUCSC | 487 | 7 | 35211 | tss;island | 0.009430 | 0.0037202 | Not queried | 1.49 | 2.09e-01 | 0.3935758299 |
|  | 1 | 179711859 | 179712361 | PDFSTATSENSEMBLUCSC | 503 | 10 | 4001 | island | 0.158089 | 0.0439092 | Not queried | 1.49 | 2.32e-02 | 0.1830867741 |
|  | 16 | 1088364 | 1088734 | PDFSTATSENSEMBLUCSC | 371 | 4 | 15510 | island | 0.488889 | 0.8143758 | Not queried | 1.49 | 3.43e-01 | 0.4672865596 |
|  | 5 | 178322737 | 178322856 | PDFSTATSENSEMBLUCSC | 120 | 5 | 32544 | tss | 0.060236 | 0.0824277 | Not queried | 1.49 | 2.22e-01 | 0.3935758299 |
|  | 1 | 6484616 | 6484980 | PDFSTATSENSEMBLUCSC | 365 | 4 | 2605 | island | 0.323861 | 0.0673099 | Not queried | 1.49 | 4.86e-01 | 0.5640816327 |
|  | 10 | 75173098 | 75173760 | PDFSTATSENSEMBLUCSC | 663 | 6 | 4634 | gene | 0.008041 | 0.0038971 | Not queried | 1.49 | 2.40e-01 | 0.4133265075 |
|  | 6 | 13924355 | 13924708 | PDFSTATSENSEMBLUCSC | 354 | 6 | 34647 | tss;island | 0.372135 | 0.6290747 | Not queried | 1.49 | 2.40e-01 | 0.4133265075 |
|  | 19 | 3687751 | 3688176 | PDFSTATSENSEMBLUCSC | 426 | 5 | 19721 | gene;island | 0.011822 | 0.0145177 | Not queried | 1.49 | 2.22e-01 | 0.3935758299 |
|  | 7 | 128784416 | 128784712 | PDFSTATSENSEMBLUCSC | 297 | 7 | 38905 | island | 0.009150 | 0.0244624 | Not queried | 1.49 | 9.73e-02 | 0.3123457953 |
|  | 6 | 27258460 | 27258767 | PDFSTATSENSEMBLUCSC | 308 | 6 | 35552 | island | 0.580771 | 0.2717177 | Not queried | 1.49 | 3.10e-01 | 0.4672865596 |
|  | 6 | 30130109 | 30130458 | PDFSTATSENSEMBLUCSC | 350 | 7 | 34764 | tss | 0.728575 | 0.8822096 | Not queried | 1.49 | 3.83e-01 | 0.5168893301 |
|  | 17 | 56064295 | 56064928 | PDFSTATSENSEMBLUCSC | 634 | 7 | 16706 | gene;island | 0.003290 | 0.0107299 | Not queried | 1.49 | 2.09e-01 | 0.3935758299 |
|  | 17 | 48785255 | 48785602 | PDFSTATSENSEMBLUCSC | 348 | 6 | 18559 | island | 0.016746 | 0.0069435 | Not queried | 1.49 | 5.89e-01 | 0.6570484697 |
|  | 7 | 121651766 | 121652024 | PDFSTATSENSEMBLUCSC | 259 | 4 | 37204 | gene | 0.006618 | 0.0201150 | Not queried | 1.49 | 4.86e-01 | 0.5640816327 |
|  | 1 | 92951720 | 92953037 | PDFSTATSENSEMBLUCSC | 1318 | 20 | 1752 | tss | 0.011000 | 0.0058006 | Not queried | 1.49 | 4.60e-02 | 0.2315877671 |
|  | 10 | 7661611 | 7661796 | PDFSTATSENSEMBLUCSC | 186 | 5 | 4484 | gene | 0.627117 | 0.8726270 | Not queried | 1.49 | 3.10e-01 | 0.4672865596 |
|  | 11 | 17373103 | 17373302 | PDFSTATSENSEMBLUCSC | 200 | 6 | 7174 | tss;island | 0.048818 | 0.0468460 | Not queried | 1.49 | 5.89e-01 | 0.6570484697 |
|  | 16 | 89440878 | 89441217 | PDFSTATSENSEMBLUCSC | 340 | 4 | 14942 | gene;island | 0.966104 | 0.8455548 | Not queried | 1.49 | 4.86e-01 | 0.5640816327 |
|  | 21 | 40817540 | 40817657 | PDFSTATSENSEMBLUCSC | 118 | 4 | 26551 | tss | 0.001813 | 0.0046743 | Not queried | 1.48 | 6.86e-01 | 0.7223604052 |
|  | 13 | 114312504 | 114312763 | PDFSTATSENSEMBLUCSC | 260 | 7 | 11554 | tss | 0.471664 | 0.7228183 | Not queried | 1.48 | 3.83e-01 | 0.5168893301 |
|  | 15 | 93615439 | 93615718 | PDFSTATSENSEMBLUCSC | 280 | 4 | 13489 | gene;island | 0.001099 | 0.0025650 | Not queried | 1.48 | 1.14e-01 | 0.3123457953 |
|  | 2 | 201171255 | 201171347 | PDFSTATSENSEMBLUCSC | 93 | 5 | 23808 | tss | 0.015146 | 0.0008757 | Not queried | 1.48 | 3.10e-01 | 0.4672865596 |
|  | 14 | 27066404 | 27066771 | PDFSTATSENSEMBLUCSC | 368 | 4 | 12052 | gene;island | 0.048525 | 0.0174840 | Not queried | 1.48 | 1.14e-01 | 0.3123457953 |
|  | 22 | 44258572 | 44258712 | PDFSTATSENSEMBLUCSC | 141 | 5 | 27205 | tss;island | 0.164561 | 0.0803602 | Not queried | 1.48 | 9.52e-02 | 0.3104222821 |
|  | 17 | 30244184 | 30244630 | PDFSTATSENSEMBLUCSC | 447 | 4 | 18191 | island | 0.631405 | 0.4257294 | Not queried | 1.48 | 6.86e-01 | 0.7223604052 |
|  | 20 | 25129126 | 25129400 | PDFSTATSENSEMBLUCSC | 275 | 4 | 25378 | gene | 0.039669 | 0.1209305 | Not queried | 1.48 | 1.14e-01 | 0.3123457953 |
|  | 3 | 26664115 | 26664404 | PDFSTATSENSEMBLUCSC | 290 | 5 | 28945 | island | 0.034622 | 0.0506999 | Not queried | 1.48 | 3.10e-01 | 0.4672865596 |
|  | 6 | 30124214 | 30124299 | PDFSTATSENSEMBLUCSC | 86 | 4 | 33731 | gene | 0.539759 | 0.7524369 | Not queried | 1.48 | 4.86e-01 | 0.5640816327 |
|  | 21 | 34442160 | 34442674 | PDFSTATSENSEMBLUCSC | 515 | 10 | 26643 | island | 0.024228 | 0.0129998 | Not queried | 1.48 | 3.15e-01 | 0.4672865596 |
|  | 18 | 10588980 | 10589360 | PDFSTATSENSEMBLUCSC | 381 | 5 | 19392 | island | 0.900586 | 0.9509761 | Not queried | 1.48 | 3.10e-01 | 0.4672865596 |
|  | 6 | 29573014 | 29573377 | PDFSTATSENSEMBLUCSC | 364 | 5 | 33666 | gene | 0.448437 | 0.7813951 | Not queried | 1.48 | 4.21e-01 | 0.5404252807 |
|  | 2 | 176994142 | 176994448 | PDFSTATSENSEMBLUCSC | 307 | 5 | 23739 | tss | 0.059107 | 0.2091567 | Not queried | 1.48 | 2.22e-01 | 0.3935758299 |
|  | 3 | 167966505 | 167967220 | PDFSTATSENSEMBLUCSC | 716 | 6 | 28705 | tss;island | 0.019935 | 0.0129735 | Not queried | 1.48 | 1.80e-01 | 0.3826488233 |
|  | 7 | 157444235 | 157444501 | PDFSTATSENSEMBLUCSC | 267 | 5 | 37334 | gene;island | 0.464058 | 0.7595352 | Not queried | 1.48 | 3.10e-01 | 0.4672865596 |
|  | 17 | 16593426 | 16593613 | PDFSTATSENSEMBLUCSC | 188 | 6 | 17161 | tss;island | 0.496981 | 0.2020077 | Not queried | 1.48 | 1.32e-01 | 0.3397986247 |
|  | 5 | 154091976 | 154092426 | PDFSTATSENSEMBLUCSC | 451 | 6 | 32450 | tss | 0.829944 | 0.9232076 | Not queried | 1.48 | 3.10e-01 | 0.4672865596 |
|  | 12 | 121163597 | 121164371 | PDFSTATSENSEMBLUCSC | 775 | 8 | 9358 | gene | 0.079598 | 0.1171663 | Not queried | 1.48 | 5.05e-01 | 0.5852592101 |
|  | 7 | 36700744 | 36701016 | PDFSTATSENSEMBLUCSC | 273 | 4 | 36966 | gene | 0.254250 | 0.4621867 | Not queried | 1.48 | 4.86e-01 | 0.5640816327 |
|  | 11 | 3253659 | 3254504 | PDFSTATSENSEMBLUCSC | 846 | 14 | 7094 | tss | 0.655817 | 0.7885070 | Not queried | 1.48 | 1.04e-01 | 0.3123457953 |
|  | 17 | 45772333 | 45772523 | PDFSTATSENSEMBLUCSC | 191 | 5 | 17443 | tss | 0.006531 | 0.0017316 | Not queried | 1.48 | 5.48e-01 | 0.6179718485 |
|  | 6 | 3445256 | 3445330 | PDFSTATSENSEMBLUCSC | 75 | 4 | 33537 | gene;tss | 0.267669 | 0.4253230 | Not queried | 1.48 | 4.86e-01 | 0.5640816327 |
|  | 11 | 1074682 | 1074873 | PDFSTATSENSEMBLUCSC | 192 | 6 | 7038 | tss | 0.506712 | 0.6923000 | Not queried | 1.48 | 2.40e-01 | 0.4133265075 |
|  | 14 | 101123263 | 101123867 | PDFSTATSENSEMBLUCSC | 605 | 7 | 13140 | island | 0.526809 | 0.8013707 | Not queried | 1.48 | 2.09e-01 | 0.3935758299 |
|  | 14 | 101123263 | 101123599 | PDFSTATSENSEMBLUCSC | 337 | 5 | 12606 | tss | 0.701154 | 0.8013707 | Not queried | 1.48 | 4.21e-01 | 0.5404252807 |
|  | 2 | 85131951 | 85132663 | PDFSTATSENSEMBLUCSC | 713 | 7 | 23478 | tss | 0.004128 | 0.0085602 | Not queried | 1.48 | 5.35e-01 | 0.6176455124 |
|  | 10 | 95030 | 95398 | PDFSTATSENSEMBLUCSC | 369 | 7 | 4429 | gene;island | 0.250066 | 0.5534574 | Not queried | 1.48 | 2.59e-01 | 0.4381244265 |
|  | 15 | 78631512 | 78631652 | PDFSTATSENSEMBLUCSC | 141 | 4 | 13833 | tss;island | 0.495456 | 0.6371056 | Not queried | 1.48 | 4.86e-01 | 0.5640816327 |
|  | 16 | 2479181 | 2479406 | PDFSTATSENSEMBLUCSC | 226 | 4 | 15603 | island | 0.002507 | 0.0078826 | Not queried | 1.48 | 4.86e-01 | 0.5640816327 |
|  | 17 | 76564972 | 76565232 | PDFSTATSENSEMBLUCSC | 261 | 4 | 16824 | gene | 0.111065 | 0.2705387 | Not queried | 1.48 | 4.86e-01 | 0.5640816327 |
|  | 19 | 54676948 | 54677232 | PDFSTATSENSEMBLUCSC | 285 | 6 | 21055 | tss | 0.227455 | 0.0951465 | Not queried | 1.48 | 6.99e-01 | 0.7290267580 |
|  | 11 | 45230723 | 45231326 | PDFSTATSENSEMBLUCSC | 604 | 6 | 6592 | gene;island | 0.507884 | 0.6536042 | Not queried | 1.48 | 3.94e-01 | 0.5236526859 |
|  | 6 | 29274521 | 29275337 | PDFSTATSENSEMBLUCSC | 817 | 12 | 33651 | gene | 0.291221 | 0.3615302 | Not queried | 1.48 | 3.19e-01 | 0.4672865596 |
|  | 6 | 32119616 | 32119691 | PDFSTATSENSEMBLUCSC | 76 | 4 | 34059 | gene | 0.100237 | 0.1603067 | Not queried | 1.48 | 4.86e-01 | 0.5640816327 |
|  | 8 | 109095918 | 109096151 | PDFSTATSENSEMBLUCSC | 234 | 7 | 39866 | tss | 0.439271 | 0.1656072 | Not queried | 1.48 | 7.10e-01 | 0.7390730975 |
|  | 6 | 43655599 | 43655795 | PDFSTATSENSEMBLUCSC | 197 | 6 | 35005 | tss | 0.021555 | 0.0041579 | Not queried | 1.48 | 2.40e-01 | 0.4133265075 |
|  | 2 | 162930666 | 162931003 | PDFSTATSENSEMBLUCSC | 338 | 4 | 22911 | gene | 0.014854 | 0.0094031 | Not queried | 1.48 | 6.86e-01 | 0.7223604052 |
|  | 14 | 56232985 | 56233178 | PDFSTATSENSEMBLUCSC | 194 | 4 | 12090 | gene | 0.563237 | 0.9143382 | Not queried | 1.47 | 1.14e-01 | 0.3123457953 |
|  | 21 | 46359575 | 46359716 | PDFSTATSENSEMBLUCSC | 142 | 4 | 26461 | gene;tss;island | 0.001674 | 0.0044663 | Not queried | 1.47 | 3.43e-01 | 0.4672865596 |
|  | 17 | 73874443 | 73874790 | PDFSTATSENSEMBLUCSC | 348 | 7 | 18765 | island | 0.060081 | 0.0129582 | Not queried | 1.47 | 1.28e-01 | 0.3397986247 |
|  | 17 | 7909776 | 7910040 | PDFSTATSENSEMBLUCSC | 265 | 4 | 16423 | gene;island | 0.717170 | 0.9203362 | Not queried | 1.47 | 4.86e-01 | 0.5640816327 |
|  | 1 | 180992098 | 180992117 | PDFSTATSENSEMBLUCSC | 20 | 4 | 2127 | tss | 0.001121 | 0.0006708 | Not queried | 1.47 | 2.00e-01 | 0.3826488233 |
|  | 17 | 8661887 | 8662011 | PDFSTATSENSEMBLUCSC | 125 | 5 | 17121 | tss | 0.477414 | 0.6294408 | Not queried | 1.47 | 1.51e-01 | 0.3618000441 |
|  | 16 | 731772 | 732216 | PDFSTATSENSEMBLUCSC | 445 | 4 | 14537 | gene;island | 0.736006 | 0.9092615 | Not queried | 1.47 | 4.86e-01 | 0.5640816327 |
|  | 1 | 46152302 | 46152494 | PDFSTATSENSEMBLUCSC | 193 | 5 | 1589 | tss | 0.003761 | 0.0032501 | Not queried | 1.47 | 1.51e-01 | 0.3618000441 |
|  | 13 | 113120945 | 113121280 | PDFSTATSENSEMBLUCSC | 336 | 4 | 11923 | island | 0.838592 | 0.9336456 | Not queried | 1.47 | 4.86e-01 | 0.5640816327 |
|  | 1 | 1148973 | 1149211 | PDFSTATSENSEMBLUCSC | 239 | 4 | 21 | gene;island | 0.029832 | 0.1012860 | Not queried | 1.47 | 2.00e-01 | 0.3826488233 |
|  | 19 | 5822318 | 5822630 | PDFSTATSENSEMBLUCSC | 313 | 4 | 20383 | tss;island | 0.261361 | 0.6348993 | Not queried | 1.47 | 3.43e-01 | 0.4672865596 |
|  | 4 | 183721565 | 183721778 | PDFSTATSENSEMBLUCSC | 214 | 4 | 30193 | gene;island | 0.651527 | 0.8944040 | Not queried | 1.47 | 4.86e-01 | 0.5640816327 |
|  | 8 | 143459774 | 143460209 | PDFSTATSENSEMBLUCSC | 436 | 6 | 39462 | gene | 0.827984 | 0.9266823 | Not queried | 1.47 | 1.80e-01 | 0.3826488233 |
|  | 3 | 145878963 | 145879277 | PDFSTATSENSEMBLUCSC | 315 | 4 | 27982 | gene | 0.048203 | 0.0271433 | Not queried | 1.47 | 1.14e-01 | 0.3123457953 |
|  | 6 | 33158503 | 33158597 | PDFSTATSENSEMBLUCSC | 95 | 4 | 34214 | gene;island | 0.573614 | 0.7555737 | Not queried | 1.47 | 6.86e-01 | 0.7223604052 |
|  | 19 | 17377962 | 17378213 | PDFSTATSENSEMBLUCSC | 252 | 4 | 20583 | tss | 0.019630 | 0.0138786 | Not queried | 1.47 | 8.86e-01 | 0.8941250130 |
|  | 16 | 23765810 | 23766116 | PDFSTATSENSEMBLUCSC | 307 | 5 | 15769 | island | 0.270799 | 0.1668973 | Not queried | 1.47 | 2.22e-01 | 0.3935758299 |
|  | 13 | 53425635 | 53426032 | PDFSTATSENSEMBLUCSC | 398 | 5 | 11764 | island | 0.003455 | 0.0116660 | Not queried | 1.47 | 6.90e-01 | 0.7223604052 |
|  | 10 | 105344723 | 105344807 | PDFSTATSENSEMBLUCSC | 85 | 4 | 4744 | gene;island | 0.437152 | 0.2396080 | Not queried | 1.47 | 1.14e-01 | 0.3123457953 |
|  | 6 | 52009019 | 52009126 | PDFSTATSENSEMBLUCSC | 108 | 4 | 35029 | tss | 0.406063 | 0.7265184 | Not queried | 1.47 | 3.43e-01 | 0.4672865596 |
|  | 15 | 43531947 | 43532243 | PDFSTATSENSEMBLUCSC | 297 | 4 | 13314 | gene | 0.172636 | 0.5344091 | Not queried | 1.47 | 3.43e-01 | 0.4672865596 |
|  | 10 | 134901199 | 134901689 | PDFSTATSENSEMBLUCSC | 491 | 9 | 6332 | island | 0.076554 | 0.0236159 | Not queried | 1.47 | 7.70e-02 | 0.2825346647 |
|  | 15 | 99789622 | 99789855 | PDFSTATSENSEMBLUCSC | 234 | 5 | 14503 | island | 0.006067 | 0.0329515 | Not queried | 1.47 | 2.22e-01 | 0.3935758299 |
|  | 14 | 60559007 | 60559200 | PDFSTATSENSEMBLUCSC | 194 | 4 | 12100 | gene;island | 0.004621 | 0.0121580 | Not queried | 1.47 | 1.14e-01 | 0.3123457953 |
|  | 12 | 10876053 | 10876242 | PDFSTATSENSEMBLUCSC | 190 | 7 | 9563 | tss | 0.001761 | 0.0015209 | Not queried | 1.47 | 2.09e-01 | 0.3935758299 |
|  | 5 | 95159365 | 95159639 | PDFSTATSENSEMBLUCSC | 275 | 4 | 32187 | tss | 0.070576 | 0.4502314 | Not queried | 1.47 | 4.86e-01 | 0.5640816327 |
|  | 7 | 27282233 | 27282444 | PDFSTATSENSEMBLUCSC | 212 | 6 | 36943 | gene;island | 0.045666 | 0.0207873 | Not queried | 1.47 | 1.52e-02 | 0.1830867741 |
|  | 5 | 7869257 | 7869428 | PDFSTATSENSEMBLUCSC | 172 | 5 | 31558 | gene | 0.002569 | 0.0069708 | Not queried | 1.47 | 4.21e-01 | 0.5404252807 |
|  | 14 | 105444384 | 105445253 | PDFSTATSENSEMBLUCSC | 870 | 10 | 13224 | island | 0.015487 | 0.0646524 | Not queried | 1.47 | 1.90e-01 | 0.3826488233 |
|  | 17 | 46894191 | 46894465 | PDFSTATSENSEMBLUCSC | 275 | 4 | 16668 | gene | 0.024477 | 0.0113202 | Not queried | 1.47 | 4.86e-01 | 0.5640816327 |
|  | 5 | 153857789 | 153858102 | PDFSTATSENSEMBLUCSC | 314 | 6 | 33329 | island | 0.066140 | 0.0408608 | Not queried | 1.47 | 1.00e+00 | 1.0000000000 |
|  | 12 | 49582874 | 49583112 | PDFSTATSENSEMBLUCSC | 239 | 7 | 9684 | tss | 0.011851 | 0.0061522 | Not queried | 1.47 | 2.59e-01 | 0.4381244265 |
|  | 5 | 133451237 | 133451474 | PDFSTATSENSEMBLUCSC | 238 | 7 | 32287 | tss | 0.012380 | 0.0225059 | Not queried | 1.47 | 3.18e-01 | 0.4672865596 |
|  | 22 | 36806414 | 36806655 | PDFSTATSENSEMBLUCSC | 242 | 4 | 27452 | island | 0.546771 | 0.3053379 | Not queried | 1.46 | 3.43e-01 | 0.4672865596 |
|  | 2 | 906044 | 906427 | PDFSTATSENSEMBLUCSC | 384 | 4 | 23156 | tss | 0.685553 | 0.8553213 | Not queried | 1.46 | 3.43e-01 | 0.4672865596 |
|  | 17 | 80200634 | 80200974 | PDFSTATSENSEMBLUCSC | 341 | 4 | 18978 | island | 0.862812 | 0.9685401 | Not queried | 1.46 | 6.86e-01 | 0.7223604052 |
|  | 5 | 140810051 | 140811312 | PDFSTATSENSEMBLUCSC | 1262 | 16 | 31836 | gene;island | 0.028637 | 0.0095387 | Not queried | 1.46 | 8.65e-02 | 0.3104222821 |
|  | 11 | 61447487 | 61447866 | PDFSTATSENSEMBLUCSC | 380 | 5 | 7336 | tss | 0.034387 | 0.0046789 | Not queried | 1.46 | 9.52e-02 | 0.3104222821 |
|  | 11 | 1274018 | 1274269 | PDFSTATSENSEMBLUCSC | 252 | 4 | 6406 | gene;island | 0.618844 | 0.6427314 | Not queried | 1.46 | 8.86e-01 | 0.8941250130 |
|  | 12 | 52400530 | 52400667 | PDFSTATSENSEMBLUCSC | 138 | 4 | 9722 | tss;island | 0.002536 | 0.0040654 | Not queried | 1.46 | 6.86e-01 | 0.7223604052 |
|  | 17 | 58677502 | 58677584 | PDFSTATSENSEMBLUCSC | 83 | 4 | 18625 | island | 0.011032 | 0.0047564 | Not queried | 1.46 | 6.86e-01 | 0.7223604052 |
|  | 17 | 33701321 | 33701529 | PDFSTATSENSEMBLUCSC | 209 | 4 | 17268 | tss;island | 0.015628 | 0.0339934 | Not queried | 1.46 | 3.43e-01 | 0.4672865596 |
|  | 18 | 59560365 | 59560495 | PDFSTATSENSEMBLUCSC | 131 | 5 | 19301 | tss | 0.026083 | 0.0611366 | Not queried | 1.46 | 8.41e-01 | 0.8635565145 |
|  | 2 | 1926366 | 1926907 | PDFSTATSENSEMBLUCSC | 542 | 6 | 22541 | gene | 0.090198 | 0.2031822 | Not queried | 1.46 | 2.40e-01 | 0.4133265075 |
|  | 17 | 7486996 | 7487593 | PDFSTATSENSEMBLUCSC | 598 | 5 | 16403 | gene | 0.005903 | 0.0028136 | Not queried | 1.46 | 3.10e-01 | 0.4672865596 |
|  | 1 | 156084490 | 156084750 | PDFSTATSENSEMBLUCSC | 261 | 5 | 814 | gene | 0.004857 | 0.0037934 | Not queried | 1.46 | 4.21e-01 | 0.5404252807 |
|  | 11 | 15136059 | 15136475 | PDFSTATSENSEMBLUCSC | 417 | 7 | 7169 | tss | 0.290035 | 0.3028075 | Not queried | 1.46 | 9.02e-01 | 0.9091904204 |
|  | 17 | 32483882 | 32484259 | PDFSTATSENSEMBLUCSC | 378 | 10 | 17258 | tss | 0.026378 | 0.0913390 | Not queried | 1.46 | 4.81e-01 | 0.5640816327 |
|  | 5 | 140254793 | 140255069 | PDFSTATSENSEMBLUCSC | 277 | 4 | 31802 | gene;island | 0.110291 | 0.2927317 | Not queried | 1.46 | 4.86e-01 | 0.5640816327 |
|  | 1 | 4832352 | 4832593 | PDFSTATSENSEMBLUCSC | 242 | 5 | 153 | gene;island | 0.533579 | 0.6081855 | Not queried | 1.46 | 4.21e-01 | 0.5404252807 |
|  | 22 | 44287772 | 44288155 | PDFSTATSENSEMBLUCSC | 384 | 8 | 27560 | island | 0.021550 | 0.1798303 | Not queried | 1.46 | 1.48e-02 | 0.1830867741 |
|  | 6 | 6004207 | 6004464 | PDFSTATSENSEMBLUCSC | 258 | 4 | 33544 | gene;island | 0.107414 | 0.0532324 | Not queried | 1.46 | 1.14e-01 | 0.3123457953 |
|  | 18 | 47792708 | 47793175 | PDFSTATSENSEMBLUCSC | 468 | 8 | 19508 | island | 0.016454 | 0.0275478 | Not queried | 1.46 | 5.74e-01 | 0.6460388787 |
|  | 4 | 10686394 | 10686610 | PDFSTATSENSEMBLUCSC | 217 | 4 | 30301 | tss | 0.016735 | 0.0339472 | Not queried | 1.46 | 2.00e-01 | 0.3826488233 |
|  | 5 | 178593785 | 178594649 | PDFSTATSENSEMBLUCSC | 865 | 9 | 31929 | gene;island | 0.072024 | 0.2156889 | Not queried | 1.46 | 7.70e-02 | 0.2825346647 |
|  | 7 | 16793463 | 16793736 | PDFSTATSENSEMBLUCSC | 274 | 4 | 36906 | gene | 0.089252 | 0.0361802 | Not queried | 1.46 | 3.43e-01 | 0.4672865596 |
|  | 6 | 32172354 | 32172871 | PDFSTATSENSEMBLUCSC | 518 | 5 | 34089 | gene | 0.213213 | 0.2714793 | Not queried | 1.46 | 5.48e-01 | 0.6179718485 |
|  | 11 | 70048796 | 70049203 | PDFSTATSENSEMBLUCSC | 408 | 11 | 7526 | tss;island | 0.068922 | 0.0920677 | Not queried | 1.46 | 5.62e-01 | 0.6339017597 |
|  | 6 | 134158982 | 134159412 | PDFSTATSENSEMBLUCSC | 431 | 5 | 34478 | gene;island | 0.004665 | 0.0040842 | Not queried | 1.46 | 6.90e-01 | 0.7223604052 |
|  | 6 | 169629684 | 169630013 | PDFSTATSENSEMBLUCSC | 330 | 7 | 34566 | gene;island | 0.846557 | 0.8533990 | Not queried | 1.46 | 3.83e-01 | 0.5168893301 |
|  | 1 | 155904084 | 155904158 | PDFSTATSENSEMBLUCSC | 75 | 4 | 809 | gene | 0.002366 | 0.0075573 | Not queried | 1.46 | 3.43e-01 | 0.4672865596 |
|  | 8 | 114447055 | 114447871 | PDFSTATSENSEMBLUCSC | 817 | 6 | 39402 | gene;island | 0.003015 | 0.0033010 | Not queried | 1.46 | 5.89e-01 | 0.6570484697 |
|  | 1 | 111022652 | 111022914 | PDFSTATSENSEMBLUCSC | 263 | 4 | 1816 | tss | 0.091135 | 0.2317514 | Not queried | 1.46 | 8.86e-01 | 0.8941250130 |
|  | 22 | 17956453 | 17956560 | PDFSTATSENSEMBLUCSC | 108 | 4 | 26943 | tss | 0.902672 | 0.7194816 | Not queried | 1.46 | 4.86e-01 | 0.5640816327 |
|  | 17 | 44060252 | 44060502 | PDFSTATSENSEMBLUCSC | 251 | 4 | 16642 | gene;island | 0.525803 | 0.8237045 | Not queried | 1.46 | 1.14e-01 | 0.3123457953 |
|  | 11 | 2883529 | 2883912 | PDFSTATSENSEMBLUCSC | 384 | 4 | 7974 | island | 0.414326 | 0.5945703 | Not queried | 1.46 | 6.86e-01 | 0.7223604052 |
|  | 20 | 825050 | 825798 | PDFSTATSENSEMBLUCSC | 749 | 10 | 25324 | gene;island | 0.014375 | 0.0369399 | Not queried | 1.46 | 1.05e-01 | 0.3123457953 |
|  | 8 | 25898191 | 25898539 | PDFSTATSENSEMBLUCSC | 349 | 4 | 39250 | gene;island | 0.039931 | 0.1061560 | Not queried | 1.46 | 4.86e-01 | 0.5640816327 |
|  | 6 | 167370215 | 167370276 | PDFSTATSENSEMBLUCSC | 62 | 4 | 35340 | tss;island | 0.001946 | 0.0059997 | Not queried | 1.46 | 2.00e-01 | 0.3826488233 |
|  | 1 | 94703079 | 94703507 | PDFSTATSENSEMBLUCSC | 429 | 10 | 3499 | island | 0.029608 | 0.0180700 | Not queried | 1.46 | 3.93e-01 | 0.5236526859 |
|  | 6 | 125475016 | 125475385 | PDFSTATSENSEMBLUCSC | 370 | 8 | 34465 | gene | 0.011325 | 0.0207548 | Not queried | 1.46 | 3.28e-01 | 0.4672865596 |
|  | 11 | 3173868 | 3174128 | PDFSTATSENSEMBLUCSC | 261 | 5 | 6475 | gene | 0.437938 | 0.6331116 | Not queried | 1.46 | 5.56e-02 | 0.2315877671 |
|  | 6 | 108487063 | 108487183 | PDFSTATSENSEMBLUCSC | 121 | 6 | 35158 | tss;island | 0.088953 | 0.0326959 | Not queried | 1.46 | 3.10e-01 | 0.4672865596 |
|  | 14 | 23235611 | 23235738 | PDFSTATSENSEMBLUCSC | 128 | 5 | 12700 | island | 0.005792 | 0.0016283 | Not queried | 1.46 | 2.22e-01 | 0.3935758299 |
|  | 8 | 49833685 | 49833956 | PDFSTATSENSEMBLUCSC | 272 | 4 | 39302 | gene;island | 0.059268 | 0.0095251 | Not queried | 1.46 | 1.14e-01 | 0.3123457953 |
|  | 13 | 36920644 | 36921174 | PDFSTATSENSEMBLUCSC | 531 | 13 | 11131 | gene;island | 0.004275 | 0.0072987 | Not queried | 1.45 | 1.39e-01 | 0.3560599333 |
|  | 13 | 91826885 | 91827042 | PDFSTATSENSEMBLUCSC | 158 | 4 | 11812 | island | 0.669580 | 0.8938454 | Not queried | 1.45 | 3.43e-01 | 0.4672865596 |
|  | 19 | 57741988 | 57742423 | PDFSTATSENSEMBLUCSC | 436 | 9 | 21107 | tss | 0.925233 | 0.7853748 | Not queried | 1.45 | 2.22e-01 | 0.3935758299 |
|  | 2 | 132439861 | 132440298 | PDFSTATSENSEMBLUCSC | 438 | 7 | 24737 | island | 0.051002 | 0.0508964 | Not queried | 1.45 | 7.10e-01 | 0.7390730975 |
|  | 19 | 17137685 | 17137714 | PDFSTATSENSEMBLUCSC | 30 | 4 | 20578 | tss | 0.002796 | 0.0122255 | Not queried | 1.45 | 4.86e-01 | 0.5640816327 |
|  | 1 | 63989080 | 63989278 | PDFSTATSENSEMBLUCSC | 199 | 5 | 565 | gene | 0.006232 | 0.0034583 | Not queried | 1.45 | 2.22e-01 | 0.3935758299 |
|  | 2 | 42396143 | 42396458 | PDFSTATSENSEMBLUCSC | 316 | 8 | 23319 | tss;island | 0.014713 | 0.0514026 | Not queried | 1.45 | 7.98e-01 | 0.8290400976 |
|  | 13 | 92001440 | 92001959 | PDFSTATSENSEMBLUCSC | 520 | 5 | 11188 | gene;tss;island | 0.003461 | 0.0010040 | Not queried | 1.45 | 2.22e-01 | 0.3935758299 |
|  | 19 | 12997997 | 12998135 | PDFSTATSENSEMBLUCSC | 139 | 4 | 21632 | island | 0.661539 | 0.8286002 | Not queried | 1.45 | 2.86e-02 | 0.1830867741 |
|  | 6 | 55444061 | 55444488 | PDFSTATSENSEMBLUCSC | 428 | 7 | 35043 | tss | 0.124939 | 0.3019253 | Not queried | 1.45 | 3.83e-01 | 0.5168893301 |
|  | 7 | 18067508 | 18067537 | PDFSTATSENSEMBLUCSC | 30 | 5 | 37457 | tss | 0.978732 | 0.9728710 | Not queried | 1.45 | 5.48e-01 | 0.6179718485 |
|  | 7 | 38670804 | 38671001 | PDFSTATSENSEMBLUCSC | 198 | 6 | 36972 | gene;island | 0.043646 | 0.0253980 | Not queried | 1.45 | 3.10e-01 | 0.4672865596 |
|  | 5 | 178368071 | 178368205 | PDFSTATSENSEMBLUCSC | 135 | 6 | 32545 | tss | 0.365431 | 0.1778000 | Not queried | 1.45 | 4.85e-01 | 0.5640816327 |
|  | 1 | 8938701 | 8938754 | PDFSTATSENSEMBLUCSC | 54 | 4 | 198 | gene | 0.002770 | 0.0041118 | Not queried | 1.45 | 8.86e-01 | 0.8941250130 |
|  | 8 | 65284438 | 65284519 | PDFSTATSENSEMBLUCSC | 82 | 4 | 40340 | island | 0.012882 | 0.0719369 | Not queried | 1.45 | 4.86e-01 | 0.5640816327 |
|  | 2 | 31805915 | 31806551 | PDFSTATSENSEMBLUCSC | 637 | 8 | 24205 | island | 0.617906 | 0.4528895 | Not queried | 1.45 | 1.61e-01 | 0.3814043454 |
|  | 1 | 117487269 | 117487644 | PDFSTATSENSEMBLUCSC | 376 | 4 | 710 | gene;island | 0.949963 | 0.7389178 | Not queried | 1.45 | 3.43e-01 | 0.4672865596 |
|  | 12 | 101603835 | 101604258 | PDFSTATSENSEMBLUCSC | 424 | 8 | 10717 | island | 0.128156 | 0.0466159 | Not queried | 1.45 | 2.79e-01 | 0.4672865596 |
|  | 8 | 82644373 | 82644668 | PDFSTATSENSEMBLUCSC | 296 | 6 | 39786 | tss | 0.017761 | 0.0059419 | Not queried | 1.45 | 2.40e-01 | 0.4133265075 |
|  | 1 | 151371944 | 151372335 | PDFSTATSENSEMBLUCSC | 392 | 5 | 3745 | island | 0.002110 | 0.0052985 | Not queried | 1.45 | 8.41e-01 | 0.8635565145 |
|  | 2 | 229046325 | 229046785 | PDFSTATSENSEMBLUCSC | 461 | 5 | 25170 | island | 0.140716 | 0.2110473 | Not queried | 1.45 | 8.41e-01 | 0.8635565145 |
|  | 4 | 206112 | 206562 | PDFSTATSENSEMBLUCSC | 451 | 8 | 30714 | island | 0.355295 | 0.4012904 | Not queried | 1.45 | 7.98e-01 | 0.8290400976 |
|  | 3 | 32859377 | 32859587 | PDFSTATSENSEMBLUCSC | 211 | 7 | 28968 | island | 0.097327 | 0.0230958 | Not queried | 1.45 | 1.75e-02 | 0.1830867741 |
|  | 22 | 20004356 | 20004382 | PDFSTATSENSEMBLUCSC | 27 | 6 | 26971 | tss;island | 0.003893 | 0.0022209 | Not queried | 1.45 | 3.10e-01 | 0.4672865596 |
|  | 11 | 131779469 | 131780499 | PDFSTATSENSEMBLUCSC | 1031 | 18 | 6978 | gene;tss;island | 0.037313 | 0.2762719 | Not queried | 1.45 | 4.24e-01 | 0.5451667310 |
|  | 7 | 50849639 | 50849931 | PDFSTATSENSEMBLUCSC | 293 | 4 | 37015 | gene;island | 0.764613 | 0.4614707 | Not queried | 1.45 | 3.43e-01 | 0.4672865596 |
|  | 4 | 115519436 | 115519593 | PDFSTATSENSEMBLUCSC | 158 | 6 | 30558 | tss;island | 0.015226 | 0.0455028 | Not queried | 1.44 | 6.49e-02 | 0.2477578846 |
|  | 17 | 79875985 | 79876287 | PDFSTATSENSEMBLUCSC | 303 | 6 | 18954 | island | 0.010624 | 0.0022474 | Not queried | 1.44 | 3.94e-01 | 0.5236526859 |
|  | 7 | 157791721 | 157792100 | PDFSTATSENSEMBLUCSC | 380 | 4 | 37356 | gene | 0.421483 | 0.6934692 | Not queried | 1.44 | 2.00e-01 | 0.3826488233 |
|  | 17 | 46827458 | 46827626 | PDFSTATSENSEMBLUCSC | 169 | 4 | 18507 | island | 0.102544 | 0.0070173 | Not queried | 1.44 | 4.86e-01 | 0.5640816327 |
|  | 3 | 63263752 | 63263832 | PDFSTATSENSEMBLUCSC | 81 | 4 | 28430 | tss;island | 0.018991 | 0.0771020 | Not queried | 1.44 | 2.00e-01 | 0.3826488233 |
|  | 17 | 7328520 | 7328921 | PDFSTATSENSEMBLUCSC | 402 | 6 | 17081 | tss | 0.279460 | 0.4931755 | Not queried | 1.44 | 1.80e-01 | 0.3826488233 |
|  | 21 | 38593636 | 38593754 | PDFSTATSENSEMBLUCSC | 119 | 4 | 26678 | island | 0.003741 | 0.0153592 | Not queried | 1.44 | 8.86e-01 | 0.8941250130 |
|  | 14 | 76452112 | 76452459 | PDFSTATSENSEMBLUCSC | 348 | 4 | 12148 | gene | 0.009232 | 0.0040705 | Not queried | 1.44 | 3.43e-01 | 0.4672865596 |
|  | 7 | 80548695 | 80548752 | PDFSTATSENSEMBLUCSC | 58 | 5 | 37675 | tss;island | 0.018082 | 0.0781476 | Not queried | 1.44 | 4.21e-01 | 0.5404252807 |
|  | 7 | 75795931 | 75796235 | PDFSTATSENSEMBLUCSC | 305 | 4 | 38594 | island | 0.008364 | 0.0474171 | Not queried | 1.44 | 3.43e-01 | 0.4672865596 |
|  | 5 | 157170413 | 157170601 | PDFSTATSENSEMBLUCSC | 189 | 5 | 32458 | tss;island | 0.002139 | 0.0027530 | Not queried | 1.44 | 4.21e-01 | 0.5404252807 |
|  | 22 | 19743759 | 19744239 | PDFSTATSENSEMBLUCSC | 481 | 8 | 27304 | island | 0.312195 | 0.5137342 | Not queried | 1.44 | 3.82e-01 | 0.5168893301 |
|  | 2 | 60812505 | 60812753 | PDFSTATSENSEMBLUCSC | 249 | 4 | 24322 | island | 0.622732 | 0.8225839 | Not queried | 1.44 | 4.86e-01 | 0.5640816327 |
|  | 1 | 38230769 | 38230887 | PDFSTATSENSEMBLUCSC | 119 | 6 | 3073 | island | 0.030313 | 0.1148596 | Not queried | 1.44 | 9.31e-02 | 0.3104222821 |
|  | 19 | 54466187 | 54466724 | PDFSTATSENSEMBLUCSC | 538 | 6 | 22391 | island | 0.026752 | 0.0945828 | Not queried | 1.44 | 4.85e-01 | 0.5640816327 |
|  | 1 | 183387128 | 183387751 | PDFSTATSENSEMBLUCSC | 624 | 6 | 4031 | island | 0.050074 | 0.0178258 | Not queried | 1.44 | 1.80e-01 | 0.3826488233 |
|  | 2 | 242212422 | 242212528 | PDFSTATSENSEMBLUCSC | 107 | 5 | 23133 | gene;tss;island | 0.007516 | 0.0180498 | Not queried | 1.44 | 6.90e-01 | 0.7223604052 |
|  | 2 | 46707626 | 46707844 | PDFSTATSENSEMBLUCSC | 219 | 4 | 22667 | gene;island | 0.848625 | 0.9339356 | Not queried | 1.44 | 4.86e-01 | 0.5640816327 |
|  | 6 | 32301073 | 32301513 | PDFSTATSENSEMBLUCSC | 441 | 4 | 34103 | gene | 0.657437 | 0.8325990 | Not queried | 1.44 | 3.43e-01 | 0.4672865596 |
|  | 8 | 145027948 | 145028505 | PDFSTATSENSEMBLUCSC | 558 | 10 | 39504 | gene;island | 0.284765 | 0.2611669 | Not queried | 1.44 | 6.31e-01 | 0.7014147777 |
|  | 17 | 76506489 | 76506855 | PDFSTATSENSEMBLUCSC | 367 | 8 | 16823 | gene | 0.741228 | 0.8951445 | Not queried | 1.44 | 1.30e-01 | 0.3397986247 |
|  | 2 | 105858730 | 105859083 | PDFSTATSENSEMBLUCSC | 354 | 4 | 22813 | gene;island | 0.672949 | 0.7292338 | Not queried | 1.44 | 6.86e-01 | 0.7223604052 |
|  | 21 | 43823604 | 43823863 | PDFSTATSENSEMBLUCSC | 260 | 5 | 26568 | tss | 0.910401 | 0.7145502 | Not queried | 1.44 | 2.22e-01 | 0.3935758299 |
|  | 2 | 183942982 | 183943203 | PDFSTATSENSEMBLUCSC | 222 | 4 | 23769 | tss | 0.016802 | 0.0720272 | Not queried | 1.44 | 2.00e-01 | 0.3826488233 |
|  | 6 | 32039796 | 32039999 | PDFSTATSENSEMBLUCSC | 204 | 7 | 34031 | gene | 0.317170 | 0.4462107 | Not queried | 1.44 | 3.18e-01 | 0.4672865596 |
|  | 19 | 5455176 | 5455386 | PDFSTATSENSEMBLUCSC | 211 | 4 | 20376 | tss | 0.595355 | 0.7993355 | Not queried | 1.44 | 2.00e-01 | 0.3826488233 |
|  | 1 | 152161237 | 152161683 | PDFSTATSENSEMBLUCSC | 447 | 4 | 3760 | island | 0.043360 | 0.3777941 | Not queried | 1.44 | 6.86e-01 | 0.7223604052 |
|  | 4 | 7941764 | 7942031 | PDFSTATSENSEMBLUCSC | 268 | 8 | 30294 | tss;island | 0.345957 | 0.7195233 | Not queried | 1.44 | 5.05e-01 | 0.5852592101 |
|  | 14 | 55907299 | 55907501 | PDFSTATSENSEMBLUCSC | 203 | 7 | 12414 | tss | 0.523643 | 0.7181888 | Not queried | 1.44 | 4.56e-01 | 0.5640816327 |
|  | 5 | 175223225 | 175223497 | PDFSTATSENSEMBLUCSC | 273 | 4 | 32508 | tss | 0.171729 | 0.0105323 | Not queried | 1.44 | 3.43e-01 | 0.4672865596 |
|  | 4 | 154710224 | 154711675 | PDFSTATSENSEMBLUCSC | 1452 | 38 | 31377 | island | 0.019601 | 0.0582636 | Not queried | 1.44 | 1.85e-02 | 0.1830867741 |
|  | 16 | 85517170 | 85517684 | PDFSTATSENSEMBLUCSC | 515 | 7 | 16156 | island | 0.470846 | 0.7427880 | Not queried | 1.44 | 2.09e-01 | 0.3935758299 |
|  | 8 | 17013409 | 17013756 | PDFSTATSENSEMBLUCSC | 348 | 4 | 39564 | tss;island | 0.013687 | 0.0064362 | Not queried | 1.44 | 3.43e-01 | 0.4672865596 |
|  | 11 | 65337543 | 65337913 | PDFSTATSENSEMBLUCSC | 371 | 5 | 7432 | tss | 0.002050 | 0.0055576 | Not queried | 1.44 | 6.90e-01 | 0.7223604052 |
|  | 15 | 34610829 | 34611069 | PDFSTATSENSEMBLUCSC | 241 | 4 | 13277 | gene | 0.003161 | 0.0054743 | Not queried | 1.44 | 8.86e-01 | 0.8941250130 |
|  | 21 | 45560663 | 45560984 | PDFSTATSENSEMBLUCSC | 322 | 4 | 26452 | gene;island | 0.630546 | 0.7321486 | Not queried | 1.44 | 6.86e-01 | 0.7223604052 |
|  | 1 | 1356565 | 1356902 | PDFSTATSENSEMBLUCSC | 338 | 4 | 2437 | island | 0.480187 | 0.6390244 | Not queried | 1.44 | 6.86e-01 | 0.7223604052 |
|  | 3 | 44626029 | 44626453 | PDFSTATSENSEMBLUCSC | 425 | 7 | 28257 | tss | 0.015951 | 0.0419183 | Not queried | 1.44 | 2.59e-01 | 0.4381244265 |
|  | 13 | 28674876 | 28675056 | PDFSTATSENSEMBLUCSC | 181 | 4 | 11368 | tss | 0.011387 | 0.0246506 | Not queried | 1.44 | 2.00e-01 | 0.3826488233 |
|  | 8 | 1821246 | 1821603 | PDFSTATSENSEMBLUCSC | 358 | 4 | 39181 | gene;island | 0.898669 | 0.9294502 | Not queried | 1.44 | 4.86e-01 | 0.5640816327 |
|  | 4 | 30721997 | 30722274 | PDFSTATSENSEMBLUCSC | 278 | 4 | 30970 | island | 0.208366 | 0.1329757 | Not queried | 1.44 | 6.86e-01 | 0.7223604052 |
|  | 20 | 43374239 | 43374406 | PDFSTATSENSEMBLUCSC | 168 | 8 | 25763 | tss;island | 0.019277 | 0.1434019 | Not queried | 1.43 | 6.50e-02 | 0.2477578846 |
|  | 3 | 128294970 | 128295141 | PDFSTATSENSEMBLUCSC | 172 | 6 | 28570 | tss | 0.278478 | 0.4425791 | Not queried | 1.43 | 3.10e-01 | 0.4672865596 |
|  | 14 | 32670315 | 32670656 | PDFSTATSENSEMBLUCSC | 342 | 4 | 12770 | island | 0.001605 | 0.0024774 | Not queried | 1.43 | 3.43e-01 | 0.4672865596 |
|  | 2 | 241938159 | 241938193 | PDFSTATSENSEMBLUCSC | 35 | 5 | 23983 | tss | 0.007660 | 0.0032372 | Not queried | 1.43 | 4.21e-01 | 0.5404252807 |
|  | 2 | 241938159 | 241938436 | PDFSTATSENSEMBLUCSC | 278 | 7 | 25283 | island | 0.007660 | 0.0079186 | Not queried | 1.43 | 7.10e-01 | 0.7390730975 |
|  | 2 | 136875897 | 136876188 | PDFSTATSENSEMBLUCSC | 292 | 7 | 23649 | tss;island | 0.003498 | 0.0029000 | Not queried | 1.43 | 3.18e-01 | 0.4672865596 |
|  | 7 | 4304779 | 4305061 | PDFSTATSENSEMBLUCSC | 283 | 4 | 36838 | gene;island | 0.359329 | 0.6048750 | Not queried | 1.43 | 2.00e-01 | 0.3826488233 |
|  | 6 | 29796351 | 29796770 | PDFSTATSENSEMBLUCSC | 420 | 15 | 33697 | gene;island | 0.402293 | 0.5486822 | Not queried | 1.43 | 1.37e-01 | 0.3516378542 |
|  | 21 | 46340776 | 46341067 | PDFSTATSENSEMBLUCSC | 292 | 7 | 26460 | gene | 0.011155 | 0.0297641 | Not queried | 1.43 | 1.65e-01 | 0.3826488233 |
|  | 2 | 242499273 | 242499542 | PDFSTATSENSEMBLUCSC | 270 | 4 | 23136 | gene;island | 0.557243 | 0.4745764 | Not queried | 1.43 | 2.00e-01 | 0.3826488233 |
|  | 16 | 30905695 | 30905875 | PDFSTATSENSEMBLUCSC | 181 | 5 | 15197 | tss;island | 0.090110 | 0.0641778 | Not queried | 1.43 | 5.48e-01 | 0.6179718485 |
|  | 3 | 101498198 | 101498416 | PDFSTATSENSEMBLUCSC | 219 | 6 | 27882 | gene;island | 0.007655 | 0.0021009 | Not queried | 1.43 | 4.85e-01 | 0.5640816327 |
|  | 10 | 28034792 | 28034856 | PDFSTATSENSEMBLUCSC | 65 | 6 | 5004 | tss | 0.099747 | 0.3413192 | Not queried | 1.43 | 1.80e-01 | 0.3826488233 |
|  | 14 | 24801616 | 24802150 | PDFSTATSENSEMBLUCSC | 535 | 4 | 12047 | gene;island | 0.330763 | 0.7215773 | Not queried | 1.43 | 4.86e-01 | 0.5640816327 |
|  | 6 | 42927940 | 42928497 | PDFSTATSENSEMBLUCSC | 558 | 22 | 34985 | tss | 0.063527 | 0.3176952 | Not queried | 1.43 | 1.31e-02 | 0.1830867741 |
|  | 11 | 798350 | 798458 | PDFSTATSENSEMBLUCSC | 109 | 4 | 7881 | island | 0.011990 | 0.0109324 | Not queried | 1.43 | 6.86e-01 | 0.7223604052 |
|  | 15 | 29407792 | 29408145 | PDFSTATSENSEMBLUCSC | 354 | 4 | 13268 | gene;island | 0.067447 | 0.3652578 | Not queried | 1.43 | 3.43e-01 | 0.4672865596 |
|  | 17 | 42019637 | 42019987 | PDFSTATSENSEMBLUCSC | 351 | 5 | 18382 | island | 0.580505 | 0.6797953 | Not queried | 1.43 | 2.22e-01 | 0.3935758299 |
|  | 10 | 102415991 | 102416715 | PDFSTATSENSEMBLUCSC | 725 | 7 | 6028 | island | 0.080828 | 0.5208127 | Not queried | 1.43 | 3.83e-01 | 0.5168893301 |
|  | 4 | 1020943 | 1021291 | PDFSTATSENSEMBLUCSC | 349 | 4 | 30759 | island | 0.692779 | 0.7996747 | Not queried | 1.43 | 6.86e-01 | 0.7223604052 |
|  | 4 | 87515184 | 87515433 | PDFSTATSENSEMBLUCSC | 250 | 8 | 30482 | tss | 0.018867 | 0.0577481 | Not queried | 1.43 | 4.99e-02 | 0.2315877671 |
|  | 6 | 44695195 | 44695536 | PDFSTATSENSEMBLUCSC | 342 | 5 | 36180 | island | 0.851604 | 0.7140631 | Not queried | 1.43 | 2.22e-01 | 0.3935758299 |
|  | 18 | 71958904 | 71959170 | PDFSTATSENSEMBLUCSC | 267 | 4 | 19126 | gene | 0.008520 | 0.0920562 | Not queried | 1.43 | 3.43e-01 | 0.4672865596 |
|  | 4 | 76650351 | 76650666 | PDFSTATSENSEMBLUCSC | 316 | 4 | 30025 | gene;island | 0.007422 | 0.0044851 | Not queried | 1.43 | 4.86e-01 | 0.5640816327 |
|  | 19 | 19651867 | 19652066 | PDFSTATSENSEMBLUCSC | 200 | 4 | 19955 | gene;island | 0.132241 | 0.1872807 | Not queried | 1.43 | 6.86e-01 | 0.7223604052 |
|  | 2 | 228029433 | 228029856 | PDFSTATSENSEMBLUCSC | 424 | 8 | 23058 | gene;tss;island | 0.017290 | 0.0350918 | Not queried | 1.43 | 1.04e-02 | 0.1806165940 |
|  | 3 | 192444985 | 192445380 | PDFSTATSENSEMBLUCSC | 396 | 5 | 28087 | gene | 0.408919 | 0.0812078 | Not queried | 1.43 | 3.10e-01 | 0.4672865596 |
|  | 9 | 124029872 | 124030048 | PDFSTATSENSEMBLUCSC | 177 | 4 | 40834 | tss | 0.593765 | 0.9110425 | Not queried | 1.43 | 4.86e-01 | 0.5640816327 |
|  | 15 | 59949760 | 59950097 | PDFSTATSENSEMBLUCSC | 338 | 8 | 13706 | tss;island | 0.010563 | 0.0115979 | Not queried | 1.43 | 4.42e-01 | 0.5640816327 |
|  | 6 | 32057053 | 32057341 | PDFSTATSENSEMBLUCSC | 289 | 4 | 34042 | gene;island | 0.310767 | 0.5661905 | Not queried | 1.43 | 6.86e-01 | 0.7223604052 |
|  | 16 | 1493343 | 1493859 | PDFSTATSENSEMBLUCSC | 517 | 5 | 14571 | gene;island | 0.930494 | 0.6780917 | Not queried | 1.43 | 2.22e-01 | 0.3935758299 |
|  | 12 | 123319894 | 123319981 | PDFSTATSENSEMBLUCSC | 88 | 4 | 10090 | tss | 0.012611 | 0.0218331 | Not queried | 1.43 | 6.86e-01 | 0.7223604052 |
|  | 17 | 146404 | 146926 | PDFSTATSENSEMBLUCSC | 523 | 5 | 16286 | gene | 0.307926 | 0.4073764 | Not queried | 1.43 | 8.41e-01 | 0.8635565145 |
|  | 17 | 15635376 | 15635468 | PDFSTATSENSEMBLUCSC | 93 | 5 | 17151 | tss | 0.500917 | 0.7299974 | Not queried | 1.43 | 3.17e-02 | 0.1866936846 |
|  | 8 | 85095275 | 85095697 | PDFSTATSENSEMBLUCSC | 423 | 5 | 39788 | tss;island | 0.028763 | 0.0219028 | Not queried | 1.43 | 5.48e-01 | 0.6179718485 |
|  | 5 | 161274307 | 161275138 | PDFSTATSENSEMBLUCSC | 832 | 9 | 31879 | gene;tss | 0.006532 | 0.0296635 | Not queried | 1.43 | 3.87e-01 | 0.5209559659 |
|  | 5 | 44388799 | 44389062 | PDFSTATSENSEMBLUCSC | 264 | 6 | 32052 | tss | 0.029112 | 0.1110427 | Not queried | 1.43 | 5.89e-01 | 0.6570484697 |
|  | 10 | 88730324 | 88730599 | PDFSTATSENSEMBLUCSC | 276 | 4 | 4664 | gene;island | 0.031482 | 0.0854891 | Not queried | 1.43 | 4.86e-01 | 0.5640816327 |
|  | 7 | 70597458 | 70597778 | PDFSTATSENSEMBLUCSC | 321 | 6 | 37627 | tss | 0.250653 | 0.1453060 | Not queried | 1.43 | 1.80e-01 | 0.3826488233 |
|  | 6 | 37225949 | 37226214 | PDFSTATSENSEMBLUCSC | 266 | 5 | 34321 | gene;tss;island | 0.001606 | 0.0016975 | Not queried | 1.43 | 5.48e-01 | 0.6179718485 |
|  | 11 | 115530932 | 115531299 | PDFSTATSENSEMBLUCSC | 368 | 5 | 8830 | island | 0.027817 | 0.0079496 | Not queried | 1.43 | 3.10e-01 | 0.4672865596 |
|  | 1 | 26605913 | 26606110 | PDFSTATSENSEMBLUCSC | 198 | 4 | 1388 | tss;island | 0.113943 | 0.0527164 | Not queried | 1.43 | 6.86e-01 | 0.7223604052 |
|  | 19 | 3606835 | 3607269 | PDFSTATSENSEMBLUCSC | 435 | 7 | 20348 | tss | 0.113927 | 0.1548680 | Not queried | 1.42 | 7.28e-02 | 0.2713481487 |
|  | 4 | 74847758 | 74848016 | PDFSTATSENSEMBLUCSC | 259 | 6 | 30431 | tss | 0.384563 | 0.6089371 | Not queried | 1.42 | 3.94e-01 | 0.5236526859 |
|  | 5 | 177412586 | 177412718 | PDFSTATSENSEMBLUCSC | 133 | 4 | 33462 | island | 0.142418 | 0.1649053 | Not queried | 1.42 | 6.86e-01 | 0.7223604052 |
|  | 1 | 68629000 | 68629133 | PDFSTATSENSEMBLUCSC | 134 | 4 | 575 | gene;island | 0.739661 | 0.5518637 | Not queried | 1.42 | 1.14e-01 | 0.3123457953 |
|  | 2 | 27341795 | 27341901 | PDFSTATSENSEMBLUCSC | 107 | 4 | 22620 | gene;tss;island | 0.027516 | 0.0105474 | Not queried | 1.42 | 3.43e-01 | 0.4672865596 |
|  | 14 | 37125292 | 37125997 | PDFSTATSENSEMBLUCSC | 706 | 8 | 12360 | tss | 0.310439 | 0.2258883 | Not queried | 1.42 | 4.42e-01 | 0.5640816327 |
|  | 10 | 63213339 | 63213510 | PDFSTATSENSEMBLUCSC | 172 | 5 | 5097 | tss | 0.107972 | 0.4735550 | Not queried | 1.42 | 4.21e-01 | 0.5404252807 |
|  | 10 | 123353298 | 123353956 | PDFSTATSENSEMBLUCSC | 659 | 12 | 4793 | gene;island | 0.684553 | 0.8153415 | Not queried | 1.42 | 2.42e-01 | 0.4149845107 |
|  | 1 | 29062689 | 29063076 | PDFSTATSENSEMBLUCSC | 388 | 7 | 1436 | tss;island | 0.006550 | 0.0073647 | Not queried | 1.42 | 6.20e-01 | 0.6899391537 |
|  | 22 | 48977177 | 48977448 | PDFSTATSENSEMBLUCSC | 272 | 4 | 26919 | gene;island | 0.496340 | 0.7375622 | Not queried | 1.42 | 2.00e-01 | 0.3826488233 |
|  | 3 | 136537607 | 136537863 | PDFSTATSENSEMBLUCSC | 257 | 9 | 29508 | island | 0.046786 | 0.0155327 | Not queried | 1.42 | 9.39e-02 | 0.3104222821 |
|  | 11 | 12398874 | 12399109 | PDFSTATSENSEMBLUCSC | 236 | 7 | 7154 | tss | 0.050502 | 0.0827144 | Not queried | 1.42 | 9.73e-02 | 0.3123457953 |
|  | 15 | 25414716 | 25414781 | PDFSTATSENSEMBLUCSC | 66 | 5 | 13526 | tss | 0.591736 | 0.7783913 | Not queried | 1.42 | 3.17e-02 | 0.1866936846 |
|  | 10 | 80733663 | 80734012 | PDFSTATSENSEMBLUCSC | 350 | 5 | 4648 | gene;island | 0.018666 | 0.0057314 | Not queried | 1.42 | 4.21e-01 | 0.5404252807 |
|  | 2 | 74753691 | 74753946 | PDFSTATSENSEMBLUCSC | 256 | 4 | 23462 | tss;island | 0.128613 | 0.5449061 | Not queried | 1.42 | 4.86e-01 | 0.5640816327 |
|  | 1 | 1992046 | 1992687 | PDFSTATSENSEMBLUCSC | 642 | 5 | 54 | gene;island | 0.542900 | 0.7346494 | Not queried | 1.42 | 5.48e-01 | 0.6179718485 |
|  | 17 | 1549098 | 1549434 | PDFSTATSENSEMBLUCSC | 337 | 4 | 16989 | tss | 0.438392 | 0.8442088 | Not queried | 1.42 | 6.86e-01 | 0.7223604052 |
|  | 19 | 15618961 | 15619355 | PDFSTATSENSEMBLUCSC | 395 | 5 | 21709 | island | 0.032841 | 0.0792637 | Not queried | 1.42 | 8.41e-01 | 0.8635565145 |
|  | 7 | 98099806 | 98100419 | PDFSTATSENSEMBLUCSC | 614 | 7 | 38713 | island | 0.110714 | 0.0256934 | Not queried | 1.42 | 1.65e-01 | 0.3826488233 |
|  | 2 | 27193211 | 27193496 | PDFSTATSENSEMBLUCSC | 286 | 6 | 23248 | tss | 0.002727 | 0.0051713 | Not queried | 1.42 | 3.94e-01 | 0.5236526859 |
|  | 19 | 53141620 | 53141793 | PDFSTATSENSEMBLUCSC | 174 | 4 | 20198 | gene;island | 0.050262 | 0.0254702 | Not queried | 1.42 | 3.43e-01 | 0.4672865596 |
|  | 11 | 4629034 | 4629432 | PDFSTATSENSEMBLUCSC | 399 | 4 | 6484 | gene | 0.018507 | 0.0059650 | Not queried | 1.42 | 4.86e-01 | 0.5640816327 |
|  | 19 | 2702863 | 2703086 | PDFSTATSENSEMBLUCSC | 224 | 10 | 20329 | tss;island | 0.008707 | 0.0026313 | Not queried | 1.42 | 2.47e-01 | 0.4242889032 |
|  | 7 | 14029285 | 14029701 | PDFSTATSENSEMBLUCSC | 417 | 8 | 36899 | gene;island | 0.014668 | 0.0141362 | Not queried | 1.42 | 7.98e-01 | 0.8290400976 |
|  | 7 | 1351539 | 1352002 | PDFSTATSENSEMBLUCSC | 464 | 8 | 38098 | island | 0.710303 | 0.9255634 | Not queried | 1.42 | 1.61e-01 | 0.3814043454 |
|  | 7 | 22705331 | 22705607 | PDFSTATSENSEMBLUCSC | 277 | 4 | 38301 | island | 0.768806 | 0.9783582 | Not queried | 1.42 | 3.43e-01 | 0.4672865596 |
|  | 18 | 13726491 | 13726506 | PDFSTATSENSEMBLUCSC | 16 | 4 | 19063 | gene | 0.003567 | 0.0018042 | Not queried | 1.42 | 4.86e-01 | 0.5640816327 |
|  | 10 | 103603722 | 103604019 | PDFSTATSENSEMBLUCSC | 298 | 5 | 5312 | tss | 0.088622 | 0.0221892 | Not queried | 1.41 | 2.22e-01 | 0.3935758299 |
|  | 8 | 124194847 | 124195192 | PDFSTATSENSEMBLUCSC | 346 | 5 | 39417 | gene | 0.529235 | 0.7488696 | Not queried | 1.41 | 4.21e-01 | 0.5404252807 |
|  | 5 | 37834909 | 37835168 | PDFSTATSENSEMBLUCSC | 260 | 5 | 32025 | tss | 0.015303 | 0.0112806 | Not queried | 1.41 | 4.21e-01 | 0.5404252807 |
|  | 4 | 159131324 | 159131785 | PDFSTATSENSEMBLUCSC | 462 | 7 | 31396 | island | 0.179342 | 0.0223667 | Not queried | 1.41 | 3.18e-01 | 0.4672865596 |
|  | 5 | 33936171 | 33936752 | PDFSTATSENSEMBLUCSC | 582 | 13 | 32723 | island | 0.209477 | 0.1119203 | Not queried | 1.41 | 1.40e-02 | 0.1830867741 |
|  | 5 | 168271613 | 168272051 | PDFSTATSENSEMBLUCSC | 439 | 7 | 31887 | gene | 0.910435 | 0.9707438 | Not queried | 1.41 | 3.18e-01 | 0.4672865596 |
|  | 11 | 129939665 | 129939713 | PDFSTATSENSEMBLUCSC | 49 | 4 | 7816 | tss | 0.009789 | 0.0133581 | Not queried | 1.41 | 6.86e-01 | 0.7223604052 |
|  | 8 | 70378380 | 70378760 | PDFSTATSENSEMBLUCSC | 381 | 6 | 39752 | tss | 0.005704 | 0.0047013 | Not queried | 1.41 | 9.37e-01 | 0.9445195058 |
|  | 19 | 48216390 | 48216502 | PDFSTATSENSEMBLUCSC | 113 | 5 | 20908 | tss | 0.007241 | 0.0380320 | Not queried | 1.41 | 3.10e-01 | 0.4672865596 |
|  | 10 | 124578028 | 124578544 | PDFSTATSENSEMBLUCSC | 517 | 5 | 6210 | island | 0.351666 | 0.5091883 | Not queried | 1.41 | 8.41e-01 | 0.8635565145 |
|  | 2 | 171671480 | 171671795 | PDFSTATSENSEMBLUCSC | 316 | 4 | 24849 | island | 0.212698 | 0.2004900 | Not queried | 1.41 | 8.86e-01 | 0.8941250130 |
|  | 6 | 7585711 | 7585896 | PDFSTATSENSEMBLUCSC | 186 | 4 | 33553 | gene | 0.742103 | 0.9058017 | Not queried | 1.41 | 3.43e-01 | 0.4672865596 |
|  | 7 | 157447022 | 157447249 | PDFSTATSENSEMBLUCSC | 228 | 4 | 37335 | gene;island | 0.931057 | 0.9635898 | Not queried | 1.41 | 2.00e-01 | 0.3826488233 |
|  | 11 | 65554042 | 65554410 | PDFSTATSENSEMBLUCSC | 369 | 8 | 7441 | tss;island | 0.024382 | 0.0120805 | Not queried | 1.41 | 1.61e-01 | 0.3814043454 |
|  | 7 | 29725137 | 29725196 | PDFSTATSENSEMBLUCSC | 60 | 4 | 37513 | tss;island | 0.003741 | 0.0020454 | Not queried | 1.41 | 6.86e-01 | 0.7223604052 |
|  | 5 | 59189649 | 59189934 | PDFSTATSENSEMBLUCSC | 286 | 8 | 32079 | tss | 0.105468 | 0.3384295 | Not queried | 1.41 | 1.30e-01 | 0.3397986247 |
|  | 15 | 40633194 | 40633416 | PDFSTATSENSEMBLUCSC | 223 | 4 | 13586 | tss | 0.496786 | 0.0903798 | Not queried | 1.41 | 6.86e-01 | 0.7223604052 |
|  | 3 | 9594151 | 9594385 | PDFSTATSENSEMBLUCSC | 235 | 4 | 27653 | gene;island | 0.150050 | 0.2561690 | Not queried | 1.41 | 4.86e-01 | 0.5640816327 |
|  | 8 | 1106401 | 1106743 | PDFSTATSENSEMBLUCSC | 343 | 5 | 40014 | island | 0.315230 | 0.5651591 | Not queried | 1.41 | 9.52e-02 | 0.3104222821 |
|  | 5 | 191127 | 191806 | PDFSTATSENSEMBLUCSC | 680 | 11 | 32573 | island | 0.161198 | 0.0482724 | Not queried | 1.41 | 7.59e-02 | 0.2811835917 |
|  | 17 | 80289500 | 80289701 | PDFSTATSENSEMBLUCSC | 202 | 4 | 16943 | gene;island | 0.551007 | 0.7125547 | Not queried | 1.41 | 4.86e-01 | 0.5640816327 |
|  | 11 | 15095017 | 15095178 | PDFSTATSENSEMBLUCSC | 162 | 6 | 8082 | island | 0.135921 | 0.0280108 | Not queried | 1.41 | 2.40e-01 | 0.4133265075 |
|  | 14 | 105154557 | 105155327 | PDFSTATSENSEMBLUCSC | 771 | 7 | 12660 | tss;island | 0.675261 | 0.9098583 | Not queried | 1.41 | 2.59e-01 | 0.4381244265 |
|  | 10 | 18999145 | 18999425 | PDFSTATSENSEMBLUCSC | 281 | 4 | 5587 | island | 0.870556 | 0.9203482 | Not queried | 1.41 | 4.86e-01 | 0.5640816327 |
|  | 11 | 47359839 | 47360291 | PDFSTATSENSEMBLUCSC | 453 | 6 | 6606 | gene;island | 0.556343 | 0.7094522 | Not queried | 1.41 | 1.80e-01 | 0.3826488233 |
|  | 6 | 168418276 | 168418527 | PDFSTATSENSEMBLUCSC | 252 | 7 | 35346 | tss | 0.192324 | 0.3800862 | Not queried | 1.41 | 2.59e-01 | 0.4381244265 |
|  | 1 | 190447232 | 190447640 | PDFSTATSENSEMBLUCSC | 409 | 5 | 2147 | tss | 0.016353 | 0.0018712 | Not queried | 1.41 | 2.22e-01 | 0.3935758299 |
|  | 4 | 85422998 | 85423302 | PDFSTATSENSEMBLUCSC | 305 | 4 | 31162 | island | 0.030936 | 0.0766657 | Not queried | 1.41 | 8.86e-01 | 0.8941250130 |
|  | 8 | 1404885 | 1405138 | PDFSTATSENSEMBLUCSC | 254 | 4 | 40025 | island | 0.457200 | 0.5885661 | Not queried | 1.41 | 4.86e-01 | 0.5640816327 |
|  | 19 | 38754529 | 38754930 | PDFSTATSENSEMBLUCSC | 402 | 5 | 20748 | tss | 0.032536 | 0.0758652 | Not queried | 1.41 | 9.52e-02 | 0.3104222821 |
|  | 20 | 4702381 | 4702538 | PDFSTATSENSEMBLUCSC | 158 | 4 | 25575 | tss | 0.616359 | 0.8038331 | Not queried | 1.41 | 2.00e-01 | 0.3826488233 |
|  | 11 | 393328 | 393648 | PDFSTATSENSEMBLUCSC | 321 | 5 | 7010 | tss;island | 0.309728 | 0.5428290 | Not queried | 1.41 | 5.48e-01 | 0.6179718485 |
|  | 10 | 44788627 | 44789077 | PDFSTATSENSEMBLUCSC | 451 | 5 | 5706 | island | 0.200488 | 0.3441300 | Not queried | 1.41 | 3.10e-01 | 0.4672865596 |
|  | 19 | 33571484 | 33571768 | PDFSTATSENSEMBLUCSC | 285 | 4 | 20681 | tss | 0.112795 | 0.4003822 | Not queried | 1.41 | 4.86e-01 | 0.5640816327 |
|  | 5 | 145316063 | 145316416 | PDFSTATSENSEMBLUCSC | 354 | 8 | 33277 | island | 0.029734 | 0.0582972 | Not queried | 1.41 | 1.30e-01 | 0.3397986247 |
|  | 6 | 33039396 | 33039804 | PDFSTATSENSEMBLUCSC | 409 | 7 | 34182 | gene | 0.006133 | 0.0095460 | Not queried | 1.41 | 6.20e-01 | 0.6899391537 |
|  | 19 | 36035831 | 36036175 | PDFSTATSENSEMBLUCSC | 345 | 5 | 19985 | gene | 0.028583 | 0.0101604 | Not queried | 1.41 | 5.56e-02 | 0.2315877671 |
|  | 19 | 36035831 | 36036387 | PDFSTATSENSEMBLUCSC | 557 | 7 | 20707 | tss | 0.018647 | 0.0101604 | Not queried | 1.41 | 1.65e-01 | 0.3826488233 |
|  | 19 | 58609269 | 58610131 | PDFSTATSENSEMBLUCSC | 863 | 15 | 20256 | gene;island | 0.060726 | 0.0222134 | Not queried | 1.41 | 2.95e-02 | 0.1866936846 |
|  | 11 | 36615733 | 36616365 | PDFSTATSENSEMBLUCSC | 633 | 9 | 6586 | gene | 0.056648 | 0.1365319 | Not queried | 1.41 | 4.36e-01 | 0.5589686471 |
|  | 8 | 70984199 | 70984559 | PDFSTATSENSEMBLUCSC | 361 | 4 | 39758 | tss;island | 0.118963 | 0.0465488 | Not queried | 1.41 | 5.71e-02 | 0.2315877671 |
|  | 19 | 41882232 | 41882327 | PDFSTATSENSEMBLUCSC | 96 | 4 | 20059 | gene;tss;island | 0.020995 | 0.0504424 | Not queried | 1.41 | 2.00e-01 | 0.3826488233 |
|  | 16 | 3142645 | 3143250 | PDFSTATSENSEMBLUCSC | 606 | 8 | 15636 | island | 0.590895 | 0.7512143 | Not queried | 1.41 | 4.42e-01 | 0.5640816327 |
|  | 20 | 42574332 | 42574362 | PDFSTATSENSEMBLUCSC | 31 | 4 | 25423 | gene;tss | 0.878759 | 0.9003424 | Not queried | 1.41 | 8.86e-01 | 0.8941250130 |
|  | 4 | 146403866 | 146404134 | PDFSTATSENSEMBLUCSC | 269 | 8 | 30146 | gene;island | 0.028988 | 0.0014241 | Not queried | 1.41 | 1.61e-01 | 0.3814043454 |
|  | 6 | 29523592 | 29524117 | PDFSTATSENSEMBLUCSC | 526 | 8 | 33660 | gene;island | 0.431393 | 0.7039538 | Not queried | 1.41 | 4.99e-02 | 0.2315877671 |
|  | 14 | 62228873 | 62229059 | PDFSTATSENSEMBLUCSC | 187 | 6 | 12445 | tss | 0.009257 | 0.0026083 | Not queried | 1.40 | 3.10e-01 | 0.4672865596 |
|  | 3 | 11058836 | 11059050 | PDFSTATSENSEMBLUCSC | 215 | 6 | 27665 | gene;island | 0.312104 | 0.5860570 | Not queried | 1.40 | 9.31e-02 | 0.3104222821 |
|  | 4 | 174459743 | 174460124 | PDFSTATSENSEMBLUCSC | 382 | 4 | 30182 | gene;island | 0.242621 | 0.0545611 | Not queried | 1.40 | 6.86e-01 | 0.7223604052 |
|  | 7 | 8301971 | 8302301 | PDFSTATSENSEMBLUCSC | 331 | 8 | 37438 | tss;island | 0.046914 | 0.1094109 | Not queried | 1.40 | 1.05e-01 | 0.3123457953 |
|  | 20 | 62151993 | 62152146 | PDFSTATSENSEMBLUCSC | 154 | 5 | 26354 | island | 0.001655 | 0.0047575 | Not queried | 1.40 | 4.21e-01 | 0.5404252807 |
|  | 19 | 49660986 | 49661083 | PDFSTATSENSEMBLUCSC | 98 | 4 | 22257 | island | 0.014077 | 0.0204678 | Not queried | 1.40 | 6.86e-01 | 0.7223604052 |
|  | 21 | 46812996 | 46813298 | PDFSTATSENSEMBLUCSC | 303 | 4 | 26753 | island | 0.541063 | 0.6589324 | Not queried | 1.40 | 6.86e-01 | 0.7223604052 |
|  | 7 | 155326713 | 155326803 | PDFSTATSENSEMBLUCSC | 91 | 4 | 38012 | tss;island | 0.017807 | 0.0440460 | Not queried | 1.40 | 6.86e-01 | 0.7223604052 |
|  | 7 | 1039875 | 1040260 | PDFSTATSENSEMBLUCSC | 386 | 4 | 36731 | gene;island | 0.209147 | 0.5178229 | Not queried | 1.40 | 3.43e-01 | 0.4672865596 |
|  | 19 | 41510026 | 41510285 | PDFSTATSENSEMBLUCSC | 260 | 7 | 20052 | gene | 0.689868 | 0.8471915 | Not queried | 1.40 | 3.83e-01 | 0.5168893301 |
|  | 3 | 48700269 | 48700498 | PDFSTATSENSEMBLUCSC | 230 | 8 | 29095 | island | 0.029456 | 0.0139085 | Not queried | 1.40 | 2.34e-01 | 0.4128374386 |
|  | 2 | 121554672 | 121554817 | PDFSTATSENSEMBLUCSC | 146 | 4 | 23600 | tss | 0.376429 | 0.4969653 | Not queried | 1.40 | 4.86e-01 | 0.5640816327 |
|  | 1 | 166853278 | 166853812 | PDFSTATSENSEMBLUCSC | 535 | 6 | 3940 | island | 0.141660 | 0.5108695 | Not queried | 1.40 | 9.37e-01 | 0.9445195058 |
|  | 10 | 47083283 | 47083504 | PDFSTATSENSEMBLUCSC | 222 | 8 | 5063 | tss | 0.084251 | 0.0217117 | Not queried | 1.40 | 2.34e-01 | 0.4128374386 |
|  | 6 | 75915730 | 75915885 | PDFSTATSENSEMBLUCSC | 156 | 9 | 35074 | tss;island | 0.082749 | 0.1456539 | Not queried | 1.40 | 7.96e-01 | 0.8275104336 |
|  | 1 | 1309161 | 1309641 | PDFSTATSENSEMBLUCSC | 481 | 4 | 33 | gene;island | 0.589247 | 0.7460055 | Not queried | 1.40 | 6.86e-01 | 0.7223604052 |
